# Supplementary material for: Association of meteorological parameters with intussusception in children aged under 2 years: results from a multisite bidirectional surveillance over 7 years in India
Source: BMJ Open. 2021 May 25;11(5):e043687. doi: 10.1136/bmjopen-2020-043687 (PMC8154980; doi:10.1136/bmjopen-2020-043687)
Supplement: Supplementary data [file bmjopen-2020-043687supp001.pdf]

Title: Association of meteorological parameters with intussusception in children aged under-two years: Results from a multisite bidirectional surveillance over seven years in India

Supplementary Documents

Index

Sl no	Details
1	Supplementary Figure 1: The study sites and their location on India map indicating the study regions
2	Supplementary Table 1: The International Classification of Diseases (ICD) codes for review of the cases from medical records
3	Supplementary Table 2: The seasons and months at the sites according to the regions
4	Supplementary Table 3: The monthly median (and IQR) meteorological data during 2010 and 2017 for regions
5	Supplementary Table 4: Monthly median (and IQR) meteorological data during 2010 and 2017 at the study sites
6	Supplementary Figure 2: The pattern of monthly meteorological parameters across the regions
7	Supplementary Figure 3: The pooled monthly (SF3a) and seasonal (SF3b) distribution of intussusception in children during July 2010 to September 2017
8	Supplementary Table 5. Pearson correlation analysis between monthly intussusception cases and monthly median meteorological parameters for the study sites
9	Supplementary Table 6. Spearman's Rank correlation analysis between monthly intussusception cases and monthly median meteorological parameters for the study sites
10	Supplementary Table 7. Regression analysis of the association between the intussusception cases and meteorological parameters for the study sites
11	Supplementary Table 8. Intussusception cases in children at different temperature ranges at pooled and regional level
12	Supplementary Table 9. Variations in the intussusception cases between the different temperature categories by Kruskal-Wallis H test
13	Supplementary Figure 4: Association of monthly intussusception cases with mean monthly temperatures
14	Supplementary Figure 5: Association of monthly intussusception cases with mean monthly humidity
15	Supplementary Figure 6: Association of monthly intussusception cases with mean monthly wind speed
16	Supplementary Figure 7: Association of monthly intussusception cases with mean monthly sunshine
17	Supplementary Figure 8: Association of monthly intussusception cases with mean monthly rainfall
18	Supplementary Table 10. Factorial analysis-of-variance of intussusception in children with season and age groups
19	Supplementary Table 11. Factorial analysis-of-variance of intussusception in children with season and regions

20	Supplementary Table 12. Multiple comparisons of seasonal intussusception in children in different seasons pooled for all sites
21	Supplementary Table 13. Multiple comparisons of seasonal intussusception in children in different seasons at regional level
22	Supplementary Table 14. Multiple comparisons of seasonal intussusception in children in different seasons for different age groups

Supplementary Figure 1: The study sites and their location on India map indicating the study regions

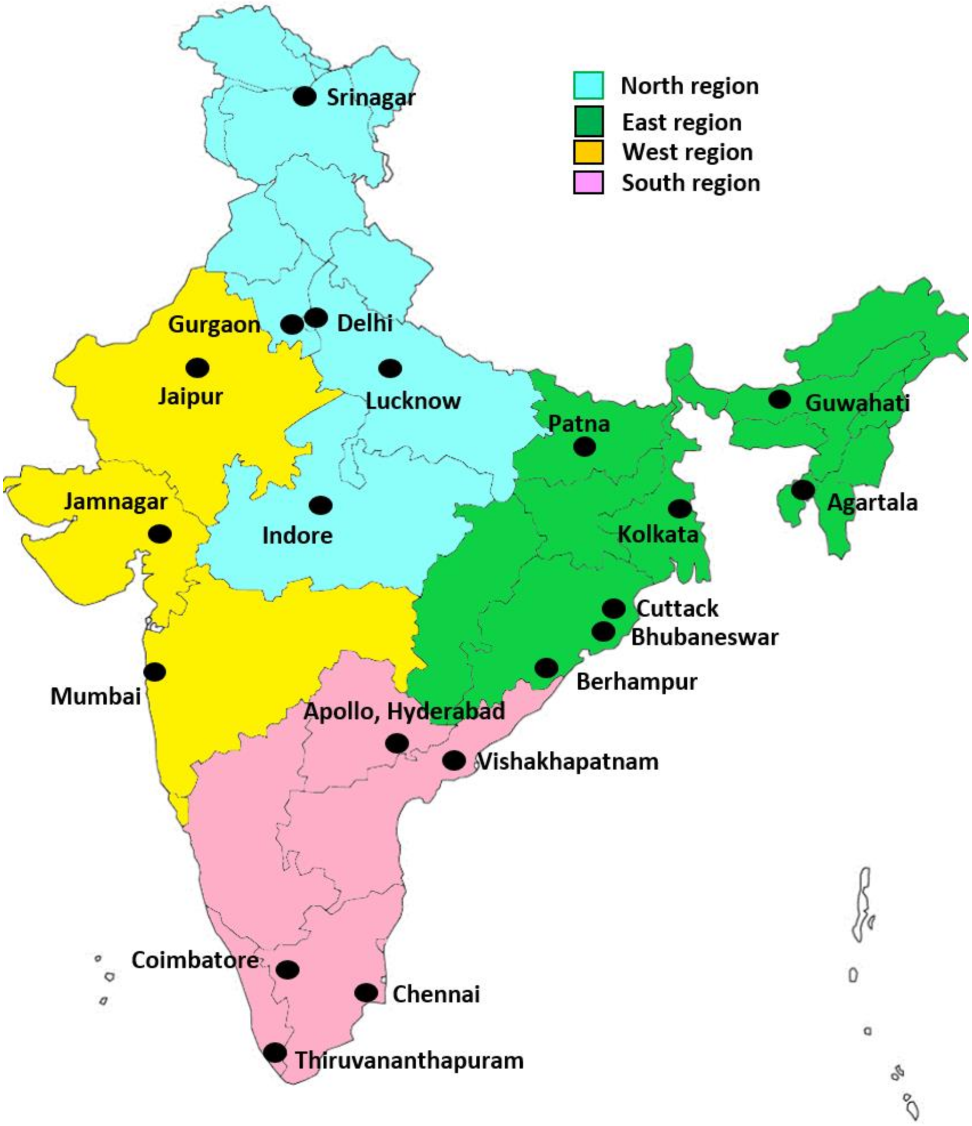

Note: The map has been prepared for use in the research project. The corresponding author is the creator of the image.

Das MK. *BMJ Open* 2021; 11:e043687. doi: 10.1136/bmjopen-2020-043687

Supplementary Table 1: The International Classification of Diseases (ICD) codes for review of the cases from medical records

Clinical conditions considered as suspected cases	Codes	
	ICD 10	ICD 9
Intussusception	K56.1	560.0
Volvulus	K56.2	560.2
Gallstone ileus	K56.3	560.31
Other impaction of intestine	K56.4	560.30
Intestinal adhesions with obstruction	K56.5	560.81
Other and unspecified intestinal obstruction or Acute intestinal obstruction or Subacute intestinal obstruction	K56.6	560.9
Ileus, unspecified	K56.7	560.1
Paralytic ileus	K56.0	
Acute abdomen	R10.0	789.0
Blood in stool with vomiting	K92.1/K92.2 with R11.2	578.1/578.9 with 787.01

Supplementary Table 2: The seasons and months at the sites according to the regions

Region/ Site (state)	Jan	Feb	Mar	Apr	May	Jun	Jul	Aug	Sep	Oct	Nov	Dec
North region												
Srinagar (Jammu & Kashmir)	W	W	W	Sp	Sp	Su	Su	M	M	A	W	W
New Delhi (Delhi)	W	Sp	Sp	Su	Su	Su	M	M	M	A	A	W
Gurgaon (Haryana)	W	Sp	Sp	Su	Su	Su	M	M	M	A	A	W
Lucknow (Uttar Pradesh)	W	W	Sp	Su	Su	Su	M	M	M	A	A	W
Indore (Madhya Pradesh)	W	W	Sp	Su	Su	Su	M	M	M	A	A	W
South region												
Vishakhapatnam (Andhra Pradesh)	W	W	Su	Su	Su	M	M	M	M	A	A	W
Hyderabad (Telangana)	W	Sp	Su	Su	Su	M	M	M	M	A	A	W
Coimbatore (Tamil Nadu)	W	Sp	Su	Su	Su	M	M	M	M	A	A	W
Chennai (Tamil Nadu)	W	Sp	Su	Su	Su	M	M	M	M	A	A	W
Trivandrum (Kerala)	W	Sp	Su	Su	Su	M	M	M	M	A	A	W
East region												
Patna (Bihar)	W	Sp	Sp	Su	Su	Su	M	M	M	A	A	W
Bhubaneswar (Odisha)	W	Sp	Su	Su	Su	M	M	M	A	A	A	W
Cuttack (Odisha)	W	Sp	Su	Su	Su	M	M	M	A	A	A	W
Berhampur (Odisha)	W	Sp	Su	Su	Su	M	M	M	A	A	A	W
Kolkata (West Bengal)	W	Sp	Su	Su	Su	M	M	M	A	A	A	W
Guwahati (Assam)	W	W	Su	Su	Su	M	M	M	A	A	A	W
Agartala (Tripura)	W	W	Su	Su	Su	M	M	M	A	A	A	W
West region												
Jaipur (Rajasthan)	W	W	Sp	Su	Su	Su	M	M	M	A	A	W
Jamnagar (Gujarat)	W	Sp	Su	Su	Su	M	M	M	M	A	A	W
Mumbai (Maharashtra)	W	Sp	Su	Su	Su	M	M	M	M	A	A	W

Note: The seasons and colour codes used

A Autumn M Monsoon Sp Spring Su Summer W Winter

Das MK. *BMJ Open* 2021; 11:e043687. doi: 10.1136/bmjopen-2020-043687

Supplementary Table 3: The monthly median (and IQR) meteorological data during 2010 and 2017 for regions

Region/ Months	Temperature (°C)	Rainfall (mm)	Wind speed (Kmph)	Humidity (%)	Sunshine (hours)
North region					
January	14 (11, 16)	5.7 (0.5, 13.2)	8.5 (7.1, 9.8)	54 (47, 61)	223 (217.5, 232.5)
February	17 (14, 20)	6.9 (0.5, 22.6)	9.6 (8.1, 10.3)	47 (39, 62)	266 (239, 277.5)
March	23 (21, 25)	4.9 (1.1, 19.9)	9.9 (9, 10.5)	37 (26, 45)	301 (291.5, 308.5)
April	31 (28, 32)	3.9 (1.3, 12.6)	10.5 (8.1, 12.2)	23 (18, 28)	337.5 (315, 345)
May	35 (34, 36)	3.3 (1.1, 14.3)	10.7 (8.9, 12.3)	22 (19, 30)	386 (383, 387.5)
June	35 (31, 37)	22 (8.1, 48.8)	11.1 (9.1, 12.9)	40 (31, 46)	361.5 (346.5, 369.5)
July	31 (26, 33)	72.5 (30, 126.7)	9.6 (8, 12.3)	59 (54, 69)	331.8 (279.3, 367)
August	29 (24, 31)	55.1 (26.3, 93.7)	9 (7, 10.8)	66 (56, 80)	337.8 (298.8, 359.3)
September	28 (25, 30)	31.6 (13.4, 58.1)	8.4 (7.2, 9.2)	56 (49, 76)	286.3 (269.8, 300)
October	26 (25, 27)	1.5 (0.2, 12.2)	6.5 (5.9, 7.2)	42 (34, 50)	250 (244, 262.5)
November	21 (20, 22)	0.1 (0, 3.2)	7.5 (6, 8.2)	33 (31, 39)	225 (222, 225)
December	16 (14, 18)	0.6 (0, 4.4)	7.8 (6.7, 8.8)	39 (35, 45)	231 (225.5, 232.5)
South region					
January	23 (23, 25)	2.8 (1.1, 7.7)	9.7 (8.6, 12.1)	67 (63, 71)	277.5 (274.5, 296.5)
February	25 (24, 26)	1.5 (0.4, 8)	10.1 (8.8, 12.2)	65 (51, 71)	277.5 (272.5, 286)
March	28 (27, 28)	10.1 (3.4, 29.8)	10.9 (8.9, 13.6)	66 (50, 71)	299.5 (294, 308.5)
April	30 (29, 31)	7.8 (2.9, 32.2)	12.1 (9.7, 17.1)	69.5 (52, 73)	317.5 (292.5, 333.5)
May	31 (29, 32)	20.2 (9.1, 30.5)	14.6 (12.9, 17.6)	64 (59, 71)	353.5 (317, 378)
June	28 (27, 31)	50.7 (16.5, 97.8)	18 (17.2, 18.5)	65 (62, 70)	291 (268.5, 319.5)
July	28 (26, 29)	43 (1.7, 78.5)	18.5 (17, 20.1)	68 (63, 74)	283.3 (255.5, 308.5)
August	27 (26, 29)	58.1 (7.9, 92.3)	16.2 (14.4, 17.6)	72 (66, 77)	275.5 (245.5, 296.5)
September	27 (26, 29)	61.7 (34.1, 99.9)	13.8 (12.3, 16.3)	72 (67, 76)	258 (239, 288)
October	27 (26, 28)	61.9 (44.4, 117.6)	10.6 (9.4, 12.2)	71 (68, 75)	246.8 (224.5, 267)
November	25 (24, 27)	34.8 (19.2, 108.9)	9.6 (7.2, 13.5)	72 (69, 75)	205.5 (191, 213)
December	24 (23, 26)	15 (3, 37.9)	10 (8.5, 14.3)	70.5 (66, 74)	225.5 (216, 265.5)
East region					
January	20 (17, 22)	1.7 (0.1, 5.8)	7.2 (6.4, 8.3)	60 (54, 64)	227 (224.5, 231)
February	23 (20, 24)	2.7 (1, 8.7)	8.2 (7, 9)	54 (49, 61)	210 (207, 237)
March	27 (25, 28)	8.5 (2.9, 19.4)	10 (8.9, 11)	56 (46, 60)	291.5 (287.5, 301)
April	31 (29, 32)	12.3 (3.5, 32.1)	12.5 (10.4, 14.6)	60 (54, 66)	355.5 (294.5, 370.5)
May	32 (30, 33)	25.2 (13.9, 54.5)	13.3 (11.2, 15)	66 (60, 69)	365.5 (347, 374.5)
June	32 (29, 33)	79.8 (53.9, 122.3)	12 (10.6, 13.1)	66 (62, 76)	307.5 (292.5, 327)
July	30 (28, 30)	118 (82.6, 175.2)	11.3 (9.8, 12.4)	73 (69.5, 83.5)	276.8 (250, 288.5)
August	29 (28, 30)	100 (70.8, 154.9)	10 (8.9, 11.1)	78 (73.5, 82)	286 (258, 305)
September	28 (28, 29)	89.9 (65.7, 133)	8.3 (7.3, 9.4)	79.5 (75, 83)	252.5 (236.3, 262)
October	26 (25, 27)	44.7 (24.8, 87.9)	6.7 (5.9, 7.6)	77 (71, 82)	287 (271.5, 295)
November	23 (21, 24)	3.6 (0.4, 20.4)	6.4 (5.7, 7.3)	66 (60, 77)	270 (256, 292.5)
December	21 (18, 22)	1 (0.2, 6.7)	7.1 (6.1, 8)	59 (50, 70)	229.5 (222.5, 240)
West region					
January	22 (15, 24)	0.1 (0, 0.9)	10 (8.5, 14.4)	48 (44, 51)	310 (255, 310)
February	24 (20, 25)	0.4 (0, 1)	10.8 (9.9, 14.5)	45 (44, 53)	278.5 (275.5, 281)
March	27 (25, 28)	0.2 (0, 1.2)	10.7 (10.1, 15.9)	47 (29, 53)	307.5 (305.5, 308.5)
April	29 (29, 31)	0.4 (0, 1.7)	11.2 (10, 18.3)	56 (20, 61)	300 (300, 315.5)

Region/ Months	Temperature (°C)	Rainfall (mm)	Wind speed (Kmph)	Humidity (%)	Sunshine (hours)
May	31 (30, 36)	0.6 (0.1, 5.5)	14.3 (10.7, 23.9)	65 (21, 68)	355 (307, 381.5)
June	31 (29, 36)	48.9 (9.7, 156.7)	16.7 (13.9, 21.7)	68 (34, 75)	311.5 (252, 359.5)
July	29 (28, 32)	121.3 (58.1, 391.4)	17.3 (13.9, 23.8)	77.5 (56.5, 82.5)	194 (158.5, 338.8)
August	28 (27, 29)	118.5 (60, 213.5)	14.1 (11.4, 22.1)	79 (73.5, 84)	196.8 (166.5, 278)
September	28 (27, 29)	70.6 (19.4, 133.8)	11.6 (9.8, 17.8)	74 (62.5, 80)	253.5 (229, 274.8)
October	28 (28, 29)	5.9 (0.5, 26.1)	9 (8.1, 12.5)	51 (41, 67)	296.5 (265, 308.5)
November	27 (23, 28)	0.1 (0, 2.3)	9 (8, 13)	44 (34, 53)	230 (225, 251.5)
December	24 (17, 25)	0 (0, 0.5)	9.8 (8.4, 14.5)	43 (39, 49)	252.5 (232.5, 293.5)

Note: Temperature (°C); Rain: Rainfall (millimetre, mm); Wind Speed (miles/second); Humidity (%); Sunshine (in hours/month)

Supplementary Table 4: Monthly median (and IQR) meteorological data during 2010 and 2017 at the study sites

Region/ Place (State) and months	Temperature (°C)	Rainfall (mm)	Wind speed (Kmph)	Humidity (%)	Sunshine (hour)
1. North region					
1.1 Srinagar (Jammu and Kashmir)					
January	-3 (-4,-1)	28.4 (16.8,41.9)	5.8 (5.3,6.3)	58 (44,69)	209.5 (205.5,218)
February	0 (-2,2)	69.9 (56.8,89)	5.7 (5.4,6.1)	60 (59,68)	241 (220,261.5)
March	4 (3,5)	63.4 (44.2,107)	5.3 (4.7,6.5)	60 (57,63)	282 (232.5,296)
April	8 (7,9)	45.3 (36.7,84.2)	4.6 (4.4,5)	55 (52,57)	330 (301.5,336)
May	13 (11,14)	31.6 (16.3,57.3)	4.3 (3.7,5.1)	49 (46,50)	387.5 (378.5,387.5)
June	17 (16,18)	15.8 (8.1,33.4)	4.2 (3.8,4.4)	46 (41,47)	372 (364.5,375)
July	19 (18.5,19)	22.6 (13,50.5)	5.4 (4.7,6)	57.5 (57,59)	387.5 (381.5,387.5)
August	18 (18,19)	25 (20.6,34.1)	5 (3.8,5.4)	59.5 (56,65)	371 (368,372.5)
September	15 (14,15)	9 (5.6,48.8)	3.9 (3.6,4.1)	50.5 (48,52.5)	300 (297.8,300)
October	9 (8,11)	10.5 (4.1,15.8)	3.3 (2.8,3.9)	40 (34,41)	262.5 (258.5,262.5)
November	4 (3,5)	8 (1.4,12.1)	4.6 (4.1,6)	33 (31,36)	222 (209.5,225)
December	0 (-1,1)	14.9 (5.3,24.9)	5.8 (5.4,6.7)	31 (27,42)	221.5 (211.5,228)
1.2 Delhi (Delhi) and 1.3. Gurgaon (Haryana)					
January	13 (11,14)	1 (0.3,5.7)	8.5 (7.9,9.2)	54 (53,62)	223 (220.5,228.5)
February	16 (15,18)	5.3 (0.2,7.8)	9.8 (8.9,10.1)	53 (43,63)	269.8 (266,277.5)
March	22 (21,23)	2.9 (1.1,6.5)	9.9 (9.2,10.4)	41 (33,43)	300 (292,303)
April	30 (29,32)	5.3 (2.7,9.8)	10.5 (8.4,10.7)	23 (17,27)	341.8 (331.5,344.5)
May	36 (35,36)	2.4 (0.9,6.7)	10.2 (9.8,10.7)	20.5 (15,22)	386 (383,386)
June	36 (36,38)	20.7 (8.5,30.9)	10.1 (9.8,11.8)	32 (23,36)	363.5 (361.5,369.5)
July	32.5 (32,34)	61.2 (30.7,98.2)	8.9 (8.1,10.2)	53.5 (51,61.5)	345 (331.8,365)
August	31 (30,32.5)	47.8 (28.5,78.9)	8.4 (7.7,9.7)	63.5 (54,72.5)	339.8 (329,349)
September	30.5 (29,31)	27.2 (11.9,61.1)	8.2 (7.7,8.6)	49.5 (47,67)	279.5 (272.3,294)
October	27 (26,28)	0.5 (0,1.1)	6.5 (6.2,7)	34 (31,43)	250 (248.5,250)
November	21 (21,22)	0 (0,0.2)	8.1 (6.5,8.2)	31 (27,40)	225 (223.5,225)
December	16 (15,17)	0.3 (0,1.5)	8.3 (6.9,9)	39 (35,46)	230.5 (225.5,231)
1.4. Lucknow (Uttar Pradesh)					
January	15 (14,16)	8.1 (4.7,9.6)	9.8 (8.6,10.1)	54 (53,58)	224 (219,229.5)
February	18 (17,20)	6.9 (0.5,22.6)	10.1 (9.8,11.3)	52 (42,58)	230.5 (224.5,239)
March	25 (24,25)	3.1 (2.6,10.5)	11.2 (11.1,12)	35 (26,37)	309.5 (303.5,315)
April	32 (32,34)	2 (1.3,3.9)	12.6 (12,13.4)	21 (18,26)	373.5 (363,375)
May	36 (36,37)	5.2 (0.2,6.9)	11.4 (11.2,12.3)	21 (18,25)	384.5 (380,386)
June	37 (34,39)	13.4 (3.2,100.6)	11.6 (11.2,12.9)	32 (25,45)	346.5 (321.5,362.5)
July	32 (31,33)	127.8 (83.6,151.1)	11.1 (10.2,12.3)	63 (57,69)	304.3 (289,321)
August	30 (30,31.5)	76.5 (50.8,95.9)	10.7 (9.4,11.1)	70.5 (58,74.5)	313.8 (304,344.8)
September	29.5 (28.5,30.5)	30.9 (21.1,61.8)	9.1 (8.5,9.6)	64.5 (55,75.5)	316 (301.3,328.3)
October	26 (25,27)	7.4 (0.2,31.3)	6.9 (6.1,7.9)	50 (42,62)	227.5 (225.5,238.5)
November	22 (21,22)	0 (0,0.7)	7.4 (6.9,8.5)	35 (34,48)	225 (218,225)
December	17 (17,18)	0.6 (0,1.6)	8.5 (7.6,9.9)	41 (38,46)	232.5 (231,232.5)
1.5 Indore (Madhya Pradesh)					
January	18 (17,19)	0.8 (0.4,9.1)	9.2 (8,10.5)	44 (32,47)	281 (277.5,282.5)
February	22 (21,23)	1 (0.2,11.1)	9.6 (8.6,11)	32 (29,39)	280 (277,290)
March	27 (27,27)	2.9 (0,6.4)	10 (9.8,10.4)	21 (18,26)	307 (301,310)
April	32 (32,33)	0.6 (0.2,2.8)	12.3 (11.2,14.4)	18 (17,21)	315 (310,317.5)
May	35 (34,35)	1.9 (0.6,2.7)	15.3 (14.7,16.8)	24 (22,30)	386 (384.5,387.5)
June	31 (31,32)	48.9 (29.6,72.2)	15.4 (14.6,16.9)	51 (47,57)	341 (294,352)

Region/ Place (State) and months	Temperature (°C)	Rainfall (mm)	Wind speed (Kmph)	Humidity (%)	Sunshine (hour)
July	27 (26,27)	124.5 (83.8,186.7)	15.5 (12.6,17.3)	80 (75,81.5)	201.5 (178.8,239)
August	25.5 (24.5,26)	101.7 (78.8,132.8)	12.1 (11.4,14.9)	88.5 (80,90)	166.5 (144.8,208.3)
September	25.5 (25,26)	52.2 (41,62.1)	9.7 (8.6,10.5)	77 (75,81)	253.5 (241.5,267.3)
October	26 (25,26)	2.3 (0.3,31.1)	7.2 (6.6,8.3)	45 (40,58)	271 (254.5,272.5)
November	23 (22,24)	0.3 (0,2.9)	7.6 (6.4,8)	37 (33,39)	225 (220.5,225)
December	19 (19,20)	0.1 (0,1.2)	8.1 (7.4,8.8)	36 (34,40)	232.5 (229.5,232.5)
2. East region					
2.1 Bhubaneswar and 2.2. Cuttack (Odisha)					
January	22 (21,22)	1.5 (0.1,5.6)	7.2 (6.4,7.4)	56.5 (56,60)	224.5 (221.5,228)
February	24.5 (24,25)	2 (0.5,8.9)	8.2 (7.1,9.3)	56 (53,62)	209.3 (202.5,210)
March	28 (28,29)	6.7 (3.6,16.6)	10.9 (9.4,11.5)	57.5 (54,62)	287.3 (275.5,290)
April	31 (31,32)	5.4 (1.6,12.3)	15 (12.4,17.2)	60.5 (57,66)	366.8 (360,372)
May	32 (32,33)	22.3 (15.4,26.9)	14.9 (13.9,15.8)	64.5 (63,67)	366.8 (362.5,375.5)
June	32 (32,33)	80 (60.3,114.7)	12.2 (12,13.7)	62.5 (62,66)	300.8 (292.5,322.5)
July	30 (30,30)	118 (80.3,137.4)	11.3 (10.1,12.1)	72 (69,73)	269.8 (253.3,283.8)
August	29 (29,30)	92.6 (67,133.3)	10.4 (9.2,11.4)	74.5 (71.5,77.5)	285 (262.3,296)
September	29 (28,29)	89.9 (67.3,112.9)	8.7 (7.3,9.8)	77 (74.5,79.5)	248.3 (234.3,257.3)
October	27 (26,27)	50.1 (25.7,89.9)	7.4 (6.7,7.9)	74 (69,82)	285.5 (263,290)
November	23.5 (23,25)	9.1 (1,28.3)	6.6 (6.2,7.2)	64.5 (60,66)	261.8 (256,268.5)
December	21 (21,22)	4.7 (0.3,11.8)	6.9 (6.6,8)	57 (53,59)	223.8 (207,229.5)
2.3 Berhampur (Odisha)					
January	22 (21,22)	1.6 (0.6,5.6)	6.9 (6.6,7.6)	66 (63,69)	224.5 (221.5,228)
February	24 (24,24)	3.6 (2.1,15.7)	8.2 (7.2,8.7)	62 (61,68)	210 (205.5,210)
March	28 (27,28)	8.5 (1.3,20.6)	10.8 (9.8,11.7)	61 (59,64)	288.5 (275.5,291)
April	30 (30,31)	9.7 (2,20.6)	13.9 (12.4,14.6)	63 (58,67)	367.5 (361.5,372)
May	32 (32,32)	13.3 (7.1,28.9)	13.3 (12.5,13.7)	67 (64,68)	368 (364,380)
June	31 (31,32)	60.7 (48.2,79.8)	11.3 (11.1,11.8)	67 (64,69)	307.5 (300,324.5)
July	29 (29,30)	82.9 (65.6,107.6)	11.1 (10.5,11.4)	73 (71,74.5)	283.5 (279.3,289)
August	29 (29,29)	93.5 (72.6,119.7)	9.4 (9.1,10)	74.5 (73,78)	294.3 (272,302.5)
September	28.5 (28,29)	91.1 (67,116)	8.1 (7.5,8.8)	76 (75,77.5)	254.8 (250.3,261.5)
October	27 (26,27)	44.9 (33.2,132.7)	8.1 (6.6,9)	72 (70,80)	289.5 (263,295)
November	24 (24,24)	44.5 (0.4,59.7)	7 (6.6,8.3)	67 (63,71)	262 (256,271.5)
December	22 (22,22)	3.3 (1,30.7)	7.8 (7,8.2)	62 (60,67)	225 (207,229.5)
2.4. Kolkata (West Bengal)					
January	20 (19,21)	0.1 (0,7.7)	9.1 (8.6,9.9)	44 (40,52)	228 (225,231)
February	23 (23,24)	4.4 (0.8,7.8)	9 (8.1,9.3)	45 (40,46)	211 (204.5,212.5)
March	28 (27,28)	8.7 (1.7,19.2)	10 (9.4,10.2)	50 (46,57)	304 (295,308.5)
April	31 (31,33)	12.5 (5.2,37.2)	14.2 (10.3,15.2)	58 (54,63)	300 (287.5,312)
May	33 (32,33)	16.2 (11.2,28.5)	15 (13.7,17.7)	61 (59,67)	371 (369.5,377.5)
June	32 (31,33)	63.7 (52.7,92.7)	13.1 (12.8,15.8)	65 (64,72)	318.5 (290.5,327)
July	30 (29.5,30)	119.7 (102.1,171.9)	12.6 (11.5,13.2)	76 (74,78)	258.8 (243.3,271.3)
August	29 (29,30)	102.7 (86,153.2)	11 (10.3,11.1)	80.5 (76,82)	277.3 (247.8,284.5)
September	28 (28,29.5)	81.4 (74.9,111.4)	9.1 (8.1,10.3)	81.5 (77,85)	236.3 (226.8,241.3)
October	27 (26,27)	27.2 (23.6,34.9)	7.3 (6.5,8.7)	72 (71,80)	280 (265.5,287.5)
November	24 (23,25)	3.3 (0.3,16.7)	7.5 (6.1,9.3)	57 (53,65)	292.5 (284,297)
December	21 (20,22)	0.1 (0,1.8)	8.7 (8.5,9.5)	47 (41,50)	230 (218,232.5)
2.5. Patna (Bihar)					
January	16 (15,17)	5.8 (0.8,8.3)	9.5 (9.4,10)	54 (51,63)	229.5 (222.5,232.5)
February	19 (18,21)	0.9 (0.4,2.7)	10.4 (9.7,11.8)	49 (47,54)	207.5 (202.5,213)
March	26 (25,26)	2.9 (1.6,5.5)	11.1 (10.1,11.7)	32 (30,33)	303.5 (291.5,305)

Region/ Place (State) and months	Temperature (°C)	Rainfall (mm)	Wind speed (Kmph)	Humidity (%)	Sunshine (hour)
April	33 (32,33)	6.5 (2.4,22.2)	12.3 (11.4,14.2)	28 (20,31)	373.5 (358.5,375)
May	35 (35,37)	23.2 (5,41.1)	13 (12.3,13.6)	35 (29,39)	377 (366.5,386)
June	36 (34,37)	47.6 (21.1,58.6)	11.9 (11.5,13.6)	42 (42,51)	337 (327,355)
July	32 (31,32.5)	150.4 (120.4,160.4)	12 (11.7,14)	65.5 (60.5,68.5)	283 (257.5,312.5)
August	31 (31,32)	63.4 (38.5,138.2)	10.6 (9.3,11.7)	65 (61.5,71.5)	318.5 (293,329.8)
September	29 (28.5,30.5)	66.7 (32.4,116.6)	8.7 (8.4,10.4)	67 (60.5,80)	273 (249.5,282.5)
October	26 (25,27)	29.1 (8.1,54.9)	6.8 (5.9,7.5)	63 (51,78)	297 (271.5,304)
November	22 (21,23)	0 (0,0.3)	6.8 (6.3,8.1)	45 (39,61)	232.5 (231,235)
December	18 (18,19)	0 (0,0.7)	8 (7.5,9.1)	49 (38,54)	231 (225,232.5)
2.6. Guwahati (Assam)					
January	17 (15,17)	3.9 (0.2,10.3)	3.9 (3.8,4.3)	67 (64,76)	228 (225,231)
February	20 (19,20)	4.9 (2.3,10.2)	4.2 (4,4.5)	56 (54,62)	245 (242,253.5)
March	24 (23,24)	14.1 (1.4,22.9)	4.9 (4.6,4.9)	46 (42,58)	299.5 (295,310)
April	26 (25,27)	39.7 (15.1,80.8)	4.5 (4.4,4.8)	62 (50,68)	292.5 (280.5,300)
May	27 (27,28)	83.1 (54.5,122.4)	4.2 (3.8,5)	69 (63,73)	344 (336.5,357)
June	28 (27,29)	236.2 (191.4,326.2)	3.8 (3.6,4.3)	81 (76,84)	323.5 (292.5,334)
July	27.5 (27,28)	344.4 (224.2,407.4)	3.8 (3.5,4.1)	87 (83.5,88.5)	317.8 (274.5,332.8)
August	28 (27.5,28)	235.2 (214.1,276.4)	3.8 (3.7,4.2)	84 (82,85)	297.3 (283,310.5)
September	27 (26.5,27)	159.3 (143.5,217.6)	3.4 (3.1,4)	84 (81.5,85)	272 (257.8,274.8)
October	24 (24,24)	48.8 (12.8,72.7)	3.6 (3.4,4.2)	79 (77,81)	294 (283,305.5)
November	20 (20,20)	4.5 (1,7.3)	3.8 (3.7,4.5)	78 (71,80)	294 (292,297)
December	17 (17,18)	2.5 (1.1,9)	4 (3.8,4.2)	74 (73,77)	250.5 (240,253.5)
2.7. Agartala (Tripura)					
January	18 (17,19)	0.3 (0.1,2.2)	6.7 (6.5,7.2)	61 (58,69)	231 (228,232.5)
February	21 (20,22)	5.6 (1.7,10.5)	7.2 (7,8.6)	53 (51,57)	246 (237,250.5)
March	25 (24,26)	22.6 (11.8,53.2)	8.9 (8.8,9.5)	57 (51,64)	295.5 (288,301)
April	28 (28,29)	113.4 (38.1,141.2)	11.1 (8.5,12.5)	67 (64,73)	266.5 (246,297)
May	29 (28,30)	113.8 (73.4,194.5)	9.7 (9.1,11.5)	74 (74,80)	307.5 (299.5,320)
June	29 (28,29)	99.2 (72.8,110.5)	11.1 (10.6,13.4)	82 (79,85)	286.5 (282.5,303.5)
July	28 (28,28)	73 (50.4,103.5)	12.1 (10.4,13.4)	84.5 (83.5,85.5)	261.8 (244.8,276)
August	28 (27.5,28)	78.4 (61.2,101.3)	10.6 (9.4,11.1)	86.5 (82,87.5)	249.3 (231.8,271)
September	27 (27,28)	57.1 (43.3,144.7)	8.4 (7.5,9.4)	86.5 (82,89)	249 (225,259)
October	25 (25,26)	55.3 (23,121.8)	5.9 (4.9,6)	85 (82,87)	284.5 (271,293.5)
November	21 (21,22)	5.2 (0.2,31)	5.5 (5.3,6.1)	78 (77,79)	300 (282,300)
December	18 (18,20)	0.7 (0.2,6.7)	6.1 (5.1,6.2)	71 (70,73)	264.5 (258.5,267.5)
3. South region					
3.1. Vishakhapatnam (Andhra Pradesh)					
January	23 (23,24)	3.5 (1.3,6.1)	10.6 (9.4,11.1)	68 (67,72)	277.5 (274.5,277.5)
February	25 (24,25)	1.4 (0.9,4.2)	10.1 (9.4,10.8)	68 (65,70)	280 (277.5,288.5)
March	27 (27,28)	3.4 (3.3,16.2)	13.6 (11.5,14)	70 (65,70)	308.5 (298,310)
April	29 (29,30)	5 (1.3,12.6)	17.4 (16.7,19.1)	71 (70,74)	330 (320,335)
May	31 (31,31)	8.6 (3.5,18)	17 (15.9,18.5)	71 (70,72)	378 (371,381.5)
June	31 (31,31)	91 (40.8,129.1)	16.9 (14.3,17.3)	70 (66,71)	274.5 (268.5,306)
July	29.5 (29,30)	77.6 (60.1,105.1)	18.5 (16.2,20.1)	73 (71.5,74.5)	252.5 (226.8,289.8)
August	29 (29,30)	111.7 (96.7,120.4)	13.8 (13.5,16.6)	74.5 (73,77)	241 (234.5,263.5)
September	29 (29,29)	127 (104.7,164.2)	11.8 (11.7,13)	76 (75.5,77)	234.5 (226.3,243.8)
October	27 (27,28)	90.4 (50.2,182.7)	11.7 (9.1,13.2)	72 (69,77)	222 (205.5,236.5)
November	25 (25,26)	46.2 (19.2,144.8)	12.1 (11.5,15.1)	70 (66,71)	207 (202,217.5)
December	24 (24,24)	4.2 (2.6,14.6)	13.8 (12,14.4)	67 (66,70)	225.5 (222,228)

Region/ Place (State) and months	Temperature (°C)	Rainfall (mm)	Wind speed (Kmph)	Humidity (%)	Sunshine (hour)
3.2. Hyderabad (Telangana)					
January	22 (21,23)	0.5 (0.1,1.5)	12.1 (10.1,12.6)	47 (46,51)	277.5 (274.5,277.5)
February	24 (24,25)	0 (0,1.6)	12.4 (10.6,12.9)	41 (38,45)	280 (277.5,288.5)
March	28 (28,29)	3.4 (0.1,10.6)	11.1 (10.3,11.9)	34 (32,39)	308.5 (298,310)
April	32 (31,33)	2.9 (1.5,5.3)	9.7 (9.2,11.9)	33 (27,35)	330 (320,335)
May	34 (33,35)	9.4 (7.3,17.6)	12.9 (12,14.4)	30 (28,34)	378 (371,381.5)
June	29 (28,29)	111.6 (33.3,131.2)	18.7 (18.1,20.7)	57 (49,63)	274.5 (268.5,306)
July	26 (26,27.5)	66.4 (47.1,81)	20.7 (18.4,23)	72.5 (65,75)	252.5 (226.8,289.8)
August	26 (25,26.5)	68.6 (36,77.7)	16.6 (15.3,17.5)	76 (70,78)	241 (234.5,263.5)
September	26 (25,26)	51.9 (40.3,74.1)	12.8 (11.3,13.9)	74.5 (70.5,78.5)	234.5 (226.3,243.8)
October	24 (24,25)	35.9 (14.9,45.2)	10.1 (9.2,10.6)	65 (59,70)	222 (205.5,236.5)
November	22 (21,23)	3.1 (0.5,18.8)	10.2 (9.5,10.8)	60 (51,62)	207 (202,217.5)
December	21 (20,22)	0.3 (0.2,1.1)	10.3 (10.1,11.3)	50 (49,53)	225.5 (222,228)
3.3. Coimbatore (Tamil Nadu)					
January	23 (23,23)	2.2 (1.1,26.6)	7.4 (6.9,8.6)	63 (63,66)	290 (285,305.5)
February	25 (24,25)	3 (0.9,8.3)	7.8 (7.4,8.9)	55 (51,56)	277 (272,286)
March	28 (27,28)	26.7 (10.1,60.5)	7.1 (7.1,8.6)	53 (50,55)	299.5 (298,302.5)
April	30.5 (30,31)	8.9 (6.7,28.2)	9.2 (8.3,11.6)	52 (52,54)	297.5 (284.5,297.5)
May	30 (29,30)	29.7 (29.7,30.5)	12.4 (11.9,13.2)	59 (59,61)	334 (317,335)
June	27 (27,27)	4.9 (4.9,6.7)	18.1 (17.7,18.3)	64 (64,66)	301.5 (285.5,302)
July	27 (27,28)	0.2 (0,1.7)	18.5 (17.9,19.3)	63 (61,64)	291 (268.5,308.5)
August	27 (26,27)	3.1 (0.9,7.9)	16.1 (14.8,17.6)	65 (63,67)	291.5 (274.5,296.5)
September	27 (27,27)	15.6 (8.3,42.8)	16.3 (14.5,17.6)	65.5 (65,67)	278 (256.5,282)
October	26 (26,26)	53.2 (53.2,63.8)	9.7 (8.3,10.6)	68 (68,71)	268.3 (254.5,270)
November	24 (24,24)	32.6 (32.6,34.9)	6.1 (6.1,7)	72 (69,78)	205.5 (194,206.5)
December	23 (23,23)	14.3 (6,15.5)	7 (6.7,7.9)	71 (67,74)	242.3 (225.5,267)
3.4. Chennai (Tamil Nadu)					
January	25 (25,25)	7.7 (4.4,10.5)	15.4 (12.4,16.6)	73 (70,75)	267 (259.5,273.5)
February	25 (25,26)	1.5 (0.3,26)	13.2 (11.7,14)	72 (70,74)	275.5 (272.5,287)
March	28 (27,28)	1.8 (1.1,10.1)	14.8 (13.6,15.5)	72 (71,73)	301 (294,304)
April	30 (30,30)	7.2 (0.3,35.3)	18.9 (16.9,20.1)	72 (71,74)	344.5 (330,352.5)
May	32 (32,32)	18.6 (8.3,37.5)	18.9 (18.2,19.1)	66 (66,67)	381.5 (355,383)
June	31 (31,32)	37.8 (19,63.2)	17.4 (16.9,18.4)	62 (61,66)	336.5 (327.5,348.5)
July	30.5 (30,31)	37.9 (22.1,61.8)	16.8 (15.7,17.5)	65 (63,65.5)	343.5 (339,354.3)
August	30 (29,30)	75.4 (61.7,88.7)	15.2 (14.1,16)	69 (66.5,70.5)	339.3 (335.8,359.8)
September	29.5 (29,30)	101.1 (91.5,105.6)	13.3 (13,13.8)	72 (70,72)	333.5 (317,347.8)
October	29 (28,29)	102.6 (81.5,154.2)	12.2 (10.9,13.8)	73 (71,74)	252 (247,263.5)
November	27 (27,27)	60 (48.4,344.4)	17.3 (15.3,17.7)	74 (72,79)	201 (190.5,213)
December	26 (25,26)	84.8 (58.6,111.3)	18 (16.5,20)	76 (72,77)	199 (188.5,208)
3.5. Trivandrum (Kerala)					
January	26 (26,26)	3.2 (1.5,7.7)	8.7 (8.2,9.5)	70 (68,71)	299.5 (296.5,301)
February	26 (26,26)	6.1 (1.4,23)	8.8 (8.7,10.7)	71 (65,72)	278.5 (269.5,284)
March	28 (27,28)	37.8 (14.8,53.6)	9.8 (9.1,10.7)	71 (70,75)	293.5 (288,299.5)
April	28 (28,29)	57.3 (32.2,115.9)	12.2 (10.7,14.3)	79 (72,79)	278 (264,285)
May	28 (28,29)	24.9 (17.4,42.5)	15 (14.6,18)	79 (72,79)	252 (245.5,267.5)
June	27 (27,28)	82.2 (46.9,110.2)	20.1 (17.4,21.5)	78 (74,79)	243 (213,256)
July	26.5 (26,27.5)	65.6 (15.1,101.1)	21.1 (18.7,22.1)	78 (75,80)	266.3 (248.3,278.8)
August	26 (26,27.5)	33.6 (7.8,72.9)	18.8 (17,20.5)	80 (75.5,81)	264.5 (240.8,285.5)
September	26.5 (26,27)	61.7 (40.3,77.2)	17 (13.8,17.4)	80 (77,81.5)	256 (244,263.3)
October	27 (26,27)	117.9 (32.3,220.7)	11.4 (10.1,14.9)	78 (75,80)	266 (245.5,286)

Region/ Place (State) and months	Temperature (°C)	Rainfall (mm)	Wind speed (Kmph)	Humidity (%)	Sunshine (hour)
November	27 (27,27)	107.7 (47,140)	8.6 (8,9.1)	74 (72,76)	189 (174,205)
December	26 (26,27)	25.1 (15.7,37)	8.9 (8.6,9.6)	72 (72,75)	272.5 (267,280.5)
4. West region					
4.1. Mumbai (Maharashtra)					
January	24 (24,25)	0 (0,0.4)	10 (9.7,10.9)	52 (51,53)	310 (310,310)
February	25 (25,26)	0.4 (0,2.9)	10 (9.2,10.2)	54 (51,55)	280 (275.5,281)
March	28 (27,28)	0.6 (0,1.3)	10.5 (9.6,10.7)	53 (51,56)	308.5 (305.5,310)
April	29 (29,29)	0.1 (0,0.2)	9.7 (9.4,10.3)	63 (61,66)	300 (292.5,300)
May	30 (29,30)	1.3 (0.6,8)	10.5 (10.1,10.7)	72 (68,73)	305.5 (292,307)
June	29 (29,29)	292.8 (156.7,370)	16 (14.6,16.7)	78 (75,78)	217 (190,252)
July	27 (27,28)	450.2 (391.4,540.5)	17.3 (16,18.7)	83.5 (82,85.5)	164.8 (147.5,193.3)
August	27 (27,27)	282.7 (213.5,376.4)	14.1 (12.7,15.5)	85.5 (84,86.5)	190.8 (186.5,212.8)
September	27 (27,27)	136.2 (103.3,196.1)	9.8 (9.5,11.7)	82 (80,83)	229.8 (225.5,245)
October	28 (27,29)	29.9 (25.5,48.4)	8.1 (7.7,8.8)	67 (67,72)	299.5 (286.5,304)
November	27 (27,28)	2.3 (0,9.9)	9 (8.6,9)	55 (53,58)	257.5 (251.5,258.5)
December	26 (25,26)	0.2 (0,3.4)	9.8 (9,10)	51 (49,55)	295 (293.5,297.5)
4.2. Jamnagar (Gujarat)					
January	22 (21,23)	0 (0,0.1)	15.8 (14.4,17)	44 (41,49)	310 (307,310)
February	24 (23,24)	0 (0,0.6)	16 (14.5,16.8)	45 (44,50)	278.5 (274.5,281)
March	27 (27,28)	0.1 (0,0.2)	16.5 (15.9,17.1)	47 (44,53)	308.5 (306,308.5)
April	29 (29,30)	0 (0,0.4)	20.8 (18.3,21.3)	56 (54,56)	300 (297,300)
May	31 (30,31)	0.1 (0.1,0.2)	24.8 (23.9,25.3)	65 (63,67)	355 (353.5,357.5)
June	31 (31,32)	35.8 (5,93.6)	23.9 (21.7,27.9)	68 (67,69)	311.5 (296.5,321)
July	29 (29,29)	119.4 (88.5,141.7)	25.4 (23.8,27.9)	77.5 (75,78)	167.8 (143.5,197.5)
August	28 (28,28.5)	61.6 (39.7,107.3)	23.2 (22.1,24.6)	79 (77,80)	156.5 (143.5,182)
September	28 (28,29)	70.6 (24.3,113.3)	18.2 (17.8,19.7)	74 (73.5,77)	244.8 (225.3,259)
October	30 (29,31)	0.8 (0.2,12.4)	14 (12.5,14.1)	51 (48,62)	310 (308,310)
November	28 (27,29)	0.1 (0,1.9)	13.9 (13,14.6)	43 (40,50)	230 (228.5,231)
December	24 (24,25)	0 (0,0)	16.2 (14.5,17.1)	43 (39,46)	252.5 (250,252.5)
4.3. Jaipur (Rajasthan)					
January	14 (14,15)	1.9 (0.3,7.9)	8.3 (8.2,8.5)	47 (42,50)	253.5 (245.5,255)
February	18 (18,20)	1 (0.3,3.5)	10.2 (8.9,10.9)	34 (31,44)	277.5 (275.5,288.5)
March	25 (24,25)	0.8 (0,4.1)	10.2 (10,11)	24 (24,29)	304 (301.5,308.5)
April	31 (31,32)	3 (0.8,4.6)	11.2 (10.5,11.7)	19 (16,20)	323 (315.5,327.5)
May	36 (36,36)	5.5 (0.4,6.9)	14.3 (13.1,16.5)	19 (15,21)	383.5 (381.5,385)
June	36 (36,37)	9.9 (3.5,20.5)	13.9 (12.4,17.4)	30 (28,34)	363 (359.5,367.5)
July	32 (32,33.5)	48.4 (33.6,69)	12.8 (10.6,13.9)	55.5 (51,56.5)	351.8 (338.8,369.5)
August	29.5 (29,31)	74.1 (59.6,138.3)	11.1 (10.5,12.7)	66.5 (58.5,73.5)	287.5 (278,302.5)
September	29.5 (28.5,30)	19.4 (14.2,31)	10.4 (9.4,11.5)	53.5 (46.5,62.5)	281.8 (274.8,290.5)
October	28 (27,28)	2.3 (0,4.7)	8.4 (7.4,9.4)	31 (27,41)	263.5 (262.5,265)
November	22 (21,23)	0 (0,0.8)	7.2 (6.7,8)	29 (26,34)	225 (223.5,225)
December	16 (16,17)	0.4 (0,0.7)	7.6 (6.3,8.4)	37 (33,39)	232.5 (229.5,232.5)

Note: IQR- Interquartile range

Supplementary Figure 2: The pattern of monthly meteorological parameters across the regions

SF2a: Monthly median temperature for different regions in India (^{0}C)

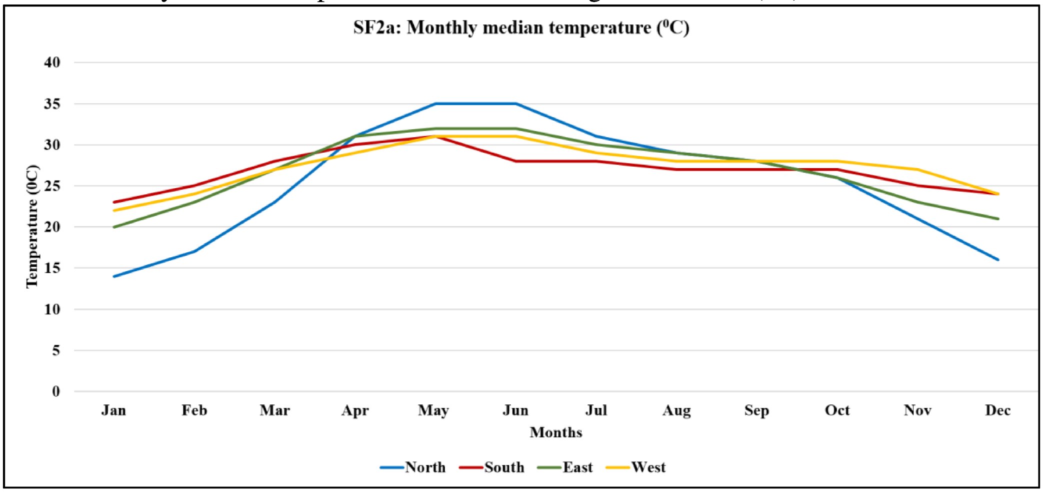

SF2b: Monthly median rainfall (mm) for different regions in India

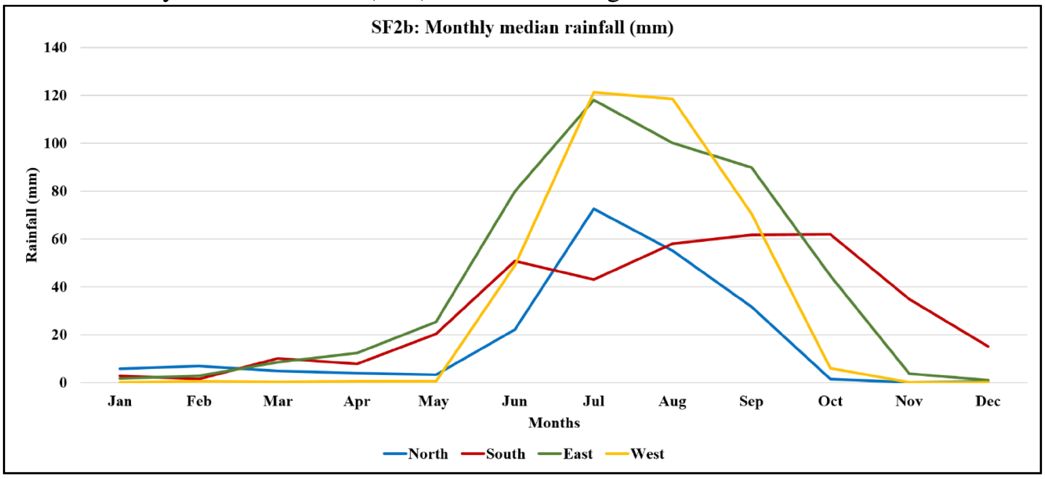

SF2c: Monthly median wind speed (Kmph) for different regions in India

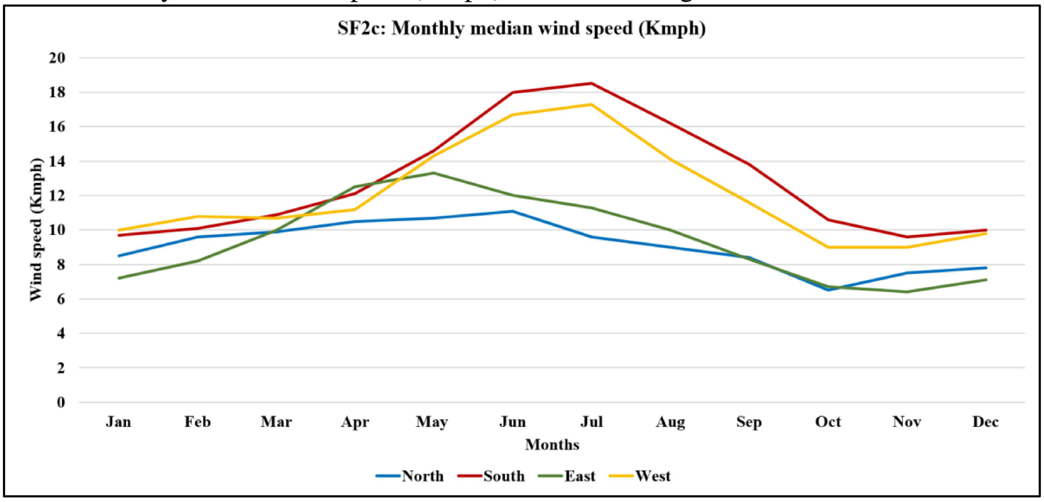

SF2d: Monthly median humidity (%) for different regions in India

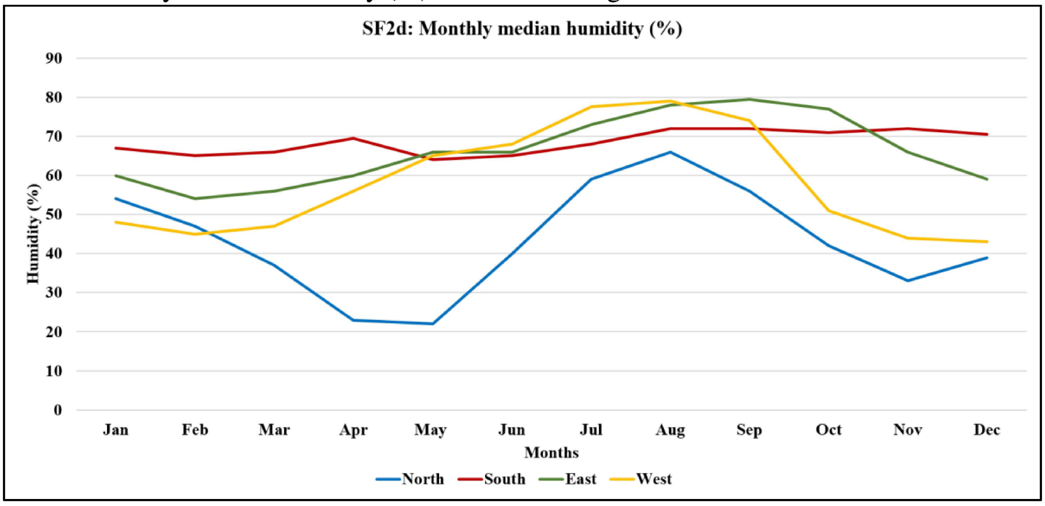

SF2e: Monthly median sunshine (hours) for different regions in India

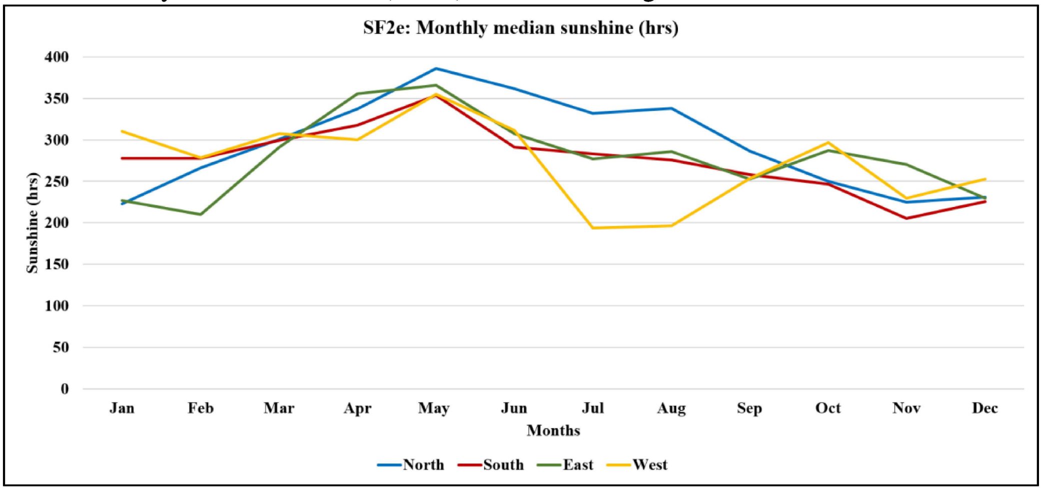

Supplementary Figure 3: The pooled monthly (SF3a) and seasonal (SF3b) distribution of intussusception in children during July 2010 to September 2017

SF3a: The monthly trend of intussusception cases

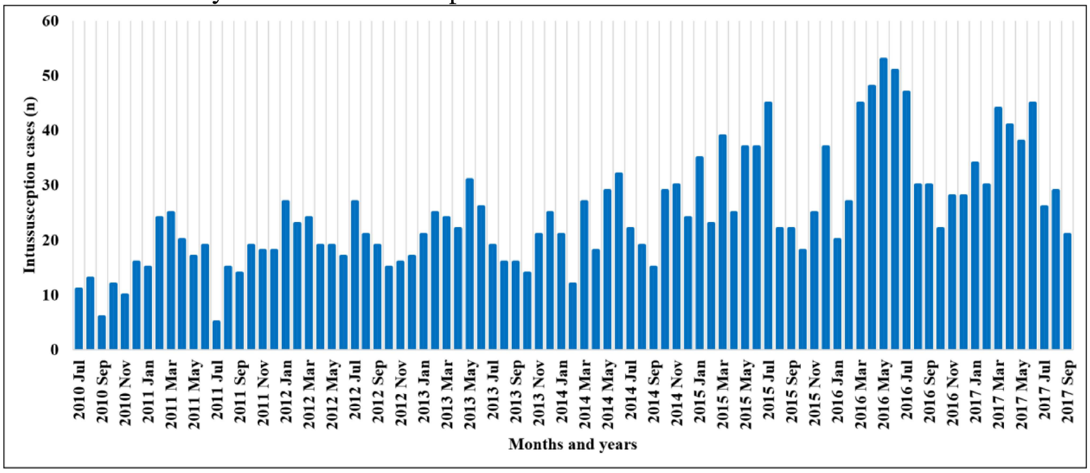

SF3b: The seasonal trend of intussusception cases

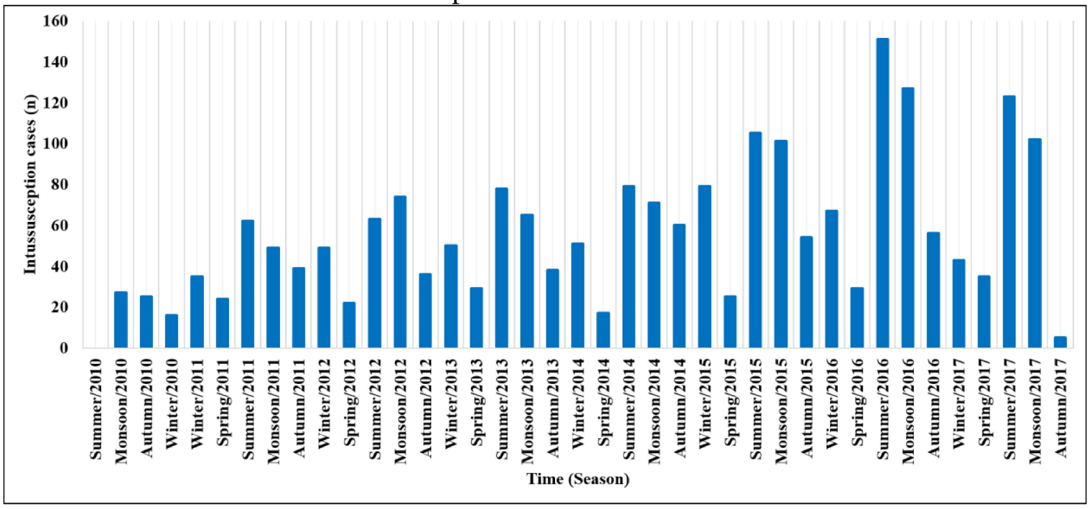

Das MK. *BMJ Open* 2021; 11:e043687. doi: 10.1136/bmjopen-2020-043687

Supplementary Table 5. Pearson correlation analysis between monthly intussusception cases and monthly median meteorological parameters for the study sites

Regions/Study sites (n, number of cases)	Pearson correlation coefficient (r) for meteorological parameters				
	Temperature (°C)	Rainfall (mm)	Wind Speed (kmph)	Humidity (%)	Sunshine (hour)
North region					
Srinagar (n=145)	.253*	.164	.183	.012	.239*
Delhi and Gurgaon (n=96)	.107	-.045	.183*	-.173*	.134
Lucknow (110)	.091	.003	.285**	-.106	.192
Indore (n=19)	.101	-.211*	.290**	-.309**	.188
East region					
Bhubaneswar/Cuttack (n=335)	.047	-.056	.125	-.167*	.035
Berhampur (n=30)	.050	.047	.035	.044	.036
Kolkata (n=80)	.220*	-.052	.291**	-.144	.253*
Patna (n=19)	-.124	-.189	-.038	-.216*	-.040
Guwahati (n=112)	.220*	.001	.317**	-.119	.194
Agartala (n=17)	.082	.080	.139	-.017	.016
South region					
Vishakhapatnam (n=77)	.088	.053	-.002	.022	.029
Hyderabad (n=2)	-.030	-.061	-.131	.022	-.096
Coimbatore (n=136)	-.015	-.075	-.155	-.028	.017
Chennai (n=92)	.052	-.017	.007	.015	-.016
Trivandrum (n=774)	.085	-.286**	-.011	-.323**	.229*
West region					
Mumbai (n=34)	-.066	-.081	-.027	-.103	.035
Jamnagar (n=54)	.110	-.017	.011	.026	.105
Jaipur (n=29)	.187	-.078	.148	.001	.122

Note: Pearson correlation coefficient (r); Significance two tailed
** Significance level- p value <0.05; ** Significance level- p value <0.01*

Supplementary Table 6. Spearman's Rank correlation analysis between monthly intussusception cases and monthly median meteorological parameters for the study sites

Regions/Study sites (n, number of cases)	Pearson correlation coefficient (r) for meteorological parameters				
	Temperature (°C)	Rainfall (mm)	Wind Speed (kmph)	Humidity (%)	Sunshine (hour)
North region					
Srinagar (n=145)	.208	.122	.146	.032	.169
Delhi and Gurgaon (n=96)	.067	-.101	.136	-.184*	.065
Lucknow (110)	.166	.064	.331**	-.101	.225*
Indore (n=19)	.095	-.148	.286**	-.301**	.138
East region					
Bhubaneswar/Cuttack (n=335)	.036	-.068	.117	-.143	-.009
Berhampur (n=30)	.058	.037	.044	.035	.021
Kolkata (n=80)	.097	-.084	.170	-.236*	.125
Patna (n=19)	-.146	-.264*	-.085	-.231*	-.040
Guwahati (n=112)	.240*	-.014	.290**	-.118	.165
Agartala (n=17)	.107	.107	.154	.021	.044
South region					
Vishakhapatnam (n=77)	.106	.081	.048	.017	.060
Hyderabad (n=2)	-.012	-.052	-.138	.040	-.043
Coimbatore (n=136)	.002	-.123	-.147	-.091	.070
Chennai (n=92)	.055	.041	.025	.055	-.027
Trivandrum (n=774)	.134	-.287**	-.026	-.353**	.301**
West region					
Mumbai (n=34)	-.025	-.140	-.092	-.120	.046
Jamnagar (n=54)	.056	.071	.019	-.009	.106
Jaipur (n=29)	.131	-.082	.069	-.075	.079

Note: Pearson correlation coefficient (r); Significance two tailed
** Significance level- p value <0.05; ** Significance level- p value <0.01*

Das MK. BMJ Open 2021; 11:e043687. doi: 10.1136/bmjopen-2020-043687

Supplementary Table 7. Regression analysis of the association between the intussusception cases and meteorological parameters for the study sites.

Study sites/ Variables	Regression coefficient, β (95% CI)	Standard error, S_β	χ2 - value	p value
North region				
Srinagar (n=145)#				
Temperature (^{0}C)	0.043(0.003,0.082)	0.020	4.479	**0.034**
Rainfall (mm)	0.007(-0.004,0.018)	0.005	1.616	0.204
Wind Speed (Kmph)	0.150(-0.048,0.347)	0.100	2.207	0.137
Humidity (%)	0.001(-0.018,0.020)	0.009	0.010	0.921
Sunshine (hour)	0.005(0.000,0.010)	0.002	3.930	**0.047**
Delhi and Gurgaon (n=96)*				
Temperature (^{0}C)	0.025(-0.005,0.055)	0.015	2.712	0.100
Rainfall (mm)	-0.003(-0.012,0.006)	0.004	0.476	0.490
Wind Speed (Kmph)	0.229(0.068,0.390)	0.082	7.805	**0.005**
Humidity (%)	-0.021(-0.036,-0.005)	0.007	7.041	**0.008**
Sunshine (hour)	0.004(0.000,0.008)	0.002	4.205	**0.040**
Lucknow (n=110)*				
Temperature (^{0}C)	0.013(-0.014,0.041)	0.014	0.873	0.350
Rainfall (mm)	0.000(-0.008,0.008)	0.004	0.001	0.972
Wind Speed (Kmph)	0.224(0.074,0.374)	0.077	8.580	**0.003**
Humidity (%)	-0.008(-0.022,0.006)	0.007	1.195	0.274
Sunshine (hour)	0.004(0.000,0.007)	0.002	3.915	**0.048**
Indore (n=19)*				
Temperature (^{0}C)	0.036(-0.033,0.105)	0.035	1.055	0.304
Rainfall (mm)	-0.039(-0.078,0.001)	0.020	3.723	0.054
Wind Speed (Kmph)	0.623(0.193,1.054)	0.219	8.062	**0.005**
Humidity (%)	-0.059(-0.097,-0.021)	0.019	9.110	**0.003**
Sunshine (hour)	0.009(0.000,0.017)	0.004	3.556	0.059
East region				
Bhubaneswar/Cuttack (n=335)#				
Temperature (^{0}C)	0.012(-0.032,0.057)	0.023	.286	0.593
Rainfall (mm)	-0.001(-0.006,0.003)	0.002	.429	0.513
Wind Speed (Kmph)	0.060(-0.021,0.141)	0.041	2.131	0.144
Humidity (%)	-0.022(-0.044,0.000)	0.011	3.953	**0.047**
Sunshine (hour)	0.001(-0.003,0.005)	0.002	.157	0.692
Berhampur (n=30)*				
Temperature (^{0}C)	0.023(-0.068,0.114)	0.046	0.239	0.625
Rainfall (mm)	0.002(-0.006,0.01)	0.004	0.211	0.646
Wind Speed (Kmph)	0.027(-0.128,0.182)	0.079	0.118	0.731
Humidity (%)	0.009(-0.032,0.051)	0.021	0.186	0.666
Sunshine (hour)	0.001(-0.006,0.009)	0.004	0.125	0.724
Kolkata (n=80)*				
Temperature (^{0}C)	0.113(0.050,0.176)	0.032	12.448	**0.000**
Rainfall (mm)	-0.002(-0.007,0.003)	0.002	0.710	0.400
Wind Speed (Kmph)	0.233(0.134,0.332)	0.050	21.269	**0.000**
Humidity (%)	-0.032(-0.059,-0.005)	0.013	5.433	**0.020**
Sunshine (hour)	0.010(0.005,0.014)	0.002	16.577	**0.000**

Patna (n=19)*				
Temperature (^{0}C)	-0.067(-0.177,0.042)	0.056	1.452	0.228
Rainfall (mm)	-0.012(-0.025,0.001)	0.006	3.157	0.076
Wind Speed (Kmph)	-0.037(-0.235,0.161)	0.101	0.137	0.711
Humidity (%)	-0.063(-0.124,-0.003)	0.031	4.208	**0.040**
Sunshine (hour)	-0.002(-0.012,0.008)	0.005	0.150	0.698
Guwahati (n=112)*				
Temperature (^{0}C)	0.050(0.002,0.098)	0.025	4.097	**0.043**
Rainfall (mm)	0.000(-0.004,0.004)	0.002	0.000	0.995
Wind Speed (Kmph)	0.118(0.038,0.199)	0.041	8.408	**0.004**
Humidity (%)	-0.012(-0.034,0.01)	0.011	1.212	0.271
Sunshine (hour)	0.004(0.000,0.008)	0.002	3.207	0.073
Agartala (n=17)*				
Temperature (^{0}C)	0.060(-0.066,0.186)	0.064	0.874	0.350
Rainfall (mm)	0.005(-0.006,0.016)	0.005	0.838	0.360
Wind Speed (Kmph)	0.166(-0.042,0.374)	0.106	2.435	0.119
Humidity (%)	-0.005(-0.061,0.050)	0.028	0.037	0.848
Sunshine (hour)	0.001(-0.009,0.011)	0.005	0.033	0.857
South region				
Vishakhapatnam (n=77)*				
Temperature (^{0}C)	0.043(-0.058,0.144)	0.051	0.702	0.402
Rainfall (mm)	0.003(-0.007,0.012)	0.005	0.255	0.613
Wind Speed (Kmph)	-0.001(-0.071,0.070)	0.036	0.001	0.981
Humidity (%)	0.008(-0.069,0.085)	0.039	0.042	0.837
Sunshine (hour)	0.001(-0.005,0.007)	0.003	0.078	0.780
Hyderabad (n=2)*				
Temperature (^{0}C)	-0.088(-0.717,0.541)	0.321	0.075	0.784
Rainfall (mm)	-0.018(-0.085,0.048)	0.034	0.298	0.585
Wind Speed (Kmph)	-0.650(-2.074,0.774)	0.726	0.801	0.371
Humidity (%)	0.051(-0.436,0.539)	0.249	0.043	0.837
Sunshine (hour)	-0.017(-0.056,0.022)	0.020	0.764	0.382
Coimbatore (n=136)*				
Temperature (^{0}C)	-0.007(-0.082,0.068)	0.038	0.033	0.857
Rainfall (mm)	-0.003(-0.011,0.004)	0.004	0.825	0.364
Wind Speed (Kmph)	-0.053(-0.108,0.002)	0.028	3.506	0.061
Humidity (%)	-0.010(-0.067,0.047)	0.029	0.119	0.730
Sunshine (hour)	0.000(-0.004,0.005)	0.002	0.043	0.836
Chennai (n=92)*				
Temperature (^{0}C)	0.027(-0.065,0.120)	0.047	0.337	0.562
Rainfall (mm)	-0.001(-0.010,0.008)	0.005	0.036	0.850
Wind Speed (Kmph)	0.003(-0.062,0.067)	0.033	0.006	0.938
Humidity (%)	0.006(-0.064,0.076)	0.036	0.028	0.868
Sunshine (hour)	0.000(-0.006,0.005)	0.003	0.030	0.863
Trivandrum (n=774)*				
Temperature (^{0}C)	0.015(-0.016,0.047)	0.016	0.895	0.344
Rainfall (mm)	-0.005(-0.008,-0.002)	0.002	10.159	**0.001**
Wind Speed (Kmph)	-0.001(-0.024,0.021)	0.011	0.016	.898
Humidity (%)	-0.044(-0.068,-0.020)	0.012	12.938	**0.000**

Sunshine (hour)	0.002(0.001,0.004)	0.001	6.538	**0.011**
West region				
Mumbai (n=34)*				
Temperature (^{0}C)	-0.040(-0.165,0.085)	0.064	0.387	0.534
Rainfall (mm)	-0.003(-0.011,0.005)	0.004	0.580	0.446
Wind Speed (Kmph)	-0.016(-0.140,0.108)	0.063	0.065	0.799
Humidity (%)	-0.013(-0.039,0.013)	0.013	0.929	0.335
Sunshine (hour)	0.001(-0.006,0.008)	0.004	0.108	0.742
Jamnagar (n=54)*				
Temperature (^{0}C)	0.066(-0.042,0.173)	0.055	1.440	0.230
Rainfall (mm)	-0.001(-0.006,0.005)	0.003	0.035	0.852
Wind Speed (Kmph)	0.006(-0.090,0.102)	0.049	0.015	0.903
Humidity (%)	0.003(-0.017,0.023)	0.010	0.080	0.777
Sunshine (hour)	0.003(-0.002,0.009)	0.003	1.305	0.253
Jaipur (n=29)*				
Temperature (^{0}C)	0.150(-0.009,0.309)	0.081	3.425	0.064
Rainfall (mm)	-0.003(-0.012,0.005)	0.004	0.600	0.439
Wind Speed (Kmph)	0.092(-0.031,0.216)	0.063	2.141	0.143
Humidity (%)	0.000(-0.027,0.027)	0.014	0.000	0.993
Sunshine (hour)	0.005(-0.003,0.013)	0.004	1.471	0.225

*Note: Tests used for regression analysis: * Poisson Regression; # Negative Binomial Regression*

Das MK. *BMJ Open* 2021; 11:e043687. doi: 10.1136/bmjopen-2020-043687

Supplementary Table 8. Intussusception cases in children at different temperature ranges at pooled and regional level

Pooled/ Region	Temperature range (°C)	Years of data collection								Total
		2010	2011	2012	2013	2014	2015	2016	2017	
Pooled	<20°C	8	14	24	29	26	39	55	51	246
	21°C -25°C	2	31	44	32	43	54	42	31	279
	26°C -30°C	56	149	153	166	164	208	245	164	1305
	>30°C	2	15	23	33	45	63	88	62	331
	Total	68	209	244	260	278	364	430	308	2161
North Region	<20°C	5	8	18	28	20	27	45	46	197
	21°C -25°C	1	5	3	2	10	7	4	1	33
	26°C -30°C	1	2	4	3	4	6	13	3	36
	>30°C	2	4	12	11	14	21	29	11	104
	Total	9	19	37	44	48	61	91	61	370
South Region	<20°C	0	0	0	0	0	0	0	0	0
	21°C -25°C	1	14	15	10	7	11	11	8	77
	26°C -30°C	49	117	113	132	116	157	167	116	967
	>30°C	0	3	2	7	1	6	6	12	37
	Total	50	134	130	149	124	174	184	136	1081
East Region	<20°C	3	6	6	1	5	10	9	4	44
	21°C -25°C	0	10	21	17	22	36	23	21	150
	26°C -30°C	6	24	26	23	37	36	53	35	240
	>30°C	0	5	8	13	20	30	46	37	159
	Total	9	45	61	54	84	112	131	97	593
West Region	<20°C	0	0	0	0	1	2	1	1	5
	21°C -25°C	0	2	5	3	4	0	4	1	19
	26°C -30°C	0	6	10	8	7	9	12	10	62
	>30°C	0	3	1	2	10	6	7	2	31
	Total	0	11	16	13	22	17	24	14	117

Das MK. *BMJ Open* 2021; 11:e043687. doi: 10.1136/bmjopen-2020-043687

Supplementary Table 9. Variations in the intussusception cases between the different temperature categories by Kruskal-Wallis H test

Level	df	χ^2-statistic	P- value
Pooled	3	15.933	< 0.001
North Region	3	21.054	< 0.001
South Region	3	50.625	< 0.001
East Region	3	3.502	0.3205
West Region	3	5.606	0.1324

Notes: Pooled- all sites/regions combined; df: degrees of freedom;
χ^2-statistic: critical chi square value

Supplementary Figure 4: Association of monthly intussusception cases with mean monthly temperatures

Pooled for all regions

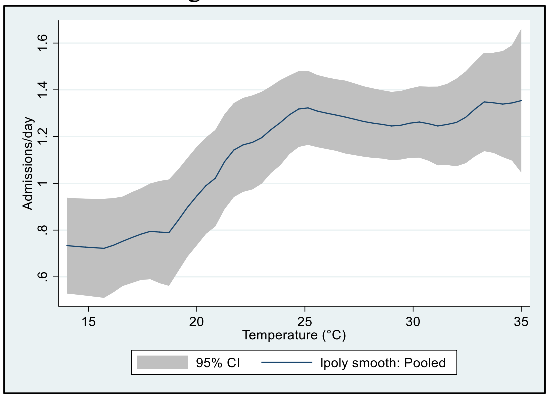

North region

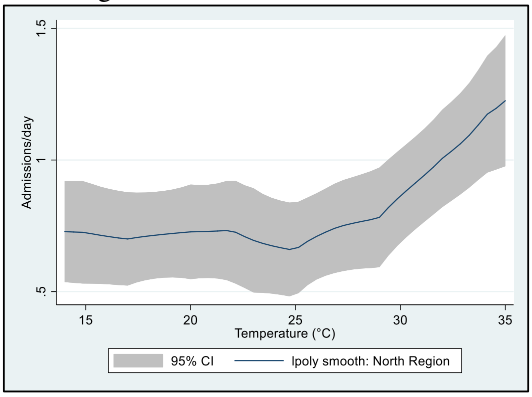

South region

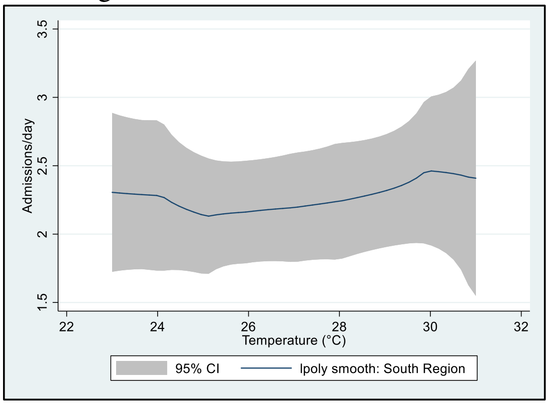

East region

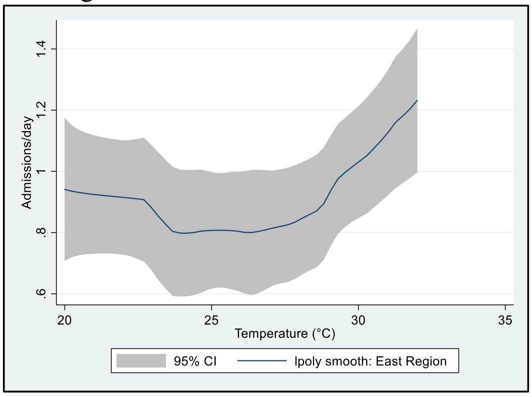

West region

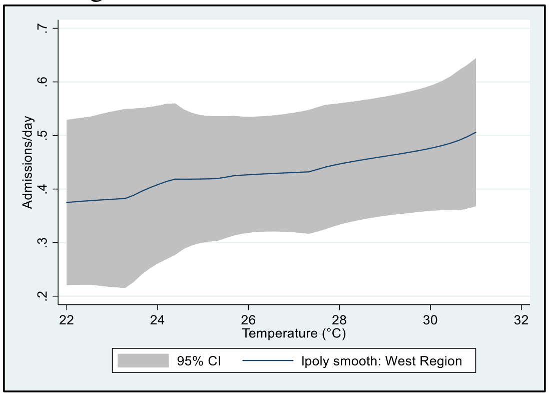

Supplementary Figure 5: Association of monthly intussusception cases with mean monthly humidity

Pooled for all regions

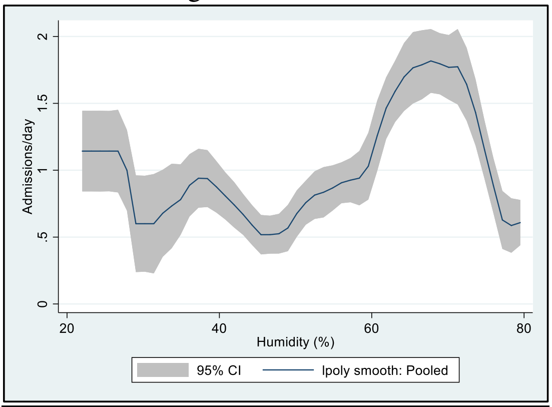

North region

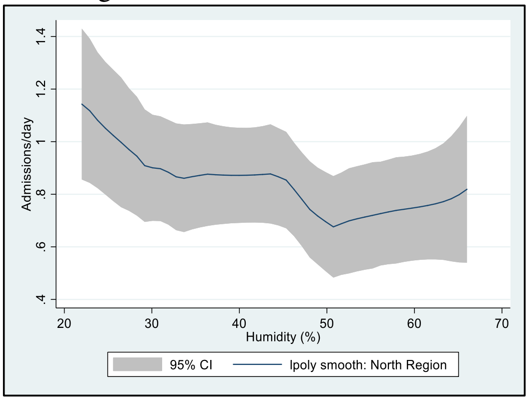

South region

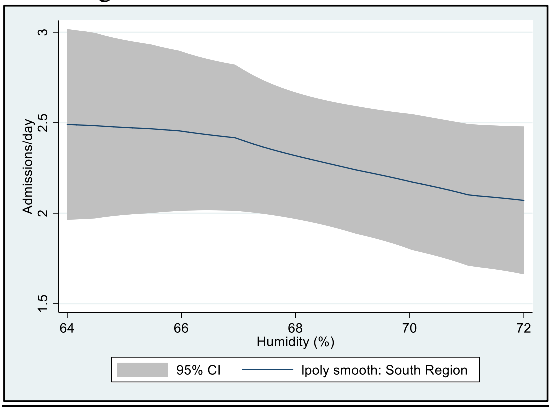

East region

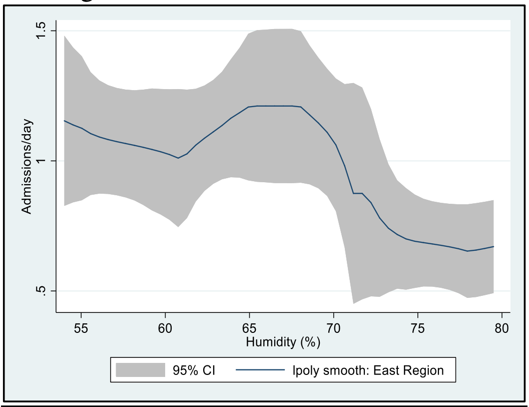

West region

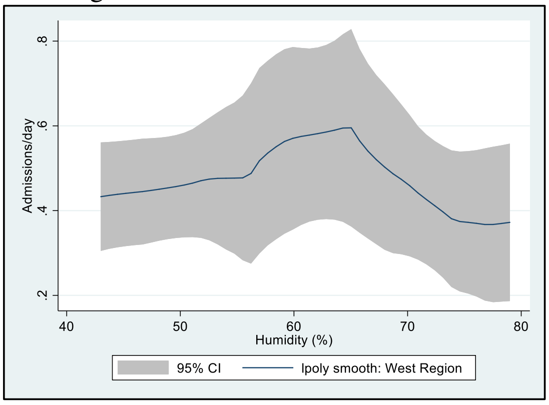

Supplementary Figure 6: Association of monthly intussusception cases with mean monthly wind speed

Pooled for all regions

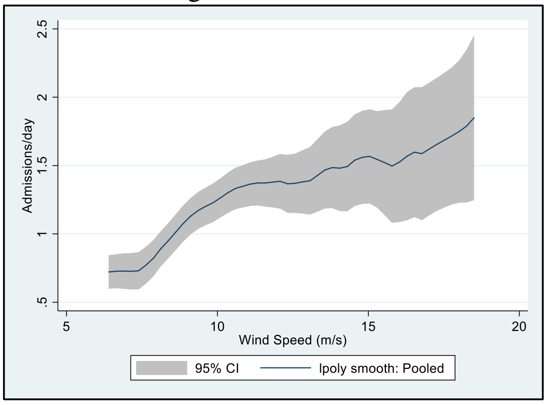

North region

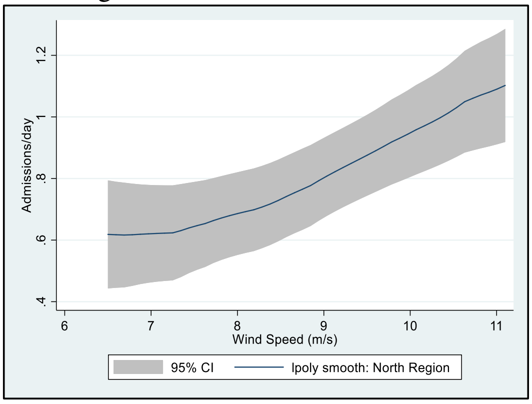

South region

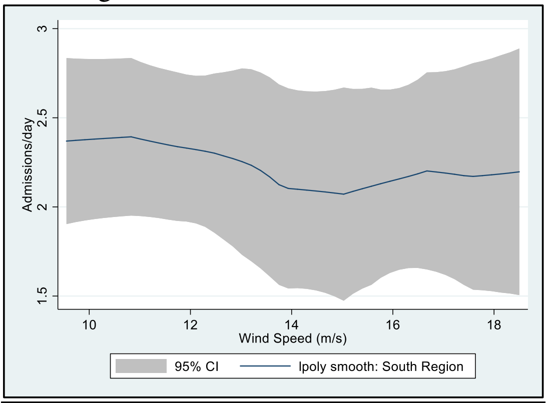

East region

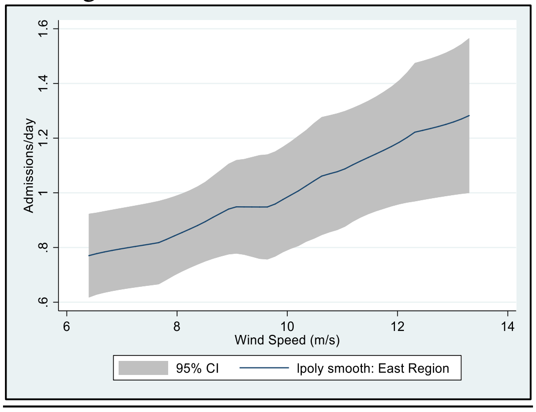

West region

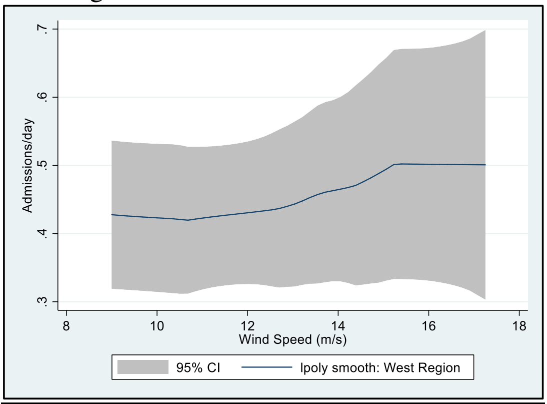

Supplementary Figure 7: Association of monthly intussusception cases with mean monthly sunshine

Pooled for all regions

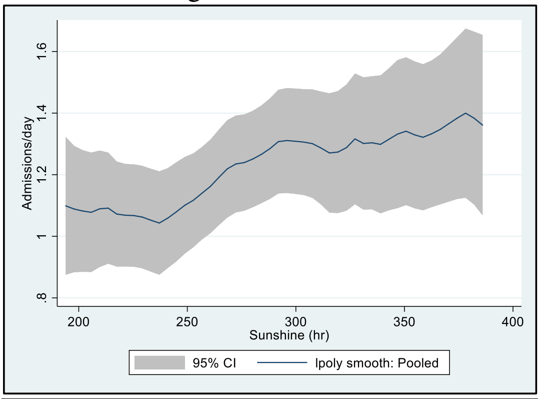

North region

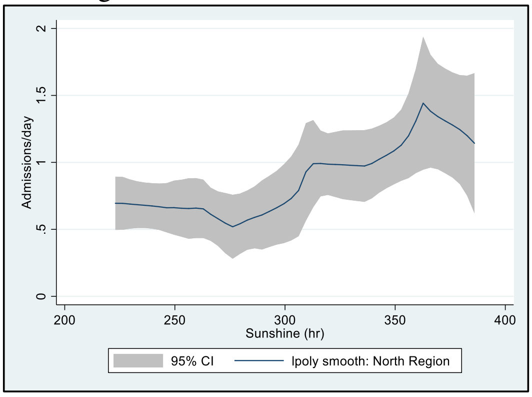

South region

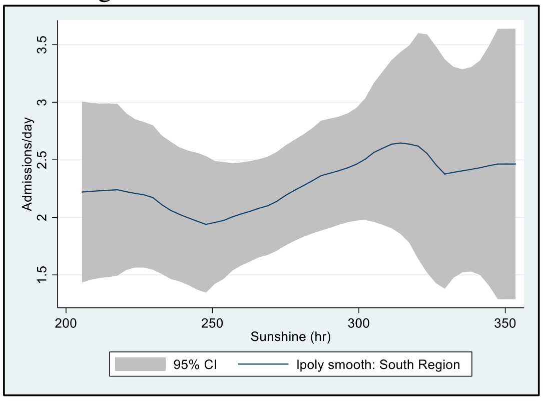

East region

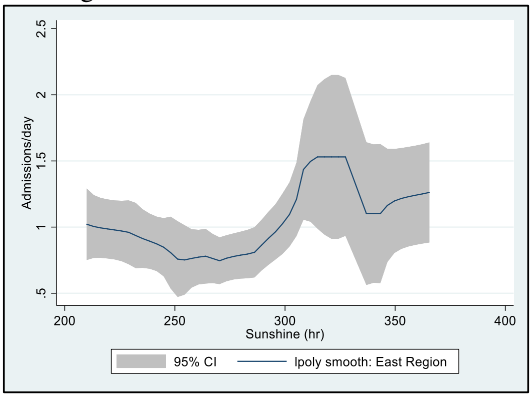

West region

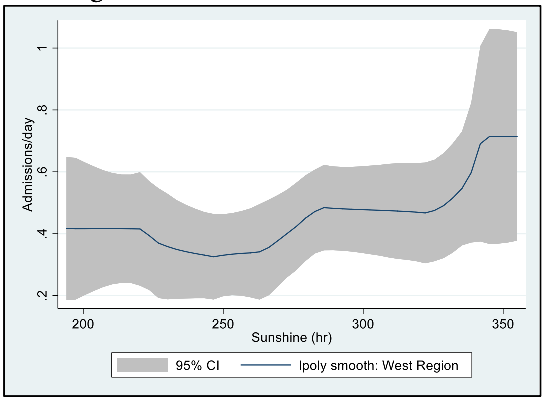

Das MK. *BMJ Open* 2021; 11:e043687. doi: 10.1136/bmjopen-2020-043687

Supplementary Figure 8: Association of monthly intussusception cases with mean monthly rainfall

Pooled for all regions

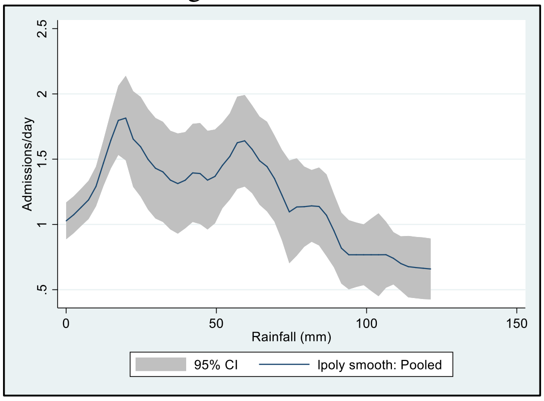

North region

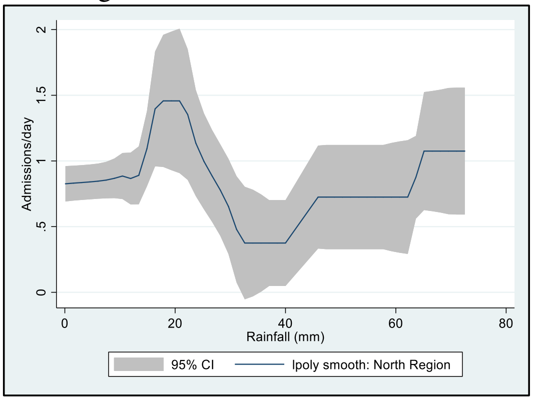

South region

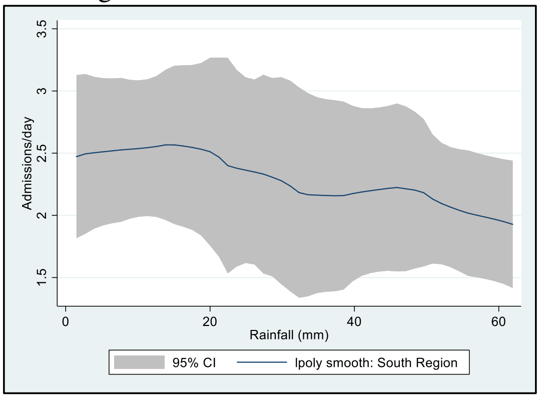

East region

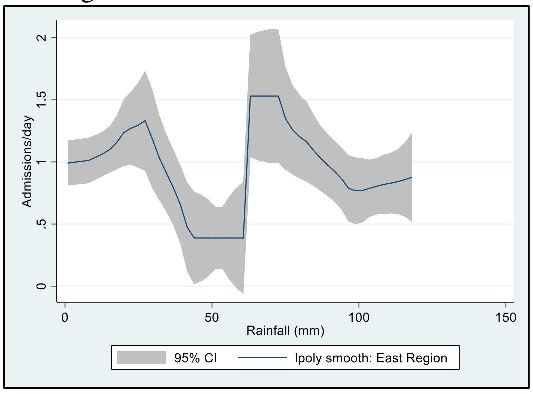

West region

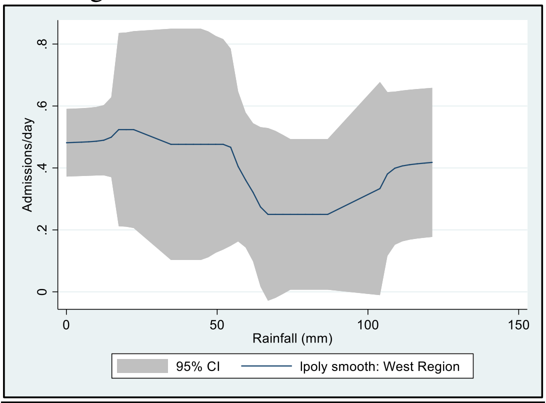

Supplementary Table 10. Factorial analysis-of-variance of intussusception in children with season and age groups

Source	Dependent variables	Type III Sum of squares	Degree of freedom	Mean square	F	p value
Corrected model	Seasonal IS cases	285.847[a]	38	7.522	1.442	**.040**
	Seasonal IS cases of 2-6 months	49.145[b]	38	1.293	1.579	**.014**
	Seasonal IS cases of 7-12 months	45.682[c]	38	1.202	1.012	.449
	Seasonal IS cases of 13-24 months	31.855[d]	38	.838	1.334	.085
Intercept	Seasonal IS cases	789.283	1	789.283	151.302	.000
	Seasonal IS cases of 2-6 months	101.336	1	101.336	123.760	.000
	Seasonal IS cases of 7-12 months	135.705	1	135.705	114.285	.000
	Seasonal IS cases of 13-24 months	40.684	1	40.684	64.740	.000
Season	Seasonal IS cases	8.674	4	2.169	.416	.797
	Seasonal IS cases of 2-6 months	1.930	4	.482	.589	.670
	Seasonal IS cases of 7-12 months	3.207	4	.802	.675	.609
	Seasonal IS cases of 13-24 months	.856	4	.214	.340	.851
Season year	Seasonal IS cases	82.483	7	11.783	2.259	**.027**
	Seasonal IS cases of 2-6 months	17.669	7	2.524	3.083	**.003**
	Seasonal IS cases of 7-12 months	3.504	7	.501	.422	.889
	Seasonal IS cases of 13-24 months	11.481	7	1.640	2.610	**.011**
Season* Season year	Seasonal IS cases	57.160	27	2.117	.406	.997
	Seasonal IS cases of 2-6 months	13.795	27	.511	.624	.934
	Seasonal IS cases of 7-12 months	19.521	27	.723	.609	.943
	Seasonal IS cases of 13-24 months	7.717	27	.286	.455	.993
Error	Seasonal IS cases	9092.547	1743	5.217		
	Seasonal IS cases of 2-6 months	1427.189	1743	.819		
	Seasonal IS cases of 7-12 months	2069.674	1743	1.187		
	Seasonal IS cases of 13-24 months	1095.327	1743	.628		
Total	Seasonal IS cases	11999.000	1782			
	Seasonal IS cases of 2-6 months	1816.000	1782			
	Seasonal IS cases of 7-12 months	2545.000	1782			
	Seasonal IS cases of 13-24 months	1272.000	1782			
Modified total	Seasonal IS cases	9378.393	1781			
	Seasonal IS cases of 2-6 months	1476.334	1781			
	Seasonal IS cases of 7-12 months	2115.356	1781			
	Seasonal IS cases of 13-24 months	1127.183	1781			

Note: a: R squared= .030 (adjusted R squared= .009); b: R squared= .033 (adjusted R squared= .012); c: R squared = .022 (adjusted R squared= .000); d: R squared= .028 (adjusted R squared= .007)

Das MK. *BMJ Open* 2021; 11:e043687. doi: 10.1136/bmjopen-2020-043687

Supplementary Table 11. Factorial analysis-of-variance of intussusception in children with season and regions

Source	Dependent variables	Type III Sum of squares	Degree of freedom	Mean square	F	p value
Corrected model	Seasonal IS cases	285.847[a]	38	7.522	1.442	**.040**
	Seasonal IS cases of North region	36.237[b]	38	.954	1.809	**.002**
	Seasonal IS cases of South region	55.831[c]	38	1.469	.321	1.000
	Seasonal IS cases of East region	53.071[d]	38	1.397	1.333	.085
	Seasonal IS cases of West region	2.721[e]	38	.072	.662	.944
Intercept	Seasonal IS cases	789.283	1	789.283	151.302	**.000**
	Seasonal IS cases of North region	29.881	1	29.881	56.676	**.000**
	Seasonal IS cases of South region	180.247	1	180.247	39.373	**.000**
	Seasonal IS cases of East region	59.322	1	59.322	56.607	**.000**
	Seasonal IS cases of West region	2.250	1	2.250	20.798	**.000**
Season	Seasonal IS cases	8.674	4	2.169	.416	.797
	Seasonal IS cases of North region	10.274	4	2.569	4.872	**.001**
	Seasonal IS cases of South region	9.239	4	2.310	.505	.732
	Seasonal IS cases of East region	1.801	4	.450	.430	.787
	Seasonal IS cases of West region	.207	4	.052	.479	.752
Season year	Seasonal IS cases	82.483	7	11.783	2.259	**.027**
	Seasonal IS cases of North region	11.119	7	1.588	3.013	**.004**
	Seasonal IS cases of South region	5.618	7	.803	.175	.990
	Seasonal IS cases of East region	18.661	7	2.666	2.544	**.013**
	Seasonal IS cases of West region	.464	7	.066	.612	.746
Season* Season year	Seasonal IS cases	57.160	27	2.117	.406	.997
	Seasonal IS cases of North region	8.198	27	.304	.576	.960
	Seasonal IS cases of South region	28.377	27	1.051	.230	1.000
	Seasonal IS cases of East region	15.527	27	.575	.549	.971
	Seasonal IS cases of West region	1.325	27	.049	.454	.993
Error	Seasonal IS cases	9092.547	1743	5.217		
	Seasonal IS cases of North region	918.939	1743	.527		
	Seasonal IS cases of South region	7979.411	1743	4.578		
	Seasonal IS cases of East region	1826.595	1743	1.048		
	Seasonal IS cases of West region	188.597	1743	.108		
Total	Seasonal IS cases	11999.000	1782			
	Seasonal IS cases of North region	1032.000	1782			
	Seasonal IS cases of South region	8691.000	1782			
	Seasonal IS cases of East region	2077.000	1782			
	Seasonal IS cases of West region	199.000	1782			
Modified total	Seasonal IS cases	9378.393	1781			
	Seasonal IS cases of North region	955.176	1781			
	Seasonal IS cases of South region	8035.242	1781			
	Seasonal IS cases of East region	1879.666	1781			
	Seasonal IS cases of West region	191.318	1781			

Note: a: R squared = .030 (adjusted R squared= .009); b: R squared = .038 (adjusted R squared= .017); c: R squared = .007 (adjusted R squared= -.015); d: R squared= .028 (adjusted R squared= .007); e: R squared = .014 (adjusted R squared= -.007)

Supplementary Table 12. Multiple comparisons of seasonal intussusception in children in different seasons pooled for all sites

Dependent variable		Season (A)	Season (B)	Mean difference (A-B)	Standard error	p value	95% CI
Seasonal IS cases pooled	Tukey HSD	Monsoon	Spring	-.175	.2285	.940	-0.8 , 0.45
			Summer	-.409	.1643	.093	-0.86 , 0.04
			Monsoon	-.038	.1573	.999	-0.47 , 0.39
			Autumn	.160	.1758	.892	-0.32 , 0.64
		Spring	Winter	.175	.2285	.940	-0.45 , 0.8
			Summer	-.234	.2221	.831	-0.84 , 0.37
			Monsoon	.137	.2169	.970	-0.46 , 0.73
			Autumn	.336	.2307	.592	-0.29 , 0.97
		Summer	Winter	.409	.1643	.093	-0.04 , 0.86
			Spring	.234	.2221	.831	-0.37 , 0.84
			Monsoon	.371	.1478	.089	-0.03 , 0.77
			Autumn	.569*	.1673	**.006**	0.11 , 1.03
		Monsoon	Winter	.038	.1573	.999	-0.39 , 0.47
			Spring	-.137	.2169	.970	-0.73 , 0.46
			Summer	-.371	.1478	.089	-0.77 , 0.03
			Autumn	.199	.1604	.729	-0.24 , 0.64
		Autumn	Winter	-.160	.1758	.892	-0.64 , 0.32
			Spring	-.336	.2307	.592	-0.97 , 0.29
			Summer	-.569*	.1673	**.006**	-1.03 , -0.11
			Monsoon	-.199	.1604	.729	-0.64 , 0.24

*Note: * The difference is significant at p<0.05 level.*

Supplementary Table 13. Multiple comparisons of seasonal intussusception in children in different seasons at regional level

Dependent variable		Season (A)	Season (B)	Mean difference (A-B)	Standard error	p value	95% CI
North Region	Tukey HSD	Winter	Spring	-.151	.0726	.231	-0.35 , 0.05
			Summer	-.039	.0522	.943	-0.18 , 0.1
			Monsoon	.125	.0500	.089	-0.01 , 0.26
			Autumn	.139	.0559	.093	-0.01 , 0.29
		Spring	Winter	.151	.0726	.231	-0.05 , 0.35
			Summer	.111	.0706	.512	-0.08 , 0.3
			Monsoon	.276*	.0690	**.001**	0.09 , 0.46
			Autumn	.290*	.0733	**.001**	0.09 , 0.49
		Summer	Winter	.039	.0522	.943	-0.1 , 0.18
			Spring	-.111	.0706	.512	-0.3 , 0.08
			Monsoon	.165*	.0470	**.004**	0.04 , 0.29
			Autumn	.179*	.0532	**.007**	0.03 , 0.32
		Monsoon	Winter	-.125	.0500	.089	-0.26 , 0.01
			Spring	-.276*	.0690	**.001**	-0.46 , -0.09
			Summer	-.165*	.0470	**.004**	-0.29 , -0.04
			Autumn	.014	.0510	.999	-0.13 , 0.15
		Autumn	Winter	-.139	.0559	.093	-0.29 , 0.01
			Spring	-.290*	.0733	**.001**	-0.49 , -0.09
			Summer	-.179*	.0532	**.007**	-0.32 , -0.03
			Monsoon	-.014	.0510	.999	-0.15 , 0.13
South Region	Tukey HSD	Winter	Spring	-.014	.2140	1.000	-0.6 , 0.57
			Summer	-.197	.1539	.705	-0.62 , 0.22
			Monsoon	-.141	.1473	.875	-0.54 , 0.26
			Autumn	.032	.1647	1.000	-0.42 , 0.48
		Spring	Winter	.014	.2140	1.000	-0.57 , 0.6
			Summer	-.183	.2080	.905	-0.75 , 0.39
			Monsoon	-.127	.2032	.971	-0.68 , 0.43
			Autumn	.046	.2161	1.000	-0.54 , 0.64
		Summer	Winter	.197	.1539	.705	-0.22 , 0.62
			Spring	.183	.2080	.905	-0.39 , 0.75
			Monsoon	.056	.1384	.994	-0.32 , 0.43
			Autumn	.229	.1568	.588	-0.2 , 0.66
		Monsoon	Winter	.141	.1473	.875	-0.26 , 0.54
			Spring	.127	.2032	.971	-0.43 , 0.68
			Summer	-.056	.1384	.994	-0.43 , 0.32
			Autumn	.173	.1503	.779	-0.24 , 0.58
		Autumn	Winter	-.032	.1647	1.000	-0.48 , 0.42
			Spring	-.046	.2161	1.000	-0.64 , 0.54
			Summer	-.229	.1568	.588	-0.66 , 0.2
			Monsoon	-.173	.1503	.779	-0.58 , 0.24
East region	Tukey HSD	Winter	Spring	.035	.1024	.997	-0.24 , 0.31
			Summer	-.125	.0736	.433	-0.33 , 0.08
			Monsoon	-.001	.0705	1.000	-0.19 , 0.19

			Autumn	.010	.0788	1.000	-0.21 , 0.23
		Spring	Winter	-.035	.1024	.997	-0.31 , 0.24
			Summer	-.160	.0995	.490	-0.43 , 0.11
			Monsoon	-.036	.0972	.996	-0.3 , 0.23
			Autumn	-.025	.1034	.999	-0.31 , 0.26
		Summer	Winter	.125	.0736	.433	-0.08 , 0.33
			Spring	.160	.0995	.490	-0.11 , 0.43
			Monsoon	.124	.0662	.331	-0.06 , 0.31
			Autumn	.135	.0750	.372	-0.07 , 0.34
		Monsoon	Winter	.001	.0705	1.000	-0.19 , 0.19
			Spring	.036	.0972	.996	-0.23 , 0.3
			Summer	-.124	.0662	.331	-0.31 , 0.06
			Autumn	.011	.0719	1.000	-0.19 , 0.21
		Autumn	Winter	-.010	.0788	1.000	-0.23 , 0.21
			Spring	.025	.1034	.999	-0.26 , 0.31
			Summer	-.135	.0750	.372	-0.34 , 0.07
			Monsoon	-.011	.0719	1.000	-0.21 , 0.19
West region	Tukey HSD	Winter	Spring	-.046	.0329	.637	-0.14 , 0.04
			Summer	-.048	.0237	.260	-0.11 , 0.02
			Monsoon	-.022	.0227	.872	-0.08 , 0.04
			Autumn	-.021	.0253	.921	-0.09 , 0.05
		Spring	Winter	.046	.0329	.637	-0.04 , 0.14
			Summer	-.002	.0320	1.000	-0.09 , 0.09
			Monsoon	.024	.0312	.941	-0.06 , 0.11
			Autumn	.025	.0332	.947	-0.07 , 0.12
		Summer	Winter	.048	.0237	.260	-0.02 , 0.11
			Spring	.002	.0320	1.000	-0.09 , 0.09
			Monsoon	.026	.0213	.743	-0.03 , 0.08
			Autumn	.027	.0241	.805	-0.04 , 0.09
		Monsoon	Winter	.022	.0227	.872	-0.04 , 0.08
			Spring	-.024	.0312	.941	-0.11 , 0.06
			Summer	-.026	.0213	.743	-0.08 , 0.03
			Autumn	.001	.0231	1.000	-0.06 , 0.06
		Autumn	Winter	.021	.0253	.921	-0.05 , 0.09
			Spring	-.025	.0332	.947	-0.12 , 0.07
			Summer	-.027	.0241	.805	-0.09 , 0.04
			Monsoon	-.001	.0231	1.000	-0.06 , 0.06

*Note: * The difference is significant at p<0.05 level.*

Das MK. *BMJ Open* 2021; 11:e043687. doi: 10.1136/bmjopen-2020-043687

Supplementary Table 14. Multiple comparisons of seasonal intussusception in children in different seasons for different age groups

Dependent variable		Season (A)	Season (B)	Mean difference (A-B)	Standard error	p value	95% CI
Seasonal IS cases in children aged 2-6 months	Tukey HSD	Winter	Spring	-.003	.0905	1.000	-0.25 , 0.24
			Summer	-.073	.0651	.798	-0.25 , 0.11
			Monsoon	.019	.0623	.998	-0.15 , 0.19
			Autumn	.123	.0696	.395	-0.07 , 0.31
		Spring	Winter	.003	.0905	1.000	-0.24 , 0.25
			Summer	-.070	.0880	.933	-0.31 , 0.17
			Monsoon	.022	.0859	.999	-0.21 , 0.26
			Autumn	.126	.0914	.643	-0.12 , 0.38
		Summer	Winter	.073	.0651	.798	-0.11 , 0.25
			Spring	.070	.0880	.933	-0.17 , 0.31
			Monsoon	.092	.0585	.517	-0.07 , 0.25
			Autumn	.195*	.0663	**.027**	0.01 , 0.38
		Monsoon	Winter	-.019	.0623	.998	-0.19 , 0.15
			Spring	-.022	.0859	.999	-0.26 , 0.21
			Summer	-.092	.0585	.517	-0.25 , 0.07
			Autumn	.104	.0636	.478	-0.07 , 0.28
		Autumn	Winter	-.123	.0696	.395	-0.31 , 0.07
			Spring	-.126	.0914	.643	-0.38 , 0.12
			Summer	-.195*	.0663	**.027**	-0.38 , -0.01
			Monsoon	-.104	.0636	.478	-0.28 , 0.07
Seasonal IS cases in children aged 7-12 months	Tukey HSD	Winter	Spring	-.185	.1090	.439	-0.48 , 0.11
			Summer	-.210	.0784	.057	-0.42 , 0
			Monsoon	-.046	.0750	.973	-0.25 , 0.16
			Autumn	.021	.0839	.999	-0.21 , 0.25
		Spring	Winter	.185	.1090	.439	-0.11 , 0.48
			Summer	-.026	.1059	.999	-0.32 , 0.26
			Monsoon	.138	.1035	.667	-0.14 , 0.42
			Autumn	.206	.1101	.336	-0.1 , 0.51
		Summer	Winter	.210	.0784	.057	0 , 0.42
			Spring	.026	.1059	.999	-0.26 , 0.32
			Monsoon	.164	.0705	.136	-0.03 , 0.36
			Autumn	.231*	.0798	**.031**	0.01 , 0.45
		Monsoon	Winter	.046	.0750	.973	-0.16 , 0.25
			Spring	-.138	.1035	.667	-0.42 , 0.14
			Summer	-.164	.0705	.136	-0.36 , 0.03
			Autumn	.067	.0765	.906	-0.14 , 0.28
		Autumn	Winter	-.021	.0839	.999	-0.25 , 0.21
			Spring	-.206	.1101	.336	-0.51 , 0.1
			Summer	-.231*	.0798	**.031**	-0.45 , -0.01
			Monsoon	-.067	.0765	.906	-0.28 , 0.14
Seasonal IS cases in	Tukey HSD	Winter	Spring	.012	.0793	1.000	-0.2 , 0.23
			Summer	-.126	.0570	.176	-0.28 , 0.03
			Monsoon	-.011	.0546	1.000	-0.16 , 0.14

Das MK. *BMJ Open* 2021; 11:e043687. doi: 10.1136/bmjopen-2020-043687

children aged 7-12 months			Autumn	.016	.0610	.999	-0.15 , 0.18
		Spring	Winter	-.012	.0793	1.000	-0.23 , 0.2
			Summer	-.138	.0771	.378	-0.35 , 0.07
			Monsoon	-.024	.0753	.998	-0.23 , 0.18
			Autumn	.004	.0801	1.000	-0.21 , 0.22
		Summer	Winter	.126	.0570	.176	-0.03 , 0.28
			Spring	.138	.0771	.378	-0.07 , 0.35
			Monsoon	.115	.0513	.167	-0.03 , 0.25
			Autumn	.143	.0581	.102	-0.02 , 0.3
		Monsoon	Winter	.011	.0546	1.000	-0.14 , 0.16
			Spring	.024	.0753	.998	-0.18 , 0.23
			Summer	-.115	.0513	.167	-0.25 , 0.03
			Autumn	.028	.0557	.987	-0.12 , 0.18
		Autumn	Winter	-.016	.0610	.999	-0.18 , 0.15
			Spring	-.004	.0801	1.000	-0.22 , 0.21
			Summer	-.143	.0581	.102	-0.3 , 0.02
			Monsoon	-.028	.0557	.987	-0.18 , 0.12

*Note: * The difference is significant at p<0.05 level.*
