# Supplementary material for: Association of meteorological parameters with intussusception in children aged under 2 years: results from a multisite bidirectional surveillance over 7 years in India
Source: BMJ Open. 2021 May 25;11(5):e043687. doi: 10.1136/bmjopen-2020-043687 (PMC8154980; doi:10.1136/bmjopen-2020-043687)
Supplement: Supplementary data [file bmjopen-2020-043687supp002.pdf]

Revised Title: Association of meteorological parameters with intussusception in children aged under-two years: Results from a multisite bidirectional surveillance over seven years in India

Manuscript ID: bmjopen-2020-043687.R2

Corresponding author: Manoja Kumar Das, MD

The Institute Ethics Committees (IECs) of all the participating institutes reviewed and approved the study protocol titled "Intussusception in Infants: Multisite Retrospective Surveillance in India". The list of IECs reviewed and approved the protocol with the reference numbers for the respective IECs are listed below.

1. Name of IRB: The INCLEN Independent Ethic Committee
   IRB Ref no: IIEC-023
   Name of the Institute: The INCLEN Trust International, New Delhi, India
   Principal Investigator's Institute/Coordinating Institute

2. Name of IRB: Institutional Ethics Committee
   IRB Ref no: IEC/SKIMS Protocol#42/2015
   Name of the Institute: Sher-I-Kashmir Institute of Medical Sciences, Srinagar, Jammu & Kashmir, India

3. Name of IRB: Institutional Ethics Committee
   IRB Ref no: IEC/MAMC/50/4/2015/308
   Name of the Institute: Maulana Azad Medical College, Delhi, India

4. Name of IRB: Medanta Institutional Ethics Committee
   IRB Ref no: MICR 559/2015
   Name of the Institute: Medanta- The Medicity, Gurgaon, Haryana, India

5. Name of IRB: The INCLEN Independent Ethic Committee
   IRB Ref no: 7951/Ethics/R.Cell-15
   Name of the Institute: King George's Medical University, Lucknow, Uttar Pradesh, India

6. Name of IRB: Institutional Ethics Committee
   IRB Ref no: EC/Oct/15/20
   Name of the Institute: Choithram Hospital and Research Centre, Indore, Madhya Pradesh, India

7. Name of IRB: Institutional Ethics Committee
   IRB Ref no: 26/12/2015
   Name of the Institute: King George Hospital, Andhra Medical College, Vishakhapatnam, Andhra Pradesh, India

8. Name of IRB: Ethics Committee
   IRB Ref no: 13/12/2015
   Name of the Institute: Apollo Hospital, Hyderabad, Telengana, India

9. Name of IRB: Institutional Human Ethics Committee

IRB Ref no: Project No 15/294
Name of the Institute: PSG Institute of Medical Sciences, Coimbatore, Tamil Nadu, India

10. Name of IRB: Institutional Ethics Committee- Clinical Studies
    IRB Ref no: 14/12/210
    Name of the Institute: Apollo Hospitals, Chennai, Tamil Nadu

11. Name of IRB: Institutional Ethics Committee
    IRB Ref no: IEC-06/05/2015/MCT
    Name of the Institute: Government Medical College & SAT Hospital, Thiruvananthapuram, Kerala, India

12. Name of IRB: Institutional Ethics Committee
    IRB Ref no: 1256/Acad
    Name of the Institute: Indira Gandhi Institute of Medical Sciences, Patna, Bihar, India

13. Name of IRB: Institutional Ethics Committee
    IRB Ref no: 210/5/10/2015
    Name of the Institute: IMS & SUM Medical College & Hospital, Bhubaneswar, Odisha, India

14. Name of IRB: Institutional Ethics Committee
    IRB Ref no: 311/27-04-16
    Name of the Institute: SCB Medical College, Cuttack, Odisha, India

15. Name of IRB: Institutional Ethics Committee
    IRB Ref no: 210/5/10/2015
    Name of the Institute: MKCG Medical College, Berhampur, Odisha, India

16. Name of IRB: Institutional Ethics Committee
    IRB Ref no: IEC/2016/197
    Name of the Institute: Institute of Post Graduate Medical Education and Research, Kolkata, West Bengal, India

17. Name of IRB: Institutional Ethics Committee
    IRB Ref no: MC/02/2015/274
    Name of the Institute: Gauhati Medical College, Guwahati, Assam, India

18. Name of IRB: Institutional Ethics Committee
    IRB Ref no: 2015/3894
    Name of the Institute: Agartala Government Medical College, Agartala, Tripura, India

19. Name of IRB: Institutional Ethics Committee
    IRB Ref no: FEHJ/IEC/15/0023
    Name of the Institute: Fortis Escorts Hospital, Jaipur, Rajasthan, India

20. Name of IRB: Institutional Ethics Committee
    IRB Ref no: IEC/46/2016
    Name of the Institute: MP Shah Government Medical College, Jamnagar, Gujarat, India

21. Name of IRB: Institutional Ethics Committee

Das MK. *BMJ Open* 2021; 11:e043687. doi: 10.1136/bmjopen-2020-043687

IRB Ref no: IEC/Pharm/288/15
Name of the Institute: Grant Medical College & JJ Hospital, Mumbai, Maharashtra, India

Das MK. *BMJ Open* 2021; 11:e043687. doi: 10.1136/bmjopen-2020-043687
