# Supplementary material for: Association of meteorological parameters with intussusception in children aged under 2 years: results from a multisite bidirectional surveillance over 7 years in India
Source: BMJ Open. 2021 May 25;11(5):e043687. doi: 10.1136/bmjopen-2020-043687 (PMC8154980; doi:10.1136/bmjopen-2020-043687)
Supplement: Author's manuscript [file bmjopen-2020-043687.draft_revisions.pdf]

BMJ Open is committed to open peer review. As part of this commitment we make the peer review history of every article we publish publicly available.

When an article is published we post the peer reviewers' comments and the authors' responses online. We also post the versions of the paper that were used during peer review. These are the versions that the peer review comments apply to.

The versions of the paper that follow are the versions that were submitted during the peer review process. They are not the versions of record or the final published versions. They should not be cited or distributed as the published version of this manuscript.

BMJ Open is an open access journal and the full, final, typeset and author-corrected version of record of the manuscript is available on our site with no access controls, subscription charges or pay-per-view fees (http://bmjopen.bmj.com).

If you have any questions on BMJ Open's open peer review process please email info.bmjopen@bmj.com

BMJ Open

Intussusception in children under-two years, seasonal variations and associations with meteorological parameters: Results from multisite bidirectional surveillance in India over eight years

Journal:	*BMJ Open*
Manuscript ID	bmjopen-2020-043687
Article Type:	Original research
Date Submitted by the Author:	11-Aug-2020
Complete List of Authors:	Study Group, The INCLEN Intussusception Surveillance; The INCLEN Trust International<br>Das, Manoja; The INCLEN Trust International, The INCLEN Trust International
Keywords:	EPIDEMIOLOGY, Public health < INFECTIOUS DISEASES, Community child health < PAEDIATRICS, Paediatric gastroenterology < PAEDIATRICS

SCHOLARONE™
Manuscripts

I, the Submitting Author has the right to grant and does grant on behalf of all authors of the Work (as defined in the below author licence), an exclusive licence and/or a non-exclusive licence for contributions from authors who are: i) UK Crown employees; ii) where BMJ has agreed a CC-BY licence shall apply, and/or iii) in accordance with the terms applicable for US Federal Government officers or employees acting as part of their official duties; on a worldwide, perpetual, irrevocable, royalty-free basis to BMJ Publishing Group Ltd ("BMJ") its licensees and where the relevant Journal is co-owned by BMJ to the co-owners of the Journal, to publish the Work in this journal and any other BMJ products and to exploit all rights, as set out in our licence.

The Submitting Author accepts and understands that any supply made under these terms is made by BMJ to the Submitting Author unless you are acting as an employee on behalf of your employer or a postgraduate student of an affiliated institution which is paying any applicable article publishing charge ("APC") for Open Access articles. Where the Submitting Author wishes to make the Work available on an Open Access basis (and intends to pay the relevant APC), the terms of reuse of such Open Access shall be governed by a Creative Commons licence – details of these licences and which Creative Commons licence will apply to this Work are set out in our licence referred to above.

Other than as permitted in any relevant BMJ Author's Self Archiving Policies, I confirm this Work has not been accepted for publication elsewhere, is not being considered for publication elsewhere and does not duplicate material already published. I confirm all authors consent to publication of this Work and authorise the granting of this licence.

For peer review only

Article Type: Original Research

Title: Intussusception in children under-two years, seasonal variations and associations with meteorological parameters: Results from multisite bidirectional surveillance in India over eight years

Short title: Association of intussusception in children with season and meteorological parameters

Author Information

The INCLEN Intussusception Surveillance Network Study Group

The INCLEN Trust International, Okhla Industrial Area, Phase 1, New Delhi -110020, India

E-mail: drmanojkdas@gmail.com

Corresponding Author

Manoja Kumar Das

Director Projects, The INCLEN Trust International, F1/5, Okhla Industrial Area, Phase 1, New Delhi -110020, INDIA

Phone: Off: +91-11-47730000; Mob: +91-9810203768; Fax: +91-11-47730001

E-mail: manoj@inclentrust.org

For peer review only

Author information

'The INCLEN Intussusception Surveillance Network Study Group' list of authors

1. Manoja Kumar Das

Director Projects, The INCLEN Trust International, New Delhi, India

Email: manoj@inclentrust.org

2. Narendra Kumar Arora

Executive Director, The INCLEN Trust International, New Delhi, India

Email: nkarora@inclentrust.org

3. Bini Gupta

Assistant Research Officer, The INCLEN Trust International, New Delhi, India

Email: drbini.gupta02@gmail.com

4. Sharad Srivastava

Assistant Program Officer, The INCLEN Trust International, New Delhi, India

Email: sharad.srivastav@inclentrust.org

5. Apoorva Sharan

Research Officer, The INCLEN Trust International, New Delhi, India

Email: apoorva@inclentrust.org

6. Arindam Ray

Senior Program Officer, Bill and Melinda Gates Foundation, India Country Office, New Delhi, India

Email: arindam.ray@gatesfoundation.org

7. Ashish Wakhlu

Professor, Department of Paediatric Surgery, King George's Medical University, Lucknow, Uttar Pradesh, India

Email: ashish_wakhlu@hotmail.com

For peer review only

8. Bhadresh R Vyas

Professor, Department of Paediatrics, MP Shah Government Medical College, Jamnagar, Gujarat, India

Email: bhadreshrvyas@yahoo.co.uk

9. Javeed Iqbal Bhat

Assistant Professor, Department of Paediatrics, Sher-I-Kashmir Institute of Medical Sciences, Srinagar, Jammu & Kashmir, India

Email: drjaveediqbal@gmail.com

10. Jayanta K. Goswami

Professor, Department of Paediatric Surgery, Gauhati Medical College, Guwahati, Assam, India

Email: jayantagoswami@hotmail.com

11. John Mathai

Professor, Department of Paediatrics, PSG Institute of Medical Sciences, Coimbatore, Tamil Nadu, India

Email: psg_peds@yahoo.com

12. Kameswari K.

Professor, Department of Paediatric Surgery, Andhra Medical College, Vishakhapatnam, Andhra Pradesh, India

Email: kameswari1956@gmail.com

13. Lalit Bharadia

Consultant Paediatric Gastroenterologist, Fortis Escorts Hospital, Jaipur, Rajasthan, India

Email: lalitbharadia@gmail.com

14. Lalit Sankhe

For peer review only

Assistant Professor, Department of Community Medicine, Grant Medical College & JJ Hospital, Mumbai, Maharashtra, India

Email: sankhelalit@yahoo.com

15. Ajayakumar M.K.

Professor, Department of Paediatric Surgery, Government Medical College & SAT Hospital, Thiruvananthapuram, Kerala, India

Email: drajaykumarmk@gmail.com

16. Neelam Mohan

Consultant Paediatrics Gastroenterology, Medanta—The Medicity, Gurgaon, Haryana, India

Email: drneelam@yahoo.com

17. Pradeep K. Jena

Professor, Department of Paediatric Surgery, SCB Medical College, Cuttack, Odisha, India

Email: drpkjena@gmail.com

18. Rachita Sarangi

Professor, Department of Paediatrics, IMS & SUM Medical College & Hospital, Bhubaneswar, Odisha, India

Email: rachitapaedia@gmail.com

19. Rashmi Shad

Consultant Paediatrics, Choithram Hospital and Research Centre, Indore, Madhya Pradesh, India

Email: drrashmishad@yahoo.com

20. Sanjib K. Debbarma

Associate Professor, Department of Paediatrics, Agartala Government Medical College, Agartala, Tripura, India

For peer review only

For peer review only - http://bmjopen.bmj.com/site/about/guidelines.xhtml

Email: dr_sanjibdb@rediffmail.com

21. Shyamala J.

Consultant Paediatrics, Apollo Hospitals, Chennai, Tamil Nadu

Email: jaymoorthy@hotmail.com

22. Simmi K. Ratan

Professor, Department of Paediatric Surgery, Maulana Azad Medical College, Delhi, India

Email: drjohnsimmi@yahoo.com

23. Suman Sarkar

Assistant Professor, Department of Paediatrics, Institute of Post Graduate Medical Education and Research, Kolkata, West Bengal, India

Email: dr.sumansarkar@gmail.com

24. Vijayendra Kumar

Professor, Department of Paediatric Surgery, Indira Gandhi Institute of Medical Sciences, Patna, Bihar, India

Email: drvijayendrakr@rediffmail.com

25. Narendra Behera

Professor, Department of Pediatrics, MKCG Medical College, Berhampur, Odisha, India

Email: naribehera@yahoo.co.in

26. Rajamani G.

Professor, Department of Paediatric Surgery, Coimbatore Medical College, Coimbatore, Tamil Nadu, India

E mail: drgrajamani@gmail.com

27. Anand P. Dubey

Professor, Department of Paediatrics, Maulana Azad Medical College, Delhi, India

Email: Anand_dubey52@hotmail.com

For peer review only

28. Atul Gupta

Consultant Paediatric Surgery, Fortis Escorts Hospital, Jaipur, Rajasthan, India

Email: atulgupta26878@gmail.com

29. Cenita J. Sam

Professor, Department of Paediatric Surgery, PSG Institute of Medical Sciences, Coimbatore, Tamil Nadu, India

Email: cenitasivamani@yahoo.com

30. Gowhar Nazir Mufti

Assistant Professor, Department of Paediatric Surgery, Sher-I-Kashmir Institute of Medical Sciences, Srinagar, Jammu & Kashmir, India

Email: gowharmufti@yahoo.co.in

31. Harsh Trivedi

Professor, Department of Paediatric Surgery, MP Shah Government Medical College, Jamnagar, Gujarat, India

Email: trivediharsh@indiatimes.com

32. Jimmy Shad

Consultant Paediatric Surgery, Apollo Hospitals, Chennai, Tamil Nadu, India

Email: shadjimmy@yahoo.co.in

33. Kaushik Lahiri

Consultant, Department of Paediatric Surgery, Gauhati Medical College, Guwahati, Assam, India

Email: Kaushik_1671979@rediffmail.com

34. Krishnaswamy R

Chief Medical Officer, Masonic Children's Hospital, Coimbatore, Tamil Nadu, India

Email: krishnaswamy24@yahoo.com

For peer review only - http://bmjopen.bmj.com/site/about/guidelines.xhtml

35. Meera Luthra

Consultant Paediatric Surgery, Medanta- The Medicity, Gurgaon, Haryana, India

Email: meera.luthra@medanta.org

36. Padmalatha P

Professor, Department of Paediatrics, Andhra Medical College, Vishakhapatnam, Andhra Pradesh, India

Email: padmap_2000@yahoo.com

37. Rakesh Kumar

Associate Professor, Department of Paediatrics, Indira Gandhi Institute of Medical Sciences, Patna, Bihar, India

Email: drjaiswalrakesh@gmail.com

38. Ruchirendu Sarkar

Professor, Department of Paediatric Surgery, Institute of Post Graduate Medical Education and Research, Kolkata, West Bengal, India

Email: ruchirendu@gmail.com

39. Santosh Kumar A.

Professor, Department of Paediatrics, Government Medical College & SAT Hospital, Thiruvananthapuram, Kerala, India

Email: hod.pediatrics.sath@gmail.com

40. Subrat Kumar Sahoo

Associate Professor, Department of Paediatric Surgery, IMS & SUM Medical College & Hospital, Bhubaneswar, Odisha, India

Email: dr_subratsahoo@yahoo.com

41. Sunil K. Ghosh

For peer review only

Associate Professor, Department of Pediatric Surgery, Agartala Government Medical College, Agartala, Tripura, India

Email: sunilkrghosh752@gmail.com

42. Sushant Mane

Assistant Professor, Department of Paediatrics, Grant Medical College & JJ Hospital, Mumbai, Maharashtra, India

Email: drsush2006@gmail.com

43. Arun Dash

Professor, Department of Pediatric Surgery, MKCG Medical College, Berhampur, Odisha

Email: dasharun@gmail.com

44. Bashir Ahmad Charoo

Professor, Department of Paediatrics, Sher-I-Kashmir Institute of Medical Sciences, Srinagar, Jammu & Kashmir, India

Email: charoobash@gmail.com

45. Bikasha Bihary Tripathy

Associate Professor, Department of Paediatric Surgery, IMS & SUM Medical College & Hospital, Bhubaneswar, Odisha, India

Email: bbtripathy.dr@gmail.com

46. Rajendra Prasad G.

Professor, Department of Paediatric Surgery, Andhra Medical College, Vishakhapatnam, Andhra Pradesh, India

Email: dr.grajendraprasad@gmail.com

47. Harish Kumar S

Paediatrics Radiologist, Apollo Hospitals, Chennai, Tamil Nadu, India

Email: drharishkumar.s@gmail.com

For peer review only

For peer review only - http://bmjopen.bmj.com/site/about/guidelines.xhtml

48. Jothilakshmi K.

Professor, Department of Paediatrics, PSG Institute of Medical Sciences, Coimbatore, Tamil Nadu, India

Email: drjothi.cbe@gmail.com

49. Nihar Ranjan Sarkar

Associate Professor, Department of Radiology, Institute of Post Graduate Medical Education and Research, Kolkata, West Bengal, India

Email: dr.niharsarkar@gmail.com

50. Pavai Arunachalam

Professor, Department of Paediatric Surgery, PSG Institute of Medical Sciences, Coimbatore, Tamil Nadu, India

Email: pavai4321@yahoo.com

51. Satya Sundar G. Mohapatra

Professor, Department of Radiology, IMS & SUM Medical College & Hospital, Bhubaneswar, Odisha, India

Email: drartifact@gmail.com

52. Saurabh Garge

Consultant Paediatric Surgery, Choithram Hospital and Research Centre, Indore, Madhya Pradesh, India

Email: saurabhgarge8@gmail.com

For peer review only

For peer review only - http://bmjopen.bmj.com/site/about/guidelines.xhtml

Abstract

Objectives: Causes and risk factors of intussusception in children remain mostly unknown. Seasonal variations in intussusception have been reported. We explored the association between intussusception in Indian children and meteorological parameters.

Methods: Data for 2161 children aged 2-24 months with primary intussusception from a multisite (20 sites) bidirectional (retrospective and prospective) surveillance in India during July 2010 to September 2017 were used. The monthly mean meteorological parameters (temperature, sunshine, rainfall, humidity and wind speed) were collected from open source website. The association between monthly intussusception cases and meteorological parameters were examined at pooled, regional and site levels using Pearson's and Spearman's rank-order correlation, factorial analysis-of-variance and Poisson regression or negative binomial regression analyses.

Results: The intussusception cases were highest in summer and lowest in autumn seasons. Pearson's correlation analysis showed that temperature ($p<0.05$), wind speed ($p<0.01$) and humidity ($p<0.01$) were associated with monthly intussusception. Spearman's' rank-order correlation analysis found that temperature ($p<0.05$), wind speed ($p<0.01$) and sunshine ($p<0.05$) were associated with monthly intussusception. Poisson regression analysis resulted that monthly intussusception cases was associated with rising temperature (North region, $p<0.01$ and East region <0.05), sunshine (North region, $p<0.01$), humidity (East region, $p<0.01$), and wind speed (East region, $p<0.01$). Factorial analysis-of-variance revealed significant seasonal difference in intussusception cases for pooled level ($p<0.05$), 2-6 months age group ($0<0.05$) and North region ($p<0.01$). Significant differences in intussusception cases between summer and autumn seasons was observed for pooled ($p<0.01$), children aged 2-6 months ($p<0.05$) and 7-12 months ($p<0.05$). Intussusception cases varied significantly between

For peer review only

For peer review only - http://bmjopen.bmj.com/site/about/guidelines.xhtml

different temperature categories (<20°C, 21°C-25°C, 26°C-30°C and >30°C) for pooled, North and South regions ($p<0.01$).

Conclusions: A seasonal variation in monthly intussusception was observed in India, with a peak in summer months and more evident for three regions. Monthly intussusception cases were positively associated with the monthly mean meteorological parameters, especially temperature.

Keywords: intussusception, children, climate, meteorological parameters, ambient temperature, season, India

For peer review only

For peer review only - http://bmjopen.bmj.com/site/about/guidelines.xhtml

Strengths and limitations of the study

- This study included large number of primary intussusception cases in children under-two tears from 20 sites in India representing different geographic and climate regions over eight years.
- Statistical methods were used to explore the association between meteorological parameters and intussusception.
- Correlation between the meteorological parameters and intussusception was explored using Pearson's correlation and Spearman's rank-order correlation analyses
- Seasonal variation for intussusception across regions was examined using factorial analysis-of-variance and regression analyses.
- With no definite population catchment and referral for the hospitals, the estimation of intussusception incidence was not possible.

For peer review only

For peer review only - http://bmjopen.bmj.com/site/about/guidelines.xhtml

1. Introduction

Intussusception is the most common acute abdomen with intestinal obstruction in children, mostly occurring in infants [1,2]. The incidence of intussusception vary widely with mean 74 per 100,000 (range: 9-328) among infants with peak during 5-7 months of age [3]. The available information from India suggest intussusception incidence to vary from 17.7 to 254 per 100,000 child-years [4,5]. These children usually present with abdominal pain or excessive crying, vomiting, bloody stools and may have abdominal mass. Ultrasound is the leading diagnostic tool. Majority of the patients can be managed by reduction method and some may need surgery. In majority of the children, the aetiology of intussusception remain unknown [6].

Intussusception has attracted attention due to its association with the rotavirus vaccine (RVV). India introduced RVV into the universal immunization program (UIP) in 2016 and expanded countrywide in 2019. A recent publication by our group reported regional variation in the intussusception cases among Indian children under-two year age [7]. More number of cases were observed during March to June months (summer months) suggesting seasonal variation. Other studies from India also reported seasonal variation [5,8-11]. There is variation in seasonality of intussusception occurrence globally. Higher number of intussusception cases have been reported during warmer months from some countries [12-16], while no seasonal variation reported from other countries [17-23]. Reports from China found the intussusception case load to be associated with meteorological parameters like temperature, sunshine and humidity [12,24]. The association between meteorological factors and intussusception remains unclear, due to various reasons including shorter study duration, smaller sample size, and no definite statistical methods to explore the association. Although the seasonal variations have been reported, no association between the intussusception and meteorological parameters have been reported from India.

For peer review only - http://bmjopen.bmj.com/site/about/guidelines.xhtml

A multisite surveillance in India documented the epidemiology and trend of intussusception as part of the vaccine safety surveillance linked to the RVV introduction. Using the data collected by the Indian surveillance network, this study investigated the possible association between intussusception cases and various meteorological parameters and seasonality at site, regional and national levels.

2. Methods

2.1. Study area and participating hospitals

This hospital-based sentinel surveillance for intussusception in under-two children at 20 Indian major tertiary care hospitals included both prospective and retrospective periods. This bidirectional surveillance was comprised of a retrospective (July 1, 2010 to March 31, 2016) and a prospective (April 1, 2016 to September 30, 2017) periods of data collection. The study sites were selected from four regions (north, south, east, and west) of the country ensuring a mix of public and private tertiary care hospitals (North: three public and two private; South: two public and three private; East: five public and one private; West: 2 public, and one private) (Supplementary Figure 1). The study sites represented different climate zones in India. The method of study sites selection have been published in the protocol [25].

2.2. Case definition, case selection and data collection

The children aged 2-24 months admitted to these hospitals with intussusception were eligible. For the retrospective surveillance period, the potential cases were identified using international classification of diseases (ICD 9/10) or diagnoses in medical record section (Supplementary Table 1). These case records were reviewed to identify the intussusception cases and all cases were recruited. For the prospective surveillance period, all age-eligible children were screened,

For peer review only - http://bmjopen.bmj.com/site/about/guidelines.xhtml

For peer review only

tracked till final diagnosis and all intussusception cases were recruited after written informed consent. The cases of first episode of intussusception were identified from the history and/or from parents. For the confirmed cases, data including demography, clinical features, management, and final outcome were abstracted using case record forms. External experts visited the study sites to assess the protocol adherence for case selection, record retrieval and data abstraction. Data team members visited the sites and repeated the case retrieval process for 2014, 2015, and 2016 to assess the retrieval completeness. Data queries were clarified with reference to the source documents.

For peer review only

2.3. Meteorological data collection

For the study sites, meteorological data including monthly mean parameters including temperature (^{0}Centigrade, ^{0}C), sunshine (total hours), rainfall (millimetre, mm), humidity (%) and wind speed (kilometre per hour, Kmph) were collected from the website, World Weather Online (https://www.worldweatheronline.com/). These meteorological data collected for the period July 2010 through September 2017 were available in the public domain.

2.4. Data Management and analysis

The descriptive analysis included proportions, means (with standard deviations), and median (with interquartile range, IQR). There was no definite population catchment area for the hospitals and the estimation of intussusception incidence was not possible. The calendar months were grouped into seasons (summer, rainy, autumn, winter and spring) for the individual sites and regions (Supplementary Table 2). The Chi-squared goodness of fit test was used to examine the periodicity of intussusception and monthly differences. Kruskal-Wallis H test was performed to document the differences between >3 groups (years, months seasons and temperature ranges: <20°C, 21°C-25°C, 26°C-30°C and >30°C). Pearson's correlation analysis

For peer review only - http://bmjopen.bmj.com/site/about/guidelines.xhtml

explored the association between monthly intussusception cases (dependent variable) and monthly mean meteorological parameters separately (independent variables). Spearman's rank-order correlation analysis was also conducted to explore whether the monthly intussusception and monthly mean meteorological parameters covary. Factorial analysis-of-variance was used to analyse the variations in the intussusception cases during the different seasons (dependent variable: seasonal intussusception cases and independent variable: season and year). Poisson regression or negative binomial regression (for parameters with wide dispersion) analyses was performed to evaluate the relationships between the monthly intussusception cases (dependent variable) and the monthly mean meteorological parameters (independent variables). The association between intussusception and meteorological parameters were examined at pooled, regional and site levels with 95% confidence interval (CI) estimation. Statistical significance was considered if $p<0.05$. Statistical analysis was performed using STATA version 15.0 (Stata Corp LLC, Texas, USA).

2.5. Ethical issues

The study protocol was reviewed and approved by all the ethics review committees of each participating institute. Confidentiality in data handling was maintained. The participants under prospective component were recruited after written informed consent from parent/legal guardian.

2.6. Patient and Public Involvement

The study participants or the public were not involved in the design, or conduct, or reporting, or dissemination plans of our research. The findings would be relevant for care seeking and case management for intussusception in children. The results on published shall be disseminated to the public through social media and through healthcare providers for appropriate care.

For peer review only - http://bmjopen.bmj.com/site/about/guidelines.xhtml

3. Results

3.1 Demography data

During July 2010 through September 2017, 2279 children aged 2-24 months with intussusception were recruited. Out of these, 118 children had past history of intussusception, and were excluded. Thus, 2161 primary intussusception cases were analysed. The demography and regional distribution of the intussusception cases across the years is shown in Table 1. There were 1431 (66.2%) boys and 730 (33.8%) girls (male-female ratio, 1.96). Majority (n=1653, 76.5%) were infants. The number of cases increased across the years with highest (31%) jump between 2014 and 2015. During 2010-2014, the children aged 7-12 months had a higher share, which changed during 2015-2017. The South region contributed the highest (50%), followed by East (27.4%), North (17.1%) and West (5.4%) regions, which were consistent across the years.

3.2. Meteorological data

The monthly meteorological parameters for the regions are shown in Supplementary Table 3. The monthly meteorological parameters for each site is given in Supplementary Table 4. Across the regions, April to June and December-February had the highest and lowest temperatures respectively. The pattern of meteorological parameters across the regions is shown in Supplementary Figure 2.

3.3. Monthly and seasonal cycles of intussusception

The Chi-squared goodness of fit test revealed obvious monthly (χ^2-statistic= 71.36; p<0.01) and seasonal differences (χ^2-statistic= 382.28; p<0.01) in the intussusception case load. Also Kruskal-Wallis H test revealed significant differences in the intussusception cases for months

For peer review only - http://bmjopen.bmj.com/site/about/guidelines.xhtml

(p<0.003) and seasons (p<0.001). As shown in Figure 1, more intussusception cases were observed during March in South region, June in North and East regions, all during summer season. Not much variation in cases was observed in West region. The intussusception cases were lowest in September and October months during these years. The peak and lowest seasons were summer (March-June; n=661, 30.6% cases) and autumn (September-October; 14.5% cases), respectively (Supplementary Figure 3).

3.4. Correlation between the intussusception and meteorological factors

Table 2 shows the Pearson's correlation analysis for pooled, regions and age groups. For pooled data, the temperature (p<0.05), wind speed (p<0.01) and humidity (p<0.01) were associated with intussusception. At regional level, intussusception cases had association with temperature for East region (p<0.05); with rainfall for North and East regions (p<0.01); with wind speed for all regions (p<0.01); with humidity for three regions except West region (p<0.01); and with sunshine for North region (p<0.01). For the age groups, intussusception cases had association with temperature for 7-12 months (p<0.01), with wind speed for all age groups (p<0.01), with humidity for 7-12 months and 13-24 months age groups (p<0.01). The Pearson correlation analysis for intussusception cases with the meteorological parameters for the study sites are given in Supplementary Table 5. Significant correlations for intussusception cases with temperature were observed for three sites, with rainfall for two sites, with wind speed and humidity for five sites each and with sunshine for three sites.

Table 3 shows the Spearman's' rank-order correlation analysis for pooled, regions and age groups. For pooled data, the temperature (p<0.05), wind speed (p<0.01) and sunshine (p<0.05) were associated with intussusception. At regional level, intussusception cases had association with temperature for North region (p<0.05); with wind speed for North and East regions

For peer review only

(p<0.01); with humidity for North and East regions (p<0.05); and with sunshine for North region (p<0.05). For the age groups, intussusception cases had association with temperature for 7-12 months (p<0.01), with wind speed for all age groups (p<0.01), with humidity and sunshine for 7-12 months age group (p<0.05). The Spearman's' Rank correlation analysis for intussusception cases with the meteorological parameters for the study sites are given in Supplementary Table 6. Significant correlations for intussusception cases with temperature were observed for one site, with rainfall for two sites, with wind speed for three sites, with humidity for five sites and with sunshine for two sites.

Regression analyses (Poisson regression and negative binomial regression) revealed significant association between intussusception cases and temperature for North (p<0.01) and East (p<0.05) regions; wind speed for North and East regions (p<0.01); humidity for East region (p<0.01) and sunshine for North region (p<0.01) (Table 4). Associations between the intussusception cases and temperature and humidity were evident across different age groups (p<0.01). The association was also significant at study sites level for temperature (three sites), humidity (six sites) and sunshine (five sites) (Supplementary Table 7).

3.5. Correlation between the intussusception, seasons and age groups

Factorial analysis-of-variance revealed significant difference in seasonal intussusception cases at pooled level (p<0.05), for 2-6 months age group (0<0.05) and for North region (p<0.01) (Supplementary Table 8 and 9). Multiple comparison revealed significant difference in intussusception cases between summer and autumn seasons (0.569; 95% CI 0.11-1.03; p<0.01) at pooled level (Supplementary Table 10). Significant differences intussusception cases between summer and monsoon (0.165; 95% CI 0.04-0.29, p<0.01), summer and autumn (0.179; 95% CI 0.03-0.32, p<0.01), spring and monsoon (0.276; 95% CI 0.09-0.46, p<0.01)

For peer review only - http://bmjopen.bmj.com/site/about/guidelines.xhtml

and spring and autumn (0.290; 95% CI 0.09-0.49, $p<0.01$) were observed for North region (Supplementary Table 11). Significant differences intussusception cases were observed between summer and autumn for children aged 2-6 months (0.195; 95% CI 0.01-0.38, $p<0.05$) and for children aged 7-12 months (0.231; 95% CI 0.01-0.45, $p<0.05$) (Supplementary Table 12).

3.6. Correlation between intussusception cases and temperature variations

The monthly average temperatures at the sites and regions varied widely; 14°C to 35°C in North, 23°C to 31°C in South, 20°C to 32°C in East and 22°C to 31°C in West regions. Chi-squared goodness of fit test showed significant difference at 20°C–30°C for pooled, North and South regions ($P<0.01$), indicating that the intussusception cases occurred more during this temperature (Supplementary Table 13). Kruskal-Wallis H test found significant variation in the intussusception cases between the different temperature categories for pooled, North and South regions ($p<0.01$ for all) (Supplementary Table 14).

4. Discussion

The seasonal influence on intussusception cases has been proposed, but with inconsistent findings from different countries and different parts of India. This study is the first to explore association between intussusception and monthly mean meteorological parameters (temperature, rainfall, wind speed, humidity and sunshine) in India. This study comprising of 2161 children with primary intussusception from 20 sites in India documented monthly and seasonal variations. Intussusception case peaks were observed during summer months (Mar-June) and troughs during autumn months (September-October). There were positive association between intussusception cases and temperature and sunshine at pooled and regional levels and among children aged 7-12 months. The associations were consistent across analysis

For peer review only - http://bmjopen.bmj.com/site/about/guidelines.xhtml

tests used (Pearson Correlation, Spearman's Rank correlation tests, Poisson regression or negative binomial regression). The association was more pronounced for North and East regions, where the temperature fluctuation across the months were widest. Further analysis using factorial analysis-of-variance also demonstrated seasonal inclination with peaking in summer season (Mar-June) in India and significant difference from autumn season (with lowest cases) ($p<0.01$). A significant difference in number of intussusception cases between the temperature ranges (<20°C, 21°C -25°C, 26°C -30°C and >30°C) was observed at pooled and regional (North and South regions) levels, which indicated rise in intussusception cases with rise in monthly mean temperature.

The findings are consistent with the report from China in 5994 children (aged 0-12 years) with intussusception, where more cases were documented in summer months with significant positive association with mean monthly meteorological parameters (temperature, sunshine, and precipitation) [12]. A modelling using the data from 13887 Chinese children with intussusception and applying the meteorological variation assumptions achieved considerable agreement with the data collected [24].

Studies on intussusception in children from India have reported higher cases during summer (March-July) and lowest during autumn (September-November) seasons. In three studies from South India, highest and lowest intussusception cases were observed during March-June and September-October respectively [5,9,10]. A multisite study from India reported highest and lowest cases during March and September months respectively [8]. A study from North India observed highest and lowest cases during June and September months [11]. A report from our group on the retrospective surveillance observed higher number of intussusception cases during summer months (March-June) over the year [7].

For peer review only - http://bmjopen.bmj.com/site/about/guidelines.xhtml

Seasonal pattern of intussusception occurrence have been observed from different countries. Higher number of intussusception cases were observed during warmer months (May-October) than cooler months (November-April) in Taiwan [14]. Among Hong Kong children with intussusception, higher cases were observed during summer months (May-July) [26]. Among Ethiopian children, higher intussusception cases were observed during June month and lower during November months [13]. Higher intussusception cases were observed during summer months and lower during winter months among South African children [15]. Among Israeli children, more intussusception cases were observed during summer months and lower during winter [16]. No seasonal variation was also observed in some studies from different countries. Studies from Latin American countries and North America didn't observe visible seasonal variation in cases [17,23]. Studies from Switzerland, France, New Zealand, Singapore and China also had no sizable seasonal variations [18-22].

The inconsistencies in the seasonal variations may be due to the sample size, age groups included and stratification in analysis. Three studies which observed definite seasonal variation [12,14,24] and two observed statistically significant association between the meteorological parameters and intussusception has larger sample size (>5000 participants). The association observed in our study may be also due to higher sample size. The reasons for the higher intussusception cases during summer is not known and the seasonality don't match with the rotavirus infection seasonality in India [27]. Further studies are needed to confirm the seasonality, association with the meteorological parameters and variations in seasonality across the regions in India.

For peer review only - http://bmjopen.bmj.com/site/about/guidelines.xhtml

A seasonal variation in another acute intestinal condition, acute appendicitis in children have bene reported. More acute appendicitis cases in children during summer months and lower during spring/autumn seasons have been reported from India, Pakistan, Iraq, China, Taiwan, Italy, Germany, Canada and United States [28-35]. Among Chinese children, association between acute appendicitis and temperature ($p<0.0001$), humidity ($p<0.0001$) and sunshine ($p<0.0001$) were documented to support the seasonal variation [31]. The intussusception and acute appendicitis, both are intestinal problems and some of the intussusception have associated appendicitis. Thus the linkage between seasons and meteorological parameters and acute intestinal conditions appears to be plausible, may mediated by intestinal infections (including activation of latent viral infections), inflammatory changes, lymphoid hyperplasia, or motility alterations. Thus the seasonal variation in these conditions and association between meteorological parameters appear epidemiologically and biologically plausible, which needs careful evaluation.

The study had several strengths; large number of intussusception cases over seven years period, sites from different regions, and statistical methods used and consistency in the findings. The consistency across the results using different statistical tests was assuring. There are several limitations in the study. A major part of this study was retrospective. The sites belong to different climate zones and had variation in the meteorological parameters. Lack of definite catchment population and referral pattern, made it difficult to estimate the intussusception incidence rate.

In conclusion, this study documented higher intussusception cases during the summer months with a positive association between number of cases with temperature and sunshine. The association was significant for the North and East regions with wider variations in the

For peer review only - http://bmjopen.bmj.com/site/about/guidelines.xhtml

534 temperature. Higher degree of suspicion for infants with compatible clinical presentation for
535 intussusception during the summer months may improve diagnosis and clinical care to prevent
536 the complications. Further studies are needed to confirm the observation and improve the
537 evidence base on the potential risk factors for the season variations and the aetiologies of
538 intussusception.

For peer review only

For peer review only - http://bmjopen.bmj.com/site/about/guidelines.xhtml

Acknowledgments

We acknowledge the support from Ministry of Health and Family Welfare, Government of India for undertaking the study. We are thankful to the hospital administrations and the clinicians at the study site institutes, who supported and facilitated undertaking of the study.

We highly value the technical guidance and inputs provided by the members of Technical Advisory Group: Mahesh K Agarwal and Pradeep Haldar, Ministry of Health and Family Welfare, Government of India; Satinder Aneja, Anju Seth and Archana Puri, Lady Hardinge Medical College, New Delhi; Ashok Patwari, Hamdard Institute of Medical Sciences & Research, New Delhi; Yogesh Kumar Sarin, Maulana Azad Medical College, New Delhi; Rakesh Aggarwal, Anshu Srivastava and Ujjal Poddar, Sanjay Gandhi Postgraduate Institute of Medical Sciences, Lucknow; Malathi Satyasekharan, Kanchi Kamakoti Chailds Trust Hospital, Chennai; Raju Sharma and Nirupam Madan, All India Institute of Medical Sciences, New Delhi; Jyoti Joshi and Deepak Polpakara, Immunization Technical Support Unit; Ministry of Health & Family Welfare, New Delhi; Umesh D. Parashar; Centers of Disease Control and Prevention, Atlanta, USA; Jan Bonhoeffer, University Children's Hospital, Basel, Switzerland and Brighton Collaboration; Naveen Thacker, Child Health Foundation, Gandhigram; Rashmi Arora, Indian Council of Medical Research, Ansari Nagar, New Delhi; and Patrick L F Zuber and Christine G. Maure, World Health Organisation, Geneva, Switzerland.

We acknowledge the contribution of the research staffs at The INCLEN Trust International: Harshpreet Kaur, Janvi Chaubey, Mrimmaya Das, Shweta Sharma and Vaibhav Jain.

We highly appreciate the efforts made by the research staffs at the study sites: Aarezo Bashir and Rafia; Sher-e-Kashmir Institute of Medical Sciences, Srinagar, Jammu & Kashmir; Prabha Shankar, Medanta-The Medicity Hospital, Gurgaon, Haryana; Anju Sharma; Maulana Azad Medical College, New Delhi; Anita Singh and Shubhanshu Srivastava, King George Medical University, Lucknow, Uttar Pradesh; Hemant Meena, Choithram Hospital, Indore, Madhya

For peer review only

Pradesh; Pankaj Kumar and Shashi Kant; Indira Gandhi Institute of Medical Sciences, Patna, Bihar; Goutam Benia, IMS & SUM Medical College & Hospital, Bhubaneshwar, Odisha; Prasntajyoti Mohanty, SVP Post Graduate Institute of Paediatrics, Cuttack, Odisha; Asit Pradhan, MKCG Medical College, Berhampur, Odisha; Angshuman Chatterjee, Institute of Postgraduate Medical Education and Research & SSKM Hospital, Kolkata, West Bengal; S. Yamuna, Andhra Medical College, Vishakhapatnam, Andhra Pradesh; Srinidhi Sudan, Apollo Hospitals, Hyderabad, Telengana; Rajesh Francis, Apollo Hospitals, Chennai, Tamil Nadu; T. Easter Chandru, PSG Institute of Medical Sciences, Coimbatore, Tamil Nadu; Deepthy R, Julie and Anju Shivakumar, Government Medical College & SAT Hospital, Thiruvananthapuram, Kerala; Archit Vaidya, Grant Medical College & JJ Hospital, Mumbai, Maharashtra; Nimesh Chouksey, MP Shah Government Medical College, Jamnagar, Gujarat; Nidhi Singh, Fortis Escorts Hospital, Jaipur, Rajasthan; Mrinmoy Gohain, Gauhati Medical College, Guwahati, Assam; Arpita Bhattachrjee, Saugat Ghosh and Tanusmita Debnath, Agartala Government Medical College, Agartala, Tripura.

For peer review only

For peer review only - http://bmjopen.bmj.com/site/about/guidelines.xhtml

Declarations

Ethics approval and consent to participate: The study protocol was reviewed and approved by The INCLEN Independent Ethics Committee, The INCLEN Trust International, New Delhi, India (Ref: IIEC 23) and all participating Study Site Institute Ethics Committees. The interviews with stakeholders were done after obtaining written informed consent.

Consent for publication: Not applicable

Availability of data and materials: All data is available with the investigators and can be provided by the corresponding author upon reasonable request.

Competing interests: The authors declare that there is no competing interests and conflict of interest.

Disclosure statement: None. There is no financial interest or benefit for the authors arisen from this project or its direct application.

Funding: This project was supported by the Bill and Melinda Gates Foundation, USA to The INCLEN Trust International (grant number OPP1116433). The funder or its representative had no role in the design of the study and collection, analysis, and interpretation of data and writing the manuscript.

Authors' contributions:

Study conceptualisation, study design, protocol development, training, data analysis, interpretation: MKD and NKA.

Study coordination, monitoring, and data cleaning: MKD, BG, and AS.

Data analysis, interpretation, and manuscript preparation: MKD, BG and SS-1.

Protocol development, quality assurance and monitoring: AR

Participant recruitment and data collection: AW, BRV, JIB, JKG, JM, KK, LB, LS, AMK, NM, PKJ, RS-1, RS-2, SKD, SJ, SKR, SS-2, VK, NB, RG, APD, AG, CJS, GNM, HT, JS, KL, KR,

For peer review only

For peer review only - http://bmjopen.bmj.com/site/about/guidelines.xhtml

ML, PP, RK, RS-3, SKA, SKS, SKG, SM, AD, BAC, BBT, RPG, HKS, JK, NRS, PA, SSGM, and SG.

All authors reviewed, provided critical input and approved the final version.

For peer review only

For peer review only - http://bmjopen.bmj.com/site/about/guidelines.xhtml

List of abbreviations

CI	Confidence interval
ICD	International Classification of Diseases
IQR	Interquartile range
RVV	Rotavirus vaccine
UIP	Universal immunization programme

For peer review only

For peer review only - http://bmjopen.bmj.com/site/about/guidelines.xhtml

References

1. Bines JE, Kohl KS, Forster J, Zanardi LR, Davis RL, Hansen J, et al. Acute intussusception in infants and children as an adverse event following immunization: case definition and guidelines of data collection, analysis, and presentation. Vaccine. 2004 Jan;22(5–6):569–74.
2. Bines JE, Ivanoff B. Acute intussusception in infants and children: Incidence, clinical presentation and management: a global perspective [Internet]. World Health Organisation; 2002 [cited 2017 May 10]. Available from: http://apps.who.int/iris/bitstream/handle/10665/67720/WHO_V-B_02.19_eng.pdf;jsessionid=74671C3CD99ED55137202F8D3D07CB73?sequence=1
3. Jiang J, Jiang B, Parashar U, Nguyen T, Bines J, Patel MM. Childhood Intussusception: A Literature Review. Cameron DW, editor. PLoS ONE. 2013 Jul 22;8(7):e68482.
4. Bahl R, Saxena M, Bhandari N, Taneja S, Mathur M, Parashar UD, et al. Population-Based Incidence of Intussusception and a Case-Control Study to Examine the Association of Intussusception with Natural Rotavirus Infection among Indian Children. The Journal of Infectious Diseases. 2009 Nov;200(s1):S277–81.
5. Jehangir S, John J, Rajkumar S, Mani B, Srinivasan R, Kang G. Intussusception in southern India: Comparison of retrospective analysis and active surveillance. Vaccine. 2014 Aug;32:A99–103.
6. Jain S, Haydel MJ. Child Intussusception. In: StatPearls [Internet]. Treasure Island (FL): StatPearls Publishing; 2020 [cited 2020 Jul 19]. Available from: http://www.ncbi.nlm.nih.gov/books/NBK431078/
7. Das MK, Arora NK, Gupta B, Sharan A, Kameswari K, Padmalatha P, et al. Intussusception in children aged under two years in India: Retrospective surveillance at nineteen tertiary care hospitals. Vaccine. 2020 Jun;S0264410X20305636.

For peer review only - http://bmjopen.bmj.com/site/about/guidelines.xhtml

8. Singh JV, Kamath V, Shetty R, Kumar V, Prasad R, Saluja T, et al. Retrospective surveillance for intussusception in children aged less than five years at two tertiary care centers in India. Vaccine. 2014 Aug;32:A95–8.

9. Bhowmick K, Kang G, Bose A, Chacko J, Boudville I, Datta SK, et al. Retrospective surveillance for intussusception in children aged less than five years in a South Indian tertiary-care hospital. J Health Popul Nutr. 2009 Oct;27(5):660–5.

10. Srinivasan R, Girish Kumar CP, Naaraayan SA, Jehangir S, Thangaraj JWV, Venkatasubramanian S, et al. Intussusception hospitalizations before rotavirus vaccine introduction: Retrospective data from two referral hospitals in Tamil Nadu, India. Vaccine. 2018 Dec;36(51):7820–5.

11. Gupta M, Kanojia R, Singha R, Tripathy JP, Mahajan K, Saxena A, et al. Intussusception Rate Among Under-Five-Children Before Introduction of Rotavirus Vaccine in North India. Journal of Tropical Pediatrics. 2018 Aug 1;64(4):326–35.

12. Guo W, Zhang S, Li J, Wang J. Association of Meteorological Factors with Pediatric Intussusception in Subtropical China: A 5-Year Analysis. Wang L, editor. PLoS ONE. 2014 Feb 28;9(2):e90521.

13. Gadisa A, Tadesse A, Hailemariam B. Patterns and seasonal variation of intussusception in children: a retrospective analysis of cases operated in a tertiary hospital in Ethiopia. Ethiop Med J. 2016 Jan;54(1):9–15.

14. Chen SC-C, Wang J-D, Hsu H-Y, Leong M-M, Tok T-S, Chin Y-Y. Epidemiology of childhood intussusception and determinants of recurrence and operation: analysis of national health insurance data between 1998 and 2007 in Taiwan. Pediatr Neonatol. 2010 Oct;51(5):285–91.

For peer review only

For peer review only - http://bmjopen.bmj.com/site/about/guidelines.xhtml

15. Moore SW, Kirsten M, Müller EW, Numanoglu A, Chitnis M, Le Grange E, et al. Retrospective surveillance of intussusception in South Africa, 1998-2003. J Infect Dis. 2010 Sep 1;202 Suppl:S156-161.

16. Greenberg D, Givon-Lavi N, Newman N, Wheeler J, Cohen Z, Dagan R. Intussusception in Children in Southern Israel: Disparity between 2 Populations. The Pediatric Infectious Disease Journal. 2008 Mar;27(3):236–40.

17. Sáez-Llorens X, Velázquez FR, Lopez P, Espinoza F, Linhares AC, Abate H, et al. A multi-country study of intussusception in children under 2 years of age in Latin America: analysis of prospective surveillance data. BMC Gastroenterol. 2013 May 27;13:95.

18. Buettcher M, Baer G, Bonhoeffer J, Schaad UB, Heininger U. Three-year surveillance of intussusception in children in Switzerland. Pediatrics. 2007 Sep;120(3):473–80.

19. Serayssol C, Abbo O, Mouttalib S, Claudet I, Labarre D, Galinier P, et al. [Seasonal pattern of intussusceptions in infants and children: is fall/winter predominance still worth consideration? A 10-year retrospective epidemiological study]. Arch Pediatr. 2014 May;21(5):476–82.

20. Chen YE, Beasley S, Grimwood K, and the New Zealand Rotavirus Study Group. Intussusception and rotavirus associated hospitalisation in New Zealand. Archives of Disease in Childhood. 2005 Jun 7;90(10):1077–81.

21. Boudville IC, Phua KB, Quak SH, Lee BW, Han HH, Verstraeten T, et al. The epidemiology of paediatric intussusception in Singapore: 1997 to 2004. Ann Acad Med Singap. 2006 Oct;35(10):674–9.

22. Liu N, Yen C, Huang T, Cui P, Tate JE, Jiang B, et al. Incidence and epidemiology of intussusception among children under 2 years of age in Chenzhou and Kaifeng, China, 2009-2013. Vaccine. 2018 14;36(51):7862–7.

For peer review only - http://bmjopen.bmj.com/site/about/guidelines.xhtml

23. Chang HG, Smith PF, Ackelsberg J, Morse DL, Glass RI. Intussusception, rotavirus diarrhea, and rotavirus vaccine use among children in New York state. Pediatrics. 2001 Jul;108(1):54–60.

24. Guo W, Geng J, Zhan Y, Tan Y, Hu Z, Pan P, et al. Forecasting and predicting intussusception in children younger than 48 months in Suzhou using a seasonal autoregressive integrated moving average model. BMJ Open. 2019 Jan;9(1):e024712.

25. Das M, Arora N, Bonhoeffer J, Zuber P, Maure C. Intussusception in Young Children: Protocol for Multisite Hospital Sentinel Surveillance in India. Methods and Protocols. 2018 Mar 22;1(2):11.

26. Nelson EAS, Tam JS, Glass RI, Parashar UD, Fok TF. Incidence of rotavirus diarrhoea and intussusception in Hong Kong using standardized hospital discharge data. The Pediatric Infectious Disease Journal. 2002 Jul;21(7):701–3.

27. Giri S, Nair NP, Mathew A, Manohar B, Simon A, Singh T, et al. Rotavirus gastroenteritis in Indian children < 5 years hospitalized for diarrhoea, 2012 to 2016. BMC Public Health. 2019 Dec;19(1):69.

28. Jangra B, Jangra M, Rattan K, Kadian Y. Seasonal and day of week variations in acute appendicitis in north Indian children. J Indian Assoc Pediatr Surg. 2013;18(1):42.

29. Ahmed W, Akhtar MS, Khan S. Seasonal variation of acute appendicitis. Pak J Med Sci [Internet]. 2018 May 24 [cited 2020 Aug 4];34(3). Available from: http://pjms.com.pk/index.php/pjms/article/view/14793

30. Al-Mendalawi M, Lattuf M. Is there a seasonal distribution of acute appendicitis in Iraqi children? Menoufia Med J. 2017;30(2):572.

31. Zhang Y, Lyu F, Kang Q, Dong Z, Zhao T, Xie S, et al. Association of meteorological factors with pediatric acute appendicitis in China: A 7-year retrospective analysis. Medicine. 2018 Oct;97(42):e12913.

For peer review only

For peer review only - http://bmjopen.bmj.com/site/about/guidelines.xhtml

32. Hsu Y-J, Fu Y-W, Chin T. Seasonal variations in the occurrence of acute appendicitis and their relationship with the presence of fecaliths in children. BMC Pediatr. 2019 Dec;19(1):443.

33. Gallerani M, Boari B, Anania G, Cavallesco G, Manfredini R. Seasonal variation in onset of acute appendicitis. Clin Ter. 2006 Apr;157(2):123–7.

34. Reinisch A, Heil J, Woeste G, Bechstein W, Liese J. The meteorological influence on seasonal alterations in the course of acute appendicitis. Journal of Surgical Research. 2017 Sep;217:137–43.

35. Fares A. Summer appendicitis. Ann Med Health Sci Res. 2014;4(1):18.

For peer review only

For peer review only - http://bmjopen.bmj.com/site/about/guidelines.xhtml

Tables

Table 1: Distribution and demography of the children with intussusception in India

Pooled	Years								
Years	2010[a]	2011	2012	2013	2014	2015	2016	2017[b]	Total
Number of cases, n	68	209	244	260	278	365	429	308	2161
Gender									
Male, n (%)	45 (66.2)	142 (67.9)	155 (63.5)	171 (65.8)	194 (69.8)	243 (66.6)	283 (66)	198 (64.3)	1431 (66.2)
Female, n (%)	23 (33.8)	67 (32.1)	89 (36.5)	89 (34.2)	84 (30.2)	122 (33.4)	146 (34)	110 (35.7)	730 (33.8)
Age groups									
2-6 months, n (%)	24 (35.3)	73 (34.9)	89 (36.5)	86 (33.1)	91 (32.7)	134 (36.7)	159 (37.1)	122 (39.6)	778 (36)
7-12 months, n (%)	33 (48.5)	92 (44)	108 (44.3)	122 (46.9)	113 (40.6)	135 (37)	155 (36.1)	117 (38)	875 (40.5)
13-24 months, n (%)	11 (16.2)	44 (21.1)	47 (19.3)	52 (20)	74 (26.6)	96 (26.3)	115 (26.8)	69 (22.4)	508 (23.5)
Regions									
North, n (%)	9 (13.2)	19 (9.1)	37 (15.2)	44 (16.9)	48 (17.3)	61 (16.7)	91 (21.2)	61 (19.8)	370 (17.1)
South, n (%)	50 (73.5)	134 (64.1)	130 (53.2)	149 (57.3)	124 (44.6)	175 (47.9)	183 (42.7)	136 (44.2)	1081 (50)
East, n (%)	9 (13.2)	45 (21.5)	61 (25)	54 (20.8)	84 (30.2)	112 (30.7)	131 (30.5)	97 (31.5)	593 (27.4)
West, n (%)	0 (0)	11 (5.3)	16 (6.6)	13 (5)	22 (7.9)	17 (4.7)	24 (5.6)	14 (4.5)	117 (5.4)

Note: a: The period of data collection was 6 months (July to December)
b: The period of data collection was 9 months (January to September)

For peer review only

For peer review only - http://bmjopen.bmj.com/site/about/guidelines.xhtml

724 Table 2. Pearson correlation analysis between monthly intussusception cases and monthly
725 mean meteorological parameters
726

Parameter	Pearson correlation coefficient (r) for meteorological parameters				
	Temperature (°C)	Rainfall (mm)	Wind speed (m/s)	Humidity (%)	Sunshine (hr)
Pooled (n= 2161)	.056*	-.025	.134**	.075**	.045
Regions					
North (n= 370)	.018	-.103**	-.119**	-.373**	.163**
South (n= 1081)	.022	-.023	.210**	.161**	-.018
East (n= 593)	.052*	.072**	-.084**	.109**	.024
West (n= (117)	.046	-.019	.107**	-.029	-.012
Age groups					
2-6 months (n= 778)	.025	-.022	.092**	.040	.037
7-12 months (n= 875)	.068**	-.012	.115**	.069**	.042
13-24 months (n= 508)	.040	-.030	.125**	.076**	.029

727 *Note: Pearson correlation coefficient (r); Significance two tailed*
728 ** Significance level- p value <0.05; ** Significance level- p value <0.01*

For peer review only

Table 3. Spearman's rank-order correlation analysis between monthly intussusception cases and monthly mean meteorological parameters

Parameter	Spearman's rank-order correlation coefficient (ρ) for meteorological parameters				
	Temperature (°C)	Rainfall (mm)	Wind speed (m/s)	Humidity (%)	Sunshine (hr)
Pooled (n= 2161)	.049*	.012	.096**	.005	.051*
Regions					
North (n= 370)	.110*	-.014	.183**	-.114*	.119*
South (n= 1081)	.028	-.031	-.024	-.027	.036
East (n= 593)	.061	-.042	.109**	-.102*	.038
West (n= (117)	.052	-.044	.000	-.067	.076
Age groups					
2-6 months (n= 778)	.035	.031	.063**	.007	.044
7-12 months (n= 875)	.064**	.036	.103**	.049*	.048*
13-24 months (n= 508)	.028	.019	.106**	.043	.016

Note: Spearman's rank-order correlation coefficient (ρ); Significance two tailed
** Significance level- p value <0.05; ** Significance level- p value <0.01*

For peer review only

For peer review only - http://bmjopen.bmj.com/site/about/guidelines.xhtml

Table 4: Regression analysis of the relationship between the monthly intussusception cases and monthly mean meteorological parameters

Variables	Regression coefficient, β (95% CI)	Standard error, S_β	χ2 - value	p value
Pooled#				
Temperature (^{0}C)	0.026 (0.011, 0.041)	0.008	12.098	0.001
Rainfall (mm)	-0.001 (-0.003, 0)	0.001	2.462	0.117
Wind Speed (Kmph)	0.082 (0.061, 0.103)	0.011	58.449	0.000
Humidity (%)	0.011 (0.007, 0.016)	0.002	21.903	0.000
Sunshine (hr)	0.002 (0, 0.003)	0.001	7.000	0.008
According to regions				
North region#				
Temperature (^{0}C)	0.027 (0.007, 0.047)	0.0102	7.195	0.007
Rainfall (mm)	0.001 (-0.005, 0.007)	0.0029	0.169	0.681
Wind Speed (Kmph)	0.208 (0.101, 0.315)	0.0545	14.556	0.000
Humidity (%)	-0.010 (-0.020, 0.001)	0.0052	3.448	0.063
Sunshine (hr)	0.004 (0.002, 0.007)	0.0013	11.006	0.001
South region#				
Temperature (^{0}C)	0.010 (-0.037, 0.058)	0.0243	0.174	0.676
Rainfall (mm)	-0.004 (-0.009, 0.000)	0.0024	3.251	0.071
Wind Speed (Kmph)	-0.011 (-0.045, 0.023)	0.0173	0.401	0.527
Humidity (%)	-0.033 (-0.070, 0.005)	0.0190	2.949	0.086
Sunshine (hr)	0.002 (-0.001, 0.004)	0.0014	1.213	0.271
East region#				
Temperature (^{0}C)	0.029 (0.001, 0.057)	0.0141	4.245	0.039
Rainfall (mm)	-0.001 (-0.004, 0.001)	0.0014	0.766	0.381
Wind Speed (Kmph)	0.091 (0.042, 0.141)	0.0251	13.244	0.000
Humidity (%)	-0.021 (-0.035, -0.007)	0.0070	9.188	0.002
Sunshine (hr)	0.002 (-6.100, 0.005)	0.0012	3.822	0.051
*West region**				
Temperature (^{0}C)	0.052 (-0.02, 0.124)	1.987	0.036	0.159
Rainfall (mm)	-0.002 (-0.006, 0.002)	0.855	0.002	0.355
Wind Speed (Kmph)	0.022 (-0.042, 0.087)	0.463	0.032	0.496
Humidity (%)	-0.002 (-0.016, 0.011)	0.105	0.006	0.746
Sunshine (hr)	0.003 (-0.001, 0.007)	2.429	0.002	0.119
According to age groups				
*2-6 months**				
Temperature (^{0}C)	0.012 (-0.004, 0.028)	.008	2.192	0.139
Rainfall (mm)	-0.001 (-0.003, 0.001)	.001	1.599	0.206
Wind Speed (Kmph)	0.059 (0.037, 0.081)	.011	28.241	0.000
Humidity (%)	0.006 (0.001, 0.011)	.003	5.276	0.022
Sunshine (hr)	0.002 (0.000, 0.003)	.001	4.615	0.032
7-12 months#				
Temperature (^{0}C)	0.038 (0.018, 0.057)	.010	14.288	0.000
Rainfall (mm)	-0.001 (-0.003, 0.002)	.001	.447	0.504
Wind Speed (Kmph)	0.08 (0.054, 0.107)	.013	36.037	0.000
Humidity (%)	0.012 (0.006, 0.018)	.003	14.660	0.000

For peer review only

For peer review only - http://bmjopen.bmj.com/site/about/guidelines.xhtml

Sunshine (hr)	0.002 (0.000, 0.004)	.001	5.144	0.023
*13-24 months**				
Temperature (^{0}C)	0.026 (0.006, 0.047)	.010	6.460	0.011
Rainfall (mm)	-0.002 (-0.005, 0.000)	.001	3.564	0.059
Wind Speed (Kmph)	0.102 (0.077, 0.128)	.013	60.620	0.000
Humidity (%)	0.017 (0.010, 0.024)	.004	22.599	0.000
Sunshine (hr)	0.002 (0.000, 0.004)	.001	3.211	0.073

*Note: Tests used for regression analysis: *Poisson Regression; # Negative Binomial Regression*

For peer review only - http://bmjopen.bmj.com/site/about/guidelines.xhtml

739 **Figure**

741 Figure 1: Monthly distribution of primary intussusception cases in children during 2010-2017

For peer review only

For peer review only - http://bmjopen.bmj.com/site/about/guidelines.xhtml

Supplementary Documents

Sl no	Details
1	Supplementary Figure 1: The study sites and their location on India map indicating the study regions
2	Supplementary Table 1: The International Classification of Diseases (ICD) codes for review of the cases from medical records
3	Supplementary Table 2: The seasons and months at the sites according to the regions
4	Supplementary Table 3: The monthly median (and IQR) meteorological data during 2010 and 2017 for regions
5	Supplementary Table 4: Monthly median (and IQR) meteorological data during 2010 and 2017 at the study sites
6	Supplementary Figure 2: The pattern of monthly meteorological parameters across the regions
7	Supplementary Figure 3: The pooled monthly (SF3a) and seasonal (SF3b) distribution of intussusception in children during July 2010 to September 2017
8	Supplementary Table 5. Pearson correlation analysis between monthly intussusception cases and monthly median meteorological parameters for the study sites
9	Supplementary Table 6. Spearman's Rank correlation analysis between monthly intussusception cases and monthly median meteorological parameters for the study sites
10	Supplementary Table 7. Regression analysis of the association between the intussusception cases and meteorological parameters for the study sites
11	Supplementary Table 8. Factorial analysis-of-variance of intussusception in children with season and age groups
12	Supplementary Table 9. Factorial analysis-of-variance of intussusception in children with season and regions
13	Supplementary Table 10. Multiple comparisons of seasonal intussusception in children in different seasons pooled for all sites
14	Supplementary Table 11. Multiple comparisons of seasonal intussusception in children in different seasons at regional level
15	Supplementary Table 12. Multiple comparisons of seasonal intussusception in children in different seasons for different age groups
16	Supplementary Table 13. Intussusception cases in children at different temperature ranges at pooled and regional level
17	Supplementary Table 14. Variations in the intussusception cases between the different temperature categories by Kruskal-Wallis H test

For peer review only

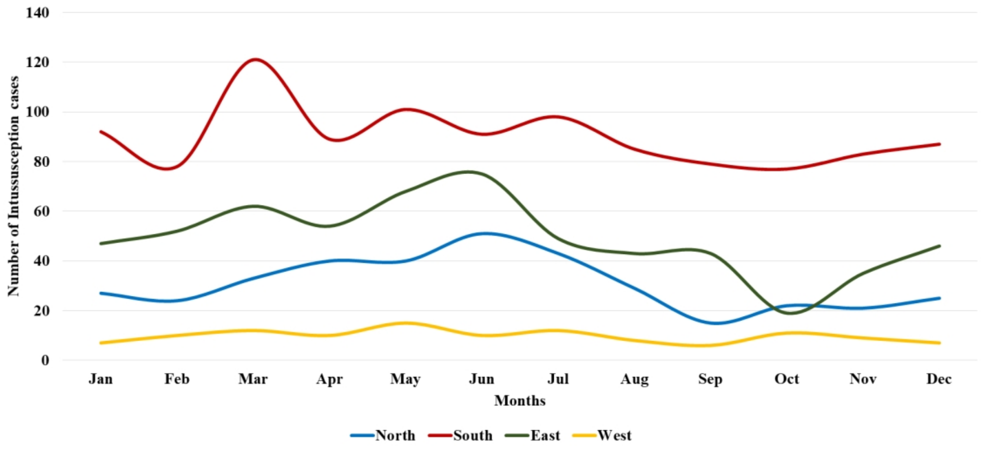

Figure 1: Monthly distribution of primary intussusception cases in children during 2010-2017

155x71mm (300 x 300 DPI)

Title: Intussusception in children under-two years, seasonal variations and associations with meteorological parameters: Results from multisite bidirectional surveillance in India over eight years

Supplementary Documents

Index

Sl no	Details	Page no
1	Supplementary Figure 1: The study sites and their location on India map indicating the study regions	2
2	Supplementary Table 1: The International Classification of Diseases (ICD) codes for review of the cases from medical records	3
3	Supplementary Table 2: The seasons and months at the sites according to the regions	4
4	Supplementary Table 3: The monthly median (and IQR) meteorological data during 2010 and 2017 for regions	5
5	Supplementary Table 4: Monthly median (and IQR) meteorological data during 2010 and 2017 at the study sites	7
6	Supplementary Figure 2: The pattern of monthly meteorological parameters across the regions	12
7	Supplementary Figure 3: The pooled monthly (SF3a) and seasonal (SF3b) distribution of intussusception in children during July 2010 to September 2017	14
8	Supplementary Table 5. Pearson correlation analysis between monthly intussusception cases and monthly median meteorological parameters for the study sites	15
9	Supplementary Table 6. Spearman's Rank correlation analysis between monthly intussusception cases and monthly median meteorological parameters for the study sites	16
10	Supplementary Table 7. Regression analysis of the association between the intussusception cases and meteorological parameters for the study sites	17
11	Supplementary Table 8. Factorial analysis-of-variance of intussusception in children with season and age groups	20
12	Supplementary Table 9. Factorial analysis-of-variance of intussusception in children with season and regions	21
13	Supplementary Table 10. Multiple comparisons of seasonal intussusception in children in different seasons pooled for all sites	22
14	Supplementary Table 11. Multiple comparisons of seasonal intussusception in children in different seasons at regional level	23
15	Supplementary Table 12. Multiple comparisons of seasonal intussusception in children in different seasons for different age groups	25
16	Supplementary Table 13. Intussusception cases in children at different temperature ranges at pooled and regional level	27
17	Supplementary Table 14. Variations in the intussusception cases between the different temperature categories by Kruskal-Wallis H test	28

For peer review only - http://bmjopen.bmj.com/site/about/guidelines.xhtml

Supplementary Figure 1: The study sites and their location on India map indicating the study regions

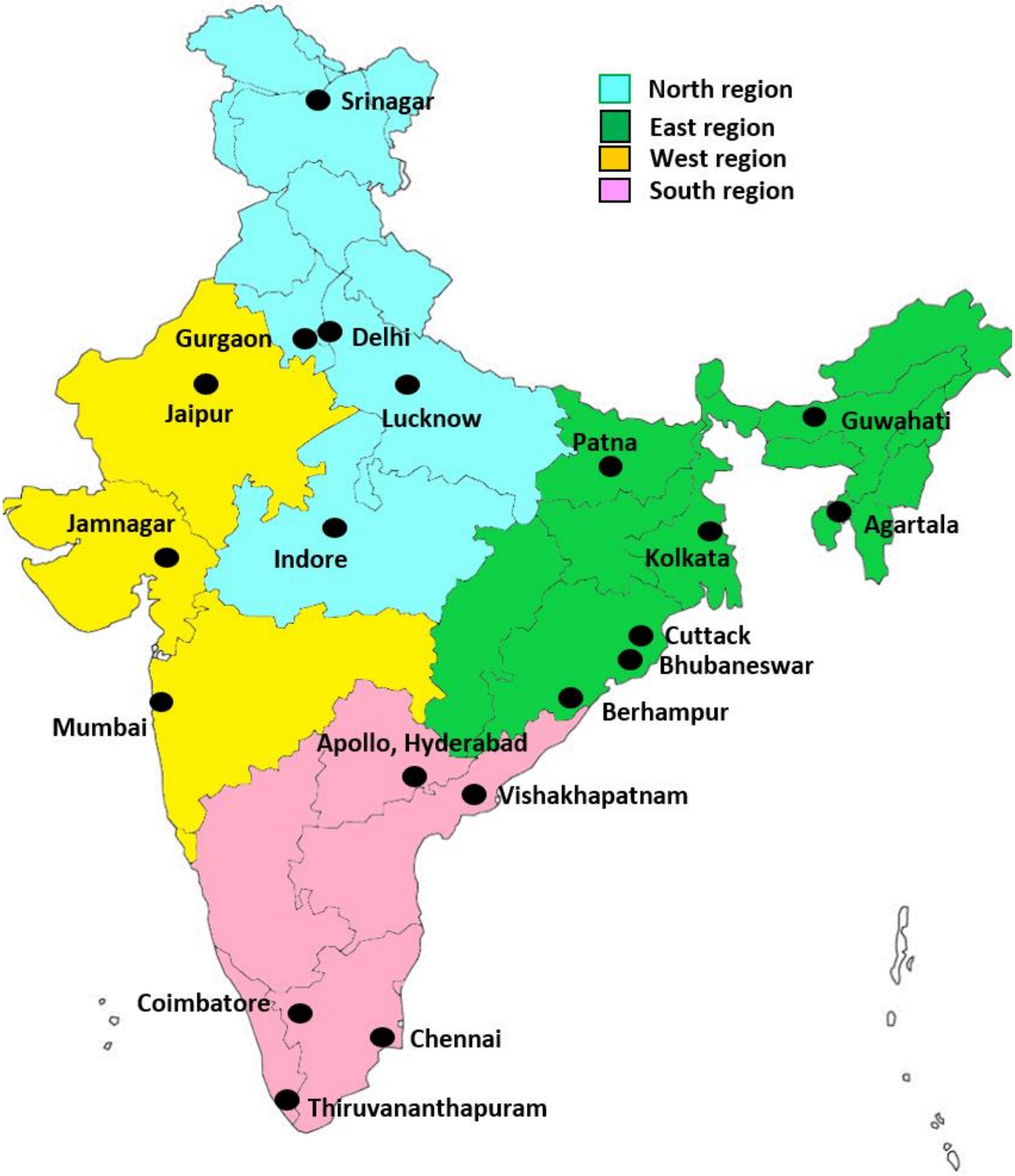

Note: The map has been prepared for use in the research project. The corresponding author is the creator of the image.

For peer review only - http://bmjopen.bmj.com/site/about/guidelines.xhtml

Supplementary Table 1: The International Classification of Diseases (ICD) codes for review of the cases from medical records

Clinical conditions considered as suspected cases	Codes	
	ICD 10	ICD 9
Intussusception	K56.1	560.0
Volvulus	K56.2	560.2
Gallstone ileus	K56.3	560.31
Other impaction of intestine	K56.4	560.30
Intestinal adhesions with obstruction	K56.5	560.81
Other and unspecified intestinal obstruction or Acute intestinal obstruction or Subacute intestinal obstruction	K56.6	560.9
Ileus, unspecified	K56.7	560.1
Paralytic ileus	K56.0	
Acute abdomen	R10.0	789.0
Blood in stool with vomiting	K92.1/K92.2 with R11.2	578.1/578.9 with 787.01

For peer review only

For peer review only - http://bmjopen.bmj.com/site/about/guidelines.xhtml

Supplementary Table 2: The seasons and months at the sites according to the regions

Region/ Site (state)	Jan	Feb	Mar	Apr	May	Jun	Jul	Aug	Sep	Oct	Nov	Dec
North region												
Srinagar (Jammu & Kashmir)	W	W	W	Sp	Sp	Su	Su	M	M	A	W	W
New Delhi (Delhi)	W	Sp	Sp	Su	Su	Su	M	M	M	A	A	W
Gurgaon (Haryana)	W	Sp	Sp	Su	Su	Su	M	M	M	A	A	W
Lucknow (Uttar Pradesh)	W	W	Sp	Su	Su	Su	M	M	M	A	A	W
Indore (Madhya Pradesh)	W	W	Sp	Su	Su	Su	M	M	M	A	A	W
South region												
Vishakhapatnam (Andhra Pradesh)	W	W	Su	Su	Su	M	M	M	M	A	A	W
Hyderabad (Telangana)	W	Sp	Su	Su	Su	M	M	M	M	A	A	W
Coimbatore (Tamil Nadu)	W	Sp	Su	Su	Su	M	M	M	M	A	A	W
Chennai (Tamil Nadu)	W	Sp	Su	Su	Su	M	M	M	M	A	A	W
Trivandrum (Kerala)	W	Sp	Su	Su	Su	M	M	M	M	A	A	W
East region												
Patna (Bihar)	W	Sp	Sp	Su	Su	Su	M	M	M	A	A	W
Bhubaneswar (Odisha)	W	Sp	Su	Su	Su	M	M	M	A	A	A	W
Cuttack (Odisha)	W	Sp	Su	Su	Su	M	M	M	A	A	A	W
Berhampur (Odisha)	W	Sp	Su	Su	Su	M	M	M	A	A	A	W
Kolkata (West Bengal)	W	Sp	Su	Su	Su	M	M	M	A	A	A	W
Guwahati (Assam)	W	W	Su	Su	Su	M	M	M	A	A	A	W
Agartala (Tripura)	W	W	Su	Su	Su	M	M	M	A	A	A	W
West region												
Jaipur (Rajasthan)	W	W	Sp	Su	Su	Su	M	M	M	A	A	W
Jamnagar (Gujarat)	W	Sp	Su	Su	Su	M	M	M	M	A	A	W
Mumbai (Maharashtra)	W	Sp	Su	Su	Su	M	M	M	M	A	A	W

Note: The seasons and colour codes used

A Autumn M Monsoon Sp Spring Su Summer W Winter

For peer review only

Supplementary Table 3: The monthly median (and IQR) meteorological data during 2010 and 2017 for regions

Region/ Months	Temperature (°C)	Rainfall (mm)	Wind speed (Kmph)	Humidity (%)	Sunshine (hours)
North region					
January	14 (11, 16)	5.7 (0.5, 13.2)	8.5 (7.1, 9.8)	54 (47, 61)	223 (217.5, 232.5)
February	17 (14, 20)	6.9 (0.5, 22.6)	9.6 (8.1, 10.3)	47 (39, 62)	266 (239, 277.5)
March	23 (21, 25)	4.9 (1.1, 19.9)	9.9 (9, 10.5)	37 (26, 45)	301 (291.5, 308.5)
April	31 (28, 32)	3.9 (1.3, 12.6)	10.5 (8.1, 12.2)	23 (18, 28)	337.5 (315, 345)
May	35 (34, 36)	3.3 (1.1, 14.3)	10.7 (8.9, 12.3)	22 (19, 30)	386 (383, 387.5)
June	35 (31, 37)	22 (8.1, 48.8)	11.1 (9.1, 12.9)	40 (31, 46)	361.5 (346.5, 369.5)
July	31 (26, 33)	72.5 (30, 126.7)	9.6 (8, 12.3)	59 (54, 69)	331.8 (279.3, 367)
August	29 (24, 31)	55.1 (26.3, 93.7)	9 (7, 10.8)	66 (56, 80)	337.8 (298.8, 359.3)
September	28 (25, 30)	31.6 (13.4, 58.1)	8.4 (7.2, 9.2)	56 (49, 76)	286.3 (269.8, 300)
October	26 (25, 27)	1.5 (0.2, 12.2)	6.5 (5.9, 7.2)	42 (34, 50)	250 (244, 262.5)
November	21 (20, 22)	0.1 (0, 3.2)	7.5 (6, 8.2)	33 (31, 39)	225 (222, 225)
December	16 (14, 18)	0.6 (0, 4.4)	7.8 (6.7, 8.8)	39 (35, 45)	231 (225.5, 232.5)
South region					
January	23 (23, 25)	2.8 (1.1, 7.7)	9.7 (8.6, 12.1)	67 (63, 71)	277.5 (274.5, 296.5)
February	25 (24, 26)	1.5 (0.4, 8)	10.1 (8.8, 12.2)	65 (51, 71)	277.5 (272.5, 286)
March	28 (27, 28)	10.1 (3.4, 29.8)	10.9 (8.9, 13.6)	66 (50, 71)	299.5 (294, 308.5)
April	30 (29, 31)	7.8 (2.9, 32.2)	12.1 (9.7, 17.1)	69.5 (52, 73)	317.5 (292.5, 333.5)
May	31 (29, 32)	20.2 (9.1, 30.5)	14.6 (12.9, 17.6)	64 (59, 71)	353.5 (317, 378)
June	28 (27, 31)	50.7 (16.5, 97.8)	18 (17.2, 18.5)	65 (62, 70)	291 (268.5, 319.5)
July	28 (26, 29)	43 (1.7, 78.5)	18.5 (17, 20.1)	68 (63, 74)	283.3 (255.5, 308.5)
August	27 (26, 29)	58.1 (7.9, 92.3)	16.2 (14.4, 17.6)	72 (66, 77)	275.5 (245.5, 296.5)
September	27 (26, 29)	61.7 (34.1, 99.9)	13.8 (12.3, 16.3)	72 (67, 76)	258 (239, 288)
October	27 (26, 28)	61.9 (44.4, 117.6)	10.6 (9.4, 12.2)	71 (68, 75)	246.8 (224.5, 267)
November	25 (24, 27)	34.8 (19.2, 108.9)	9.6 (7.2, 13.5)	72 (69, 75)	205.5 (191, 213)
December	24 (23, 26)	15 (3, 37.9)	10 (8.5, 14.3)	70.5 (66, 74)	225.5 (216, 265.5)
East region					
January	20 (17, 22)	1.7 (0.1, 5.8)	7.2 (6.4, 8.3)	60 (54, 64)	227 (224.5, 231)
February	23 (20, 24)	2.7 (1, 8.7)	8.2 (7, 9)	54 (49, 61)	210 (207, 237)
March	27 (25, 28)	8.5 (2.9, 19.4)	10 (8.9, 11)	56 (46, 60)	291.5 (287.5, 301)
April	31 (29, 32)	12.3 (3.5, 32.1)	12.5 (10.4, 14.6)	60 (54, 66)	355.5 (294.5, 370.5)
May	32 (30, 33)	25.2 (13.9, 54.5)	13.3 (11.2, 15)	66 (60, 69)	365.5 (347, 374.5)
June	32 (29, 33)	79.8 (53.9, 122.3)	12 (10.6, 13.1)	66 (62, 76)	307.5 (292.5, 327)
July	30 (28, 30)	118 (82.6, 175.2)	11.3 (9.8, 12.4)	73 (69.5, 83.5)	276.8 (250, 288.5)
August	29 (28, 30)	100 (70.8, 154.9)	10 (8.9, 11.1)	78 (73.5, 82)	286 (258, 305)
September	28 (28, 29)	89.9 (65.7, 133)	8.3 (7.3, 9.4)	79.5 (75, 83)	252.5 (236.3, 262)
October	26 (25, 27)	44.7 (24.8, 87.9)	6.7 (5.9, 7.6)	77 (71, 82)	287 (271.5, 295)
November	23 (21, 24)	3.6 (0.4, 20.4)	6.4 (5.7, 7.3)	66 (60, 77)	270 (256, 292.5)
December	21 (18, 22)	1 (0.2, 6.7)	7.1 (6.1, 8)	59 (50, 70)	229.5 (222.5, 240)
West region					
January	22 (15, 24)	0.1 (0, 0.9)	10 (8.5, 14.4)	48 (44, 51)	310 (255, 310)
February	24 (20, 25)	0.4 (0, 1)	10.8 (9.9, 14.5)	45 (44, 53)	278.5 (275.5, 281)
March	27 (25, 28)	0.2 (0, 1.2)	10.7 (10.1, 15.9)	47 (29, 53)	307.5 (305.5, 308.5)
April	29 (29, 31)	0.4 (0, 1.7)	11.2 (10, 18.3)	56 (20, 61)	300 (300, 315.5)

For peer review only - http://bmjopen.bmj.com/site/about/guidelines.xhtml

Region/ Months	Temperature (°C)	Rainfall (mm)	Wind speed (Kmph)	Humidity (%)	Sunshine (hours)
May	31 (30, 36)	0.6 (0.1, 5.5)	14.3 (10.7, 23.9)	65 (21, 68)	355 (307, 381.5)
June	31 (29, 36)	48.9 (9.7, 156.7)	16.7 (13.9, 21.7)	68 (34, 75)	311.5 (252, 359.5)
July	29 (28, 32)	121.3 (58.1, 391.4)	17.3 (13.9, 23.8)	77.5 (56.5, 82.5)	194 (158.5, 338.8)
August	28 (27, 29)	118.5 (60, 213.5)	14.1 (11.4, 22.1)	79 (73.5, 84)	196.8 (166.5, 278)
September	28 (27, 29)	70.6 (19.4, 133.8)	11.6 (9.8, 17.8)	74 (62.5, 80)	253.5 (229, 274.8)
October	28 (28, 29)	5.9 (0.5, 26.1)	9 (8.1, 12.5)	51 (41, 67)	296.5 (265, 308.5)
November	27 (23, 28)	0.1 (0, 2.3)	9 (8, 13)	44 (34, 53)	230 (225, 251.5)
December	24 (17, 25)	0 (0, 0.5)	9.8 (8.4, 14.5)	43 (39, 49)	252.5 (232.5, 293.5)

Note: Temperature (°C); Rain: Rainfall (millimetre, mm); Wind Speed (miles/second); Humidity (%); Sunshine (in hours/month)

For peer review only

For peer review only - http://bmjopen.bmj.com/site/about/guidelines.xhtml

Supplementary Table 4: Monthly median (and IQR) meteorological data during 2010 and 2017 at the study sites

Region/ Place (State) and months	Temperature (°C)	Rainfall (mm)	Wind speed (Kmph)	Humidity (%)	Sunshine (hour)
1. North region					
1.1 Srinagar (Jammu and Kashmir)					
January	-3 (-4,-1)	28.4 (16.8,41.9)	5.8 (5.3,6.3)	58 (44,69)	209.5 (205.5,218)
February	0 (-2,2)	69.9 (56.8,89)	5.7 (5.4,6.1)	60 (59,68)	241 (220,261.5)
March	4 (3,5)	63.4 (44.2,107)	5.3 (4.7,6.5)	60 (57,63)	282 (232.5,296)
April	8 (7,9)	45.3 (36.7,84.2)	4.6 (4.4,5)	55 (52,57)	330 (301.5,336)
May	13 (11,14)	31.6 (16.3,57.3)	4.3 (3.7,5.1)	49 (46,50)	387.5 (378.5,387.5)
June	17 (16,18)	15.8 (8.1,33.4)	4.2 (3.8,4.4)	46 (41,47)	372 (364.5,375)
July	19 (18.5,19)	22.6 (13,50.5)	5.4 (4.7,6)	57.5 (57,59)	387.5 (381.5,387.5)
August	18 (18,19)	25 (20.6,34.1)	5 (3.8,5.4)	59.5 (56,65)	371 (368,372.5)
September	15 (14,15)	9 (5.6,48.8)	3.9 (3.6,4.1)	50.5 (48,52.5)	300 (297.8,300)
October	9 (8,11)	10.5 (4.1,15.8)	3.3 (2.8,3.9)	40 (34,41)	262.5 (258.5,262.5)
November	4 (3,5)	8 (1.4,12.1)	4.6 (4.1,6)	33 (31,36)	222 (209.5,225)
December	0 (-1,1)	14.9 (5.3,24.9)	5.8 (5.4,6.7)	31 (27,42)	221.5 (211.5,228)
1.2 Delhi (Delhi) and 1.3. Gurgaon (Haryana)					
January	13 (11,14)	1 (0.3,5.7)	8.5 (7.9,9.2)	54 (53,62)	223 (220.5,228.5)
February	16 (15,18)	5.3 (0.2,7.8)	9.8 (8.9,10.1)	53 (43,63)	269.8 (266,277.5)
March	22 (21,23)	2.9 (1.1,6.5)	9.9 (9.2,10.4)	41 (33,43)	300 (292,303)
April	30 (29,32)	5.3 (2.7,9.8)	10.5 (8.4,10.7)	23 (17,27)	341.8 (331.5,344.5)
May	36 (35,36)	2.4 (0.9,6.7)	10.2 (9.8,10.7)	20.5 (15,22)	386 (383,386)
June	36 (36,38)	20.7 (8.5,30.9)	10.1 (9.8,11.8)	32 (23,36)	363.5 (361.5,369.5)
July	32.5 (32,34)	61.2 (30.7,98.2)	8.9 (8.1,10.2)	53.5 (51,61.5)	345 (331.8,365)
August	31 (30,32.5)	47.8 (28.5,78.9)	8.4 (7.7,9.7)	63.5 (54,72.5)	339.8 (329,349)
September	30.5 (29,31)	27.2 (11.9,61.1)	8.2 (7.7,8.6)	49.5 (47,67)	279.5 (272.3,294)
October	27 (26,28)	0.5 (0,1.1)	6.5 (6.2,7)	34 (31,43)	250 (248.5,250)
November	21 (21,22)	0 (0,0.2)	8.1 (6.5,8.2)	31 (27,40)	225 (223.5,225)
December	16 (15,17)	0.3 (0,1.5)	8.3 (6.9,9)	39 (35,46)	230.5 (225.5,231)
1.4. Lucknow (Uttar Pradesh)					
January	15 (14,16)	8.1 (4.7,9.6)	9.8 (8.6,10.1)	54 (53,58)	224 (219,229.5)
February	18 (17,20)	6.9 (0.5,22.6)	10.1 (9.8,11.3)	52 (42,58)	230.5 (224.5,239)
March	25 (24,25)	3.1 (2.6,10.5)	11.2 (11.1,12)	35 (26,37)	309.5 (303.5,315)
April	32 (32,34)	2 (1.3,3.9)	12.6 (12,13.4)	21 (18,26)	373.5 (363,375)
May	36 (36,37)	5.2 (0.2,6.9)	11.4 (11.2,12.3)	21 (18,25)	384.5 (380,386)
June	37 (34,39)	13.4 (3.2,100.6)	11.6 (11.2,12.9)	32 (25,45)	346.5 (321.5,362.5)
July	32 (31,33)	127.8 (83.6,151.1)	11.1 (10.2,12.3)	63 (57,69)	304.3 (289,321)
August	30 (30,31.5)	76.5 (50.8,95.9)	10.7 (9.4,11.1)	70.5 (58,74.5)	313.8 (304,344.8)
September	29.5 (28.5,30.5)	30.9 (21.1,61.8)	9.1 (8.5,9.6)	64.5 (55,75.5)	316 (301.3,328.3)
October	26 (25,27)	7.4 (0.2,31.3)	6.9 (6.1,7.9)	50 (42,62)	227.5 (225.5,238.5)
November	22 (21,22)	0 (0,0.7)	7.4 (6.9,8.5)	35 (34,48)	225 (218,225)
December	17 (17,18)	0.6 (0,1.6)	8.5 (7.6,9.9)	41 (38,46)	232.5 (231,232.5)
1.5 Indore (Madhya Pradesh)					
January	18 (17,19)	0.8 (0.4,9.1)	9.2 (8,10.5)	44 (32,47)	281 (277.5,282.5)
February	22 (21,23)	1 (0.2,11.1)	9.6 (8.6,11)	32 (29,39)	280 (277,290)
March	27 (27,27)	2.9 (0,6.4)	10 (9.8,10.4)	21 (18,26)	307 (301,310)
April	32 (32,33)	0.6 (0.2,2.8)	12.3 (11.2,14.4)	18 (17,21)	315 (310,317.5)
May	35 (34,35)	1.9 (0.6,2.7)	15.3 (14.7,16.8)	24 (22,30)	386 (384.5,387.5)
June	31 (31,32)	48.9 (29.6,72.2)	15.4 (14.6,16.9)	51 (47,57)	341 (294,352)

For peer review only - http://bmjopen.bmj.com/site/about/guidelines.xhtml

Region/ Place (State) and months	Temperature (°C)	Rainfall (mm)	Wind speed (Kmph)	Humidity (%)	Sunshine (hour)
July	27 (26,27)	124.5 (83.8,186.7)	15.5 (12.6,17.3)	80 (75,81.5)	201.5 (178.8,239)
August	25.5 (24.5,26)	101.7 (78.8,132.8)	12.1 (11.4,14.9)	88.5 (80,90)	166.5 (144.8,208.3)
September	25.5 (25,26)	52.2 (41,62.1)	9.7 (8.6,10.5)	77 (75,81)	253.5 (241.5,267.3)
October	26 (25,26)	2.3 (0.3,31.1)	7.2 (6.6,8.3)	45 (40,58)	271 (254.5,272.5)
November	23 (22,24)	0.3 (0,2.9)	7.6 (6.4,8)	37 (33,39)	225 (220.5,225)
December	19 (19,20)	0.1 (0,1.2)	8.1 (7.4,8.8)	36 (34,40)	232.5 (229.5,232.5)
2. East region					
2.1 Bhubaneswar and 2.2. Cuttack (Odisha)					
January	22 (21,22)	1.5 (0.1,5.6)	7.2 (6.4,7.4)	56.5 (56,60)	224.5 (221.5,228)
February	24.5 (24,25)	2 (0.5,8.9)	8.2 (7.1,9.3)	56 (53,62)	209.3 (202.5,210)
March	28 (28,29)	6.7 (3.6,16.6)	10.9 (9.4,11.5)	57.5 (54,62)	287.3 (275.5,290)
April	31 (31,32)	5.4 (1.6,12.3)	15 (12.4,17.2)	60.5 (57,66)	366.8 (360,372)
May	32 (32,33)	22.3 (15.4,26.9)	14.9 (13.9,15.8)	64.5 (63,67)	366.8 (362.5,375.5)
June	32 (32,33)	80 (60.3,114.7)	12.2 (12,13.7)	62.5 (62,66)	300.8 (292.5,322.5)
July	30 (30,30)	118 (80.3,137.4)	11.3 (10.1,12.1)	72 (69,73)	269.8 (253.3,283.8)
August	29 (29,30)	92.6 (67,133.3)	10.4 (9.2,11.4)	74.5 (71.5,77.5)	285 (262.3,296)
September	29 (28,29)	89.9 (67.3,112.9)	8.7 (7.3,9.8)	77 (74.5,79.5)	248.3 (234.3,257.3)
October	27 (26,27)	50.1 (25.7,89.9)	7.4 (6.7,7.9)	74 (69,82)	285.5 (263,290)
November	23.5 (23,25)	9.1 (1,28.3)	6.6 (6.2,7.2)	64.5 (60,66)	261.8 (256,268.5)
December	21 (21,22)	4.7 (0.3,11.8)	6.9 (6.6,8)	57 (53,59)	223.8 (207,229.5)
2.3 Berhampur (Odisha)					
January	22 (21,22)	1.6 (0.6,5.6)	6.9 (6.6,7.6)	66 (63,69)	224.5 (221.5,228)
February	24 (24,24)	3.6 (2.1,15.7)	8.2 (7.2,8.7)	62 (61,68)	210 (205.5,210)
March	28 (27,28)	8.5 (1.3,20.6)	10.8 (9.8,11.7)	61 (59,64)	288.5 (275.5,291)
April	30 (30,31)	9.7 (2,20.6)	13.9 (12.4,14.6)	63 (58,67)	367.5 (361.5,372)
May	32 (32,32)	13.3 (7.1,28.9)	13.3 (12.5,13.7)	67 (64,68)	368 (364,380)
June	31 (31,32)	60.7 (48.2,79.8)	11.3 (11.1,11.8)	67 (64,69)	307.5 (300,324.5)
July	29 (29,30)	82.9 (65.6,107.6)	11.1 (10.5,11.4)	73 (71,74.5)	283.5 (279.3,289)
August	29 (29,29)	93.5 (72.6,119.7)	9.4 (9.1,10)	74.5 (73,78)	294.3 (272,302.5)
September	28.5 (28,29)	91.1 (67,116)	8.1 (7.5,8.8)	76 (75,77.5)	254.8 (250.3,261.5)
October	27 (26,27)	44.9 (33.2,132.7)	8.1 (6.6,9)	72 (70,80)	289.5 (263,295)
November	24 (24,24)	44.5 (0.4,59.7)	7 (6.6,8.3)	67 (63,71)	262 (256,271.5)
December	22 (22,22)	3.3 (1,30.7)	7.8 (7,8.2)	62 (60,67)	225 (207,229.5)
2.4. Kolkata (West Bengal)					
January	20 (19,21)	0.1 (0,7.7)	9.1 (8.6,9.9)	44 (40,52)	228 (225,231)
February	23 (23,24)	4.4 (0.8,7.8)	9 (8.1,9.3)	45 (40,46)	211 (204.5,212.5)
March	28 (27,28)	8.7 (1.7,19.2)	10 (9.4,10.2)	50 (46,57)	304 (295,308.5)
April	31 (31,33)	12.5 (5.2,37.2)	14.2 (10.3,15.2)	58 (54,63)	300 (287.5,312)
May	33 (32,33)	16.2 (11.2,28.5)	15 (13.7,17.7)	61 (59,67)	371 (369.5,377.5)
June	32 (31,33)	63.7 (52.7,92.7)	13.1 (12.8,15.8)	65 (64,72)	318.5 (290.5,327)
July	30 (29.5,30)	119.7 (102.1,171.9)	12.6 (11.5,13.2)	76 (74,78)	258.8 (243.3,271.3)
August	29 (29,30)	102.7 (86,153.2)	11 (10.3,11.1)	80.5 (76,82)	277.3 (247.8,284.5)
September	28 (28,29.5)	81.4 (74.9,111.4)	9.1 (8.1,10.3)	81.5 (77,85)	236.3 (226.8,241.3)
October	27 (26,27)	27.2 (23.6,34.9)	7.3 (6.5,8.7)	72 (71,80)	280 (265.5,287.5)
November	24 (23,25)	3.3 (0.3,16.7)	7.5 (6.1,9.3)	57 (53,65)	292.5 (284,297)
December	21 (20,22)	0.1 (0,1.8)	8.7 (8.5,9.5)	47 (41,50)	230 (218,232.5)
2.5. Patna (Bihar)					
January	16 (15,17)	5.8 (0.8,8.3)	9.5 (9.4,10)	54 (51,63)	229.5 (222.5,232.5)
February	19 (18,21)	0.9 (0.4,2.7)	10.4 (9.7,11.8)	49 (47,54)	207.5 (202.5,213)
March	26 (25,26)	2.9 (1.6,5.5)	11.1 (10.1,11.7)	32 (30,33)	303.5 (291.5,305)

For peer review only - http://bmjopen.bmj.com/site/about/guidelines.xhtml

Region/ Place (State) and months	Temperature (°C)	Rainfall (mm)	Wind speed (Kmph)	Humidity (%)	Sunshine (hour)
April	33 (32,33)	6.5 (2.4,22.2)	12.3 (11.4,14.2)	28 (20,31)	373.5 (358.5,375)
May	35 (35,37)	23.2 (5,41.1)	13 (12.3,13.6)	35 (29,39)	377 (366.5,386)
June	36 (34,37)	47.6 (21.1,58.6)	11.9 (11.5,13.6)	42 (42,51)	337 (327,355)
July	32 (31,32.5)	150.4 (120.4,160.4)	12 (11.7,14)	65.5 (60.5,68.5)	283 (257.5,312.5)
August	31 (31,32)	63.4 (38.5,138.2)	10.6 (9.3,11.7)	65 (61.5,71.5)	318.5 (293,329.8)
September	29 (28.5,30.5)	66.7 (32.4,116.6)	8.7 (8.4,10.4)	67 (60.5,80)	273 (249.5,282.5)
October	26 (25,27)	29.1 (8.1,54.9)	6.8 (5.9,7.5)	63 (51,78)	297 (271.5,304)
November	22 (21,23)	0 (0,0.3)	6.8 (6.3,8.1)	45 (39,61)	232.5 (231,235)
December	18 (18,19)	0 (0,0.7)	8 (7.5,9.1)	49 (38,54)	231 (225,232.5)
2.6. Guwahati (Assam)					
January	17 (15,17)	3.9 (0.2,10.3)	3.9 (3.8,4.3)	67 (64,76)	228 (225,231)
February	20 (19,20)	4.9 (2.3,10.2)	4.2 (4,4.5)	56 (54,62)	245 (242,253.5)
March	24 (23,24)	14.1 (1.4,22.9)	4.9 (4.6,4.9)	46 (42,58)	299.5 (295,310)
April	26 (25,27)	39.7 (15.1,80.8)	4.5 (4.4,4.8)	62 (50,68)	292.5 (280.5,300)
May	27 (27,28)	83.1 (54.5,122.4)	4.2 (3.8,5)	69 (63,73)	344 (336.5,357)
June	28 (27,29)	236.2 (191.4,326.2)	3.8 (3.6,4.3)	81 (76,84)	323.5 (292.5,334)
July	27.5 (27,28)	344.4 (224.2,407.4)	3.8 (3.5,4.1)	87 (83.5,88.5)	317.8 (274.5,332.8)
August	28 (27.5,28)	235.2 (214.1,276.4)	3.8 (3.7,4.2)	84 (82,85)	297.3 (283,310.5)
September	27 (26.5,27)	159.3 (143.5,217.6)	3.4 (3.1,4)	84 (81.5,85)	272 (257.8,274.8)
October	24 (24,24)	48.8 (12.8,72.7)	3.6 (3.4,4.2)	79 (77,81)	294 (283,305.5)
November	20 (20,20)	4.5 (1,7.3)	3.8 (3.7,4.5)	78 (71,80)	294 (292,297)
December	17 (17,18)	2.5 (1.1,9)	4 (3.8,4.2)	74 (73,77)	250.5 (240,253.5)
2.7. Agartala (Tripura)					
January	18 (17,19)	0.3 (0.1,2.2)	6.7 (6.5,7.2)	61 (58,69)	231 (228,232.5)
February	21 (20,22)	5.6 (1.7,10.5)	7.2 (7,8.6)	53 (51,57)	246 (237,250.5)
March	25 (24,26)	22.6 (11.8,53.2)	8.9 (8.8,9.5)	57 (51,64)	295.5 (288,301)
April	28 (28,29)	113.4 (38.1,141.2)	11.1 (8.5,12.5)	67 (64,73)	266.5 (246,297)
May	29 (28,30)	113.8 (73.4,194.5)	9.7 (9.1,11.5)	74 (74,80)	307.5 (299.5,320)
June	29 (28,29)	99.2 (72.8,110.5)	11.1 (10.6,13.4)	82 (79,85)	286.5 (282.5,303.5)
July	28 (28,28)	73 (50.4,103.5)	12.1 (10.4,13.4)	84.5 (83.5,85.5)	261.8 (244.8,276)
August	28 (27.5,28)	78.4 (61.2,101.3)	10.6 (9.4,11.1)	86.5 (82,87.5)	249.3 (231.8,271)
September	27 (27,28)	57.1 (43.3,144.7)	8.4 (7.5,9.4)	86.5 (82,89)	249 (225,259)
October	25 (25,26)	55.3 (23,121.8)	5.9 (4.9,6)	85 (82,87)	284.5 (271,293.5)
November	21 (21,22)	5.2 (0.2,31)	5.5 (5.3,6.1)	78 (77,79)	300 (282,300)
December	18 (18,20)	0.7 (0.2,6.7)	6.1 (5.1,6.2)	71 (70,73)	264.5 (258.5,267.5)
3. South region					
3.1. Vishakhapatnam (Andhra Pradesh)					
January	23 (23,24)	3.5 (1.3,6.1)	10.6 (9.4,11.1)	68 (67,72)	277.5 (274.5,277.5)
February	25 (24,25)	1.4 (0.9,4.2)	10.1 (9.4,10.8)	68 (65,70)	280 (277.5,288.5)
March	27 (27,28)	3.4 (3.3,16.2)	13.6 (11.5,14)	70 (65,70)	308.5 (298,310)
April	29 (29,30)	5 (1.3,12.6)	17.4 (16.7,19.1)	71 (70,74)	330 (320,335)
May	31 (31,31)	8.6 (3.5,18)	17 (15.9,18.5)	71 (70,72)	378 (371,381.5)
June	31 (31,31)	91 (40.8,129.1)	16.9 (14.3,17.3)	70 (66,71)	274.5 (268.5,306)
July	29.5 (29,30)	77.6 (60.1,105.1)	18.5 (16.2,20.1)	73 (71.5,74.5)	252.5 (226.8,289.8)
August	29 (29,30)	111.7 (96.7,120.4)	13.8 (13.5,16.6)	74.5 (73,77)	241 (234.5,263.5)
September	29 (29,29)	127 (104.7,164.2)	11.8 (11.7,13)	76 (75.5,77)	234.5 (226.3,243.8)
October	27 (27,28)	90.4 (50.2,182.7)	11.7 (9.1,13.2)	72 (69,77)	222 (205.5,236.5)
November	25 (25,26)	46.2 (19.2,144.8)	12.1 (11.5,15.1)	70 (66,71)	207 (202,217.5)
December	24 (24,24)	4.2 (2.6,14.6)	13.8 (12,14.4)	67 (66,70)	225.5 (222,228)

For peer review only - http://bmjopen.bmj.com/site/about/guidelines.xhtml

Region/ Place (State) and months	Temperature (°C)	Rainfall (mm)	Wind speed (Kmph)	Humidity (%)	Sunshine (hour)
3.2. Hyderabad (Telangana)					
January	22 (21,23)	0.5 (0.1,1.5)	12.1 (10.1,12.6)	47 (46,51)	277.5 (274.5,277.5)
February	24 (24,25)	0 (0,1.6)	12.4 (10.6,12.9)	41 (38,45)	280 (277.5,288.5)
March	28 (28,29)	3.4 (0.1,10.6)	11.1 (10.3,11.9)	34 (32,39)	308.5 (298,310)
April	32 (31,33)	2.9 (1.5,5.3)	9.7 (9.2,11.9)	33 (27,35)	330 (320,335)
May	34 (33,35)	9.4 (7.3,17.6)	12.9 (12,14.4)	30 (28,34)	378 (371,381.5)
June	29 (28,29)	111.6 (33.3,131.2)	18.7 (18.1,20.7)	57 (49,63)	274.5 (268.5,306)
July	26 (26,27.5)	66.4 (47.1,81)	20.7 (18.4,23)	72.5 (65,75)	252.5 (226.8,289.8)
August	26 (25,26.5)	68.6 (36,77.7)	16.6 (15.3,17.5)	76 (70,78)	241 (234.5,263.5)
September	26 (25,26)	51.9 (40.3,74.1)	12.8 (11.3,13.9)	74.5 (70.5,78.5)	234.5 (226.3,243.8)
October	24 (24,25)	35.9 (14.9,45.2)	10.1 (9.2,10.6)	65 (59,70)	222 (205.5,236.5)
November	22 (21,23)	3.1 (0.5,18.8)	10.2 (9.5,10.8)	60 (51,62)	207 (202,217.5)
December	21 (20,22)	0.3 (0.2,1.1)	10.3 (10.1,11.3)	50 (49,53)	225.5 (222,228)
3.3. Coimbatore (Tamil Nadu)					
January	23 (23,23)	2.2 (1.1,26.6)	7.4 (6.9,8.6)	63 (63,66)	290 (285,305.5)
February	25 (24,25)	3 (0.9,8.3)	7.8 (7.4,8.9)	55 (51,56)	277 (272,286)
March	28 (27,28)	26.7 (10.1,60.5)	7.1 (7.1,8.6)	53 (50,55)	299.5 (298,302.5)
April	30.5 (30,31)	8.9 (6.7,28.2)	9.2 (8.3,11.6)	52 (52,54)	297.5 (284.5,297.5)
May	30 (29,30)	29.7 (29.7,30.5)	12.4 (11.9,13.2)	59 (59,61)	334 (317,335)
June	27 (27,27)	4.9 (4.9,6.7)	18.1 (17.7,18.3)	64 (64,66)	301.5 (285.5,302)
July	27 (27,28)	0.2 (0,1.7)	18.5 (17.9,19.3)	63 (61,64)	291 (268.5,308.5)
August	27 (26,27)	3.1 (0.9,7.9)	16.1 (14.8,17.6)	65 (63,67)	291.5 (274.5,296.5)
September	27 (27,27)	15.6 (8.3,42.8)	16.3 (14.5,17.6)	65.5 (65,67)	278 (256.5,282)
October	26 (26,26)	53.2 (53.2,63.8)	9.7 (8.3,10.6)	68 (68,71)	268.3 (254.5,270)
November	24 (24,24)	32.6 (32.6,34.9)	6.1 (6.1,7)	72 (69,78)	205.5 (194,206.5)
December	23 (23,23)	14.3 (6,15.5)	7 (6.7,7.9)	71 (67,74)	242.3 (225.5,267)
3.4. Chennai (Tamil Nadu)					
January	25 (25,25)	7.7 (4.4,10.5)	15.4 (12.4,16.6)	73 (70,75)	267 (259.5,273.5)
February	25 (25,26)	1.5 (0.3,26)	13.2 (11.7,14)	72 (70,74)	275.5 (272.5,287)
March	28 (27,28)	1.8 (1.1,10.1)	14.8 (13.6,15.5)	72 (71,73)	301 (294,304)
April	30 (30,30)	7.2 (0.3,35.3)	18.9 (16.9,20.1)	72 (71,74)	344.5 (330,352.5)
May	32 (32,32)	18.6 (8.3,37.5)	18.9 (18.2,19.1)	66 (66,67)	381.5 (355,383)
June	31 (31,32)	37.8 (19,63.2)	17.4 (16.9,18.4)	62 (61,66)	336.5 (327.5,348.5)
July	30.5 (30,31)	37.9 (22.1,61.8)	16.8 (15.7,17.5)	65 (63,65.5)	343.5 (339,354.3)
August	30 (29,30)	75.4 (61.7,88.7)	15.2 (14.1,16)	69 (66.5,70.5)	339.3 (335.8,359.8)
September	29.5 (29,30)	101.1 (91.5,105.6)	13.3 (13,13.8)	72 (70,72)	333.5 (317,347.8)
October	29 (28,29)	102.6 (81.5,154.2)	12.2 (10.9,13.8)	73 (71,74)	252 (247,263.5)
November	27 (27,27)	60 (48.4,344.4)	17.3 (15.3,17.7)	74 (72,79)	201 (190.5,213)
December	26 (25,26)	84.8 (58.6,111.3)	18 (16.5,20)	76 (72,77)	199 (188.5,208)
3.5. Trivandrum (Kerala)					
January	26 (26,26)	3.2 (1.5,7.7)	8.7 (8.2,9.5)	70 (68,71)	299.5 (296.5,301)
February	26 (26,26)	6.1 (1.4,23)	8.8 (8.7,10.7)	71 (65,72)	278.5 (269.5,284)
March	28 (27,28)	37.8 (14.8,53.6)	9.8 (9.1,10.7)	71 (70,75)	293.5 (288,299.5)
April	28 (28,29)	57.3 (32.2,115.9)	12.2 (10.7,14.3)	79 (72,79)	278 (264,285)
May	28 (28,29)	24.9 (17.4,42.5)	15 (14.6,18)	79 (72,79)	252 (245.5,267.5)
June	27 (27,28)	82.2 (46.9,110.2)	20.1 (17.4,21.5)	78 (74,79)	243 (213,256)
July	26.5 (26,27.5)	65.6 (15.1,101.1)	21.1 (18.7,22.1)	78 (75,80)	266.3 (248.3,278.8)
August	26 (26,27.5)	33.6 (7.8,72.9)	18.8 (17,20.5)	80 (75.5,81)	264.5 (240.8,285.5)
September	26.5 (26,27)	61.7 (40.3,77.2)	17 (13.8,17.4)	80 (77,81.5)	256 (244,263.3)
October	27 (26,27)	117.9 (32.3,220.7)	11.4 (10.1,14.9)	78 (75,80)	266 (245.5,286)

For peer review only - http://bmjopen.bmj.com/site/about/guidelines.xhtml

Region/ Place (State) and months	Temperature (°C)	Rainfall (mm)	Wind speed (Kmph)	Humidity (%)	Sunshine (hour)
November	27 (27,27)	107.7 (47,140)	8.6 (8,9.1)	74 (72,76)	189 (174,205)
December	26 (26,27)	25.1 (15.7,37)	8.9 (8.6,9.6)	72 (72,75)	272.5 (267,280.5)
4. West region					
4.1. Mumbai (Maharashtra)					
January	24 (24,25)	0 (0,0.4)	10 (9.7,10.9)	52 (51,53)	310 (310,310)
February	25 (25,26)	0.4 (0,2.9)	10 (9.2,10.2)	54 (51,55)	280 (275.5,281)
March	28 (27,28)	0.6 (0,1.3)	10.5 (9.6,10.7)	53 (51,56)	308.5 (305.5,310)
April	29 (29,29)	0.1 (0,0.2)	9.7 (9.4,10.3)	63 (61,66)	300 (292.5,300)
May	30 (29,30)	1.3 (0.6,8)	10.5 (10.1,10.7)	72 (68,73)	305.5 (292,307)
June	29 (29,29)	292.8 (156.7,370)	16 (14.6,16.7)	78 (75,78)	217 (190,252)
July	27 (27,28)	450.2 (391.4,540.5)	17.3 (16,18.7)	83.5 (82,85.5)	164.8 (147.5,193.3)
August	27 (27,27)	282.7 (213.5,376.4)	14.1 (12.7,15.5)	85.5 (84,86.5)	190.8 (186.5,212.8)
September	27 (27,27)	136.2 (103.3,196.1)	9.8 (9.5,11.7)	82 (80,83)	229.8 (225.5,245)
October	28 (27,29)	29.9 (25.5,48.4)	8.1 (7.7,8.8)	67 (67,72)	299.5 (286.5,304)
November	27 (27,28)	2.3 (0,9.9)	9 (8.6,9)	55 (53,58)	257.5 (251.5,258.5)
December	26 (25,26)	0.2 (0,3.4)	9.8 (9,10)	51 (49,55)	295 (293.5,297.5)
4.2. Jamnagar (Gujarat)					
January	22 (21,23)	0 (0,0.1)	15.8 (14.4,17)	44 (41,49)	310 (307,310)
February	24 (23,24)	0 (0,0.6)	16 (14.5,16.8)	45 (44,50)	278.5 (274.5,281)
March	27 (27,28)	0.1 (0,0.2)	16.5 (15.9,17.1)	47 (44,53)	308.5 (306,308.5)
April	29 (29,30)	0 (0,0.4)	20.8 (18.3,21.3)	56 (54,56)	300 (297,300)
May	31 (30,31)	0.1 (0.1,0.2)	24.8 (23.9,25.3)	65 (63,67)	355 (353.5,357.5)
June	31 (31,32)	35.8 (5,93.6)	23.9 (21.7,27.9)	68 (67,69)	311.5 (296.5,321)
July	29 (29,29)	119.4 (88.5,141.7)	25.4 (23.8,27.9)	77.5 (75,78)	167.8 (143.5,197.5)
August	28 (28,28.5)	61.6 (39.7,107.3)	23.2 (22.1,24.6)	79 (77,80)	156.5 (143.5,182)
September	28 (28,29)	70.6 (24.3,113.3)	18.2 (17.8,19.7)	74 (73.5,77)	244.8 (225.3,259)
October	30 (29,31)	0.8 (0.2,12.4)	14 (12.5,14.1)	51 (48,62)	310 (308,310)
November	28 (27,29)	0.1 (0,1.9)	13.9 (13,14.6)	43 (40,50)	230 (228.5,231)
December	24 (24,25)	0 (0,0)	16.2 (14.5,17.1)	43 (39,46)	252.5 (250,252.5)
4.3. Jaipur (Rajasthan)					
January	14 (14,15)	1.9 (0.3,7.9)	8.3 (8.2,8.5)	47 (42,50)	253.5 (245.5,255)
February	18 (18,20)	1 (0.3,3.5)	10.2 (8.9,10.9)	34 (31,44)	277.5 (275.5,288.5)
March	25 (24,25)	0.8 (0,4.1)	10.2 (10,11)	24 (24,29)	304 (301.5,308.5)
April	31 (31,32)	3 (0.8,4.6)	11.2 (10.5,11.7)	19 (16,20)	323 (315.5,327.5)
May	36 (36,36)	5.5 (0.4,6.9)	14.3 (13.1,16.5)	19 (15,21)	383.5 (381.5,385)
June	36 (36,37)	9.9 (3.5,20.5)	13.9 (12.4,17.4)	30 (28,34)	363 (359.5,367.5)
July	32 (32,33.5)	48.4 (33.6,69)	12.8 (10.6,13.9)	55.5 (51,56.5)	351.8 (338.8,369.5)
August	29.5 (29,31)	74.1 (59.6,138.3)	11.1 (10.5,12.7)	66.5 (58.5,73.5)	287.5 (278,302.5)
September	29.5 (28.5,30)	19.4 (14.2,31)	10.4 (9.4,11.5)	53.5 (46.5,62.5)	281.8 (274.8,290.5)
October	28 (27,28)	2.3 (0,4.7)	8.4 (7.4,9.4)	31 (27,41)	263.5 (262.5,265)
November	22 (21,23)	0 (0,0.8)	7.2 (6.7,8)	29 (26,34)	225 (223.5,225)
December	16 (16,17)	0.4 (0,0.7)	7.6 (6.3,8.4)	37 (33,39)	232.5 (229.5,232.5)

Note: IQR- Interquartile range

For peer review only - http://bmjopen.bmj.com/site/about/guidelines.xhtml

Supplementary Figure 2: The pattern of monthly meteorological parameters across the regions

SF2a: Monthly median temperature for different regions in India (^{0}C)

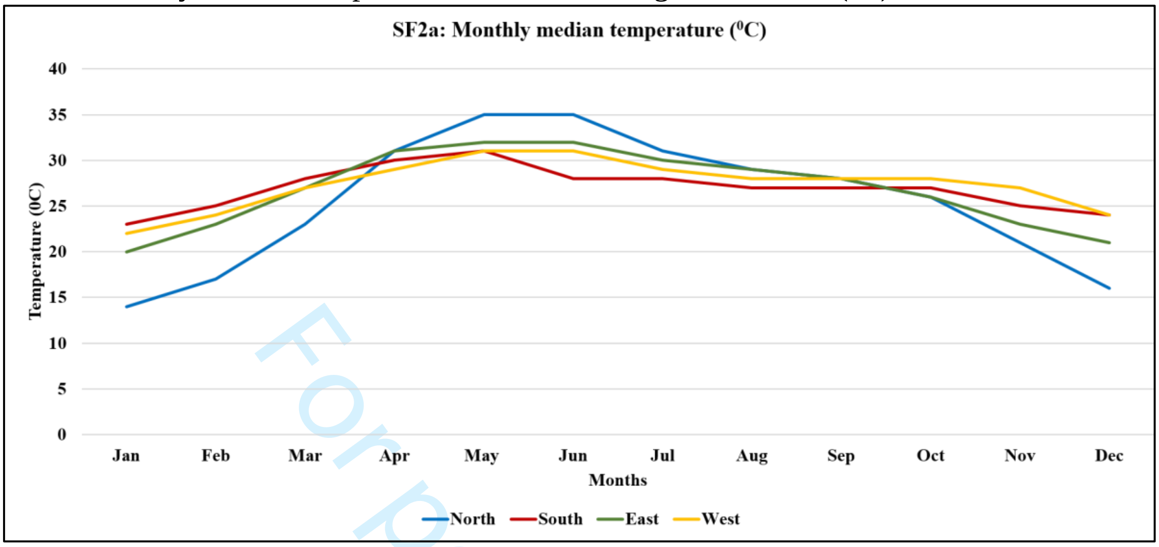

SF2b: Monthly median rainfall (mm) for different regions in India

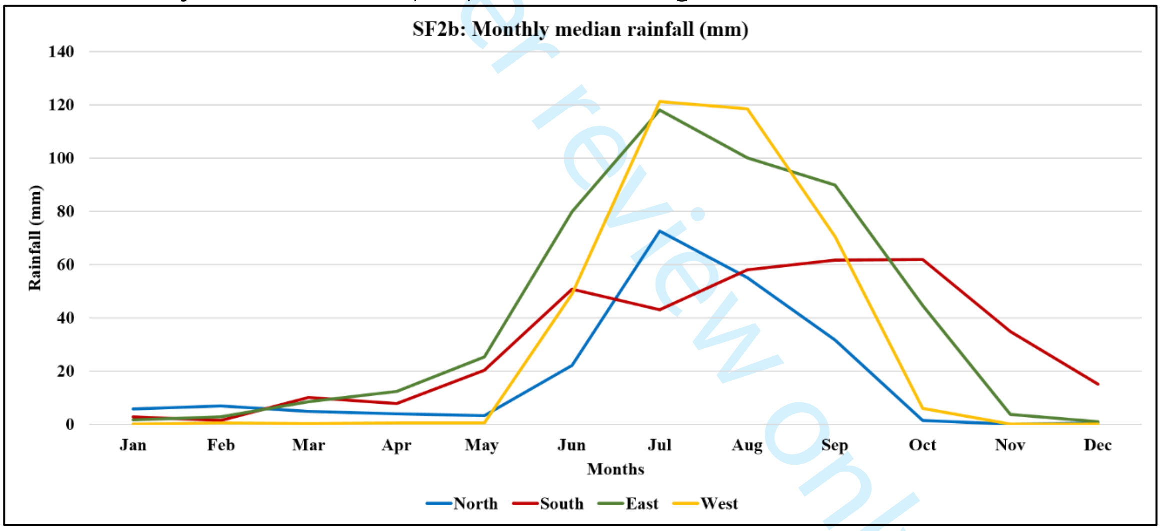

For peer review only

SF2c: Monthly median wind speed (Kmph) for different regions in India

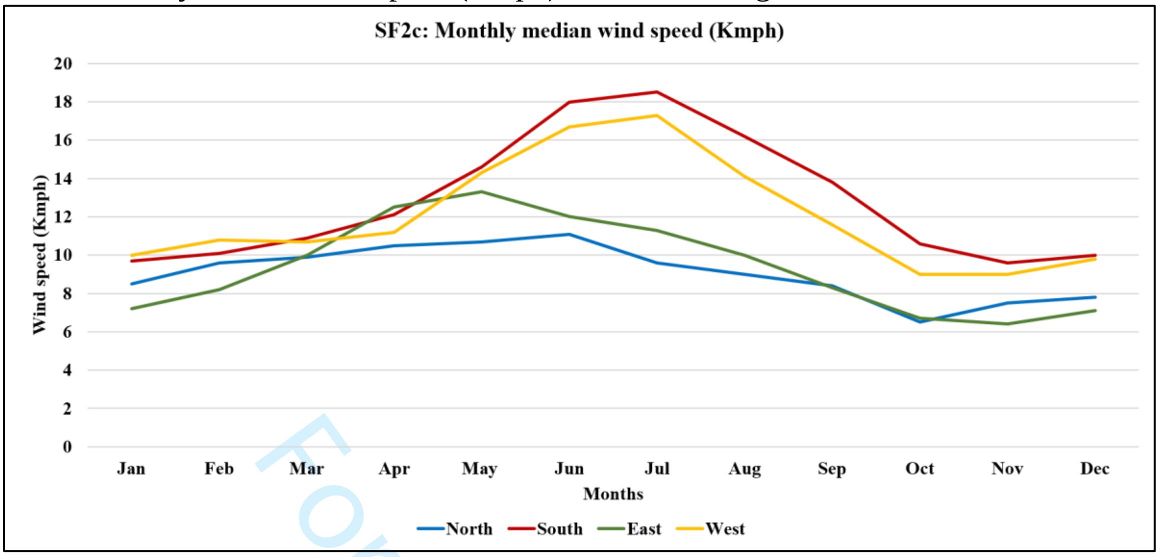

SF2d: Monthly median humidity (%) for different regions in India

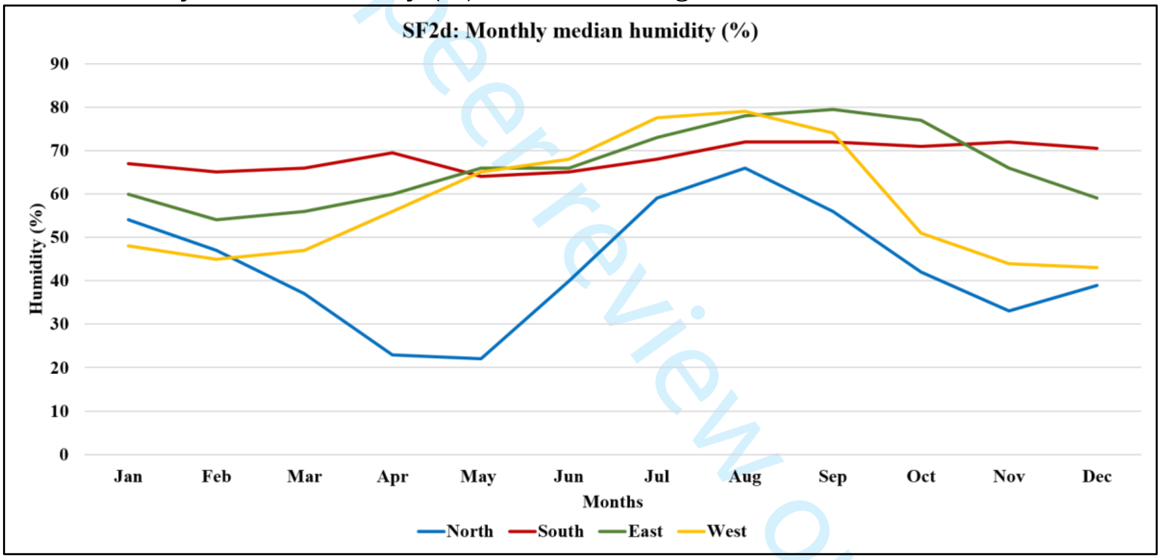

SF2e: Monthly median sunshine (hours) for different regions in India

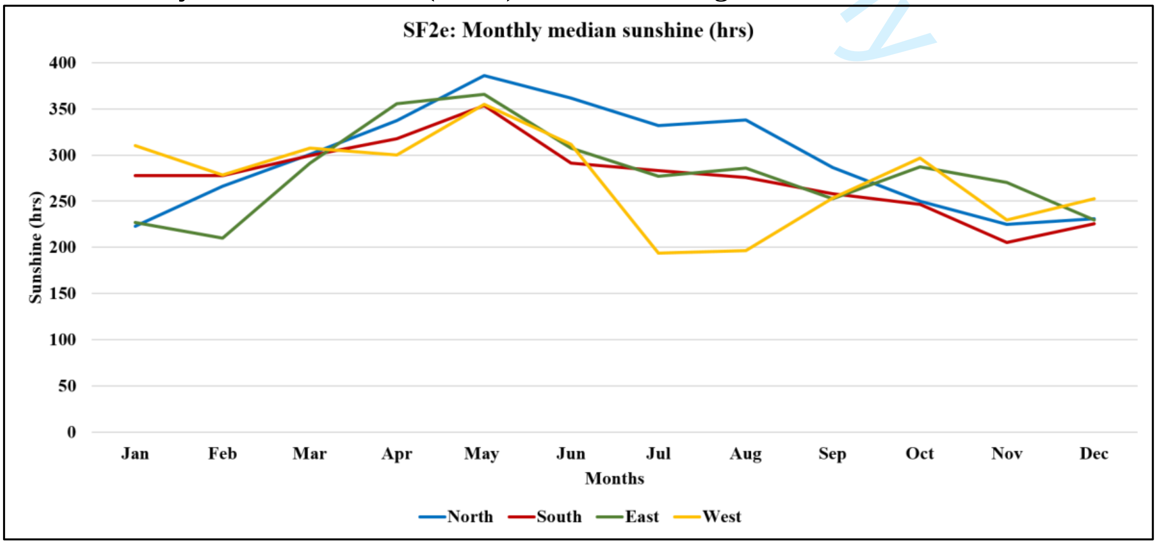

For peer review only - http://bmjopen.bmj.com/site/about/guidelines.xhtml

Supplementary Figure 3: The pooled monthly (SF3a) and seasonal (SF3b) distribution of intussusception in children during July 2010 to September 2017

SF3a: The monthly trend of intussusception cases

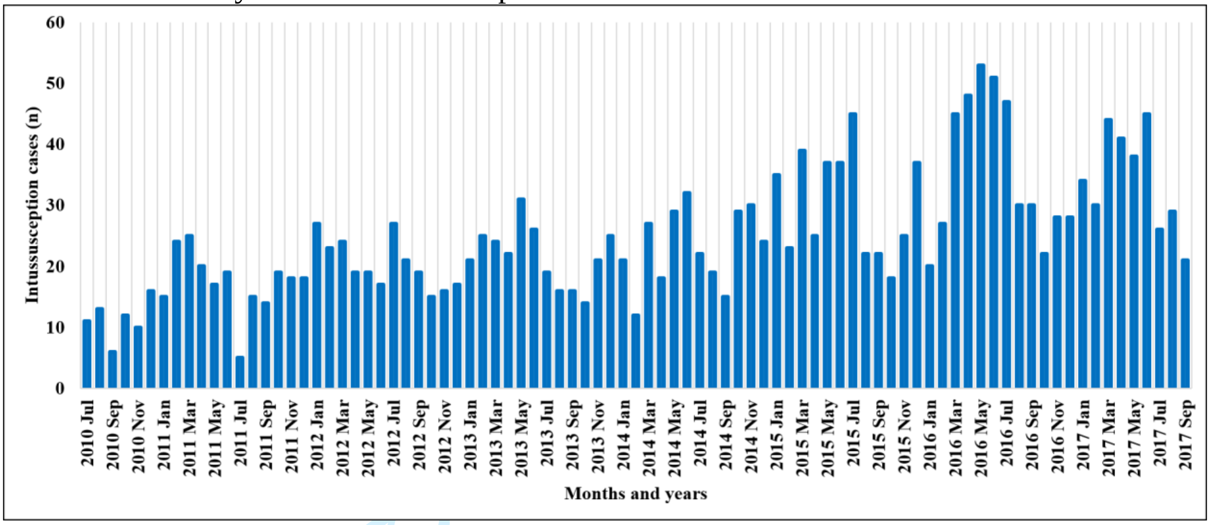

SF3b: The seasonal trend of intussusception cases

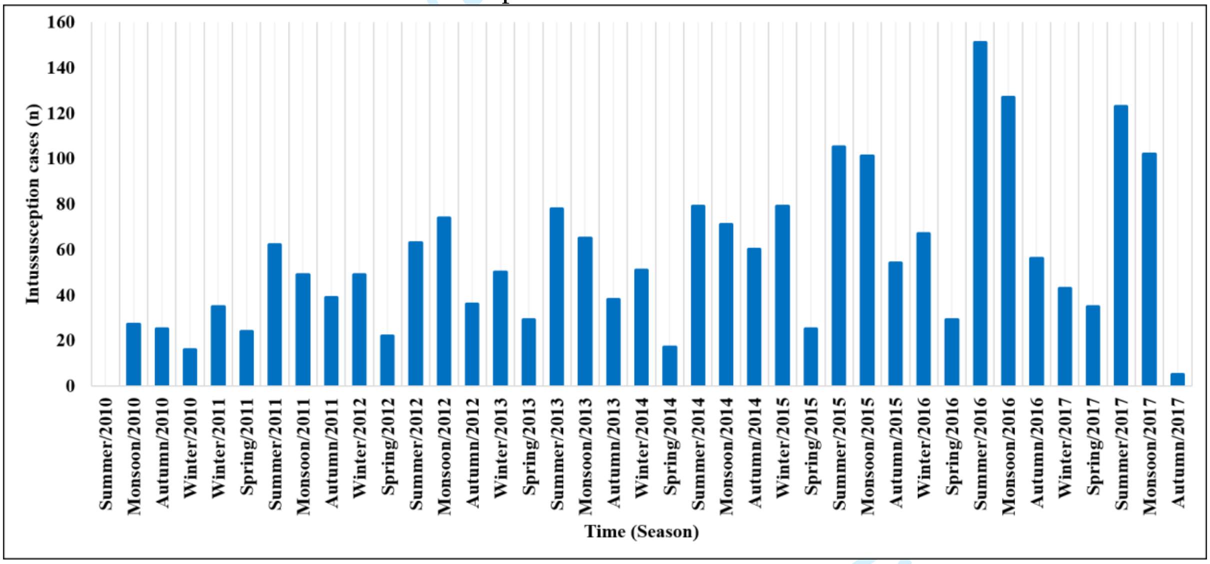

For peer review only - http://bmjopen.bmj.com/site/about/guidelines.xhtml

Supplementary Table 5. Pearson correlation analysis between monthly intussusception cases and monthly median meteorological parameters for the study sites

Regions/Study sites (n, number of cases)	Pearson correlation coefficient (r) for meteorological parameters				
	Temperature (°C)	Rainfall (mm)	Wind Speed (kmph)	Humidity (%)	Sunshine (hour)
North region					
Srinagar (n=145)	.253*	.164	.183	.012	.239*
Delhi and Gurgaon (n=96)	.107	-.045	.183*	-.173*	.134
Lucknow (110)	.091	.003	.285**	-.106	.192
Indore (n=19)	.101	-.211*	.290**	-.309**	.188
East region					
Bhubaneswar/Cuttack (n=335)	.047	-.056	.125	-.167*	.035
Berhampur (n=30)	.050	.047	.035	.044	.036
Kolkata (n=80)	.220*	-.052	.291**	-.144	.253*
Patna (n=19)	-.124	-.189	-.038	-.216*	-.040
Guwahati (n=112)	.220*	.001	.317**	-.119	.194
Agartala (n=17)	.082	.080	.139	-.017	.016
South region					
Vishakhapatnam (n=77)	.088	.053	-.002	.022	.029
Hyderabad (n=2)	-.030	-.061	-.131	.022	-.096
Coimbatore (n=136)	-.015	-.075	-.155	-.028	.017
Chennai (n=92)	.052	-.017	.007	.015	-.016
Trivandrum (n=774)	.085	-.286**	-.011	-.323**	.229*
West region					
Mumbai (n=34)	-.066	-.081	-.027	-.103	.035
Jamnagar (n=54)	.110	-.017	.011	.026	.105
Jaipur (n=29)	.187	-.078	.148	.001	.122

Note: Pearson correlation coefficient (r); Significance two tailed
** Significance level- p value <0.05; ** Significance level- p value <0.01*

For peer review only

Supplementary Table 6. Spearman's Rank correlation analysis between monthly intussusception cases and monthly median meteorological parameters for the study sites

Regions/Study sites (n, number of cases)	Pearson correlation coefficient (r) for meteorological parameters				
	Temperature (°C)	Rainfall (mm)	Wind Speed (kmph)	Humidity (%)	Sunshine (hour)
North region					
Srinagar (n=145)	.208	.122	.146	.032	.169
Delhi and Gurgaon (n=96)	.067	-.101	.136	-.184*	.065
Lucknow (110)	.166	.064	.331**	-.101	.225*
Indore (n=19)	.095	-.148	.286**	-.301**	.138
East region					
Bhubaneswar/Cuttack (n=335)	.036	-.068	.117	-.143	-.009
Berhampur (n=30)	.058	.037	.044	.035	.021
Kolkata (n=80)	.097	-.084	.170	-.236*	.125
Patna (n=19)	-.146	-.264*	-.085	-.231*	-.040
Guwahati (n=112)	.240*	-.014	.290**	-.118	.165
Agartala (n=17)	.107	.107	.154	.021	.044
South region					
Vishakhapatnam (n=77)	.106	.081	.048	.017	.060
Hyderabad (n=2)	-.012	-.052	-.138	.040	-.043
Coimbatore (n=136)	.002	-.123	-.147	-.091	.070
Chennai (n=92)	.055	.041	.025	.055	-.027
Trivandrum (n=774)	.134	-.287**	-.026	-.353**	.301**
West region					
Mumbai (n=34)	-.025	-.140	-.092	-.120	.046
Jamnagar (n=54)	.056	.071	.019	-.009	.106
Jaipur (n=29)	.131	-.082	.069	-.075	.079

Note: Pearson correlation coefficient (r); Significance two tailed
** Significance level- p value <0.05; ** Significance level- p value <0.01*

For peer review only

For peer review only - http://bmjopen.bmj.com/site/about/guidelines.xhtml

Supplementary Table 7. Regression analysis of the association between the intussusception cases and meteorological parameters for the study sites.

Study sites/ Variables	Regression coefficient, β (95% CI)	Standard error, S_β	χ2 - value	p value
North region				
Srinagar (n=145)[#]				
Temperature (^{0}C)	0.043(0.003,0.082)	0.020	4.479	**0.034**
Rainfall (mm)	0.007(-0.004,0.018)	0.005	1.616	0.204
Wind Speed (Kmph)	0.150(-0.048,0.347)	0.100	2.207	0.137
Humidity (%)	0.001(-0.018,0.020)	0.009	0.010	0.921
Sunshine (hour)	0.005(0.000,0.010)	0.002	3.930	**0.047**
Delhi and Gurgaon (n=96)[*]				
Temperature (^{0}C)	0.025(-0.005,0.055)	0.015	2.712	0.100
Rainfall (mm)	-0.003(-0.012,0.006)	0.004	0.476	0.490
Wind Speed (Kmph)	0.229(0.068,0.390)	0.082	7.805	**0.005**
Humidity (%)	-0.021(-0.036,-0.005)	0.007	7.041	**0.008**
Sunshine (hour)	0.004(0.000,0.008)	0.002	4.205	**0.040**
Lucknow (n=110)[*]				
Temperature (^{0}C)	0.013(-0.014,0.041)	0.014	0.873	0.350
Rainfall (mm)	0.000(-0.008,0.008)	0.004	0.001	0.972
Wind Speed (Kmph)	0.224(0.074,0.374)	0.077	8.580	**0.003**
Humidity (%)	-0.008(-0.022,0.006)	0.007	1.195	0.274
Sunshine (hour)	0.004(0.000,0.007)	0.002	3.915	**0.048**
Indore (n=19)[*]				
Temperature (^{0}C)	0.036(-0.033,0.105)	0.035	1.055	0.304
Rainfall (mm)	-0.039(-0.078,0.001)	0.020	3.723	0.054
Wind Speed (Kmph)	0.623(0.193,1.054)	0.219	8.062	**0.005**
Humidity (%)	-0.059(-0.097,-0.021)	0.019	9.110	**0.003**
Sunshine (hour)	0.009(0.000,0.017)	0.004	3.556	0.059
East region				
Bhubaneswar/Cuttack (n=335)[#]				
Temperature (^{0}C)	0.012(-0.032,0.057)	0.023	.286	0.593
Rainfall (mm)	-0.001(-0.006,0.003)	0.002	.429	0.513
Wind Speed (Kmph)	0.060(-0.021,0.141)	0.041	2.131	0.144
Humidity (%)	-0.022(-0.044,0.000)	0.011	3.953	**0.047**
Sunshine (hour)	0.001(-0.003,0.005)	0.002	.157	0.692
Berhampur (n=30)[*]				
Temperature (^{0}C)	0.023(-0.068,0.114)	0.046	0.239	0.625
Rainfall (mm)	0.002(-0.006,0.01)	0.004	0.211	0.646
Wind Speed (Kmph)	0.027(-0.128,0.182)	0.079	0.118	0.731
Humidity (%)	0.009(-0.032,0.051)	0.021	0.186	0.666
Sunshine (hour)	0.001(-0.006,0.009)	0.004	0.125	0.724
Kolkata (n=80)[*]				
Temperature (^{0}C)	0.113(0.050,0.176)	0.032	12.448	**0.000**
Rainfall (mm)	-0.002(-0.007,0.003)	0.002	0.710	0.400
Wind Speed (Kmph)	0.233(0.134,0.332)	0.050	21.269	**0.000**
Humidity (%)	-0.032(-0.059,-0.005)	0.013	5.433	**0.020**
Sunshine (hour)	0.010(0.005,0.014)	0.002	16.577	**0.000**

For peer review only - http://bmjopen.bmj.com/site/about/guidelines.xhtml

Patna (n=19)*				
Temperature (^{0}C)	-0.067(-0.177,0.042)	0.056	1.452	0.228
Rainfall (mm)	-0.012(-0.025,0.001)	0.006	3.157	0.076
Wind Speed (Kmph)	-0.037(-0.235,0.161)	0.101	0.137	0.711
Humidity (%)	-0.063(-0.124,-0.003)	0.031	4.208	**0.040**
Sunshine (hour)	-0.002(-0.012,0.008)	0.005	0.150	0.698
Guwahati (n=112)*				
Temperature (^{0}C)	0.050(0.002,0.098)	0.025	4.097	**0.043**
Rainfall (mm)	0.000(-0.004,0.004)	0.002	0.000	0.995
Wind Speed (Kmph)	0.118(0.038,0.199)	0.041	8.408	**0.004**
Humidity (%)	-0.012(-0.034,0.01)	0.011	1.212	0.271
Sunshine (hour)	0.004(0.000,0.008)	0.002	3.207	0.073
Agartala (n=17)*				
Temperature (^{0}C)	0.060(-0.066,0.186)	0.064	0.874	0.350
Rainfall (mm)	0.005(-0.006,0.016)	0.005	0.838	0.360
Wind Speed (Kmph)	0.166(-0.042,0.374)	0.106	2.435	0.119
Humidity (%)	-0.005(-0.061,0.050)	0.028	0.037	0.848
Sunshine (hour)	0.001(-0.009,0.011)	0.005	0.033	0.857
South region				
Vishakhapatnam (n=77)*				
Temperature (^{0}C)	0.043(-0.058,0.144)	0.051	0.702	0.402
Rainfall (mm)	0.003(-0.007,0.012)	0.005	0.255	0.613
Wind Speed (Kmph)	-0.001(-0.071,0.070)	0.036	0.001	0.981
Humidity (%)	0.008(-0.069,0.085)	0.039	0.042	0.837
Sunshine (hour)	0.001(-0.005,0.007)	0.003	0.078	0.780
Hyderabad (n=2)*				
Temperature (^{0}C)	-0.088(-0.717,0.541)	0.321	0.075	0.784
Rainfall (mm)	-0.018(-0.085,0.048)	0.034	0.298	0.585
Wind Speed (Kmph)	-0.650(-2.074,0.774)	0.726	0.801	0.371
Humidity (%)	0.051(-0.436,0.539)	0.249	0.043	0.837
Sunshine (hour)	-0.017(-0.056,0.022)	0.020	0.764	0.382
Coimbatore (n=136)*				
Temperature (^{0}C)	-0.007(-0.082,0.068)	0.038	0.033	0.857
Rainfall (mm)	-0.003(-0.011,0.004)	0.004	0.825	0.364
Wind Speed (Kmph)	-0.053(-0.108,0.002)	0.028	3.506	0.061
Humidity (%)	-0.010(-0.067,0.047)	0.029	0.119	0.730
Sunshine (hour)	0.000(-0.004,0.005)	0.002	0.043	0.836
Chennai (n=92)*				
Temperature (^{0}C)	0.027(-0.065,0.120)	0.047	0.337	0.562
Rainfall (mm)	-0.001(-0.010,0.008)	0.005	0.036	0.850
Wind Speed (Kmph)	0.003(-0.062,0.067)	0.033	0.006	0.938
Humidity (%)	0.006(-0.064,0.076)	0.036	0.028	0.868
Sunshine (hour)	0.000(-0.006,0.005)	0.003	0.030	0.863
Trivandrum (n=774)*				
Temperature (^{0}C)	0.015(-0.016,0.047)	0.016	0.895	0.344
Rainfall (mm)	-0.005(-0.008,-0.002)	0.002	10.159	**0.001**
Wind Speed (Kmph)	-0.001(-0.024,0.021)	0.011	0.016	.898
Humidity (%)	-0.044(-0.068,-0.020)	0.012	12.938	**0.000**

For peer review only - http://bmjopen.bmj.com/site/about/guidelines.xhtml

Sunshine (hour)	0.002(0.001,0.004)	0.001	6.538	**0.011**
West region				
Mumbai (n=34)*				
Temperature (^{0}C)	-0.040(-0.165,0.085)	0.064	0.387	0.534
Rainfall (mm)	-0.003(-0.011,0.005)	0.004	0.580	0.446
Wind Speed (Kmph)	-0.016(-0.140,0.108)	0.063	0.065	0.799
Humidity (%)	-0.013(-0.039,0.013)	0.013	0.929	0.335
Sunshine (hour)	0.001(-0.006,0.008)	0.004	0.108	0.742
Jamnagar (n=54)*				
Temperature (^{0}C)	0.066(-0.042,0.173)	0.055	1.440	0.230
Rainfall (mm)	-0.001(-0.006,0.005)	0.003	0.035	0.852
Wind Speed (Kmph)	0.006(-0.090,0.102)	0.049	0.015	0.903
Humidity (%)	0.003(-0.017,0.023)	0.010	0.080	0.777
Sunshine (hour)	0.003(-0.002,0.009)	0.003	1.305	0.253
Jaipur (n=29)*				
Temperature (^{0}C)	0.150(-0.009,0.309)	0.081	3.425	0.064
Rainfall (mm)	-0.003(-0.012,0.005)	0.004	0.600	0.439
Wind Speed (Kmph)	0.092(-0.031,0.216)	0.063	2.141	0.143
Humidity (%)	0.000(-0.027,0.027)	0.014	0.000	0.993
Sunshine (hour)	0.005(-0.003,0.013)	0.004	1.471	0.225

*Note: Tests used for regression analysis: * Poisson Regression; # Negative Binomial Regression*

For peer review only

Supplementary Table 8. Factorial analysis-of-variance of intussusception in children with season and age groups

Source	Dependent variables	Type III Sum of squares	Degree of freedom	Mean square	F	p value
Corrected model	Seasonal IS cases	285.847[a]	38	7.522	1.442	**.040**
	Seasonal IS cases of 2-6 months	49.145[b]	38	1.293	1.579	**.014**
	Seasonal IS cases of 7-12 months	45.682[c]	38	1.202	1.012	.449
	Seasonal IS cases of 13-24 months	31.855[d]	38	.838	1.334	.085
Intercept	Seasonal IS cases	789.283	1	789.283	151.302	.000
	Seasonal IS cases of 2-6 months	101.336	1	101.336	123.760	.000
	Seasonal IS cases of 7-12 months	135.705	1	135.705	114.285	.000
	Seasonal IS cases of 13-24 months	40.684	1	40.684	64.740	.000
Season	Seasonal IS cases	8.674	4	2.169	.416	.797
	Seasonal IS cases of 2-6 months	1.930	4	.482	.589	.670
	Seasonal IS cases of 7-12 months	3.207	4	.802	.675	.609
	Seasonal IS cases of 13-24 months	.856	4	.214	.340	.851
Season year	Seasonal IS cases	82.483	7	11.783	2.259	**.027**
	Seasonal IS cases of 2-6 months	17.669	7	2.524	3.083	**.003**
	Seasonal IS cases of 7-12 months	3.504	7	.501	.422	.889
	Seasonal IS cases of 13-24 months	11.481	7	1.640	2.610	**.011**
Season* Season year	Seasonal IS cases	57.160	27	2.117	.406	.997
	Seasonal IS cases of 2-6 months	13.795	27	.511	.624	.934
	Seasonal IS cases of 7-12 months	19.521	27	.723	.609	.943
	Seasonal IS cases of 13-24 months	7.717	27	.286	.455	.993
Error	Seasonal IS cases	9092.547	1743	5.217		
	Seasonal IS cases of 2-6 months	1427.189	1743	.819		
	Seasonal IS cases of 7-12 months	2069.674	1743	1.187		
	Seasonal IS cases of 13-24 months	1095.327	1743	.628		
Total	Seasonal IS cases	11999.000	1782			
	Seasonal IS cases of 2-6 months	1816.000	1782			
	Seasonal IS cases of 7-12 months	2545.000	1782			
	Seasonal IS cases of 13-24 months	1272.000	1782			
Modified total	Seasonal IS cases	9378.393	1781			
	Seasonal IS cases of 2-6 months	1476.334	1781			
	Seasonal IS cases of 7-12 months	2115.356	1781			
	Seasonal IS cases of 13-24 months	1127.183	1781			

Note: a: R squared= .030 (adjusted R squared= .009); b: R squared= .033 (adjusted R squared= .012); c: R squared = .022 (adjusted R squared= .000); d: R squared= .028 (adjusted R squared= .007)

For peer review only - http://bmjopen.bmj.com/site/about/guidelines.xhtml

Supplementary Table 9. Factorial analysis-of-variance of intussusception in children with season and regions

Source	Dependent variables	Type III Sum of squares	Degree of freedom	Mean square	F	p value
Corrected model	Seasonal IS cases	285.847[a]	38	7.522	1.442	**.040**
	Seasonal IS cases of North region	36.237[b]	38	.954	1.809	**.002**
	Seasonal IS cases of South region	55.831[c]	38	1.469	.321	1.000
	Seasonal IS cases of East region	53.071[d]	38	1.397	1.333	.085
	Seasonal IS cases of West region	2.721[e]	38	.072	.662	.944
Intercept	Seasonal IS cases	789.283	1	789.283	151.302	**.000**
	Seasonal IS cases of North region	29.881	1	29.881	56.676	**.000**
	Seasonal IS cases of South region	180.247	1	180.247	39.373	**.000**
	Seasonal IS cases of East region	59.322	1	59.322	56.607	**.000**
	Seasonal IS cases of West region	2.250	1	2.250	20.798	**.000**
Season	Seasonal IS cases	8.674	4	2.169	.416	.797
	Seasonal IS cases of North region	10.274	4	2.569	4.872	**.001**
	Seasonal IS cases of South region	9.239	4	2.310	.505	.732
	Seasonal IS cases of East region	1.801	4	.450	.430	.787
	Seasonal IS cases of West region	.207	4	.052	.479	.752
Season year	Seasonal IS cases	82.483	7	11.783	2.259	**.027**
	Seasonal IS cases of North region	11.119	7	1.588	3.013	**.004**
	Seasonal IS cases of South region	5.618	7	.803	.175	.990
	Seasonal IS cases of East region	18.661	7	2.666	2.544	**.013**
	Seasonal IS cases of West region	.464	7	.066	.612	.746
Season* Season year	Seasonal IS cases	57.160	27	2.117	.406	.997
	Seasonal IS cases of North region	8.198	27	.304	.576	.960
	Seasonal IS cases of South region	28.377	27	1.051	.230	1.000
	Seasonal IS cases of East region	15.527	27	.575	.549	.971
	Seasonal IS cases of West region	1.325	27	.049	.454	.993
Error	Seasonal IS cases	9092.547	1743	5.217		
	Seasonal IS cases of North region	918.939	1743	.527		
	Seasonal IS cases of South region	7979.411	1743	4.578		
	Seasonal IS cases of East region	1826.595	1743	1.048		
	Seasonal IS cases of West region	188.597	1743	.108		
Total	Seasonal IS cases	11999.000	1782			
	Seasonal IS cases of North region	1032.000	1782			
	Seasonal IS cases of South region	8691.000	1782			
	Seasonal IS cases of East region	2077.000	1782			
	Seasonal IS cases of West region	199.000	1782			
Modified total	Seasonal IS cases	9378.393	1781			
	Seasonal IS cases of North region	955.176	1781			
	Seasonal IS cases of South region	8035.242	1781			
	Seasonal IS cases of East region	1879.666	1781			
	Seasonal IS cases of West region	191.318	1781			

Note: a: R squared = .030 (adjusted R squared= .009); b: R squared = .038 (adjusted R squared= .017); c: R squared = .007 (adjusted R squared= -.015); d: R squared= .028 (adjusted R squared= .007); e: R squared = .014 (adjusted R squared= -.007)

For peer review only - http://bmjopen.bmj.com/site/about/guidelines.xhtml

Supplementary Table 10. Multiple comparisons of seasonal intussusception in children in different seasons pooled for all sites

Dependent variable		Season (A)	Season (B)	Mean difference (A-B)	Standard error	p value	95% CI
Seasonal IS cases pooled	Tukey HSD	Monsoon	Spring	-.175	.2285	.940	-0.8 , 0.45
			Summer	-.409	.1643	.093	-0.86 , 0.04
			Monsoon	-.038	.1573	.999	-0.47 , 0.39
			Autumn	.160	.1758	.892	-0.32 , 0.64
		Spring	Winter	.175	.2285	.940	-0.45 , 0.8
			Summer	-.234	.2221	.831	-0.84 , 0.37
			Monsoon	.137	.2169	.970	-0.46 , 0.73
			Autumn	.336	.2307	.592	-0.29 , 0.97
		Summer	Winter	.409	.1643	.093	-0.04 , 0.86
			Spring	.234	.2221	.831	-0.37 , 0.84
			Monsoon	.371	.1478	.089	-0.03 , 0.77
			Autumn	.569*	.1673	**.006**	0.11 , 1.03
		Monsoon	Winter	.038	.1573	.999	-0.39 , 0.47
			Spring	-.137	.2169	.970	-0.73 , 0.46
			Summer	-.371	.1478	.089	-0.77 , 0.03
			Autumn	.199	.1604	.729	-0.24 , 0.64
		Autumn	Winter	-.160	.1758	.892	-0.64 , 0.32
			Spring	-.336	.2307	.592	-0.97 , 0.29
			Summer	-.569*	.1673	**.006**	-1.03 , -0.11
			Monsoon	-.199	.1604	.729	-0.64 , 0.24

*Note: * The difference is significant at p<0.05 level.*

For peer review only

Supplementary Table 11. Multiple comparisons of seasonal intussusception in children in different seasons at regional level

Dependent variable		Season (A)	Season (B)	Mean difference (A-B)	Standard error	p value	95% CI
North Region	Tukey HSD	Winter	Spring	-.151	.0726	.231	-0.35 , 0.05
			Summer	-.039	.0522	.943	-0.18 , 0.1
			Monsoon	.125	.0500	.089	-0.01 , 0.26
			Autumn	.139	.0559	.093	-0.01 , 0.29
		Spring	Winter	.151	.0726	.231	-0.05 , 0.35
			Summer	.111	.0706	.512	-0.08 , 0.3
			Monsoon	.276*	.0690	**.001**	0.09 , 0.46
			Autumn	.290*	.0733	**.001**	0.09 , 0.49
		Summer	Winter	.039	.0522	.943	-0.1 , 0.18
			Spring	-.111	.0706	.512	-0.3 , 0.08
			Monsoon	.165*	.0470	**.004**	0.04 , 0.29
			Autumn	.179*	.0532	**.007**	0.03 , 0.32
		Monsoon	Winter	-.125	.0500	.089	-0.26 , 0.01
			Spring	-.276*	.0690	**.001**	-0.46 , -0.09
			Summer	-.165*	.0470	**.004**	-0.29 , -0.04
			Autumn	.014	.0510	.999	-0.13 , 0.15
		Autumn	Winter	-.139	.0559	.093	-0.29 , 0.01
			Spring	-.290*	.0733	**.001**	-0.49 , -0.09
			Summer	-.179*	.0532	**.007**	-0.32 , -0.03
			Monsoon	-.014	.0510	.999	-0.15 , 0.13
South Region	Tukey HSD	Winter	Spring	-.014	.2140	1.000	-0.6 , 0.57
			Summer	-.197	.1539	.705	-0.62 , 0.22
			Monsoon	-.141	.1473	.875	-0.54 , 0.26
			Autumn	.032	.1647	1.000	-0.42 , 0.48
		Spring	Winter	.014	.2140	1.000	-0.57 , 0.6
			Summer	-.183	.2080	.905	-0.75 , 0.39
			Monsoon	-.127	.2032	.971	-0.68 , 0.43
			Autumn	.046	.2161	1.000	-0.54 , 0.64
		Summer	Winter	.197	.1539	.705	-0.22 , 0.62
			Spring	.183	.2080	.905	-0.39 , 0.75
			Monsoon	.056	.1384	.994	-0.32 , 0.43
			Autumn	.229	.1568	.588	-0.2 , 0.66
		Monsoon	Winter	.141	.1473	.875	-0.26 , 0.54
			Spring	.127	.2032	.971	-0.43 , 0.68
			Summer	-.056	.1384	.994	-0.43 , 0.32
			Autumn	.173	.1503	.779	-0.24 , 0.58
		Autumn	Winter	-.032	.1647	1.000	-0.48 , 0.42
			Spring	-.046	.2161	1.000	-0.64 , 0.54
			Summer	-.229	.1568	.588	-0.66 , 0.2
			Monsoon	-.173	.1503	.779	-0.58 , 0.24
East region	Tukey HSD	Winter	Spring	.035	.1024	.997	-0.24 , 0.31
			Summer	-.125	.0736	.433	-0.33 , 0.08
			Monsoon	-.001	.0705	1.000	-0.19 , 0.19

For peer review only - http://bmjopen.bmj.com/site/about/guidelines.xhtml

			Autumn	.010	.0788	1.000	-0.21 , 0.23
		Spring	Winter	-.035	.1024	.997	-0.31 , 0.24
			Summer	-.160	.0995	.490	-0.43 , 0.11
			Monsoon	-.036	.0972	.996	-0.3 , 0.23
			Autumn	-.025	.1034	.999	-0.31 , 0.26
		Summer	Winter	.125	.0736	.433	-0.08 , 0.33
			Spring	.160	.0995	.490	-0.11 , 0.43
			Monsoon	.124	.0662	.331	-0.06 , 0.31
			Autumn	.135	.0750	.372	-0.07 , 0.34
		Monsoon	Winter	.001	.0705	1.000	-0.19 , 0.19
			Spring	.036	.0972	.996	-0.23 , 0.3
			Summer	-.124	.0662	.331	-0.31 , 0.06
			Autumn	.011	.0719	1.000	-0.19 , 0.21
		Autumn	Winter	-.010	.0788	1.000	-0.23 , 0.21
			Spring	.025	.1034	.999	-0.26 , 0.31
			Summer	-.135	.0750	.372	-0.34 , 0.07
			Monsoon	-.011	.0719	1.000	-0.21 , 0.19
West region	Tukey HSD	Winter	Spring	-.046	.0329	.637	-0.14 , 0.04
			Summer	-.048	.0237	.260	-0.11 , 0.02
			Monsoon	-.022	.0227	.872	-0.08 , 0.04
			Autumn	-.021	.0253	.921	-0.09 , 0.05
		Spring	Winter	.046	.0329	.637	-0.04 , 0.14
			Summer	-.002	.0320	1.000	-0.09 , 0.09
			Monsoon	.024	.0312	.941	-0.06 , 0.11
			Autumn	.025	.0332	.947	-0.07 , 0.12
		Summer	Winter	.048	.0237	.260	-0.02 , 0.11
			Spring	.002	.0320	1.000	-0.09 , 0.09
			Monsoon	.026	.0213	.743	-0.03 , 0.08
			Autumn	.027	.0241	.805	-0.04 , 0.09
		Monsoon	Winter	.022	.0227	.872	-0.04 , 0.08
			Spring	-.024	.0312	.941	-0.11 , 0.06
			Summer	-.026	.0213	.743	-0.08 , 0.03
			Autumn	.001	.0231	1.000	-0.06 , 0.06
		Autumn	Winter	.021	.0253	.921	-0.05 , 0.09
			Spring	-.025	.0332	.947	-0.12 , 0.07
			Summer	-.027	.0241	.805	-0.09 , 0.04
			Monsoon	-.001	.0231	1.000	-0.06 , 0.06

*Note: * The difference is significant at p<0.05 level.*

For peer review only - http://bmjopen.bmj.com/site/about/guidelines.xhtml

Supplementary Table 12. Multiple comparisons of seasonal intussusception in children in different seasons for different age groups

Dependent variable		Season (A)	Season (B)	Mean difference (A-B)	Standard error	p value	95% CI
Seasonal IS cases in children aged 2-6 months	Tukey HSD	Winter	Spring	-.003	.0905	1.000	-0.25 , 0.24
			Summer	-.073	.0651	.798	-0.25 , 0.11
			Monsoon	.019	.0623	.998	-0.15 , 0.19
			Autumn	.123	.0696	.395	-0.07 , 0.31
		Spring	Winter	.003	.0905	1.000	-0.24 , 0.25
			Summer	-.070	.0880	.933	-0.31 , 0.17
			Monsoon	.022	.0859	.999	-0.21 , 0.26
			Autumn	.126	.0914	.643	-0.12 , 0.38
		Summer	Winter	.073	.0651	.798	-0.11 , 0.25
			Spring	.070	.0880	.933	-0.17 , 0.31
			Monsoon	.092	.0585	.517	-0.07 , 0.25
			Autumn	.195*	.0663	**.027**	0.01 , 0.38
		Monsoon	Winter	-.019	.0623	.998	-0.19 , 0.15
			Spring	-.022	.0859	.999	-0.26 , 0.21
			Summer	-.092	.0585	.517	-0.25 , 0.07
			Autumn	.104	.0636	.478	-0.07 , 0.28
		Autumn	Winter	-.123	.0696	.395	-0.31 , 0.07
			Spring	-.126	.0914	.643	-0.38 , 0.12
			Summer	-.195*	.0663	**.027**	-0.38 , -0.01
			Monsoon	-.104	.0636	.478	-0.28 , 0.07
Seasonal IS cases in children aged 7-12 months	Tukey HSD	Winter	Spring	-.185	.1090	.439	-0.48 , 0.11
			Summer	-.210	.0784	.057	-0.42 , 0
			Monsoon	-.046	.0750	.973	-0.25 , 0.16
			Autumn	.021	.0839	.999	-0.21 , 0.25
		Spring	Winter	.185	.1090	.439	-0.11 , 0.48
			Summer	-.026	.1059	.999	-0.32 , 0.26
			Monsoon	.138	.1035	.667	-0.14 , 0.42
			Autumn	.206	.1101	.336	-0.1 , 0.51
		Summer	Winter	.210	.0784	.057	0 , 0.42
			Spring	.026	.1059	.999	-0.26 , 0.32
			Monsoon	.164	.0705	.136	-0.03 , 0.36
			Autumn	.231*	.0798	**.031**	0.01 , 0.45
		Monsoon	Winter	.046	.0750	.973	-0.16 , 0.25
			Spring	-.138	.1035	.667	-0.42 , 0.14
			Summer	-.164	.0705	.136	-0.36 , 0.03
			Autumn	.067	.0765	.906	-0.14 , 0.28
		Autumn	Winter	-.021	.0839	.999	-0.25 , 0.21
			Spring	-.206	.1101	.336	-0.51 , 0.1
			Summer	-.231*	.0798	**.031**	-0.45 , -0.01
			Monsoon	-.067	.0765	.906	-0.28 , 0.14
Seasonal IS cases in	Tukey HSD	Winter	Spring	.012	.0793	1.000	-0.2 , 0.23
			Summer	-.126	.0570	.176	-0.28 , 0.03
			Monsoon	-.011	.0546	1.000	-0.16 , 0.14

For peer review only

For peer review only - http://bmjopen.bmj.com/site/about/guidelines.xhtml

children aged 7-12 months			Autumn	.016	.0610	.999	-0.15 , 0.18
		Spring	Winter	-.012	.0793	1.000	-0.23 , 0.2
			Summer	-.138	.0771	.378	-0.35 , 0.07
			Monsoon	-.024	.0753	.998	-0.23 , 0.18
			Autumn	.004	.0801	1.000	-0.21 , 0.22
		Summer	Winter	.126	.0570	.176	-0.03 , 0.28
			Spring	.138	.0771	.378	-0.07 , 0.35
			Monsoon	.115	.0513	.167	-0.03 , 0.25
			Autumn	.143	.0581	.102	-0.02 , 0.3
		Monsoon	Winter	.011	.0546	1.000	-0.14 , 0.16
			Spring	.024	.0753	.998	-0.18 , 0.23
			Summer	-.115	.0513	.167	-0.25 , 0.03
			Autumn	.028	.0557	.987	-0.12 , 0.18
		Autumn	Winter	-.016	.0610	.999	-0.18 , 0.15
			Spring	-.004	.0801	1.000	-0.22 , 0.21
			Summer	-.143	.0581	.102	-0.3 , 0.02
			Monsoon	-.028	.0557	.987	-0.18 , 0.12

*Note: * The difference is significant at p<0.05 level.*

For peer review only

Supplementary Table 13. Intussusception cases in children at different temperature ranges at pooled and regional level

Pooled/ Region	Temperature range (°C)	Years of data collection								Total
		2010	2011	2012	2013	2014	2015	2016	2017	
Pooled	<20°C	8	14	24	29	26	39	55	51	246
	21°C -25°C	2	31	44	32	43	54	42	31	279
	26°C -30°C	56	149	153	166	164	208	245	164	1305
	>30°C	2	15	23	33	45	63	88	62	331
	Total	68	209	244	260	278	364	430	308	2161
North Region	<20°C	5	8	18	28	20	27	45	46	197
	21°C -25°C	1	5	3	2	10	7	4	1	33
	26°C -30°C	1	2	4	3	4	6	13	3	36
	>30°C	2	4	12	11	14	21	29	11	104
	Total	9	19	37	44	48	61	91	61	370
South Region	<20°C	0	0	0	0	0	0	0	0	0
	21°C -25°C	1	14	15	10	7	11	11	8	77
	26°C -30°C	49	117	113	132	116	157	167	116	967
	>30°C	0	3	2	7	1	6	6	12	37
	Total	50	134	130	149	124	174	184	136	1081
East Region	<20°C	3	6	6	1	5	10	9	4	44
	21°C -25°C	0	10	21	17	22	36	23	21	150
	26°C -30°C	6	24	26	23	37	36	53	35	240
	>30°C	0	5	8	13	20	30	46	37	159
	Total	9	45	61	54	84	112	131	97	593
West Region	<20°C	0	0	0	0	1	2	1	1	5
	21°C -25°C	0	2	5	3	4	0	4	1	19
	26°C -30°C	0	6	10	8	7	9	12	10	62
	>30°C	0	3	1	2	10	6	7	2	31
	Total	0	11	16	13	22	17	24	14	117

For peer review only

Supplementary Table 14. Variations in the intussusception cases between the different temperature categories by Kruskal-Wallis H test

Level	df	χ^2-statistic	P- value
Pooled	3	15.933	< 0.001
North Region	3	21.054	< 0.001
South Region	3	50.625	< 0.001
East Region	3	3.502	0.3205
West Region	3	5.606	0.1324

Notes: Pooled- all sites/regions combined; df: degrees of freedom; χ^2-statistic: critical chi square value

For peer review only

Title: Intussusception in children under-two years, seasonal variations and associations with meteorological parameters: Results from multisite bidirectional surveillance in India over eight years

Corresponding author: Manoja Kumar Das, MD

The IRBS reviewed and approved the study protocol titled "Intussusception in Infants: Multisite Retrospective Surveillance in India"

1. Name of IRB: The INCLEN Independent Ethic Committee
   Name of the Institute: The INCLEN Trust International, New Delhi, India
   Principal Investigator's Institute/Coordinating Institute

2. Name of IRB: The INCLEN Independent Ethic Committee
   Name of the Institute: King George's Medical University, Lucknow, Uttar Pradesh, India

3. Name of IRB: Institutional Ethics Committee
   Name of the Institute: MP Shah Government Medical College, Jamnagar, Gujarat, India

4. Name of IRB: Institutional Ethics Committee
   Name of the Institute: Sher-I-Kashmir Institute of Medical Sciences, Srinagar, Jammu & Kashmir, India

5. Name of IRB: Institutional Ethics Committee
   Name of the Institute: Gauhati Medical College, Guwahati, Assam, India

6. Name of IRB: Institutional Human Ethics Committee
   Name of the Institute: PSG Institute of Medical Sciences, Coimbatore, Tamil Nadu, India

7. Name of IRB: Institutional Ethics Committee
   Name of the Institute: King George Hospital, Andhra Medical College, Vishakhapatnam, Andhra Pradesh, India

8. Name of IRB: Institutional Ethics Committee
   Name of the Institute: Fortis Escorts Hospital, Jaipur, Rajasthan, India

9. Name of IRB: Institutional Ethics Committee
   Name of the Institute: Grant Medical College & JJ Hospital, Mumbai, Maharashtra, India

10. Name of IRB: Institutional Ethics Committee
    Name of the Institute: Government Medical College & SAT Hospital, Thiruvananthapuram, Kerala, India

11. Name of IRB: Medanta Institutional Ethics Committee
    Name of the Institute: Medanta—The Medicity, Gurgaon, Haryana, India

12. Name of IRB: Institutional Ethics Committee
    Name of the Institute: SCB Medical College, Cuttack, Odisha, India

13. Name of IRB: Institutional Ethics Committee

For peer review only

Name of the Institute: IMS & SUM Medical College & Hospital, Bhubaneswar, Odisha, India

14. Name of IRB: Institutional Ethics Committee
    Name of the Institute: Choithram Hospital and Research Centre, Indore, Madhya Pradesh, India
15. Name of IRB: Institutional Ethics Committee
    Name of the Institute: Agartala Government Medical College, Agartala, Tripura, India

16. Name of IRB: Institutional Ethics Committee- Clinical Studies
    Name of the Institute: Apollo Hospitals, Chennai, Tamil Nadu

17. Name of IRB: Institutional Ethics Committee
    Name of the Institute: Maulana Azad Medical College, Delhi, India

18. Name of IRB: Institutional Ethics Committee
    Name of the Institute: Institute of Post Graduate Medical Education and Research, Kolkata, West Bengal, India

19. Name of IRB: Institutional Ethics Committee
    Name of the Institute: Indira Gandhi Institute of Medical Sciences, Patna, Bihar, India

20. Name of IRB: Ethics Committee
    Name of the Institute: Apollo Hospital, Hyderabad, Telengana, India

For peer review only

Manuscript Title: Intussusception in children under-two years, seasonal variations and associations with meteorological parameters: Results from multisite bidirectional surveillance in India over eight years

STROBES Checklist

Section/Topic	Item #	Recommendation	Reported on page #
**Title and abstract**	1	(*a*) Indicate the study's design with a commonly used term in the title or the abstract	1
		(*b*) Provide in the abstract an informative and balanced summary of what was done and what was found	1-2
**Introduction**			
Background/rationale	2	Explain the scientific background and rationale for the investigation being reported	4
Objectives	3	State specific objectives, including any pre-specified hypotheses	5
**Methods**			
Study design	4	Present key elements of study design early in the paper	5
Setting	5	Describe the setting, locations, and relevant dates, including periods of recruitment, exposure, follow-up, and data collection	5
Participants	6	(*a*) *Cohort study*—Give the eligibility criteria, and the sources and methods of selection of participants. Describe methods of follow-up<br>*Case-control study*—Give the eligibility criteria, and the sources and methods of case ascertainment and control selection. Give the rationale for the choice of cases and controls<br>*Cross-sectional study*—Give the eligibility criteria, and the sources and methods of selection of participants	5
		(*b*) *Cohort study*—For matched studies, give matching criteria and number of exposed and unexposed<br>*Case-control study*—For matched studies, give matching criteria and the number of controls per case	
Variables	7	Clearly define all outcomes, exposures, predictors, potential confounders, and effect modifiers. Give diagnostic criteria, if applicable	5-6
Data sources/ measurement	8*	For each variable of interest, give sources of data and details of methods of assessment (measurement). Describe comparability of assessment methods if there is more than one group	5-6
Bias	9	Describe any efforts to address potential sources of bias	6

For peer review only

Study size	10	Explain how the study size was arrived at	5-6
Quantitative variables	11	Explain how quantitative variables were handled in the analyses. If applicable, describe which groupings were chosen and why	6
Statistical methods	12	(*a*) Describe all statistical methods, including those used to control for confounding	6-7
		(*b*) Describe any methods used to examine subgroups and interactions	6-7
		(*c*) Explain how missing data were addressed	6
		(*d*) *Cohort study*—If applicable, explain how loss to follow-up was addressed<br>*Case-control study*—If applicable, explain how matching of cases and controls was addressed<br>*Cross-sectional study*—If applicable, describe analytical methods taking account of sampling strategy	6-7
		(*e*) Describe any sensitivity analyses	6-7
**Results**			
Participants	13*	(a) Report numbers of individuals at each stage of study—eg numbers potentially eligible, examined for eligibility, confirmed eligible, included in the study, completing follow-up, and analysed	7
		(b) Give reasons for non-participation at each stage	NA
		(c) Consider use of a flow diagram	Narration given
Descriptive data	14*	(a) Give characteristics of study participants (eg demographic, clinical, social) and information on exposures and potential confounders	7-8
		(b) Indicate number of participants with missing data for each variable of interest	7-8 and tables
		(c) *Cohort study*—Summarise follow-up time (eg, average and total amount)	
Outcome data	15*	*Cohort study*—Report numbers of outcome events or summary measures over time	7-11<br>Given in tables and results
		Case-control study—Report numbers in each exposure category, or summary measures of exposure	
		Cross-sectional study—Report numbers of outcome events or summary measures	
Main results	16	(*a*) Give unadjusted estimates and, if applicable, confounder-adjusted estimates and their precision (eg, 95% confidence interval). Make clear which confounders were adjusted for and why they were included	7-11<br>Given in tables and results
		(*b*) Report category boundaries when continuous variables were categorized	7-11<br>Given in tables and

For peer review only

			results
		(*c*) If relevant, consider translating estimates of relative risk into absolute risk for a meaningful time period	NA
Other analyses	17	Report other analyses done—eg analyses of subgroups and interactions, and sensitivity analyses	7-11<br>Given in tables and results
**Discussion**			
Key results	18	Summarise key results with reference to study objectives	11-12
Limitations	19	Discuss limitations of the study, taking into account sources of potential bias or imprecision. Discuss both direction and magnitude of any potential bias	14
Interpretation	20	Give a cautious overall interpretation of results considering objectives, limitations, multiplicity of analyses, results from similar studies, and other relevant evidence	11-14
Generalisability	21	Discuss the generalisability (external validity) of the study results	14
**Other information**			
Funding	22	Give the source of funding and the role of the funders for the present study and, if applicable, for the original study on which the present article is based	17

*Give information separately for cases and controls in case-control studies and, if applicable, for exposed and unexposed groups in cohort and cross-sectional studies.

Note: An Explanation and Elaboration article discusses each checklist item and gives methodological background and published examples of transparent reporting. The STROBE checklist is best used in conjunction with this article (freely available on the Web sites of PLoS Medicine at http://www.plosmedicine.org/, Annals of Internal Medicine at http://www.annals.org/, and Epidemiology at http://www.epidem.com/). Information on the STROBE Initiative is available at www.strobe-statement.org.

For peer review only

BMJ Open

Association of meteorological parameters with intussusception in children aged under-two years: Results from a multisite bidirectional surveillance over seven years in India

Journal:	*BMJ Open*
Manuscript ID	bmjopen-2020-043687.R1
Article Type:	Original research
Date Submitted by the Author:	07-Dec-2020
Complete List of Authors:	Study Group, The INCLEN Intussusception Surveillance; The INCLEN Trust International<br>Das, Manoja; The INCLEN Trust International, The INCLEN Trust International
**Primary Subject Heading**:	Public health
Secondary Subject Heading:	Epidemiology, Paediatrics
Keywords:	EPIDEMIOLOGY, Public health < INFECTIOUS DISEASES, Community child health < PAEDIATRICS, Paediatric gastroenterology < PAEDIATRICS

SCHOLARONE™
Manuscripts

I, the Submitting Author has the right to grant and does grant on behalf of all authors of the Work (as defined in the below author licence), an exclusive licence and/or a non-exclusive licence for contributions from authors who are: i) UK Crown employees; ii) where BMJ has agreed a CC-BY licence shall apply, and/or iii) in accordance with the terms applicable for US Federal Government officers or employees acting as part of their official duties; on a worldwide, perpetual, irrevocable, royalty-free basis to BMJ Publishing Group Ltd ("BMJ") its licensees and where the relevant Journal is co-owned by BMJ to the co-owners of the Journal, to publish the Work in this journal and any other BMJ products and to exploit all rights, as set out in our licence.

The Submitting Author accepts and understands that any supply made under these terms is made by BMJ to the Submitting Author unless you are acting as an employee on behalf of your employer or a postgraduate student of an affiliated institution which is paying any applicable article publishing charge ("APC") for Open Access articles. Where the Submitting Author wishes to make the Work available on an Open Access basis (and intends to pay the relevant APC), the terms of reuse of such Open Access shall be governed by a Creative Commons licence – details of these licences and which Creative Commons licence will apply to this Work are set out in our licence referred to above.

Other than as permitted in any relevant BMJ Author's Self Archiving Policies, I confirm this Work has not been accepted for publication elsewhere, is not being considered for publication elsewhere and does not duplicate material already published. I confirm all authors consent to publication of this Work and authorise the granting of this licence.

For peer review only

Article Type: Original Research

Revised Title: Association of meteorological parameters with intussusception in children aged under-two years: Results from a multisite bidirectional surveillance over seven years in India

Short title: Association of intussusception in Indian children with meteorological parameters

For peer review only

Author Information

The INCLEN Intussusception Surveillance Network Study Group

The INCLEN Trust International, Okhla Industrial Area, Phase 1, New Delhi -110020, India

E-mail: drmanojkdas@gmail.com

Corresponding Author

Manoja Kumar Das

Director Projects, The INCLEN Trust International, F1/5, Okhla Industrial Area, Phase 1, New Delhi -110020, INDIA

Phone: Off: +91-11-47730000; Mob: +91-9810203768; Fax: +91-11-47730001

E-mail: manoj@inclentrust.org

For peer review only - http://bmjopen.bmj.com/site/about/guidelines.xhtml

Author information

'The INCLEN Intussusception Surveillance Network Study Group' list of authors

1. Manoja Kumar Das

   Director Projects, The INCLEN Trust International, New Delhi, India

   Email: manoj@inclentrust.org

2. Narendra Kumar Arora

   Executive Director, The INCLEN Trust International, New Delhi, India

   Email: nkarora@inclentrust.org

3. Bini Gupta

   Assistant Research Officer, The INCLEN Trust International, New Delhi, India

   Email: drbini.gupta02@gmail.com

4. Sharad Srivastava

   Assistant Program Officer, The INCLEN Trust International, New Delhi, India

   Email: sharad.srivastav@inclentrust.org

5. Apoorva Sharan

   Research Officer, The INCLEN Trust International, New Delhi, India

   Email: apoorva@inclentrust.org

6. Arindam Ray

   Senior Program Officer, Bill and Melinda Gates Foundation, India Country Office, New Delhi, India

   Email: arindam.ray@gatesfoundation.org

7. Ashish Wakhlu

   Professor, Department of Paediatric Surgery, King George's Medical University, Lucknow, Uttar Pradesh, India

   Email: ashish_wakhlu@hotmail.com

For peer review only

8. Bhadresh R Vyas

Professor, Department of Paediatrics, MP Shah Government Medical College, Jamnagar, Gujarat, India

Email: bhadreshrvyas@yahoo.co.uk

9. Javeed Iqbal Bhat

Assistant Professor, Department of Paediatrics, Sher-I-Kashmir Institute of Medical Sciences, Srinagar, Jammu & Kashmir, India

Email: drjaveediqbal@gmail.com

10. Jayanta K. Goswami

Professor, Department of Paediatric Surgery, Gauhati Medical College, Guwahati, Assam, India

Email: jayantagoswami@hotmail.com

11. John Mathai

Professor, Department of Paediatrics, PSG Institute of Medical Sciences, Coimbatore, Tamil Nadu, India

Email: psg_peds@yahoo.com

12. Kameswari K.

Professor, Department of Paediatric Surgery, Andhra Medical College, Vishakhapatnam, Andhra Pradesh, India

Email: kameswari1956@gmail.com

13. Lalit Bharadia

Consultant Paediatric Gastroenterologist, Fortis Escorts Hospital, Jaipur, Rajasthan, India

Email: lalitbharadia@gmail.com

14. Lalit Sankhe

For peer review only

Assistant Professor, Department of Community Medicine, Grant Medical College & JJ Hospital, Mumbai, Maharashtra, India

Email: sankhelalit@yahoo.com

15. Ajayakumar M.K.

Professor, Department of Paediatric Surgery, Government Medical College & SAT Hospital, Thiruvananthapuram, Kerala, India

Email: drajaykumarmk@gmail.com

16. Neelam Mohan

Consultant Paediatrics Gastroenterology, Medanta—The Medicity, Gurgaon, Haryana, India

Email: drneelam@yahoo.com

17. Pradeep K. Jena

Professor, Department of Paediatric Surgery, SCB Medical College, Cuttack, Odisha, India

Email: drpkjena@gmail.com

18. Rachita Sarangi

Professor, Department of Paediatrics, IMS & SUM Medical College & Hospital, Bhubaneswar, Odisha, India

Email: rachitapaedia@gmail.com

19. Rashmi Shad

Consultant Paediatrics, Choithram Hospital and Research Centre, Indore, Madhya Pradesh, India

Email: drrashmishad@yahoo.com

20. Sanjib K. Debbarma

Associate Professor, Department of Paediatrics, Agartala Government Medical College, Agartala, Tripura, India

For peer review only

For peer review only - http://bmjopen.bmj.com/site/about/guidelines.xhtml

Email: dr_sanjibdb@rediffmail.com

21. Shyamala J.

Consultant Paediatrics, Apollo Hospitals, Chennai, Tamil Nadu

Email: jaymoorthy@hotmail.com

22. Simmi K. Ratan

Professor, Department of Paediatric Surgery, Maulana Azad Medical College, Delhi, India

Email: drjohnsimmi@yahoo.com

23. Suman Sarkar

Assistant Professor, Department of Paediatrics, Institute of Post Graduate Medical Education and Research, Kolkata, West Bengal, India

Email: dr.sumansarkar@gmail.com

24. Vijayendra Kumar

Professor, Department of Paediatric Surgery, Indira Gandhi Institute of Medical Sciences, Patna, Bihar, India

Email: drvijayendrakr@rediffmail.com

25. Narendra Behera

Professor, Department of Pediatrics, MKCG Medical College, Berhampur, Odisha, India

Email: naribehera@yahoo.co.in

26. Rajamani G.

Professor, Department of Paediatric Surgery, Coimbatore Medical College, Coimbatore, Tamil Nadu, India

E mail: drgrajamani@gmail.com

27. Anand P. Dubey

Professor, Department of Paediatrics, Maulana Azad Medical College, Delhi, India

Email: Anand_dubey52@hotmail.com

For peer review only

28. Atul Gupta

Consultant Paediatric Surgery, Fortis Escorts Hospital, Jaipur, Rajasthan, India

Email: atulgupta26878@gmail.com

29. Cenita J. Sam

Professor, Department of Paediatric Surgery, PSG Institute of Medical Sciences, Coimbatore, Tamil Nadu, India

Email: cenitasivamani@yahoo.com

30. Gowhar Nazir Mufti

Assistant Professor, Department of Paediatric Surgery, Sher-I-Kashmir Institute of Medical Sciences, Srinagar, Jammu & Kashmir, India

Email: gowharmufti@yahoo.co.in

31. Harsh Trivedi

Professor, Department of Paediatric Surgery, MP Shah Government Medical College, Jamnagar, Gujarat, India

Email: trivediharsh@indiatimes.com

32. Jimmy Shad

Consultant Paediatric Surgery, Apollo Hospitals, Chennai, Tamil Nadu, India

Email: shadjimmy@yahoo.co.in

33. Kaushik Lahiri

Consultant, Department of Paediatric Surgery, Gauhati Medical College, Guwahati, Assam, India

Email: Kaushik_1671979@rediffmail.com

34. Krishnaswamy R

Chief Medical Officer, Masonic Children's Hospital, Coimbatore, Tamil Nadu, India

Email: krishnaswamy24@yahoo.com

For peer review only

For peer review only - http://bmjopen.bmj.com/site/about/guidelines.xhtml

35. Meera Luthra

Consultant Paediatric Surgery, Medanta- The Medicity, Gurgaon, Haryana, India

Email: meera.luthra@medanta.org

36. Padmalatha P

Professor, Department of Paediatrics, Andhra Medical College, Vishakhapatnam, Andhra Pradesh, India

Email: padmap_2000@yahoo.com

37. Rakesh Kumar

Associate Professor, Department of Paediatrics, Indira Gandhi Institute of Medical Sciences, Patna, Bihar, India

Email: drjaiswalrakesh@gmail.com

38. Ruchirendu Sarkar

Professor, Department of Paediatric Surgery, Institute of Post Graduate Medical Education and Research, Kolkata, West Bengal, India

Email: ruchirendu@gmail.com

39. Santosh Kumar A.

Professor, Department of Paediatrics, Government Medical College & SAT Hospital, Thiruvananthapuram, Kerala, India

Email: hod.pediatrics.sath@gmail.com

40. Subrat Kumar Sahoo

Associate Professor, Department of Paediatric Surgery, IMS & SUM Medical College & Hospital, Bhubaneswar, Odisha, India

Email: dr_subratsahoo@yahoo.com

41. Sunil K. Ghosh

For peer review only - http://bmjopen.bmj.com/site/about/guidelines.xhtml

Associate Professor, Department of Pediatric Surgery, Agartala Government Medical College, Agartala, Tripura, India

Email: sunilkrghosh752@gmail.com

42. Sushant Mane

Assistant Professor, Department of Paediatrics, Grant Medical College & JJ Hospital, Mumbai, Maharashtra, India

Email: drsush2006@gmail.com

43. Arun Dash

Professor, Department of Pediatric Surgery, MKCG Medical College, Berhampur, Odisha

Email: dasharun@gmail.com

44. Bashir Ahmad Charoo

Professor, Department of Paediatrics, Sher-I-Kashmir Institute of Medical Sciences, Srinagar, Jammu & Kashmir, India

Email: charoobash@gmail.com

45. Bikasha Bihary Tripathy

Associate Professor, Department of Paediatric Surgery, IMS & SUM Medical College & Hospital, Bhubaneswar, Odisha, India

Email: bbtripathy.dr@gmail.com

46. Rajendra Prasad G.

Professor, Department of Paediatric Surgery, Andhra Medical College, Vishakhapatnam, Andhra Pradesh, India

Email: dr.grajendraprasad@gmail.com

47. Harish Kumar S

Paediatrics Radiologist, Apollo Hospitals, Chennai, Tamil Nadu, India

Email: drharishkumar.s@gmail.com

For peer review only

For peer review only - http://bmjopen.bmj.com/site/about/guidelines.xhtml

48. Jothilakshmi K.

Professor, Department of Paediatrics, PSG Institute of Medical Sciences, Coimbatore, Tamil Nadu, India

Email: drjothi.cbe@gmail.com

49. Nihar Ranjan Sarkar

Associate Professor, Department of Radiology, Institute of Post Graduate Medical Education and Research, Kolkata, West Bengal, India

Email: dr.niharsarkar@gmail.com

50. Pavai Arunachalam

Professor, Department of Paediatric Surgery, PSG Institute of Medical Sciences, Coimbatore, Tamil Nadu, India

Email: pavai4321@yahoo.com

51. Satya Sundar G. Mohapatra

Professor, Department of Radiology, IMS & SUM Medical College & Hospital, Bhubaneswar, Odisha, India

Email: drartifact@gmail.com

52. Saurabh Garge

Consultant Paediatric Surgery, Choithram Hospital and Research Centre, Indore, Madhya Pradesh, India

Email: saurabhgarge8@gmail.com

For peer review only

Abstract

Objectives: The study aimed to document the association between intussusception in Indian children and meteorological parameters and examine the regional variations.

Design: A bidirectional (retrospective and prospective) surveillance included intussusception cases between July 2010 and September 2017.

Setting: At 20 hospitals in India, retrospective case record review during July 2010 and March 2016 and prospective surveillance during April 2016 and September 2017 were performed.

Participants: 2161 children aged 2-24 months with primary intussusception were included.

Interventions: The monthly mean meteorological parameters (temperature, sunshine, rainfall, humidity and wind speed) for the study sites were collected.

Methods: The association between monthly intussusception cases and meteorological parameters were examined at pooled, regional and site levels using Pearson's (r) and Spearman's rank-order (ρ) correlation, factorial analysis-of-variance and Poisson regression or negative binomial regression analyses.

Results: The intussusception cases were highest in summer and lowest in autumn seasons. Pearson's correlation analysis showed that temperature (r=0.056;p<0.05), wind speed (r=0.134;p<0.01) and humidity (r=0.075;p<0.01) were associated with monthly intussusception. Spearman's' rank-order correlation analysis found that temperature (ρ=0.049;p<0.05), wind speed (ρ=0.096;p<0.01) and sunshine (ρ=0.051;p<0.05) were associated with monthly intussusception. Poisson regression analysis resulted that monthly intussusception cases was associated with rising temperature (North region, p<0.01 and East region, p<0.05), sunshine (North region,p<0.01), humidity (East region,p<0.01), and wind speed (East region,p<0.01). Factorial analysis-of-variance revealed significant seasonal difference in intussusception cases for pooled level (p<0.05), 2-6 months age group (p<0.05) and North region (p<0.01). Significant differences in intussusception cases between summer

For peer review only

For peer review only - http://bmjopen.bmj.com/site/about/guidelines.xhtml

and autumn seasons was observed for pooled ($p<0.01$), children aged 2-6 months ($p<0.05$) and 7-12 months ($p<0.05$).

Conclusions: A significant correlation between intussusception and temperature, humidity and wind speed was observed at pooled and regional level in India. A peak in summer months was noted, which may be used for prediction, early detection and referral for appropriate management of intussusception.

Keywords: intussusception, children, climate, meteorological parameters, ambient temperature, season, India

For peer review only

For peer review only - http://bmjopen.bmj.com/site/about/guidelines.xhtml

Strengths and limitations of the study

- This study included intussusception cases in children from 20 sites in India representing different geographic and climate regions.
- Correlation between the meteorological parameters and intussusception was explored using Pearson's correlation and Spearman's rank-order correlation analyses
- Seasonal variation for intussusception across regions was examined using factorial analysis-of-variance and regression analyses.

For peer review only

For peer review only - http://bmjopen.bmj.com/site/about/guidelines.xhtml

1. Introduction

Intussusception is the most common acute abdomen with intestinal obstruction in children, mostly occurring in infants [1,2]. The incidence of intussusception vary widely with mean 74 per 100,000 (range: 9-328) among infants with peak during 5-7 months of age [3]. The available information from India suggest intussusception incidence to vary from 17.7 to 254 per 100,000 child-years [4,5]. These children usually present with abdominal pain or excessive crying, vomiting, bloody stools and may have abdominal mass. Ultrasound is the leading diagnostic tool. Majority of the patients can be managed by reduction method and some may need surgery. In majority of the children, the aetiology of intussusception remain unknown [6].

Intussusception has attracted attention due to its association with the rotavirus vaccine (RVV). India introduced RVV into the universal immunization program (UIP) in 2016 and expanded countrywide in 2019. A recent publication by our group reported regional variation in the intussusception cases among Indian children under-two year age [7]. More number of cases were observed during March to June months (summer months) suggesting seasonal variation. Other studies from India also reported seasonal variation [5,8-11]. There is variation in seasonality of intussusception occurrence globally. Higher number of intussusception cases have been reported during warmer months from some countries [12-16], while no seasonal variation reported from other countries [17-23]. Reports from China found the intussusception case load to be associated with meteorological parameters like temperature, sunshine and humidity [12,24]. In several reports from different countries, the association between meteorological factors and intussusception remains unclear, due to various reasons including shorter study duration, smaller sample size, and no definite statistical methods to explore the association. Although the seasonal variations have been reported, no association between the intussusception and meteorological parameters have been reported from India.

For peer review only - http://bmjopen.bmj.com/site/about/guidelines.xhtml

A multisite surveillance in India documented the epidemiology and trend of intussusception as part of the vaccine safety surveillance linked to the RVV introduction. Using the data collected by the Indian surveillance network, this study investigated the possible association between intussusception cases and various meteorological parameters and seasonality at site, regional and national levels.

2. Methods

2.1. Study area and participating hospitals

This hospital-based sentinel surveillance for intussusception in under-two year children at 20 Indian major tertiary care hospitals included both prospective and retrospective periods. This bidirectional surveillance was comprised of a retrospective (July 1, 2010 to March 31, 2016) and a prospective (April 1, 2016 to September 30, 2017) periods of data collection. The study sites were selected from four regions (north, south, east, and west) of the country ensuring a mix of public and private tertiary care hospitals (North: three public and two private; South: two public and three private; East: five public and one private; West: 2 public, and one private) (Supplementary Figure 1). The study sites represented different climate zones in India. The method of study sites selection have been published in the protocol [25].

2.2. Case definition, case selection and data collection

The children aged 2-24 months admitted to these hospitals with intussusception were eligible. For the retrospective surveillance period, the potential cases were identified using international classification of diseases (ICD 9/10) or diagnoses in medical record section (Supplementary Table 1). These case records were reviewed to identify the intussusception cases and all cases were recruited. For the prospective surveillance period, all age-eligible children were screened,

For peer review only - http://bmjopen.bmj.com/site/about/guidelines.xhtml

tracked till final diagnosis and all intussusception cases were recruited after written informed consent. The cases of first episode of intussusception were identified from the history and/or from parents. For the confirmed cases, data including demography, clinical features, management, and final outcome were abstracted using case record forms. External experts visited the study sites to assess the protocol adherence for case selection, record retrieval and data abstraction. Data team members visited the sites and repeated the case retrieval process for 2014, 2015, and 2016 to assess the retrieval completeness. Data queries were clarified with reference to the source documents.

2.3. Meteorological data collection

For the study sites, meteorological data including monthly mean parameters including temperature (^{0}Centigrade, ^{0}C), sunshine (total hours), rainfall (millimetre, mm), humidity (%) and wind speed (kilometre per hour, Kmph) were collected from the website, World Weather Online (https://www.worldweatheronline.com/). These meteorological data collected for the period July 2010 through September 2017 were available in the public domain.

2.4. Data Management and analysis

The descriptive analysis included proportions, means (with standard deviations), and median (with interquartile range, IQR). There was no definite population catchment area for the hospitals and the estimation of intussusception incidence was not possible. The calendar months were grouped into seasons (summer, rainy, autumn, winter and spring) for the individual sites and regions (Supplementary Table 2). The Chi-squared goodness of fit test was used to examine the periodicity of intussusception and monthly differences. Kruskal-Wallis H test (χ^2) was performed to document the differences between >3 groups (years, months seasons and temperature ranges: <20°C, 21°C-25°C, 26°C-30°C and >30°C). Pearson's correlation (r)

For peer review only - http://bmjopen.bmj.com/site/about/guidelines.xhtml

analysis explored the association between monthly intussusception cases (dependent variable) and monthly mean meteorological parameters separately (independent variables). Spearman's rank-order correlation (ρ) analysis was also conducted to explore whether the monthly intussusception and monthly mean meteorological parameters covary. Factorial analysis-of-variance (F) was used to analyse the variations in the intussusception cases during the different seasons (dependent variable: seasonal intussusception cases and independent variable: season and year). Poisson regression or negative binomial regression (β (for parameters with wide dispersion) analyses was performed to evaluate the relationships between the monthly intussusception cases (dependent variable) and the monthly mean meteorological parameters (independent variables). The association between intussusception and meteorological parameters were examined at pooled, regional and site levels with 95% confidence interval (CI) estimation. Statistical significance was considered if $p<0.05$. Statistical analysis was performed using STATA version 15.0 (Stata Corp LLC, Texas, USA).

2.5. Ethical issues

The study protocol was reviewed and approved by all the ethics review committees of each participating institute. Confidentiality in data handling was maintained. The participants under prospective component were recruited after written informed consent from parent/legal guardian.

2.6. Patient and Public Involvement

The study participants or the public were not involved in the design, or conduct, or reporting, or dissemination plans of our research. The findings would be relevant for care seeking and case management for intussusception in children. The results published shall be disseminated to the public through social media and through healthcare providers.

For peer review only - http://bmjopen.bmj.com/site/about/guidelines.xhtml

3. Results

3.1 Demography data

During July 2010 through September 2017, 2279 children aged 2-24 months with intussusception were recruited. Out of these, 118 children had past history of intussusception, and were excluded. Thus, 2161 primary intussusception cases were analysed. The demography and regional distribution of the intussusception cases across the years is shown in Table 1. There were 1431 (66.2%) boys and 730 (33.8%) girls (male-female ratio, 1.96). Majority (n=1653, 76.5%) were infants. The number of cases increased across the years with highest (31%) jump between 2014 and 2015. During 2010-2014, the children aged 7-12 months had a higher share, which changed during 2015-2017. The South region contributed the highest (50%), followed by East (27.4%), North (17.1%) and West (5.4%) regions, which were consistent across the years.

3.2. Meteorological data

The monthly meteorological parameters for the regions are shown in Supplementary Table 3. The monthly meteorological parameters for each site is given in Supplementary Table 4. Across the regions, April to June and December-February had the highest and lowest temperatures respectively. The pattern of meteorological parameters across the regions is shown in Supplementary Figure 2.

3.3. Monthly and seasonal cycles of intussusception

The Chi-squared goodness of fit test revealed obvious monthly (χ^2-statistic= 71.36; $p<0.01$) and seasonal differences (χ^2-statistic= 382.28; $p<0.01$) in the intussusception case load. Also Kruskal-Wallis H test revealed significant differences in the intussusception cases for months ($p<0.01$) and seasons ($p<0.01$). As shown in Figure 1, more intussusception cases were

For peer review only

For peer review only - http://bmjopen.bmj.com/site/about/guidelines.xhtml

observed during March in South region, June in North and East regions, all during summer season. Not much variation in cases was observed in West region. The intussusception cases were lowest in September and October months during these years. The peak and lowest seasons were summer (March-June; n=661, 30.6% cases) and autumn (September-October; 14.5% cases), respectively (Supplementary Figure 3).

3.4. Correlation between the intussusception and meteorological factors

Table 2 shows the Pearson's correlation analysis for pooled, regions and age groups. For pooled data, the temperature (r=0.056; $p<0.05$), wind speed (r=0.134; $p<0.01$) and humidity (r=0.075; $p<0.01$) had significant association with intussusception. At regional level, intussusception cases had association with temperature for East region (r=0.052; $p<0.05$); with rainfall for North (r=-0.103; $p<0.01$) and East regions (r=0.073; $p<0.01$); with wind speed for all regions (r=-0.084 to 0.21; $p<0.01$); with humidity for three regions except West region (r=-0.373 to 0.161; $p<0.01$); and with sunshine for North region (r=0.163; $p<0.01$). For the age groups, intussusception cases had association with temperature for 7-12 months (r=0.068; $p<0.01$), with wind speed for all age groups (r=-0.092 to 0.125; $p<0.01$), with humidity for 7-12 months (r=0.069; $p<0.01$) and 13-24 months (r=0.076; $p<0.01$) age groups. The Pearson correlation analysis for intussusception cases with the meteorological parameters for the study sites are given in Supplementary Table 5. Significant correlations for intussusception cases with temperature were observed for three sites, with rainfall for two sites, with wind speed and humidity for five sites each and with sunshine for three sites.

Table 3 shows the Spearman's' rank-order correlation analysis for pooled, regions and age groups. For pooled data, the temperature (ρ=0.049; $p<0.05$), wind speed (ρ=0.096; $p<0.01$) and sunshine (ρ=0.051; $p<0.05$) were associated with intussusception. At regional level,

For peer review only

intussusception cases had association with temperature for North region (ρ=0.110; p<0.05); with wind speed for North (ρ=0.183; p<0.01) and East (ρ=0.109; p<0.01) regions; with humidity for North (ρ=-0.114; p<0.05) and East (ρ=-0.102; p<0.05) regions; and with sunshine for North region ((ρ=0.119; p<0.05). For the age groups, intussusception cases had association with temperature for 7-12 months ((ρ=0.064; p<0.01), with wind speed for all age groups ((ρ=0.063 to 0.106; p<0.01), with humidity (ρ=0.049; p<0.05) and sunshine (ρ=0.048; p<0.05) for 7-12 months age group. The Spearman's' Rank correlation analysis for intussusception cases with the meteorological parameters for the study sites are given in Supplementary Table 6. Significant correlations for intussusception cases with temperature were observed for one site, with rainfall for two sites, with wind speed for three sites, with humidity for five sites and with sunshine for two sites.

Regression analyses (Poisson regression and negative binomial regression) revealed significant association between intussusception cases and temperature for pooled level (β=0.026; 95% CI 0.011, 0.041; p<0.01) North (β=0.027; 95% CI 0.007, 0.047; p<0.01) and East (β=0.029; 95% CI 0.001, 0.057; p<0.05) regions; wind speed for pooled level (β=0.082; 95% CI 0.061, 0.103; p<0.01), North (β=0.208; 95% CI 0.101, 0.315; p<0.01) and East (β=0.091; 95% CI 0.042, 0.141; p<0.01) regions; humidity for pooled level (β=0.011; 95% CI 0.007, 0.016; p<0.01), East (β=-0.021; 95% CI -0.035, -0.007; p<0.01) region and sunshine for pooled level (β=0.002; 95% CI 0, 0.003; p<0.01), North (β=0.004; 95% CI 0.002, 0.007; p<0.01) region (Table 4). Associations between the intussusception cases and temperature and humidity were evident across different age groups (p<0.01). The association was also significant at study sites level for temperature (three sites), humidity (six sites) and sunshine (five sites) (Supplementary Table 7).

For peer review only

For peer review only - http://bmjopen.bmj.com/site/about/guidelines.xhtml

The monthly average temperatures at the sites and regions varied widely; 14°C to 35°C in North, 23°C to 31°C in South, 20°C to 32°C in East and 22°C to 31°C in West regions. Chi-squared goodness of fit test showed significant difference at 20°C–30°C for pooled, North and South regions ($p<0.01$), indicating that the intussusception cases occurred more during this temperature (Supplementary Table 8/13). Kruskal-Wallis H test found significant variation in the intussusception cases between the different temperature categories for pooled ($\chi^2=15.933$; $p<0.01$), North ($\chi^2=21.054$; $p<0.01$) and South ($\chi^2=50.625$; $p<0.01$) regions (Supplementary Table 9/14). The number of intussusception cases increased when the mean daily temperature and wind speed increased and mean daily humidity decreased at pooled level and for North and East regions (Supplementary Figure 4-8).

3.5. Correlation between the intussusception, seasons and age groups

Factorial analysis-of-variance revealed significant difference in seasonal intussusception cases at pooled level ($F=1.442$; $p<0.05$), for 2-6 months age group ($F=1.579$; $p<0.05$) and for North region ($F=1.809$; $p<0.01$) (Supplementary Table 10/8 and 11/9). Multiple comparison revealed significant difference in intussusception cases between summer and autumn seasons (mean difference=0.569; 95% CI 0.11-1.03; $p<0.01$) at pooled level (Supplementary Table 12/10). Significant differences intussusception cases between summer and monsoon (mean difference=0.165; 95% CI 0.04-0.29, $p<0.01$), summer and autumn (mean difference=0.179; 95% CI 0.03-0.32, $p<0.01$), spring and monsoon (mean difference=0.276; 95% CI 0.09-0.46, $p<0.01$) and spring and autumn (mean difference=0.290; 95% CI 0.09-0.49, $p<0.01$) were observed for North region (Supplementary Table 13/11). Significant differences intussusception cases were observed between summer and autumn for children aged 2-6 months (mean difference=0.195; 95% CI 0.01-0.38, $p<0.05$) and for children aged 7-12 months (mean difference=0.231; 95% CI 0.01-0.45, $p<0.05$) (Supplementary Table 14/12).

For peer review only - http://bmjopen.bmj.com/site/about/guidelines.xhtml

4. Discussion

The seasonal influence on intussusception cases has been proposed, but with inconsistent findings from different countries and different parts of India. This study is the first to explore association between intussusception and monthly mean meteorological parameters (temperature, rainfall, wind speed, humidity and sunshine) in India. This study comprising of 2161 children with primary intussusception from 20 sites in India documented monthly and seasonal variations. Intussusception case peaks were observed during summer months (Mar-June) and troughs during autumn months (September-October). There were positive correlation between intussusception cases and meteorological parameters like temperature, humidity, wind speed and sunshine at pooled and regional levels and among children aged 7-12 months. The associations were consistent across analysis tests used (Pearson correlation, Spearman's Rank correlation tests, Poisson regression or negative binomial regression). The association was more pronounced for North and East regions, where the meteorological parameters fluctuation across the months were widest. Further analysis using factorial analysis-of-variance also demonstrated seasonal inclination with peaking in summer season (Mar-June) in India and significant difference from autumn season (with lowest cases) ($p<0.01$). A significant difference in number of intussusception cases between the temperature ranges (<20°C, 21°C -25°C, 26°C -30°C and >30°C) was observed at pooled and regional (North and South regions) levels, which indicated rise in intussusception cases with rise in monthly mean temperature. The intussusception cases increased with rise in daily temperature and wind speed and fall in humidity.

For peer review only - http://bmjopen.bmj.com/site/about/guidelines.xhtml

479 The findings are consistent with the report from China in 5994 children (aged 0-12 years) with intussusception, where more cases were documented in summer months with significant positive association with mean monthly meteorological parameters (temperature, sunshine, and precipitation) [12]. A modelling using the data from 13887 Chinese children with intussusception and applying the meteorological variation assumptions achieved considerable agreement with the data collected [24]. These reports from China didn't include humidity as a variable.

487 Studies on intussusception in children from India have reported higher cases during summer (March-July) and lowest during autumn (September-November) seasons. In three studies from South India, highest and lowest intussusception cases were observed during March-June and September-October respectively [5,9,10]. A multisite study from India reported highest and lowest cases during March and September months respectively [8]. A study from North India observed highest and lowest cases during June and September months [11]. A report from our group on the retrospective surveillance observed higher number of intussusception cases during summer months (March-June) over the year [7].

496 Seasonal pattern of intussusception occurrence have been observed from different countries. Higher number of intussusception cases were observed during warmer months (May-October) than cooler months (November-April) in Taiwan [14]. Among Hong Kong children with intussusception, higher cases were observed during summer months (May-July) [26]. Among Ethiopian children, higher intussusception cases were observed during June month and lower during November months [13]. Higher intussusception cases were observed during summer months and lower during winter months among South African children [15]. Among Israeli children, more intussusception cases were observed during summer months and lower during

For peer review only - http://bmjopen.bmj.com/site/about/guidelines.xhtml

winter [16]. No seasonal variation was also observed in some studies from different countries. Studies from Latin American countries and North America didn’t observe visible seasonal variation in cases [17,23]. Studies from Switzerland, France, New Zealand, Singapore and China also had no sizable seasonal variations [18-22].

The inconsistencies in the seasonal variations may be due to the sample size, age groups included and stratification in analysis. Three studies which observed definite seasonal variation [12,14,24] and two observed statistically significant association between the meteorological parameters and intussusception has larger sample size (>5000 participants). The association observed in our study may be also due to higher sample size. The reasons for the higher intussusception cases during summer is not known and the seasonality don’t match with the rotavirus infection seasonality in India [27]. According to the reports from some developed countries (United States and Australia), the intussusception incidence have declined over decades and also the prevalence of prodrome infection symptoms (gastrointestinal and respiratory infections) in these children over the same period [28-30]. Further studies are needed to confirm the seasonality, association with the meteorological parameters and variations in seasonality across the regions in India along with the influences of the preventive healthcare services coverage, care seeking practices and access to the sanitation, hygiene and changes in dietary practices.

A seasonal variation in another acute intestinal condition, acute appendicitis in children have been reported. More acute appendicitis cases in children during summer months and lower during spring/autumn seasons have been reported from India, Pakistan, Iraq, China, Taiwan, Italy, Germany, Canada and United States [31-38]. Among Chinese children, association between acute appendicitis and temperature ($p<0.01$), humidity ($p<0.01$) and sunshine

For peer review only

For peer review only - http://bmjopen.bmj.com/site/about/guidelines.xhtml

(p<0.01) were documented to support the seasonal variation [34]. Similar to the intussusception, the appendicitis incidence has also declined in developed countries over decades, possible due to changes in the hygiene, sanitation, dietary practices, access to healthcare and reduction in the infectious diseases [39]. The intussusception and acute appendicitis, both are intestinal problems and some of the intussusception have associated appendicitis. The climate factors are unlikely to directly cause intussusception or intussusception in children. But the linkage between seasonality and meteorological parameters and acute intestinal conditions appears to be plausible, which may mediated by intestinal and non-intestinal infections (including activation of latent viral infections), mucosal inflammatory changes, lymphoid hyperplasia, or motility alterations and dietary intake changes. Although no direct relationship with the rotavirus infection could be established, higher gastrointestinal infection during summer and early rainy seasons may be triggers for higher intussusception in Indian context. The changes in intussusception and appendicitis incidence over time in parallel with the industrialization, economic improvement, improved access to vaccination, healthcare and sanitation and hygiene practices suggest possible association. Such information in Indian and developing country context is not available. Thus the seasonal variation in these conditions and association between meteorological parameters appear epidemiologically and biologically plausible, which needs further evaluation. We used Pearson's correlation coefficient as the primary analysis to document the strength and direction of relationship between the meteorological parameters and intussusception. As secondary analysis and checking for the consistency in the findings, we used Spearman's rank-order correlation analysis to identify strength of association between ranked parameters and intussusception variables.

The Pearson's correlation coefficient (r) and Spearman's correlation coefficient (ρ) values for different meteorological parameters were similar, although the r values for temperature,

For peer review only

For peer review only - http://bmjopen.bmj.com/site/about/guidelines.xhtml

rainfall, wind speed and humidity were higher than the ρ values, but had r value was lower for sunshine than the ρ value at pooled level. The differences between the r and ρ values were relatively small, which comforts and supports the consistent association between the variables. The consistency in findings from factorial analysis-of-variance and regression analysis with the correlation coefficients further confirms the association between the meteorological parameters and intussusception.

The study had several strengths; large number of intussusception cases over seven years period, sites from different regions, and statistical methods used and consistency in the findings. The consistency across the results using different statistical tests was assuring. There are several limitations in the study. A major part of this study was retrospective. The sites belong to different climate zones and had variation in the meteorological parameters. Lack of definite catchment population and referral pattern, made it difficult to estimate the intussusception incidence rate.

In conclusion, this study documented higher intussusception cases during the summer months with a positive association between number of cases with temperature and sunshine. Also significant positive correlation with the humidity and wind speed was observed. The association was significant for the North and East regions with wider variations in the meteorological parameters across seasons. Higher degree of suspicion for infants with compatible clinical presentation for intussusception during the summer months may improve diagnosis and clinical care to prevent the complications. The study provides evidence for predicting the intussusception pattern in children and sensitizing the healthcare providers at community and peripheral facilities for early detection and quick referral of patients to minimise the surgical interventions and avoid fatalities. Further studies are needed to confirm

For peer review only

the observation and improve the evidence base on the potential risk factors for the season variations and the aetiologies of intussusception.

For peer review only - http://bmjopen.bmj.com/site/about/guidelines.xhtml

Acknowledgments

We acknowledge the support from Ministry of Health and Family Welfare, Government of India for undertaking the study. We are thankful to the hospital administrations and the clinicians at the study site institutes, who supported and facilitated undertaking of the study.

We highly value the technical guidance and inputs provided by the members of Technical Advisory Group: Mahesh K Agarwal and Pradeep Haldar, Ministry of Health and Family Welfare, Government of India; Satinder Aneja, Anju Seth and Archana Puri, Lady Hardinge Medical College, New Delhi; Ashok Patwari, Hamdard Institute of Medical Sciences & Research, New Delhi; Yogesh Kumar Sarin, Maulana Azad Medical College, New Delhi; Rakesh Aggarwal, Anshu Srivastava and Ujjal Poddar, Sanjay Gandhi Postgraduate Institute of Medical Sciences, Lucknow; Malathi Satyasekharan, Kanchi Kamakoti Chailds Trust Hospital, Chennai; Raju Sharma and Nirupam Madan, All India Institute of Medical Sciences, New Delhi; Jyoti Joshi and Deepak Polpakara, Immunization Technical Support Unit; Ministry of Health & Family Welfare, New Delhi; Umesh D. Parashar; Centers of Disease Control and Prevention, Atlanta, USA; Jan Bonhoeffer, University Children's Hospital, Basel, Switzerland and Brighton Collaboration; Naveen Thacker, Child Health Foundation, Gandhigram; Rashmi Arora, Indian Council of Medical Research, Ansari Nagar, New Delhi; and Patrick L F Zuber and Christine G. Maure, World Health Organisation, Geneva, Switzerland.

We acknowledge the contribution of the research staffs at The INCLEN Trust International: Harshpreet Kaur, Janvi Chaubey, Mrimmaya Das, Shweta Sharma and Vaibhav Jain.

We highly appreciate the efforts made by the research staffs at the study sites: Aarezo Bashir and Rafia; Sher-e-Kashmir Institute of Medical Sciences, Srinagar, Jammu & Kashmir; Prabha Shankar, Medanta-The Medicity Hospital, Gurgaon, Haryana; Anju Sharma; Maulana Azad Medical College, New Delhi; Anita Singh and Shubhanshu Srivastava, King George Medical University, Lucknow, Uttar Pradesh; Hemant Meena, Choithram Hospital, Indore, Madhya

For peer review only

Pradesh; Pankaj Kumar and Shashi Kant; Indira Gandhi Institute of Medical Sciences, Patna, Bihar; Goutam Benia, IMS & SUM Medical College & Hospital, Bhubaneshwar, Odisha; Prasntajyoti Mohanty, SVP Post Graduate Institute of Paediatrics, Cuttack, Odisha; Asit Pradhan, MKCG Medical College, Berhampur, Odisha; Angshuman Chatterjee, Institute of Postgraduate Medical Education and Research & SSKM Hospital, Kolkata, West Bengal; S. Yamuna, Andhra Medical College, Vishakhapatnam, Andhra Pradesh; Srinidhi Sudan, Apollo Hospitals, Hyderabad, Telengana; Rajesh Francis, Apollo Hospitals, Chennai, Tamil Nadu; T. Easter Chandru, PSG Institute of Medical Sciences, Coimbatore, Tamil Nadu; Deepthy R, Julie and Anju Shivakumar, Government Medical College & SAT Hospital, Thiruvananthapuram, Kerala; Archit Vaidya, Grant Medical College & JJ Hospital, Mumbai, Maharashtra; Nimesh Chouksey, MP Shah Government Medical College, Jamnagar, Gujarat; Nidhi Singh, Fortis Escorts Hospital, Jaipur, Rajasthan; Mrinmoy Gohain, Gauhati Medical College, Guwahati, Assam; Arpita Bhattachrjee, Saugat Ghosh and Tanusmita Debnath, Agartala Government Medical College, Agartala, Tripura.

For peer review only

For peer review only - http://bmjopen.bmj.com/site/about/guidelines.xhtml

Declarations

Ethics approval and consent to participate: The study protocol was reviewed and approved by the ethics committees of all the participating institutes (Supplementary document 2). The interviews with stakeholders were done after obtaining written informed consent.

Consent for publication: Not applicable

Availability of data and materials: All data is available with the investigators and can be provided by the corresponding author upon reasonable request.

Competing interests: The authors declare that there is no competing interests and conflict of interest.

Disclosure statement: None. There is no financial interest or benefit for the authors arisen from this project or its direct application.

Funding: This project was supported by the Bill and Melinda Gates Foundation, USA to The INCLEN Trust International (grant number OPP1116433). The funder or its representative had no role in the design of the study and collection, analysis, and interpretation of data and writing the manuscript.

Authors' contributions:

Study conceptualisation, study design, protocol development, training, data collection, data cleaning, data analysis, interpretation, coordination, monitoring, quality assurance, manuscript writing: The INCLEN Intussusception Surveillance Network Study Group.

All authors reviewed, provided critical input and approved the final version.

For peer review only

List of abbreviations

CI	Confidence interval
ICD	International Classification of Diseases
IQR	Interquartile range
RVV	Rotavirus vaccine
UIP	Universal immunization programme

For peer review only

For peer review only - http://bmjopen.bmj.com/site/about/guidelines.xhtml

References

1. Bines JE, Kohl KS, Forster J, Zanardi LR, Davis RL, Hansen J, et al. Acute intussusception in infants and children as an adverse event following immunization: case definition and guidelines of data collection, analysis, and presentation. Vaccine. 2004 Jan;22(5–6):569–74.
2. Bines JE, Ivanoff B. Acute intussusception in infants and children: Incidence, clinical presentation and management: a global perspective [Internet]. World Health Organisation; 2002 [cited 2017 May 10]. Available from: http://apps.who.int/iris/bitstream/handle/10665/67720/WHO_V-B_02.19_eng.pdf;jsessionid=74671C3CD99ED55137202F8D3D07CB73?sequence=1
3. Jiang J, Jiang B, Parashar U, Nguyen T, Bines J, Patel MM. Childhood Intussusception: A Literature Review. Cameron DW, editor. PLoS ONE. 2013 Jul 22;8(7):e68482.
4. Bahl R, Saxena M, Bhandari N, Taneja S, Mathur M, Parashar UD, et al. Population-Based Incidence of Intussusception and a Case-Control Study to Examine the Association of Intussusception with Natural Rotavirus Infection among Indian Children. The Journal of Infectious Diseases. 2009 Nov;200(s1):S277–81.
5. Jehangir S, John J, Rajkumar S, Mani B, Srinivasan R, Kang G. Intussusception in southern India: Comparison of retrospective analysis and active surveillance. Vaccine. 2014 Aug;32:A99–103.
6. Jain S, Haydel MJ. Child Intussusception. In: StatPearls [Internet]. Treasure Island (FL): StatPearls Publishing; 2020 [cited 2020 Jul 19]. Available from: http://www.ncbi.nlm.nih.gov/books/NBK431078/
7. Das MK, Arora NK, Gupta B, Sharan A, Kameswari K, Padmalatha P, et al. Intussusception in children aged under two years in India: Retrospective surveillance at nineteen tertiary care hospitals. Vaccine. 2020 Jun;S0264410X20305636.

For peer review only - http://bmjopen.bmj.com/site/about/guidelines.xhtml

8. Singh JV, Kamath V, Shetty R, Kumar V, Prasad R, Saluja T, et al. Retrospective surveillance for intussusception in children aged less than five years at two tertiary care centers in India. Vaccine. 2014 Aug;32:A95–8.

9. Bhowmick K, Kang G, Bose A, Chacko J, Boudville I, Datta SK, et al. Retrospective surveillance for intussusception in children aged less than five years in a South Indian tertiary-care hospital. J Health Popul Nutr. 2009 Oct;27(5):660–5.

10. Srinivasan R, Girish Kumar CP, Naaraayan SA, Jehangir S, Thangaraj JWV, Venkatasubramanian S, et al. Intussusception hospitalizations before rotavirus vaccine introduction: Retrospective data from two referral hospitals in Tamil Nadu, India. Vaccine. 2018 Dec;36(51):7820–5.

11. Gupta M, Kanojia R, Singha R, Tripathy JP, Mahajan K, Saxena A, et al. Intussusception Rate Among Under-Five-Children Before Introduction of Rotavirus Vaccine in North India. Journal of Tropical Pediatrics. 2018 Aug 1;64(4):326–35.

12. Guo W, Zhang S, Li J, Wang J. Association of Meteorological Factors with Pediatric Intussusception in Subtropical China: A 5-Year Analysis. Wang L, editor. PLoS ONE. 2014 Feb 28;9(2):e90521.

13. Gadisa A, Tadesse A, Hailemariam B. Patterns and seasonal variation of intussusception in children: a retrospective analysis of cases operated in a tertiary hospital in Ethiopia. Ethiop Med J. 2016 Jan;54(1):9–15.

14. Chen SC-C, Wang J-D, Hsu H-Y, Leong M-M, Tok T-S, Chin Y-Y. Epidemiology of childhood intussusception and determinants of recurrence and operation: analysis of national health insurance data between 1998 and 2007 in Taiwan. Pediatr Neonatol. 2010 Oct;51(5):285–91.

For peer review only

For peer review only - http://bmjopen.bmj.com/site/about/guidelines.xhtml

15. Moore SW, Kirsten M, Müller EW, Numanoglu A, Chitnis M, Le Grange E, et al. Retrospective surveillance of intussusception in South Africa, 1998-2003. J Infect Dis. 2010 Sep 1;202 Suppl:S156-161.

16. Greenberg D, Givon-Lavi N, Newman N, Wheeler J, Cohen Z, Dagan R. Intussusception in Children in Southern Israel: Disparity between 2 Populations. The Pediatric Infectious Disease Journal. 2008 Mar;27(3):236–40.

17. Sáez-Llorens X, Velázquez FR, Lopez P, Espinoza F, Linhares AC, Abate H, et al. A multi-country study of intussusception in children under 2 years of age in Latin America: analysis of prospective surveillance data. BMC Gastroenterol. 2013 May 27;13:95.

18. Buettcher M, Baer G, Bonhoeffer J, Schaad UB, Heininger U. Three-year surveillance of intussusception in children in Switzerland. Pediatrics. 2007 Sep;120(3):473–80.

19. Serayssol C, Abbo O, Mouttalib S, Claudet I, Labarre D, Galinier P, et al. [Seasonal pattern of intussusceptions in infants and children: is fall/winter predominance still worth consideration? A 10-year retrospective epidemiological study]. Arch Pediatr. 2014 May;21(5):476–82.

20. Chen YE, Beasley S, Grimwood K, and the New Zealand Rotavirus Study Group. Intussusception and rotavirus associated hospitalisation in New Zealand. Archives of Disease in Childhood. 2005 Jun 7;90(10):1077–81.

21. Boudville IC, Phua KB, Quak SH, Lee BW, Han HH, Verstraeten T, et al. The epidemiology of paediatric intussusception in Singapore: 1997 to 2004. Ann Acad Med Singap. 2006 Oct;35(10):674–9.

22. Liu N, Yen C, Huang T, Cui P, Tate JE, Jiang B, et al. Incidence and epidemiology of intussusception among children under 2 years of age in Chenzhou and Kaifeng, China, 2009-2013. Vaccine. 2018 14;36(51):7862–7.

For peer review only

For peer review only - http://bmjopen.bmj.com/site/about/guidelines.xhtml

23. Chang HG, Smith PF, Ackelsberg J, Morse DL, Glass RI. Intussusception, rotavirus diarrhea, and rotavirus vaccine use among children in New York state. Pediatrics. 2001 Jul;108(1):54–60.

24. Guo W, Geng J, Zhan Y, Tan Y, Hu Z, Pan P, et al. Forecasting and predicting intussusception in children younger than 48 months in Suzhou using a seasonal autoregressive integrated moving average model. BMJ Open. 2019 Jan;9(1):e024712.

25. Das M, Arora N, Bonhoeffer J, Zuber P, Maure C. Intussusception in Young Children: Protocol for Multisite Hospital Sentinel Surveillance in India. Methods and Protocols. 2018 Mar 22;1(2):11.

26. Nelson EAS, Tam JS, Glass RI, Parashar UD, Fok TF. Incidence of rotavirus diarrhoea and intussusception in Hong Kong using standardized hospital discharge data. The Pediatric Infectious Disease Journal. 2002 Jul;21(7):701–3.

27. Giri S, Nair NP, Mathew A, Manohar B, Simon A, Singh T, et al. Rotavirus gastroenteritis in Indian children < 5 years hospitalized for diarrhoea, 2012 to 2016. BMC Public Health. 2019 Dec;19(1):69.

28. Parashar UD, Holman RC, Cummings KC, Staggs NW, Curns AT, Zimmerman CM, et al. Trends in Intussusception-Associated Hospitalizations and Deaths Among US Infants. Pediatrics. 2000 Dec 1;106(6):1413–21.

29. Desai R, Curns AT, Patel MM, Parashar UD. Trends in intussusception-associated deaths among US infants from 1979-2007. J Pediatr. 2012 Mar;160(3):456–60.

30. Justice FA, Auldist AW, Bines JE. Intussusception: trends in clinical presentation and management. J Gastroenterol Hepatol. 2006 May;21(5):842–6.

31. Jangra B, Jangra M, Rattan K, Kadian Y. Seasonal and day of week variations in acute appendicitis in north Indian children. J Indian Assoc Pediatr Surg. 2013;18(1):42.

For peer review only - http://bmjopen.bmj.com/site/about/guidelines.xhtml

32. Ahmed W, Akhtar MS, Khan S. Seasonal variation of acute appendicitis. Pak J Med Sci [Internet]. 2018 May 24 [cited 2020 Aug 4];34(3). Available from: http://pjms.com.pk/index.php/pjms/article/view/14793
33. Al-Mendalawi M, Lattuf M. Is there a seasonal distribution of acute appendicitis in Iraqi children? Menoufia Med J. 2017;30(2):572.
34. Zhang Y, Lyu F, Kang Q, Dong Z, Zhao T, Xie S, et al. Association of meteorological factors with pediatric acute appendicitis in China: A 7-year retrospective analysis. Medicine. 2018 Oct;97(42):e12913.
35. Hsu Y-J, Fu Y-W, Chin T. Seasonal variations in the occurrence of acute appendicitis and their relationship with the presence of fecaliths in children. BMC Pediatr. 2019 Dec;19(1):443.
36. Gallerani M, Boari B, Anania G, Cavallesco G, Manfredini R. Seasonal variation in onset of acute appendicitis. Clin Ter. 2006 Apr;157(2):123–7.
37. Reinisch A, Heil J, Woeste G, Bechstein W, Liese J. The meteorological influence on seasonal alterations in the course of acute appendicitis. Journal of Surgical Research. 2017 Sep;217:137–43.
38. Fares A. Summer appendicitis. Ann Med Health Sci Res. 2014;4(1):18.
39. Ferris M, Quan S, Kaplan BS, Molodecky N, Ball CG, Chernoff GW, et al. The Global Incidence of Appendicitis: A Systematic Review of Population-based Studies. Ann Surg. 2017;266(2):237–41.

For peer review only

For peer review only - http://bmjopen.bmj.com/site/about/guidelines.xhtml

Tables

Table 1: Distribution and demography of the children with intussusception in India

Pooled	Years								
Years	2010[a]	2011	2012	2013	2014	2015	2016	2017[b]	Total
Number of cases, n	68	209	244	260	278	365	429	308	2161
Gender									
Male, n (%)	45 (66.2)	142 (67.9)	155 (63.5)	171 (65.8)	194 (69.8)	243 (66.6)	283 (66)	198 (64.3)	1431 (66.2)
Female, n (%)	23 (33.8)	67 (32.1)	89 (36.5)	89 (34.2)	84 (30.2)	122 (33.4)	146 (34)	110 (35.7)	730 (33.8)
Age groups									
2-6 months, n (%)	24 (35.3)	73 (34.9)	89 (36.5)	86 (33.1)	91 (32.7)	134 (36.7)	159 (37.1)	122 (39.6)	778 (36)
7-12 months, n (%)	33 (48.5)	92 (44)	108 (44.3)	122 (46.9)	113 (40.6)	135 (37)	155 (36.1)	117 (38)	875 (40.5)
13-24 months, n (%)	11 (16.2)	44 (21.1)	47 (19.3)	52 (20)	74 (26.6)	96 (26.3)	115 (26.8)	69 (22.4)	508 (23.5)
Regions									
North, n (%)	9 (13.2)	19 (9.1)	37 (15.2)	44 (16.9)	48 (17.3)	61 (16.7)	91 (21.2)	61 (19.8)	370 (17.1)
South, n (%)	50 (73.5)	134 (64.1)	130 (53.2)	149 (57.3)	124 (44.6)	175 (47.9)	183 (42.7)	136 (44.2)	1081 (50)
East, n (%)	9 (13.2)	45 (21.5)	61 (25)	54 (20.8)	84 (30.2)	112 (30.7)	131 (30.5)	97 (31.5)	593 (27.4)
West, n (%)	0 (0)	11 (5.3)	16 (6.6)	13 (5)	22 (7.9)	17 (4.7)	24 (5.6)	14 (4.5)	117 (5.4)

Note: a: The period of data collection was 6 months (July to December)
b: The period of data collection was 9 months (January to September)

For peer review only

768 Table 2. Pearson correlation analysis between monthly intussusception cases and monthly
769 mean meteorological parameters
770

Parameter	Pearson correlation coefficient (r) for meteorological parameters				
	Temperature (°C)	Rainfall (mm)	Wind speed (m/s)	Humidity (%)	Sunshine (hr)
Pooled (n= 2161)	.056*	-.025	.134**	.075**	.045
Regions					
North (n= 370)	.018	-.103**	-.119**	-.373**	.163**
South (n= 1081)	.022	-.023	.210**	.161**	-.018
East (n= 593)	.052*	.072**	-.084**	.109**	.024
West (n= (117)	.046	-.019	.107**	-.029	-.012
Age groups					
2-6 months (n= 778)	.025	-.022	.092**	.040	.037
7-12 months (n= 875)	.068**	-.012	.115**	.069**	.042
13-24 months (n= 508)	.040	-.030	.125**	.076**	.029

771 *Note: Pearson correlation coefficient (r); Significance two tailed*
772 ** Significance level- p value <0.05; ** Significance level- p value <0.01*

For peer review only

For peer review only - http://bmjopen.bmj.com/site/about/guidelines.xhtml

Table 3. Spearman's rank-order correlation analysis between monthly intussusception cases and monthly mean meteorological parameters

Parameter	Spearman's rank-order correlation coefficient (ρ) for meteorological parameters				
	Temperature (°C)	Rainfall (mm)	Wind speed (m/s)	Humidity (%)	Sunshine (hr)
Pooled (n= 2161)	.049*	.012	.096**	.005	.051*
Regions					
North (n= 370)	.110*	-.014	.183**	-.114*	.119*
South (n= 1081)	.028	-.031	-.024	-.027	.036
East (n= 593)	.061	-.042	.109**	-.102*	.038
West (n= (117)	.052	-.044	.000	-.067	.076
Age groups					
2-6 months (n= 778)	.035	.031	.063**	.007	.044
7-12 months (n= 875)	.064**	.036	.103**	.049*	.048*
13-24 months (n= 508)	.028	.019	.106**	.043	.016

Note: Spearman's rank-order correlation coefficient (ρ); Significance two tailed
** Significance level- p value <0.05; ** Significance level- p value <0.01*

For peer review only

For peer review only - http://bmjopen.bmj.com/site/about/guidelines.xhtml

Table 4: Regression analysis of the relationship between the monthly intussusception cases and monthly mean meteorological parameters

Variables	Regression coefficient, β (95% CI)	Standard error, $S_β$	χ2 - value	p value
Pooled#				
Temperature (^{0}C)	0.026 (0.011, 0.041)	0.008	12.098	<0.01
Rainfall (mm)	-0.001 (-0.003, 0)	0.001	2.462	0.117
Wind Speed (Kmph)	0.082 (0.061, 0.103)	0.011	58.449	<0.01
Humidity (%)	0.011 (0.007, 0.016)	0.002	21.903	<0.01
Sunshine (hr)	0.002 (0, 0.003)	0.001	7.000	<0.01
According to regions				
North region#				
Temperature (^{0}C)	0.027 (0.007, 0.047)	0.0102	7.195	<0.01
Rainfall (mm)	0.001 (-0.005, 0.007)	0.0029	0.169	0.681
Wind Speed (Kmph)	0.208 (0.101, 0.315)	0.0545	14.556	<0.01
Humidity (%)	-0.010 (-0.020, 0.001)	0.0052	3.448	0.063
Sunshine (hr)	0.004 (0.002, 0.007)	0.0013	11.006	<0.01
South region#				
Temperature (^{0}C)	0.010 (-0.037, 0.058)	0.0243	0.174	0.676
Rainfall (mm)	-0.004 (-0.009, 0.000)	0.0024	3.251	0.071
Wind Speed (Kmph)	-0.011 (-0.045, 0.023)	0.0173	0.401	0.527
Humidity (%)	-0.033 (-0.070, 0.005)	0.0190	2.949	0.086
Sunshine (hr)	0.002 (-0.001, 0.004)	0.0014	1.213	0.271
East region#				
Temperature (^{0}C)	0.029 (0.001, 0.057)	0.0141	4.245	0.039
Rainfall (mm)	-0.001 (-0.004, 0.001)	0.0014	0.766	0.381
Wind Speed (Kmph)	0.091 (0.042, 0.141)	0.0251	13.244	<0.01
Humidity (%)	-0.021 (-0.035, -0.007)	0.0070	9.188	<0.01
Sunshine (hr)	0.002 (-6.100, 0.005)	0.0012	3.822	0.051
*West region**				
Temperature (^{0}C)	0.052 (-0.02, 0.124)	1.987	0.036	0.159
Rainfall (mm)	-0.002 (-0.006, 0.002)	0.855	0.002	0.355
Wind Speed (Kmph)	0.022 (-0.042, 0.087)	0.463	0.032	0.496
Humidity (%)	-0.002 (-0.016, 0.011)	0.105	0.006	0.746
Sunshine (hr)	0.003 (-0.001, 0.007)	2.429	0.002	0.119
According to age groups				
*2-6 months**				
Temperature (^{0}C)	0.012 (-0.004, 0.028)	.008	2.192	0.139
Rainfall (mm)	-0.001 (-0.003, 0.001)	.001	1.599	0.206
Wind Speed (Kmph)	0.059 (0.037, 0.081)	.011	28.241	<0.01
Humidity (%)	0.006 (0.001, 0.011)	.003	5.276	0.022
Sunshine (hr)	0.002 (0.000, 0.003)	.001	4.615	0.032
7-12 months#				
Temperature (^{0}C)	0.038 (0.018, 0.057)	.010	14.288	<0.01
Rainfall (mm)	-0.001 (-0.003, 0.002)	.001	.447	0.504
Wind Speed (Kmph)	0.08 (0.054, 0.107)	.013	36.037	<0.01

For peer review only

Humidity (%)	0.012 (0.006, 0.018)	.003	14.660	<0.01
Sunshine (hr)	0.002 (0.000, 0.004)	.001	5.144	0.023
*13-24 months**				
Temperature (^{0}C)	0.026 (0.006, 0.047)	.010	6.460	0.011
Rainfall (mm)	-0.002 (-0.005, 0.000)	.001	3.564	0.059
Wind Speed (Kmph)	0.102 (0.077, 0.128)	.013	60.620	<0.01
Humidity (%)	0.017 (0.010, 0.024)	.004	22.599	<0.01
Sunshine (hr)	0.002 (0.000, 0.004)	.001	3.211	0.073

*Note: Tests used for regression analysis: *Poisson Regression; # Negative Binomial Regression*

For peer review only

For peer review only - http://bmjopen.bmj.com/site/about/guidelines.xhtml

782 **Figure**

783 Figure 1: Monthly distribution of primary intussusception cases in children during 2010-2017

For peer review only - http://bmjopen.bmj.com/site/about/guidelines.xhtml

Supplementary Documents

Sl no	Details
1	Supplementary Figure 1: The study sites and their location on India map indicating the study regions
2	Supplementary Table 1: The International Classification of Diseases (ICD) codes for review of the cases from medical records
3	Supplementary Table 2: The seasons and months at the sites according to the regions
4	Supplementary Table 3: The monthly median (and IQR) meteorological data during 2010 and 2017 for regions
5	Supplementary Table 4: Monthly median (and IQR) meteorological data during 2010 and 2017 at the study sites
6	Supplementary Figure 2: The pattern of monthly meteorological parameters across the regions
7	Supplementary Figure 3: The pooled monthly (SF3a) and seasonal (SF3b) distribution of intussusception in children during July 2010 to September 2017
8	Supplementary Table 5. Pearson correlation analysis between monthly intussusception cases and monthly median meteorological parameters for the study sites
9	Supplementary Table 6. Spearman's Rank correlation analysis between monthly intussusception cases and monthly median meteorological parameters for the study sites
10	Supplementary Table 7. Regression analysis of the association between the intussusception cases and meteorological parameters for the study sites
11	Supplementary Table 8. Intussusception cases in children at different temperature ranges at pooled and regional level
12	Supplementary Table 9. Variations in the intussusception cases between the different temperature categories by Kruskal-Wallis H test
13	Supplementary Figure 4: Association of monthly intussusception cases with mean monthly temperatures
14	Supplementary Figure 5: Association of monthly intussusception cases with mean monthly humidity
15	Supplementary Figure 6: Association of monthly intussusception cases with mean monthly wind speed
16	Supplementary Figure 7: Association of monthly intussusception cases with mean monthly sunshine
17	Supplementary Figure 8: Association of monthly intussusception cases with mean monthly rainfall
18	Supplementary Table 10. Factorial analysis-of-variance of intussusception in children with season and age groups
19	Supplementary Table 11. Factorial analysis-of-variance of intussusception in children with season and regions
20	Supplementary Table 12. Multiple comparisons of seasonal intussusception in children in different seasons pooled for all sites
21	Supplementary Table 13. Multiple comparisons of seasonal intussusception in children in different seasons at regional level
22	Supplementary Table 14. Multiple comparisons of seasonal intussusception in children in different seasons for different age groups

For peer review only

For peer review only - http://bmjopen.bmj.com/site/about/guidelines.xhtml

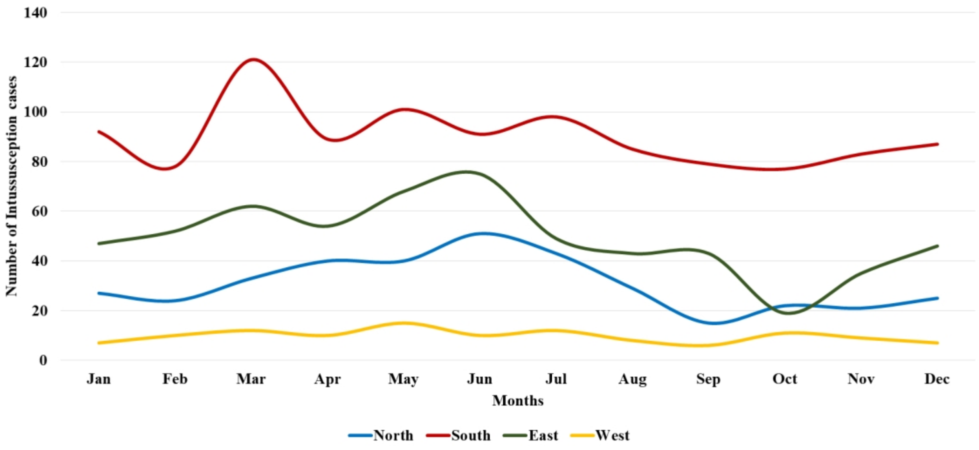

Figure 1: Monthly distribution of primary intussusception cases in children during 2010-2017

155x71mm (300 x 300 DPI)

Title: Association of meteorological parameters with intussusception in children aged under-two years: Results from a multisite bidirectional surveillance over seven years in India

Supplementary Documents

Index

Sl no	Details
1	Supplementary Figure 1: The study sites and their location on India map indicating the study regions
2	Supplementary Table 1: The International Classification of Diseases (ICD) codes for review of the cases from medical records
3	Supplementary Table 2: The seasons and months at the sites according to the regions
4	Supplementary Table 3: The monthly median (and IQR) meteorological data during 2010 and 2017 for regions
5	Supplementary Table 4: Monthly median (and IQR) meteorological data during 2010 and 2017 at the study sites
6	Supplementary Figure 2: The pattern of monthly meteorological parameters across the regions
7	Supplementary Figure 3: The pooled monthly (SF3a) and seasonal (SF3b) distribution of intussusception in children during July 2010 to September 2017
8	Supplementary Table 5. Pearson correlation analysis between monthly intussusception cases and monthly median meteorological parameters for the study sites
9	Supplementary Table 6. Spearman's Rank correlation analysis between monthly intussusception cases and monthly median meteorological parameters for the study sites
10	Supplementary Table 7. Regression analysis of the association between the intussusception cases and meteorological parameters for the study sites
11	Supplementary Table 8. Intussusception cases in children at different temperature ranges at pooled and regional level
12	Supplementary Table 9. Variations in the intussusception cases between the different temperature categories by Kruskal-Wallis H test
13	Supplementary Figure 4: Association of monthly intussusception cases with mean monthly temperatures
14	Supplementary Figure 5: Association of monthly intussusception cases with mean monthly humidity
15	Supplementary Figure 6: Association of monthly intussusception cases with mean monthly wind speed
16	Supplementary Figure 7: Association of monthly intussusception cases with mean monthly sunshine
17	Supplementary Figure 8: Association of monthly intussusception cases with mean monthly rainfall
18	Supplementary Table 10. Factorial analysis-of-variance of intussusception in children with season and age groups
19	Supplementary Table 11. Factorial analysis-of-variance of intussusception in children with season and regions

For peer review only

20	Supplementary Table 12. Multiple comparisons of seasonal intussusception in children in different seasons pooled for all sites
21	Supplementary Table 13. Multiple comparisons of seasonal intussusception in children in different seasons at regional level
22	Supplementary Table 14. Multiple comparisons of seasonal intussusception in children in different seasons for different age groups

For peer review only

For peer review only - http://bmjopen.bmj.com/site/about/guidelines.xhtml

Supplementary Figure 1: The study sites and their location on India map indicating the study regions

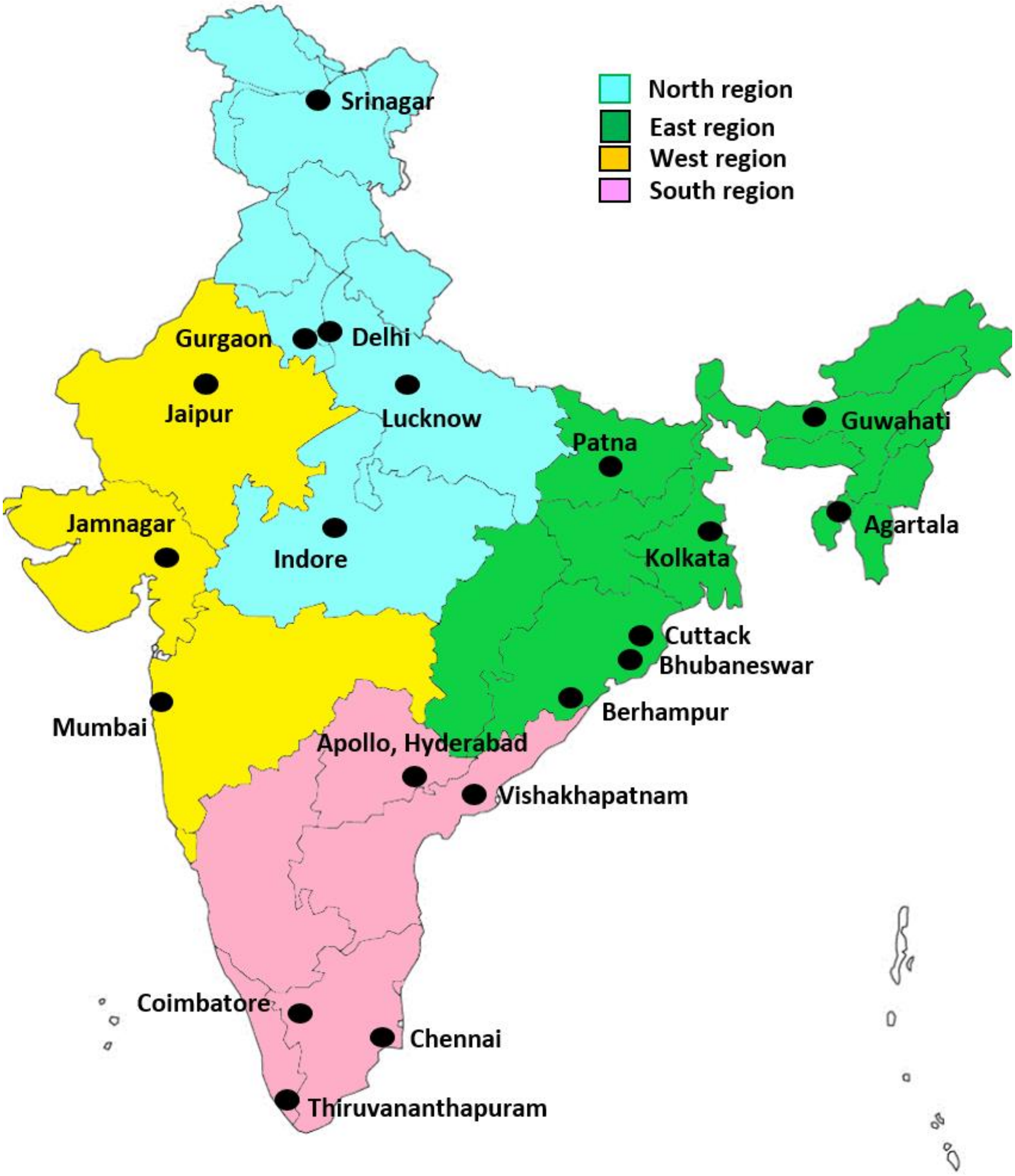

Note: The map has been prepared for use in the research project. The corresponding author is the creator of the image.

For peer review only - http://bmjopen.bmj.com/site/about/guidelines.xhtml

Supplementary Table 1: The International Classification of Diseases (ICD) codes for review of the cases from medical records

Clinical conditions considered as suspected cases	Codes	
	ICD 10	ICD 9
Intussusception	K56.1	560.0
Volvulus	K56.2	560.2
Gallstone ileus	K56.3	560.31
Other impaction of intestine	K56.4	560.30
Intestinal adhesions with obstruction	K56.5	560.81
Other and unspecified intestinal obstruction or Acute intestinal obstruction or Subacute intestinal obstruction	K56.6	560.9
Ileus, unspecified	K56.7	560.1
Paralytic ileus	K56.0	
Acute abdomen	R10.0	789.0
Blood in stool with vomiting	K92.1/K92.2 with R11.2	578.1/578.9 with 787.01

For peer review only

For peer review only - http://bmjopen.bmj.com/site/about/guidelines.xhtml

Supplementary Table 2: The seasons and months at the sites according to the regions

Region/ Site (state)	Jan	Feb	Mar	Apr	May	Jun	Jul	Aug	Sep	Oct	Nov	Dec
North region												
Srinagar (Jammu & Kashmir)	W	W	W	Sp	Sp	Su	Su	M	M	A	W	W
New Delhi (Delhi)	W	Sp	Sp	Su	Su	Su	M	M	M	A	A	W
Gurgaon (Haryana)	W	Sp	Sp	Su	Su	Su	M	M	M	A	A	W
Lucknow (Uttar Pradesh)	W	W	Sp	Su	Su	Su	M	M	M	A	A	W
Indore (Madhya Pradesh)	W	W	Sp	Su	Su	Su	M	M	M	A	A	W
South region												
Vishakhapatnam (Andhra Pradesh)	W	W	Su	Su	Su	M	M	M	M	A	A	W
Hyderabad (Telangana)	W	Sp	Su	Su	Su	M	M	M	M	A	A	W
Coimbatore (Tamil Nadu)	W	Sp	Su	Su	Su	M	M	M	M	A	A	W
Chennai (Tamil Nadu)	W	Sp	Su	Su	Su	M	M	M	M	A	A	W
Trivandrum (Kerala)	W	Sp	Su	Su	Su	M	M	M	M	A	A	W
East region												
Patna (Bihar)	W	Sp	Sp	Su	Su	Su	M	M	M	A	A	W
Bhubaneswar (Odisha)	W	Sp	Su	Su	Su	M	M	M	A	A	A	W
Cuttack (Odisha)	W	Sp	Su	Su	Su	M	M	M	A	A	A	W
Berhampur (Odisha)	W	Sp	Su	Su	Su	M	M	M	A	A	A	W
Kolkata (West Bengal)	W	Sp	Su	Su	Su	M	M	M	A	A	A	W
Guwahati (Assam)	W	W	Su	Su	Su	M	M	M	A	A	A	W
Agartala (Tripura)	W	W	Su	Su	Su	M	M	M	A	A	A	W
West region												
Jaipur (Rajasthan)	W	W	Sp	Su	Su	Su	M	M	M	A	A	W
Jamnagar (Gujarat)	W	Sp	Su	Su	Su	M	M	M	M	A	A	W
Mumbai (Maharashtra)	W	Sp	Su	Su	Su	M	M	M	M	A	A	W

Note: The seasons and colour codes used

A Autumn M Monsoon Sp Spring Su Summer W Winter

For peer review only

Supplementary Table 3: The monthly median (and IQR) meteorological data during 2010 and 2017 for regions

Region/ Months	Temperature (°C)	Rainfall (mm)	Wind speed (Kmph)	Humidity (%)	Sunshine (hours)
North region					
January	14 (11, 16)	5.7 (0.5, 13.2)	8.5 (7.1, 9.8)	54 (47, 61)	223 (217.5, 232.5)
February	17 (14, 20)	6.9 (0.5, 22.6)	9.6 (8.1, 10.3)	47 (39, 62)	266 (239, 277.5)
March	23 (21, 25)	4.9 (1.1, 19.9)	9.9 (9, 10.5)	37 (26, 45)	301 (291.5, 308.5)
April	31 (28, 32)	3.9 (1.3, 12.6)	10.5 (8.1, 12.2)	23 (18, 28)	337.5 (315, 345)
May	35 (34, 36)	3.3 (1.1, 14.3)	10.7 (8.9, 12.3)	22 (19, 30)	386 (383, 387.5)
June	35 (31, 37)	22 (8.1, 48.8)	11.1 (9.1, 12.9)	40 (31, 46)	361.5 (346.5, 369.5)
July	31 (26, 33)	72.5 (30, 126.7)	9.6 (8, 12.3)	59 (54, 69)	331.8 (279.3, 367)
August	29 (24, 31)	55.1 (26.3, 93.7)	9 (7, 10.8)	66 (56, 80)	337.8 (298.8, 359.3)
September	28 (25, 30)	31.6 (13.4, 58.1)	8.4 (7.2, 9.2)	56 (49, 76)	286.3 (269.8, 300)
October	26 (25, 27)	1.5 (0.2, 12.2)	6.5 (5.9, 7.2)	42 (34, 50)	250 (244, 262.5)
November	21 (20, 22)	0.1 (0, 3.2)	7.5 (6, 8.2)	33 (31, 39)	225 (222, 225)
December	16 (14, 18)	0.6 (0, 4.4)	7.8 (6.7, 8.8)	39 (35, 45)	231 (225.5, 232.5)
South region					
January	23 (23, 25)	2.8 (1.1, 7.7)	9.7 (8.6, 12.1)	67 (63, 71)	277.5 (274.5, 296.5)
February	25 (24, 26)	1.5 (0.4, 8)	10.1 (8.8, 12.2)	65 (51, 71)	277.5 (272.5, 286)
March	28 (27, 28)	10.1 (3.4, 29.8)	10.9 (8.9, 13.6)	66 (50, 71)	299.5 (294, 308.5)
April	30 (29, 31)	7.8 (2.9, 32.2)	12.1 (9.7, 17.1)	69.5 (52, 73)	317.5 (292.5, 333.5)
May	31 (29, 32)	20.2 (9.1, 30.5)	14.6 (12.9, 17.6)	64 (59, 71)	353.5 (317, 378)
June	28 (27, 31)	50.7 (16.5, 97.8)	18 (17.2, 18.5)	65 (62, 70)	291 (268.5, 319.5)
July	28 (26, 29)	43 (1.7, 78.5)	18.5 (17, 20.1)	68 (63, 74)	283.3 (255.5, 308.5)
August	27 (26, 29)	58.1 (7.9, 92.3)	16.2 (14.4, 17.6)	72 (66, 77)	275.5 (245.5, 296.5)
September	27 (26, 29)	61.7 (34.1, 99.9)	13.8 (12.3, 16.3)	72 (67, 76)	258 (239, 288)
October	27 (26, 28)	61.9 (44.4, 117.6)	10.6 (9.4, 12.2)	71 (68, 75)	246.8 (224.5, 267)
November	25 (24, 27)	34.8 (19.2, 108.9)	9.6 (7.2, 13.5)	72 (69, 75)	205.5 (191, 213)
December	24 (23, 26)	15 (3, 37.9)	10 (8.5, 14.3)	70.5 (66, 74)	225.5 (216, 265.5)
East region					
January	20 (17, 22)	1.7 (0.1, 5.8)	7.2 (6.4, 8.3)	60 (54, 64)	227 (224.5, 231)
February	23 (20, 24)	2.7 (1, 8.7)	8.2 (7, 9)	54 (49, 61)	210 (207, 237)
March	27 (25, 28)	8.5 (2.9, 19.4)	10 (8.9, 11)	56 (46, 60)	291.5 (287.5, 301)
April	31 (29, 32)	12.3 (3.5, 32.1)	12.5 (10.4, 14.6)	60 (54, 66)	355.5 (294.5, 370.5)
May	32 (30, 33)	25.2 (13.9, 54.5)	13.3 (11.2, 15)	66 (60, 69)	365.5 (347, 374.5)
June	32 (29, 33)	79.8 (53.9, 122.3)	12 (10.6, 13.1)	66 (62, 76)	307.5 (292.5, 327)
July	30 (28, 30)	118 (82.6, 175.2)	11.3 (9.8, 12.4)	73 (69.5, 83.5)	276.8 (250, 288.5)
August	29 (28, 30)	100 (70.8, 154.9)	10 (8.9, 11.1)	78 (73.5, 82)	286 (258, 305)
September	28 (28, 29)	89.9 (65.7, 133)	8.3 (7.3, 9.4)	79.5 (75, 83)	252.5 (236.3, 262)
October	26 (25, 27)	44.7 (24.8, 87.9)	6.7 (5.9, 7.6)	77 (71, 82)	287 (271.5, 295)
November	23 (21, 24)	3.6 (0.4, 20.4)	6.4 (5.7, 7.3)	66 (60, 77)	270 (256, 292.5)
December	21 (18, 22)	1 (0.2, 6.7)	7.1 (6.1, 8)	59 (50, 70)	229.5 (222.5, 240)
West region					
January	22 (15, 24)	0.1 (0, 0.9)	10 (8.5, 14.4)	48 (44, 51)	310 (255, 310)
February	24 (20, 25)	0.4 (0, 1)	10.8 (9.9, 14.5)	45 (44, 53)	278.5 (275.5, 281)
March	27 (25, 28)	0.2 (0, 1.2)	10.7 (10.1, 15.9)	47 (29, 53)	307.5 (305.5, 308.5)
April	29 (29, 31)	0.4 (0, 1.7)	11.2 (10, 18.3)	56 (20, 61)	300 (300, 315.5)

For peer review only - http://bmjopen.bmj.com/site/about/guidelines.xhtml

Region/ Months	Temperature (°C)	Rainfall (mm)	Wind speed (Kmph)	Humidity (%)	Sunshine (hours)
May	31 (30, 36)	0.6 (0.1, 5.5)	14.3 (10.7, 23.9)	65 (21, 68)	355 (307, 381.5)
June	31 (29, 36)	48.9 (9.7, 156.7)	16.7 (13.9, 21.7)	68 (34, 75)	311.5 (252, 359.5)
July	29 (28, 32)	121.3 (58.1, 391.4)	17.3 (13.9, 23.8)	77.5 (56.5, 82.5)	194 (158.5, 338.8)
August	28 (27, 29)	118.5 (60, 213.5)	14.1 (11.4, 22.1)	79 (73.5, 84)	196.8 (166.5, 278)
September	28 (27, 29)	70.6 (19.4, 133.8)	11.6 (9.8, 17.8)	74 (62.5, 80)	253.5 (229, 274.8)
October	28 (28, 29)	5.9 (0.5, 26.1)	9 (8.1, 12.5)	51 (41, 67)	296.5 (265, 308.5)
November	27 (23, 28)	0.1 (0, 2.3)	9 (8, 13)	44 (34, 53)	230 (225, 251.5)
December	24 (17, 25)	0 (0, 0.5)	9.8 (8.4, 14.5)	43 (39, 49)	252.5 (232.5, 293.5)

Note: Temperature (°C); Rain: Rainfall (millimetre, mm); Wind Speed (miles/second); Humidity (%); Sunshine (in hours/month)

For peer review only

Supplementary Table 4: Monthly median (and IQR) meteorological data during 2010 and 2017 at the study sites

Region/ Place (State) and months	Temperature (°C)	Rainfall (mm)	Wind speed (Kmph)	Humidity (%)	Sunshine (hour)
1. North region					
1.1 Srinagar (Jammu and Kashmir)					
January	-3 (-4,-1)	28.4 (16.8,41.9)	5.8 (5.3,6.3)	58 (44,69)	209.5 (205.5,218)
February	0 (-2,2)	69.9 (56.8,89)	5.7 (5.4,6.1)	60 (59,68)	241 (220,261.5)
March	4 (3,5)	63.4 (44.2,107)	5.3 (4.7,6.5)	60 (57,63)	282 (232.5,296)
April	8 (7,9)	45.3 (36.7,84.2)	4.6 (4.4,5)	55 (52,57)	330 (301.5,336)
May	13 (11,14)	31.6 (16.3,57.3)	4.3 (3.7,5.1)	49 (46,50)	387.5 (378.5,387.5)
June	17 (16,18)	15.8 (8.1,33.4)	4.2 (3.8,4.4)	46 (41,47)	372 (364.5,375)
July	19 (18.5,19)	22.6 (13,50.5)	5.4 (4.7,6)	57.5 (57,59)	387.5 (381.5,387.5)
August	18 (18,19)	25 (20.6,34.1)	5 (3.8,5.4)	59.5 (56,65)	371 (368,372.5)
September	15 (14,15)	9 (5.6,48.8)	3.9 (3.6,4.1)	50.5 (48,52.5)	300 (297.8,300)
October	9 (8,11)	10.5 (4.1,15.8)	3.3 (2.8,3.9)	40 (34,41)	262.5 (258.5,262.5)
November	4 (3,5)	8 (1.4,12.1)	4.6 (4.1,6)	33 (31,36)	222 (209.5,225)
December	0 (-1,1)	14.9 (5.3,24.9)	5.8 (5.4,6.7)	31 (27,42)	221.5 (211.5,228)
1.2 Delhi (Delhi) and 1.3. Gurgaon (Haryana)					
January	13 (11,14)	1 (0.3,5.7)	8.5 (7.9,9.2)	54 (53,62)	223 (220.5,228.5)
February	16 (15,18)	5.3 (0.2,7.8)	9.8 (8.9,10.1)	53 (43,63)	269.8 (266,277.5)
March	22 (21,23)	2.9 (1.1,6.5)	9.9 (9.2,10.4)	41 (33,43)	300 (292,303)
April	30 (29,32)	5.3 (2.7,9.8)	10.5 (8.4,10.7)	23 (17,27)	341.8 (331.5,344.5)
May	36 (35,36)	2.4 (0.9,6.7)	10.2 (9.8,10.7)	20.5 (15,22)	386 (383,386)
June	36 (36,38)	20.7 (8.5,30.9)	10.1 (9.8,11.8)	32 (23,36)	363.5 (361.5,369.5)
July	32.5 (32,34)	61.2 (30.7,98.2)	8.9 (8.1,10.2)	53.5 (51,61.5)	345 (331.8,365)
August	31 (30,32.5)	47.8 (28.5,78.9)	8.4 (7.7,9.7)	63.5 (54,72.5)	339.8 (329,349)
September	30.5 (29,31)	27.2 (11.9,61.1)	8.2 (7.7,8.6)	49.5 (47,67)	279.5 (272.3,294)
October	27 (26,28)	0.5 (0,1.1)	6.5 (6.2,7)	34 (31,43)	250 (248.5,250)
November	21 (21,22)	0 (0,0.2)	8.1 (6.5,8.2)	31 (27,40)	225 (223.5,225)
December	16 (15,17)	0.3 (0,1.5)	8.3 (6.9,9)	39 (35,46)	230.5 (225.5,231)
1.4. Lucknow (Uttar Pradesh)					
January	15 (14,16)	8.1 (4.7,9.6)	9.8 (8.6,10.1)	54 (53,58)	224 (219,229.5)
February	18 (17,20)	6.9 (0.5,22.6)	10.1 (9.8,11.3)	52 (42,58)	230.5 (224.5,239)
March	25 (24,25)	3.1 (2.6,10.5)	11.2 (11.1,12)	35 (26,37)	309.5 (303.5,315)
April	32 (32,34)	2 (1.3,3.9)	12.6 (12,13.4)	21 (18,26)	373.5 (363,375)
May	36 (36,37)	5.2 (0.2,6.9)	11.4 (11.2,12.3)	21 (18,25)	384.5 (380,386)
June	37 (34,39)	13.4 (3.2,100.6)	11.6 (11.2,12.9)	32 (25,45)	346.5 (321.5,362.5)
July	32 (31,33)	127.8 (83.6,151.1)	11.1 (10.2,12.3)	63 (57,69)	304.3 (289,321)
August	30 (30,31.5)	76.5 (50.8,95.9)	10.7 (9.4,11.1)	70.5 (58,74.5)	313.8 (304,344.8)
September	29.5 (28.5,30.5)	30.9 (21.1,61.8)	9.1 (8.5,9.6)	64.5 (55,75.5)	316 (301.3,328.3)
October	26 (25,27)	7.4 (0.2,31.3)	6.9 (6.1,7.9)	50 (42,62)	227.5 (225.5,238.5)
November	22 (21,22)	0 (0,0.7)	7.4 (6.9,8.5)	35 (34,48)	225 (218,225)
December	17 (17,18)	0.6 (0,1.6)	8.5 (7.6,9.9)	41 (38,46)	232.5 (231,232.5)
1.5 Indore (Madhya Pradesh)					
January	18 (17,19)	0.8 (0.4,9.1)	9.2 (8,10.5)	44 (32,47)	281 (277.5,282.5)
February	22 (21,23)	1 (0.2,11.1)	9.6 (8.6,11)	32 (29,39)	280 (277,290)
March	27 (27,27)	2.9 (0,6.4)	10 (9.8,10.4)	21 (18,26)	307 (301,310)
April	32 (32,33)	0.6 (0.2,2.8)	12.3 (11.2,14.4)	18 (17,21)	315 (310,317.5)
May	35 (34,35)	1.9 (0.6,2.7)	15.3 (14.7,16.8)	24 (22,30)	386 (384.5,387.5)
June	31 (31,32)	48.9 (29.6,72.2)	15.4 (14.6,16.9)	51 (47,57)	341 (294,352)

For peer review only - http://bmjopen.bmj.com/site/about/guidelines.xhtml

Region/ Place (State) and months	Temperature (°C)	Rainfall (mm)	Wind speed (Kmph)	Humidity (%)	Sunshine (hour)
July	27 (26,27)	124.5 (83.8,186.7)	15.5 (12.6,17.3)	80 (75,81.5)	201.5 (178.8,239)
August	25.5 (24.5,26)	101.7 (78.8,132.8)	12.1 (11.4,14.9)	88.5 (80,90)	166.5 (144.8,208.3)
September	25.5 (25,26)	52.2 (41,62.1)	9.7 (8.6,10.5)	77 (75,81)	253.5 (241.5,267.3)
October	26 (25,26)	2.3 (0.3,31.1)	7.2 (6.6,8.3)	45 (40,58)	271 (254.5,272.5)
November	23 (22,24)	0.3 (0,2.9)	7.6 (6.4,8)	37 (33,39)	225 (220.5,225)
December	19 (19,20)	0.1 (0,1.2)	8.1 (7.4,8.8)	36 (34,40)	232.5 (229.5,232.5)
2. East region					
2.1 Bhubaneswar and 2.2. Cuttack (Odisha)					
January	22 (21,22)	1.5 (0.1,5.6)	7.2 (6.4,7.4)	56.5 (56,60)	224.5 (221.5,228)
February	24.5 (24,25)	2 (0.5,8.9)	8.2 (7.1,9.3)	56 (53,62)	209.3 (202.5,210)
March	28 (28,29)	6.7 (3.6,16.6)	10.9 (9.4,11.5)	57.5 (54,62)	287.3 (275.5,290)
April	31 (31,32)	5.4 (1.6,12.3)	15 (12.4,17.2)	60.5 (57,66)	366.8 (360,372)
May	32 (32,33)	22.3 (15.4,26.9)	14.9 (13.9,15.8)	64.5 (63,67)	366.8 (362.5,375.5)
June	32 (32,33)	80 (60.3,114.7)	12.2 (12,13.7)	62.5 (62,66)	300.8 (292.5,322.5)
July	30 (30,30)	118 (80.3,137.4)	11.3 (10.1,12.1)	72 (69,73)	269.8 (253.3,283.8)
August	29 (29,30)	92.6 (67,133.3)	10.4 (9.2,11.4)	74.5 (71.5,77.5)	285 (262.3,296)
September	29 (28,29)	89.9 (67.3,112.9)	8.7 (7.3,9.8)	77 (74.5,79.5)	248.3 (234.3,257.3)
October	27 (26,27)	50.1 (25.7,89.9)	7.4 (6.7,7.9)	74 (69,82)	285.5 (263,290)
November	23.5 (23,25)	9.1 (1,28.3)	6.6 (6.2,7.2)	64.5 (60,66)	261.8 (256,268.5)
December	21 (21,22)	4.7 (0.3,11.8)	6.9 (6.6,8)	57 (53,59)	223.8 (207,229.5)
2.3 Berhampur (Odisha)					
January	22 (21,22)	1.6 (0.6,5.6)	6.9 (6.6,7.6)	66 (63,69)	224.5 (221.5,228)
February	24 (24,24)	3.6 (2.1,15.7)	8.2 (7.2,8.7)	62 (61,68)	210 (205.5,210)
March	28 (27,28)	8.5 (1.3,20.6)	10.8 (9.8,11.7)	61 (59,64)	288.5 (275.5,291)
April	30 (30,31)	9.7 (2,20.6)	13.9 (12.4,14.6)	63 (58,67)	367.5 (361.5,372)
May	32 (32,32)	13.3 (7.1,28.9)	13.3 (12.5,13.7)	67 (64,68)	368 (364,380)
June	31 (31,32)	60.7 (48.2,79.8)	11.3 (11.1,11.8)	67 (64,69)	307.5 (300,324.5)
July	29 (29,30)	82.9 (65.6,107.6)	11.1 (10.5,11.4)	73 (71,74.5)	283.5 (279.3,289)
August	29 (29,29)	93.5 (72.6,119.7)	9.4 (9.1,10)	74.5 (73,78)	294.3 (272,302.5)
September	28.5 (28,29)	91.1 (67,116)	8.1 (7.5,8.8)	76 (75,77.5)	254.8 (250.3,261.5)
October	27 (26,27)	44.9 (33.2,132.7)	8.1 (6.6,9)	72 (70,80)	289.5 (263,295)
November	24 (24,24)	44.5 (0.4,59.7)	7 (6.6,8.3)	67 (63,71)	262 (256,271.5)
December	22 (22,22)	3.3 (1,30.7)	7.8 (7,8.2)	62 (60,67)	225 (207,229.5)
2.4. Kolkata (West Bengal)					
January	20 (19,21)	0.1 (0,7.7)	9.1 (8.6,9.9)	44 (40,52)	228 (225,231)
February	23 (23,24)	4.4 (0.8,7.8)	9 (8.1,9.3)	45 (40,46)	211 (204.5,212.5)
March	28 (27,28)	8.7 (1.7,19.2)	10 (9.4,10.2)	50 (46,57)	304 (295,308.5)
April	31 (31,33)	12.5 (5.2,37.2)	14.2 (10.3,15.2)	58 (54,63)	300 (287.5,312)
May	33 (32,33)	16.2 (11.2,28.5)	15 (13.7,17.7)	61 (59,67)	371 (369.5,377.5)
June	32 (31,33)	63.7 (52.7,92.7)	13.1 (12.8,15.8)	65 (64,72)	318.5 (290.5,327)
July	30 (29.5,30)	119.7 (102.1,171.9)	12.6 (11.5,13.2)	76 (74,78)	258.8 (243.3,271.3)
August	29 (29,30)	102.7 (86,153.2)	11 (10.3,11.1)	80.5 (76,82)	277.3 (247.8,284.5)
September	28 (28,29.5)	81.4 (74.9,111.4)	9.1 (8.1,10.3)	81.5 (77,85)	236.3 (226.8,241.3)
October	27 (26,27)	27.2 (23.6,34.9)	7.3 (6.5,8.7)	72 (71,80)	280 (265.5,287.5)
November	24 (23,25)	3.3 (0.3,16.7)	7.5 (6.1,9.3)	57 (53,65)	292.5 (284,297)
December	21 (20,22)	0.1 (0,1.8)	8.7 (8.5,9.5)	47 (41,50)	230 (218,232.5)
2.5. Patna (Bihar)					
January	16 (15,17)	5.8 (0.8,8.3)	9.5 (9.4,10)	54 (51,63)	229.5 (222.5,232.5)
February	19 (18,21)	0.9 (0.4,2.7)	10.4 (9.7,11.8)	49 (47,54)	207.5 (202.5,213)
March	26 (25,26)	2.9 (1.6,5.5)	11.1 (10.1,11.7)	32 (30,33)	303.5 (291.5,305)

For peer review only - http://bmjopen.bmj.com/site/about/guidelines.xhtml

Region/ Place (State) and months	Temperature (°C)	Rainfall (mm)	Wind speed (Kmph)	Humidity (%)	Sunshine (hour)
April	33 (32,33)	6.5 (2.4,22.2)	12.3 (11.4,14.2)	28 (20,31)	373.5 (358.5,375)
May	35 (35,37)	23.2 (5,41.1)	13 (12.3,13.6)	35 (29,39)	377 (366.5,386)
June	36 (34,37)	47.6 (21.1,58.6)	11.9 (11.5,13.6)	42 (42,51)	337 (327,355)
July	32 (31,32.5)	150.4 (120.4,160.4)	12 (11.7,14)	65.5 (60.5,68.5)	283 (257.5,312.5)
August	31 (31,32)	63.4 (38.5,138.2)	10.6 (9.3,11.7)	65 (61.5,71.5)	318.5 (293,329.8)
September	29 (28.5,30.5)	66.7 (32.4,116.6)	8.7 (8.4,10.4)	67 (60.5,80)	273 (249.5,282.5)
October	26 (25,27)	29.1 (8.1,54.9)	6.8 (5.9,7.5)	63 (51,78)	297 (271.5,304)
November	22 (21,23)	0 (0,0.3)	6.8 (6.3,8.1)	45 (39,61)	232.5 (231,235)
December	18 (18,19)	0 (0,0.7)	8 (7.5,9.1)	49 (38,54)	231 (225,232.5)
2.6. Guwahati (Assam)					
January	17 (15,17)	3.9 (0.2,10.3)	3.9 (3.8,4.3)	67 (64,76)	228 (225,231)
February	20 (19,20)	4.9 (2.3,10.2)	4.2 (4,4.5)	56 (54,62)	245 (242,253.5)
March	24 (23,24)	14.1 (1.4,22.9)	4.9 (4.6,4.9)	46 (42,58)	299.5 (295,310)
April	26 (25,27)	39.7 (15.1,80.8)	4.5 (4.4,4.8)	62 (50,68)	292.5 (280.5,300)
May	27 (27,28)	83.1 (54.5,122.4)	4.2 (3.8,5)	69 (63,73)	344 (336.5,357)
June	28 (27,29)	236.2 (191.4,326.2)	3.8 (3.6,4.3)	81 (76,84)	323.5 (292.5,334)
July	27.5 (27,28)	344.4 (224.2,407.4)	3.8 (3.5,4.1)	87 (83.5,88.5)	317.8 (274.5,332.8)
August	28 (27.5,28)	235.2 (214.1,276.4)	3.8 (3.7,4.2)	84 (82,85)	297.3 (283,310.5)
September	27 (26.5,27)	159.3 (143.5,217.6)	3.4 (3.1,4)	84 (81.5,85)	272 (257.8,274.8)
October	24 (24,24)	48.8 (12.8,72.7)	3.6 (3.4,4.2)	79 (77,81)	294 (283,305.5)
November	20 (20,20)	4.5 (1,7.3)	3.8 (3.7,4.5)	78 (71,80)	294 (292,297)
December	17 (17,18)	2.5 (1.1,9)	4 (3.8,4.2)	74 (73,77)	250.5 (240,253.5)
2.7. Agartala (Tripura)					
January	18 (17,19)	0.3 (0.1,2.2)	6.7 (6.5,7.2)	61 (58,69)	231 (228,232.5)
February	21 (20,22)	5.6 (1.7,10.5)	7.2 (7,8.6)	53 (51,57)	246 (237,250.5)
March	25 (24,26)	22.6 (11.8,53.2)	8.9 (8.8,9.5)	57 (51,64)	295.5 (288,301)
April	28 (28,29)	113.4 (38.1,141.2)	11.1 (8.5,12.5)	67 (64,73)	266.5 (246,297)
May	29 (28,30)	113.8 (73.4,194.5)	9.7 (9.1,11.5)	74 (74,80)	307.5 (299.5,320)
June	29 (28,29)	99.2 (72.8,110.5)	11.1 (10.6,13.4)	82 (79,85)	286.5 (282.5,303.5)
July	28 (28,28)	73 (50.4,103.5)	12.1 (10.4,13.4)	84.5 (83.5,85.5)	261.8 (244.8,276)
August	28 (27.5,28)	78.4 (61.2,101.3)	10.6 (9.4,11.1)	86.5 (82,87.5)	249.3 (231.8,271)
September	27 (27,28)	57.1 (43.3,144.7)	8.4 (7.5,9.4)	86.5 (82,89)	249 (225,259)
October	25 (25,26)	55.3 (23,121.8)	5.9 (4.9,6)	85 (82,87)	284.5 (271,293.5)
November	21 (21,22)	5.2 (0.2,31)	5.5 (5.3,6.1)	78 (77,79)	300 (282,300)
December	18 (18,20)	0.7 (0.2,6.7)	6.1 (5.1,6.2)	71 (70,73)	264.5 (258.5,267.5)
3. South region					
3.1. Vishakhapatnam (Andhra Pradesh)					
January	23 (23,24)	3.5 (1.3,6.1)	10.6 (9.4,11.1)	68 (67,72)	277.5 (274.5,277.5)
February	25 (24,25)	1.4 (0.9,4.2)	10.1 (9.4,10.8)	68 (65,70)	280 (277.5,288.5)
March	27 (27,28)	3.4 (3.3,16.2)	13.6 (11.5,14)	70 (65,70)	308.5 (298,310)
April	29 (29,30)	5 (1.3,12.6)	17.4 (16.7,19.1)	71 (70,74)	330 (320,335)
May	31 (31,31)	8.6 (3.5,18)	17 (15.9,18.5)	71 (70,72)	378 (371,381.5)
June	31 (31,31)	91 (40.8,129.1)	16.9 (14.3,17.3)	70 (66,71)	274.5 (268.5,306)
July	29.5 (29,30)	77.6 (60.1,105.1)	18.5 (16.2,20.1)	73 (71.5,74.5)	252.5 (226.8,289.8)
August	29 (29,30)	111.7 (96.7,120.4)	13.8 (13.5,16.6)	74.5 (73,77)	241 (234.5,263.5)
September	29 (29,29)	127 (104.7,164.2)	11.8 (11.7,13)	76 (75.5,77)	234.5 (226.3,243.8)
October	27 (27,28)	90.4 (50.2,182.7)	11.7 (9.1,13.2)	72 (69,77)	222 (205.5,236.5)
November	25 (25,26)	46.2 (19.2,144.8)	12.1 (11.5,15.1)	70 (66,71)	207 (202,217.5)
December	24 (24,24)	4.2 (2.6,14.6)	13.8 (12,14.4)	67 (66,70)	225.5 (222,228)

For peer review only - http://bmjopen.bmj.com/site/about/guidelines.xhtml

Region/ Place (State) and months	Temperature (°C)	Rainfall (mm)	Wind speed (Kmph)	Humidity (%)	Sunshine (hour)
3.2. Hyderabad (Telangana)					
January	22 (21,23)	0.5 (0.1,1.5)	12.1 (10.1,12.6)	47 (46,51)	277.5 (274.5,277.5)
February	24 (24,25)	0 (0,1.6)	12.4 (10.6,12.9)	41 (38,45)	280 (277.5,288.5)
March	28 (28,29)	3.4 (0.1,10.6)	11.1 (10.3,11.9)	34 (32,39)	308.5 (298,310)
April	32 (31,33)	2.9 (1.5,5.3)	9.7 (9.2,11.9)	33 (27,35)	330 (320,335)
May	34 (33,35)	9.4 (7.3,17.6)	12.9 (12,14.4)	30 (28,34)	378 (371,381.5)
June	29 (28,29)	111.6 (33.3,131.2)	18.7 (18.1,20.7)	57 (49,63)	274.5 (268.5,306)
July	26 (26,27.5)	66.4 (47.1,81)	20.7 (18.4,23)	72.5 (65,75)	252.5 (226.8,289.8)
August	26 (25,26.5)	68.6 (36,77.7)	16.6 (15.3,17.5)	76 (70,78)	241 (234.5,263.5)
September	26 (25,26)	51.9 (40.3,74.1)	12.8 (11.3,13.9)	74.5 (70.5,78.5)	234.5 (226.3,243.8)
October	24 (24,25)	35.9 (14.9,45.2)	10.1 (9.2,10.6)	65 (59,70)	222 (205.5,236.5)
November	22 (21,23)	3.1 (0.5,18.8)	10.2 (9.5,10.8)	60 (51,62)	207 (202,217.5)
December	21 (20,22)	0.3 (0.2,1.1)	10.3 (10.1,11.3)	50 (49,53)	225.5 (222,228)
3.3. Coimbatore (Tamil Nadu)					
January	23 (23,23)	2.2 (1.1,26.6)	7.4 (6.9,8.6)	63 (63,66)	290 (285,305.5)
February	25 (24,25)	3 (0.9,8.3)	7.8 (7.4,8.9)	55 (51,56)	277 (272,286)
March	28 (27,28)	26.7 (10.1,60.5)	7.1 (7.1,8.6)	53 (50,55)	299.5 (298,302.5)
April	30.5 (30,31)	8.9 (6.7,28.2)	9.2 (8.3,11.6)	52 (52,54)	297.5 (284.5,297.5)
May	30 (29,30)	29.7 (29.7,30.5)	12.4 (11.9,13.2)	59 (59,61)	334 (317,335)
June	27 (27,27)	4.9 (4.9,6.7)	18.1 (17.7,18.3)	64 (64,66)	301.5 (285.5,302)
July	27 (27,28)	0.2 (0,1.7)	18.5 (17.9,19.3)	63 (61,64)	291 (268.5,308.5)
August	27 (26,27)	3.1 (0.9,7.9)	16.1 (14.8,17.6)	65 (63,67)	291.5 (274.5,296.5)
September	27 (27,27)	15.6 (8.3,42.8)	16.3 (14.5,17.6)	65.5 (65,67)	278 (256.5,282)
October	26 (26,26)	53.2 (53.2,63.8)	9.7 (8.3,10.6)	68 (68,71)	268.3 (254.5,270)
November	24 (24,24)	32.6 (32.6,34.9)	6.1 (6.1,7)	72 (69,78)	205.5 (194,206.5)
December	23 (23,23)	14.3 (6,15.5)	7 (6.7,7.9)	71 (67,74)	242.3 (225.5,267)
3.4. Chennai (Tamil Nadu)					
January	25 (25,25)	7.7 (4.4,10.5)	15.4 (12.4,16.6)	73 (70,75)	267 (259.5,273.5)
February	25 (25,26)	1.5 (0.3,26)	13.2 (11.7,14)	72 (70,74)	275.5 (272.5,287)
March	28 (27,28)	1.8 (1.1,10.1)	14.8 (13.6,15.5)	72 (71,73)	301 (294,304)
April	30 (30,30)	7.2 (0.3,35.3)	18.9 (16.9,20.1)	72 (71,74)	344.5 (330,352.5)
May	32 (32,32)	18.6 (8.3,37.5)	18.9 (18.2,19.1)	66 (66,67)	381.5 (355,383)
June	31 (31,32)	37.8 (19,63.2)	17.4 (16.9,18.4)	62 (61,66)	336.5 (327.5,348.5)
July	30.5 (30,31)	37.9 (22.1,61.8)	16.8 (15.7,17.5)	65 (63,65.5)	343.5 (339,354.3)
August	30 (29,30)	75.4 (61.7,88.7)	15.2 (14.1,16)	69 (66.5,70.5)	339.3 (335.8,359.8)
September	29.5 (29,30)	101.1 (91.5,105.6)	13.3 (13,13.8)	72 (70,72)	333.5 (317,347.8)
October	29 (28,29)	102.6 (81.5,154.2)	12.2 (10.9,13.8)	73 (71,74)	252 (247,263.5)
November	27 (27,27)	60 (48.4,344.4)	17.3 (15.3,17.7)	74 (72,79)	201 (190.5,213)
December	26 (25,26)	84.8 (58.6,111.3)	18 (16.5,20)	76 (72,77)	199 (188.5,208)
3.5. Trivandrum (Kerala)					
January	26 (26,26)	3.2 (1.5,7.7)	8.7 (8.2,9.5)	70 (68,71)	299.5 (296.5,301)
February	26 (26,26)	6.1 (1.4,23)	8.8 (8.7,10.7)	71 (65,72)	278.5 (269.5,284)
March	28 (27,28)	37.8 (14.8,53.6)	9.8 (9.1,10.7)	71 (70,75)	293.5 (288,299.5)
April	28 (28,29)	57.3 (32.2,115.9)	12.2 (10.7,14.3)	79 (72,79)	278 (264,285)
May	28 (28,29)	24.9 (17.4,42.5)	15 (14.6,18)	79 (72,79)	252 (245.5,267.5)
June	27 (27,28)	82.2 (46.9,110.2)	20.1 (17.4,21.5)	78 (74,79)	243 (213,256)
July	26.5 (26,27.5)	65.6 (15.1,101.1)	21.1 (18.7,22.1)	78 (75,80)	266.3 (248.3,278.8)
August	26 (26,27.5)	33.6 (7.8,72.9)	18.8 (17,20.5)	80 (75.5,81)	264.5 (240.8,285.5)
September	26.5 (26,27)	61.7 (40.3,77.2)	17 (13.8,17.4)	80 (77,81.5)	256 (244,263.3)
October	27 (26,27)	117.9 (32.3,220.7)	11.4 (10.1,14.9)	78 (75,80)	266 (245.5,286)

For peer review only - http://bmjopen.bmj.com/site/about/guidelines.xhtml

Region/ Place (State) and months	Temperature (°C)	Rainfall (mm)	Wind speed (Kmph)	Humidity (%)	Sunshine (hour)
November	27 (27,27)	107.7 (47,140)	8.6 (8,9.1)	74 (72,76)	189 (174,205)
December	26 (26,27)	25.1 (15.7,37)	8.9 (8.6,9.6)	72 (72,75)	272.5 (267,280.5)
4. West region					
4.1. Mumbai (Maharashtra)					
January	24 (24,25)	0 (0,0.4)	10 (9.7,10.9)	52 (51,53)	310 (310,310)
February	25 (25,26)	0.4 (0,2.9)	10 (9.2,10.2)	54 (51,55)	280 (275.5,281)
March	28 (27,28)	0.6 (0,1.3)	10.5 (9.6,10.7)	53 (51,56)	308.5 (305.5,310)
April	29 (29,29)	0.1 (0,0.2)	9.7 (9.4,10.3)	63 (61,66)	300 (292.5,300)
May	30 (29,30)	1.3 (0.6,8)	10.5 (10.1,10.7)	72 (68,73)	305.5 (292,307)
June	29 (29,29)	292.8 (156.7,370)	16 (14.6,16.7)	78 (75,78)	217 (190,252)
July	27 (27,28)	450.2 (391.4,540.5)	17.3 (16,18.7)	83.5 (82,85.5)	164.8 (147.5,193.3)
August	27 (27,27)	282.7 (213.5,376.4)	14.1 (12.7,15.5)	85.5 (84,86.5)	190.8 (186.5,212.8)
September	27 (27,27)	136.2 (103.3,196.1)	9.8 (9.5,11.7)	82 (80,83)	229.8 (225.5,245)
October	28 (27,29)	29.9 (25.5,48.4)	8.1 (7.7,8.8)	67 (67,72)	299.5 (286.5,304)
November	27 (27,28)	2.3 (0,9.9)	9 (8.6,9)	55 (53,58)	257.5 (251.5,258.5)
December	26 (25,26)	0.2 (0,3.4)	9.8 (9,10)	51 (49,55)	295 (293.5,297.5)
4.2. Jamnagar (Gujarat)					
January	22 (21,23)	0 (0,0.1)	15.8 (14.4,17)	44 (41,49)	310 (307,310)
February	24 (23,24)	0 (0,0.6)	16 (14.5,16.8)	45 (44,50)	278.5 (274.5,281)
March	27 (27,28)	0.1 (0,0.2)	16.5 (15.9,17.1)	47 (44,53)	308.5 (306,308.5)
April	29 (29,30)	0 (0,0.4)	20.8 (18.3,21.3)	56 (54,56)	300 (297,300)
May	31 (30,31)	0.1 (0.1,0.2)	24.8 (23.9,25.3)	65 (63,67)	355 (353.5,357.5)
June	31 (31,32)	35.8 (5,93.6)	23.9 (21.7,27.9)	68 (67,69)	311.5 (296.5,321)
July	29 (29,29)	119.4 (88.5,141.7)	25.4 (23.8,27.9)	77.5 (75,78)	167.8 (143.5,197.5)
August	28 (28,28.5)	61.6 (39.7,107.3)	23.2 (22.1,24.6)	79 (77,80)	156.5 (143.5,182)
September	28 (28,29)	70.6 (24.3,113.3)	18.2 (17.8,19.7)	74 (73.5,77)	244.8 (225.3,259)
October	30 (29,31)	0.8 (0.2,12.4)	14 (12.5,14.1)	51 (48,62)	310 (308,310)
November	28 (27,29)	0.1 (0,1.9)	13.9 (13,14.6)	43 (40,50)	230 (228.5,231)
December	24 (24,25)	0 (0,0)	16.2 (14.5,17.1)	43 (39,46)	252.5 (250,252.5)
4.3. Jaipur (Rajasthan)					
January	14 (14,15)	1.9 (0.3,7.9)	8.3 (8.2,8.5)	47 (42,50)	253.5 (245.5,255)
February	18 (18,20)	1 (0.3,3.5)	10.2 (8.9,10.9)	34 (31,44)	277.5 (275.5,288.5)
March	25 (24,25)	0.8 (0,4.1)	10.2 (10,11)	24 (24,29)	304 (301.5,308.5)
April	31 (31,32)	3 (0.8,4.6)	11.2 (10.5,11.7)	19 (16,20)	323 (315.5,327.5)
May	36 (36,36)	5.5 (0.4,6.9)	14.3 (13.1,16.5)	19 (15,21)	383.5 (381.5,385)
June	36 (36,37)	9.9 (3.5,20.5)	13.9 (12.4,17.4)	30 (28,34)	363 (359.5,367.5)
July	32 (32,33.5)	48.4 (33.6,69)	12.8 (10.6,13.9)	55.5 (51,56.5)	351.8 (338.8,369.5)
August	29.5 (29,31)	74.1 (59.6,138.3)	11.1 (10.5,12.7)	66.5 (58.5,73.5)	287.5 (278,302.5)
September	29.5 (28.5,30)	19.4 (14.2,31)	10.4 (9.4,11.5)	53.5 (46.5,62.5)	281.8 (274.8,290.5)
October	28 (27,28)	2.3 (0,4.7)	8.4 (7.4,9.4)	31 (27,41)	263.5 (262.5,265)
November	22 (21,23)	0 (0,0.8)	7.2 (6.7,8)	29 (26,34)	225 (223.5,225)
December	16 (16,17)	0.4 (0,0.7)	7.6 (6.3,8.4)	37 (33,39)	232.5 (229.5,232.5)

Note: IQR- Interquartile range

For peer review only - http://bmjopen.bmj.com/site/about/guidelines.xhtml

Supplementary Figure 2: The pattern of monthly meteorological parameters across the regions

SF2a: Monthly median temperature for different regions in India (^{0}C)

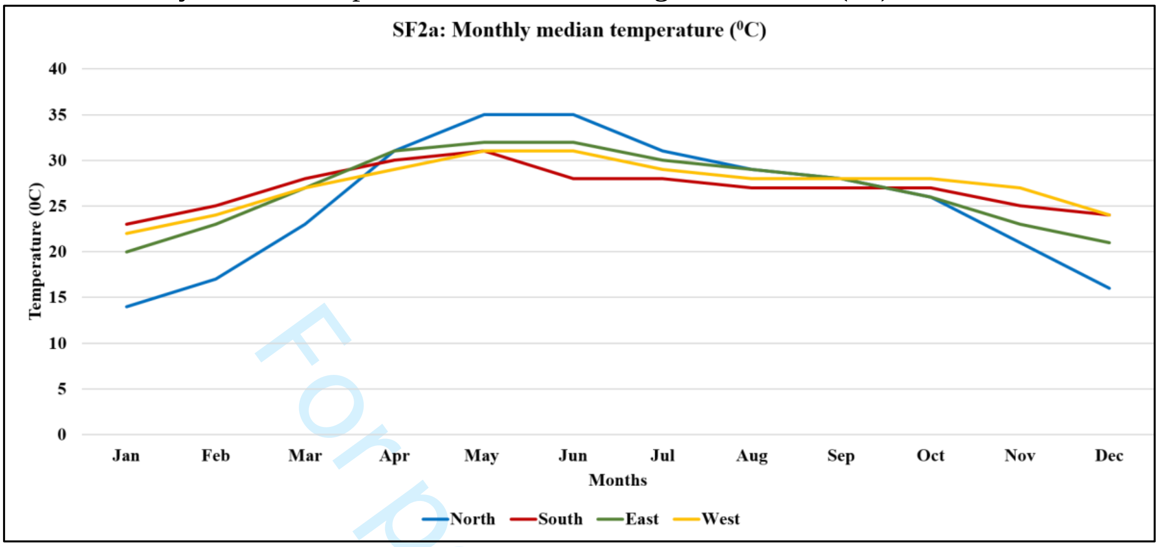

SF2b: Monthly median rainfall (mm) for different regions in India

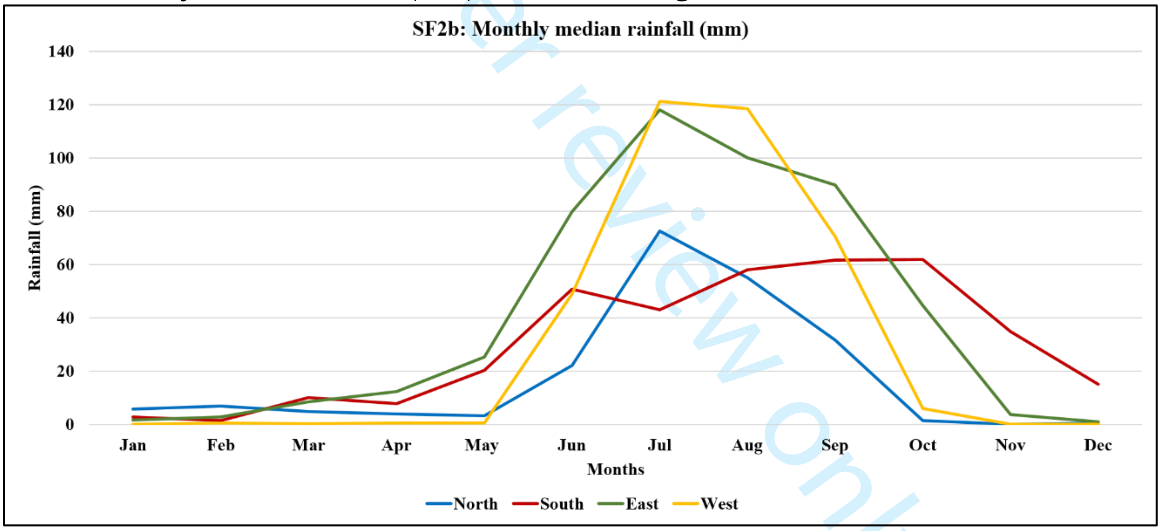

For peer review only - http://bmjopen.bmj.com/site/about/guidelines.xhtml

SF2c: Monthly median wind speed (Kmph) for different regions in India

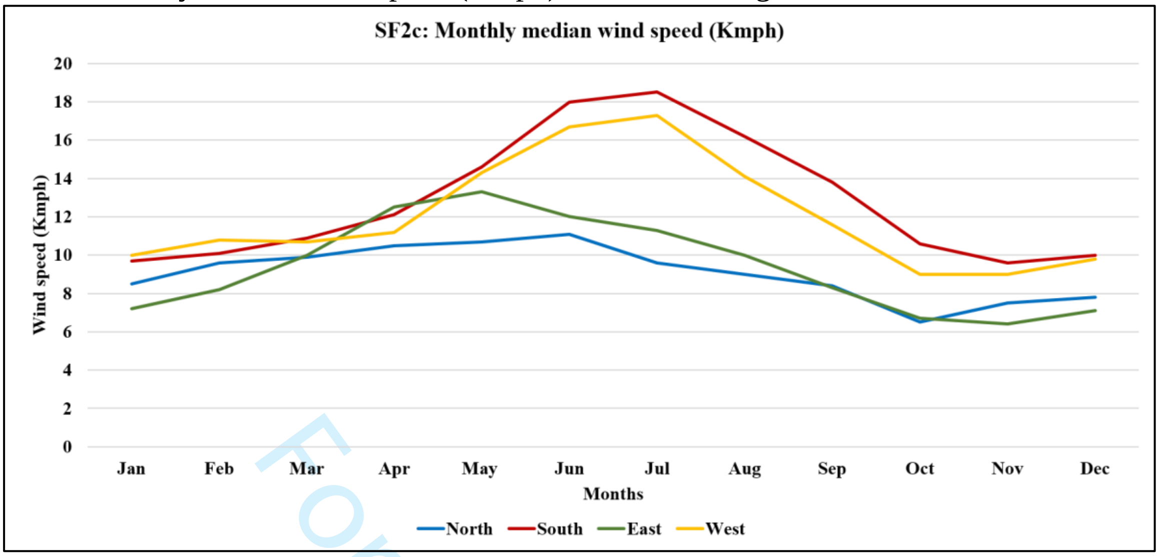

SF2d: Monthly median humidity (%) for different regions in India

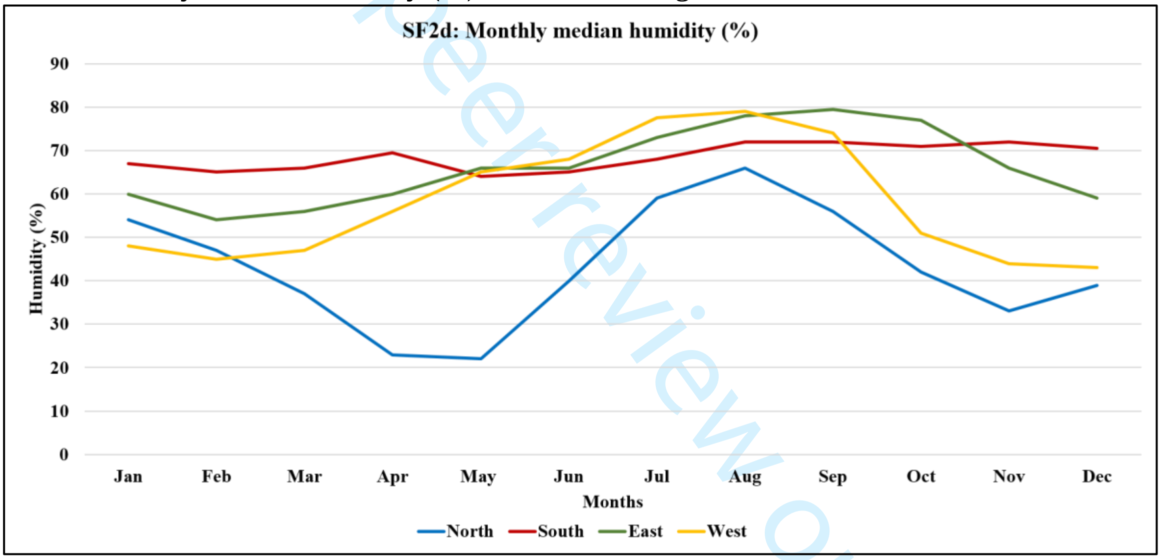

SF2e: Monthly median sunshine (hours) for different regions in India

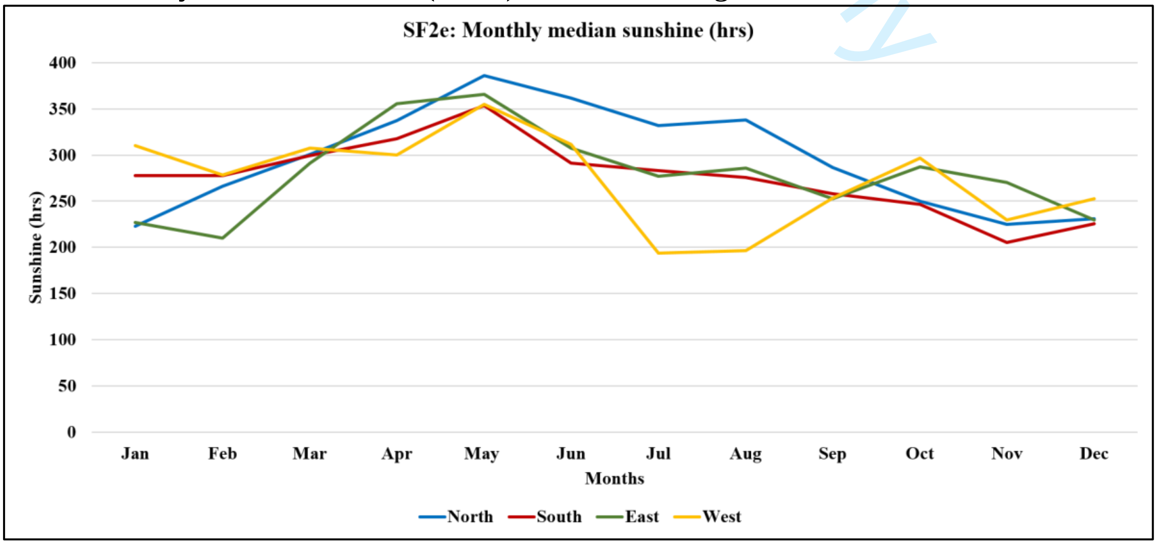

For peer review only - http://bmjopen.bmj.com/site/about/guidelines.xhtml

Supplementary Figure 3: The pooled monthly (SF3a) and seasonal (SF3b) distribution of intussusception in children during July 2010 to September 2017

SF3a: The monthly trend of intussusception cases

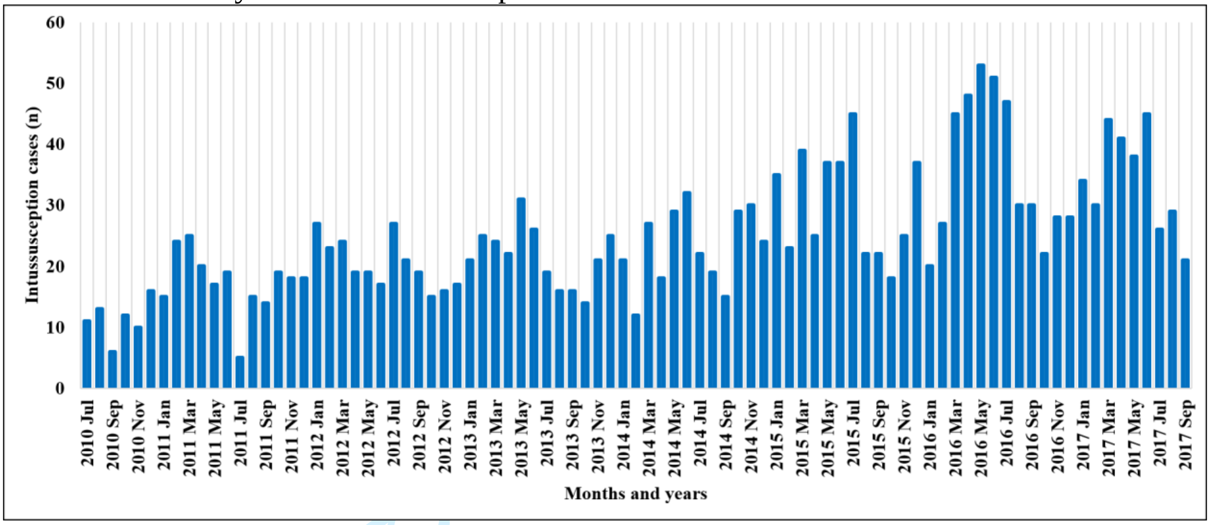

SF3b: The seasonal trend of intussusception cases

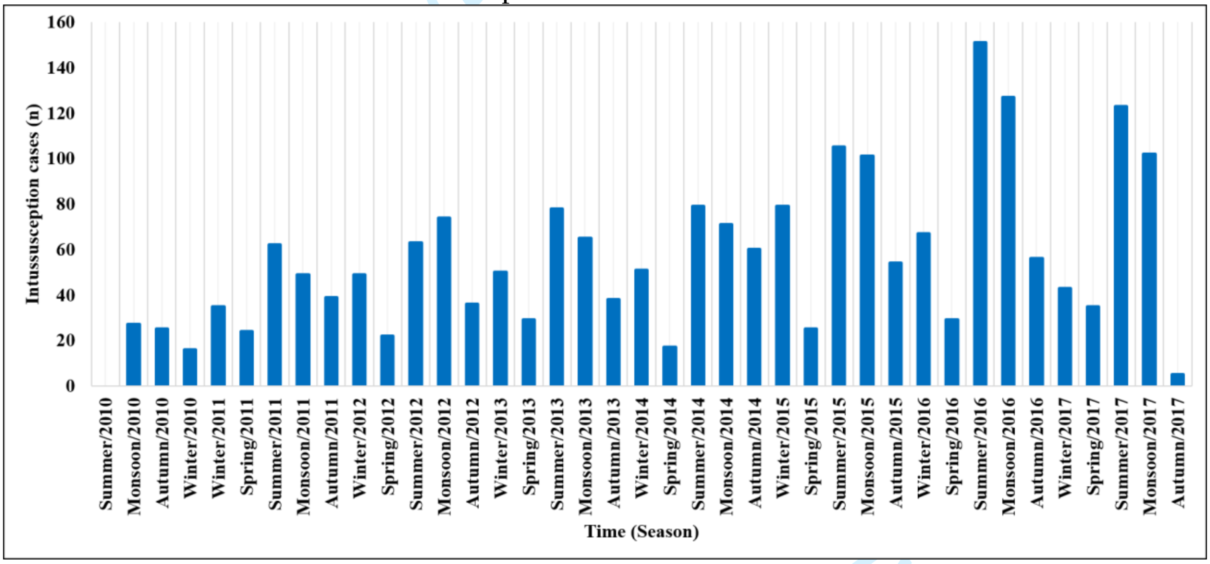

For peer review only - http://bmjopen.bmj.com/site/about/guidelines.xhtml

Supplementary Table 5. Pearson correlation analysis between monthly intussusception cases and monthly median meteorological parameters for the study sites

Regions/Study sites (n, number of cases)	Pearson correlation coefficient (r) for meteorological parameters				
	Temperature (°C)	Rainfall (mm)	Wind Speed (kmph)	Humidity (%)	Sunshine (hour)
North region					
Srinagar (n=145)	.253*	.164	.183	.012	.239*
Delhi and Gurgaon (n=96)	.107	-.045	.183*	-.173*	.134
Lucknow (110)	.091	.003	.285**	-.106	.192
Indore (n=19)	.101	-.211*	.290**	-.309**	.188
East region					
Bhubaneswar/Cuttack (n=335)	.047	-.056	.125	-.167*	.035
Berhampur (n=30)	.050	.047	.035	.044	.036
Kolkata (n=80)	.220*	-.052	.291**	-.144	.253*
Patna (n=19)	-.124	-.189	-.038	-.216*	-.040
Guwahati (n=112)	.220*	.001	.317**	-.119	.194
Agartala (n=17)	.082	.080	.139	-.017	.016
South region					
Vishakhapatnam (n=77)	.088	.053	-.002	.022	.029
Hyderabad (n=2)	-.030	-.061	-.131	.022	-.096
Coimbatore (n=136)	-.015	-.075	-.155	-.028	.017
Chennai (n=92)	.052	-.017	.007	.015	-.016
Trivandrum (n=774)	.085	-.286**	-.011	-.323**	.229*
West region					
Mumbai (n=34)	-.066	-.081	-.027	-.103	.035
Jamnagar (n=54)	.110	-.017	.011	.026	.105
Jaipur (n=29)	.187	-.078	.148	.001	.122

Note: Pearson correlation coefficient (r); Significance two tailed
** Significance level- p value <0.05; ** Significance level- p value <0.01*

For peer review only

Supplementary Table 6. Spearman's Rank correlation analysis between monthly intussusception cases and monthly median meteorological parameters for the study sites

Regions/Study sites (n, number of cases)	Pearson correlation coefficient (r) for meteorological parameters				
	Temperature (°C)	Rainfall (mm)	Wind Speed (kmph)	Humidity (%)	Sunshine (hour)
North region					
Srinagar (n=145)	.208	.122	.146	.032	.169
Delhi and Gurgaon (n=96)	.067	-.101	.136	-.184*	.065
Lucknow (110)	.166	.064	.331**	-.101	.225*
Indore (n=19)	.095	-.148	.286**	-.301**	.138
East region					
Bhubaneswar/Cuttack (n=335)	.036	-.068	.117	-.143	-.009
Berhampur (n=30)	.058	.037	.044	.035	.021
Kolkata (n=80)	.097	-.084	.170	-.236*	.125
Patna (n=19)	-.146	-.264*	-.085	-.231*	-.040
Guwahati (n=112)	.240*	-.014	.290**	-.118	.165
Agartala (n=17)	.107	.107	.154	.021	.044
South region					
Vishakhapatnam (n=77)	.106	.081	.048	.017	.060
Hyderabad (n=2)	-.012	-.052	-.138	.040	-.043
Coimbatore (n=136)	.002	-.123	-.147	-.091	.070
Chennai (n=92)	.055	.041	.025	.055	-.027
Trivandrum (n=774)	.134	-.287**	-.026	-.353**	.301**
West region					
Mumbai (n=34)	-.025	-.140	-.092	-.120	.046
Jamnagar (n=54)	.056	.071	.019	-.009	.106
Jaipur (n=29)	.131	-.082	.069	-.075	.079

Note: Pearson correlation coefficient (r); Significance two tailed
** Significance level- p value <0.05; ** Significance level- p value <0.01*

For peer review only

For peer review only - http://bmjopen.bmj.com/site/about/guidelines.xhtml

Supplementary Table 7. Regression analysis of the association between the intussusception cases and meteorological parameters for the study sites.

Study sites/ Variables	Regression coefficient, β (95% CI)	Standard error, S_β	χ2 - value	p value
North region				
Srinagar (n=145)[#]				
Temperature (^{0}C)	0.043(0.003,0.082)	0.020	4.479	**0.034**
Rainfall (mm)	0.007(-0.004,0.018)	0.005	1.616	0.204
Wind Speed (Kmph)	0.150(-0.048,0.347)	0.100	2.207	0.137
Humidity (%)	0.001(-0.018,0.020)	0.009	0.010	0.921
Sunshine (hour)	0.005(0.000,0.010)	0.002	3.930	**0.047**
Delhi and Gurgaon (n=96)[*]				
Temperature (^{0}C)	0.025(-0.005,0.055)	0.015	2.712	0.100
Rainfall (mm)	-0.003(-0.012,0.006)	0.004	0.476	0.490
Wind Speed (Kmph)	0.229(0.068,0.390)	0.082	7.805	**0.005**
Humidity (%)	-0.021(-0.036,-0.005)	0.007	7.041	**0.008**
Sunshine (hour)	0.004(0.000,0.008)	0.002	4.205	**0.040**
Lucknow (n=110)[*]				
Temperature (^{0}C)	0.013(-0.014,0.041)	0.014	0.873	0.350
Rainfall (mm)	0.000(-0.008,0.008)	0.004	0.001	0.972
Wind Speed (Kmph)	0.224(0.074,0.374)	0.077	8.580	**0.003**
Humidity (%)	-0.008(-0.022,0.006)	0.007	1.195	0.274
Sunshine (hour)	0.004(0.000,0.007)	0.002	3.915	**0.048**
Indore (n=19)[*]				
Temperature (^{0}C)	0.036(-0.033,0.105)	0.035	1.055	0.304
Rainfall (mm)	-0.039(-0.078,0.001)	0.020	3.723	0.054
Wind Speed (Kmph)	0.623(0.193,1.054)	0.219	8.062	**0.005**
Humidity (%)	-0.059(-0.097,-0.021)	0.019	9.110	**0.003**
Sunshine (hour)	0.009(0.000,0.017)	0.004	3.556	0.059
East region				
Bhubaneswar/Cuttack (n=335)[#]				
Temperature (^{0}C)	0.012(-0.032,0.057)	0.023	.286	0.593
Rainfall (mm)	-0.001(-0.006,0.003)	0.002	.429	0.513
Wind Speed (Kmph)	0.060(-0.021,0.141)	0.041	2.131	0.144
Humidity (%)	-0.022(-0.044,0.000)	0.011	3.953	**0.047**
Sunshine (hour)	0.001(-0.003,0.005)	0.002	.157	0.692
Berhampur (n=30)[*]				
Temperature (^{0}C)	0.023(-0.068,0.114)	0.046	0.239	0.625
Rainfall (mm)	0.002(-0.006,0.01)	0.004	0.211	0.646
Wind Speed (Kmph)	0.027(-0.128,0.182)	0.079	0.118	0.731
Humidity (%)	0.009(-0.032,0.051)	0.021	0.186	0.666
Sunshine (hour)	0.001(-0.006,0.009)	0.004	0.125	0.724
Kolkata (n=80)[*]				
Temperature (^{0}C)	0.113(0.050,0.176)	0.032	12.448	**0.000**
Rainfall (mm)	-0.002(-0.007,0.003)	0.002	0.710	0.400
Wind Speed (Kmph)	0.233(0.134,0.332)	0.050	21.269	**0.000**
Humidity (%)	-0.032(-0.059,-0.005)	0.013	5.433	**0.020**
Sunshine (hour)	0.010(0.005,0.014)	0.002	16.577	**0.000**

For peer review only - http://bmjopen.bmj.com/site/about/guidelines.xhtml

Patna (n=19)*				
Temperature (^{0}C)	-0.067(-0.177,0.042)	0.056	1.452	0.228
Rainfall (mm)	-0.012(-0.025,0.001)	0.006	3.157	0.076
Wind Speed (Kmph)	-0.037(-0.235,0.161)	0.101	0.137	0.711
Humidity (%)	-0.063(-0.124,-0.003)	0.031	4.208	**0.040**
Sunshine (hour)	-0.002(-0.012,0.008)	0.005	0.150	0.698
Guwahati (n=112)*				
Temperature (^{0}C)	0.050(0.002,0.098)	0.025	4.097	**0.043**
Rainfall (mm)	0.000(-0.004,0.004)	0.002	0.000	0.995
Wind Speed (Kmph)	0.118(0.038,0.199)	0.041	8.408	**0.004**
Humidity (%)	-0.012(-0.034,0.01)	0.011	1.212	0.271
Sunshine (hour)	0.004(0.000,0.008)	0.002	3.207	0.073
Agartala (n=17)*				
Temperature (^{0}C)	0.060(-0.066,0.186)	0.064	0.874	0.350
Rainfall (mm)	0.005(-0.006,0.016)	0.005	0.838	0.360
Wind Speed (Kmph)	0.166(-0.042,0.374)	0.106	2.435	0.119
Humidity (%)	-0.005(-0.061,0.050)	0.028	0.037	0.848
Sunshine (hour)	0.001(-0.009,0.011)	0.005	0.033	0.857
South region				
Vishakhapatnam (n=77)*				
Temperature (^{0}C)	0.043(-0.058,0.144)	0.051	0.702	0.402
Rainfall (mm)	0.003(-0.007,0.012)	0.005	0.255	0.613
Wind Speed (Kmph)	-0.001(-0.071,0.070)	0.036	0.001	0.981
Humidity (%)	0.008(-0.069,0.085)	0.039	0.042	0.837
Sunshine (hour)	0.001(-0.005,0.007)	0.003	0.078	0.780
Hyderabad (n=2)*				
Temperature (^{0}C)	-0.088(-0.717,0.541)	0.321	0.075	0.784
Rainfall (mm)	-0.018(-0.085,0.048)	0.034	0.298	0.585
Wind Speed (Kmph)	-0.650(-2.074,0.774)	0.726	0.801	0.371
Humidity (%)	0.051(-0.436,0.539)	0.249	0.043	0.837
Sunshine (hour)	-0.017(-0.056,0.022)	0.020	0.764	0.382
Coimbatore (n=136)*				
Temperature (^{0}C)	-0.007(-0.082,0.068)	0.038	0.033	0.857
Rainfall (mm)	-0.003(-0.011,0.004)	0.004	0.825	0.364
Wind Speed (Kmph)	-0.053(-0.108,0.002)	0.028	3.506	0.061
Humidity (%)	-0.010(-0.067,0.047)	0.029	0.119	0.730
Sunshine (hour)	0.000(-0.004,0.005)	0.002	0.043	0.836
Chennai (n=92)*				
Temperature (^{0}C)	0.027(-0.065,0.120)	0.047	0.337	0.562
Rainfall (mm)	-0.001(-0.010,0.008)	0.005	0.036	0.850
Wind Speed (Kmph)	0.003(-0.062,0.067)	0.033	0.006	0.938
Humidity (%)	0.006(-0.064,0.076)	0.036	0.028	0.868
Sunshine (hour)	0.000(-0.006,0.005)	0.003	0.030	0.863
Trivandrum (n=774)*				
Temperature (^{0}C)	0.015(-0.016,0.047)	0.016	0.895	0.344
Rainfall (mm)	-0.005(-0.008,-0.002)	0.002	10.159	**0.001**
Wind Speed (Kmph)	-0.001(-0.024,0.021)	0.011	0.016	.898
Humidity (%)	-0.044(-0.068,-0.020)	0.012	12.938	**0.000**

For peer review only - http://bmjopen.bmj.com/site/about/guidelines.xhtml

Sunshine (hour)	0.002(0.001,0.004)	0.001	6.538	**0.011**
West region				
Mumbai (n=34)*				
Temperature (^{0}C)	-0.040(-0.165,0.085)	0.064	0.387	0.534
Rainfall (mm)	-0.003(-0.011,0.005)	0.004	0.580	0.446
Wind Speed (Kmph)	-0.016(-0.140,0.108)	0.063	0.065	0.799
Humidity (%)	-0.013(-0.039,0.013)	0.013	0.929	0.335
Sunshine (hour)	0.001(-0.006,0.008)	0.004	0.108	0.742
Jamnagar (n=54)*				
Temperature (^{0}C)	0.066(-0.042,0.173)	0.055	1.440	0.230
Rainfall (mm)	-0.001(-0.006,0.005)	0.003	0.035	0.852
Wind Speed (Kmph)	0.006(-0.090,0.102)	0.049	0.015	0.903
Humidity (%)	0.003(-0.017,0.023)	0.010	0.080	0.777
Sunshine (hour)	0.003(-0.002,0.009)	0.003	1.305	0.253
Jaipur (n=29)*				
Temperature (^{0}C)	0.150(-0.009,0.309)	0.081	3.425	0.064
Rainfall (mm)	-0.003(-0.012,0.005)	0.004	0.600	0.439
Wind Speed (Kmph)	0.092(-0.031,0.216)	0.063	2.141	0.143
Humidity (%)	0.000(-0.027,0.027)	0.014	0.000	0.993
Sunshine (hour)	0.005(-0.003,0.013)	0.004	1.471	0.225

*Note: Tests used for regression analysis: * Poisson Regression; # Negative Binomial Regression*

For peer review only

Supplementary Table 8. Intussusception cases in children at different temperature ranges at pooled and regional level

Pooled/ Region	Temperature range (°C)	Years of data collection								Total
		2010	2011	2012	2013	2014	2015	2016	2017	
Pooled	<20°C	8	14	24	29	26	39	55	51	246
	21°C -25°C	2	31	44	32	43	54	42	31	279
	26°C -30°C	56	149	153	166	164	208	245	164	1305
	>30°C	2	15	23	33	45	63	88	62	331
	Total	68	209	244	260	278	364	430	308	2161
North Region	<20°C	5	8	18	28	20	27	45	46	197
	21°C -25°C	1	5	3	2	10	7	4	1	33
	26°C -30°C	1	2	4	3	4	6	13	3	36
	>30°C	2	4	12	11	14	21	29	11	104
	Total	9	19	37	44	48	61	91	61	370
South Region	<20°C	0	0	0	0	0	0	0	0	0
	21°C -25°C	1	14	15	10	7	11	11	8	77
	26°C -30°C	49	117	113	132	116	157	167	116	967
	>30°C	0	3	2	7	1	6	6	12	37
	Total	50	134	130	149	124	174	184	136	1081
East Region	<20°C	3	6	6	1	5	10	9	4	44
	21°C -25°C	0	10	21	17	22	36	23	21	150
	26°C -30°C	6	24	26	23	37	36	53	35	240
	>30°C	0	5	8	13	20	30	46	37	159
	Total	9	45	61	54	84	112	131	97	593
West Region	<20°C	0	0	0	0	1	2	1	1	5
	21°C -25°C	0	2	5	3	4	0	4	1	19
	26°C -30°C	0	6	10	8	7	9	12	10	62
	>30°C	0	3	1	2	10	6	7	2	31
	Total	0	11	16	13	22	17	24	14	117

For peer review only

Supplementary Table 9. Variations in the intussusception cases between the different temperature categories by Kruskal-Wallis H test

Level	df	χ^2-statistic	P- value
Pooled	3	15.933	< 0.001
North Region	3	21.054	< 0.001
South Region	3	50.625	< 0.001
East Region	3	3.502	0.3205
West Region	3	5.606	0.1324

Notes: Pooled- all sites/regions combined; df: degrees of freedom; χ^2-statistic: critical chi square value

For peer review only

For peer review only - http://bmjopen.bmj.com/site/about/guidelines.xhtml

Supplementary Figure 4: Association of monthly intussusception cases with mean monthly temperatures

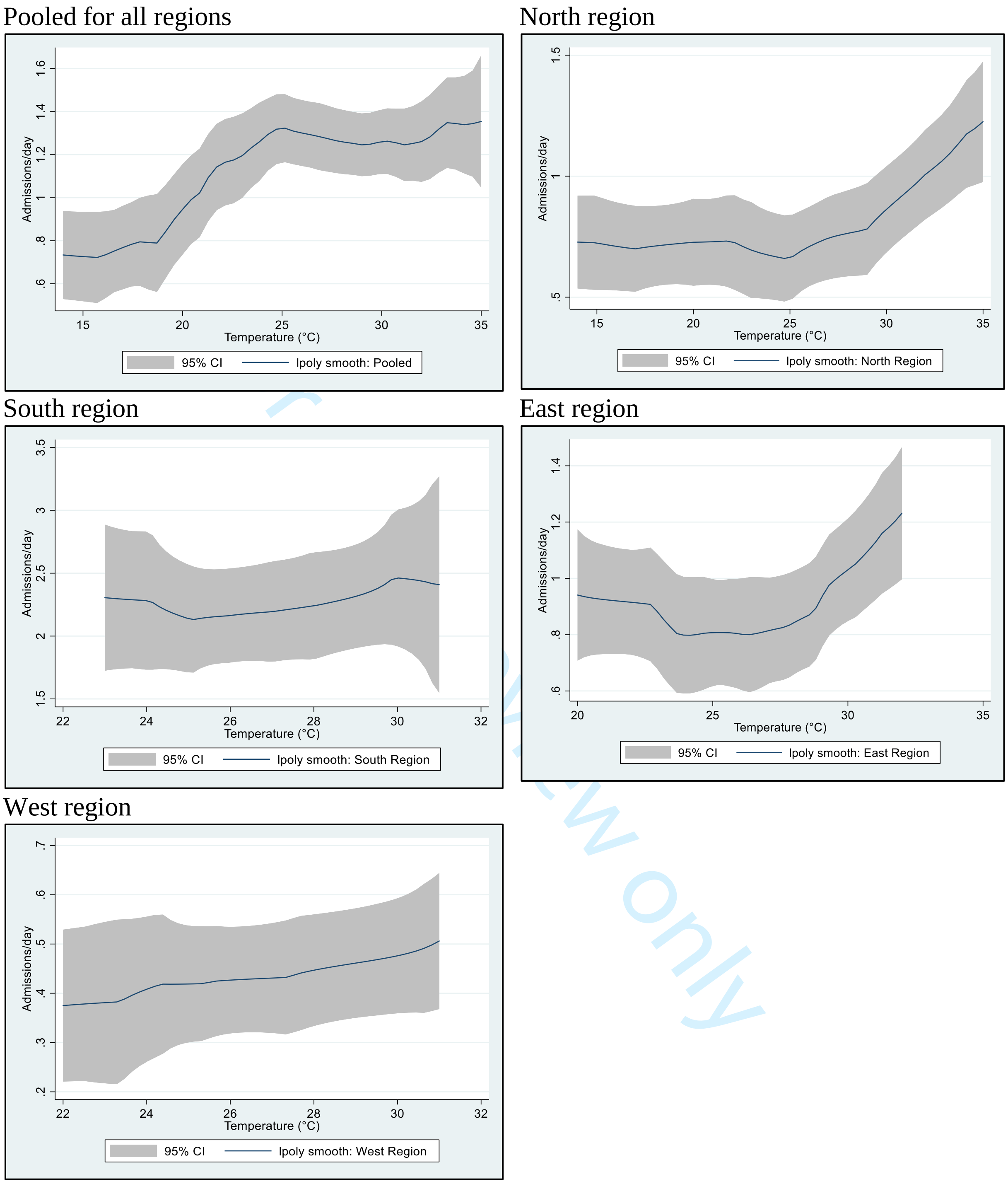

Supplementary Figure 5: Association of monthly intussusception cases with mean monthly humidity

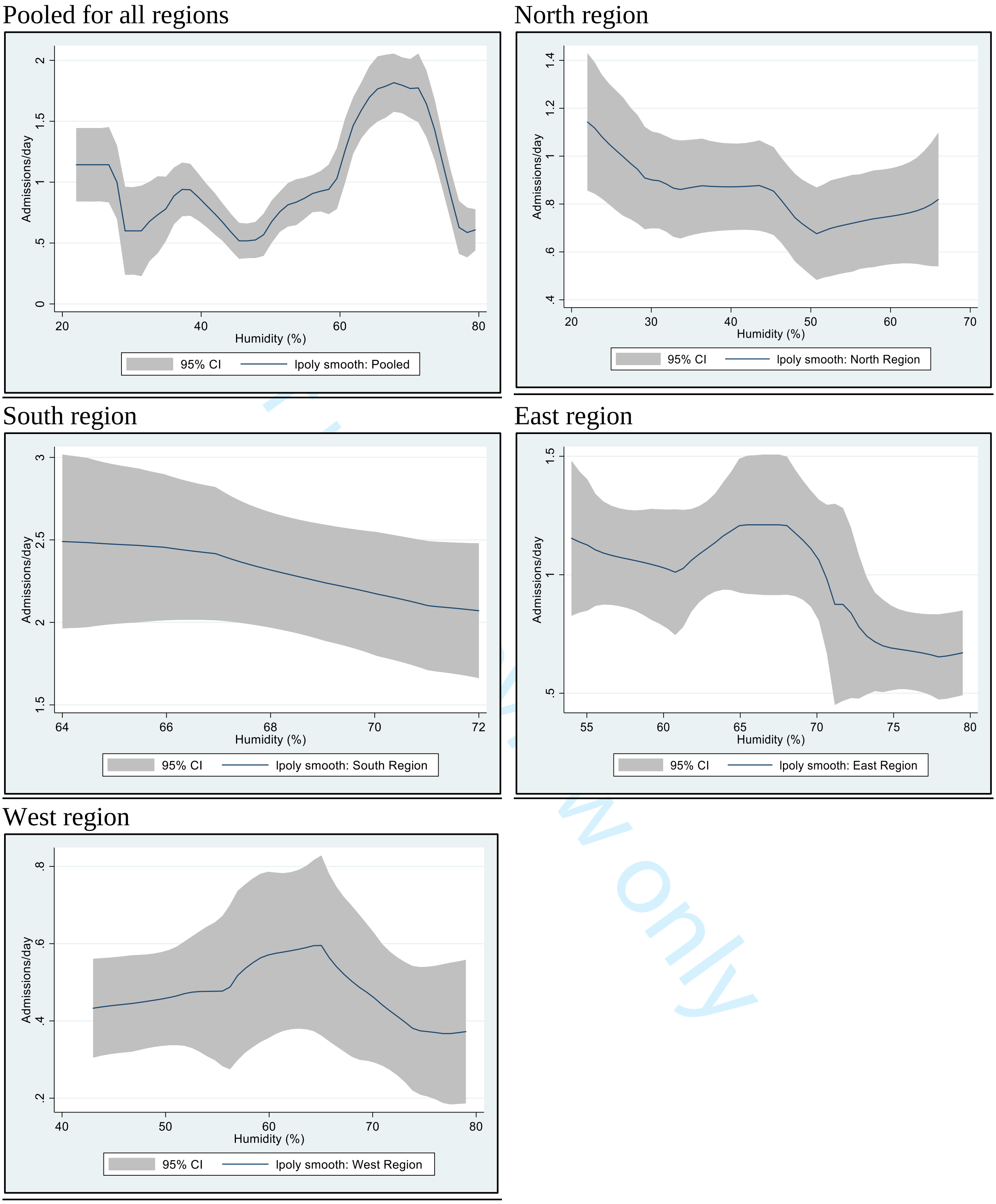

For peer review only - http://bmjopen.bmj.com/site/about/guidelines.xhtml

Supplementary Figure 6: Association of monthly intussusception cases with mean monthly wind speed

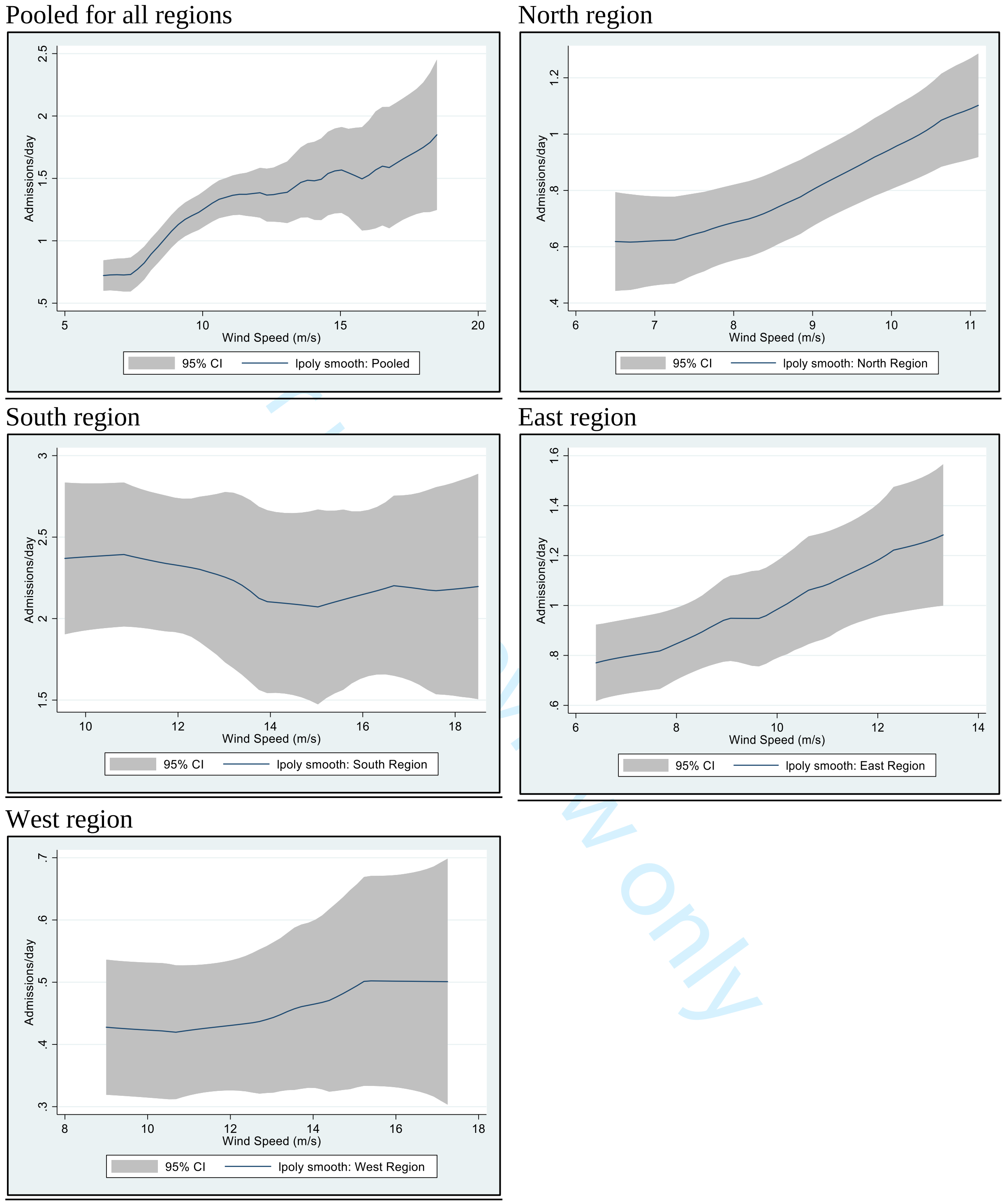

For peer review only - http://bmjopen.bmj.com/site/about/guidelines.xhtml

Supplementary Figure 7: Association of monthly intussusception cases with mean monthly sunshine

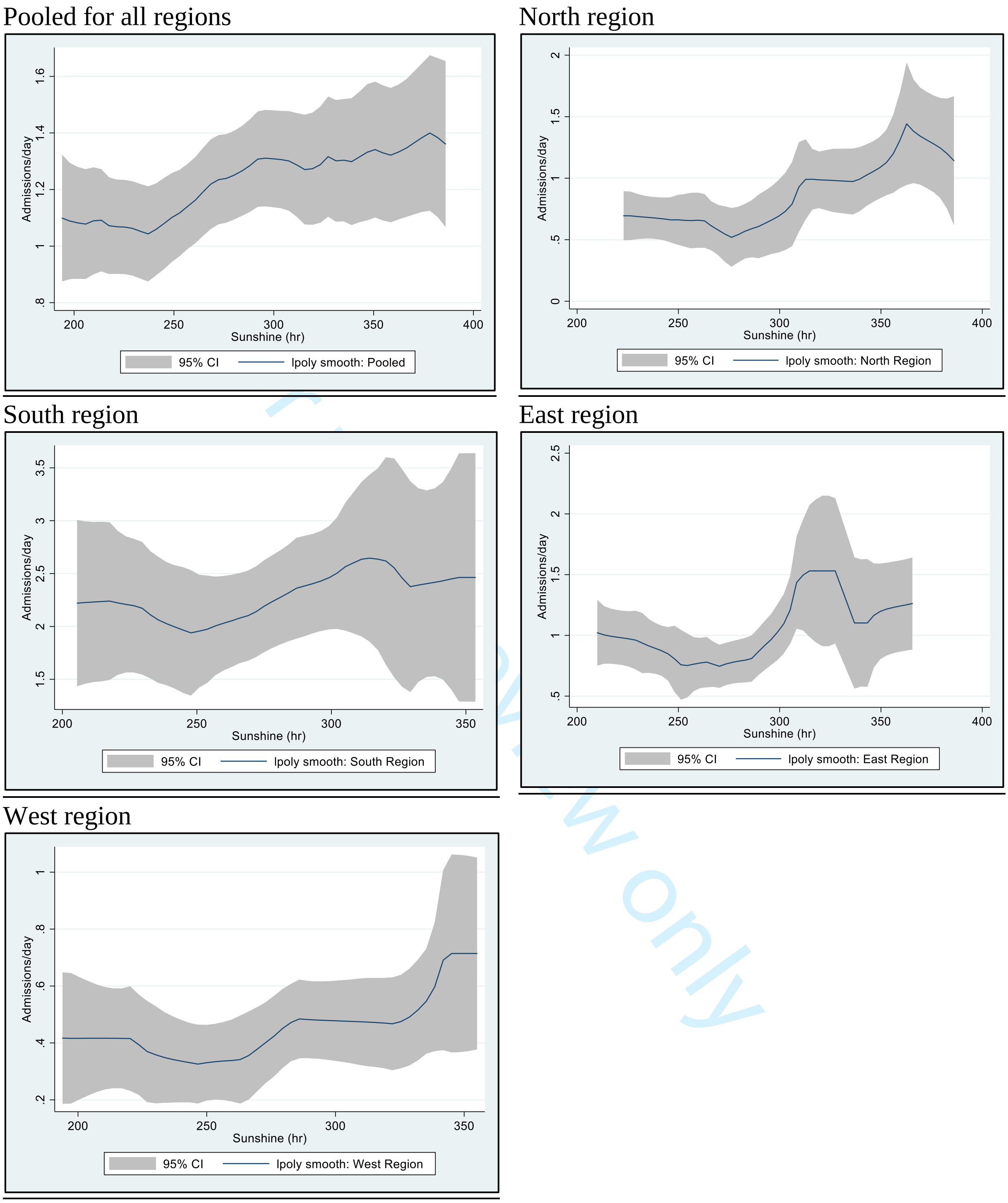

For peer review only - http://bmjopen.bmj.com/site/about/guidelines.xhtml

Supplementary Figure 8: Association of monthly intussusception cases with mean monthly rainfall

Pooled for all regions

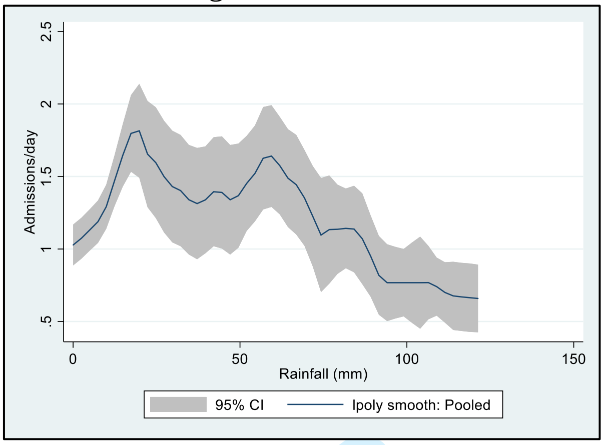

North region

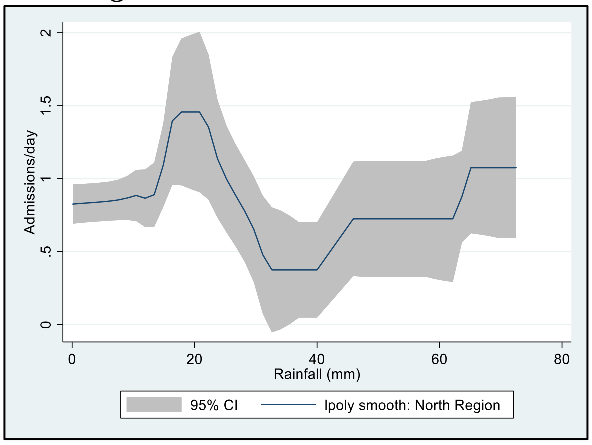

South region

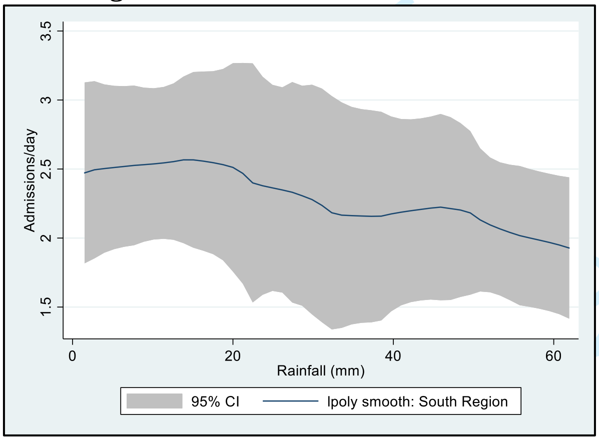

East region

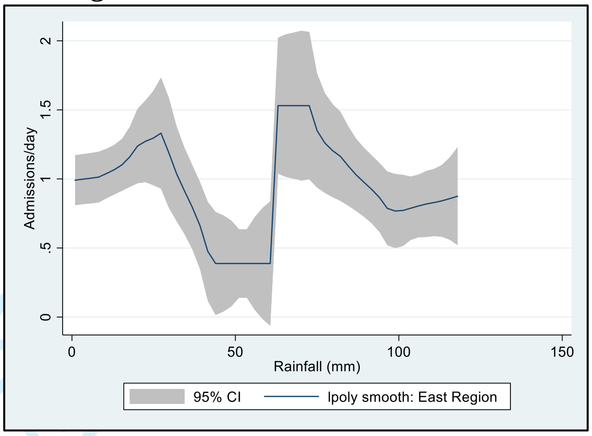

West region

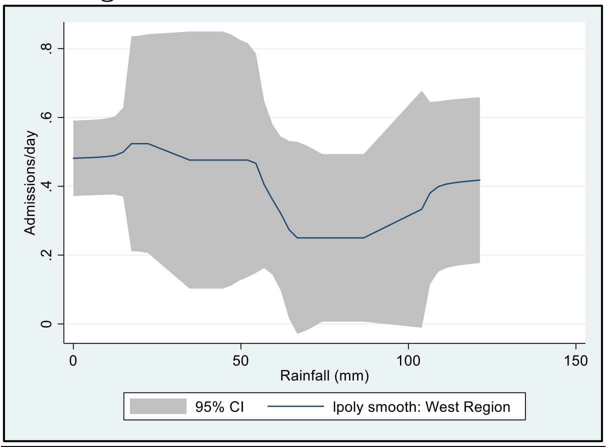

Supplementary Table 10. Factorial analysis-of-variance of intussusception in children with season and age groups

Source	Dependent variables	Type III Sum of squares	Degree of freedom	Mean square	F	p value
Corrected model	Seasonal IS cases	285.847[a]	38	7.522	1.442	**.040**
	Seasonal IS cases of 2-6 months	49.145[b]	38	1.293	1.579	**.014**
	Seasonal IS cases of 7-12 months	45.682[c]	38	1.202	1.012	.449
	Seasonal IS cases of 13-24 months	31.855[d]	38	.838	1.334	.085
Intercept	Seasonal IS cases	789.283	1	789.283	151.302	.000
	Seasonal IS cases of 2-6 months	101.336	1	101.336	123.760	.000
	Seasonal IS cases of 7-12 months	135.705	1	135.705	114.285	.000
	Seasonal IS cases of 13-24 months	40.684	1	40.684	64.740	.000
Season	Seasonal IS cases	8.674	4	2.169	.416	.797
	Seasonal IS cases of 2-6 months	1.930	4	.482	.589	.670
	Seasonal IS cases of 7-12 months	3.207	4	.802	.675	.609
	Seasonal IS cases of 13-24 months	.856	4	.214	.340	.851
Season year	Seasonal IS cases	82.483	7	11.783	2.259	**.027**
	Seasonal IS cases of 2-6 months	17.669	7	2.524	3.083	**.003**
	Seasonal IS cases of 7-12 months	3.504	7	.501	.422	.889
	Seasonal IS cases of 13-24 months	11.481	7	1.640	2.610	**.011**
Season* Season year	Seasonal IS cases	57.160	27	2.117	.406	.997
	Seasonal IS cases of 2-6 months	13.795	27	.511	.624	.934
	Seasonal IS cases of 7-12 months	19.521	27	.723	.609	.943
	Seasonal IS cases of 13-24 months	7.717	27	.286	.455	.993
Error	Seasonal IS cases	9092.547	1743	5.217		
	Seasonal IS cases of 2-6 months	1427.189	1743	.819		
	Seasonal IS cases of 7-12 months	2069.674	1743	1.187		
	Seasonal IS cases of 13-24 months	1095.327	1743	.628		
Total	Seasonal IS cases	11999.000	1782			
	Seasonal IS cases of 2-6 months	1816.000	1782			
	Seasonal IS cases of 7-12 months	2545.000	1782			
	Seasonal IS cases of 13-24 months	1272.000	1782			
Modified total	Seasonal IS cases	9378.393	1781			
	Seasonal IS cases of 2-6 months	1476.334	1781			
	Seasonal IS cases of 7-12 months	2115.356	1781			
	Seasonal IS cases of 13-24 months	1127.183	1781			

Note: a: R squared= .030 (adjusted R squared= .009); b: R squared= .033 (adjusted R squared= .012); c: R squared = .022 (adjusted R squared= .000); d: R squared= .028 (adjusted R squared= .007)

For peer review only

Supplementary Table 11. Factorial analysis-of-variance of intussusception in children with season and regions

Source	Dependent variables	Type III Sum of squares	Degree of freedom	Mean square	F	p value
Corrected model	Seasonal IS cases	285.847[a]	38	7.522	1.442	**.040**
	Seasonal IS cases of North region	36.237[b]	38	.954	1.809	**.002**
	Seasonal IS cases of South region	55.831[c]	38	1.469	.321	1.000
	Seasonal IS cases of East region	53.071[d]	38	1.397	1.333	.085
	Seasonal IS cases of West region	2.721[e]	38	.072	.662	.944
Intercept	Seasonal IS cases	789.283	1	789.283	151.302	**.000**
	Seasonal IS cases of North region	29.881	1	29.881	56.676	**.000**
	Seasonal IS cases of South region	180.247	1	180.247	39.373	**.000**
	Seasonal IS cases of East region	59.322	1	59.322	56.607	**.000**
	Seasonal IS cases of West region	2.250	1	2.250	20.798	**.000**
Season	Seasonal IS cases	8.674	4	2.169	.416	.797
	Seasonal IS cases of North region	10.274	4	2.569	4.872	**.001**
	Seasonal IS cases of South region	9.239	4	2.310	.505	.732
	Seasonal IS cases of East region	1.801	4	.450	.430	.787
	Seasonal IS cases of West region	.207	4	.052	.479	.752
Season year	Seasonal IS cases	82.483	7	11.783	2.259	**.027**
	Seasonal IS cases of North region	11.119	7	1.588	3.013	**.004**
	Seasonal IS cases of South region	5.618	7	.803	.175	.990
	Seasonal IS cases of East region	18.661	7	2.666	2.544	**.013**
	Seasonal IS cases of West region	.464	7	.066	.612	.746
Season* Season year	Seasonal IS cases	57.160	27	2.117	.406	.997
	Seasonal IS cases of North region	8.198	27	.304	.576	.960
	Seasonal IS cases of South region	28.377	27	1.051	.230	1.000
	Seasonal IS cases of East region	15.527	27	.575	.549	.971
	Seasonal IS cases of West region	1.325	27	.049	.454	.993
Error	Seasonal IS cases	9092.547	1743	5.217		
	Seasonal IS cases of North region	918.939	1743	.527		
	Seasonal IS cases of South region	7979.411	1743	4.578		
	Seasonal IS cases of East region	1826.595	1743	1.048		
	Seasonal IS cases of West region	188.597	1743	.108		
Total	Seasonal IS cases	11999.000	1782			
	Seasonal IS cases of North region	1032.000	1782			
	Seasonal IS cases of South region	8691.000	1782			
	Seasonal IS cases of East region	2077.000	1782			
	Seasonal IS cases of West region	199.000	1782			
Modified total	Seasonal IS cases	9378.393	1781			
	Seasonal IS cases of North region	955.176	1781			
	Seasonal IS cases of South region	8035.242	1781			
	Seasonal IS cases of East region	1879.666	1781			
	Seasonal IS cases of West region	191.318	1781			

Note: a: R squared = .030 (adjusted R squared= .009); b: R squared = .038 (adjusted R squared= .017); c: R squared = .007 (adjusted R squared= -.015); d: R squared= .028 (adjusted R squared= .007); e: R squared = .014 (adjusted R squared= -.007)

For peer review only - http://bmjopen.bmj.com/site/about/guidelines.xhtml

Supplementary Table 12. Multiple comparisons of seasonal intussusception in children in different seasons pooled for all sites

Dependent variable		Season (A)	Season (B)	Mean difference (A-B)	Standard error	p value	95% CI
Seasonal IS cases pooled	Tukey HSD	Monsoon	Spring	-.175	.2285	.940	-0.8 , 0.45
			Summer	-.409	.1643	.093	-0.86 , 0.04
			Monsoon	-.038	.1573	.999	-0.47 , 0.39
			Autumn	.160	.1758	.892	-0.32 , 0.64
		Spring	Winter	.175	.2285	.940	-0.45 , 0.8
			Summer	-.234	.2221	.831	-0.84 , 0.37
			Monsoon	.137	.2169	.970	-0.46 , 0.73
			Autumn	.336	.2307	.592	-0.29 , 0.97
		Summer	Winter	.409	.1643	.093	-0.04 , 0.86
			Spring	.234	.2221	.831	-0.37 , 0.84
			Monsoon	.371	.1478	.089	-0.03 , 0.77
			Autumn	.569*	.1673	**.006**	0.11 , 1.03
		Monsoon	Winter	.038	.1573	.999	-0.39 , 0.47
			Spring	-.137	.2169	.970	-0.73 , 0.46
			Summer	-.371	.1478	.089	-0.77 , 0.03
			Autumn	.199	.1604	.729	-0.24 , 0.64
		Autumn	Winter	-.160	.1758	.892	-0.64 , 0.32
			Spring	-.336	.2307	.592	-0.97 , 0.29
			Summer	-.569*	.1673	**.006**	-1.03 , -0.11
			Monsoon	-.199	.1604	.729	-0.64 , 0.24

*Note: * The difference is significant at p<0.05 level.*

For peer review only

For peer review only - http://bmjopen.bmj.com/site/about/guidelines.xhtml

Supplementary Table 13. Multiple comparisons of seasonal intussusception in children in different seasons at regional level

Dependent variable		Season (A)	Season (B)	Mean difference (A-B)	Standard error	p value	95% CI
North Region	Tukey HSD	Winter	Spring	-.151	.0726	.231	-0.35 , 0.05
			Summer	-.039	.0522	.943	-0.18 , 0.1
			Monsoon	.125	.0500	.089	-0.01 , 0.26
			Autumn	.139	.0559	.093	-0.01 , 0.29
		Spring	Winter	.151	.0726	.231	-0.05 , 0.35
			Summer	.111	.0706	.512	-0.08 , 0.3
			Monsoon	.276*	.0690	**.001**	0.09 , 0.46
			Autumn	.290*	.0733	**.001**	0.09 , 0.49
		Summer	Winter	.039	.0522	.943	-0.1 , 0.18
			Spring	-.111	.0706	.512	-0.3 , 0.08
			Monsoon	.165*	.0470	**.004**	0.04 , 0.29
			Autumn	.179*	.0532	**.007**	0.03 , 0.32
		Monsoon	Winter	-.125	.0500	.089	-0.26 , 0.01
			Spring	-.276*	.0690	**.001**	-0.46 , -0.09
			Summer	-.165*	.0470	**.004**	-0.29 , -0.04
			Autumn	.014	.0510	.999	-0.13 , 0.15
		Autumn	Winter	-.139	.0559	.093	-0.29 , 0.01
			Spring	-.290*	.0733	**.001**	-0.49 , -0.09
			Summer	-.179*	.0532	**.007**	-0.32 , -0.03
			Monsoon	-.014	.0510	.999	-0.15 , 0.13
South Region	Tukey HSD	Winter	Spring	-.014	.2140	1.000	-0.6 , 0.57
			Summer	-.197	.1539	.705	-0.62 , 0.22
			Monsoon	-.141	.1473	.875	-0.54 , 0.26
			Autumn	.032	.1647	1.000	-0.42 , 0.48
		Spring	Winter	.014	.2140	1.000	-0.57 , 0.6
			Summer	-.183	.2080	.905	-0.75 , 0.39
			Monsoon	-.127	.2032	.971	-0.68 , 0.43
			Autumn	.046	.2161	1.000	-0.54 , 0.64
		Summer	Winter	.197	.1539	.705	-0.22 , 0.62
			Spring	.183	.2080	.905	-0.39 , 0.75
			Monsoon	.056	.1384	.994	-0.32 , 0.43
			Autumn	.229	.1568	.588	-0.2 , 0.66
		Monsoon	Winter	.141	.1473	.875	-0.26 , 0.54
			Spring	.127	.2032	.971	-0.43 , 0.68
			Summer	-.056	.1384	.994	-0.43 , 0.32
			Autumn	.173	.1503	.779	-0.24 , 0.58
		Autumn	Winter	-.032	.1647	1.000	-0.48 , 0.42
			Spring	-.046	.2161	1.000	-0.64 , 0.54
			Summer	-.229	.1568	.588	-0.66 , 0.2
			Monsoon	-.173	.1503	.779	-0.58 , 0.24
East region	Tukey HSD	Winter	Spring	.035	.1024	.997	-0.24 , 0.31
			Summer	-.125	.0736	.433	-0.33 , 0.08
			Monsoon	-.001	.0705	1.000	-0.19 , 0.19

For peer review only - http://bmjopen.bmj.com/site/about/guidelines.xhtml

			Autumn	.010	.0788	1.000	-0.21 , 0.23
		Spring	Winter	-.035	.1024	.997	-0.31 , 0.24
			Summer	-.160	.0995	.490	-0.43 , 0.11
			Monsoon	-.036	.0972	.996	-0.3 , 0.23
			Autumn	-.025	.1034	.999	-0.31 , 0.26
		Summer	Winter	.125	.0736	.433	-0.08 , 0.33
			Spring	.160	.0995	.490	-0.11 , 0.43
			Monsoon	.124	.0662	.331	-0.06 , 0.31
			Autumn	.135	.0750	.372	-0.07 , 0.34
		Monsoon	Winter	.001	.0705	1.000	-0.19 , 0.19
			Spring	.036	.0972	.996	-0.23 , 0.3
			Summer	-.124	.0662	.331	-0.31 , 0.06
			Autumn	.011	.0719	1.000	-0.19 , 0.21
		Autumn	Winter	-.010	.0788	1.000	-0.23 , 0.21
			Spring	.025	.1034	.999	-0.26 , 0.31
			Summer	-.135	.0750	.372	-0.34 , 0.07
			Monsoon	-.011	.0719	1.000	-0.21 , 0.19
West region	Tukey HSD	Winter	Spring	-.046	.0329	.637	-0.14 , 0.04
			Summer	-.048	.0237	.260	-0.11 , 0.02
			Monsoon	-.022	.0227	.872	-0.08 , 0.04
			Autumn	-.021	.0253	.921	-0.09 , 0.05
		Spring	Winter	.046	.0329	.637	-0.04 , 0.14
			Summer	-.002	.0320	1.000	-0.09 , 0.09
			Monsoon	.024	.0312	.941	-0.06 , 0.11
			Autumn	.025	.0332	.947	-0.07 , 0.12
		Summer	Winter	.048	.0237	.260	-0.02 , 0.11
			Spring	.002	.0320	1.000	-0.09 , 0.09
			Monsoon	.026	.0213	.743	-0.03 , 0.08
			Autumn	.027	.0241	.805	-0.04 , 0.09
		Monsoon	Winter	.022	.0227	.872	-0.04 , 0.08
			Spring	-.024	.0312	.941	-0.11 , 0.06
			Summer	-.026	.0213	.743	-0.08 , 0.03
			Autumn	.001	.0231	1.000	-0.06 , 0.06
		Autumn	Winter	.021	.0253	.921	-0.05 , 0.09
			Spring	-.025	.0332	.947	-0.12 , 0.07
			Summer	-.027	.0241	.805	-0.09 , 0.04
			Monsoon	-.001	.0231	1.000	-0.06 , 0.06

*Note: * The difference is significant at p<0.05 level.*

For peer review only

Supplementary Table 14. Multiple comparisons of seasonal intussusception in children in different seasons for different age groups

Dependent variable		Season (A)	Season (B)	Mean difference (A-B)	Standard error	p value	95% CI
Seasonal IS cases in children aged 2-6 months	Tukey HSD	Winter	Spring	-.003	.0905	1.000	-0.25 , 0.24
			Summer	-.073	.0651	.798	-0.25 , 0.11
			Monsoon	.019	.0623	.998	-0.15 , 0.19
			Autumn	.123	.0696	.395	-0.07 , 0.31
		Spring	Winter	.003	.0905	1.000	-0.24 , 0.25
			Summer	-.070	.0880	.933	-0.31 , 0.17
			Monsoon	.022	.0859	.999	-0.21 , 0.26
			Autumn	.126	.0914	.643	-0.12 , 0.38
		Summer	Winter	.073	.0651	.798	-0.11 , 0.25
			Spring	.070	.0880	.933	-0.17 , 0.31
			Monsoon	.092	.0585	.517	-0.07 , 0.25
			Autumn	.195*	.0663	**.027**	0.01 , 0.38
		Monsoon	Winter	-.019	.0623	.998	-0.19 , 0.15
			Spring	-.022	.0859	.999	-0.26 , 0.21
			Summer	-.092	.0585	.517	-0.25 , 0.07
			Autumn	.104	.0636	.478	-0.07 , 0.28
		Autumn	Winter	-.123	.0696	.395	-0.31 , 0.07
			Spring	-.126	.0914	.643	-0.38 , 0.12
			Summer	-.195*	.0663	**.027**	-0.38 , -0.01
			Monsoon	-.104	.0636	.478	-0.28 , 0.07
Seasonal IS cases in children aged 7-12 months	Tukey HSD	Winter	Spring	-.185	.1090	.439	-0.48 , 0.11
			Summer	-.210	.0784	.057	-0.42 , 0
			Monsoon	-.046	.0750	.973	-0.25 , 0.16
			Autumn	.021	.0839	.999	-0.21 , 0.25
		Spring	Winter	.185	.1090	.439	-0.11 , 0.48
			Summer	-.026	.1059	.999	-0.32 , 0.26
			Monsoon	.138	.1035	.667	-0.14 , 0.42
			Autumn	.206	.1101	.336	-0.1 , 0.51
		Summer	Winter	.210	.0784	.057	0 , 0.42
			Spring	.026	.1059	.999	-0.26 , 0.32
			Monsoon	.164	.0705	.136	-0.03 , 0.36
			Autumn	.231*	.0798	**.031**	0.01 , 0.45
		Monsoon	Winter	.046	.0750	.973	-0.16 , 0.25
			Spring	-.138	.1035	.667	-0.42 , 0.14
			Summer	-.164	.0705	.136	-0.36 , 0.03
			Autumn	.067	.0765	.906	-0.14 , 0.28
		Autumn	Winter	-.021	.0839	.999	-0.25 , 0.21
			Spring	-.206	.1101	.336	-0.51 , 0.1
			Summer	-.231*	.0798	**.031**	-0.45 , -0.01
			Monsoon	-.067	.0765	.906	-0.28 , 0.14
Seasonal IS cases in	Tukey HSD	Winter	Spring	.012	.0793	1.000	-0.2 , 0.23
			Summer	-.126	.0570	.176	-0.28 , 0.03
			Monsoon	-.011	.0546	1.000	-0.16 , 0.14

For peer review only - http://bmjopen.bmj.com/site/about/guidelines.xhtml

children aged 7-12 months			Autumn	.016	.0610	.999	-0.15 , 0.18
		Spring	Winter	-.012	.0793	1.000	-0.23 , 0.2
			Summer	-.138	.0771	.378	-0.35 , 0.07
			Monsoon	-.024	.0753	.998	-0.23 , 0.18
			Autumn	.004	.0801	1.000	-0.21 , 0.22
		Summer	Winter	.126	.0570	.176	-0.03 , 0.28
			Spring	.138	.0771	.378	-0.07 , 0.35
			Monsoon	.115	.0513	.167	-0.03 , 0.25
			Autumn	.143	.0581	.102	-0.02 , 0.3
		Monsoon	Winter	.011	.0546	1.000	-0.14 , 0.16
			Spring	.024	.0753	.998	-0.18 , 0.23
			Summer	-.115	.0513	.167	-0.25 , 0.03
			Autumn	.028	.0557	.987	-0.12 , 0.18
		Autumn	Winter	-.016	.0610	.999	-0.18 , 0.15
			Spring	-.004	.0801	1.000	-0.22 , 0.21
			Summer	-.143	.0581	.102	-0.3 , 0.02
			Monsoon	-.028	.0557	.987	-0.18 , 0.12

*Note: * The difference is significant at p<0.05 level.*

For peer review only

Title: Association of meteorological parameters with intussusception in children aged under-two years: Results from a multisite bidirectional surveillance over seven years in India

Corresponding author: Manoja Kumar Das

The IRBS reviewed and approved the study protocol titled "Intussusception in Infants: Multisite Retrospective Surveillance in India"

1. Name of IRB: The INCLEN Independent Ethic Committee
   Name of the Institute: The INCLEN Trust International, New Delhi, India
   Principal Investigator's Institute/Coordinating Institute

2. Name of IRB: The INCLEN Independent Ethic Committee
   Name of the Institute: King George's Medical University, Lucknow, Uttar Pradesh, India

3. Name of IRB: Institutional Ethics Committee
   Name of the Institute: MP Shah Government Medical College, Jamnagar, Gujarat, India

4. Name of IRB: Institutional Ethics Committee
   Name of the Institute: Sher-I-Kashmir Institute of Medical Sciences, Srinagar, Jammu & Kashmir, India

5. Name of IRB: Institutional Ethics Committee
   Name of the Institute: Gauhati Medical College, Guwahati, Assam, India

6. Name of IRB: Institutional Human Ethics Committee
   Name of the Institute: PSG Institute of Medical Sciences, Coimbatore, Tamil Nadu, India

7. Name of IRB: Institutional Ethics Committee
   Name of the Institute: King George Hospital, Andhra Medical College, Vishakhapatnam, Andhra Pradesh, India

8. Name of IRB: Institutional Ethics Committee
   Name of the Institute: Fortis Escorts Hospital, Jaipur, Rajasthan, India

9. Name of IRB: Institutional Ethics Committee
   Name of the Institute: Grant Medical College & JJ Hospital, Mumbai, Maharashtra, India

10. Name of IRB: Institutional Ethics Committee
    Name of the Institute: Government Medical College & SAT Hospital, Thiruvananthapuram, Kerala, India

11. Name of IRB: Medanta Institutional Ethics Committee
    Name of the Institute: Medanta—The Medicity, Gurgaon, Haryana, India

12. Name of IRB: Institutional Ethics Committee
    Name of the Institute: SCB Medical College, Cuttack, Odisha, India

13. Name of IRB: Institutional Ethics Committee

For peer review only

Name of the Institute: IMS & SUM Medical College & Hospital, Bhubaneswar, Odisha, India

14. Name of IRB: Institutional Ethics Committee
    Name of the Institute: Choithram Hospital and Research Centre, Indore, Madhya Pradesh, India
15. Name of IRB: Institutional Ethics Committee
    Name of the Institute: Agartala Government Medical College, Agartala, Tripura, India

16. Name of IRB: Institutional Ethics Committee- Clinical Studies
    Name of the Institute: Apollo Hospitals, Chennai, Tamil Nadu

17. Name of IRB: Institutional Ethics Committee
    Name of the Institute: Maulana Azad Medical College, Delhi, India

18. Name of IRB: Institutional Ethics Committee
    Name of the Institute: Institute of Post Graduate Medical Education and Research, Kolkata, West Bengal, India

19. Name of IRB: Institutional Ethics Committee
    Name of the Institute: Indira Gandhi Institute of Medical Sciences, Patna, Bihar, India

20. Name of IRB: Ethics Committee
    Name of the Institute: Apollo Hospital, Hyderabad, Telengana, India

21. Name of IRB: Institutional Ethics Committee
    Name of the Institute: MKCG Medical College, Berhampur, Odisha, India

For peer review only

Manuscript Title: Intussusception in children under-two years, seasonal variations and associations with meteorological parameters: Results from multisite bidirectional surveillance in India over eight years

STROBES Checklist

Section/Topic	Item #	Recommendation	Reported on page #
**Title and abstract**	1	(*a*) Indicate the study's design with a commonly used term in the title or the abstract	1
		(*b*) Provide in the abstract an informative and balanced summary of what was done and what was found	1-2
**Introduction**			
Background/rationale	2	Explain the scientific background and rationale for the investigation being reported	4
Objectives	3	State specific objectives, including any pre-specified hypotheses	5
**Methods**			
Study design	4	Present key elements of study design early in the paper	5
Setting	5	Describe the setting, locations, and relevant dates, including periods of recruitment, exposure, follow-up, and data collection	5
Participants	6	(*a*) *Cohort study*—Give the eligibility criteria, and the sources and methods of selection of participants. Describe methods of follow-up<br>*Case-control study*—Give the eligibility criteria, and the sources and methods of case ascertainment and control selection. Give the rationale for the choice of cases and controls<br>*Cross-sectional study*—Give the eligibility criteria, and the sources and methods of selection of participants	5
		(*b*) *Cohort study*—For matched studies, give matching criteria and number of exposed and unexposed<br>*Case-control study*—For matched studies, give matching criteria and the number of controls per case	
Variables	7	Clearly define all outcomes, exposures, predictors, potential confounders, and effect modifiers. Give diagnostic criteria, if applicable	5-6
Data sources/ measurement	8*	For each variable of interest, give sources of data and details of methods of assessment (measurement). Describe comparability of assessment methods if there is more than one group	5-6
Bias	9	Describe any efforts to address potential sources of bias	6

For peer review only

Study size	10	Explain how the study size was arrived at	5-6
Quantitative variables	11	Explain how quantitative variables were handled in the analyses. If applicable, describe which groupings were chosen and why	6
Statistical methods	12	(*a*) Describe all statistical methods, including those used to control for confounding	6-7
		(*b*) Describe any methods used to examine subgroups and interactions	6-7
		(*c*) Explain how missing data were addressed	6
		(*d*) *Cohort study*—If applicable, explain how loss to follow-up was addressed<br>*Case-control study*—If applicable, explain how matching of cases and controls was addressed<br>*Cross-sectional study*—If applicable, describe analytical methods taking account of sampling strategy	6-7
		(*e*) Describe any sensitivity analyses	6-7
**Results**			
Participants	13*	(a) Report numbers of individuals at each stage of study—eg numbers potentially eligible, examined for eligibility, confirmed eligible, included in the study, completing follow-up, and analysed	7
		(b) Give reasons for non-participation at each stage	NA
		(c) Consider use of a flow diagram	Narration given
Descriptive data	14*	(a) Give characteristics of study participants (eg demographic, clinical, social) and information on exposures and potential confounders	7-8
		(b) Indicate number of participants with missing data for each variable of interest	7-8 and tables
		(c) *Cohort study*—Summarise follow-up time (eg, average and total amount)	
Outcome data	15*	*Cohort study*—Report numbers of outcome events or summary measures over time	7-11<br>Given in tables and results
		Case-control study—Report numbers in each exposure category, or summary measures of exposure	
		Cross-sectional study—Report numbers of outcome events or summary measures	
Main results	16	(*a*) Give unadjusted estimates and, if applicable, confounder-adjusted estimates and their precision (eg, 95% confidence interval). Make clear which confounders were adjusted for and why they were included	7-11<br>Given in tables and results
		(*b*) Report category boundaries when continuous variables were categorized	7-11<br>Given in tables and

For peer review only

			results
		(*c*) If relevant, consider translating estimates of relative risk into absolute risk for a meaningful time period	NA
Other analyses	17	Report other analyses done—eg analyses of subgroups and interactions, and sensitivity analyses	7-11<br>Given in tables and results
**Discussion**			
Key results	18	Summarise key results with reference to study objectives	11-12
Limitations	19	Discuss limitations of the study, taking into account sources of potential bias or imprecision. Discuss both direction and magnitude of any potential bias	14
Interpretation	20	Give a cautious overall interpretation of results considering objectives, limitations, multiplicity of analyses, results from similar studies, and other relevant evidence	11-14
Generalisability	21	Discuss the generalisability (external validity) of the study results	14
**Other information**			
Funding	22	Give the source of funding and the role of the funders for the present study and, if applicable, for the original study on which the present article is based	17

*Give information separately for cases and controls in case-control studies and, if applicable, for exposed and unexposed groups in cohort and cross-sectional studies.

Note: An Explanation and Elaboration article discusses each checklist item and gives methodological background and published examples of transparent reporting. The STROBE checklist is best used in conjunction with this article (freely available on the Web sites of PLoS Medicine at http://www.plosmedicine.org/, Annals of Internal Medicine at http://www.annals.org/, and Epidemiology at http://www.epidem.com/). Information on the STROBE Initiative is available at www.strobe-statement.org.

For peer review only

BMJ Open

Association of meteorological parameters with intussusception in children aged under-two years: Results from a multisite bidirectional surveillance over seven years in India

Journal:	*BMJ Open*
Manuscript ID	bmjopen-2020-043687.R2
Article Type:	Original research
Date Submitted by the Author:	08-Feb-2021
Complete List of Authors:	Study Group, The INCLEN Intussusception Surveillance; The INCLEN Trust International<br>Das, Manoja; The INCLEN Trust International
**Primary Subject Heading**:	Public health
Secondary Subject Heading:	Epidemiology, Paediatrics
Keywords:	EPIDEMIOLOGY, Public health < INFECTIOUS DISEASES, Community child health < PAEDIATRICS, Paediatric gastroenterology < PAEDIATRICS

SCHOLARONE™
Manuscripts

I, the Submitting Author has the right to grant and does grant on behalf of all authors of the Work (as defined in the below author licence), an exclusive licence and/or a non-exclusive licence for contributions from authors who are: i) UK Crown employees; ii) where BMJ has agreed a CC-BY licence shall apply, and/or iii) in accordance with the terms applicable for US Federal Government officers or employees acting as part of their official duties; on a worldwide, perpetual, irrevocable, royalty-free basis to BMJ Publishing Group Ltd ("BMJ") its licensees and where the relevant Journal is co-owned by BMJ to the co-owners of the Journal, to publish the Work in this journal and any other BMJ products and to exploit all rights, as set out in our licence.

The Submitting Author accepts and understands that any supply made under these terms is made by BMJ to the Submitting Author unless you are acting as an employee on behalf of your employer or a postgraduate student of an affiliated institution which is paying any applicable article publishing charge ("APC") for Open Access articles. Where the Submitting Author wishes to make the Work available on an Open Access basis (and intends to pay the relevant APC), the terms of reuse of such Open Access shall be governed by a Creative Commons licence – details of these licences and which Creative Commons licence will apply to this Work are set out in our licence referred to above.

Other than as permitted in any relevant BMJ Author's Self Archiving Policies, I confirm this Work has not been accepted for publication elsewhere, is not being considered for publication elsewhere and does not duplicate material already published. I confirm all authors consent to publication of this Work and authorise the granting of this licence.

For peer review only

Article Type: Original Research

Revised Title: Association of meteorological parameters with intussusception in children aged under-two years: Results from a multisite bidirectional surveillance over seven years in India

Short title: Association of intussusception in Indian children with meteorological parameters

Author Information

The INCLEN Intussusception Surveillance Network Study Group

The INCLEN Trust International, Okhla Industrial Area, Phase 1, New Delhi -110020, India

E-mail: drmanojkdas@gmail.com

Corresponding Author

Manoja Kumar Das

Director Projects, The INCLEN Trust International, F1/5, Okhla Industrial Area, Phase 1, New Delhi -110020, INDIA

Phone: Off: +91-11-47730000; Mob: +91-9810203768; Fax: +91-11-47730001

E-mail: manoj@inclentrust.org

For peer review only

Author information

'The INCLEN Intussusception Surveillance Network Study Group' list of authors

1. Manoja Kumar Das

Director Projects, The INCLEN Trust International, New Delhi, India

Email: manoj@inclentrust.org

2. Narendra Kumar Arora

Executive Director, The INCLEN Trust International, New Delhi, India

Email: nkarora@inclentrust.org

3. Bini Gupta

Assistant Research Officer, The INCLEN Trust International, New Delhi, India

Email: drbini.gupta02@gmail.com

4. Sharad Srivastava

Assistant Program Officer, The INCLEN Trust International, New Delhi, India

Email: sharad.srivastav@inclentrust.org

5. Apoorva Sharan

Research Officer, The INCLEN Trust International, New Delhi, India

Email: apoorva@inclentrust.org

6. Arindam Ray

Senior Program Officer, Bill and Melinda Gates Foundation, India Country Office, New Delhi, India

Email: arindam.ray@gatesfoundation.org

7. Ashish Wakhlu

Professor, Department of Paediatric Surgery, King George's Medical University, Lucknow, Uttar Pradesh, India

Email: ashish_wakhlu@hotmail.com

For peer review only - http://bmjopen.bmj.com/site/about/guidelines.xhtml

8. Bhadresh R Vyas

Professor, Department of Paediatrics, MP Shah Government Medical College, Jamnagar, Gujarat, India

Email: bhadreshrvyas@yahoo.co.uk

9. Javeed Iqbal Bhat

Assistant Professor, Department of Paediatrics, Sher-I-Kashmir Institute of Medical Sciences, Srinagar, Jammu & Kashmir, India

Email: drjaveediqbal@gmail.com

10. Jayanta K. Goswami

Professor, Department of Paediatric Surgery, Gauhati Medical College, Guwahati, Assam, India

Email: jayantagoswami@hotmail.com

11. John Mathai

Professor, Department of Paediatrics, PSG Institute of Medical Sciences, Coimbatore, Tamil Nadu, India

Email: psg_peds@yahoo.com

12. Kameswari K.

Professor, Department of Paediatric Surgery, Andhra Medical College, Vishakhapatnam, Andhra Pradesh, India

Email: kameswari1956@gmail.com

13. Lalit Bharadia

Consultant Paediatric Gastroenterologist, Fortis Escorts Hospital, Jaipur, Rajasthan, India

Email: lalitbharadia@gmail.com

14. Lalit Sankhe

For peer review only

Assistant Professor, Department of Community Medicine, Grant Medical College & JJ Hospital, Mumbai, Maharashtra, India

Email: sankhelalit@yahoo.com

15. Ajayakumar M.K.

Professor, Department of Paediatric Surgery, Government Medical College & SAT Hospital, Thiruvananthapuram, Kerala, India

Email: drajaykumarmk@gmail.com

16. Neelam Mohan

Consultant Paediatrics Gastroenterology, Medanta—The Medicity, Gurgaon, Haryana, India

Email: drneelam@yahoo.com

17. Pradeep K. Jena

Professor, Department of Paediatric Surgery, SCB Medical College, Cuttack, Odisha, India

Email: drpkjena@gmail.com

18. Rachita Sarangi

Professor, Department of Paediatrics, IMS & SUM Medical College & Hospital, Bhubaneswar, Odisha, India

Email: rachitapaedia@gmail.com

19. Rashmi Shad

Consultant Paediatrics, Choithram Hospital and Research Centre, Indore, Madhya Pradesh, India

Email: drrashmishad@yahoo.com

20. Sanjib K. Debbarma

Associate Professor, Department of Paediatrics, Agartala Government Medical College, Agartala, Tripura, India

For peer review only

Email: dr_sanjibdb@rediffmail.com

21. Shyamala J.

Consultant Paediatrics, Apollo Hospitals, Chennai, Tamil Nadu

Email: jaymoorthy@hotmail.com

22. Simmi K. Ratan

Professor, Department of Paediatric Surgery, Maulana Azad Medical College, Delhi, India

Email: drjohnsimmi@yahoo.com

23. Suman Sarkar

Assistant Professor, Department of Paediatrics, Institute of Post Graduate Medical Education and Research, Kolkata, West Bengal, India

Email: dr.sumansarkar@gmail.com

24. Vijayendra Kumar

Professor, Department of Paediatric Surgery, Indira Gandhi Institute of Medical Sciences, Patna, Bihar, India

Email: drvijayendrakr@rediffmail.com

25. Narendra Behera

Professor, Department of Pediatrics, MKCG Medical College, Berhampur, Odisha, India

Email: naribehera@yahoo.co.in

26. Rajamani G.

Professor, Department of Paediatric Surgery, Coimbatore Medical College, Coimbatore, Tamil Nadu, India

E mail: drgrajamani@gmail.com

27. Anand P. Dubey

Professor, Department of Paediatrics, Maulana Azad Medical College, Delhi, India

Email: Anand_dubey52@hotmail.com

For peer review only

28. Atul Gupta

Consultant Paediatric Surgery, Fortis Escorts Hospital, Jaipur, Rajasthan, India

Email: atulgupta26878@gmail.com

29. Cenita J. Sam

Professor, Department of Paediatric Surgery, PSG Institute of Medical Sciences, Coimbatore, Tamil Nadu, India

Email: cenitasivamani@yahoo.com

30. Gowhar Nazir Mufti

Assistant Professor, Department of Paediatric Surgery, Sher-I-Kashmir Institute of Medical Sciences, Srinagar, Jammu & Kashmir, India

Email: gowharmufti@yahoo.co.in

31. Harsh Trivedi

Professor, Department of Paediatric Surgery, MP Shah Government Medical College, Jamnagar, Gujarat, India

Email: trivediharsh@indiatimes.com

32. Jimmy Shad

Consultant Paediatric Surgery, Apollo Hospitals, Chennai, Tamil Nadu, India

Email: shadjimmy@yahoo.co.in

33. Kaushik Lahiri

Consultant, Department of Paediatric Surgery, Gauhati Medical College, Guwahati, Assam, India

Email: Kaushik_1671979@rediffmail.com

34. Krishnaswamy R

Chief Medical Officer, Masonic Children's Hospital, Coimbatore, Tamil Nadu, India

Email: krishnaswamy24@yahoo.com

For peer review only

For peer review only - http://bmjopen.bmj.com/site/about/guidelines.xhtml

35. Meera Luthra

Consultant Paediatric Surgery, Medanta- The Medicity, Gurgaon, Haryana, India

Email: meera.luthra@medanta.org

36. Padmalatha P

Professor, Department of Paediatrics, Andhra Medical College, Vishakhapatnam, Andhra Pradesh, India

Email: padmap_2000@yahoo.com

37. Rakesh Kumar

Associate Professor, Department of Paediatrics, Indira Gandhi Institute of Medical Sciences, Patna, Bihar, India

Email: drjaiswalrakesh@gmail.com

38. Ruchirendu Sarkar

Professor, Department of Paediatric Surgery, Institute of Post Graduate Medical Education and Research, Kolkata, West Bengal, India

Email: ruchirendu@gmail.com

39. Santosh Kumar A.

Professor, Department of Paediatrics, Government Medical College & SAT Hospital, Thiruvananthapuram, Kerala, India

Email: hod.pediatrics.sath@gmail.com

40. Subrat Kumar Sahoo

Associate Professor, Department of Paediatric Surgery, IMS & SUM Medical College & Hospital, Bhubaneswar, Odisha, India

Email: dr_subratsahoo@yahoo.com

41. Sunil K. Ghosh

For peer review only - http://bmjopen.bmj.com/site/about/guidelines.xhtml

Associate Professor, Department of Pediatric Surgery, Agartala Government Medical College, Agartala, Tripura, India

Email: sunilkrghosh752@gmail.com

42. Sushant Mane

Assistant Professor, Department of Paediatrics, Grant Medical College & JJ Hospital, Mumbai, Maharashtra, India

Email: drsush2006@gmail.com

43. Arun Dash

Professor, Department of Pediatric Surgery, MKCG Medical College, Berhampur, Odisha

Email: dasharun@gmail.com

44. Bashir Ahmad Charoo

Professor, Department of Paediatrics, Sher-I-Kashmir Institute of Medical Sciences, Srinagar, Jammu & Kashmir, India

Email: charoobash@gmail.com

45. Bikasha Bihary Tripathy

Associate Professor, Department of Paediatric Surgery, IMS & SUM Medical College & Hospital, Bhubaneswar, Odisha, India

Email: bbtripathy.dr@gmail.com

46. Rajendra Prasad G.

Professor, Department of Paediatric Surgery, Andhra Medical College, Vishakhapatnam, Andhra Pradesh, India

Email: dr.grajendraprasad@gmail.com

47. Harish Kumar S

Paediatrics Radiologist, Apollo Hospitals, Chennai, Tamil Nadu, India

Email: drharishkumar.s@gmail.com

For peer review only

48. Jothilakshmi K.

Professor, Department of Paediatrics, PSG Institute of Medical Sciences, Coimbatore, Tamil Nadu, India

Email: drjothi.cbe@gmail.com

49. Nihar Ranjan Sarkar

Associate Professor, Department of Radiology, Institute of Post Graduate Medical Education and Research, Kolkata, West Bengal, India

Email: dr.niharsarkar@gmail.com

50. Pavai Arunachalam

Professor, Department of Paediatric Surgery, PSG Institute of Medical Sciences, Coimbatore, Tamil Nadu, India

Email: pavai4321@yahoo.com

51. Satya Sundar G. Mohapatra

Professor, Department of Radiology, IMS & SUM Medical College & Hospital, Bhubaneswar, Odisha, India

Email: drartifact@gmail.com

52. Saurabh Garge

Consultant Paediatric Surgery, Choithram Hospital and Research Centre, Indore, Madhya Pradesh, India

Email: saurabhgarge8@gmail.com

For peer review only - http://bmjopen.bmj.com/site/about/guidelines.xhtml

Abstract

Objectives: The study aimed to document the association between intussusception in Indian children and meteorological parameters and examine regional variations.

Design: A bidirectional (retrospective and prospective) surveillance included intussusception cases between July 2010 and September 2017.

Setting: At 20 hospitals in India, retrospective case record review during July 2010 and March 2016 and prospective surveillance during April 2016 and September 2017 were performed.

Participants: 2161 children aged 2-24 months with first intussusception episode were included.

Interventions: The monthly mean meteorological parameters (temperature, sunshine, rainfall, humidity and wind speed) for the study sites were collected.

Methods: The association between monthly intussusception cases and meteorological parameters were examined at pooled, regional and site levels using Pearson's (r) and Spearman's rank-order (ρ) correlation, factorial analysis-of-variance and Poisson regression or negative binomial regression analyses.

Results: The intussusception cases were highest in summer and lowest in autumn seasons. Pearson's correlation analysis showed that temperature (r=0.056;$p<0.05$), wind speed (r=0.134;$p<0.01$) and humidity (r=0.075;$p<0.01$) were associated with monthly intussusception. Spearman's' rank-order correlation analysis found that temperature (ρ=0.049;$p<0.05$), wind speed (ρ=0.096;$p<0.01$) and sunshine (ρ=0.051;$p<0.05$) were associated with monthly intussusception. Poisson regression analysis resulted that monthly intussusception cases was associated with rising temperature (North region, $p<0.01$ and East region, $p<0.05$), sunshine (North region,$p<0.01$), humidity (East region,$p<0.01$), and wind speed (East region,$p<0.01$). Factorial analysis-of-variance revealed significant seasonal difference in intussusception cases for pooled level ($p<0.05$), 2-6 months age group ($p<0.05$)

For peer review only - http://bmjopen.bmj.com/site/about/guidelines.xhtml

and North region ($p<0.01$). Significant differences in intussusception cases between summer and autumn seasons was observed for pooled ($p<0.01$), children aged 2-6 months ($p<0.05$) and 7-12 months ($p<0.05$).

Conclusions: A significant correlation between intussusception and temperature, humidity and wind speed was observed at pooled and regional level in India. A peak in summer months was noted, which may be used for prediction, early detection and referral for appropriate management of intussusception.

Keywords: intussusception, children, climate, meteorological parameters, ambient temperature, season, India

For peer review only

For peer review only - http://bmjopen.bmj.com/site/about/guidelines.xhtml

Strengths and limitations of the study

- This study included intussusception cases in children from 20 sites in India representing different geographic and climate regions.
- Relatively large number of intussusception cases in children over seven years were analysed at pooled, regional and site levels using multiple statistical tests for consistency and triangulation.
- Consistency in the correlation between the meteorological parameters and intussusception using multiple statistical tests was reassuring.
- Data for over five years were collected by retrospective surveillance may be a limitation.

For peer review only

1. Introduction

Intussusception is the most common acute abdomen with intestinal obstruction in children, mostly occurring in infants [1,2]. The incidence of intussusception vary widely with mean 74 per 100,000 (range: 9-328) among infants with peak during 5-7 months of age [3]. The available information from India suggest intussusception incidence to vary from 17.7 to 254 per 100,000 child-years [4,5]. These children usually present with abdominal pain or excessive crying, vomiting, bloody stools and may have abdominal mass. Ultrasound is the leading diagnostic tool. Majority of the patients can be managed by reduction method and some may need surgery. In majority of the children, the aetiology of intussusception remain unknown [6].

Intussusception has attracted attention due to its association with the rotavirus vaccine (RVV). India introduced RVV into the universal immunization program (UIP) in 2016 and expanded countrywide in 2019. A recent publication by our group reported regional variation in the intussusception cases among Indian children under-two year age [7]. More number of cases were observed during March to June months (summer months) suggesting seasonal variation. Other studies from India also reported seasonal variation [5,8-11]. There is variation in seasonality of intussusception occurrence globally. Higher number of intussusception cases have been reported during warmer months from some countries [12-16], while no seasonal variation reported from other countries [17-23]. Reports from China found the intussusception case load to be associated with meteorological parameters like temperature, sunshine and humidity [12,24]. In several reports from different countries, the association between meteorological factors and intussusception remains unclear, due to various reasons including shorter study duration, smaller sample size, and no definite statistical methods to explore the association. Although the seasonal variations have been reported, no association between the intussusception and meteorological parameters have been reported from India.

For peer review only - http://bmjopen.bmj.com/site/about/guidelines.xhtml

A multisite surveillance in India documented the epidemiology and trend of intussusception as part of the vaccine safety surveillance linked to the RVV introduction. Using the data collected by the Indian surveillance network, this study investigated the possible association between intussusception cases and various meteorological parameters and seasonality at site, regional and national levels.

2. Methods

2.1. Study area and participating hospitals

This hospital-based sentinel surveillance for intussusception in under-two year children at 20 Indian major tertiary care hospitals included both prospective and retrospective periods. This bidirectional surveillance was comprised of a retrospective (July 1, 2010 to March 31, 2016) and a prospective (April 1, 2016 to September 30, 2017) periods of data collection. The study sites were selected from four regions (north, south, east, and west) of the country ensuring a mix of public and private tertiary care hospitals (North: three public and two private; South: two public and three private; East: five public and one private; West: 2 public, and one private) (Supplementary Figure 1). The study sites represented different climate zones in India. The method of study sites selection have been published in the protocol [25].

2.2. Case definition, case selection and data collection

The children aged 2-24 months admitted to these hospitals with intussusception were eligible. For the retrospective surveillance period, the potential cases were identified using international classification of diseases (ICD 9/10) or diagnoses in medical record section (Supplementary Table 1). These case records were reviewed to identify the intussusception cases and all cases were recruited. For the prospective surveillance period, all age-eligible children were screened,

For peer review only - http://bmjopen.bmj.com/site/about/guidelines.xhtml

tracked till final diagnosis and all intussusception cases were recruited after written informed consent. Only the first intussusception cases were included in analysis. The cases of first episode of intussusception were identified from the history and/or from parents. For the confirmed cases, data including demography, clinical features, management, and final outcome were abstracted using case record forms. External experts visited the study sites to assess the protocol adherence for case selection, record retrieval and data abstraction. Data team members visited the sites and repeated the case retrieval process for 2014, 2015, and 2016 to assess the retrieval completeness. Data queries were clarified with reference to the source documents.

2.3. Meteorological data collection

For the study sites, meteorological data including monthly mean parameters including temperature (^{0}Centigrade, ^{0}C), sunshine (total hours), rainfall (millimetre, mm), humidity (%) and wind speed (kilometre per hour, Kmph) were collected from the website, World Weather Online (https://www.worldweatheronline.com/). These meteorological data collected for the period July 2010 through September 2017 were available in the public domain.

2.4. Data Management and analysis

The descriptive analysis included proportions, means (with standard deviations), and median (with interquartile range, IQR). There was no definite population catchment area for the hospitals and the estimation of intussusception incidence was not possible. The calendar months were grouped into seasons (summer, rainy, autumn, winter and spring) for the individual sites and regions (Supplementary Table 2). The Chi-squared goodness of fit test was used to examine the periodicity of intussusception and monthly differences. Kruskal-Wallis H test (χ^2) was performed to document the differences between >3 groups (years, months seasons and temperature ranges: <20°C, 21°C-25°C, 26°C-30°C and >30°C). Pearson's correlation (*r*)

For peer review only - http://bmjopen.bmj.com/site/about/guidelines.xhtml

analysis explored the association between monthly intussusception cases (dependent variable) and monthly mean meteorological parameters separately (independent variables). Spearman's rank-order correlation (ρ) analysis was also conducted to explore whether the monthly intussusception and monthly mean meteorological parameters covary. Factorial analysis-of-variance (F) was used to analyse the variations in the intussusception cases during the different seasons (dependent variable: seasonal intussusception cases and independent variable: season and year). Poisson regression or negative binomial regression (β (for parameters with wide dispersion) analyses was performed to evaluate the relationships between the monthly intussusception cases (dependent variable) and the monthly mean meteorological parameters (independent variables). The association between intussusception and meteorological parameters were examined at pooled, regional and site levels with 95% confidence interval (CI) estimation. Statistical significance was considered if $p<0.05$. Statistical analysis was performed using STATA version 15.0 (Stata Corp LLC, Texas, USA).

2.5. Ethical issues

The study protocol was reviewed and approved by all the ethics review committees of each participating institute. Confidentiality in data handling was maintained. The participants under prospective component were recruited after written informed consent from parent/legal guardian.

2.6. Patient and Public Involvement

The study participants or the public were not involved in the design, or conduct, or reporting, or dissemination plans of our research. The findings would be relevant for care seeking and case management for intussusception in children. The results published shall be disseminated to the public through social media and through healthcare providers.

For peer review only

3. Results

3.1 Demography data

During July 2010 through September 2017, 2279 children aged 2-24 months with intussusception were recruited. Out of these, 118 children had past history of intussusception, and were excluded. Thus, 2161 children with first intussusception were analysed. The demography and regional distribution of the intussusception cases across the years is shown in Table 1. There were 1431 (66.2%) boys and 730 (33.8%) girls (male-female ratio, 1.96). Majority (n=1653, 76.5%) were infants. The number of cases increased across the years with highest (31%) jump between 2014 and 2015. During 2010-2014, the children aged 7-12 months had a higher share, which changed during 2015-2017. The South region contributed the highest (50%), followed by East (27.4%), North (17.1%) and West (5.4%) regions, which were consistent across the years.

3.2. Meteorological data

The monthly meteorological parameters for the regions are shown in Supplementary Table 3. The monthly meteorological parameters for each site is given in Supplementary Table 4. Across the regions, April to June and December-February had the highest and lowest temperatures respectively. The pattern of meteorological parameters across the regions is shown in Supplementary Figure 2.

3.3. Monthly and seasonal cycles of intussusception

The Chi-squared goodness of fit test revealed obvious monthly (χ^2-statistic= 71.36; $p<0.01$) and seasonal differences (χ^2-statistic= 382.28; $p<0.01$) in the intussusception case load. Also Kruskal-Wallis H test revealed significant differences in the intussusception cases for months ($p<0.01$) and seasons ($p<0.01$). As shown in Figure 1, more intussusception cases were

For peer review only

For peer review only - http://bmjopen.bmj.com/site/about/guidelines.xhtml

observed during March in South region, June in North and East regions, all during summer season. Not much variation in cases was observed in West region. The intussusception cases were lowest in September and October months during these years. The peak and lowest seasons were summer (March-June; n=661, 30.6% cases) and autumn (September-October; 14.5% cases), respectively (Supplementary Figure 3).

3.4. Correlation between the intussusception and meteorological factors

Table 2 shows the Pearson's correlation analysis for pooled, regions and age groups. For pooled data, the temperature (r=0.056; p<0.05), wind speed (r=0.134; p<0.01) and humidity (r=0.075; p<0.01) had significant association with intussusception. At regional level, intussusception cases had association with temperature for East region (r=0.052; p<0.05); with rainfall for North (r=-0.103; p<0.01) and East regions (r=0.073; p<0.01); with wind speed for all regions (r=-0.084 to 0.21; p<0.01); with humidity for three regions except West region (r=-0.373 to 0.161; p<0.01); and with sunshine for North region (r=0.163; p<0.01). For the age groups, intussusception cases had association with temperature for 7-12 months (r=0.068; p<0.01), with wind speed for all age groups (r=-0.092 to 0.125; p<0.01), with humidity for 7-12 months (r=0.069; p<0.01) and 13-24 months (r=0.076; p<0.01) age groups. The Pearson correlation analysis for intussusception cases with the meteorological parameters for the study sites are given in Supplementary Table 5. Significant correlations for intussusception cases with temperature were observed for three sites, with rainfall for two sites, with wind speed and humidity for five sites each and with sunshine for three sites.

Table 3 shows the Spearman's' rank-order correlation analysis for pooled, regions and age groups. For pooled data, the temperature (ρ=0.049; p<0.05), wind speed (ρ=0.096; p<0.01) and sunshine (ρ=0.051; p<0.05) were associated with intussusception. At regional level,

For peer review only - http://bmjopen.bmj.com/site/about/guidelines.xhtml

intussusception cases had association with temperature for North region (ρ=0.110; p<0.05); with wind speed for North (ρ=0.183; p<0.01) and East (ρ=0.109; p<0.01) regions; with humidity for North (ρ=-0.114; p<0.05) and East (ρ=-0.102; p<0.05) regions; and with sunshine for North region ((ρ=0.119; p<0.05). For the age groups, intussusception cases had association with temperature for 7-12 months ((ρ=0.064; p<0.01), with wind speed for all age groups ((ρ=0.063 to 0.106; p<0.01), with humidity (ρ=0.049; p<0.05) and sunshine (ρ=0.048; p<0.05) for 7-12 months age group. The Spearman's' Rank correlation analysis for intussusception cases with the meteorological parameters for the study sites are given in Supplementary Table 6. Significant correlations for intussusception cases with temperature were observed for one site, with rainfall for two sites, with wind speed for three sites, with humidity for five sites and with sunshine for two sites.

Regression analyses (Poisson regression and negative binomial regression) revealed significant association between intussusception cases and temperature for pooled level (β=0.026; 95% CI 0.011, 0.041; p<0.01) North (β=0.027; 95% CI 0.007, 0.047; p<0.01) and East (β=0.029; 95% CI 0.001, 0.057; p<0.05) regions; wind speed for pooled level (β=0.082; 95% CI 0.061, 0.103; p<0.01), North (β=0.208; 95% CI 0.101, 0.315; p<0.01) and East (β=0.091; 95% CI 0.042, 0.141; p<0.01) regions; humidity for pooled level (β=0.011; 95% CI 0.007, 0.016; p<0.01), East (β=-0.021; 95% CI -0.035, -0.007; p<0.01) region and sunshine for pooled level (β=0.002; 95% CI 0, 0.003; p<0.01), North (β=0.004; 95% CI 0.002, 0.007; p<0.01) region (Table 4). Associations between the intussusception cases and temperature and humidity were evident across different age groups (p<0.01). The association was also significant at study sites level for temperature (three sites), humidity (six sites) and sunshine (five sites) (Supplementary Table 7).

For peer review only

For peer review only - http://bmjopen.bmj.com/site/about/guidelines.xhtml

The monthly average temperatures at the sites and regions varied widely; 14°C to 35°C in North, 23°C to 31°C in South, 20°C to 32°C in East and 22°C to 31°C in West regions. Chi-squared goodness of fit test showed significant difference at 20°C–30°C for pooled, North and South regions ($p<0.01$), indicating that the intussusception cases occurred more during this temperature (Supplementary Table 8/13). Kruskal-Wallis H test found significant variation in the intussusception cases between the different temperature categories for pooled ($\chi^2=15.933$; $p<0.01$), North ($\chi^2=21.054$; $p<0.01$) and South ($\chi^2=50.625$; $p<0.01$) regions (Supplementary Table 9/14). The number of intussusception cases increased when the mean daily temperature and wind speed increased and mean daily humidity decreased at pooled level and for North and East regions (Supplementary Figure 4-8).

3.5. Correlation between the intussusception, seasons and age groups

Factorial analysis-of-variance revealed significant difference in seasonal intussusception cases at pooled level ($F=1.442$; $p<0.05$), for 2-6 months age group ($F=1.579$; $p<0.05$) and for North region ($F=1.809$; $p<0.01$) (Supplementary Table 10/8 and 11/9). Multiple comparison revealed significant difference in intussusception cases between summer and autumn seasons (mean difference=0.569; 95% CI 0.11-1.03; $p<0.01$) at pooled level (Supplementary Table 12/10). Significant differences intussusception cases between summer and monsoon (mean difference=0.165; 95% CI 0.04-0.29, $p<0.01$), summer and autumn (mean difference=0.179; 95% CI 0.03-0.32, $p<0.01$), spring and monsoon (mean difference=0.276; 95% CI 0.09-0.46, $p<0.01$) and spring and autumn (mean difference=0.290; 95% CI 0.09-0.49, $p<0.01$) were observed for North region (Supplementary Table 13/11). Significant differences intussusception cases were observed between summer and autumn for children aged 2-6 months (mean difference=0.195; 95% CI 0.01-0.38, $p<0.05$) and for children aged 7-12 months (mean difference=0.231; 95% CI 0.01-0.45, $p<0.05$) (Supplementary Table 14/12).

For peer review only

For peer review only - http://bmjopen.bmj.com/site/about/guidelines.xhtml

4. Discussion

The seasonal influence on intussusception cases has been proposed, but with inconsistent findings from different countries and different parts of India. This study is the first to explore association between intussusception and monthly mean meteorological parameters (temperature, rainfall, wind speed, humidity and sunshine) in India. This study comprising of 2161 children with first intussusception from 20 sites in India documented monthly and seasonal variations. Intussusception case peaks were observed during summer months (Mar-June) and troughs during autumn months (September-October). There were positive correlation between intussusception cases and meteorological parameters like temperature, humidity, wind speed and sunshine at pooled and regional levels and among children aged 7-12 months. The associations were consistent across analysis tests used (Pearson correlation, Spearman's Rank correlation tests, Poisson regression or negative binomial regression). The association was more pronounced for North and East regions, where the meteorological parameters fluctuation across the months were widest. Further analysis using factorial analysis-of-variance also demonstrated seasonal inclination with peaking in summer season (Mar-June) in India and significant difference from autumn season (with lowest cases) ($p<0.01$). A significant difference in number of intussusception cases between the temperature ranges (<20°C, 21°C - 25°C, 26°C -30°C and >30°C) was observed at pooled and regional (North and South regions) levels, which indicated rise in intussusception cases with rise in monthly mean temperature. The intussusception cases increased with rise in daily temperature and wind speed and fall in humidity.

For peer review only - http://bmjopen.bmj.com/site/about/guidelines.xhtml

The findings are consistent with the report from China in 5994 children (aged 0-12 years) with intussusception, where more cases were documented in summer months with significant positive association with mean monthly meteorological parameters (temperature, sunshine, and precipitation) [12]. A modelling using the data from 13887 Chinese children with intussusception and applying the meteorological variation assumptions achieved considerable agreement with the data collected [24]. These reports from China didn't include humidity as a variable.

Studies on intussusception in children from India have reported higher cases during summer (March-July) and lowest during autumn (September-November) seasons. In three studies from South India, highest and lowest intussusception cases were observed during March-June and September-October respectively [5,9,10]. A multisite study from India reported highest and lowest cases during March and September months respectively [8]. A study from North India observed highest and lowest cases during June and September months [11]. A report from our group on the retrospective surveillance observed higher number of intussusception cases during summer months (March-June) over the year [7].

Seasonal pattern of intussusception occurrence have been observed from different countries. Higher number of intussusception cases were observed during warmer months (May-October) than cooler months (November-April) in Taiwan [14]. Among Hong Kong children with intussusception, higher cases were observed during summer months (May-July) [26]. Seasonality in intussusception case occurrence was observed in Korean children with highest during warmer months (July-August) and lowest during cooler months (February) [27]. Among Ethiopian children, higher intussusception cases were observed during June month and lower during November months [13]. Higher intussusception cases were observed during summer

For peer review only

months and lower during winter months among South African children [15]. Among Israeli children, more intussusception cases were observed during summer months and lower during winter [16]. No seasonal variation was also observed in some studies from different countries. Studies from Latin American countries and North America didn't observe visible seasonal variation in cases [17,23]. Studies from Switzerland, France, New Zealand, Singapore and China also had no sizable seasonal variations [18-22].

The inconsistencies in the seasonal variations may be due to the sample size, age groups included and stratification in analysis. Three studies which observed definite seasonal variation [12,14,24] and two observed statistically significant association between the meteorological parameters and intussusception has larger sample size (>5000 participants). The association observed in our study may be also due to higher sample size. The reasons for the higher intussusception cases during summer is not known and the seasonality don't match with the rotavirus infection seasonality in India [28]. According to the reports from some developed countries (United States and Australia), the intussusception incidence have declined over decades and also the prevalence of prodrome infection symptoms (gastrointestinal and respiratory infections) in these children over the same period [29-31]. Further studies are needed to confirm the seasonality, association with the meteorological parameters and variations in seasonality across the regions in India along with the influences of the preventive healthcare services coverage, care seeking practices and access to the sanitation, hygiene and changes in dietary practices.

A seasonal variation in another acute intestinal condition, acute appendicitis in children have been reported. More acute appendicitis cases in children during summer months and lower during spring/autumn seasons have been reported from India, Pakistan, Iraq, China, Taiwan,

For peer review only

Italy, Germany, Canada and United States [32-39]. Among Chinese children, association between acute appendicitis and temperature ($p<0.01$), humidity ($p<0.01$) and sunshine ($p<0.01$) were documented to support the seasonal variation [35]. Similar to the intussusception, the appendicitis incidence has also declined in developed countries over decades, possible due to changes in the hygiene, sanitation, dietary practices, access to healthcare and reduction in the infectious diseases [40]. The intussusception and acute appendicitis, both are intestinal problems and some of the intussusception have associated appendicitis [41-43]. The climate factors are unlikely to directly cause intussusception or intussusception in children. But the linkage between seasonality and meteorological parameters and acute intestinal conditions appears to be plausible, which may mediated by intestinal and non-intestinal infections (including activation of latent viral infections), mucosal inflammatory changes, lymphoid hyperplasia, or motility alterations and dietary intake changes. Although no direct relationship with the rotavirus infection could be established, higher gastrointestinal infection during summer and early rainy seasons may be triggers for higher intussusception in Indian context. The changes in intussusception and appendicitis incidence over time in parallel with the industrialization, economic improvement, improved access to vaccination, healthcare and sanitation and hygiene practices suggest possible association. Such information in Indian and developing country context is not available. Thus the seasonal variation in these conditions and association between meteorological parameters appear epidemiologically and biologically plausible, which needs further evaluation. We used Pearson's correlation coefficient as the primary analysis to document the strength and direction of relationship between the meteorological parameters and intussusception. As secondary analysis and checking for the consistency in the findings, we used Spearman's rank-order correlation analysis to identify strength of association between ranked parameters and intussusception variables.

For peer review only

The Pearson's correlation coefficient (r) and Spearman's correlation coefficient (ρ) values for different meteorological parameters were similar, although the r values for temperature, rainfall, wind speed and humidity were higher than the ρ values, but had r value was lower for sunshine than the ρ value at pooled level. The differences between the r and ρ values were relatively small, which comforts and supports the consistent association between the variables. The consistency in findings from factorial analysis-of-variance and regression analysis with the correlation coefficients further confirms the association between the meteorological parameters and intussusception.

The study had several strengths; relatively large number of intussusception cases over seven years period, sites from different regions, and statistical methods used and consistency in the findings. The consistency across the results using different statistical tests was assuring. There are several limitations in the study. A major part of this study was retrospective. The sites belong to different climate zones and had variation in the meteorological parameters. Lack of definite catchment population and referral pattern, made it difficult to estimate the intussusception incidence rate.

In conclusion, this study documented higher intussusception cases during the summer months with a positive association between number of cases with temperature and sunshine. Also significant positive correlation with the humidity and wind speed was observed. The association was significant for the North and East regions with wider variations in the meteorological parameters across seasons. Higher degree of suspicion for infants with compatible clinical presentation for intussusception during the summer months may improve diagnosis and clinical care to prevent the complications. The study provides evidence for predicting the intussusception pattern in children and sensitizing the healthcare providers at

For peer review only - http://bmjopen.bmj.com/site/about/guidelines.xhtml

community and peripheral facilities for early detection and quick referral of patients to minimise the surgical interventions and avoid fatalities. Further studies are needed to confirm the observation and improve the evidence base on the potential risk factors for the season variations and the aetiologies of intussusception.

For peer review only

For peer review only - http://bmjopen.bmj.com/site/about/guidelines.xhtml

Acknowledgments

We acknowledge the support from Ministry of Health and Family Welfare, Government of India for undertaking the study. We are thankful to the hospital administrations and the clinicians at the study site institutes, who supported and facilitated undertaking of the study.

We highly value the technical guidance and inputs provided by the members of Technical Advisory Group: Mahesh K Agarwal and Pradeep Haldar, Ministry of Health and Family Welfare, Government of India; Satinder Aneja, Anju Seth and Archana Puri, Lady Hardinge Medical College, New Delhi; Ashok Patwari, Hamdard Institute of Medical Sciences & Research, New Delhi; Yogesh Kumar Sarin, Maulana Azad Medical College, New Delhi; Rakesh Aggarwal, Anshu Srivastava and Ujjal Poddar, Sanjay Gandhi Postgraduate Institute of Medical Sciences, Lucknow; Malathi Satyasekharan, Kanchi Kamakoti Chailds Trust Hospital, Chennai; Raju Sharma and Nirupam Madan, All India Institute of Medical Sciences, New Delhi; Jyoti Joshi and Deepak Polpakara, Immunization Technical Support Unit; Ministry of Health & Family Welfare, New Delhi; Umesh D. Parashar; Centers of Disease Control and Prevention, Atlanta, USA; Jan Bonhoeffer, University Children's Hospital, Basel, Switzerland and Brighton Collaboration; Naveen Thacker, Child Health Foundation, Gandhigram; Rashmi Arora, Indian Council of Medical Research, Ansari Nagar, New Delhi; and Patrick L F Zuber and Christine G. Maure, World Health Organisation, Geneva, Switzerland.

We acknowledge the contribution of the research staffs at The INCLEN Trust International: Harshpreet Kaur, Janvi Chaubey, Mrimmaya Das, Shweta Sharma and Vaibhav Jain.

We highly appreciate the efforts made by the research staffs at the study sites: Aarezo Bashir and Rafia; Sher-e-Kashmir Institute of Medical Sciences, Srinagar, Jammu & Kashmir; Prabha Shankar, Medanta-The Medicity Hospital, Gurgaon, Haryana; Anju Sharma; Maulana Azad Medical College, New Delhi; Anita Singh and Shubhanshu Srivastava, King George Medical University, Lucknow, Uttar Pradesh; Hemant Meena, Choithram Hospital, Indore, Madhya

For peer review only

Pradesh; Pankaj Kumar and Shashi Kant; Indira Gandhi Institute of Medical Sciences, Patna, Bihar; Goutam Benia, IMS & SUM Medical College & Hospital, Bhubaneshwar, Odisha; Prasntajyoti Mohanty, SVP Post Graduate Institute of Paediatrics, Cuttack, Odisha; Asit Pradhan, MKCG Medical College, Berhampur, Odisha; Angshuman Chatterjee, Institute of Postgraduate Medical Education and Research & SSKM Hospital, Kolkata, West Bengal; S. Yamuna, Andhra Medical College, Vishakhapatnam, Andhra Pradesh; Srinidhi Sudan, Apollo Hospitals, Hyderabad, Telengana; Rajesh Francis, Apollo Hospitals, Chennai, Tamil Nadu; T. Easter Chandru, PSG Institute of Medical Sciences, Coimbatore, Tamil Nadu; Deepthy R, Julie and Anju Shivakumar, Government Medical College & SAT Hospital, Thiruvananthapuram, Kerala; Archit Vaidya, Grant Medical College & JJ Hospital, Mumbai, Maharashtra; Nimesh Chouksey, MP Shah Government Medical College, Jamnagar, Gujarat; Nidhi Singh, Fortis Escorts Hospital, Jaipur, Rajasthan; Mrinmoy Gohain, Gauhati Medical College, Guwahati, Assam; Arpita Bhattachrjee, Saugat Ghosh and Tanusmita Debnath, Agartala Government Medical College, Agartala, Tripura.

For peer review only

Declarations

Ethics approval and consent to participate: The study protocol was reviewed and approved by the ethics committees of all the participating institutes (Supplementary Document 2). The participants were recruited after obtaining written informed consent.

Consent for publication: Not applicable

Availability of data and materials: All data are available with the investigators and can be provided by the corresponding author upon reasonable request.

Competing interests: The authors declare that there is no competing interests and conflict of interest.

Disclosure statement: None. There is no financial interest or benefit for the authors arisen from this project or its direct application.

Funding: This project was supported by the Bill and Melinda Gates Foundation, USA to The INCLEN Trust International (grant number OPP1116433). The funder or its representative had no role in the design of the study and collection, analysis, and interpretation of data and writing the manuscript.

Authors' contributions:

Study conceptualisation, study design, protocol development, training, data analysis, interpretation: MKD and NKA.

Study coordination, monitoring, and data cleaning: MKD, BG, and AS.

Data analysis, interpretation, and manuscript preparation: MKD, BG and SS-1.

Protocol development, quality assurance and monitoring: AR

Participant recruitment and data collection: AW, BRV, JIB, JKG, JM, KK, LB, LS, AMK, NM, PKJ, RS-1, RS-2, SKD, SJ, SKR, SS-2, VK, NB, RG, APD, AG, CJS, GNM, HT, JS, KL, KR, ML, PP, RK, RS-3, SKA, SKS, SKG, SM, AD, BAC, BBT, RPG, HKS, JK, NRS, PA, SSGM, and SG.

For peer review only

650 All authors reviewed, provided critical input and approved the final version.

For peer review only

List of abbreviations

CI Confidence interval

ICD International Classification of Diseases

IQR Interquartile range

RVV Rotavirus vaccine

UIP Universal immunization programme

For peer review only

For peer review only - http://bmjopen.bmj.com/site/about/guidelines.xhtml

References

1. Bines JE, Kohl KS, Forster J, Zanardi LR, Davis RL, Hansen J, et al. Acute intussusception in infants and children as an adverse event following immunization: case definition and guidelines of data collection, analysis, and presentation. Vaccine. 2004 Jan;22(5–6):569–74.
2. Bines JE, Ivanoff B. Acute intussusception in infants and children: Incidence, clinical presentation and management: a global perspective [Internet]. World Health Organisation; 2002 [cited 2017 May 10]. Available from: http://apps.who.int/iris/bitstream/handle/10665/67720/WHO_V-B_02.19_eng.pdf;jsessionid=74671C3CD99ED55137202F8D3D07CB73?sequence=1
3. Jiang J, Jiang B, Parashar U, Nguyen T, Bines J, Patel MM. Childhood Intussusception: A Literature Review. Cameron DW, editor. PLoS ONE. 2013 Jul 22;8(7):e68482.
4. Bahl R, Saxena M, Bhandari N, Taneja S, Mathur M, Parashar UD, et al. Population-Based Incidence of Intussusception and a Case-Control Study to Examine the Association of Intussusception with Natural Rotavirus Infection among Indian Children. The Journal of Infectious Diseases. 2009 Nov;200(s1):S277–81.
5. Jehangir S, John J, Rajkumar S, Mani B, Srinivasan R, Kang G. Intussusception in southern India: Comparison of retrospective analysis and active surveillance. Vaccine. 2014 Aug;32:A99–103.
6. Jain S, Haydel MJ. Child Intussusception. In: StatPearls [Internet]. Treasure Island (FL): StatPearls Publishing; 2020 [cited 2020 Jul 19]. Available from: http://www.ncbi.nlm.nih.gov/books/NBK431078/
7. Das MK, Arora NK, Gupta B, Sharan A, Kameswari K, Padmalatha P, et al. Intussusception in children aged under two years in India: Retrospective surveillance at nineteen tertiary care hospitals. Vaccine. 2020 Jun;S0264410X20305636.

For peer review only

For peer review only - http://bmjopen.bmj.com/site/about/guidelines.xhtml

8. Singh JV, Kamath V, Shetty R, Kumar V, Prasad R, Saluja T, et al. Retrospective surveillance for intussusception in children aged less than five years at two tertiary care centers in India. Vaccine. 2014 Aug;32:A95–8.

9. Bhowmick K, Kang G, Bose A, Chacko J, Boudville I, Datta SK, et al. Retrospective surveillance for intussusception in children aged less than five years in a South Indian tertiary-care hospital. J Health Popul Nutr. 2009 Oct;27(5):660–5.

10. Srinivasan R, Girish Kumar CP, Naaraayan SA, Jehangir S, Thangaraj JWV, Venkatasubramanian S, et al. Intussusception hospitalizations before rotavirus vaccine introduction: Retrospective data from two referral hospitals in Tamil Nadu, India. Vaccine. 2018 Dec;36(51):7820–5.

11. Gupta M, Kanojia R, Singha R, Tripathy JP, Mahajan K, Saxena A, et al. Intussusception Rate Among Under-Five-Children Before Introduction of Rotavirus Vaccine in North India. Journal of Tropical Pediatrics. 2018 Aug 1;64(4):326–35.

12. Guo W, Zhang S, Li J, Wang J. Association of Meteorological Factors with Pediatric Intussusception in Subtropical China: A 5-Year Analysis. Wang L, editor. PLoS ONE. 2014 Feb 28;9(2):e90521.

13. Gadisa A, Tadesse A, Hailemariam B. Patterns and seasonal variation of intussusception in children: a retrospective analysis of cases operated in a tertiary hospital in Ethiopia. Ethiop Med J. 2016 Jan;54(1):9–15.

14. Chen SC-C, Wang J-D, Hsu H-Y, Leong M-M, Tok T-S, Chin Y-Y. Epidemiology of childhood intussusception and determinants of recurrence and operation: analysis of national health insurance data between 1998 and 2007 in Taiwan. Pediatr Neonatol. 2010 Oct;51(5):285–91.

For peer review only

For peer review only - http://bmjopen.bmj.com/site/about/guidelines.xhtml

15. Moore SW, Kirsten M, Müller EW, Numanoglu A, Chitnis M, Le Grange E, et al. Retrospective surveillance of intussusception in South Africa, 1998-2003. J Infect Dis. 2010 Sep 1;202 Suppl:S156-161.

16. Greenberg D, Givon-Lavi N, Newman N, Wheeler J, Cohen Z, Dagan R. Intussusception in Children in Southern Israel: Disparity between 2 Populations. The Pediatric Infectious Disease Journal. 2008 Mar;27(3):236–40.

17. Sáez-Llorens X, Velázquez FR, Lopez P, Espinoza F, Linhares AC, Abate H, et al. A multi-country study of intussusception in children under 2 years of age in Latin America: analysis of prospective surveillance data. BMC Gastroenterol. 2013 May 27;13:95.

18. Buettcher M, Baer G, Bonhoeffer J, Schaad UB, Heininger U. Three-year surveillance of intussusception in children in Switzerland. Pediatrics. 2007 Sep;120(3):473–80.

19. Serayssol C, Abbo O, Mouttalib S, Claudet I, Labarre D, Galinier P, et al. [Seasonal pattern of intussusceptions in infants and children: is fall/winter predominance still worth consideration? A 10-year retrospective epidemiological study]. Arch Pediatr. 2014 May;21(5):476–82.

20. Chen YE, Beasley S, Grimwood K, and the New Zealand Rotavirus Study Group. Intussusception and rotavirus associated hospitalisation in New Zealand. Archives of Disease in Childhood. 2005 Jun 7;90(10):1077–81.

21. Boudville IC, Phua KB, Quak SH, Lee BW, Han HH, Verstraeten T, et al. The epidemiology of paediatric intussusception in Singapore: 1997 to 2004. Ann Acad Med Singap. 2006 Oct;35(10):674–9.

22. Liu N, Yen C, Huang T, Cui P, Tate JE, Jiang B, et al. Incidence and epidemiology of intussusception among children under 2 years of age in Chenzhou and Kaifeng, China, 2009-2013. Vaccine. 2018 14;36(51):7862–7.

For peer review only

For peer review only - http://bmjopen.bmj.com/site/about/guidelines.xhtml

23. Chang HG, Smith PF, Ackelsberg J, Morse DL, Glass RI. Intussusception, rotavirus diarrhea, and rotavirus vaccine use among children in New York state. Pediatrics. 2001 Jul;108(1):54–60.

24. Guo W, Geng J, Zhan Y, Tan Y, Hu Z, Pan P, et al. Forecasting and predicting intussusception in children younger than 48 months in Suzhou using a seasonal autoregressive integrated moving average model. BMJ Open. 2019 Jan;9(1):e024712.

25. Das M, Arora N, Bonhoeffer J, Zuber P, Maure C. Intussusception in Young Children: Protocol for Multisite Hospital Sentinel Surveillance in India. Methods and Protocols. 2018 Mar 22;1(2):11.

26. Nelson EAS, Tam JS, Glass RI, Parashar UD, Fok TF. Incidence of rotavirus diarrhoea and intussusception in Hong Kong using standardized hospital discharge data. The Pediatric Infectious Disease Journal. 2002 Jul;21(7):701–3.

27. Jo S, Lim IS, Chae SA, Yun SW, Lee NM, Kim SY, et al. Characteristics of intussusception among children in Korea: a nationwide epidemiological study. BMC Pediatr. 2019 Dec;19(1):211.

28. Giri S, Nair NP, Mathew A, Manohar B, Simon A, Singh T, et al. Rotavirus gastroenteritis in Indian children < 5 years hospitalized for diarrhoea, 2012 to 2016. BMC Public Health. 2019 Dec;19(1):69.

29. Parashar UD, Holman RC, Cummings KC, Staggs NW, Curns AT, Zimmerman CM, et al. Trends in Intussusception-Associated Hospitalizations and Deaths Among US Infants. Pediatrics. 2000 Dec 1;106(6):1413–21.

30. Desai R, Curns AT, Patel MM, Parashar UD. Trends in intussusception-associated deaths among US infants from 1979-2007. J Pediatr. 2012 Mar;160(3):456–60.

31. Justice FA, Auldist AW, Bines JE. Intussusception: trends in clinical presentation and management. J Gastroenterol Hepatol. 2006 May;21(5):842–6.

For peer review only

For peer review only - http://bmjopen.bmj.com/site/about/guidelines.xhtml

32. Jangra B, Jangra M, Rattan K, Kadian Y. Seasonal and day of week variations in acute appendicitis in north Indian children. J Indian Assoc Pediatr Surg. 2013;18(1):42.

33. Ahmed W, Akhtar MS, Khan S. Seasonal variation of acute appendicitis. Pak J Med Sci [Internet]. 2018 May 24 [cited 2020 Aug 4];34(3). Available from: http://pjms.com.pk/index.php/pjms/article/view/14793

34. Al-Mendalawi M, Lattuf M. Is there a seasonal distribution of acute appendicitis in Iraqi children? Menoufia Med J. 2017;30(2):572.

35. Zhang Y, Lyu F, Kang Q, Dong Z, Zhao T, Xie S, et al. Association of meteorological factors with pediatric acute appendicitis in China: A 7-year retrospective analysis. Medicine. 2018 Oct;97(42):e12913.

36. Hsu Y-J, Fu Y-W, Chin T. Seasonal variations in the occurrence of acute appendicitis and their relationship with the presence of fecaliths in children. BMC Pediatr. 2019 Dec;19(1):443.

37. Gallerani M, Boari B, Anania G, Cavallesco G, Manfredini R. Seasonal variation in onset of acute appendicitis. Clin Ter. 2006 Apr;157(2):123–7.

38. Reinisch A, Heil J, Woeste G, Bechstein W, Liese J. The meteorological influence on seasonal alterations in the course of acute appendicitis. Journal of Surgical Research. 2017 Sep;217:137–43.

39. Fares A. Summer appendicitis. Ann Med Health Sci Res. 2014;4(1):18.

40. Ferris M, Quan S, Kaplan BS, Molodecky N, Ball CG, Chernoff GW, et al. The Global Incidence of Appendicitis: A Systematic Review of Population-based Studies. Ann Surg. 2017;266(2):237–41.

41. Atkinson G, Gay B, Naffis D. Intussusception of the appendix in children. Am J Roentgenol. 1976 Jun;126(6):1164–8.

For peer review only

For peer review only - http://bmjopen.bmj.com/site/about/guidelines.xhtml

42. Ozkisacik SK, Erdem AO, Coskun O, Yazici M. Small bowel intussusception together with appendicitis in childhood: A case report. J Pediatr Surg Case Rep. 2015 Jan;3(1):25–6.

43. Marjon L, Hull N, Thomas K. Concurrent acute appendicitis and ileocolic intussusception in a 1-year-old child. Radiol Case Rep. 2018 Jun;13(3):655–7.

For peer review only

For peer review only - http://bmjopen.bmj.com/site/about/guidelines.xhtml

Tables

Table 1: Distribution and demography of the children with intussusception in India

Pooled	Years								
Years	2010[a]	2011	2012	2013	2014	2015	2016	2017[b]	Total
Number of cases, n	68	209	244	260	278	365	429	308	2161
Gender									
Male, n (%)	45 (66.2)	142 (67.9)	155 (63.5)	171 (65.8)	194 (69.8)	243 (66.6)	283 (66)	198 (64.3)	1431 (66.2)
Female, n (%)	23 (33.8)	67 (32.1)	89 (36.5)	89 (34.2)	84 (30.2)	122 (33.4)	146 (34)	110 (35.7)	730 (33.8)
Age groups									
2-6 months, n (%)	24 (35.3)	73 (34.9)	89 (36.5)	86 (33.1)	91 (32.7)	134 (36.7)	159 (37.1)	122 (39.6)	778 (36)
7-12 months, n (%)	33 (48.5)	92 (44)	108 (44.3)	122 (46.9)	113 (40.6)	135 (37)	155 (36.1)	117 (38)	875 (40.5)
13-24 months, n (%)	11 (16.2)	44 (21.1)	47 (19.3)	52 (20)	74 (26.6)	96 (26.3)	115 (26.8)	69 (22.4)	508 (23.5)
Regions									
North, n (%)	9 (13.2)	19 (9.1)	37 (15.2)	44 (16.9)	48 (17.3)	61 (16.7)	91 (21.2)	61 (19.8)	370 (17.1)
South, n (%)	50 (73.5)	134 (64.1)	130 (53.2)	149 (57.3)	124 (44.6)	175 (47.9)	183 (42.7)	136 (44.2)	1081 (50)
East, n (%)	9 (13.2)	45 (21.5)	61 (25)	54 (20.8)	84 (30.2)	112 (30.7)	131 (30.5)	97 (31.5)	593 (27.4)
West, n (%)	0 (0)	11 (5.3)	16 (6.6)	13 (5)	22 (7.9)	17 (4.7)	24 (5.6)	14 (4.5)	117 (5.4)

Note: a: The period of data collection was 6 months (July to December)
b: The period of data collection was 9 months (January to September)

For peer review only

For peer review only - http://bmjopen.bmj.com/site/about/guidelines.xhtml

Table 2. Pearson correlation analysis between monthly intussusception cases and monthly mean meteorological parameters

Parameter	Pearson correlation coefficient (r) for meteorological parameters				
	Temperature (°C)	Rainfall (mm)	Wind speed (m/s)	Humidity (%)	Sunshine (hr)
Pooled (n= 2161)	.056*	-.025	.134**	.075**	.045
Regions					
North (n= 370)	.018	-.103**	-.119**	-.373**	.163**
South (n= 1081)	.022	-.023	.210**	.161**	-.018
East (n= 593)	.052*	.072**	-.084**	.109**	.024
West (n= (117)	.046	-.019	.107**	-.029	-.012
Age groups					
2-6 months (n= 778)	.025	-.022	.092**	.040	.037
7-12 months (n= 875)	.068**	-.012	.115**	.069**	.042
13-24 months (n= 508)	.040	-.030	.125**	.076**	.029

Note: Pearson correlation coefficient (r); Significance two tailed

** Significance level- p value <0.05; ** Significance level- p value <0.01*

For peer review only

For peer review only - http://bmjopen.bmj.com/site/about/guidelines.xhtml

Table 3. Spearman's rank-order correlation analysis between monthly intussusception cases and monthly mean meteorological parameters

Parameter	Spearman's rank-order correlation coefficient (ρ) for meteorological parameters				
	Temperature (°C)	Rainfall (mm)	Wind speed (m/s)	Humidity (%)	Sunshine (hr)
Pooled (n= 2161)	.049*	.012	.096**	.005	.051*
Regions					
North (n= 370)	.110*	-.014	.183**	-.114*	.119*
South (n= 1081)	.028	-.031	-.024	-.027	.036
East (n= 593)	.061	-.042	.109**	-.102*	.038
West (n= (117)	.052	-.044	.000	-.067	.076
Age groups					
2-6 months (n= 778)	.035	.031	.063**	.007	.044
7-12 months (n= 875)	.064**	.036	.103**	.049*	.048*
13-24 months (n= 508)	.028	.019	.106**	.043	.016

Note: Spearman's rank-order correlation coefficient (ρ); Significance two tailed
** Significance level- p value <0.05; ** Significance level- p value <0.01*

For peer review only

For peer review only - http://bmjopen.bmj.com/site/about/guidelines.xhtml

Table 4: Regression analysis of the relationship between the monthly intussusception cases and monthly mean meteorological parameters

Variables	Regression coefficient, β (95% CI)	Standard error, S_β	χ2 - value	p value
Pooled[#]				
Temperature (^{0}C)	0.026 (0.011, 0.041)	0.008	12.098	<0.01
Rainfall (mm)	-0.001 (-0.003, 0)	0.001	2.462	0.117
Wind Speed (Kmph)	0.082 (0.061, 0.103)	0.011	58.449	<0.01
Humidity (%)	0.011 (0.007, 0.016)	0.002	21.903	<0.01
Sunshine (hr)	0.002 (0, 0.003)	0.001	7.000	<0.01
According to regions				
North region[#]				
Temperature (^{0}C)	0.027 (0.007, 0.047)	0.0102	7.195	<0.01
Rainfall (mm)	0.001 (-0.005, 0.007)	0.0029	0.169	0.681
Wind Speed (Kmph)	0.208 (0.101, 0.315)	0.0545	14.556	<0.01
Humidity (%)	-0.010 (-0.020, 0.001)	0.0052	3.448	0.063
Sunshine (hr)	0.004 (0.002, 0.007)	0.0013	11.006	<0.01
South region[#]				
Temperature (^{0}C)	0.010 (-0.037, 0.058)	0.0243	0.174	0.676
Rainfall (mm)	-0.004 (-0.009, 0.000)	0.0024	3.251	0.071
Wind Speed (Kmph)	-0.011 (-0.045, 0.023)	0.0173	0.401	0.527
Humidity (%)	-0.033 (-0.070, 0.005)	0.0190	2.949	0.086
Sunshine (hr)	0.002 (-0.001, 0.004)	0.0014	1.213	0.271
East region[#]				
Temperature (^{0}C)	0.029 (0.001, 0.057)	0.0141	4.245	0.039
Rainfall (mm)	-0.001 (-0.004, 0.001)	0.0014	0.766	0.381
Wind Speed (Kmph)	0.091 (0.042, 0.141)	0.0251	13.244	<0.01
Humidity (%)	-0.021 (-0.035, -0.007)	0.0070	9.188	<0.01
Sunshine (hr)	0.002 (-6.100, 0.005)	0.0012	3.822	0.051
*West region**				
Temperature (^{0}C)	0.052 (-0.02, 0.124)	1.987	0.036	0.159
Rainfall (mm)	-0.002 (-0.006, 0.002)	0.855	0.002	0.355
Wind Speed (Kmph)	0.022 (-0.042, 0.087)	0.463	0.032	0.496
Humidity (%)	-0.002 (-0.016, 0.011)	0.105	0.006	0.746
Sunshine (hr)	0.003 (-0.001, 0.007)	2.429	0.002	0.119
According to age groups				
*2-6 months**				
Temperature (^{0}C)	0.012 (-0.004, 0.028)	.008	2.192	0.139
Rainfall (mm)	-0.001 (-0.003, 0.001)	.001	1.599	0.206
Wind Speed (Kmph)	0.059 (0.037, 0.081)	.011	28.241	<0.01
Humidity (%)	0.006 (0.001, 0.011)	.003	5.276	0.022
Sunshine (hr)	0.002 (0.000, 0.003)	.001	4.615	0.032
7-12 months[#]				
Temperature (^{0}C)	0.038 (0.018, 0.057)	.010	14.288	<0.01
Rainfall (mm)	-0.001 (-0.003, 0.002)	.001	.447	0.504
Wind Speed (Kmph)	0.08 (0.054, 0.107)	.013	36.037	<0.01

For peer review only

Humidity (%)	0.012 (0.006, 0.018)	.003	14.660	<0.01
Sunshine (hr)	0.002 (0.000, 0.004)	.001	5.144	0.023
*13-24 months**				
Temperature (^{0}C)	0.026 (0.006, 0.047)	.010	6.460	0.011
Rainfall (mm)	-0.002 (-0.005, 0.000)	.001	3.564	0.059
Wind Speed (Kmph)	0.102 (0.077, 0.128)	.013	60.620	<0.01
Humidity (%)	0.017 (0.010, 0.024)	.004	22.599	<0.01
Sunshine (hr)	0.002 (0.000, 0.004)	.001	3.211	0.073

*Note: Tests used for regression analysis: *Poisson Regression; # Negative Binomial Regression*

For peer review only

802 **Figure**

803 Figure 1: Monthly distribution of intussusception cases in children during 2010-2017

For peer review only

For peer review only - http://bmjopen.bmj.com/site/about/guidelines.xhtml

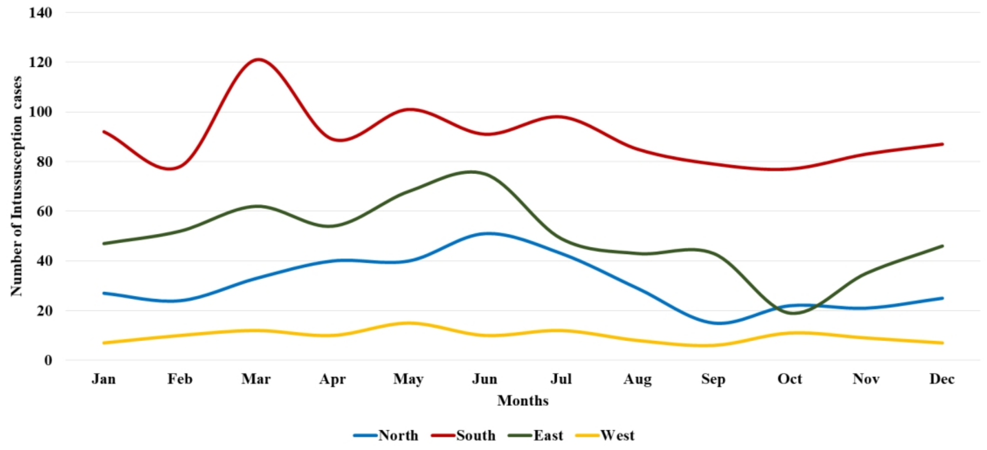

Figure 1: Monthly distribution of primary intussusception cases in children during 2010-2017

155x71mm (300 x 300 DPI)

Title: Association of meteorological parameters with intussusception in children aged under-two years: Results from a multisite bidirectional surveillance over seven years in India

Supplementary Documents

Index

Sl no	Details
1	Supplementary Figure 1: The study sites and their location on India map indicating the study regions
2	Supplementary Table 1: The International Classification of Diseases (ICD) codes for review of the cases from medical records
3	Supplementary Table 2: The seasons and months at the sites according to the regions
4	Supplementary Table 3: The monthly median (and IQR) meteorological data during 2010 and 2017 for regions
5	Supplementary Table 4: Monthly median (and IQR) meteorological data during 2010 and 2017 at the study sites
6	Supplementary Figure 2: The pattern of monthly meteorological parameters across the regions
7	Supplementary Figure 3: The pooled monthly (SF3a) and seasonal (SF3b) distribution of intussusception in children during July 2010 to September 2017
8	Supplementary Table 5. Pearson correlation analysis between monthly intussusception cases and monthly median meteorological parameters for the study sites
9	Supplementary Table 6. Spearman's Rank correlation analysis between monthly intussusception cases and monthly median meteorological parameters for the study sites
10	Supplementary Table 7. Regression analysis of the association between the intussusception cases and meteorological parameters for the study sites
11	Supplementary Table 8. Intussusception cases in children at different temperature ranges at pooled and regional level
12	Supplementary Table 9. Variations in the intussusception cases between the different temperature categories by Kruskal-Wallis H test
13	Supplementary Figure 4: Association of monthly intussusception cases with mean monthly temperatures
14	Supplementary Figure 5: Association of monthly intussusception cases with mean monthly humidity
15	Supplementary Figure 6: Association of monthly intussusception cases with mean monthly wind speed
16	Supplementary Figure 7: Association of monthly intussusception cases with mean monthly sunshine
17	Supplementary Figure 8: Association of monthly intussusception cases with mean monthly rainfall
18	Supplementary Table 10. Factorial analysis-of-variance of intussusception in children with season and age groups
19	Supplementary Table 11. Factorial analysis-of-variance of intussusception in children with season and regions

For peer review only

20	Supplementary Table 12. Multiple comparisons of seasonal intussusception in children in different seasons pooled for all sites
21	Supplementary Table 13. Multiple comparisons of seasonal intussusception in children in different seasons at regional level
22	Supplementary Table 14. Multiple comparisons of seasonal intussusception in children in different seasons for different age groups

For peer review only

Supplementary Figure 1: The study sites and their location on India map indicating the study regions

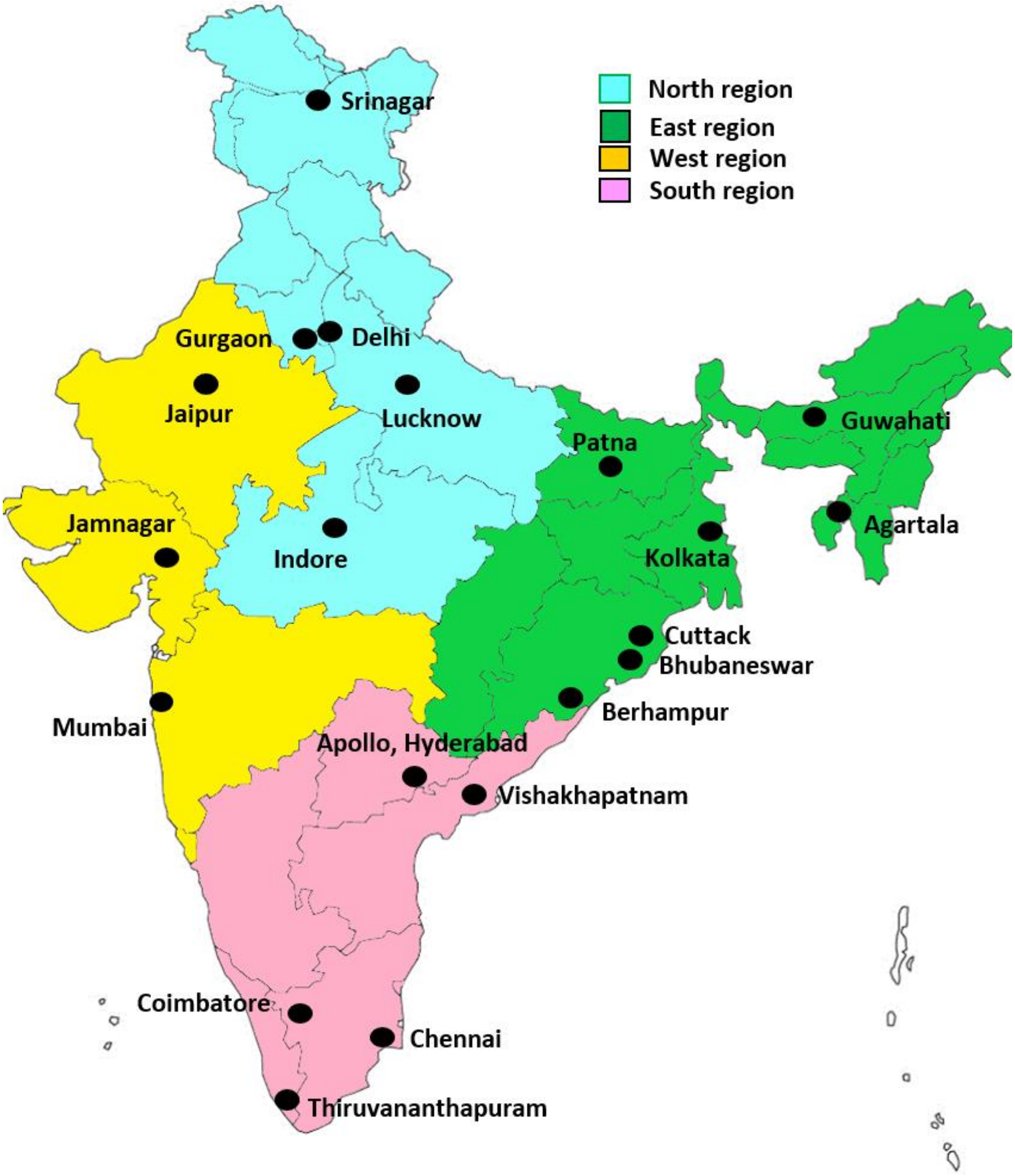

Note: The map has been prepared for use in the research project. The corresponding author is the creator of the image.

For peer review only - http://bmjopen.bmj.com/site/about/guidelines.xhtml

Supplementary Table 1: The International Classification of Diseases (ICD) codes for review of the cases from medical records

Clinical conditions considered as suspected cases	Codes	
	ICD 10	ICD 9
Intussusception	K56.1	560.0
Volvulus	K56.2	560.2
Gallstone ileus	K56.3	560.31
Other impaction of intestine	K56.4	560.30
Intestinal adhesions with obstruction	K56.5	560.81
Other and unspecified intestinal obstruction or Acute intestinal obstruction or Subacute intestinal obstruction	K56.6	560.9
Ileus, unspecified	K56.7	560.1
Paralytic ileus	K56.0	
Acute abdomen	R10.0	789.0
Blood in stool with vomiting	K92.1/K92.2 with R11.2	578.1/578.9 with 787.01

For peer review only

Supplementary Table 2: The seasons and months at the sites according to the regions

Region/ Site (state)	Jan	Feb	Mar	Apr	May	Jun	Jul	Aug	Sep	Oct	Nov	Dec
North region												
Srinagar (Jammu & Kashmir)	W	W	W	Sp	Sp	Su	Su	M	M	A	W	W
New Delhi (Delhi)	W	Sp	Sp	Su	Su	Su	M	M	M	A	A	W
Gurgaon (Haryana)	W	Sp	Sp	Su	Su	Su	M	M	M	A	A	W
Lucknow (Uttar Pradesh)	W	W	Sp	Su	Su	Su	M	M	M	A	A	W
Indore (Madhya Pradesh)	W	W	Sp	Su	Su	Su	M	M	M	A	A	W
South region												
Vishakhapatnam (Andhra Pradesh)	W	W	Su	Su	Su	M	M	M	M	A	A	W
Hyderabad (Telangana)	W	Sp	Su	Su	Su	M	M	M	M	A	A	W
Coimbatore (Tamil Nadu)	W	Sp	Su	Su	Su	M	M	M	M	A	A	W
Chennai (Tamil Nadu)	W	Sp	Su	Su	Su	M	M	M	M	A	A	W
Trivandrum (Kerala)	W	Sp	Su	Su	Su	M	M	M	M	A	A	W
East region												
Patna (Bihar)	W	Sp	Sp	Su	Su	Su	M	M	M	A	A	W
Bhubaneswar (Odisha)	W	Sp	Su	Su	Su	M	M	M	A	A	A	W
Cuttack (Odisha)	W	Sp	Su	Su	Su	M	M	M	A	A	A	W
Berhampur (Odisha)	W	Sp	Su	Su	Su	M	M	M	A	A	A	W
Kolkata (West Bengal)	W	Sp	Su	Su	Su	M	M	M	A	A	A	W
Guwahati (Assam)	W	W	Su	Su	Su	M	M	M	A	A	A	W
Agartala (Tripura)	W	W	Su	Su	Su	M	M	M	A	A	A	W
West region												
Jaipur (Rajasthan)	W	W	Sp	Su	Su	Su	M	M	M	A	A	W
Jamnagar (Gujarat)	W	Sp	Su	Su	Su	M	M	M	M	A	A	W
Mumbai (Maharashtra)	W	Sp	Su	Su	Su	M	M	M	M	A	A	W

Note: The seasons and colour codes used

A	Autumn	M	Monsoon	Sp	Spring	Su	Summer	W	Winter

For peer review only - http://bmjopen.bmj.com/site/about/guidelines.xhtml

Supplementary Table 3: The monthly median (and IQR) meteorological data during 2010 and 2017 for regions

Region/ Months	Temperature (°C)	Rainfall (mm)	Wind speed (Kmph)	Humidity (%)	Sunshine (hours)
North region					
January	14 (11, 16)	5.7 (0.5, 13.2)	8.5 (7.1, 9.8)	54 (47, 61)	223 (217.5, 232.5)
February	17 (14, 20)	6.9 (0.5, 22.6)	9.6 (8.1, 10.3)	47 (39, 62)	266 (239, 277.5)
March	23 (21, 25)	4.9 (1.1, 19.9)	9.9 (9, 10.5)	37 (26, 45)	301 (291.5, 308.5)
April	31 (28, 32)	3.9 (1.3, 12.6)	10.5 (8.1, 12.2)	23 (18, 28)	337.5 (315, 345)
May	35 (34, 36)	3.3 (1.1, 14.3)	10.7 (8.9, 12.3)	22 (19, 30)	386 (383, 387.5)
June	35 (31, 37)	22 (8.1, 48.8)	11.1 (9.1, 12.9)	40 (31, 46)	361.5 (346.5, 369.5)
July	31 (26, 33)	72.5 (30, 126.7)	9.6 (8, 12.3)	59 (54, 69)	331.8 (279.3, 367)
August	29 (24, 31)	55.1 (26.3, 93.7)	9 (7, 10.8)	66 (56, 80)	337.8 (298.8, 359.3)
September	28 (25, 30)	31.6 (13.4, 58.1)	8.4 (7.2, 9.2)	56 (49, 76)	286.3 (269.8, 300)
October	26 (25, 27)	1.5 (0.2, 12.2)	6.5 (5.9, 7.2)	42 (34, 50)	250 (244, 262.5)
November	21 (20, 22)	0.1 (0, 3.2)	7.5 (6, 8.2)	33 (31, 39)	225 (222, 225)
December	16 (14, 18)	0.6 (0, 4.4)	7.8 (6.7, 8.8)	39 (35, 45)	231 (225.5, 232.5)
South region					
January	23 (23, 25)	2.8 (1.1, 7.7)	9.7 (8.6, 12.1)	67 (63, 71)	277.5 (274.5, 296.5)
February	25 (24, 26)	1.5 (0.4, 8)	10.1 (8.8, 12.2)	65 (51, 71)	277.5 (272.5, 286)
March	28 (27, 28)	10.1 (3.4, 29.8)	10.9 (8.9, 13.6)	66 (50, 71)	299.5 (294, 308.5)
April	30 (29, 31)	7.8 (2.9, 32.2)	12.1 (9.7, 17.1)	69.5 (52, 73)	317.5 (292.5, 333.5)
May	31 (29, 32)	20.2 (9.1, 30.5)	14.6 (12.9, 17.6)	64 (59, 71)	353.5 (317, 378)
June	28 (27, 31)	50.7 (16.5, 97.8)	18 (17.2, 18.5)	65 (62, 70)	291 (268.5, 319.5)
July	28 (26, 29)	43 (1.7, 78.5)	18.5 (17, 20.1)	68 (63, 74)	283.3 (255.5, 308.5)
August	27 (26, 29)	58.1 (7.9, 92.3)	16.2 (14.4, 17.6)	72 (66, 77)	275.5 (245.5, 296.5)
September	27 (26, 29)	61.7 (34.1, 99.9)	13.8 (12.3, 16.3)	72 (67, 76)	258 (239, 288)
October	27 (26, 28)	61.9 (44.4, 117.6)	10.6 (9.4, 12.2)	71 (68, 75)	246.8 (224.5, 267)
November	25 (24, 27)	34.8 (19.2, 108.9)	9.6 (7.2, 13.5)	72 (69, 75)	205.5 (191, 213)
December	24 (23, 26)	15 (3, 37.9)	10 (8.5, 14.3)	70.5 (66, 74)	225.5 (216, 265.5)
East region					
January	20 (17, 22)	1.7 (0.1, 5.8)	7.2 (6.4, 8.3)	60 (54, 64)	227 (224.5, 231)
February	23 (20, 24)	2.7 (1, 8.7)	8.2 (7, 9)	54 (49, 61)	210 (207, 237)
March	27 (25, 28)	8.5 (2.9, 19.4)	10 (8.9, 11)	56 (46, 60)	291.5 (287.5, 301)
April	31 (29, 32)	12.3 (3.5, 32.1)	12.5 (10.4, 14.6)	60 (54, 66)	355.5 (294.5, 370.5)
May	32 (30, 33)	25.2 (13.9, 54.5)	13.3 (11.2, 15)	66 (60, 69)	365.5 (347, 374.5)
June	32 (29, 33)	79.8 (53.9, 122.3)	12 (10.6, 13.1)	66 (62, 76)	307.5 (292.5, 327)
July	30 (28, 30)	118 (82.6, 175.2)	11.3 (9.8, 12.4)	73 (69.5, 83.5)	276.8 (250, 288.5)
August	29 (28, 30)	100 (70.8, 154.9)	10 (8.9, 11.1)	78 (73.5, 82)	286 (258, 305)
September	28 (28, 29)	89.9 (65.7, 133)	8.3 (7.3, 9.4)	79.5 (75, 83)	252.5 (236.3, 262)
October	26 (25, 27)	44.7 (24.8, 87.9)	6.7 (5.9, 7.6)	77 (71, 82)	287 (271.5, 295)
November	23 (21, 24)	3.6 (0.4, 20.4)	6.4 (5.7, 7.3)	66 (60, 77)	270 (256, 292.5)
December	21 (18, 22)	1 (0.2, 6.7)	7.1 (6.1, 8)	59 (50, 70)	229.5 (222.5, 240)
West region					
January	22 (15, 24)	0.1 (0, 0.9)	10 (8.5, 14.4)	48 (44, 51)	310 (255, 310)
February	24 (20, 25)	0.4 (0, 1)	10.8 (9.9, 14.5)	45 (44, 53)	278.5 (275.5, 281)
March	27 (25, 28)	0.2 (0, 1.2)	10.7 (10.1, 15.9)	47 (29, 53)	307.5 (305.5, 308.5)
April	29 (29, 31)	0.4 (0, 1.7)	11.2 (10, 18.3)	56 (20, 61)	300 (300, 315.5)

For peer review only - http://bmjopen.bmj.com/site/about/guidelines.xhtml

Region/ Months	Temperature (°C)	Rainfall (mm)	Wind speed (Kmph)	Humidity (%)	Sunshine (hours)
May	31 (30, 36)	0.6 (0.1, 5.5)	14.3 (10.7, 23.9)	65 (21, 68)	355 (307, 381.5)
June	31 (29, 36)	48.9 (9.7, 156.7)	16.7 (13.9, 21.7)	68 (34, 75)	311.5 (252, 359.5)
July	29 (28, 32)	121.3 (58.1, 391.4)	17.3 (13.9, 23.8)	77.5 (56.5, 82.5)	194 (158.5, 338.8)
August	28 (27, 29)	118.5 (60, 213.5)	14.1 (11.4, 22.1)	79 (73.5, 84)	196.8 (166.5, 278)
September	28 (27, 29)	70.6 (19.4, 133.8)	11.6 (9.8, 17.8)	74 (62.5, 80)	253.5 (229, 274.8)
October	28 (28, 29)	5.9 (0.5, 26.1)	9 (8.1, 12.5)	51 (41, 67)	296.5 (265, 308.5)
November	27 (23, 28)	0.1 (0, 2.3)	9 (8, 13)	44 (34, 53)	230 (225, 251.5)
December	24 (17, 25)	0 (0, 0.5)	9.8 (8.4, 14.5)	43 (39, 49)	252.5 (232.5, 293.5)

Note: Temperature (°C); Rain: Rainfall (millimetre, mm); Wind Speed (miles/second); Humidity (%); Sunshine (in hours/month)

For peer review only

For peer review only - http://bmjopen.bmj.com/site/about/guidelines.xhtml

Supplementary Table 4: Monthly median (and IQR) meteorological data during 2010 and 2017 at the study sites

Region/ Place (State) and months	Temperature (°C)	Rainfall (mm)	Wind speed (Kmph)	Humidity (%)	Sunshine (hour)
1. North region					
1.1 Srinagar (Jammu and Kashmir)					
January	-3 (-4,-1)	28.4 (16.8,41.9)	5.8 (5.3,6.3)	58 (44,69)	209.5 (205.5,218)
February	0 (-2,2)	69.9 (56.8,89)	5.7 (5.4,6.1)	60 (59,68)	241 (220,261.5)
March	4 (3,5)	63.4 (44.2,107)	5.3 (4.7,6.5)	60 (57,63)	282 (232.5,296)
April	8 (7,9)	45.3 (36.7,84.2)	4.6 (4.4,5)	55 (52,57)	330 (301.5,336)
May	13 (11,14)	31.6 (16.3,57.3)	4.3 (3.7,5.1)	49 (46,50)	387.5 (378.5,387.5)
June	17 (16,18)	15.8 (8.1,33.4)	4.2 (3.8,4.4)	46 (41,47)	372 (364.5,375)
July	19 (18.5,19)	22.6 (13,50.5)	5.4 (4.7,6)	57.5 (57,59)	387.5 (381.5,387.5)
August	18 (18,19)	25 (20.6,34.1)	5 (3.8,5.4)	59.5 (56,65)	371 (368,372.5)
September	15 (14,15)	9 (5.6,48.8)	3.9 (3.6,4.1)	50.5 (48,52.5)	300 (297.8,300)
October	9 (8,11)	10.5 (4.1,15.8)	3.3 (2.8,3.9)	40 (34,41)	262.5 (258.5,262.5)
November	4 (3,5)	8 (1.4,12.1)	4.6 (4.1,6)	33 (31,36)	222 (209.5,225)
December	0 (-1,1)	14.9 (5.3,24.9)	5.8 (5.4,6.7)	31 (27,42)	221.5 (211.5,228)
1.2 Delhi (Delhi) and 1.3. Gurgaon (Haryana)					
January	13 (11,14)	1 (0.3,5.7)	8.5 (7.9,9.2)	54 (53,62)	223 (220.5,228.5)
February	16 (15,18)	5.3 (0.2,7.8)	9.8 (8.9,10.1)	53 (43,63)	269.8 (266,277.5)
March	22 (21,23)	2.9 (1.1,6.5)	9.9 (9.2,10.4)	41 (33,43)	300 (292,303)
April	30 (29,32)	5.3 (2.7,9.8)	10.5 (8.4,10.7)	23 (17,27)	341.8 (331.5,344.5)
May	36 (35,36)	2.4 (0.9,6.7)	10.2 (9.8,10.7)	20.5 (15,22)	386 (383,386)
June	36 (36,38)	20.7 (8.5,30.9)	10.1 (9.8,11.8)	32 (23,36)	363.5 (361.5,369.5)
July	32.5 (32,34)	61.2 (30.7,98.2)	8.9 (8.1,10.2)	53.5 (51,61.5)	345 (331.8,365)
August	31 (30,32.5)	47.8 (28.5,78.9)	8.4 (7.7,9.7)	63.5 (54,72.5)	339.8 (329,349)
September	30.5 (29,31)	27.2 (11.9,61.1)	8.2 (7.7,8.6)	49.5 (47,67)	279.5 (272.3,294)
October	27 (26,28)	0.5 (0,1.1)	6.5 (6.2,7)	34 (31,43)	250 (248.5,250)
November	21 (21,22)	0 (0,0.2)	8.1 (6.5,8.2)	31 (27,40)	225 (223.5,225)
December	16 (15,17)	0.3 (0,1.5)	8.3 (6.9,9)	39 (35,46)	230.5 (225.5,231)
1.4. Lucknow (Uttar Pradesh)					
January	15 (14,16)	8.1 (4.7,9.6)	9.8 (8.6,10.1)	54 (53,58)	224 (219,229.5)
February	18 (17,20)	6.9 (0.5,22.6)	10.1 (9.8,11.3)	52 (42,58)	230.5 (224.5,239)
March	25 (24,25)	3.1 (2.6,10.5)	11.2 (11.1,12)	35 (26,37)	309.5 (303.5,315)
April	32 (32,34)	2 (1.3,3.9)	12.6 (12,13.4)	21 (18,26)	373.5 (363,375)
May	36 (36,37)	5.2 (0.2,6.9)	11.4 (11.2,12.3)	21 (18,25)	384.5 (380,386)
June	37 (34,39)	13.4 (3.2,100.6)	11.6 (11.2,12.9)	32 (25,45)	346.5 (321.5,362.5)
July	32 (31,33)	127.8 (83.6,151.1)	11.1 (10.2,12.3)	63 (57,69)	304.3 (289,321)
August	30 (30,31.5)	76.5 (50.8,95.9)	10.7 (9.4,11.1)	70.5 (58,74.5)	313.8 (304,344.8)
September	29.5 (28.5,30.5)	30.9 (21.1,61.8)	9.1 (8.5,9.6)	64.5 (55,75.5)	316 (301.3,328.3)
October	26 (25,27)	7.4 (0.2,31.3)	6.9 (6.1,7.9)	50 (42,62)	227.5 (225.5,238.5)
November	22 (21,22)	0 (0,0.7)	7.4 (6.9,8.5)	35 (34,48)	225 (218,225)
December	17 (17,18)	0.6 (0,1.6)	8.5 (7.6,9.9)	41 (38,46)	232.5 (231,232.5)
1.5 Indore (Madhya Pradesh)					
January	18 (17,19)	0.8 (0.4,9.1)	9.2 (8,10.5)	44 (32,47)	281 (277.5,282.5)
February	22 (21,23)	1 (0.2,11.1)	9.6 (8.6,11)	32 (29,39)	280 (277,290)
March	27 (27,27)	2.9 (0,6.4)	10 (9.8,10.4)	21 (18,26)	307 (301,310)
April	32 (32,33)	0.6 (0.2,2.8)	12.3 (11.2,14.4)	18 (17,21)	315 (310,317.5)
May	35 (34,35)	1.9 (0.6,2.7)	15.3 (14.7,16.8)	24 (22,30)	386 (384.5,387.5)
June	31 (31,32)	48.9 (29.6,72.2)	15.4 (14.6,16.9)	51 (47,57)	341 (294,352)

For peer review only - http://bmjopen.bmj.com/site/about/guidelines.xhtml

Region/ Place (State) and months	Temperature (°C)	Rainfall (mm)	Wind speed (Kmph)	Humidity (%)	Sunshine (hour)
July	27 (26,27)	124.5 (83.8,186.7)	15.5 (12.6,17.3)	80 (75,81.5)	201.5 (178.8,239)
August	25.5 (24.5,26)	101.7 (78.8,132.8)	12.1 (11.4,14.9)	88.5 (80,90)	166.5 (144.8,208.3)
September	25.5 (25,26)	52.2 (41,62.1)	9.7 (8.6,10.5)	77 (75,81)	253.5 (241.5,267.3)
October	26 (25,26)	2.3 (0.3,31.1)	7.2 (6.6,8.3)	45 (40,58)	271 (254.5,272.5)
November	23 (22,24)	0.3 (0,2.9)	7.6 (6.4,8)	37 (33,39)	225 (220.5,225)
December	19 (19,20)	0.1 (0,1.2)	8.1 (7.4,8.8)	36 (34,40)	232.5 (229.5,232.5)
2. East region					
2.1 Bhubaneswar and 2.2. Cuttack (Odisha)					
January	22 (21,22)	1.5 (0.1,5.6)	7.2 (6.4,7.4)	56.5 (56,60)	224.5 (221.5,228)
February	24.5 (24,25)	2 (0.5,8.9)	8.2 (7.1,9.3)	56 (53,62)	209.3 (202.5,210)
March	28 (28,29)	6.7 (3.6,16.6)	10.9 (9.4,11.5)	57.5 (54,62)	287.3 (275.5,290)
April	31 (31,32)	5.4 (1.6,12.3)	15 (12.4,17.2)	60.5 (57,66)	366.8 (360,372)
May	32 (32,33)	22.3 (15.4,26.9)	14.9 (13.9,15.8)	64.5 (63,67)	366.8 (362.5,375.5)
June	32 (32,33)	80 (60.3,114.7)	12.2 (12,13.7)	62.5 (62,66)	300.8 (292.5,322.5)
July	30 (30,30)	118 (80.3,137.4)	11.3 (10.1,12.1)	72 (69,73)	269.8 (253.3,283.8)
August	29 (29,30)	92.6 (67,133.3)	10.4 (9.2,11.4)	74.5 (71.5,77.5)	285 (262.3,296)
September	29 (28,29)	89.9 (67.3,112.9)	8.7 (7.3,9.8)	77 (74.5,79.5)	248.3 (234.3,257.3)
October	27 (26,27)	50.1 (25.7,89.9)	7.4 (6.7,7.9)	74 (69,82)	285.5 (263,290)
November	23.5 (23,25)	9.1 (1,28.3)	6.6 (6.2,7.2)	64.5 (60,66)	261.8 (256,268.5)
December	21 (21,22)	4.7 (0.3,11.8)	6.9 (6.6,8)	57 (53,59)	223.8 (207,229.5)
2.3 Berhampur (Odisha)					
January	22 (21,22)	1.6 (0.6,5.6)	6.9 (6.6,7.6)	66 (63,69)	224.5 (221.5,228)
February	24 (24,24)	3.6 (2.1,15.7)	8.2 (7.2,8.7)	62 (61,68)	210 (205.5,210)
March	28 (27,28)	8.5 (1.3,20.6)	10.8 (9.8,11.7)	61 (59,64)	288.5 (275.5,291)
April	30 (30,31)	9.7 (2,20.6)	13.9 (12.4,14.6)	63 (58,67)	367.5 (361.5,372)
May	32 (32,32)	13.3 (7.1,28.9)	13.3 (12.5,13.7)	67 (64,68)	368 (364,380)
June	31 (31,32)	60.7 (48.2,79.8)	11.3 (11.1,11.8)	67 (64,69)	307.5 (300,324.5)
July	29 (29,30)	82.9 (65.6,107.6)	11.1 (10.5,11.4)	73 (71,74.5)	283.5 (279.3,289)
August	29 (29,29)	93.5 (72.6,119.7)	9.4 (9.1,10)	74.5 (73,78)	294.3 (272,302.5)
September	28.5 (28,29)	91.1 (67,116)	8.1 (7.5,8.8)	76 (75,77.5)	254.8 (250.3,261.5)
October	27 (26,27)	44.9 (33.2,132.7)	8.1 (6.6,9)	72 (70,80)	289.5 (263,295)
November	24 (24,24)	44.5 (0.4,59.7)	7 (6.6,8.3)	67 (63,71)	262 (256,271.5)
December	22 (22,22)	3.3 (1,30.7)	7.8 (7,8.2)	62 (60,67)	225 (207,229.5)
2.4. Kolkata (West Bengal)					
January	20 (19,21)	0.1 (0,7.7)	9.1 (8.6,9.9)	44 (40,52)	228 (225,231)
February	23 (23,24)	4.4 (0.8,7.8)	9 (8.1,9.3)	45 (40,46)	211 (204.5,212.5)
March	28 (27,28)	8.7 (1.7,19.2)	10 (9.4,10.2)	50 (46,57)	304 (295,308.5)
April	31 (31,33)	12.5 (5.2,37.2)	14.2 (10.3,15.2)	58 (54,63)	300 (287.5,312)
May	33 (32,33)	16.2 (11.2,28.5)	15 (13.7,17.7)	61 (59,67)	371 (369.5,377.5)
June	32 (31,33)	63.7 (52.7,92.7)	13.1 (12.8,15.8)	65 (64,72)	318.5 (290.5,327)
July	30 (29.5,30)	119.7 (102.1,171.9)	12.6 (11.5,13.2)	76 (74,78)	258.8 (243.3,271.3)
August	29 (29,30)	102.7 (86,153.2)	11 (10.3,11.1)	80.5 (76,82)	277.3 (247.8,284.5)
September	28 (28,29.5)	81.4 (74.9,111.4)	9.1 (8.1,10.3)	81.5 (77,85)	236.3 (226.8,241.3)
October	27 (26,27)	27.2 (23.6,34.9)	7.3 (6.5,8.7)	72 (71,80)	280 (265.5,287.5)
November	24 (23,25)	3.3 (0.3,16.7)	7.5 (6.1,9.3)	57 (53,65)	292.5 (284,297)
December	21 (20,22)	0.1 (0,1.8)	8.7 (8.5,9.5)	47 (41,50)	230 (218,232.5)
2.5. Patna (Bihar)					
January	16 (15,17)	5.8 (0.8,8.3)	9.5 (9.4,10)	54 (51,63)	229.5 (222.5,232.5)
February	19 (18,21)	0.9 (0.4,2.7)	10.4 (9.7,11.8)	49 (47,54)	207.5 (202.5,213)
March	26 (25,26)	2.9 (1.6,5.5)	11.1 (10.1,11.7)	32 (30,33)	303.5 (291.5,305)

For peer review only - http://bmjopen.bmj.com/site/about/guidelines.xhtml

Region/ Place (State) and months	Temperature (°C)	Rainfall (mm)	Wind speed (Kmph)	Humidity (%)	Sunshine (hour)
April	33 (32,33)	6.5 (2.4,22.2)	12.3 (11.4,14.2)	28 (20,31)	373.5 (358.5,375)
May	35 (35,37)	23.2 (5,41.1)	13 (12.3,13.6)	35 (29,39)	377 (366.5,386)
June	36 (34,37)	47.6 (21.1,58.6)	11.9 (11.5,13.6)	42 (42,51)	337 (327,355)
July	32 (31,32.5)	150.4 (120.4,160.4)	12 (11.7,14)	65.5 (60.5,68.5)	283 (257.5,312.5)
August	31 (31,32)	63.4 (38.5,138.2)	10.6 (9.3,11.7)	65 (61.5,71.5)	318.5 (293,329.8)
September	29 (28.5,30.5)	66.7 (32.4,116.6)	8.7 (8.4,10.4)	67 (60.5,80)	273 (249.5,282.5)
October	26 (25,27)	29.1 (8.1,54.9)	6.8 (5.9,7.5)	63 (51,78)	297 (271.5,304)
November	22 (21,23)	0 (0,0.3)	6.8 (6.3,8.1)	45 (39,61)	232.5 (231,235)
December	18 (18,19)	0 (0,0.7)	8 (7.5,9.1)	49 (38,54)	231 (225,232.5)
2.6. Guwahati (Assam)					
January	17 (15,17)	3.9 (0.2,10.3)	3.9 (3.8,4.3)	67 (64,76)	228 (225,231)
February	20 (19,20)	4.9 (2.3,10.2)	4.2 (4,4.5)	56 (54,62)	245 (242,253.5)
March	24 (23,24)	14.1 (1.4,22.9)	4.9 (4.6,4.9)	46 (42,58)	299.5 (295,310)
April	26 (25,27)	39.7 (15.1,80.8)	4.5 (4.4,4.8)	62 (50,68)	292.5 (280.5,300)
May	27 (27,28)	83.1 (54.5,122.4)	4.2 (3.8,5)	69 (63,73)	344 (336.5,357)
June	28 (27,29)	236.2 (191.4,326.2)	3.8 (3.6,4.3)	81 (76,84)	323.5 (292.5,334)
July	27.5 (27,28)	344.4 (224.2,407.4)	3.8 (3.5,4.1)	87 (83.5,88.5)	317.8 (274.5,332.8)
August	28 (27.5,28)	235.2 (214.1,276.4)	3.8 (3.7,4.2)	84 (82,85)	297.3 (283,310.5)
September	27 (26.5,27)	159.3 (143.5,217.6)	3.4 (3.1,4)	84 (81.5,85)	272 (257.8,274.8)
October	24 (24,24)	48.8 (12.8,72.7)	3.6 (3.4,4.2)	79 (77,81)	294 (283,305.5)
November	20 (20,20)	4.5 (1,7.3)	3.8 (3.7,4.5)	78 (71,80)	294 (292,297)
December	17 (17,18)	2.5 (1.1,9)	4 (3.8,4.2)	74 (73,77)	250.5 (240,253.5)
2.7. Agartala (Tripura)					
January	18 (17,19)	0.3 (0.1,2.2)	6.7 (6.5,7.2)	61 (58,69)	231 (228,232.5)
February	21 (20,22)	5.6 (1.7,10.5)	7.2 (7,8.6)	53 (51,57)	246 (237,250.5)
March	25 (24,26)	22.6 (11.8,53.2)	8.9 (8.8,9.5)	57 (51,64)	295.5 (288,301)
April	28 (28,29)	113.4 (38.1,141.2)	11.1 (8.5,12.5)	67 (64,73)	266.5 (246,297)
May	29 (28,30)	113.8 (73.4,194.5)	9.7 (9.1,11.5)	74 (74,80)	307.5 (299.5,320)
June	29 (28,29)	99.2 (72.8,110.5)	11.1 (10.6,13.4)	82 (79,85)	286.5 (282.5,303.5)
July	28 (28,28)	73 (50.4,103.5)	12.1 (10.4,13.4)	84.5 (83.5,85.5)	261.8 (244.8,276)
August	28 (27.5,28)	78.4 (61.2,101.3)	10.6 (9.4,11.1)	86.5 (82,87.5)	249.3 (231.8,271)
September	27 (27,28)	57.1 (43.3,144.7)	8.4 (7.5,9.4)	86.5 (82,89)	249 (225,259)
October	25 (25,26)	55.3 (23,121.8)	5.9 (4.9,6)	85 (82,87)	284.5 (271,293.5)
November	21 (21,22)	5.2 (0.2,31)	5.5 (5.3,6.1)	78 (77,79)	300 (282,300)
December	18 (18,20)	0.7 (0.2,6.7)	6.1 (5.1,6.2)	71 (70,73)	264.5 (258.5,267.5)
3. South region					
3.1. Vishakhapatnam (Andhra Pradesh)					
January	23 (23,24)	3.5 (1.3,6.1)	10.6 (9.4,11.1)	68 (67,72)	277.5 (274.5,277.5)
February	25 (24,25)	1.4 (0.9,4.2)	10.1 (9.4,10.8)	68 (65,70)	280 (277.5,288.5)
March	27 (27,28)	3.4 (3.3,16.2)	13.6 (11.5,14)	70 (65,70)	308.5 (298,310)
April	29 (29,30)	5 (1.3,12.6)	17.4 (16.7,19.1)	71 (70,74)	330 (320,335)
May	31 (31,31)	8.6 (3.5,18)	17 (15.9,18.5)	71 (70,72)	378 (371,381.5)
June	31 (31,31)	91 (40.8,129.1)	16.9 (14.3,17.3)	70 (66,71)	274.5 (268.5,306)
July	29.5 (29,30)	77.6 (60.1,105.1)	18.5 (16.2,20.1)	73 (71.5,74.5)	252.5 (226.8,289.8)
August	29 (29,30)	111.7 (96.7,120.4)	13.8 (13.5,16.6)	74.5 (73,77)	241 (234.5,263.5)
September	29 (29,29)	127 (104.7,164.2)	11.8 (11.7,13)	76 (75.5,77)	234.5 (226.3,243.8)
October	27 (27,28)	90.4 (50.2,182.7)	11.7 (9.1,13.2)	72 (69,77)	222 (205.5,236.5)
November	25 (25,26)	46.2 (19.2,144.8)	12.1 (11.5,15.1)	70 (66,71)	207 (202,217.5)
December	24 (24,24)	4.2 (2.6,14.6)	13.8 (12,14.4)	67 (66,70)	225.5 (222,228)

For peer review only - http://bmjopen.bmj.com/site/about/guidelines.xhtml

Region/ Place (State) and months	Temperature (°C)	Rainfall (mm)	Wind speed (Kmph)	Humidity (%)	Sunshine (hour)
3.2. Hyderabad (Telangana)					
January	22 (21,23)	0.5 (0.1,1.5)	12.1 (10.1,12.6)	47 (46,51)	277.5 (274.5,277.5)
February	24 (24,25)	0 (0,1.6)	12.4 (10.6,12.9)	41 (38,45)	280 (277.5,288.5)
March	28 (28,29)	3.4 (0.1,10.6)	11.1 (10.3,11.9)	34 (32,39)	308.5 (298,310)
April	32 (31,33)	2.9 (1.5,5.3)	9.7 (9.2,11.9)	33 (27,35)	330 (320,335)
May	34 (33,35)	9.4 (7.3,17.6)	12.9 (12,14.4)	30 (28,34)	378 (371,381.5)
June	29 (28,29)	111.6 (33.3,131.2)	18.7 (18.1,20.7)	57 (49,63)	274.5 (268.5,306)
July	26 (26,27.5)	66.4 (47.1,81)	20.7 (18.4,23)	72.5 (65,75)	252.5 (226.8,289.8)
August	26 (25,26.5)	68.6 (36,77.7)	16.6 (15.3,17.5)	76 (70,78)	241 (234.5,263.5)
September	26 (25,26)	51.9 (40.3,74.1)	12.8 (11.3,13.9)	74.5 (70.5,78.5)	234.5 (226.3,243.8)
October	24 (24,25)	35.9 (14.9,45.2)	10.1 (9.2,10.6)	65 (59,70)	222 (205.5,236.5)
November	22 (21,23)	3.1 (0.5,18.8)	10.2 (9.5,10.8)	60 (51,62)	207 (202,217.5)
December	21 (20,22)	0.3 (0.2,1.1)	10.3 (10.1,11.3)	50 (49,53)	225.5 (222,228)
3.3. Coimbatore (Tamil Nadu)					
January	23 (23,23)	2.2 (1.1,26.6)	7.4 (6.9,8.6)	63 (63,66)	290 (285,305.5)
February	25 (24,25)	3 (0.9,8.3)	7.8 (7.4,8.9)	55 (51,56)	277 (272,286)
March	28 (27,28)	26.7 (10.1,60.5)	7.1 (7.1,8.6)	53 (50,55)	299.5 (298,302.5)
April	30.5 (30,31)	8.9 (6.7,28.2)	9.2 (8.3,11.6)	52 (52,54)	297.5 (284.5,297.5)
May	30 (29,30)	29.7 (29.7,30.5)	12.4 (11.9,13.2)	59 (59,61)	334 (317,335)
June	27 (27,27)	4.9 (4.9,6.7)	18.1 (17.7,18.3)	64 (64,66)	301.5 (285.5,302)
July	27 (27,28)	0.2 (0,1.7)	18.5 (17.9,19.3)	63 (61,64)	291 (268.5,308.5)
August	27 (26,27)	3.1 (0.9,7.9)	16.1 (14.8,17.6)	65 (63,67)	291.5 (274.5,296.5)
September	27 (27,27)	15.6 (8.3,42.8)	16.3 (14.5,17.6)	65.5 (65,67)	278 (256.5,282)
October	26 (26,26)	53.2 (53.2,63.8)	9.7 (8.3,10.6)	68 (68,71)	268.3 (254.5,270)
November	24 (24,24)	32.6 (32.6,34.9)	6.1 (6.1,7)	72 (69,78)	205.5 (194,206.5)
December	23 (23,23)	14.3 (6,15.5)	7 (6.7,7.9)	71 (67,74)	242.3 (225.5,267)
3.4. Chennai (Tamil Nadu)					
January	25 (25,25)	7.7 (4.4,10.5)	15.4 (12.4,16.6)	73 (70,75)	267 (259.5,273.5)
February	25 (25,26)	1.5 (0.3,26)	13.2 (11.7,14)	72 (70,74)	275.5 (272.5,287)
March	28 (27,28)	1.8 (1.1,10.1)	14.8 (13.6,15.5)	72 (71,73)	301 (294,304)
April	30 (30,30)	7.2 (0.3,35.3)	18.9 (16.9,20.1)	72 (71,74)	344.5 (330,352.5)
May	32 (32,32)	18.6 (8.3,37.5)	18.9 (18.2,19.1)	66 (66,67)	381.5 (355,383)
June	31 (31,32)	37.8 (19,63.2)	17.4 (16.9,18.4)	62 (61,66)	336.5 (327.5,348.5)
July	30.5 (30,31)	37.9 (22.1,61.8)	16.8 (15.7,17.5)	65 (63,65.5)	343.5 (339,354.3)
August	30 (29,30)	75.4 (61.7,88.7)	15.2 (14.1,16)	69 (66.5,70.5)	339.3 (335.8,359.8)
September	29.5 (29,30)	101.1 (91.5,105.6)	13.3 (13,13.8)	72 (70,72)	333.5 (317,347.8)
October	29 (28,29)	102.6 (81.5,154.2)	12.2 (10.9,13.8)	73 (71,74)	252 (247,263.5)
November	27 (27,27)	60 (48.4,344.4)	17.3 (15.3,17.7)	74 (72,79)	201 (190.5,213)
December	26 (25,26)	84.8 (58.6,111.3)	18 (16.5,20)	76 (72,77)	199 (188.5,208)
3.5. Trivandrum (Kerala)					
January	26 (26,26)	3.2 (1.5,7.7)	8.7 (8.2,9.5)	70 (68,71)	299.5 (296.5,301)
February	26 (26,26)	6.1 (1.4,23)	8.8 (8.7,10.7)	71 (65,72)	278.5 (269.5,284)
March	28 (27,28)	37.8 (14.8,53.6)	9.8 (9.1,10.7)	71 (70,75)	293.5 (288,299.5)
April	28 (28,29)	57.3 (32.2,115.9)	12.2 (10.7,14.3)	79 (72,79)	278 (264,285)
May	28 (28,29)	24.9 (17.4,42.5)	15 (14.6,18)	79 (72,79)	252 (245.5,267.5)
June	27 (27,28)	82.2 (46.9,110.2)	20.1 (17.4,21.5)	78 (74,79)	243 (213,256)
July	26.5 (26,27.5)	65.6 (15.1,101.1)	21.1 (18.7,22.1)	78 (75,80)	266.3 (248.3,278.8)
August	26 (26,27.5)	33.6 (7.8,72.9)	18.8 (17,20.5)	80 (75.5,81)	264.5 (240.8,285.5)
September	26.5 (26,27)	61.7 (40.3,77.2)	17 (13.8,17.4)	80 (77,81.5)	256 (244,263.3)
October	27 (26,27)	117.9 (32.3,220.7)	11.4 (10.1,14.9)	78 (75,80)	266 (245.5,286)

For peer review only

Region/ Place (State) and months	Temperature (°C)	Rainfall (mm)	Wind speed (Kmph)	Humidity (%)	Sunshine (hour)
November	27 (27,27)	107.7 (47,140)	8.6 (8,9.1)	74 (72,76)	189 (174,205)
December	26 (26,27)	25.1 (15.7,37)	8.9 (8.6,9.6)	72 (72,75)	272.5 (267,280.5)
4. West region					
4.1. Mumbai (Maharashtra)					
January	24 (24,25)	0 (0,0.4)	10 (9.7,10.9)	52 (51,53)	310 (310,310)
February	25 (25,26)	0.4 (0,2.9)	10 (9.2,10.2)	54 (51,55)	280 (275.5,281)
March	28 (27,28)	0.6 (0,1.3)	10.5 (9.6,10.7)	53 (51,56)	308.5 (305.5,310)
April	29 (29,29)	0.1 (0,0.2)	9.7 (9.4,10.3)	63 (61,66)	300 (292.5,300)
May	30 (29,30)	1.3 (0.6,8)	10.5 (10.1,10.7)	72 (68,73)	305.5 (292,307)
June	29 (29,29)	292.8 (156.7,370)	16 (14.6,16.7)	78 (75,78)	217 (190,252)
July	27 (27,28)	450.2 (391.4,540.5)	17.3 (16,18.7)	83.5 (82,85.5)	164.8 (147.5,193.3)
August	27 (27,27)	282.7 (213.5,376.4)	14.1 (12.7,15.5)	85.5 (84,86.5)	190.8 (186.5,212.8)
September	27 (27,27)	136.2 (103.3,196.1)	9.8 (9.5,11.7)	82 (80,83)	229.8 (225.5,245)
October	28 (27,29)	29.9 (25.5,48.4)	8.1 (7.7,8.8)	67 (67,72)	299.5 (286.5,304)
November	27 (27,28)	2.3 (0,9.9)	9 (8.6,9)	55 (53,58)	257.5 (251.5,258.5)
December	26 (25,26)	0.2 (0,3.4)	9.8 (9,10)	51 (49,55)	295 (293.5,297.5)
4.2. Jamnagar (Gujarat)					
January	22 (21,23)	0 (0,0.1)	15.8 (14.4,17)	44 (41,49)	310 (307,310)
February	24 (23,24)	0 (0,0.6)	16 (14.5,16.8)	45 (44,50)	278.5 (274.5,281)
March	27 (27,28)	0.1 (0,0.2)	16.5 (15.9,17.1)	47 (44,53)	308.5 (306,308.5)
April	29 (29,30)	0 (0,0.4)	20.8 (18.3,21.3)	56 (54,56)	300 (297,300)
May	31 (30,31)	0.1 (0.1,0.2)	24.8 (23.9,25.3)	65 (63,67)	355 (353.5,357.5)
June	31 (31,32)	35.8 (5,93.6)	23.9 (21.7,27.9)	68 (67,69)	311.5 (296.5,321)
July	29 (29,29)	119.4 (88.5,141.7)	25.4 (23.8,27.9)	77.5 (75,78)	167.8 (143.5,197.5)
August	28 (28,28.5)	61.6 (39.7,107.3)	23.2 (22.1,24.6)	79 (77,80)	156.5 (143.5,182)
September	28 (28,29)	70.6 (24.3,113.3)	18.2 (17.8,19.7)	74 (73.5,77)	244.8 (225.3,259)
October	30 (29,31)	0.8 (0.2,12.4)	14 (12.5,14.1)	51 (48,62)	310 (308,310)
November	28 (27,29)	0.1 (0,1.9)	13.9 (13,14.6)	43 (40,50)	230 (228.5,231)
December	24 (24,25)	0 (0,0)	16.2 (14.5,17.1)	43 (39,46)	252.5 (250,252.5)
4.3. Jaipur (Rajasthan)					
January	14 (14,15)	1.9 (0.3,7.9)	8.3 (8.2,8.5)	47 (42,50)	253.5 (245.5,255)
February	18 (18,20)	1 (0.3,3.5)	10.2 (8.9,10.9)	34 (31,44)	277.5 (275.5,288.5)
March	25 (24,25)	0.8 (0,4.1)	10.2 (10,11)	24 (24,29)	304 (301.5,308.5)
April	31 (31,32)	3 (0.8,4.6)	11.2 (10.5,11.7)	19 (16,20)	323 (315.5,327.5)
May	36 (36,36)	5.5 (0.4,6.9)	14.3 (13.1,16.5)	19 (15,21)	383.5 (381.5,385)
June	36 (36,37)	9.9 (3.5,20.5)	13.9 (12.4,17.4)	30 (28,34)	363 (359.5,367.5)
July	32 (32,33.5)	48.4 (33.6,69)	12.8 (10.6,13.9)	55.5 (51,56.5)	351.8 (338.8,369.5)
August	29.5 (29,31)	74.1 (59.6,138.3)	11.1 (10.5,12.7)	66.5 (58.5,73.5)	287.5 (278,302.5)
September	29.5 (28.5,30)	19.4 (14.2,31)	10.4 (9.4,11.5)	53.5 (46.5,62.5)	281.8 (274.8,290.5)
October	28 (27,28)	2.3 (0,4.7)	8.4 (7.4,9.4)	31 (27,41)	263.5 (262.5,265)
November	22 (21,23)	0 (0,0.8)	7.2 (6.7,8)	29 (26,34)	225 (223.5,225)
December	16 (16,17)	0.4 (0,0.7)	7.6 (6.3,8.4)	37 (33,39)	232.5 (229.5,232.5)

Note: IQR- Interquartile range

For peer review only - http://bmjopen.bmj.com/site/about/guidelines.xhtml

Supplementary Figure 2: The pattern of monthly meteorological parameters across the regions

SF2a: Monthly median temperature for different regions in India (^{0}C)

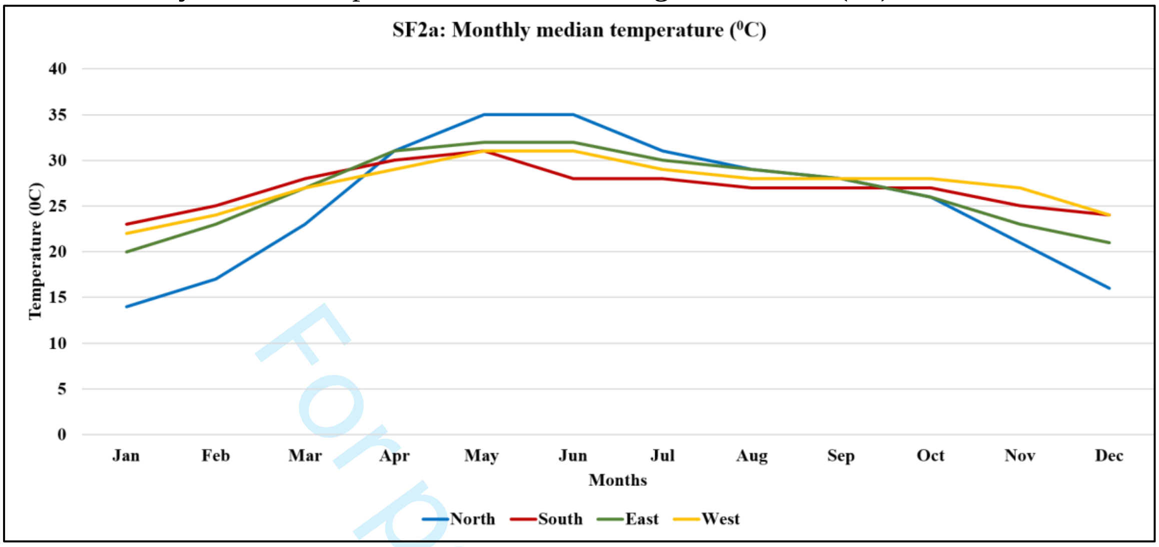

SF2b: Monthly median rainfall (mm) for different regions in India

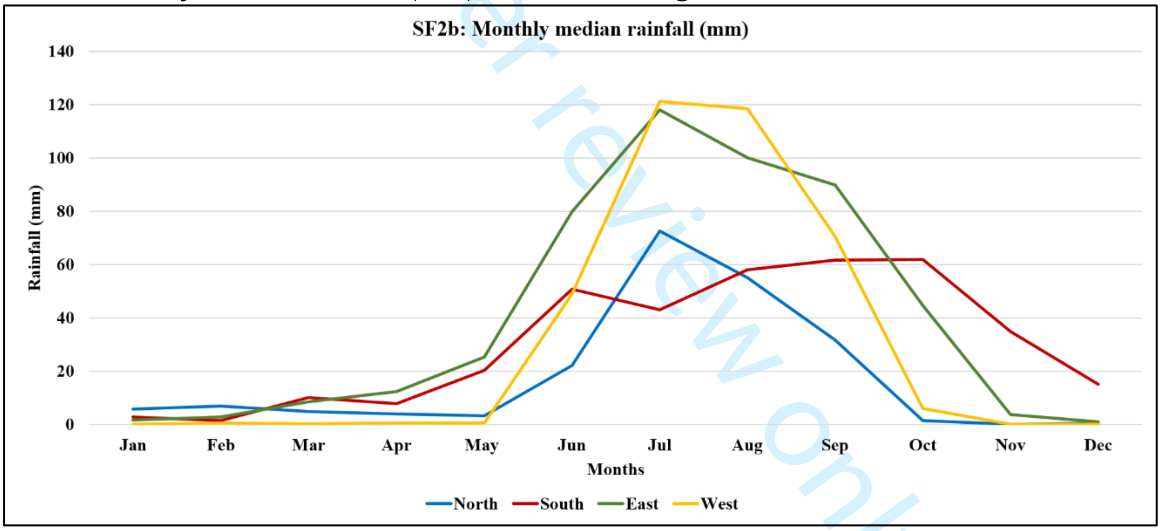

For peer review only

SF2c: Monthly median wind speed (Kmph) for different regions in India

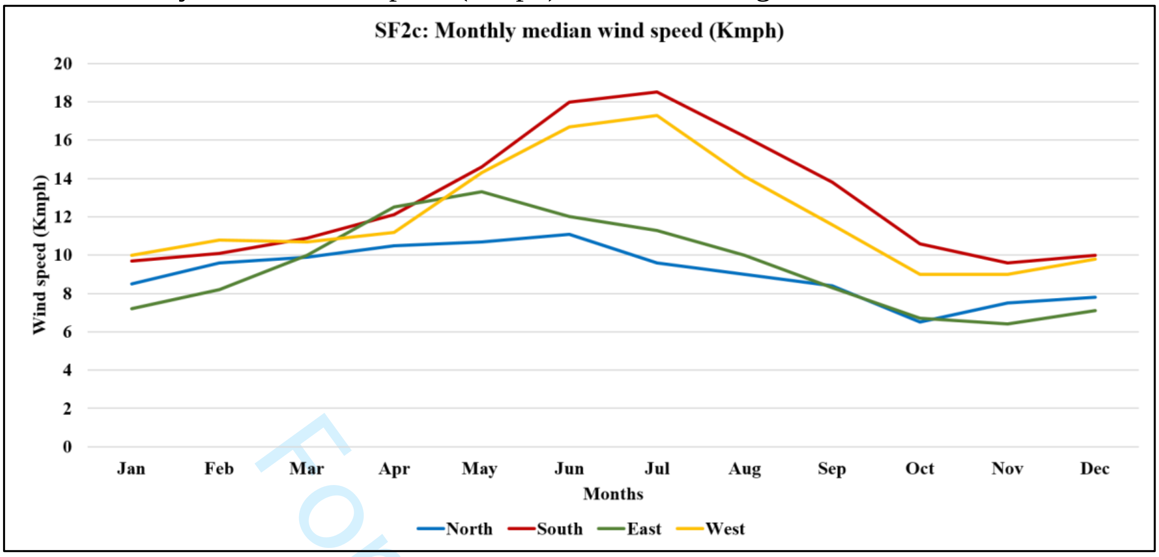

SF2d: Monthly median humidity (%) for different regions in India

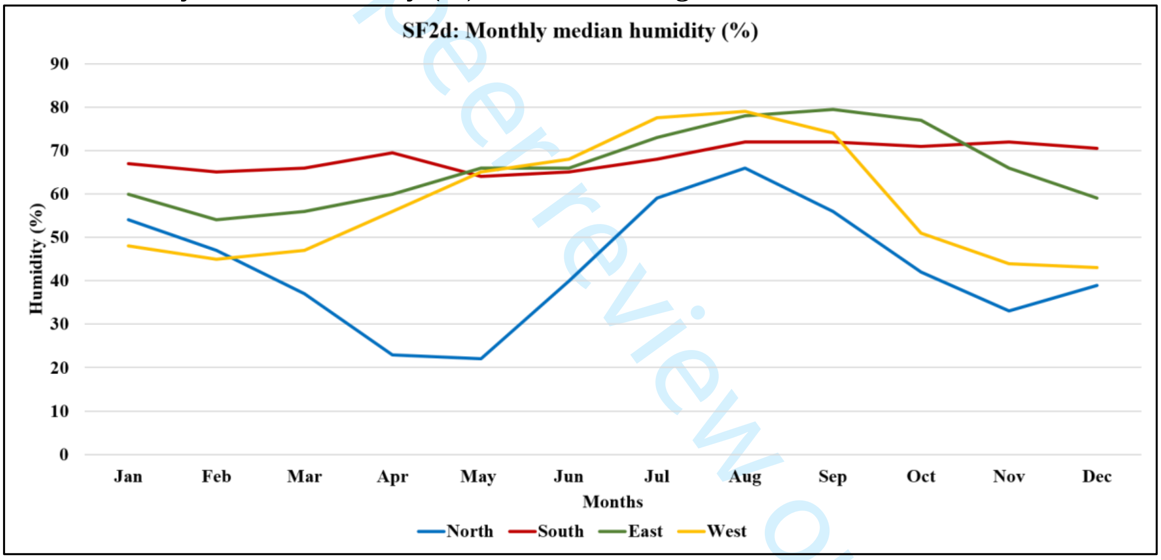

SF2e: Monthly median sunshine (hours) for different regions in India

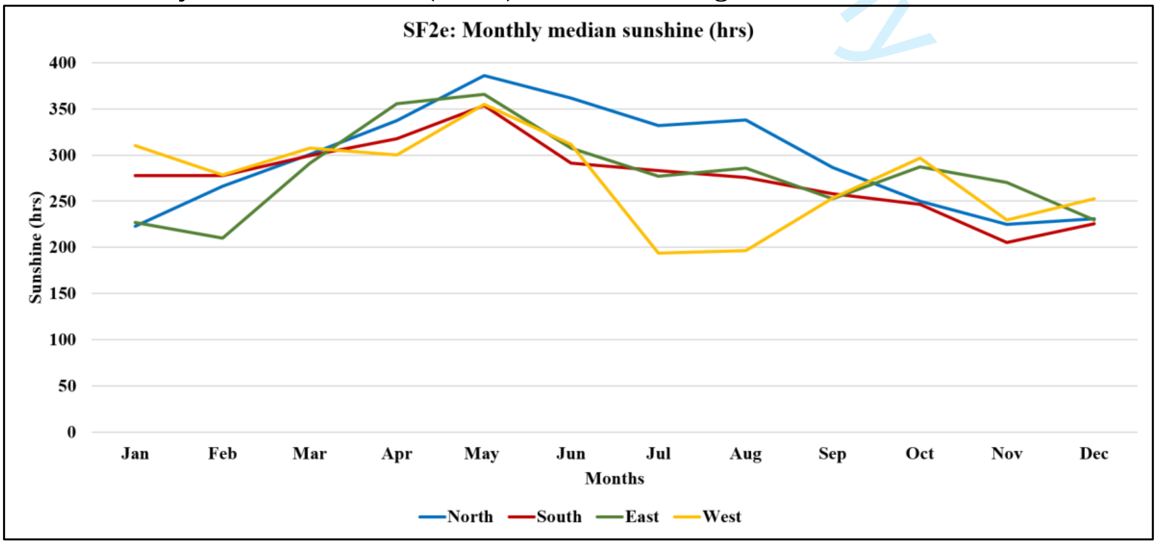

For peer review only - http://bmjopen.bmj.com/site/about/guidelines.xhtml

Supplementary Figure 3: The pooled monthly (SF3a) and seasonal (SF3b) distribution of intussusception in children during July 2010 to September 2017

SF3a: The monthly trend of intussusception cases

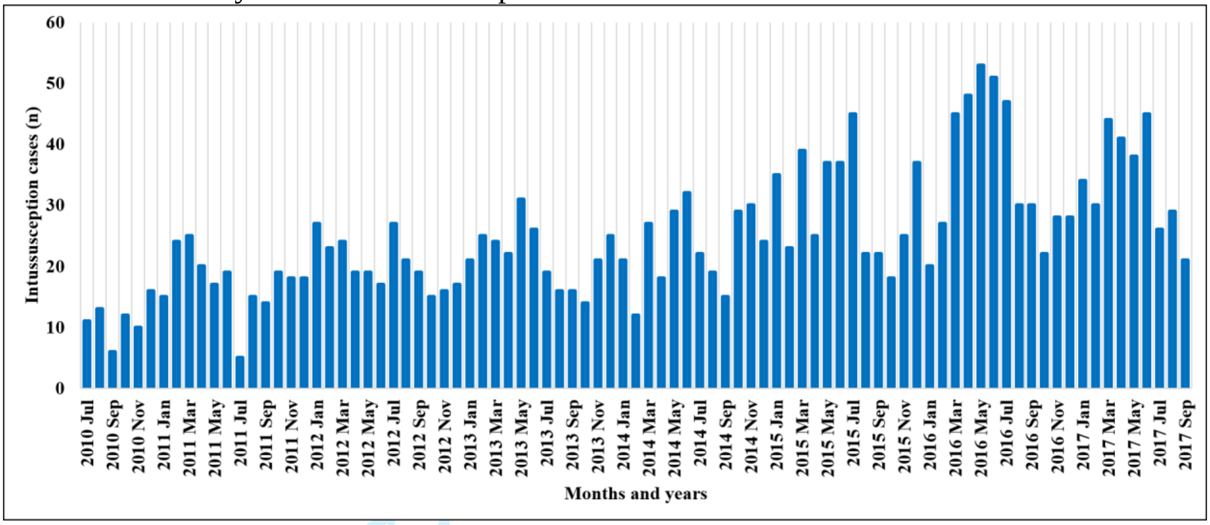

SF3b: The seasonal trend of intussusception cases

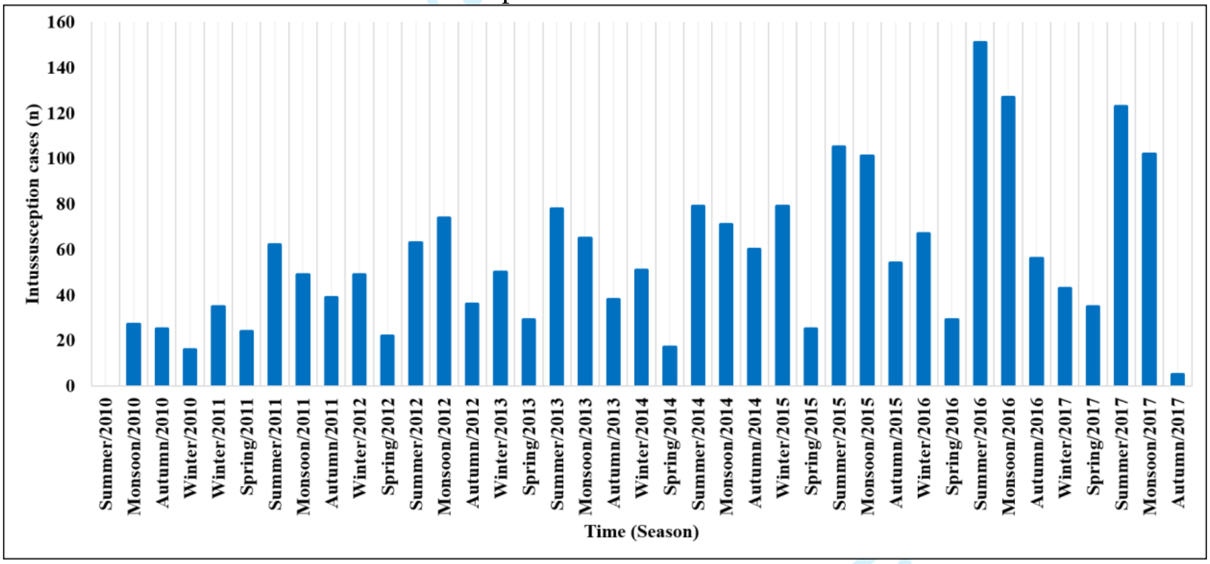

For peer review only - http://bmjopen.bmj.com/site/about/guidelines.xhtml

Supplementary Table 5. Pearson correlation analysis between monthly intussusception cases and monthly median meteorological parameters for the study sites

Regions/Study sites (n, number of cases)	Pearson correlation coefficient (r) for meteorological parameters				
	Temperature (°C)	Rainfall (mm)	Wind Speed (kmph)	Humidity (%)	Sunshine (hour)
North region					
Srinagar (n=145)	.253*	.164	.183	.012	.239*
Delhi and Gurgaon (n=96)	.107	-.045	.183*	-.173*	.134
Lucknow (110)	.091	.003	.285**	-.106	.192
Indore (n=19)	.101	-.211*	.290**	-.309**	.188
East region					
Bhubaneswar/Cuttack (n=335)	.047	-.056	.125	-.167*	.035
Berhampur (n=30)	.050	.047	.035	.044	.036
Kolkata (n=80)	.220*	-.052	.291**	-.144	.253*
Patna (n=19)	-.124	-.189	-.038	-.216*	-.040
Guwahati (n=112)	.220*	.001	.317**	-.119	.194
Agartala (n=17)	.082	.080	.139	-.017	.016
South region					
Vishakhapatnam (n=77)	.088	.053	-.002	.022	.029
Hyderabad (n=2)	-.030	-.061	-.131	.022	-.096
Coimbatore (n=136)	-.015	-.075	-.155	-.028	.017
Chennai (n=92)	.052	-.017	.007	.015	-.016
Trivandrum (n=774)	.085	-.286**	-.011	-.323**	.229*
West region					
Mumbai (n=34)	-.066	-.081	-.027	-.103	.035
Jamnagar (n=54)	.110	-.017	.011	.026	.105
Jaipur (n=29)	.187	-.078	.148	.001	.122

Note: Pearson correlation coefficient (r); Significance two tailed
** Significance level- p value <0.05; ** Significance level- p value <0.01*

For peer review only

For peer review only - http://bmjopen.bmj.com/site/about/guidelines.xhtml

Supplementary Table 6. Spearman's Rank correlation analysis between monthly intussusception cases and monthly median meteorological parameters for the study sites

Regions/Study sites (n, number of cases)	Pearson correlation coefficient (r) for meteorological parameters				
	Temperature (°C)	Rainfall (mm)	Wind Speed (kmph)	Humidity (%)	Sunshine (hour)
North region					
Srinagar (n=145)	.208	.122	.146	.032	.169
Delhi and Gurgaon (n=96)	.067	-.101	.136	-.184*	.065
Lucknow (110)	.166	.064	.331**	-.101	.225*
Indore (n=19)	.095	-.148	.286**	-.301**	.138
East region					
Bhubaneswar/Cuttack (n=335)	.036	-.068	.117	-.143	-.009
Berhampur (n=30)	.058	.037	.044	.035	.021
Kolkata (n=80)	.097	-.084	.170	-.236*	.125
Patna (n=19)	-.146	-.264*	-.085	-.231*	-.040
Guwahati (n=112)	.240*	-.014	.290**	-.118	.165
Agartala (n=17)	.107	.107	.154	.021	.044
South region					
Vishakhapatnam (n=77)	.106	.081	.048	.017	.060
Hyderabad (n=2)	-.012	-.052	-.138	.040	-.043
Coimbatore (n=136)	.002	-.123	-.147	-.091	.070
Chennai (n=92)	.055	.041	.025	.055	-.027
Trivandrum (n=774)	.134	-.287**	-.026	-.353**	.301**
West region					
Mumbai (n=34)	-.025	-.140	-.092	-.120	.046
Jamnagar (n=54)	.056	.071	.019	-.009	.106
Jaipur (n=29)	.131	-.082	.069	-.075	.079

Note: Pearson correlation coefficient (r); Significance two tailed
** Significance level- p value <0.05; ** Significance level- p value <0.01*

For peer review only - http://bmjopen.bmj.com/site/about/guidelines.xhtml

Supplementary Table 7. Regression analysis of the association between the intussusception cases and meteorological parameters for the study sites.

Study sites/ Variables	Regression coefficient, β (95% CI)	Standard error, S_β	χ2 - value	p value
North region				
Srinagar (n=145)[#]				
Temperature (^{0}C)	0.043(0.003,0.082)	0.020	4.479	**0.034**
Rainfall (mm)	0.007(-0.004,0.018)	0.005	1.616	0.204
Wind Speed (Kmph)	0.150(-0.048,0.347)	0.100	2.207	0.137
Humidity (%)	0.001(-0.018,0.020)	0.009	0.010	0.921
Sunshine (hour)	0.005(0.000,0.010)	0.002	3.930	**0.047**
Delhi and Gurgaon (n=96)[*]				
Temperature (^{0}C)	0.025(-0.005,0.055)	0.015	2.712	0.100
Rainfall (mm)	-0.003(-0.012,0.006)	0.004	0.476	0.490
Wind Speed (Kmph)	0.229(0.068,0.390)	0.082	7.805	**0.005**
Humidity (%)	-0.021(-0.036,-0.005)	0.007	7.041	**0.008**
Sunshine (hour)	0.004(0.000,0.008)	0.002	4.205	**0.040**
Lucknow (n=110)[*]				
Temperature (^{0}C)	0.013(-0.014,0.041)	0.014	0.873	0.350
Rainfall (mm)	0.000(-0.008,0.008)	0.004	0.001	0.972
Wind Speed (Kmph)	0.224(0.074,0.374)	0.077	8.580	**0.003**
Humidity (%)	-0.008(-0.022,0.006)	0.007	1.195	0.274
Sunshine (hour)	0.004(0.000,0.007)	0.002	3.915	**0.048**
Indore (n=19)[*]				
Temperature (^{0}C)	0.036(-0.033,0.105)	0.035	1.055	0.304
Rainfall (mm)	-0.039(-0.078,0.001)	0.020	3.723	0.054
Wind Speed (Kmph)	0.623(0.193,1.054)	0.219	8.062	**0.005**
Humidity (%)	-0.059(-0.097,-0.021)	0.019	9.110	**0.003**
Sunshine (hour)	0.009(0.000,0.017)	0.004	3.556	0.059
East region				
Bhubaneswar/Cuttack (n=335)[#]				
Temperature (^{0}C)	0.012(-0.032,0.057)	0.023	.286	0.593
Rainfall (mm)	-0.001(-0.006,0.003)	0.002	.429	0.513
Wind Speed (Kmph)	0.060(-0.021,0.141)	0.041	2.131	0.144
Humidity (%)	-0.022(-0.044,0.000)	0.011	3.953	**0.047**
Sunshine (hour)	0.001(-0.003,0.005)	0.002	.157	0.692
Berhampur (n=30)[*]				
Temperature (^{0}C)	0.023(-0.068,0.114)	0.046	0.239	0.625
Rainfall (mm)	0.002(-0.006,0.01)	0.004	0.211	0.646
Wind Speed (Kmph)	0.027(-0.128,0.182)	0.079	0.118	0.731
Humidity (%)	0.009(-0.032,0.051)	0.021	0.186	0.666
Sunshine (hour)	0.001(-0.006,0.009)	0.004	0.125	0.724
Kolkata (n=80)[*]				
Temperature (^{0}C)	0.113(0.050,0.176)	0.032	12.448	**0.000**
Rainfall (mm)	-0.002(-0.007,0.003)	0.002	0.710	0.400
Wind Speed (Kmph)	0.233(0.134,0.332)	0.050	21.269	**0.000**
Humidity (%)	-0.032(-0.059,-0.005)	0.013	5.433	**0.020**
Sunshine (hour)	0.010(0.005,0.014)	0.002	16.577	**0.000**

For peer review only - http://bmjopen.bmj.com/site/about/guidelines.xhtml

Patna (n=19)*				
Temperature (⁰C)	-0.067(-0.177,0.042)	0.056	1.452	0.228
Rainfall (mm)	-0.012(-0.025,0.001)	0.006	3.157	0.076
Wind Speed (Kmph)	-0.037(-0.235,0.161)	0.101	0.137	0.711
Humidity (%)	-0.063(-0.124,-0.003)	0.031	4.208	**0.040**
Sunshine (hour)	-0.002(-0.012,0.008)	0.005	0.150	0.698
Guwahati (n=112)*				
Temperature (⁰C)	0.050(0.002,0.098)	0.025	4.097	**0.043**
Rainfall (mm)	0.000(-0.004,0.004)	0.002	0.000	0.995
Wind Speed (Kmph)	0.118(0.038,0.199)	0.041	8.408	**0.004**
Humidity (%)	-0.012(-0.034,0.01)	0.011	1.212	0.271
Sunshine (hour)	0.004(0.000,0.008)	0.002	3.207	0.073
Agartala (n=17)*				
Temperature (⁰C)	0.060(-0.066,0.186)	0.064	0.874	0.350
Rainfall (mm)	0.005(-0.006,0.016)	0.005	0.838	0.360
Wind Speed (Kmph)	0.166(-0.042,0.374)	0.106	2.435	0.119
Humidity (%)	-0.005(-0.061,0.050)	0.028	0.037	0.848
Sunshine (hour)	0.001(-0.009,0.011)	0.005	0.033	0.857
South region				
Vishakhapatnam (n=77)*				
Temperature (⁰C)	0.043(-0.058,0.144)	0.051	0.702	0.402
Rainfall (mm)	0.003(-0.007,0.012)	0.005	0.255	0.613
Wind Speed (Kmph)	-0.001(-0.071,0.070)	0.036	0.001	0.981
Humidity (%)	0.008(-0.069,0.085)	0.039	0.042	0.837
Sunshine (hour)	0.001(-0.005,0.007)	0.003	0.078	0.780
Hyderabad (n=2)*				
Temperature (⁰C)	-0.088(-0.717,0.541)	0.321	0.075	0.784
Rainfall (mm)	-0.018(-0.085,0.048)	0.034	0.298	0.585
Wind Speed (Kmph)	-0.650(-2.074,0.774)	0.726	0.801	0.371
Humidity (%)	0.051(-0.436,0.539)	0.249	0.043	0.837
Sunshine (hour)	-0.017(-0.056,0.022)	0.020	0.764	0.382
Coimbatore (n=136)*				
Temperature (⁰C)	-0.007(-0.082,0.068)	0.038	0.033	0.857
Rainfall (mm)	-0.003(-0.011,0.004)	0.004	0.825	0.364
Wind Speed (Kmph)	-0.053(-0.108,0.002)	0.028	3.506	0.061
Humidity (%)	-0.010(-0.067,0.047)	0.029	0.119	0.730
Sunshine (hour)	0.000(-0.004,0.005)	0.002	0.043	0.836
Chennai (n=92)*				
Temperature (⁰C)	0.027(-0.065,0.120)	0.047	0.337	0.562
Rainfall (mm)	-0.001(-0.010,0.008)	0.005	0.036	0.850
Wind Speed (Kmph)	0.003(-0.062,0.067)	0.033	0.006	0.938
Humidity (%)	0.006(-0.064,0.076)	0.036	0.028	0.868
Sunshine (hour)	0.000(-0.006,0.005)	0.003	0.030	0.863
Trivandrum (n=774)*				
Temperature (⁰C)	0.015(-0.016,0.047)	0.016	0.895	0.344
Rainfall (mm)	-0.005(-0.008,-0.002)	0.002	10.159	**0.001**
Wind Speed (Kmph)	-0.001(-0.024,0.021)	0.011	0.016	.898
Humidity (%)	-0.044(-0.068,-0.020)	0.012	12.938	**0.000**

For peer review only - http://bmjopen.bmj.com/site/about/guidelines.xhtml

Sunshine (hour)	0.002(0.001,0.004)	0.001	6.538	**0.011**
West region				
Mumbai (n=34)*				
Temperature (^{0}C)	-0.040(-0.165,0.085)	0.064	0.387	0.534
Rainfall (mm)	-0.003(-0.011,0.005)	0.004	0.580	0.446
Wind Speed (Kmph)	-0.016(-0.140,0.108)	0.063	0.065	0.799
Humidity (%)	-0.013(-0.039,0.013)	0.013	0.929	0.335
Sunshine (hour)	0.001(-0.006,0.008)	0.004	0.108	0.742
Jamnagar (n=54)*				
Temperature (^{0}C)	0.066(-0.042,0.173)	0.055	1.440	0.230
Rainfall (mm)	-0.001(-0.006,0.005)	0.003	0.035	0.852
Wind Speed (Kmph)	0.006(-0.090,0.102)	0.049	0.015	0.903
Humidity (%)	0.003(-0.017,0.023)	0.010	0.080	0.777
Sunshine (hour)	0.003(-0.002,0.009)	0.003	1.305	0.253
Jaipur (n=29)*				
Temperature (^{0}C)	0.150(-0.009,0.309)	0.081	3.425	0.064
Rainfall (mm)	-0.003(-0.012,0.005)	0.004	0.600	0.439
Wind Speed (Kmph)	0.092(-0.031,0.216)	0.063	2.141	0.143
Humidity (%)	0.000(-0.027,0.027)	0.014	0.000	0.993
Sunshine (hour)	0.005(-0.003,0.013)	0.004	1.471	0.225

*Note: Tests used for regression analysis: * Poisson Regression; # Negative Binomial Regression*

For peer review only

For peer review only - http://bmjopen.bmj.com/site/about/guidelines.xhtml

Supplementary Table 8. Intussusception cases in children at different temperature ranges at pooled and regional level

Pooled/ Region	Temperature range (°C)	Years of data collection								Total
		2010	2011	2012	2013	2014	2015	2016	2017	
Pooled	<20°C	8	14	24	29	26	39	55	51	246
	21°C -25°C	2	31	44	32	43	54	42	31	279
	26°C -30°C	56	149	153	166	164	208	245	164	1305
	>30°C	2	15	23	33	45	63	88	62	331
	Total	68	209	244	260	278	364	430	308	2161
North Region	<20°C	5	8	18	28	20	27	45	46	197
	21°C -25°C	1	5	3	2	10	7	4	1	33
	26°C -30°C	1	2	4	3	4	6	13	3	36
	>30°C	2	4	12	11	14	21	29	11	104
	Total	9	19	37	44	48	61	91	61	370
South Region	<20°C	0	0	0	0	0	0	0	0	0
	21°C -25°C	1	14	15	10	7	11	11	8	77
	26°C -30°C	49	117	113	132	116	157	167	116	967
	>30°C	0	3	2	7	1	6	6	12	37
	Total	50	134	130	149	124	174	184	136	1081
East Region	<20°C	3	6	6	1	5	10	9	4	44
	21°C -25°C	0	10	21	17	22	36	23	21	150
	26°C -30°C	6	24	26	23	37	36	53	35	240
	>30°C	0	5	8	13	20	30	46	37	159
	Total	9	45	61	54	84	112	131	97	593
West Region	<20°C	0	0	0	0	1	2	1	1	5
	21°C -25°C	0	2	5	3	4	0	4	1	19
	26°C -30°C	0	6	10	8	7	9	12	10	62
	>30°C	0	3	1	2	10	6	7	2	31
	Total	0	11	16	13	22	17	24	14	117

For peer review only

Supplementary Table 9. Variations in the intussusception cases between the different temperature categories by Kruskal-Wallis H test

Level	df	χ^2-statistic	P- value
Pooled	3	15.933	< 0.001
North Region	3	21.054	< 0.001
South Region	3	50.625	< 0.001
East Region	3	3.502	0.3205
West Region	3	5.606	0.1324

Notes: Pooled- all sites/regions combined; df: degrees of freedom; χ^2-statistic: critical chi square value

For peer review only

Supplementary Figure 4: Association of monthly intussusception cases with mean monthly temperatures

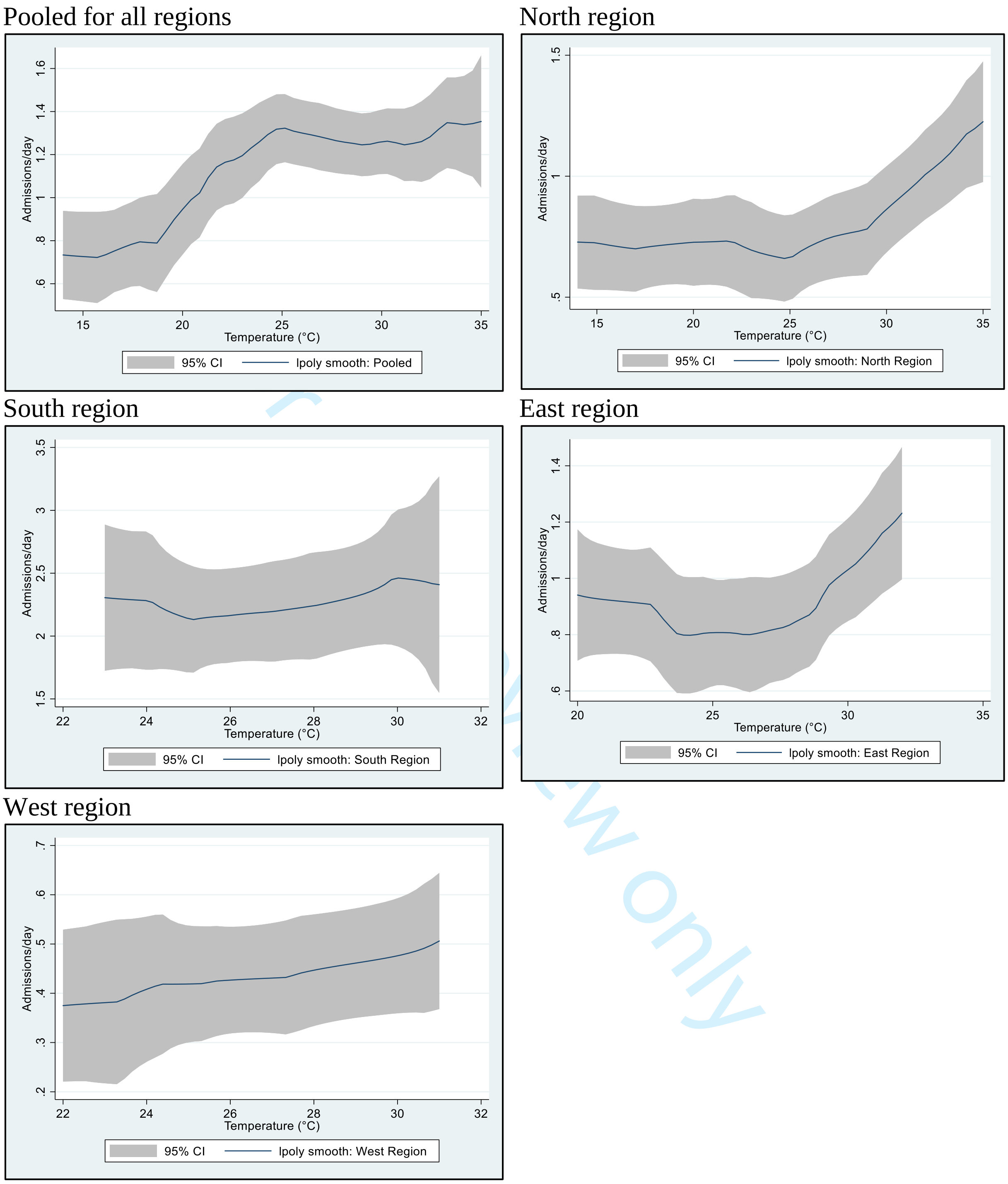

Supplementary Figure 5: Association of monthly intussusception cases with mean monthly humidity

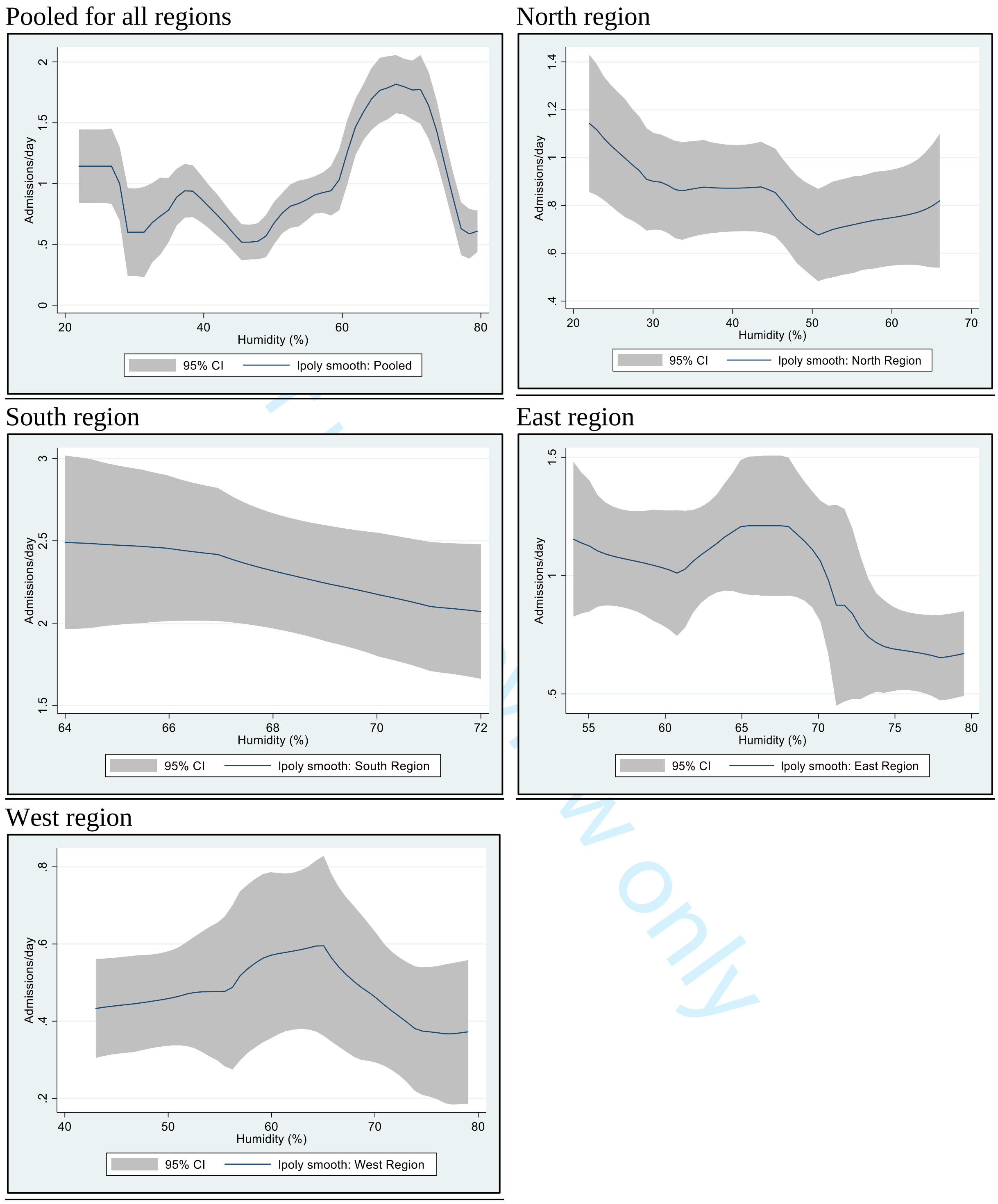

For peer review only - http://bmjopen.bmj.com/site/about/guidelines.xhtml

Supplementary Figure 6: Association of monthly intussusception cases with mean monthly wind speed

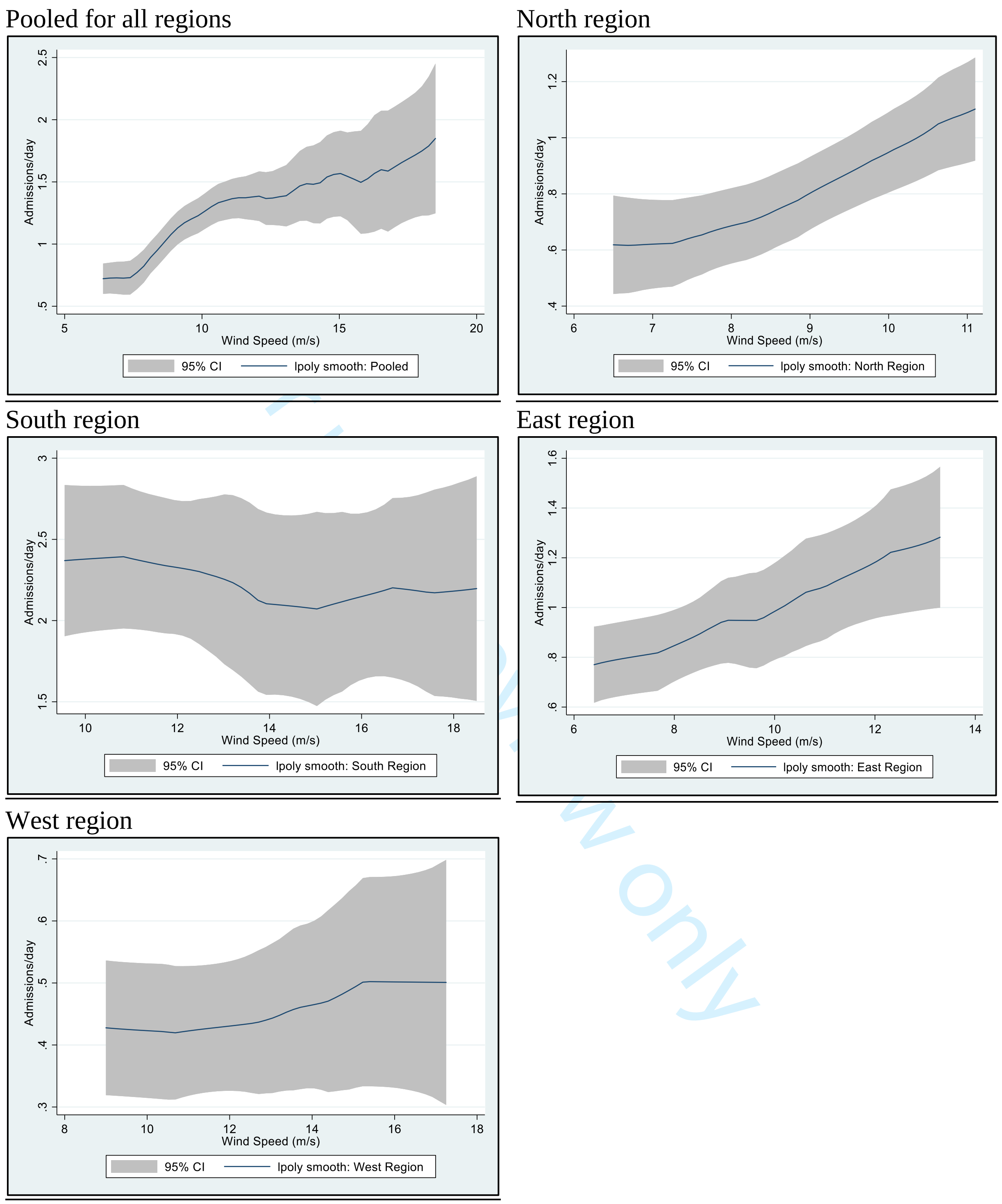

For peer review only - http://bmjopen.bmj.com/site/about/guidelines.xhtml

Supplementary Figure 7: Association of monthly intussusception cases with mean monthly sunshine

Pooled for all regions

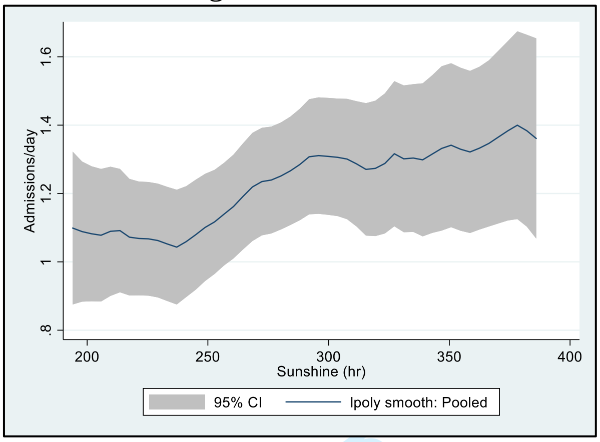

North region

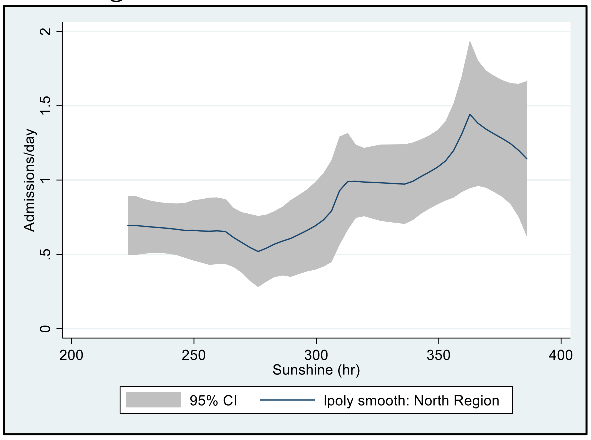

South region

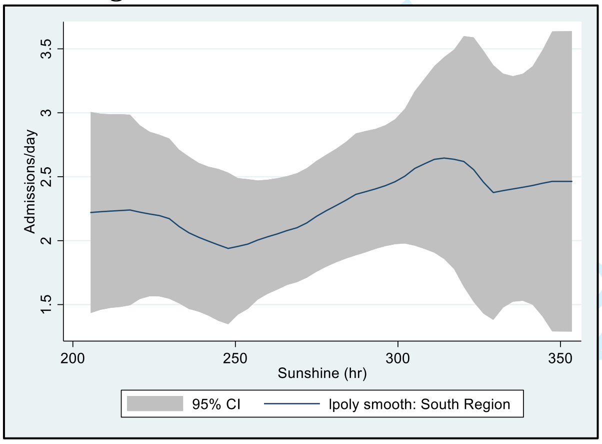

East region

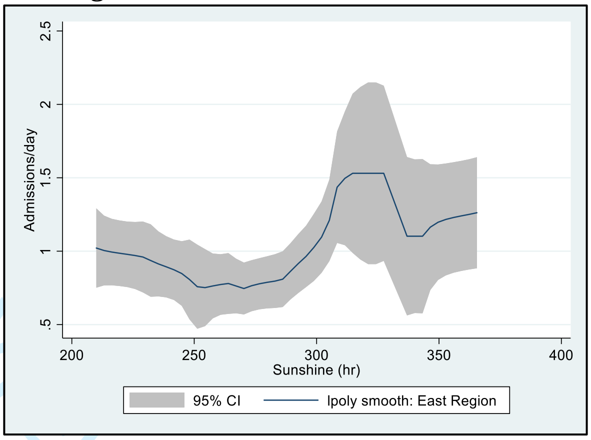

West region

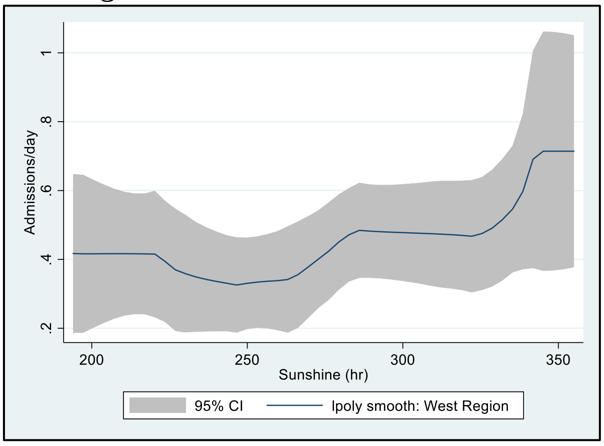

For peer review only

Supplementary Figure 8: Association of monthly intussusception cases with mean monthly rainfall

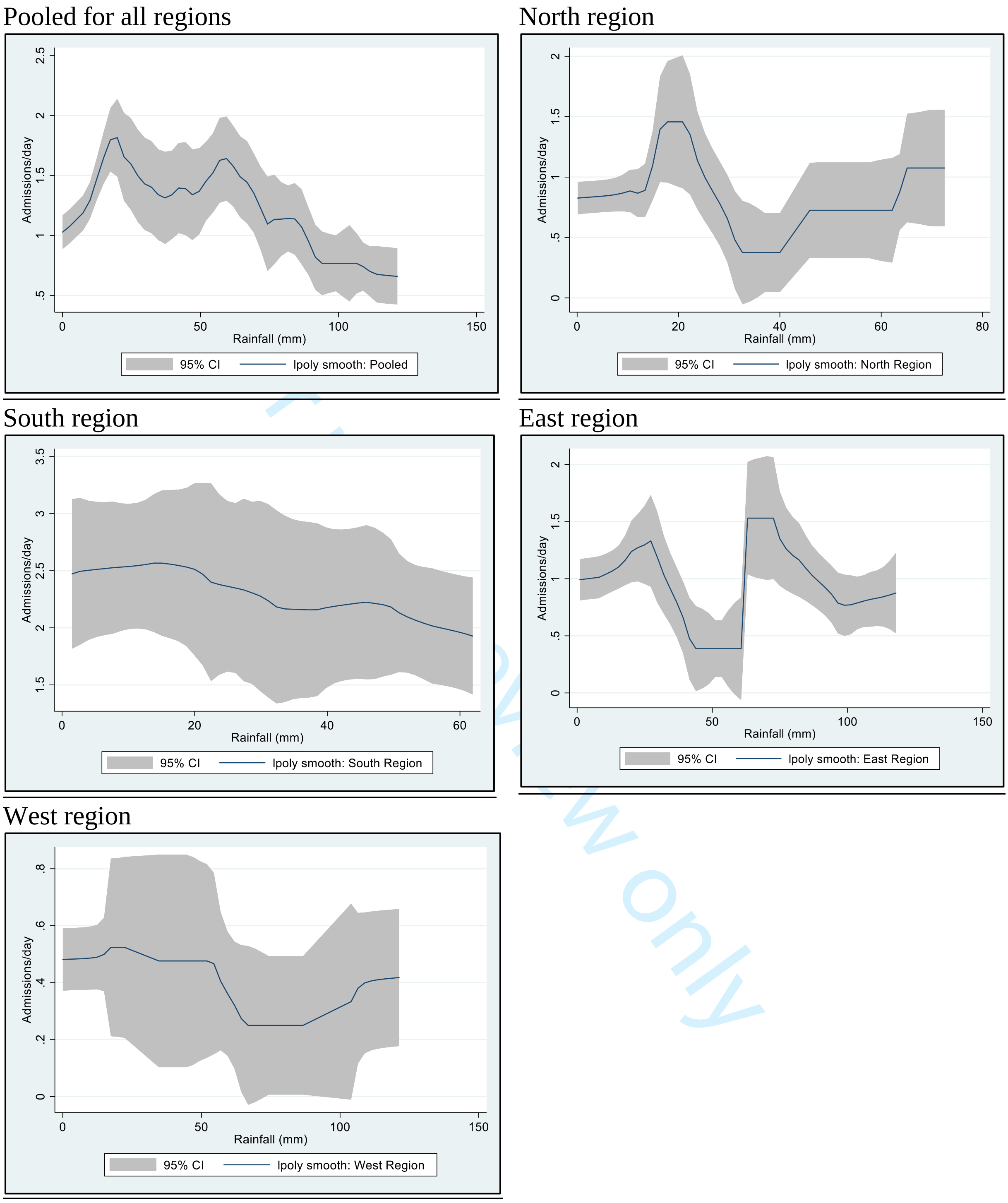

For peer review only

Supplementary Table 10. Factorial analysis-of-variance of intussusception in children with season and age groups

Source	Dependent variables	Type III Sum of squares	Degree of freedom	Mean square	F	p value
Corrected model	Seasonal IS cases	285.847[a]	38	7.522	1.442	**.040**
	Seasonal IS cases of 2-6 months	49.145[b]	38	1.293	1.579	**.014**
	Seasonal IS cases of 7-12 months	45.682[c]	38	1.202	1.012	.449
	Seasonal IS cases of 13-24 months	31.855[d]	38	.838	1.334	.085
Intercept	Seasonal IS cases	789.283	1	789.283	151.302	.000
	Seasonal IS cases of 2-6 months	101.336	1	101.336	123.760	.000
	Seasonal IS cases of 7-12 months	135.705	1	135.705	114.285	.000
	Seasonal IS cases of 13-24 months	40.684	1	40.684	64.740	.000
Season	Seasonal IS cases	8.674	4	2.169	.416	.797
	Seasonal IS cases of 2-6 months	1.930	4	.482	.589	.670
	Seasonal IS cases of 7-12 months	3.207	4	.802	.675	.609
	Seasonal IS cases of 13-24 months	.856	4	.214	.340	.851
Season year	Seasonal IS cases	82.483	7	11.783	2.259	**.027**
	Seasonal IS cases of 2-6 months	17.669	7	2.524	3.083	**.003**
	Seasonal IS cases of 7-12 months	3.504	7	.501	.422	.889
	Seasonal IS cases of 13-24 months	11.481	7	1.640	2.610	**.011**
Season* Season year	Seasonal IS cases	57.160	27	2.117	.406	.997
	Seasonal IS cases of 2-6 months	13.795	27	.511	.624	.934
	Seasonal IS cases of 7-12 months	19.521	27	.723	.609	.943
	Seasonal IS cases of 13-24 months	7.717	27	.286	.455	.993
Error	Seasonal IS cases	9092.547	1743	5.217		
	Seasonal IS cases of 2-6 months	1427.189	1743	.819		
	Seasonal IS cases of 7-12 months	2069.674	1743	1.187		
	Seasonal IS cases of 13-24 months	1095.327	1743	.628		
Total	Seasonal IS cases	11999.000	1782			
	Seasonal IS cases of 2-6 months	1816.000	1782			
	Seasonal IS cases of 7-12 months	2545.000	1782			
	Seasonal IS cases of 13-24 months	1272.000	1782			
Modified total	Seasonal IS cases	9378.393	1781			
	Seasonal IS cases of 2-6 months	1476.334	1781			
	Seasonal IS cases of 7-12 months	2115.356	1781			
	Seasonal IS cases of 13-24 months	1127.183	1781			

Note: a: R squared= .030 (adjusted R squared= .009); b: R squared= .033 (adjusted R squared= .012); c: R squared = .022 (adjusted R squared= .000); d: R squared= .028 (adjusted R squared= .007)

For peer review only

Supplementary Table 11. Factorial analysis-of-variance of intussusception in children with season and regions

Source	Dependent variables	Type III Sum of squares	Degree of freedom	Mean square	F	p value
Corrected model	Seasonal IS cases	285.847[a]	38	7.522	1.442	**.040**
	Seasonal IS cases of North region	36.237[b]	38	.954	1.809	**.002**
	Seasonal IS cases of South region	55.831[c]	38	1.469	.321	1.000
	Seasonal IS cases of East region	53.071[d]	38	1.397	1.333	.085
	Seasonal IS cases of West region	2.721[e]	38	.072	.662	.944
Intercept	Seasonal IS cases	789.283	1	789.283	151.302	**.000**
	Seasonal IS cases of North region	29.881	1	29.881	56.676	**.000**
	Seasonal IS cases of South region	180.247	1	180.247	39.373	**.000**
	Seasonal IS cases of East region	59.322	1	59.322	56.607	**.000**
	Seasonal IS cases of West region	2.250	1	2.250	20.798	**.000**
Season	Seasonal IS cases	8.674	4	2.169	.416	.797
	Seasonal IS cases of North region	10.274	4	2.569	4.872	**.001**
	Seasonal IS cases of South region	9.239	4	2.310	.505	.732
	Seasonal IS cases of East region	1.801	4	.450	.430	.787
	Seasonal IS cases of West region	.207	4	.052	.479	.752
Season year	Seasonal IS cases	82.483	7	11.783	2.259	**.027**
	Seasonal IS cases of North region	11.119	7	1.588	3.013	**.004**
	Seasonal IS cases of South region	5.618	7	.803	.175	.990
	Seasonal IS cases of East region	18.661	7	2.666	2.544	**.013**
	Seasonal IS cases of West region	.464	7	.066	.612	.746
Season* Season year	Seasonal IS cases	57.160	27	2.117	.406	.997
	Seasonal IS cases of North region	8.198	27	.304	.576	.960
	Seasonal IS cases of South region	28.377	27	1.051	.230	1.000
	Seasonal IS cases of East region	15.527	27	.575	.549	.971
	Seasonal IS cases of West region	1.325	27	.049	.454	.993
Error	Seasonal IS cases	9092.547	1743	5.217		
	Seasonal IS cases of North region	918.939	1743	.527		
	Seasonal IS cases of South region	7979.411	1743	4.578		
	Seasonal IS cases of East region	1826.595	1743	1.048		
	Seasonal IS cases of West region	188.597	1743	.108		
Total	Seasonal IS cases	11999.000	1782			
	Seasonal IS cases of North region	1032.000	1782			
	Seasonal IS cases of South region	8691.000	1782			
	Seasonal IS cases of East region	2077.000	1782			
	Seasonal IS cases of West region	199.000	1782			
Modified total	Seasonal IS cases	9378.393	1781			
	Seasonal IS cases of North region	955.176	1781			
	Seasonal IS cases of South region	8035.242	1781			
	Seasonal IS cases of East region	1879.666	1781			
	Seasonal IS cases of West region	191.318	1781			

Note: a: R squared = .030 (adjusted R squared= .009); b: R squared = .038 (adjusted R squared= .017); c: R squared = .007 (adjusted R squared= -.015); d: R squared= .028 (adjusted R squared= .007); e: R squared = .014 (adjusted R squared= -.007)

For peer review only - http://bmjopen.bmj.com/site/about/guidelines.xhtml

Supplementary Table 12. Multiple comparisons of seasonal intussusception in children in different seasons pooled for all sites

Dependent variable		Season (A)	Season (B)	Mean difference (A-B)	Standard error	p value	95% CI
Seasonal IS cases pooled	Tukey HSD	Monsoon	Spring	-.175	.2285	.940	-0.8 , 0.45
			Summer	-.409	.1643	.093	-0.86 , 0.04
			Monsoon	-.038	.1573	.999	-0.47 , 0.39
			Autumn	.160	.1758	.892	-0.32 , 0.64
		Spring	Winter	.175	.2285	.940	-0.45 , 0.8
			Summer	-.234	.2221	.831	-0.84 , 0.37
			Monsoon	.137	.2169	.970	-0.46 , 0.73
			Autumn	.336	.2307	.592	-0.29 , 0.97
		Summer	Winter	.409	.1643	.093	-0.04 , 0.86
			Spring	.234	.2221	.831	-0.37 , 0.84
			Monsoon	.371	.1478	.089	-0.03 , 0.77
			Autumn	.569*	.1673	**.006**	0.11 , 1.03
		Monsoon	Winter	.038	.1573	.999	-0.39 , 0.47
			Spring	-.137	.2169	.970	-0.73 , 0.46
			Summer	-.371	.1478	.089	-0.77 , 0.03
			Autumn	.199	.1604	.729	-0.24 , 0.64
		Autumn	Winter	-.160	.1758	.892	-0.64 , 0.32
			Spring	-.336	.2307	.592	-0.97 , 0.29
			Summer	-.569*	.1673	**.006**	-1.03 , -0.11
			Monsoon	-.199	.1604	.729	-0.64 , 0.24

*Note: * The difference is significant at $p<0.05$ level.*

For peer review only

Supplementary Table 13. Multiple comparisons of seasonal intussusception in children in different seasons at regional level

Dependent variable		Season (A)	Season (B)	Mean difference (A-B)	Standard error	p value	95% CI
North Region	Tukey HSD	Winter	Spring	-.151	.0726	.231	-0.35 , 0.05
			Summer	-.039	.0522	.943	-0.18 , 0.1
			Monsoon	.125	.0500	.089	-0.01 , 0.26
			Autumn	.139	.0559	.093	-0.01 , 0.29
		Spring	Winter	.151	.0726	.231	-0.05 , 0.35
			Summer	.111	.0706	.512	-0.08 , 0.3
			Monsoon	.276*	.0690	**.001**	0.09 , 0.46
			Autumn	.290*	.0733	**.001**	0.09 , 0.49
		Summer	Winter	.039	.0522	.943	-0.1 , 0.18
			Spring	-.111	.0706	.512	-0.3 , 0.08
			Monsoon	.165*	.0470	**.004**	0.04 , 0.29
			Autumn	.179*	.0532	**.007**	0.03 , 0.32
		Monsoon	Winter	-.125	.0500	.089	-0.26 , 0.01
			Spring	-.276*	.0690	**.001**	-0.46 , -0.09
			Summer	-.165*	.0470	**.004**	-0.29 , -0.04
			Autumn	.014	.0510	.999	-0.13 , 0.15
		Autumn	Winter	-.139	.0559	.093	-0.29 , 0.01
			Spring	-.290*	.0733	**.001**	-0.49 , -0.09
			Summer	-.179*	.0532	**.007**	-0.32 , -0.03
			Monsoon	-.014	.0510	.999	-0.15 , 0.13
South Region	Tukey HSD	Winter	Spring	-.014	.2140	1.000	-0.6 , 0.57
			Summer	-.197	.1539	.705	-0.62 , 0.22
			Monsoon	-.141	.1473	.875	-0.54 , 0.26
			Autumn	.032	.1647	1.000	-0.42 , 0.48
		Spring	Winter	.014	.2140	1.000	-0.57 , 0.6
			Summer	-.183	.2080	.905	-0.75 , 0.39
			Monsoon	-.127	.2032	.971	-0.68 , 0.43
			Autumn	.046	.2161	1.000	-0.54 , 0.64
		Summer	Winter	.197	.1539	.705	-0.22 , 0.62
			Spring	.183	.2080	.905	-0.39 , 0.75
			Monsoon	.056	.1384	.994	-0.32 , 0.43
			Autumn	.229	.1568	.588	-0.2 , 0.66
		Monsoon	Winter	.141	.1473	.875	-0.26 , 0.54
			Spring	.127	.2032	.971	-0.43 , 0.68
			Summer	-.056	.1384	.994	-0.43 , 0.32
			Autumn	.173	.1503	.779	-0.24 , 0.58
		Autumn	Winter	-.032	.1647	1.000	-0.48 , 0.42
			Spring	-.046	.2161	1.000	-0.64 , 0.54
			Summer	-.229	.1568	.588	-0.66 , 0.2
			Monsoon	-.173	.1503	.779	-0.58 , 0.24
East region	Tukey HSD	Winter	Spring	.035	.1024	.997	-0.24 , 0.31
			Summer	-.125	.0736	.433	-0.33 , 0.08
			Monsoon	-.001	.0705	1.000	-0.19 , 0.19

For peer review only - http://bmjopen.bmj.com/site/about/guidelines.xhtml

			Autumn	.010	.0788	1.000	-0.21 , 0.23
		Spring	Winter	-.035	.1024	.997	-0.31 , 0.24
			Summer	-.160	.0995	.490	-0.43 , 0.11
			Monsoon	-.036	.0972	.996	-0.3 , 0.23
			Autumn	-.025	.1034	.999	-0.31 , 0.26
		Summer	Winter	.125	.0736	.433	-0.08 , 0.33
			Spring	.160	.0995	.490	-0.11 , 0.43
			Monsoon	.124	.0662	.331	-0.06 , 0.31
			Autumn	.135	.0750	.372	-0.07 , 0.34
		Monsoon	Winter	.001	.0705	1.000	-0.19 , 0.19
			Spring	.036	.0972	.996	-0.23 , 0.3
			Summer	-.124	.0662	.331	-0.31 , 0.06
			Autumn	.011	.0719	1.000	-0.19 , 0.21
		Autumn	Winter	-.010	.0788	1.000	-0.23 , 0.21
			Spring	.025	.1034	.999	-0.26 , 0.31
			Summer	-.135	.0750	.372	-0.34 , 0.07
			Monsoon	-.011	.0719	1.000	-0.21 , 0.19
West region	Tukey HSD	Winter	Spring	-.046	.0329	.637	-0.14 , 0.04
			Summer	-.048	.0237	.260	-0.11 , 0.02
			Monsoon	-.022	.0227	.872	-0.08 , 0.04
			Autumn	-.021	.0253	.921	-0.09 , 0.05
		Spring	Winter	.046	.0329	.637	-0.04 , 0.14
			Summer	-.002	.0320	1.000	-0.09 , 0.09
			Monsoon	.024	.0312	.941	-0.06 , 0.11
			Autumn	.025	.0332	.947	-0.07 , 0.12
		Summer	Winter	.048	.0237	.260	-0.02 , 0.11
			Spring	.002	.0320	1.000	-0.09 , 0.09
			Monsoon	.026	.0213	.743	-0.03 , 0.08
			Autumn	.027	.0241	.805	-0.04 , 0.09
		Monsoon	Winter	.022	.0227	.872	-0.04 , 0.08
			Spring	-.024	.0312	.941	-0.11 , 0.06
			Summer	-.026	.0213	.743	-0.08 , 0.03
			Autumn	.001	.0231	1.000	-0.06 , 0.06
		Autumn	Winter	.021	.0253	.921	-0.05 , 0.09
			Spring	-.025	.0332	.947	-0.12 , 0.07
			Summer	-.027	.0241	.805	-0.09 , 0.04
			Monsoon	-.001	.0231	1.000	-0.06 , 0.06

*Note: * The difference is significant at p<0.05 level.*

For peer review only - http://bmjopen.bmj.com/site/about/guidelines.xhtml

Supplementary Table 14. Multiple comparisons of seasonal intussusception in children in different seasons for different age groups

Dependent variable		Season (A)	Season (B)	Mean difference (A-B)	Standard error	p value	95% CI
Seasonal IS cases in children aged 2-6 months	Tukey HSD	Winter	Spring	-.003	.0905	1.000	-0.25 , 0.24
			Summer	-.073	.0651	.798	-0.25 , 0.11
			Monsoon	.019	.0623	.998	-0.15 , 0.19
			Autumn	.123	.0696	.395	-0.07 , 0.31
		Spring	Winter	.003	.0905	1.000	-0.24 , 0.25
			Summer	-.070	.0880	.933	-0.31 , 0.17
			Monsoon	.022	.0859	.999	-0.21 , 0.26
			Autumn	.126	.0914	.643	-0.12 , 0.38
		Summer	Winter	.073	.0651	.798	-0.11 , 0.25
			Spring	.070	.0880	.933	-0.17 , 0.31
			Monsoon	.092	.0585	.517	-0.07 , 0.25
			Autumn	.195*	.0663	**.027**	0.01 , 0.38
		Monsoon	Winter	-.019	.0623	.998	-0.19 , 0.15
			Spring	-.022	.0859	.999	-0.26 , 0.21
			Summer	-.092	.0585	.517	-0.25 , 0.07
			Autumn	.104	.0636	.478	-0.07 , 0.28
		Autumn	Winter	-.123	.0696	.395	-0.31 , 0.07
			Spring	-.126	.0914	.643	-0.38 , 0.12
			Summer	-.195*	.0663	**.027**	-0.38 , -0.01
			Monsoon	-.104	.0636	.478	-0.28 , 0.07
Seasonal IS cases in children aged 7-12 months	Tukey HSD	Winter	Spring	-.185	.1090	.439	-0.48 , 0.11
			Summer	-.210	.0784	.057	-0.42 , 0
			Monsoon	-.046	.0750	.973	-0.25 , 0.16
			Autumn	.021	.0839	.999	-0.21 , 0.25
		Spring	Winter	.185	.1090	.439	-0.11 , 0.48
			Summer	-.026	.1059	.999	-0.32 , 0.26
			Monsoon	.138	.1035	.667	-0.14 , 0.42
			Autumn	.206	.1101	.336	-0.1 , 0.51
		Summer	Winter	.210	.0784	.057	0 , 0.42
			Spring	.026	.1059	.999	-0.26 , 0.32
			Monsoon	.164	.0705	.136	-0.03 , 0.36
			Autumn	.231*	.0798	**.031**	0.01 , 0.45
		Monsoon	Winter	.046	.0750	.973	-0.16 , 0.25
			Spring	-.138	.1035	.667	-0.42 , 0.14
			Summer	-.164	.0705	.136	-0.36 , 0.03
			Autumn	.067	.0765	.906	-0.14 , 0.28
		Autumn	Winter	-.021	.0839	.999	-0.25 , 0.21
			Spring	-.206	.1101	.336	-0.51 , 0.1
			Summer	-.231*	.0798	**.031**	-0.45 , -0.01
			Monsoon	-.067	.0765	.906	-0.28 , 0.14
Seasonal IS cases in	Tukey HSD	Winter	Spring	.012	.0793	1.000	-0.2 , 0.23
			Summer	-.126	.0570	.176	-0.28 , 0.03
			Monsoon	-.011	.0546	1.000	-0.16 , 0.14

For peer review only

children aged 7-12 months			Autumn	.016	.0610	.999	-0.15 , 0.18
		Spring	Winter	-.012	.0793	1.000	-0.23 , 0.2
			Summer	-.138	.0771	.378	-0.35 , 0.07
			Monsoon	-.024	.0753	.998	-0.23 , 0.18
			Autumn	.004	.0801	1.000	-0.21 , 0.22
		Summer	Winter	.126	.0570	.176	-0.03 , 0.28
			Spring	.138	.0771	.378	-0.07 , 0.35
			Monsoon	.115	.0513	.167	-0.03 , 0.25
			Autumn	.143	.0581	.102	-0.02 , 0.3
		Monsoon	Winter	.011	.0546	1.000	-0.14 , 0.16
			Spring	.024	.0753	.998	-0.18 , 0.23
			Summer	-.115	.0513	.167	-0.25 , 0.03
			Autumn	.028	.0557	.987	-0.12 , 0.18
		Autumn	Winter	-.016	.0610	.999	-0.18 , 0.15
			Spring	-.004	.0801	1.000	-0.22 , 0.21
			Summer	-.143	.0581	.102	-0.3 , 0.02
			Monsoon	-.028	.0557	.987	-0.18 , 0.12

*Note: * The difference is significant at p<0.05 level.*

For peer review only

For peer review only - http://bmjopen.bmj.com/site/about/guidelines.xhtml

Revised Title: Association of meteorological parameters with intussusception in children aged under-two years: Results from a multisite bidirectional surveillance over seven years in India

Manuscript ID: bmjopen-2020-043687.R2

Corresponding author: Manoja Kumar Das, MD

The Institute Ethics Committees (IECs) of all the participating institutes reviewed and approved the study protocol titled "Intussusception in Infants: Multisite Retrospective Surveillance in India". The list of IECs reviewed and approved the protocol with the reference numbers for the respective IECs are listed below.

1. Name of IRB: The INCLEN Independent Ethic Committee
   IRB Ref no: IIEC-023
   Name of the Institute: The INCLEN Trust International, New Delhi, India
   Principal Investigator's Institute/Coordinating Institute

2. Name of IRB: Institutional Ethics Committee
   IRB Ref no: IEC/SKIMS Protocol#42/2015
   Name of the Institute: Sher-I-Kashmir Institute of Medical Sciences, Srinagar, Jammu & Kashmir, India

3. Name of IRB: Institutional Ethics Committee
   IRB Ref no: IEC/MAMC/50/4/2015/308
   Name of the Institute: Maulana Azad Medical College, Delhi, India

4. Name of IRB: Medanta Institutional Ethics Committee
   IRB Ref no: MICR 559/2015
   Name of the Institute: Medanta- The Medicity, Gurgaon, Haryana, India

5. Name of IRB: The INCLEN Independent Ethic Committee
   IRB Ref no: 7951/Ethics/R.Cell-15
   Name of the Institute: King George's Medical University, Lucknow, Uttar Pradesh, India

6. Name of IRB: Institutional Ethics Committee
   IRB Ref no: EC/Oct/15/20
   Name of the Institute: Choithram Hospital and Research Centre, Indore, Madhya Pradesh, India

7. Name of IRB: Institutional Ethics Committee
   IRB Ref no: 26/12/2015
   Name of the Institute: King George Hospital, Andhra Medical College, Vishakhapatnam, Andhra Pradesh, India

8. Name of IRB: Ethics Committee
   IRB Ref no: 13/12/2015
   Name of the Institute: Apollo Hospital, Hyderabad, Telengana, India

9. Name of IRB: Institutional Human Ethics Committee

For peer review only

IRB Ref no: Project No 15/294
Name of the Institute: PSG Institute of Medical Sciences, Coimbatore, Tamil Nadu, India

10. Name of IRB: Institutional Ethics Committee- Clinical Studies
IRB Ref no: 14/12/210
Name of the Institute: Apollo Hospitals, Chennai, Tamil Nadu

11. Name of IRB: Institutional Ethics Committee
IRB Ref no: IEC-06/05/2015/MCT
Name of the Institute: Government Medical College & SAT Hospital, Thiruvananthapuram, Kerala, India

12. Name of IRB: Institutional Ethics Committee
IRB Ref no: 1256/Acad
Name of the Institute: Indira Gandhi Institute of Medical Sciences, Patna, Bihar, India

13. Name of IRB: Institutional Ethics Committee
IRB Ref no: 210/5/10/2015
Name of the Institute: IMS & SUM Medical College & Hospital, Bhubaneswar, Odisha, India

14. Name of IRB: Institutional Ethics Committee
IRB Ref no: 311/27-04-16
Name of the Institute: SCB Medical College, Cuttack, Odisha, India

15. Name of IRB: Institutional Ethics Committee
IRB Ref no: 210/5/10/2015
Name of the Institute: MKCG Medical College, Berhampur, Odisha, India

16. Name of IRB: Institutional Ethics Committee
IRB Ref no: IEC/2016/197
Name of the Institute: Institute of Post Graduate Medical Education and Research, Kolkata, West Bengal, India

17. Name of IRB: Institutional Ethics Committee
IRB Ref no: MC/02/2015/274
Name of the Institute: Gauhati Medical College, Guwahati, Assam, India

18. Name of IRB: Institutional Ethics Committee
IRB Ref no: 2015/3894
Name of the Institute: Agartala Government Medical College, Agartala, Tripura, India

19. Name of IRB: Institutional Ethics Committee
IRB Ref no: FEHJ/IEC/15/0023
Name of the Institute: Fortis Escorts Hospital, Jaipur, Rajasthan, India

20. Name of IRB: Institutional Ethics Committee
IRB Ref no: IEC/46/2016
Name of the Institute: MP Shah Government Medical College, Jamnagar, Gujarat, India

21. Name of IRB: Institutional Ethics Committee

For peer review only - http://bmjopen.bmj.com/site/about/guidelines.xhtml

For peer review only

IRB Ref no: IEC/Pharm/288/15
Name of the Institute: Grant Medical College & JJ Hospital, Mumbai, Maharashtra, India

For peer review only

For peer review only - http://bmjopen.bmj.com/site/about/guidelines.xhtml

Manuscript Title: Intussusception in children under-two years, seasonal variations and associations with meteorological parameters: Results from multisite bidirectional surveillance in India over eight years

STROBES Checklist

**Section/Topic**	**Item #**	**Recommendation**	**Reported on page #**
**Title and abstract**	1	(*a*) Indicate the study's design with a commonly used term in the title or the abstract	1
		(*b*) Provide in the abstract an informative and balanced summary of what was done and what was found	1-2
**Introduction**			
Background/rationale	2	Explain the scientific background and rationale for the investigation being reported	4
Objectives	3	State specific objectives, including any pre-specified hypotheses	5
**Methods**			
Study design	4	Present key elements of study design early in the paper	5
Setting	5	Describe the setting, locations, and relevant dates, including periods of recruitment, exposure, follow-up, and data collection	5
Participants	6	(*a*) *Cohort study*—Give the eligibility criteria, and the sources and methods of selection of participants. Describe methods of follow-up<br>*Case-control study*—Give the eligibility criteria, and the sources and methods of case ascertainment and control selection. Give the rationale for the choice of cases and controls<br>*Cross-sectional study*—Give the eligibility criteria, and the sources and methods of selection of participants	5
		(*b*) *Cohort study*—For matched studies, give matching criteria and number of exposed and unexposed<br>*Case-control study*—For matched studies, give matching criteria and the number of controls per case	
Variables	7	Clearly define all outcomes, exposures, predictors, potential confounders, and effect modifiers. Give diagnostic criteria, if applicable	5-6
Data sources/ measurement	8*	For each variable of interest, give sources of data and details of methods of assessment (measurement). Describe comparability of assessment methods if there is more than one group	5-6
Bias	9	Describe any efforts to address potential sources of bias	6

For peer review only

Study size	10	Explain how the study size was arrived at	5-6
Quantitative variables	11	Explain how quantitative variables were handled in the analyses. If applicable, describe which groupings were chosen and why	6
Statistical methods	12	(*a*) Describe all statistical methods, including those used to control for confounding	6-7
		(*b*) Describe any methods used to examine subgroups and interactions	6-7
		(*c*) Explain how missing data were addressed	6
		(*d*) *Cohort study*—If applicable, explain how loss to follow-up was addressed<br>*Case-control study*—If applicable, explain how matching of cases and controls was addressed<br>*Cross-sectional study*—If applicable, describe analytical methods taking account of sampling strategy	6-7
		(*e*) Describe any sensitivity analyses	6-7
**Results**			
Participants	13*	(a) Report numbers of individuals at each stage of study—eg numbers potentially eligible, examined for eligibility, confirmed eligible, included in the study, completing follow-up, and analysed	7
		(b) Give reasons for non-participation at each stage	NA
		(c) Consider use of a flow diagram	Narration given
Descriptive data	14*	(a) Give characteristics of study participants (eg demographic, clinical, social) and information on exposures and potential confounders	7-8
		(b) Indicate number of participants with missing data for each variable of interest	7-8 and tables
		(c) *Cohort study*—Summarise follow-up time (eg, average and total amount)	
Outcome data	15*	*Cohort study*—Report numbers of outcome events or summary measures over time	7-11<br>Given in tables and results
		Case-control study—Report numbers in each exposure category, or summary measures of exposure	
		Cross-sectional study—Report numbers of outcome events or summary measures	
Main results	16	(*a*) Give unadjusted estimates and, if applicable, confounder-adjusted estimates and their precision (eg, 95% confidence interval). Make clear which confounders were adjusted for and why they were included	7-11<br>Given in tables and results
		(*b*) Report category boundaries when continuous variables were categorized	7-11<br>Given in tables and

For peer review only

			results
		(*c*) If relevant, consider translating estimates of relative risk into absolute risk for a meaningful time period	NA
Other analyses	17	Report other analyses done—eg analyses of subgroups and interactions, and sensitivity analyses	7-11<br>Given in tables and results
**Discussion**			
Key results	18	Summarise key results with reference to study objectives	11-12
Limitations	19	Discuss limitations of the study, taking into account sources of potential bias or imprecision. Discuss both direction and magnitude of any potential bias	14
Interpretation	20	Give a cautious overall interpretation of results considering objectives, limitations, multiplicity of analyses, results from similar studies, and other relevant evidence	11-14
Generalisability	21	Discuss the generalisability (external validity) of the study results	14
**Other information**			
Funding	22	Give the source of funding and the role of the funders for the present study and, if applicable, for the original study on which the present article is based	17

*Give information separately for cases and controls in case-control studies and, if applicable, for exposed and unexposed groups in cohort and cross-sectional studies.

Note: An Explanation and Elaboration article discusses each checklist item and gives methodological background and published examples of transparent reporting. The STROBE checklist is best used in conjunction with this article (freely available on the Web sites of PLoS Medicine at http://www.plosmedicine.org/, Annals of Internal Medicine at http://www.annals.org/, and Epidemiology at http://www.epidem.com/). Information on the STROBE Initiative is available at www.strobe-statement.org.

For peer review only

BMJ Open

Association of meteorological parameters with intussusception in children aged under-two years: Results from a multisite bidirectional surveillance over seven years in India

Journal:	*BMJ Open*
Manuscript ID	bmjopen-2020-043687.R3
Article Type:	Original research
Date Submitted by the Author:	02-Mar-2021
Complete List of Authors:	Study Group, The INCLEN Intussusception Surveillance; The INCLEN Trust International<br>Das, Manoja; The INCLEN Trust International
**Primary Subject Heading**:	Public health
Secondary Subject Heading:	Epidemiology, Paediatrics
Keywords:	EPIDEMIOLOGY, Public health < INFECTIOUS DISEASES, Community child health < PAEDIATRICS, Paediatric gastroenterology < PAEDIATRICS

SCHOLARONE™
Manuscripts

I, the Submitting Author has the right to grant and does grant on behalf of all authors of the Work (as defined in the below author licence), an exclusive licence and/or a non-exclusive licence for contributions from authors who are: i) UK Crown employees; ii) where BMJ has agreed a CC-BY licence shall apply, and/or iii) in accordance with the terms applicable for US Federal Government officers or employees acting as part of their official duties; on a worldwide, perpetual, irrevocable, royalty-free basis to BMJ Publishing Group Ltd ("BMJ") its licensees and where the relevant Journal is co-owned by BMJ to the co-owners of the Journal, to publish the Work in this journal and any other BMJ products and to exploit all rights, as set out in our licence.

The Submitting Author accepts and understands that any supply made under these terms is made by BMJ to the Submitting Author unless you are acting as an employee on behalf of your employer or a postgraduate student of an affiliated institution which is paying any applicable article publishing charge ("APC") for Open Access articles. Where the Submitting Author wishes to make the Work available on an Open Access basis (and intends to pay the relevant APC), the terms of reuse of such Open Access shall be governed by a Creative Commons licence – details of these licences and which Creative Commons licence will apply to this Work are set out in our licence referred to above.

Other than as permitted in any relevant BMJ Author's Self Archiving Policies, I confirm this Work has not been accepted for publication elsewhere, is not being considered for publication elsewhere and does not duplicate material already published. I confirm all authors consent to publication of this Work and authorise the granting of this licence.

For peer review only

Article Type: Original Research

Revised Title: Association of meteorological parameters with intussusception in children aged under-two years: Results from a multisite bidirectional surveillance over seven years in India

Short title: Association of intussusception in Indian children with meteorological parameters

Author Information

The INCLEN Intussusception Surveillance Network Study Group

The INCLEN Trust International, Okhla Industrial Area, Phase 1, New Delhi -110020, India

E-mail: drmanojkdas@gmail.com

Corresponding Author

Manoja Kumar Das

Director Projects, The INCLEN Trust International, F1/5, Okhla Industrial Area, Phase 1, New Delhi -110020, INDIA

Phone: Off: +91-11-47730000; Mob: +91-9810203768; Fax: +91-11-47730001

E-mail: manoj@inclentrust.org

For peer review only

Author information

'The INCLEN Intussusception Surveillance Network Study Group' list of authors

1. Manoja Kumar Das

Director Projects, The INCLEN Trust International, New Delhi, India

Email: manoj@inclentrust.org

2. Narendra Kumar Arora

Executive Director, The INCLEN Trust International, New Delhi, India

Email: nkarora@inclentrust.org

3. Bini Gupta

Assistant Research Officer, The INCLEN Trust International, New Delhi, India

Email: drbini.gupta02@gmail.com

4. Sharad Srivastava

Assistant Program Officer, The INCLEN Trust International, New Delhi, India

Email: sharad.srivastav@inclentrust.org

5. Apoorva Sharan

Research Officer, The INCLEN Trust International, New Delhi, India

Email: apoorva@inclentrust.org

6. Arindam Ray

Senior Program Officer, Bill and Melinda Gates Foundation, India Country Office, New Delhi, India

Email: arindam.ray@gatesfoundation.org

7. Ashish Wakhlu

Professor, Department of Paediatric Surgery, King George's Medical University, Lucknow, Uttar Pradesh, India

Email: ashish_wakhlu@hotmail.com

For peer review only

8. Bhadresh R Vyas

Professor, Department of Paediatrics, MP Shah Government Medical College, Jamnagar, Gujarat, India

Email: bhadreshrvyas@yahoo.co.uk

9. Javeed Iqbal Bhat

Assistant Professor, Department of Paediatrics, Sher-I-Kashmir Institute of Medical Sciences, Srinagar, Jammu & Kashmir, India

Email: drjaveediqbal@gmail.com

10. Jayanta K. Goswami

Professor, Department of Paediatric Surgery, Gauhati Medical College, Guwahati, Assam, India

Email: jayantagoswami@hotmail.com

11. John Mathai

Professor, Department of Paediatrics, PSG Institute of Medical Sciences, Coimbatore, Tamil Nadu, India

Email: psg_peds@yahoo.com

12. Kameswari K.

Professor, Department of Paediatric Surgery, Andhra Medical College, Vishakhapatnam, Andhra Pradesh, India

Email: kameswari1956@gmail.com

13. Lalit Bharadia

Consultant Paediatric Gastroenterologist, Fortis Escorts Hospital, Jaipur, Rajasthan, India

Email: lalitbharadia@gmail.com

14. Lalit Sankhe

For peer review only

Assistant Professor, Department of Community Medicine, Grant Medical College & JJ Hospital, Mumbai, Maharashtra, India

Email: sankhelalit@yahoo.com

15. Ajayakumar M.K.

Professor, Department of Paediatric Surgery, Government Medical College & SAT Hospital, Thiruvananthapuram, Kerala, India

Email: drajaykumarmk@gmail.com

16. Neelam Mohan

Consultant Paediatrics Gastroenterology, Medanta—The Medicity, Gurgaon, Haryana, India

Email: drneelam@yahoo.com

17. Pradeep K. Jena

Professor, Department of Paediatric Surgery, SCB Medical College, Cuttack, Odisha, India

Email: drpkjena@gmail.com

18. Rachita Sarangi

Professor, Department of Paediatrics, IMS & SUM Medical College & Hospital, Bhubaneswar, Odisha, India

Email: rachitapaedia@gmail.com

19. Rashmi Shad

Consultant Paediatrics, Choithram Hospital and Research Centre, Indore, Madhya Pradesh, India

Email: drrashmishad@yahoo.com

20. Sanjib K. Debbarma

Associate Professor, Department of Paediatrics, Agartala Government Medical College, Agartala, Tripura, India

For peer review only

For peer review only - http://bmjopen.bmj.com/site/about/guidelines.xhtml

Email: dr_sanjibdb@rediffmail.com

21. Shyamala J.

Consultant Paediatrics, Apollo Hospitals, Chennai, Tamil Nadu

Email: jaymoorthy@hotmail.com

22. Simmi K. Ratan

Professor, Department of Paediatric Surgery, Maulana Azad Medical College, Delhi, India

Email: drjohnsimmi@yahoo.com

23. Suman Sarkar

Assistant Professor, Department of Paediatrics, Institute of Post Graduate Medical Education and Research, Kolkata, West Bengal, India

Email: dr.sumansarkar@gmail.com

24. Vijayendra Kumar

Professor, Department of Paediatric Surgery, Indira Gandhi Institute of Medical Sciences, Patna, Bihar, India

Email: drvijayendrakr@rediffmail.com

25. Narendra Behera

Professor, Department of Pediatrics, MKCG Medical College, Berhampur, Odisha, India

Email: naribehera@yahoo.co.in

26. Rajamani G.

Professor, Department of Paediatric Surgery, Coimbatore Medical College, Coimbatore, Tamil Nadu, India

E mail: drgrajamani@gmail.com

27. Anand P. Dubey

Professor, Department of Paediatrics, Maulana Azad Medical College, Delhi, India

Email: Anand_dubey52@hotmail.com

For peer review only - http://bmjopen.bmj.com/site/about/guidelines.xhtml

28. Atul Gupta

Consultant Paediatric Surgery, Fortis Escorts Hospital, Jaipur, Rajasthan, India

Email: atulgupta26878@gmail.com

29. Cenita J. Sam

Professor, Department of Paediatric Surgery, PSG Institute of Medical Sciences, Coimbatore, Tamil Nadu, India

Email: cenitasivamani@yahoo.com

30. Gowhar Nazir Mufti

Assistant Professor, Department of Paediatric Surgery, Sher-I-Kashmir Institute of Medical Sciences, Srinagar, Jammu & Kashmir, India

Email: gowharmufti@yahoo.co.in

31. Harsh Trivedi

Professor, Department of Paediatric Surgery, MP Shah Government Medical College, Jamnagar, Gujarat, India

Email: trivediharsh@indiatimes.com

32. Jimmy Shad

Consultant Paediatric Surgery, Apollo Hospitals, Chennai, Tamil Nadu, India

Email: shadjimmy@yahoo.co.in

33. Kaushik Lahiri

Consultant, Department of Paediatric Surgery, Gauhati Medical College, Guwahati, Assam, India

Email: Kaushik_1671979@rediffmail.com

34. Krishnaswamy R

Chief Medical Officer, Masonic Children's Hospital, Coimbatore, Tamil Nadu, India

Email: krishnaswamy24@yahoo.com

For peer review only

35. Meera Luthra

Consultant Paediatric Surgery, Medanta- The Medicity, Gurgaon, Haryana, India

Email: meera.luthra@medanta.org

36. Padmalatha P

Professor, Department of Paediatrics, Andhra Medical College, Vishakhapatnam, Andhra Pradesh, India

Email: padmap_2000@yahoo.com

37. Rakesh Kumar

Associate Professor, Department of Paediatrics, Indira Gandhi Institute of Medical Sciences, Patna, Bihar, India

Email: drjaiswalrakesh@gmail.com

38. Ruchirendu Sarkar

Professor, Department of Paediatric Surgery, Institute of Post Graduate Medical Education and Research, Kolkata, West Bengal, India

Email: ruchirendu@gmail.com

39. Santosh Kumar A.

Professor, Department of Paediatrics, Government Medical College & SAT Hospital, Thiruvananthapuram, Kerala, India

Email: hod.pediatrics.sath@gmail.com

40. Subrat Kumar Sahoo

Associate Professor, Department of Paediatric Surgery, IMS & SUM Medical College & Hospital, Bhubaneswar, Odisha, India

Email: dr_subratsahoo@yahoo.com

41. Sunil K. Ghosh

For peer review only

For peer review only - http://bmjopen.bmj.com/site/about/guidelines.xhtml

Associate Professor, Department of Pediatric Surgery, Agartala Government Medical College, Agartala, Tripura, India

Email: sunilkrghosh752@gmail.com

42. Sushant Mane

Assistant Professor, Department of Paediatrics, Grant Medical College & JJ Hospital, Mumbai, Maharashtra, India

Email: drsush2006@gmail.com

43. Arun Dash

Professor, Department of Pediatric Surgery, MKCG Medical College, Berhampur, Odisha

Email: dasharun@gmail.com

44. Bashir Ahmad Charoo

Professor, Department of Paediatrics, Sher-I-Kashmir Institute of Medical Sciences, Srinagar, Jammu & Kashmir, India

Email: charoobash@gmail.com

45. Bikasha Bihary Tripathy

Associate Professor, Department of Paediatric Surgery, IMS & SUM Medical College & Hospital, Bhubaneswar, Odisha, India

Email: bbtripathy.dr@gmail.com

46. Rajendra Prasad G.

Professor, Department of Paediatric Surgery, Andhra Medical College, Vishakhapatnam, Andhra Pradesh, India

Email: dr.grajendraprasad@gmail.com

47. Harish Kumar S

Paediatrics Radiologist, Apollo Hospitals, Chennai, Tamil Nadu, India

Email: drharishkumar.s@gmail.com

For peer review only

For peer review only - http://bmjopen.bmj.com/site/about/guidelines.xhtml

48. Jothilakshmi K.

Professor, Department of Paediatrics, PSG Institute of Medical Sciences, Coimbatore, Tamil Nadu, India

Email: drjothi.cbe@gmail.com

49. Nihar Ranjan Sarkar

Associate Professor, Department of Radiology, Institute of Post Graduate Medical Education and Research, Kolkata, West Bengal, India

Email: dr.niharsarkar@gmail.com

50. Pavai Arunachalam

Professor, Department of Paediatric Surgery, PSG Institute of Medical Sciences, Coimbatore, Tamil Nadu, India

Email: pavai4321@yahoo.com

51. Satya Sundar G. Mohapatra

Professor, Department of Radiology, IMS & SUM Medical College & Hospital, Bhubaneswar, Odisha, India

Email: drartifact@gmail.com

52. Saurabh Garge

Consultant Paediatric Surgery, Choithram Hospital and Research Centre, Indore, Madhya Pradesh, India

Email: saurabhgarge8@gmail.com

For peer review only

Abstract

Objectives: The study aimed to document the association between intussusception in Indian children and meteorological parameters and examine regional variations.

Design: A bidirectional (retrospective and prospective) surveillance between July 2010 and September 2017.

Setting: At 20 hospitals in India, retrospective case record review during July 2010 and March 2016 and prospective surveillance during April 2016 and September 2017 were performed.

Participants: 2161 children aged 2-24 months with first intussusception episode were included.

Interventions: The monthly mean meteorological parameters (temperature, sunshine, rainfall, humidity, and wind speed) for the study sites were collected.

Methods: The association between monthly intussusception cases and meteorological parameters were examined at pooled, regional and site levels using Pearson's (r) and Spearman's rank-order (ρ) correlation, factorial analysis-of-variance and Poisson regression or negative binomial regression analyses.

Results: The intussusception cases were highest in summer and lowest in autumn seasons. Pearson's correlation analysis showed that temperature (r=0.056;p<0.05), wind speed (r=0.134;p<0.01) and humidity (r=0.075;p<0.01) were associated with monthly intussusception cases. Spearman's rank-order correlation analysis found that temperature (ρ=0.049;p<0.05), wind speed (ρ=0.096;p<0.01) and sunshine (ρ=0.051;p<0.05) were associated with monthly intussusception cases. Poisson regression analysis resulted that monthly intussusception case was associated with rising temperature (North region, p<0.01 and East region, p<0.05), sunshine (North region,p<0.01), humidity (East region,p<0.01), and wind speed (East region,p<0.01). Factorial analysis-of-variance revealed a significant seasonal difference in intussusception cases for pooled level (p<0.05), 2-6 months age group (p<0.05),

For peer review only - http://bmjopen.bmj.com/site/about/guidelines.xhtml

and North region ($p<0.01$). Significant differences in intussusception cases between summer and autumn seasons were observed for pooled ($p<0.01$), children aged 2-6 months ($p<0.05$), and 7-12 months ($p<0.05$).

Conclusions: Significant correlations between intussusception cases and temperature, humidity, and wind speed were observed at pooled and regional level in India. A peak in summer months was noted, which may be used for prediction, early detection and referral for appropriate management of intussusception.

For peer review only

Keywords: intussusception, children, climate, meteorological parameters, ambient temperature, season, India

For peer review only - http://bmjopen.bmj.com/site/about/guidelines.xhtml

Strengths and limitations of the study

- This study included intussusception cases in children from 20 sites in India representing different geographic and climate regions.
- Relatively large number of intussusception cases in children over seven years were analysed at pooled, regional and site levels using multiple statistical tests for consistency and triangulation.
- Consistency in the correlation between the meteorological parameters and intussusception using multiple statistical tests was reassuring.
- Data for over five years were collected by retrospective surveillance may be a limitation.

For peer review only

For peer review only - http://bmjopen.bmj.com/site/about/guidelines.xhtml

1. Introduction

Intussusception is the most common acute abdomen with intestinal obstruction in children, mostly occurring in infants [1,2]. The incidence of intussusception varies widely with a mean of 74 per 100,000 (range: 9-328) among infants with the peak during 5-7 months of age [3]. The available reports from India suggest that intussusception incidence varies from 17.7 to 254 per 100,000 child-years [4,5]. These children usually present with abdominal pain or excessive crying, vomiting, bloody stools, and may have abdominal mass. Ultrasound is the leading diagnostic tool. The majority of the patients can be managed by reduction method and some may need surgery. The aetiology of intussusception remains unknown in the majority of cases [6].

Intussusception has attracted attention due to its association with the rotavirus vaccine (RVV). India introduced RVV under the universal immunization program (UIP) in 2016 and expanded countrywide by 2019. A recent publication by our group reported regional variation in the intussusception cases among Indian children under-two years of age [7]. More number of cases were observed during March to June months (summer months) suggesting seasonal variation. Other studies from India also reported seasonal variations [5,8-11]. There is variation in the seasonality of intussusception occurrence globally. A higher number of intussusception cases have been reported during warmer months from some countries [12-16], while no seasonal variation was reported from other countries [17-23]. Reports from China found the intussusception caseload to be associated with meteorological parameters like temperature, sunshine, and humidity [12,24]. In several reports from different countries, the association between meteorological factors and intussusception remains unclear, due to various reasons, including shorter study duration, smaller sample size, and no definite statistical methods to

For peer review only - http://bmjopen.bmj.com/site/about/guidelines.xhtml

explore the association. Although seasonal variations have been observed, no association between the intussusception and meteorological parameters has been reported from India.

Multisite surveillance in India documented the epidemiology and trend of intussusception in children as part of the vaccine safety surveillance linked to the RVV introduction. Using the data collected by the Indian surveillance network, this study investigated the possible association between intussusception cases and various meteorological parameters and seasonality at the site, regional and national levels.

2. Methods

2.1. Study area and participating hospitals

This hospital-based sentinel surveillance for intussusception in under-two year children at 20 tertiary care hospitals in India included both prospective and retrospective periods. This bidirectional surveillance was comprised of retrospective (July 1, 2010, to March 31, 2016) and prospective (April 1, 2016, to September 30, 2017) periods of data collection. The study sites were selected from four regions (north, south, east, and west) of the country ensuring a mix of public and private tertiary care hospitals (North: three public and two private; South: two public and three private; East: five public and one private; West: 2 public, and one private) (Supplementary Figure 1). The study sites represented different climate zones in India. The method of study site selection has been detailed in the protocol published [25].

2.2. Case definition, case selection, and data collection

The children aged 2-24 months admitted to these hospitals with intussusception were eligible. For the retrospective surveillance period, the potential cases were identified using the International Classification of Diseases (ICD 9/10) or the diagnoses in the medical record

For peer review only - http://bmjopen.bmj.com/site/about/guidelines.xhtml

section (Supplementary Table 1). These case records were reviewed to identify the intussusception cases and all identified cases were recruited. For the prospective surveillance period, all age-eligible children were screened, tracked till final diagnosis and all intussusception cases were recruited after written informed consent. Only the first intussusception cases were included in the analysis. The cases with the first episode of intussusception were identified from the history and/or from parents. For the confirmed cases, data including demography, clinical features, management, and outcome were abstracted using case record forms. External experts visited the study sites to assess the protocol adherence for case selection, record retrieval, and data abstraction. Data team members visited the sites and repeated the case retrieval process for the years, 2014, 2015, and 2016 to assess the retrieval completeness. The data queries were clarified referring to the source documents.

2.3. Meteorological data collection

For the study sites, monthly mean meteorological data including temperature (^{0}Centigrade, ^{0}C), sunshine (total hours), rainfall (millimeter, mm), humidity (%) and wind speed (kilometer per hour, Kmph) were collected from the website, World Weather Online (https://www.worldweatheronline.com/). These meteorological data collected for the period July 2010 through September 2017 were available in the public domain.

2.4. Data Management and analysis

The descriptive analysis included proportions, means (with standard deviations), and median (with interquartile range, IQR). There was no definite population catchment area for the hospitals and the estimation of intussusception incidence was not possible. The calendar months were grouped into seasons (summer, rainy, autumn, winter, and spring) for the individual sites and regions (Supplementary Table 2). Chi-squared goodness of fit test was used

For peer review only

For peer review only - http://bmjopen.bmj.com/site/about/guidelines.xhtml

to examine the periodicity of intussusception and monthly differences. Kruskal-Wallis H test (χ^2) was performed to document the differences between >3 groups (years, months seasons and temperature ranges: <20°C, 21°C-25°C, 26°C-30°C, and >30°C). Pearson's correlation (r) analysis explored the association between monthly intussusception cases (dependent variable) and monthly mean meteorological parameters separately (independent variables). Spearman's rank-order correlation (ρ) analysis was also conducted to explore whether the monthly intussusception and monthly mean meteorological parameters covary. Factorial analysis-of-variance (F) was used to analyze the variations in the intussusception cases during the different seasons (dependent variable: seasonal intussusception cases and independent variable: season and year). Poisson regression or negative binomial regression (β (for parameters with wide dispersion) analyses were performed to evaluate the relationships between the monthly intussusception cases (dependent variable) and the monthly mean meteorological parameters (independent variables). The association between intussusception and meteorological parameters was examined at pooled, regional, and site levels with 95% confidence interval (CI) estimation. Statistical significance was considered if $p<0.05$. Statistical analysis was performed using STATA version 15.0 (Stata Corp LLC, Texas, USA).

2.5. Ethical issues

The study protocol was reviewed and approved by all the ethics review committees of each participating institute (Supplementary Document 2). Confidentiality in data handling was maintained. The participants under the prospective component were recruited after written informed consent from the parent/legal guardian.

2.6. Patient and Public Involvement

For peer review only

For peer review only - http://bmjopen.bmj.com/site/about/guidelines.xhtml

The study participants or the public were not involved in the design, or conduct, or reporting, or dissemination plans of our research. The findings would be relevant for care-seeking and case management for intussusception in children. The results published shall be disseminated to the public through social media and healthcare providers.

3. Results

3.1 Demography data

From July 2010 through September 2017, 2279 children aged 2-24 months with intussusception were recruited. Out of these, 118 children with a history of intussusception were excluded. Thus, 2161 children with the first intussusception were analyzed. The demography and regional distribution of the intussusception cases across the years are shown in Table 1. There were 1431 (66.2%) boys and 730 (33.8%) girls (male-female ratio, 1.96). Majority (n=1653, 76.5%) were infants. The number of cases increased across the years with the highest (31%) jump between 2014 and 2015. During 2010-2014, the children aged 7-12 months had a higher share, which changed during 2015-2017. The South region contributed the highest (50%), followed by East (27.4%), North (17.1%) and West (5.4%) regions, which were consistent across the years.

3.2. Meteorological data

The monthly meteorological parameters for the regions are shown in Supplementary Table 3. The monthly meteorological parameters for each site are given in Supplementary Table 4. Across the regions, April to June and December-February had the highest and lowest temperatures respectively. The pattern of meteorological parameters across the regions is shown in Supplementary Figure 2.

For peer review only

For peer review only - http://bmjopen.bmj.com/site/about/guidelines.xhtml

3.3. Monthly and seasonal cycles of intussusception

The Chi-squared goodness of fit test revealed obvious monthly (χ^2-statistic= 71.36; p<0.01) and seasonal differences (χ^2-statistic= 382.28; p<0.01) in the intussusception caseload. Also, the Kruskal-Wallis H test revealed significant differences in the intussusception cases for months (p<0.01) and seasons (p<0.01). As shown in Figure 1, more intussusception cases were observed during March in the South region, June in North and East regions, all during the summer season. Not much variation in cases was observed in the West region. The intussusception cases were lowest in September and October months during these years. The peak and lowest seasons were summer (March-June; n=661, 30.6% cases) and autumn (September-October; 14.5% cases), respectively (Supplementary Figure 3).

3.4. Correlation between the intussusception and meteorological factors

Table 2 shows Pearson's correlation analysis for pooled, regions and age groups. For pooled data, the temperature (r=0.056; p<0.05), wind speed (r=0.134; p<0.01) and humidity (r=0.075; p<0.01) had significant association with intussusception. At regional level, intussusception cases had association with temperature for East region (r=0.052; p<0.05); with rainfall for North (r=-0.103; p<0.01) and East regions (r=0.073; p<0.01); with wind speed for all regions (r=-0.084 to 0.21; p<0.01); with humidity for three regions except West region (r=-0.373 to 0.161; p<0.01); and with sunshine for North region (r=0.163; p<0.01). For the age groups, intussusception cases had association with temperature for 7-12 months (r=0.068; p<0.01), with wind speed for all age groups (r=-0.092 to 0.125; p<0.01), with humidity for 7-12 months (r=0.069; p<0.01) and 13-24 months (r=0.076; p<0.01) age groups. The Pearson correlation analysis for intussusception cases with the meteorological parameters for the study sites is given in Supplementary Table 5. Significant correlations for intussusception cases with

For peer review only - http://bmjopen.bmj.com/site/about/guidelines.xhtml

temperature were observed for three sites, with rainfall for two sites, with wind speed and humidity for five sites each, and with sunshine for three sites.

Table 3 shows Spearman's rank-order correlation analysis for pooled, regions and age groups. For pooled data, the temperature (ρ=0.049; p<0.05), wind speed (ρ=0.096; p<0.01) and sunshine (ρ=0.051; p<0.05) were associated with intussusception. At regional level, intussusception cases had association with temperature for North region (ρ=0.110; p<0.05); with wind speed for North (ρ=0.183; p<0.01) and East (ρ=0.109; p<0.01) regions; with humidity for North (ρ=-0.114; p<0.05) and East (ρ=-0.102; p<0.05) regions; and with sunshine for North region ((ρ=0.119; p<0.05). For the age groups, intussusception cases had association with temperature for 7-12 months ((ρ=0.064; p<0.01), with wind speed for all age groups ((ρ=0.063 to 0.106; p<0.01), with humidity (ρ=0.049; p<0.05) and sunshine (ρ=0.048; p<0.05) for 7-12 months age group. The Spearman's Rank correlation analysis for intussusception cases with the meteorological parameters for the study sites are given in Supplementary Table 6. Significant correlations for intussusception cases with temperature were observed for one site, with rainfall for two sites, with wind speed for three sites, with humidity for five sites, and with sunshine for two sites.

Regression analyses (Poisson regression and negative binomial regression) revealed significant association between intussusception cases and temperature for pooled level (β=0.026; 95% CI 0.011, 0.041; p<0.01) North (β=0.027; 95% CI 0.007, 0.047; p<0.01) and East (β=0.029; 95% CI 0.001, 0.057; p<0.05) regions; wind speed for pooled level (β=0.082; 95% CI 0.061, 0.103; p<0.01), North (β=0.208; 95% CI 0.101, 0.315; p<0.01) and East (β=0.091; 95% CI 0.042, 0.141; p<0.01) regions; humidity for pooled level (β=0.011; 95% CI 0.007, 0.016; p<0.01), East (β=-0.021; 95% CI -0.035, -0.007; p<0.01) region and sunshine for pooled level (β=0.002;

For peer review only - http://bmjopen.bmj.com/site/about/guidelines.xhtml

95% CI 0, 0.003; p<0.01), North (β=0.004; 95% CI 0.002, 0.007; p<0.01) region (Table 4). Associations between the intussusception cases and temperature and humidity were evident across different age groups (p<0.01). The association was also significant at study sites level for temperature (three sites), humidity (six sites) and sunshine (five sites) (Supplementary Table 7).

The monthly average temperatures at the sites and regions varied widely; 14°C to 35°C in North, 23°C to 31°C in South, 20°C to 32°C in East and 22°C to 31°C in West regions. Chi-squared goodness of fit test showed significant difference at 20°C–30°C for pooled, North and South regions (p<0.01), indicating that the intussusception cases occurred more during this temperature (Supplementary Table 8/13). Kruskal-Wallis H test found significant variation in the intussusception cases between the different temperature categories for pooled (χ^2=15.933; p<0.01), North (χ^2=21.054; p<0.01) and South (χ^2=50.625; p<0.01) regions (Supplementary Table 9/14). The number of intussusception cases increased when the mean daily temperature and the wind speed increased and mean daily humidity decreased at the pooled level and for North and East regions (Supplementary Figure 4-8).

3.5. Correlation between the intussusception, seasons and age groups

Factorial analysis-of-variance revealed significant difference in seasonal intussusception cases at pooled level (F=1.442; p<0.05), for 2-6 months age group (F=1.579; p<0.05) and for North region (F=1.809; p<0.01) (Supplementary Table 10/8 and 11/9). Multiple comparison revealed significant difference in the intussusception cases between the summer and autumn seasons (mean difference=0.569; 95% CI 0.11-1.03; p<0.01) at the pooled level (Supplementary Table 12/10). Significant differences in the intussusception cases between the summer and monsoon (mean difference=0.165; 95% CI 0.04-0.29, p<0.01), summer and autumn (mean difference=0.179; 95% CI 0.03-0.32, p<0.01), spring and monsoon (mean difference=0.276;

For peer review only

95% CI 0.09-0.46, p<0.01) and spring and autumn (mean difference=0.290; 95% CI 0.09-0.49, p<0.01) were observed for North region (Supplementary Table 13/11). Significant differences in the intussusception cases were observed between the summer and autumn for children aged 2-6 months (mean difference=0.195; 95% CI 0.01-0.38, p<0.05) and for children aged 7-12 months (mean difference=0.231; 95% CI 0.01-0.45, p<0.05) (Supplementary Table 14/12).

4. Discussion

The seasonal influence on intussusception cases has been proposed, but with inconsistent findings from different countries and different parts of India. This study is the first to explore the association between intussusception and monthly mean meteorological parameters (temperature, rainfall, wind speed, humidity, and sunshine) in India. This study comprising 2161 children with the first intussusception from 20 sites in India documented monthly and seasonal variations. Intussusception case peaks were observed during summer months (Mar-June) and troughs during autumn months (September-October). There was a positive correlation between intussusception cases and meteorological parameters like temperature, humidity, wind speed, and sunshine at pooled and regional levels and among children aged 7-12 months. The associations were consistent across analysis tests used (Pearson correlation, Spearman's Rank correlation tests, Poisson regression, or negative binomial regression). The association was more pronounced for North and East regions, where the meteorological parameters fluctuation across the months were widest. Further analysis using factorial analysis-of-variance also demonstrated seasonal inclination with peaking in the summer season (Mar-June) in India and significant difference from the autumn season (with lowest cases) (p<0.01). A significant difference in the number of intussusception cases between the temperature ranges (<20°C, 21°C -25°C, 26°C -30°C, and >30°C) was observed at pooled and regional (North and South regions) levels, which indicated a rise in intussusception cases with the rise in monthly

For peer review only - http://bmjopen.bmj.com/site/about/guidelines.xhtml

mean temperature. The intussusception cases increased with the rise in daily temperature and wind speed and fall in humidity.

The findings are consistent with the report from China in 5994 children (aged 0-12 years) with intussusception, where more cases were documented in summer months with a significant positive association with mean monthly meteorological parameters (temperature, sunshine, and precipitation) [12]. Modelling using the data from 13887 Chinese children with intussusception and applying the meteorological variation assumptions achieved a considerable agreement with the data collected [24]. These reports from China didn't include humidity as a variable.

Studies on intussusception in children from India have reported higher cases during summer (March-July) and lowest during autumn (September-November) seasons. In three studies from South India, the highest and lowest intussusception cases were observed during March-June and September-October respectively [5,9,10]. A multisite study from India reported the highest and lowest cases during March and September months respectively [8]. A study from North India observed the highest and lowest cases during June and September months [11]. A report from our group on the retrospective surveillance observed a higher number of intussusception cases during the summer months (March-June) over the year [7].

Seasonal patterns of intussusception occurrence have been observed from different countries. A higher number of intussusception cases were observed during the warmer months (May-October) than cooler months (November-April) in Taiwan [14]. Among Hong Kong children with intussusception, higher cases were observed during the summer months (May-July) [26]. Seasonality in intussusception case occurrence was observed in Korean children with highest during warmer months (July-August) and lowest during cooler months (February) [27]. Among

For peer review only - http://bmjopen.bmj.com/site/about/guidelines.xhtml

Ethiopian children, higher intussusception cases were observed during June months and lower during November months [13]. Higher intussusception cases were observed during summer months and lower during winter months among South African children [15]. Among Israeli children, more intussusception cases were observed during the summer months and lower during winter [16]. No seasonal variation was also observed in some studies from different countries. Studies from Latin American countries and North America didn't observe visible seasonal variation in cases [17,23]. Studies from Switzerland, France, New Zealand, Singapore, and China also had no sizable seasonal variations [18-22].

The inconsistencies in the seasonal variations may be due to the sample size, age groups included, and stratification in the analysis. Three studies the observed definite seasonal variation [12,14,24] and two observed a statistically significant association between the meteorological parameters and intussusception has a larger sample size (>5000 participants). The association observed in our study may be also due to the higher sample size. The reasons for the higher intussusception cases during summer are not known and the seasonality doesn't match with the rotavirus infection seasonality in India [28]. According to the reports from some developed countries (the United States and Australia), the intussusception incidence has declined over decades, and also the prevalence of prodrome infection symptoms (gastrointestinal and respiratory infections) in these children over the same period [29-31]. Further studies are needed to confirm the seasonality, association with the meteorological parameters, and variations in seasonality across the regions in India along with the influences of the preventive healthcare services coverage, care-seeking practices, and access to sanitation, hygiene, and changes in dietary practices.

For peer review only - http://bmjopen.bmj.com/site/about/guidelines.xhtml

For peer review only

A seasonal variation in another acute intestinal condition, acute appendicitis in children has been reported. More acute appendicitis cases in children during summer months and lower during spring/autumn seasons have been reported from India, Pakistan, Iraq, China, Taiwan, Italy, Germany, Canada, and United States [32-39]. Among Chinese children, associations between acute appendicitis and temperature ($p<0.01$), humidity ($p<0.01$), and sunshine ($p<0.01$) were documented to support the seasonal variation [35]. Similar to intussusception, the appendicitis incidence has also declined in developed countries over decades, possibly due to changes in hygiene, sanitation, dietary practices, access to healthcare, and reduction in infectious diseases [40]. Intussusception and acute appendicitis, are intestinal problems and some of intussusceptions have associated appendicitis [41-43]. The climate factors are unlikely to directly cause intussusception or intussusception in children. But the linkage between seasonality and meteorological parameters and acute intestinal conditions appears to be plausible, which may be mediated by intestinal and non-intestinal infections (including activation of latent viral infections), mucosal inflammatory changes, lymphoid hyperplasia, or motility alterations and dietary intake changes. Although no direct relationship with the rotavirus infection could be established, higher gastrointestinal infection during summer and early rainy seasons may be triggers for higher intussusception in the Indian context. The changes in intussusception and appendicitis incidence over time in parallel with industrialization, economic improvement, improved access to vaccination, healthcare, and sanitation and hygiene practices suggest a possible association. Such information in the Indian and developing country context is not available. Thus the seasonal variation in these conditions and association between meteorological parameters appear epidemiologically and biologically plausible, which needs further evaluation. We used Pearson's correlation coefficient as the primary analysis to document the strength and direction of the relationship between the meteorological parameters and intussusception. As a secondary analysis and checking for

For peer review only

consistency in the findings, we used Spearman's rank-order correlation analysis to identify the strength of the association between ranked parameters and intussusception variables.

The Pearson's correlation coefficient (r) and Spearman's correlation coefficient (ρ) values for different meteorological parameters were similar, although the r values for temperature, rainfall, wind speed, and humidity were higher than the ρ values, but had r value lower for sunshine than the ρ value at the pooled level. The differences between the r and ρ values were relatively small, which supports the consistent association between the variables. The consistency in findings from factorial analysis-of-variance and regression analysis with the correlation coefficients further confirms the association between the meteorological parameters and intussusception.

The study had several strengths. It included a relatively large number of intussusception cases over seven years period recruited from twenty sites representing different regions. This study explored the association between intussusception cases and meteorological parameters at site, regional and pooled national levels. There was consistency in the correlation between intussusception cases and meteorological parameters across multiple statistical methods used, which was convincing. There are several limitations in the study. A major part of this study was retrospective. The sites belong to different climate zones and had variations in the meteorological parameters. The lack of a definite catchment population and referral pattern made it difficult to estimate the intussusception incidence rate.

In conclusion, this study documented higher intussusception cases during the summer months with a positive association between the number of cases with temperature and sunshine. Also, a significant positive correlation with the humidity and wind speed was observed. The association was significant for the North and East regions with wider variations in the

For peer review only

meteorological parameters across seasons. A higher degree of suspicion for infants with the compatible clinical presentation for intussusception during the summer months may improve diagnosis and clinical care to prevent complications. The study provides evidence for predicting the intussusception pattern in children and sensitizing the healthcare providers at community and peripheral facilities for early detection and quick referral of patients to minimize the surgical interventions and avoid fatalities. Further studies are needed to confirm the observation and improve the evidence base on the potential risk factors for the season variations and the aetiologies of intussusception.

For peer review only

For peer review only - http://bmjopen.bmj.com/site/about/guidelines.xhtml

Acknowledgments

We acknowledge the support from Ministry of Health and Family Welfare, Government of India for undertaking the study. We are thankful to the hospital administrations and the clinicians at the study site institutes, who supported and facilitated undertaking of the study.

We highly value the technical guidance and inputs provided by the members of Technical Advisory Group: Mahesh K Agarwal and Pradeep Haldar, Ministry of Health and Family Welfare, Government of India; Satinder Aneja, Anju Seth and Archana Puri, Lady Hardinge Medical College, New Delhi; Ashok Patwari, Hamdard Institute of Medical Sciences & Research, New Delhi; Yogesh Kumar Sarin, Maulana Azad Medical College, New Delhi; Rakesh Aggarwal, Anshu Srivastava and Ujjal Poddar, Sanjay Gandhi Postgraduate Institute of Medical Sciences, Lucknow; Malathi Satyasekharan, Kanchi Kamakoti Chailds Trust Hospital, Chennai; Raju Sharma and Nirupam Madan, All India Institute of Medical Sciences, New Delhi; Jyoti Joshi and Deepak Polpakara, Immunization Technical Support Unit; Ministry of Health & Family Welfare, New Delhi; Umesh D. Parashar; Centers of Disease Control and Prevention, Atlanta, USA; Jan Bonhoeffer, University Children's Hospital, Basel, Switzerland and Brighton Collaboration; Naveen Thacker, Child Health Foundation, Gandhigram; Rashmi Arora, Indian Council of Medical Research, Ansari Nagar, New Delhi; and Patrick L F Zuber and Christine G. Maure, World Health Organisation, Geneva, Switzerland.

We acknowledge the contribution of the research staffs at The INCLEN Trust International: Harshpreet Kaur, Janvi Chaubey, Mrimmaya Das, Shweta Sharma and Vaibhav Jain.

We highly appreciate the efforts made by the research staffs at the study sites: Aarezo Bashir and Rafia; Sher-e-Kashmir Institute of Medical Sciences, Srinagar, Jammu & Kashmir; Prabha Shankar, Medanta-The Medicity Hospital, Gurgaon, Haryana; Anju Sharma; Maulana Azad Medical College, New Delhi; Anita Singh and Shubhanshu Srivastava, King George Medical University, Lucknow, Uttar Pradesh; Hemant Meena, Choithram Hospital, Indore, Madhya

For peer review only

For peer review only - http://bmjopen.bmj.com/site/about/guidelines.xhtml

Pradesh; Pankaj Kumar and Shashi Kant; Indira Gandhi Institute of Medical Sciences, Patna, Bihar; Goutam Benia, IMS & SUM Medical College & Hospital, Bhubaneshwar, Odisha; Prasntajyoti Mohanty, SVP Post Graduate Institute of Paediatrics, Cuttack, Odisha; Asit Pradhan, MKCG Medical College, Berhampur, Odisha; Angshuman Chatterjee, Institute of Postgraduate Medical Education and Research & SSKM Hospital, Kolkata, West Bengal; S. Yamuna, Andhra Medical College, Vishakhapatnam, Andhra Pradesh; Srinidhi Sudan, Apollo Hospitals, Hyderabad, Telengana; Rajesh Francis, Apollo Hospitals, Chennai, Tamil Nadu; T. Easter Chandru, PSG Institute of Medical Sciences, Coimbatore, Tamil Nadu; Deepthy R, Julie and Anju Shivakumar, Government Medical College & SAT Hospital, Thiruvananthapuram, Kerala; Archit Vaidya, Grant Medical College & JJ Hospital, Mumbai, Maharashtra; Nimesh Chouksey, MP Shah Government Medical College, Jamnagar, Gujarat; Nidhi Singh, Fortis Escorts Hospital, Jaipur, Rajasthan; Mrinmoy Gohain, Gauhati Medical College, Guwahati, Assam; Arpita Bhattachrjee, Saugat Ghosh and Tanusmita Debnath, Agartala Government Medical College, Agartala, Tripura.

For peer review only

For peer review only - http://bmjopen.bmj.com/site/about/guidelines.xhtml

Declarations

Ethics approval and consent to participate: The study protocol was reviewed and approved by the ethics committees of all the participating institutes (Supplementary Document 2). The participants were recruited after obtaining written informed consent.

Consent for publication: Not applicable

Availability of data and materials: All data are available with the investigators and can be provided by the corresponding author upon reasonable request.

Competing interests: The authors declare that there is no competing interests and conflict of interest.

Disclosure statement: None. There is no financial interest or benefit for the authors arisen from this project or its direct application.

Funding: This project was supported by the Bill and Melinda Gates Foundation, USA to The INCLEN Trust International (grant number OPP1116433). The funder or its representative had no role in the design of the study and collection, analysis, and interpretation of data and writing the manuscript.

Authors' contributions:

Study conceptualisation, study design, protocol development, training, data analysis, interpretation: MKD and NKA.

Study coordination, monitoring, and data cleaning: MKD, BG, and AS.

Data analysis, interpretation, and manuscript preparation: MKD, BG and SS-1.

Protocol development, quality assurance and monitoring: AR

Participant recruitment and data collection: AW, BRV, JIB, JKG, JM, KK, LB, LS, AMK, NM, PKJ, RS-1, RS-2, SKD, SJ, SKR, SS-2, VK, NB, RG, APD, AG, CJS, GNM, HT, JS, KL, KR, ML, PP, RK, RS-3, SKA, SKS, SKG, SM, AD, BAC, BBT, RPG, HKS, JK, NRS, PA, SSGM, and SG.

For peer review only - http://bmjopen.bmj.com/site/about/guidelines.xhtml

652 All authors reviewed, provided critical input and approved the final version.

For peer review only

For peer review only - http://bmjopen.bmj.com/site/about/guidelines.xhtml

List of abbreviations

CI	Confidence interval
ICD	International Classification of Diseases
IQR	Interquartile range
RVV	Rotavirus vaccine
UIP	Universal immunization programme

For peer review only

References

1. Bines JE, Kohl KS, Forster J, Zanardi LR, Davis RL, Hansen J, et al. Acute intussusception in infants and children as an adverse event following immunization: case definition and guidelines of data collection, analysis, and presentation. Vaccine. 2004 Jan;22(5–6):569–74.
2. Bines JE, Ivanoff B. Acute intussusception in infants and children: Incidence, clinical presentation and management: a global perspective [Internet]. World Health Organisation; 2002 [cited 2017 May 10]. Available from: http://apps.who.int/iris/bitstream/handle/10665/67720/WHO_V-B_02.19_eng.pdf;jsessionid=74671C3CD99ED55137202F8D3D07CB73?sequence=1
3. Jiang J, Jiang B, Parashar U, Nguyen T, Bines J, Patel MM. Childhood Intussusception: A Literature Review. Cameron DW, editor. PLoS ONE. 2013 Jul 22;8(7):e68482.
4. Bahl R, Saxena M, Bhandari N, Taneja S, Mathur M, Parashar UD, et al. Population-Based Incidence of Intussusception and a Case-Control Study to Examine the Association of Intussusception with Natural Rotavirus Infection among Indian Children. The Journal of Infectious Diseases. 2009 Nov;200(s1):S277–81.
5. Jehangir S, John J, Rajkumar S, Mani B, Srinivasan R, Kang G. Intussusception in southern India: Comparison of retrospective analysis and active surveillance. Vaccine. 2014 Aug;32:A99–103.
6. Jain S, Haydel MJ. Child Intussusception. In: StatPearls [Internet]. Treasure Island (FL): StatPearls Publishing; 2020 [cited 2020 Jul 19]. Available from: http://www.ncbi.nlm.nih.gov/books/NBK431078/
7. Das MK, Arora NK, Gupta B, Sharan A, Kameswari K, Padmalatha P, et al. Intussusception in children aged under two years in India: Retrospective surveillance at nineteen tertiary care hospitals. Vaccine. 2020 Jun;S0264410X20305636.

For peer review only

For peer review only - http://bmjopen.bmj.com/site/about/guidelines.xhtml

8. Singh JV, Kamath V, Shetty R, Kumar V, Prasad R, Saluja T, et al. Retrospective surveillance for intussusception in children aged less than five years at two tertiary care centers in India. Vaccine. 2014 Aug;32:A95–8.

9. Bhowmick K, Kang G, Bose A, Chacko J, Boudville I, Datta SK, et al. Retrospective surveillance for intussusception in children aged less than five years in a South Indian tertiary-care hospital. J Health Popul Nutr. 2009 Oct;27(5):660–5.

10. Srinivasan R, Girish Kumar CP, Naaraayan SA, Jehangir S, Thangaraj JWV, Venkatasubramanian S, et al. Intussusception hospitalizations before rotavirus vaccine introduction: Retrospective data from two referral hospitals in Tamil Nadu, India. Vaccine. 2018 Dec;36(51):7820–5.

11. Gupta M, Kanojia R, Singha R, Tripathy JP, Mahajan K, Saxena A, et al. Intussusception Rate Among Under-Five-Children Before Introduction of Rotavirus Vaccine in North India. Journal of Tropical Pediatrics. 2018 Aug 1;64(4):326–35.

12. Guo W, Zhang S, Li J, Wang J. Association of Meteorological Factors with Pediatric Intussusception in Subtropical China: A 5-Year Analysis. Wang L, editor. PLoS ONE. 2014 Feb 28;9(2):e90521.

13. Gadisa A, Tadesse A, Hailemariam B. Patterns and seasonal variation of intussusception in children: a retrospective analysis of cases operated in a tertiary hospital in Ethiopia. Ethiop Med J. 2016 Jan;54(1):9–15.

14. Chen SC-C, Wang J-D, Hsu H-Y, Leong M-M, Tok T-S, Chin Y-Y. Epidemiology of childhood intussusception and determinants of recurrence and operation: analysis of national health insurance data between 1998 and 2007 in Taiwan. Pediatr Neonatol. 2010 Oct;51(5):285–91.

For peer review only

For peer review only - http://bmjopen.bmj.com/site/about/guidelines.xhtml

15. Moore SW, Kirsten M, Müller EW, Numanoglu A, Chitnis M, Le Grange E, et al. Retrospective surveillance of intussusception in South Africa, 1998-2003. J Infect Dis. 2010 Sep 1;202 Suppl:S156-161.

16. Greenberg D, Givon-Lavi N, Newman N, Wheeler J, Cohen Z, Dagan R. Intussusception in Children in Southern Israel: Disparity between 2 Populations. The Pediatric Infectious Disease Journal. 2008 Mar;27(3):236–40.

17. Sáez-Llorens X, Velázquez FR, Lopez P, Espinoza F, Linhares AC, Abate H, et al. A multi-country study of intussusception in children under 2 years of age in Latin America: analysis of prospective surveillance data. BMC Gastroenterol. 2013 May 27;13:95.

18. Buettcher M, Baer G, Bonhoeffer J, Schaad UB, Heininger U. Three-year surveillance of intussusception in children in Switzerland. Pediatrics. 2007 Sep;120(3):473–80.

19. Serayssol C, Abbo O, Mouttalib S, Claudet I, Labarre D, Galinier P, et al. [Seasonal pattern of intussusceptions in infants and children: is fall/winter predominance still worth consideration? A 10-year retrospective epidemiological study]. Arch Pediatr. 2014 May;21(5):476–82.

20. Chen YE, Beasley S, Grimwood K, and the New Zealand Rotavirus Study Group. Intussusception and rotavirus associated hospitalisation in New Zealand. Archives of Disease in Childhood. 2005 Jun 7;90(10):1077–81.

21. Boudville IC, Phua KB, Quak SH, Lee BW, Han HH, Verstraeten T, et al. The epidemiology of paediatric intussusception in Singapore: 1997 to 2004. Ann Acad Med Singap. 2006 Oct;35(10):674–9.

22. Liu N, Yen C, Huang T, Cui P, Tate JE, Jiang B, et al. Incidence and epidemiology of intussusception among children under 2 years of age in Chenzhou and Kaifeng, China, 2009-2013. Vaccine. 2018 14;36(51):7862–7.

For peer review only

For peer review only - http://bmjopen.bmj.com/site/about/guidelines.xhtml

23. Chang HG, Smith PF, Ackelsberg J, Morse DL, Glass RI. Intussusception, rotavirus diarrhea, and rotavirus vaccine use among children in New York state. Pediatrics. 2001 Jul;108(1):54–60.

24. Guo W, Geng J, Zhan Y, Tan Y, Hu Z, Pan P, et al. Forecasting and predicting intussusception in children younger than 48 months in Suzhou using a seasonal autoregressive integrated moving average model. BMJ Open. 2019 Jan;9(1):e024712.

25. Das M, Arora N, Bonhoeffer J, Zuber P, Maure C. Intussusception in Young Children: Protocol for Multisite Hospital Sentinel Surveillance in India. Methods and Protocols. 2018 Mar 22;1(2):11.

26. Nelson EAS, Tam JS, Glass RI, Parashar UD, Fok TF. Incidence of rotavirus diarrhoea and intussusception in Hong Kong using standardized hospital discharge data. The Pediatric Infectious Disease Journal. 2002 Jul;21(7):701–3.

27. Jo S, Lim IS, Chae SA, Yun SW, Lee NM, Kim SY, et al. Characteristics of intussusception among children in Korea: a nationwide epidemiological study. BMC Pediatr. 2019 Dec;19(1):211.

28. Giri S, Nair NP, Mathew A, Manohar B, Simon A, Singh T, et al. Rotavirus gastroenteritis in Indian children < 5 years hospitalized for diarrhoea, 2012 to 2016. BMC Public Health. 2019 Dec;19(1):69.

29. Parashar UD, Holman RC, Cummings KC, Staggs NW, Curns AT, Zimmerman CM, et al. Trends in Intussusception-Associated Hospitalizations and Deaths Among US Infants. Pediatrics. 2000 Dec 1;106(6):1413–21.

30. Desai R, Curns AT, Patel MM, Parashar UD. Trends in intussusception-associated deaths among US infants from 1979-2007. J Pediatr. 2012 Mar;160(3):456–60.

31. Justice FA, Auldist AW, Bines JE. Intussusception: trends in clinical presentation and management. J Gastroenterol Hepatol. 2006 May;21(5):842–6.

For peer review only

32. Jangra B, Jangra M, Rattan K, Kadian Y. Seasonal and day of week variations in acute appendicitis in north Indian children. J Indian Assoc Pediatr Surg. 2013;18(1):42.

33. Ahmed W, Akhtar MS, Khan S. Seasonal variation of acute appendicitis. Pak J Med Sci [Internet]. 2018 May 24 [cited 2020 Aug 4];34(3). Available from: http://pjms.com.pk/index.php/pjms/article/view/14793

34. Al-Mendalawi M, Lattuf M. Is there a seasonal distribution of acute appendicitis in Iraqi children? Menoufia Med J. 2017;30(2):572.

35. Zhang Y, Lyu F, Kang Q, Dong Z, Zhao T, Xie S, et al. Association of meteorological factors with pediatric acute appendicitis in China: A 7-year retrospective analysis. Medicine. 2018 Oct;97(42):e12913.

36. Hsu Y-J, Fu Y-W, Chin T. Seasonal variations in the occurrence of acute appendicitis and their relationship with the presence of fecaliths in children. BMC Pediatr. 2019 Dec;19(1):443.

37. Gallerani M, Boari B, Anania G, Cavallesco G, Manfredini R. Seasonal variation in onset of acute appendicitis. Clin Ter. 2006 Apr;157(2):123–7.

38. Reinisch A, Heil J, Woeste G, Bechstein W, Liese J. The meteorological influence on seasonal alterations in the course of acute appendicitis. Journal of Surgical Research. 2017 Sep;217:137–43.

39. Fares A. Summer appendicitis. Ann Med Health Sci Res. 2014;4(1):18.

40. Ferris M, Quan S, Kaplan BS, Molodecky N, Ball CG, Chernoff GW, et al. The Global Incidence of Appendicitis: A Systematic Review of Population-based Studies. Ann Surg. 2017;266(2):237–41.

41. Atkinson G, Gay B, Naffis D. Intussusception of the appendix in children. Am J Roentgenol. 1976 Jun;126(6):1164–8.

For peer review only

For peer review only - http://bmjopen.bmj.com/site/about/guidelines.xhtml

780 42. Ozkisacik SK, Erdem AO, Coskun O, Yazici M. Small bowel intussusception together with appendicitis in childhood: A case report. J Pediatr Surg Case Rep. 2015 Jan;3(1):25–6.

782 43. Marjon L, Hull N, Thomas K. Concurrent acute appendicitis and ileocolic intussusception in a 1-year-old child. Radiol Case Rep. 2018 Jun;13(3):655–7.

For peer review only

For peer review only - http://bmjopen.bmj.com/site/about/guidelines.xhtml

Tables

Table 1: Distribution and demography of the children with intussusception in India

Pooled	Years								
Years	2010[a]	2011	2012	2013	2014	2015	2016	2017[b]	Total
Number of cases, n	68	209	244	260	278	365	429	308	2161
Gender									
Male, n (%)	45 (66.2)	142 (67.9)	155 (63.5)	171 (65.8)	194 (69.8)	243 (66.6)	283 (66)	198 (64.3)	1431 (66.2)
Female, n (%)	23 (33.8)	67 (32.1)	89 (36.5)	89 (34.2)	84 (30.2)	122 (33.4)	146 (34)	110 (35.7)	730 (33.8)
Age groups									
2-6 months, n (%)	24 (35.3)	73 (34.9)	89 (36.5)	86 (33.1)	91 (32.7)	134 (36.7)	159 (37.1)	122 (39.6)	778 (36)
7-12 months, n (%)	33 (48.5)	92 (44)	108 (44.3)	122 (46.9)	113 (40.6)	135 (37)	155 (36.1)	117 (38)	875 (40.5)
13-24 months, n (%)	11 (16.2)	44 (21.1)	47 (19.3)	52 (20)	74 (26.6)	96 (26.3)	115 (26.8)	69 (22.4)	508 (23.5)
Regions									
North, n (%)	9 (13.2)	19 (9.1)	37 (15.2)	44 (16.9)	48 (17.3)	61 (16.7)	91 (21.2)	61 (19.8)	370 (17.1)
South, n (%)	50 (73.5)	134 (64.1)	130 (53.2)	149 (57.3)	124 (44.6)	175 (47.9)	183 (42.7)	136 (44.2)	1081 (50)
East, n (%)	9 (13.2)	45 (21.5)	61 (25)	54 (20.8)	84 (30.2)	112 (30.7)	131 (30.5)	97 (31.5)	593 (27.4)
West, n (%)	0 (0)	11 (5.3)	16 (6.6)	13 (5)	22 (7.9)	17 (4.7)	24 (5.6)	14 (4.5)	117 (5.4)

Note: a: The period of data collection was 6 months (July to December)
b: The period of data collection was 9 months (January to September)

For peer review only

Table 2. Pearson correlation analysis between monthly intussusception cases and monthly mean meteorological parameters

Parameter	Pearson correlation coefficient (r) for meteorological parameters				
	Temperature (°C)	Rainfall (mm)	Wind speed (m/s)	Humidity (%)	Sunshine (hr)
Pooled (n= 2161)	.056*	-.025	.134**	.075**	.045
Regions					
North (n= 370)	.018	-.103**	-.119**	-.373**	.163**
South (n= 1081)	.022	-.023	.210**	.161**	-.018
East (n= 593)	.052*	.072**	-.084**	.109**	.024
West (n= (117)	.046	-.019	.107**	-.029	-.012
Age groups					
2-6 months (n= 778)	.025	-.022	.092**	.040	.037
7-12 months (n= 875)	.068**	-.012	.115**	.069**	.042
13-24 months (n= 508)	.040	-.030	.125**	.076**	.029

Note: Pearson correlation coefficient (r); Significance two tailed

** Significance level- p value <0.05; ** Significance level- p value <0.01*

For peer review only

For peer review only - http://bmjopen.bmj.com/site/about/guidelines.xhtml

795 Table 3. Spearman's rank-order correlation analysis between monthly intussusception cases
796 and monthly mean meteorological parameters
797

Parameter	Spearman's rank-order correlation coefficient (ρ) for meteorological parameters				
	Temperature (°C)	Rainfall (mm)	Wind speed (m/s)	Humidity (%)	Sunshine (hr)
Pooled (n= 2161)	.049*	.012	.096**	.005	.051*
Regions					
North (n= 370)	.110*	-.014	.183**	-.114*	.119*
South (n= 1081)	.028	-.031	-.024	-.027	.036
East (n= 593)	.061	-.042	.109**	-.102*	.038
West (n= (117)	.052	-.044	.000	-.067	.076
Age groups					
2-6 months (n= 778)	.035	.031	.063**	.007	.044
7-12 months (n= 875)	.064**	.036	.103**	.049*	.048*
13-24 months (n= 508)	.028	.019	.106**	.043	.016

798 *Note: Spearman's rank-order correlation coefficient (ρ); Significance two tailed*
799 ** Significance level- p value <0.05; ** Significance level- p value <0.01*

For peer review only

Table 4: Regression analysis of the relationship between the monthly intussusception cases and monthly mean meteorological parameters

Variables	Regression coefficient, β (95% CI)	Standard error, $S_β$	χ2 - value	p value
Pooled[#]				
Temperature (^{0}C)	0.026 (0.011, 0.041)	0.008	12.098	<0.01
Rainfall (mm)	-0.001 (-0.003, 0)	0.001	2.462	0.117
Wind Speed (Kmph)	0.082 (0.061, 0.103)	0.011	58.449	<0.01
Humidity (%)	0.011 (0.007, 0.016)	0.002	21.903	<0.01
Sunshine (hr)	0.002 (0, 0.003)	0.001	7.000	<0.01
According to regions				
North region[#]				
Temperature (^{0}C)	0.027 (0.007, 0.047)	0.0102	7.195	<0.01
Rainfall (mm)	0.001 (-0.005, 0.007)	0.0029	0.169	0.681
Wind Speed (Kmph)	0.208 (0.101, 0.315)	0.0545	14.556	<0.01
Humidity (%)	-0.010 (-0.020, 0.001)	0.0052	3.448	0.063
Sunshine (hr)	0.004 (0.002, 0.007)	0.0013	11.006	<0.01
South region[#]				
Temperature (^{0}C)	0.010 (-0.037, 0.058)	0.0243	0.174	0.676
Rainfall (mm)	-0.004 (-0.009, 0.000)	0.0024	3.251	0.071
Wind Speed (Kmph)	-0.011 (-0.045, 0.023)	0.0173	0.401	0.527
Humidity (%)	-0.033 (-0.070, 0.005)	0.0190	2.949	0.086
Sunshine (hr)	0.002 (-0.001, 0.004)	0.0014	1.213	0.271
East region[#]				
Temperature (^{0}C)	0.029 (0.001, 0.057)	0.0141	4.245	0.039
Rainfall (mm)	-0.001 (-0.004, 0.001)	0.0014	0.766	0.381
Wind Speed (Kmph)	0.091 (0.042, 0.141)	0.0251	13.244	<0.01
Humidity (%)	-0.021 (-0.035, -0.007)	0.0070	9.188	<0.01
Sunshine (hr)	0.002 (-6.100, 0.005)	0.0012	3.822	0.051
*West region**				
Temperature (^{0}C)	0.052 (-0.02, 0.124)	1.987	0.036	0.159
Rainfall (mm)	-0.002 (-0.006, 0.002)	0.855	0.002	0.355
Wind Speed (Kmph)	0.022 (-0.042, 0.087)	0.463	0.032	0.496
Humidity (%)	-0.002 (-0.016, 0.011)	0.105	0.006	0.746
Sunshine (hr)	0.003 (-0.001, 0.007)	2.429	0.002	0.119
According to age groups				
*2-6 months**				
Temperature (^{0}C)	0.012 (-0.004, 0.028)	.008	2.192	0.139
Rainfall (mm)	-0.001 (-0.003, 0.001)	.001	1.599	0.206
Wind Speed (Kmph)	0.059 (0.037, 0.081)	.011	28.241	<0.01
Humidity (%)	0.006 (0.001, 0.011)	.003	5.276	0.022
Sunshine (hr)	0.002 (0.000, 0.003)	.001	4.615	0.032
7-12 months[#]				
Temperature (^{0}C)	0.038 (0.018, 0.057)	.010	14.288	<0.01
Rainfall (mm)	-0.001 (-0.003, 0.002)	.001	.447	0.504
Wind Speed (Kmph)	0.08 (0.054, 0.107)	.013	36.037	<0.01

For peer review only

Humidity (%)	0.012 (0.006, 0.018)	.003	14.660	<0.01
Sunshine (hr)	0.002 (0.000, 0.004)	.001	5.144	0.023
*13-24 months**				
Temperature (^{0}C)	0.026 (0.006, 0.047)	.010	6.460	0.011
Rainfall (mm)	-0.002 (-0.005, 0.000)	.001	3.564	0.059
Wind Speed (Kmph)	0.102 (0.077, 0.128)	.013	60.620	<0.01
Humidity (%)	0.017 (0.010, 0.024)	.004	22.599	<0.01
Sunshine (hr)	0.002 (0.000, 0.004)	.001	3.211	0.073

*Note: Tests used for regression analysis: *Poisson Regression; # Negative Binomial Regression*

For peer review only

For peer review only - http://bmjopen.bmj.com/site/about/guidelines.xhtml

804 **Figure**

805 Figure 1: Monthly distribution of intussusception cases in children during 2010-2017

For peer review only

For peer review only - http://bmjopen.bmj.com/site/about/guidelines.xhtml

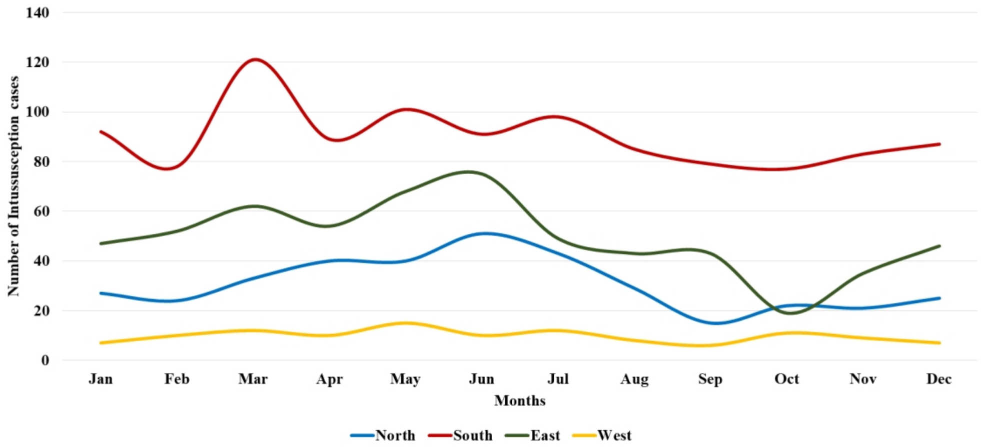

Figure 1: Monthly distribution of primary intussusception cases in children during 2010-2017

155x71mm (300 x 300 DPI)

Title: Association of meteorological parameters with intussusception in children aged under-two years: Results from a multisite bidirectional surveillance over seven years in India

Supplementary Documents

Index

Sl no	Details
1	Supplementary Figure 1: The study sites and their location on India map indicating the study regions
2	Supplementary Table 1: The International Classification of Diseases (ICD) codes for review of the cases from medical records
3	Supplementary Table 2: The seasons and months at the sites according to the regions
4	Supplementary Table 3: The monthly median (and IQR) meteorological data during 2010 and 2017 for regions
5	Supplementary Table 4: Monthly median (and IQR) meteorological data during 2010 and 2017 at the study sites
6	Supplementary Figure 2: The pattern of monthly meteorological parameters across the regions
7	Supplementary Figure 3: The pooled monthly (SF3a) and seasonal (SF3b) distribution of intussusception in children during July 2010 to September 2017
8	Supplementary Table 5. Pearson correlation analysis between monthly intussusception cases and monthly median meteorological parameters for the study sites
9	Supplementary Table 6. Spearman's Rank correlation analysis between monthly intussusception cases and monthly median meteorological parameters for the study sites
10	Supplementary Table 7. Regression analysis of the association between the intussusception cases and meteorological parameters for the study sites
11	Supplementary Table 8. Intussusception cases in children at different temperature ranges at pooled and regional level
12	Supplementary Table 9. Variations in the intussusception cases between the different temperature categories by Kruskal-Wallis H test
13	Supplementary Figure 4: Association of monthly intussusception cases with mean monthly temperatures
14	Supplementary Figure 5: Association of monthly intussusception cases with mean monthly humidity
15	Supplementary Figure 6: Association of monthly intussusception cases with mean monthly wind speed
16	Supplementary Figure 7: Association of monthly intussusception cases with mean monthly sunshine
17	Supplementary Figure 8: Association of monthly intussusception cases with mean monthly rainfall
18	Supplementary Table 10. Factorial analysis-of-variance of intussusception in children with season and age groups
19	Supplementary Table 11. Factorial analysis-of-variance of intussusception in children with season and regions

For peer review only

20	Supplementary Table 12. Multiple comparisons of seasonal intussusception in children in different seasons pooled for all sites
21	Supplementary Table 13. Multiple comparisons of seasonal intussusception in children in different seasons at regional level
22	Supplementary Table 14. Multiple comparisons of seasonal intussusception in children in different seasons for different age groups

For peer review only

Supplementary Figure 1: The study sites and their location on India map indicating the study regions

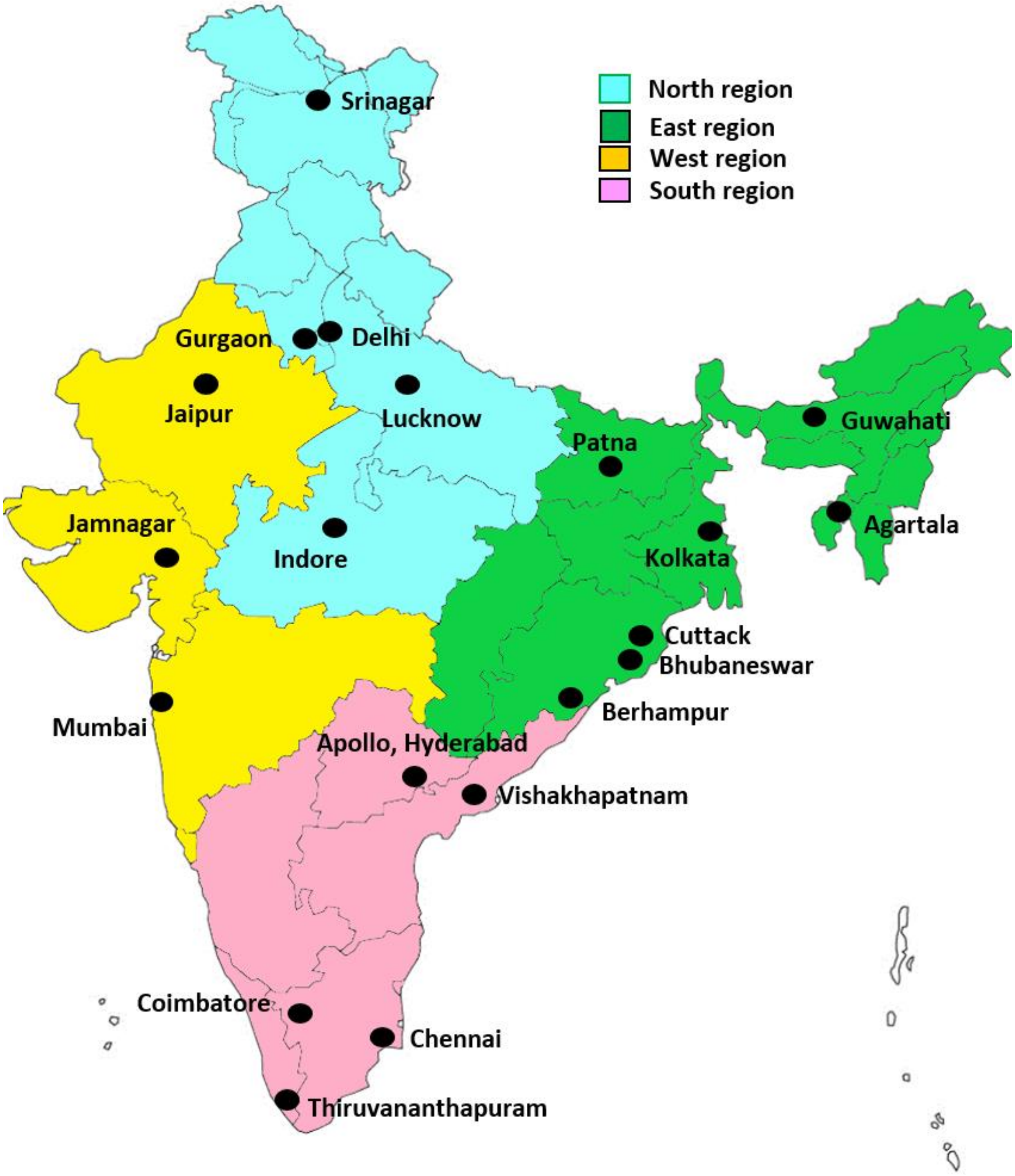

Note: The map has been prepared for use in the research project. The corresponding author is the creator of the image.

Supplementary Table 1: The International Classification of Diseases (ICD) codes for review of the cases from medical records

Clinical conditions considered as suspected cases	Codes	
	ICD 10	ICD 9
Intussusception	K56.1	560.0
Volvulus	K56.2	560.2
Gallstone ileus	K56.3	560.31
Other impaction of intestine	K56.4	560.30
Intestinal adhesions with obstruction	K56.5	560.81
Other and unspecified intestinal obstruction or<br>Acute intestinal obstruction or<br>Subacute intestinal obstruction	K56.6	560.9
Ileus, unspecified	K56.7	560.1
Paralytic ileus	K56.0	
Acute abdomen	R10.0	789.0
Blood in stool with vomiting	K92.1/K92.2 with R11.2	578.1/578.9 with 787.01

For peer review only - http://bmjopen.bmj.com/site/about/guidelines.xhtml

Supplementary Table 2: The seasons and months at the sites according to the regions

Region/ Site (state)	Jan	Feb	Mar	Apr	May	Jun	Jul	Aug	Sep	Oct	Nov	Dec
North region												
Srinagar (Jammu & Kashmir)	W	W	W	Sp	Sp	Su	Su	M	M	A	W	W
New Delhi (Delhi)	W	Sp	Sp	Su	Su	Su	M	M	M	A	A	W
Gurgaon (Haryana)	W	Sp	Sp	Su	Su	Su	M	M	M	A	A	W
Lucknow (Uttar Pradesh)	W	W	Sp	Su	Su	Su	M	M	M	A	A	W
Indore (Madhya Pradesh)	W	W	Sp	Su	Su	Su	M	M	M	A	A	W
South region												
Vishakhapatnam (Andhra Pradesh)	W	W	Su	Su	Su	M	M	M	M	A	A	W
Hyderabad (Telangana)	W	Sp	Su	Su	Su	M	M	M	M	A	A	W
Coimbatore (Tamil Nadu)	W	Sp	Su	Su	Su	M	M	M	M	A	A	W
Chennai (Tamil Nadu)	W	Sp	Su	Su	Su	M	M	M	M	A	A	W
Trivandrum (Kerala)	W	Sp	Su	Su	Su	M	M	M	M	A	A	W
East region												
Patna (Bihar)	W	Sp	Sp	Su	Su	Su	M	M	M	A	A	W
Bhubaneswar (Odisha)	W	Sp	Su	Su	Su	M	M	M	A	A	A	W
Cuttack (Odisha)	W	Sp	Su	Su	Su	M	M	M	A	A	A	W
Berhampur (Odisha)	W	Sp	Su	Su	Su	M	M	M	A	A	A	W
Kolkata (West Bengal)	W	Sp	Su	Su	Su	M	M	M	A	A	A	W
Guwahati (Assam)	W	W	Su	Su	Su	M	M	M	A	A	A	W
Agartala (Tripura)	W	W	Su	Su	Su	M	M	M	A	A	A	W
West region												
Jaipur (Rajasthan)	W	W	Sp	Su	Su	Su	M	M	M	A	A	W
Jamnagar (Gujarat)	W	Sp	Su	Su	Su	M	M	M	M	A	A	W
Mumbai (Maharashtra)	W	Sp	Su	Su	Su	M	M	M	M	A	A	W

Note: The seasons and colour codes used

A	Autumn	M	Monsoon	Sp	Spring	Su	Summer	W	Winter

For peer review only

Supplementary Table 3: The monthly median (and IQR) meteorological data during 2010 and 2017 for regions

Region/ Months	Temperature (°C)	Rainfall (mm)	Wind speed (Kmph)	Humidity (%)	Sunshine (hours)
North region					
January	14 (11, 16)	5.7 (0.5, 13.2)	8.5 (7.1, 9.8)	54 (47, 61)	223 (217.5, 232.5)
February	17 (14, 20)	6.9 (0.5, 22.6)	9.6 (8.1, 10.3)	47 (39, 62)	266 (239, 277.5)
March	23 (21, 25)	4.9 (1.1, 19.9)	9.9 (9, 10.5)	37 (26, 45)	301 (291.5, 308.5)
April	31 (28, 32)	3.9 (1.3, 12.6)	10.5 (8.1, 12.2)	23 (18, 28)	337.5 (315, 345)
May	35 (34, 36)	3.3 (1.1, 14.3)	10.7 (8.9, 12.3)	22 (19, 30)	386 (383, 387.5)
June	35 (31, 37)	22 (8.1, 48.8)	11.1 (9.1, 12.9)	40 (31, 46)	361.5 (346.5, 369.5)
July	31 (26, 33)	72.5 (30, 126.7)	9.6 (8, 12.3)	59 (54, 69)	331.8 (279.3, 367)
August	29 (24, 31)	55.1 (26.3, 93.7)	9 (7, 10.8)	66 (56, 80)	337.8 (298.8, 359.3)
September	28 (25, 30)	31.6 (13.4, 58.1)	8.4 (7.2, 9.2)	56 (49, 76)	286.3 (269.8, 300)
October	26 (25, 27)	1.5 (0.2, 12.2)	6.5 (5.9, 7.2)	42 (34, 50)	250 (244, 262.5)
November	21 (20, 22)	0.1 (0, 3.2)	7.5 (6, 8.2)	33 (31, 39)	225 (222, 225)
December	16 (14, 18)	0.6 (0, 4.4)	7.8 (6.7, 8.8)	39 (35, 45)	231 (225.5, 232.5)
South region					
January	23 (23, 25)	2.8 (1.1, 7.7)	9.7 (8.6, 12.1)	67 (63, 71)	277.5 (274.5, 296.5)
February	25 (24, 26)	1.5 (0.4, 8)	10.1 (8.8, 12.2)	65 (51, 71)	277.5 (272.5, 286)
March	28 (27, 28)	10.1 (3.4, 29.8)	10.9 (8.9, 13.6)	66 (50, 71)	299.5 (294, 308.5)
April	30 (29, 31)	7.8 (2.9, 32.2)	12.1 (9.7, 17.1)	69.5 (52, 73)	317.5 (292.5, 333.5)
May	31 (29, 32)	20.2 (9.1, 30.5)	14.6 (12.9, 17.6)	64 (59, 71)	353.5 (317, 378)
June	28 (27, 31)	50.7 (16.5, 97.8)	18 (17.2, 18.5)	65 (62, 70)	291 (268.5, 319.5)
July	28 (26, 29)	43 (1.7, 78.5)	18.5 (17, 20.1)	68 (63, 74)	283.3 (255.5, 308.5)
August	27 (26, 29)	58.1 (7.9, 92.3)	16.2 (14.4, 17.6)	72 (66, 77)	275.5 (245.5, 296.5)
September	27 (26, 29)	61.7 (34.1, 99.9)	13.8 (12.3, 16.3)	72 (67, 76)	258 (239, 288)
October	27 (26, 28)	61.9 (44.4, 117.6)	10.6 (9.4, 12.2)	71 (68, 75)	246.8 (224.5, 267)
November	25 (24, 27)	34.8 (19.2, 108.9)	9.6 (7.2, 13.5)	72 (69, 75)	205.5 (191, 213)
December	24 (23, 26)	15 (3, 37.9)	10 (8.5, 14.3)	70.5 (66, 74)	225.5 (216, 265.5)
East region					
January	20 (17, 22)	1.7 (0.1, 5.8)	7.2 (6.4, 8.3)	60 (54, 64)	227 (224.5, 231)
February	23 (20, 24)	2.7 (1, 8.7)	8.2 (7, 9)	54 (49, 61)	210 (207, 237)
March	27 (25, 28)	8.5 (2.9, 19.4)	10 (8.9, 11)	56 (46, 60)	291.5 (287.5, 301)
April	31 (29, 32)	12.3 (3.5, 32.1)	12.5 (10.4, 14.6)	60 (54, 66)	355.5 (294.5, 370.5)
May	32 (30, 33)	25.2 (13.9, 54.5)	13.3 (11.2, 15)	66 (60, 69)	365.5 (347, 374.5)
June	32 (29, 33)	79.8 (53.9, 122.3)	12 (10.6, 13.1)	66 (62, 76)	307.5 (292.5, 327)
July	30 (28, 30)	118 (82.6, 175.2)	11.3 (9.8, 12.4)	73 (69.5, 83.5)	276.8 (250, 288.5)
August	29 (28, 30)	100 (70.8, 154.9)	10 (8.9, 11.1)	78 (73.5, 82)	286 (258, 305)
September	28 (28, 29)	89.9 (65.7, 133)	8.3 (7.3, 9.4)	79.5 (75, 83)	252.5 (236.3, 262)
October	26 (25, 27)	44.7 (24.8, 87.9)	6.7 (5.9, 7.6)	77 (71, 82)	287 (271.5, 295)
November	23 (21, 24)	3.6 (0.4, 20.4)	6.4 (5.7, 7.3)	66 (60, 77)	270 (256, 292.5)
December	21 (18, 22)	1 (0.2, 6.7)	7.1 (6.1, 8)	59 (50, 70)	229.5 (222.5, 240)
West region					
January	22 (15, 24)	0.1 (0, 0.9)	10 (8.5, 14.4)	48 (44, 51)	310 (255, 310)
February	24 (20, 25)	0.4 (0, 1)	10.8 (9.9, 14.5)	45 (44, 53)	278.5 (275.5, 281)
March	27 (25, 28)	0.2 (0, 1.2)	10.7 (10.1, 15.9)	47 (29, 53)	307.5 (305.5, 308.5)
April	29 (29, 31)	0.4 (0, 1.7)	11.2 (10, 18.3)	56 (20, 61)	300 (300, 315.5)

For peer review only - http://bmjopen.bmj.com/site/about/guidelines.xhtml

Region/ Months	Temperature (°C)	Rainfall (mm)	Wind speed (Kmph)	Humidity (%)	Sunshine (hours)
May	31 (30, 36)	0.6 (0.1, 5.5)	14.3 (10.7, 23.9)	65 (21, 68)	355 (307, 381.5)
June	31 (29, 36)	48.9 (9.7, 156.7)	16.7 (13.9, 21.7)	68 (34, 75)	311.5 (252, 359.5)
July	29 (28, 32)	121.3 (58.1, 391.4)	17.3 (13.9, 23.8)	77.5 (56.5, 82.5)	194 (158.5, 338.8)
August	28 (27, 29)	118.5 (60, 213.5)	14.1 (11.4, 22.1)	79 (73.5, 84)	196.8 (166.5, 278)
September	28 (27, 29)	70.6 (19.4, 133.8)	11.6 (9.8, 17.8)	74 (62.5, 80)	253.5 (229, 274.8)
October	28 (28, 29)	5.9 (0.5, 26.1)	9 (8.1, 12.5)	51 (41, 67)	296.5 (265, 308.5)
November	27 (23, 28)	0.1 (0, 2.3)	9 (8, 13)	44 (34, 53)	230 (225, 251.5)
December	24 (17, 25)	0 (0, 0.5)	9.8 (8.4, 14.5)	43 (39, 49)	252.5 (232.5, 293.5)

Note: Temperature (°C); Rain: Rainfall (millimetre, mm); Wind Speed (miles/second); Humidity (%); Sunshine (in hours/month)

For peer review only

Supplementary Table 4: Monthly median (and IQR) meteorological data during 2010 and 2017 at the study sites

Region/ Place (State) and months	Temperature (°C)	Rainfall (mm)	Wind speed (Kmph)	Humidity (%)	Sunshine (hour)
1. North region					
1.1 Srinagar (Jammu and Kashmir)					
January	-3 (-4,-1)	28.4 (16.8,41.9)	5.8 (5.3,6.3)	58 (44,69)	209.5 (205.5,218)
February	0 (-2,2)	69.9 (56.8,89)	5.7 (5.4,6.1)	60 (59,68)	241 (220,261.5)
March	4 (3,5)	63.4 (44.2,107)	5.3 (4.7,6.5)	60 (57,63)	282 (232.5,296)
April	8 (7,9)	45.3 (36.7,84.2)	4.6 (4.4,5)	55 (52,57)	330 (301.5,336)
May	13 (11,14)	31.6 (16.3,57.3)	4.3 (3.7,5.1)	49 (46,50)	387.5 (378.5,387.5)
June	17 (16,18)	15.8 (8.1,33.4)	4.2 (3.8,4.4)	46 (41,47)	372 (364.5,375)
July	19 (18.5,19)	22.6 (13,50.5)	5.4 (4.7,6)	57.5 (57,59)	387.5 (381.5,387.5)
August	18 (18,19)	25 (20.6,34.1)	5 (3.8,5.4)	59.5 (56,65)	371 (368,372.5)
September	15 (14,15)	9 (5.6,48.8)	3.9 (3.6,4.1)	50.5 (48,52.5)	300 (297.8,300)
October	9 (8,11)	10.5 (4.1,15.8)	3.3 (2.8,3.9)	40 (34,41)	262.5 (258.5,262.5)
November	4 (3,5)	8 (1.4,12.1)	4.6 (4.1,6)	33 (31,36)	222 (209.5,225)
December	0 (-1,1)	14.9 (5.3,24.9)	5.8 (5.4,6.7)	31 (27,42)	221.5 (211.5,228)
1.2 Delhi (Delhi) and 1.3. Gurgaon (Haryana)					
January	13 (11,14)	1 (0.3,5.7)	8.5 (7.9,9.2)	54 (53,62)	223 (220.5,228.5)
February	16 (15,18)	5.3 (0.2,7.8)	9.8 (8.9,10.1)	53 (43,63)	269.8 (266,277.5)
March	22 (21,23)	2.9 (1.1,6.5)	9.9 (9.2,10.4)	41 (33,43)	300 (292,303)
April	30 (29,32)	5.3 (2.7,9.8)	10.5 (8.4,10.7)	23 (17,27)	341.8 (331.5,344.5)
May	36 (35,36)	2.4 (0.9,6.7)	10.2 (9.8,10.7)	20.5 (15,22)	386 (383,386)
June	36 (36,38)	20.7 (8.5,30.9)	10.1 (9.8,11.8)	32 (23,36)	363.5 (361.5,369.5)
July	32.5 (32,34)	61.2 (30.7,98.2)	8.9 (8.1,10.2)	53.5 (51,61.5)	345 (331.8,365)
August	31 (30,32.5)	47.8 (28.5,78.9)	8.4 (7.7,9.7)	63.5 (54,72.5)	339.8 (329,349)
September	30.5 (29,31)	27.2 (11.9,61.1)	8.2 (7.7,8.6)	49.5 (47,67)	279.5 (272.3,294)
October	27 (26,28)	0.5 (0,1.1)	6.5 (6.2,7)	34 (31,43)	250 (248.5,250)
November	21 (21,22)	0 (0,0.2)	8.1 (6.5,8.2)	31 (27,40)	225 (223.5,225)
December	16 (15,17)	0.3 (0,1.5)	8.3 (6.9,9)	39 (35,46)	230.5 (225.5,231)
1.4. Lucknow (Uttar Pradesh)					
January	15 (14,16)	8.1 (4.7,9.6)	9.8 (8.6,10.1)	54 (53,58)	224 (219,229.5)
February	18 (17,20)	6.9 (0.5,22.6)	10.1 (9.8,11.3)	52 (42,58)	230.5 (224.5,239)
March	25 (24,25)	3.1 (2.6,10.5)	11.2 (11.1,12)	35 (26,37)	309.5 (303.5,315)
April	32 (32,34)	2 (1.3,3.9)	12.6 (12,13.4)	21 (18,26)	373.5 (363,375)
May	36 (36,37)	5.2 (0.2,6.9)	11.4 (11.2,12.3)	21 (18,25)	384.5 (380,386)
June	37 (34,39)	13.4 (3.2,100.6)	11.6 (11.2,12.9)	32 (25,45)	346.5 (321.5,362.5)
July	32 (31,33)	127.8 (83.6,151.1)	11.1 (10.2,12.3)	63 (57,69)	304.3 (289,321)
August	30 (30,31.5)	76.5 (50.8,95.9)	10.7 (9.4,11.1)	70.5 (58,74.5)	313.8 (304,344.8)
September	29.5 (28.5,30.5)	30.9 (21.1,61.8)	9.1 (8.5,9.6)	64.5 (55,75.5)	316 (301.3,328.3)
October	26 (25,27)	7.4 (0.2,31.3)	6.9 (6.1,7.9)	50 (42,62)	227.5 (225.5,238.5)
November	22 (21,22)	0 (0,0.7)	7.4 (6.9,8.5)	35 (34,48)	225 (218,225)
December	17 (17,18)	0.6 (0,1.6)	8.5 (7.6,9.9)	41 (38,46)	232.5 (231,232.5)
1.5 Indore (Madhya Pradesh)					
January	18 (17,19)	0.8 (0.4,9.1)	9.2 (8,10.5)	44 (32,47)	281 (277.5,282.5)
February	22 (21,23)	1 (0.2,11.1)	9.6 (8.6,11)	32 (29,39)	280 (277,290)
March	27 (27,27)	2.9 (0,6.4)	10 (9.8,10.4)	21 (18,26)	307 (301,310)
April	32 (32,33)	0.6 (0.2,2.8)	12.3 (11.2,14.4)	18 (17,21)	315 (310,317.5)
May	35 (34,35)	1.9 (0.6,2.7)	15.3 (14.7,16.8)	24 (22,30)	386 (384.5,387.5)
June	31 (31,32)	48.9 (29.6,72.2)	15.4 (14.6,16.9)	51 (47,57)	341 (294,352)

For peer review only - http://bmjopen.bmj.com/site/about/guidelines.xhtml

Region/ Place (State) and months	Temperature (°C)	Rainfall (mm)	Wind speed (Kmph)	Humidity (%)	Sunshine (hour)
July	27 (26,27)	124.5 (83.8,186.7)	15.5 (12.6,17.3)	80 (75,81.5)	201.5 (178.8,239)
August	25.5 (24.5,26)	101.7 (78.8,132.8)	12.1 (11.4,14.9)	88.5 (80,90)	166.5 (144.8,208.3)
September	25.5 (25,26)	52.2 (41,62.1)	9.7 (8.6,10.5)	77 (75,81)	253.5 (241.5,267.3)
October	26 (25,26)	2.3 (0.3,31.1)	7.2 (6.6,8.3)	45 (40,58)	271 (254.5,272.5)
November	23 (22,24)	0.3 (0,2.9)	7.6 (6.4,8)	37 (33,39)	225 (220.5,225)
December	19 (19,20)	0.1 (0,1.2)	8.1 (7.4,8.8)	36 (34,40)	232.5 (229.5,232.5)
2. East region					
2.1 Bhubaneswar and 2.2. Cuttack (Odisha)					
January	22 (21,22)	1.5 (0.1,5.6)	7.2 (6.4,7.4)	56.5 (56,60)	224.5 (221.5,228)
February	24.5 (24,25)	2 (0.5,8.9)	8.2 (7.1,9.3)	56 (53,62)	209.3 (202.5,210)
March	28 (28,29)	6.7 (3.6,16.6)	10.9 (9.4,11.5)	57.5 (54,62)	287.3 (275.5,290)
April	31 (31,32)	5.4 (1.6,12.3)	15 (12.4,17.2)	60.5 (57,66)	366.8 (360,372)
May	32 (32,33)	22.3 (15.4,26.9)	14.9 (13.9,15.8)	64.5 (63,67)	366.8 (362.5,375.5)
June	32 (32,33)	80 (60.3,114.7)	12.2 (12,13.7)	62.5 (62,66)	300.8 (292.5,322.5)
July	30 (30,30)	118 (80.3,137.4)	11.3 (10.1,12.1)	72 (69,73)	269.8 (253.3,283.8)
August	29 (29,30)	92.6 (67,133.3)	10.4 (9.2,11.4)	74.5 (71.5,77.5)	285 (262.3,296)
September	29 (28,29)	89.9 (67.3,112.9)	8.7 (7.3,9.8)	77 (74.5,79.5)	248.3 (234.3,257.3)
October	27 (26,27)	50.1 (25.7,89.9)	7.4 (6.7,7.9)	74 (69,82)	285.5 (263,290)
November	23.5 (23,25)	9.1 (1,28.3)	6.6 (6.2,7.2)	64.5 (60,66)	261.8 (256,268.5)
December	21 (21,22)	4.7 (0.3,11.8)	6.9 (6.6,8)	57 (53,59)	223.8 (207,229.5)
2.3 Berhampur (Odisha)					
January	22 (21,22)	1.6 (0.6,5.6)	6.9 (6.6,7.6)	66 (63,69)	224.5 (221.5,228)
February	24 (24,24)	3.6 (2.1,15.7)	8.2 (7.2,8.7)	62 (61,68)	210 (205.5,210)
March	28 (27,28)	8.5 (1.3,20.6)	10.8 (9.8,11.7)	61 (59,64)	288.5 (275.5,291)
April	30 (30,31)	9.7 (2,20.6)	13.9 (12.4,14.6)	63 (58,67)	367.5 (361.5,372)
May	32 (32,32)	13.3 (7.1,28.9)	13.3 (12.5,13.7)	67 (64,68)	368 (364,380)
June	31 (31,32)	60.7 (48.2,79.8)	11.3 (11.1,11.8)	67 (64,69)	307.5 (300,324.5)
July	29 (29,30)	82.9 (65.6,107.6)	11.1 (10.5,11.4)	73 (71,74.5)	283.5 (279.3,289)
August	29 (29,29)	93.5 (72.6,119.7)	9.4 (9.1,10)	74.5 (73,78)	294.3 (272,302.5)
September	28.5 (28,29)	91.1 (67,116)	8.1 (7.5,8.8)	76 (75,77.5)	254.8 (250.3,261.5)
October	27 (26,27)	44.9 (33.2,132.7)	8.1 (6.6,9)	72 (70,80)	289.5 (263,295)
November	24 (24,24)	44.5 (0.4,59.7)	7 (6.6,8.3)	67 (63,71)	262 (256,271.5)
December	22 (22,22)	3.3 (1,30.7)	7.8 (7,8.2)	62 (60,67)	225 (207,229.5)
2.4. Kolkata (West Bengal)					
January	20 (19,21)	0.1 (0,7.7)	9.1 (8.6,9.9)	44 (40,52)	228 (225,231)
February	23 (23,24)	4.4 (0.8,7.8)	9 (8.1,9.3)	45 (40,46)	211 (204.5,212.5)
March	28 (27,28)	8.7 (1.7,19.2)	10 (9.4,10.2)	50 (46,57)	304 (295,308.5)
April	31 (31,33)	12.5 (5.2,37.2)	14.2 (10.3,15.2)	58 (54,63)	300 (287.5,312)
May	33 (32,33)	16.2 (11.2,28.5)	15 (13.7,17.7)	61 (59,67)	371 (369.5,377.5)
June	32 (31,33)	63.7 (52.7,92.7)	13.1 (12.8,15.8)	65 (64,72)	318.5 (290.5,327)
July	30 (29.5,30)	119.7 (102.1,171.9)	12.6 (11.5,13.2)	76 (74,78)	258.8 (243.3,271.3)
August	29 (29,30)	102.7 (86,153.2)	11 (10.3,11.1)	80.5 (76,82)	277.3 (247.8,284.5)
September	28 (28,29.5)	81.4 (74.9,111.4)	9.1 (8.1,10.3)	81.5 (77,85)	236.3 (226.8,241.3)
October	27 (26,27)	27.2 (23.6,34.9)	7.3 (6.5,8.7)	72 (71,80)	280 (265.5,287.5)
November	24 (23,25)	3.3 (0.3,16.7)	7.5 (6.1,9.3)	57 (53,65)	292.5 (284,297)
December	21 (20,22)	0.1 (0,1.8)	8.7 (8.5,9.5)	47 (41,50)	230 (218,232.5)
2.5. Patna (Bihar)					
January	16 (15,17)	5.8 (0.8,8.3)	9.5 (9.4,10)	54 (51,63)	229.5 (222.5,232.5)
February	19 (18,21)	0.9 (0.4,2.7)	10.4 (9.7,11.8)	49 (47,54)	207.5 (202.5,213)
March	26 (25,26)	2.9 (1.6,5.5)	11.1 (10.1,11.7)	32 (30,33)	303.5 (291.5,305)

For peer review only - http://bmjopen.bmj.com/site/about/guidelines.xhtml

Region/ Place (State) and months	Temperature (°C)	Rainfall (mm)	Wind speed (Kmph)	Humidity (%)	Sunshine (hour)
April	33 (32,33)	6.5 (2.4,22.2)	12.3 (11.4,14.2)	28 (20,31)	373.5 (358.5,375)
May	35 (35,37)	23.2 (5,41.1)	13 (12.3,13.6)	35 (29,39)	377 (366.5,386)
June	36 (34,37)	47.6 (21.1,58.6)	11.9 (11.5,13.6)	42 (42,51)	337 (327,355)
July	32 (31,32.5)	150.4 (120.4,160.4)	12 (11.7,14)	65.5 (60.5,68.5)	283 (257.5,312.5)
August	31 (31,32)	63.4 (38.5,138.2)	10.6 (9.3,11.7)	65 (61.5,71.5)	318.5 (293,329.8)
September	29 (28.5,30.5)	66.7 (32.4,116.6)	8.7 (8.4,10.4)	67 (60.5,80)	273 (249.5,282.5)
October	26 (25,27)	29.1 (8.1,54.9)	6.8 (5.9,7.5)	63 (51,78)	297 (271.5,304)
November	22 (21,23)	0 (0,0.3)	6.8 (6.3,8.1)	45 (39,61)	232.5 (231,235)
December	18 (18,19)	0 (0,0.7)	8 (7.5,9.1)	49 (38,54)	231 (225,232.5)
2.6. Guwahati (Assam)					
January	17 (15,17)	3.9 (0.2,10.3)	3.9 (3.8,4.3)	67 (64,76)	228 (225,231)
February	20 (19,20)	4.9 (2.3,10.2)	4.2 (4,4.5)	56 (54,62)	245 (242,253.5)
March	24 (23,24)	14.1 (1.4,22.9)	4.9 (4.6,4.9)	46 (42,58)	299.5 (295,310)
April	26 (25,27)	39.7 (15.1,80.8)	4.5 (4.4,4.8)	62 (50,68)	292.5 (280.5,300)
May	27 (27,28)	83.1 (54.5,122.4)	4.2 (3.8,5)	69 (63,73)	344 (336.5,357)
June	28 (27,29)	236.2 (191.4,326.2)	3.8 (3.6,4.3)	81 (76,84)	323.5 (292.5,334)
July	27.5 (27,28)	344.4 (224.2,407.4)	3.8 (3.5,4.1)	87 (83.5,88.5)	317.8 (274.5,332.8)
August	28 (27.5,28)	235.2 (214.1,276.4)	3.8 (3.7,4.2)	84 (82,85)	297.3 (283,310.5)
September	27 (26.5,27)	159.3 (143.5,217.6)	3.4 (3.1,4)	84 (81.5,85)	272 (257.8,274.8)
October	24 (24,24)	48.8 (12.8,72.7)	3.6 (3.4,4.2)	79 (77,81)	294 (283,305.5)
November	20 (20,20)	4.5 (1,7.3)	3.8 (3.7,4.5)	78 (71,80)	294 (292,297)
December	17 (17,18)	2.5 (1.1,9)	4 (3.8,4.2)	74 (73,77)	250.5 (240,253.5)
2.7. Agartala (Tripura)					
January	18 (17,19)	0.3 (0.1,2.2)	6.7 (6.5,7.2)	61 (58,69)	231 (228,232.5)
February	21 (20,22)	5.6 (1.7,10.5)	7.2 (7,8.6)	53 (51,57)	246 (237,250.5)
March	25 (24,26)	22.6 (11.8,53.2)	8.9 (8.8,9.5)	57 (51,64)	295.5 (288,301)
April	28 (28,29)	113.4 (38.1,141.2)	11.1 (8.5,12.5)	67 (64,73)	266.5 (246,297)
May	29 (28,30)	113.8 (73.4,194.5)	9.7 (9.1,11.5)	74 (74,80)	307.5 (299.5,320)
June	29 (28,29)	99.2 (72.8,110.5)	11.1 (10.6,13.4)	82 (79,85)	286.5 (282.5,303.5)
July	28 (28,28)	73 (50.4,103.5)	12.1 (10.4,13.4)	84.5 (83.5,85.5)	261.8 (244.8,276)
August	28 (27.5,28)	78.4 (61.2,101.3)	10.6 (9.4,11.1)	86.5 (82,87.5)	249.3 (231.8,271)
September	27 (27,28)	57.1 (43.3,144.7)	8.4 (7.5,9.4)	86.5 (82,89)	249 (225,259)
October	25 (25,26)	55.3 (23,121.8)	5.9 (4.9,6)	85 (82,87)	284.5 (271,293.5)
November	21 (21,22)	5.2 (0.2,31)	5.5 (5.3,6.1)	78 (77,79)	300 (282,300)
December	18 (18,20)	0.7 (0.2,6.7)	6.1 (5.1,6.2)	71 (70,73)	264.5 (258.5,267.5)
3. South region					
3.1. Vishakhapatnam (Andhra Pradesh)					
January	23 (23,24)	3.5 (1.3,6.1)	10.6 (9.4,11.1)	68 (67,72)	277.5 (274.5,277.5)
February	25 (24,25)	1.4 (0.9,4.2)	10.1 (9.4,10.8)	68 (65,70)	280 (277.5,288.5)
March	27 (27,28)	3.4 (3.3,16.2)	13.6 (11.5,14)	70 (65,70)	308.5 (298,310)
April	29 (29,30)	5 (1.3,12.6)	17.4 (16.7,19.1)	71 (70,74)	330 (320,335)
May	31 (31,31)	8.6 (3.5,18)	17 (15.9,18.5)	71 (70,72)	378 (371,381.5)
June	31 (31,31)	91 (40.8,129.1)	16.9 (14.3,17.3)	70 (66,71)	274.5 (268.5,306)
July	29.5 (29,30)	77.6 (60.1,105.1)	18.5 (16.2,20.1)	73 (71.5,74.5)	252.5 (226.8,289.8)
August	29 (29,30)	111.7 (96.7,120.4)	13.8 (13.5,16.6)	74.5 (73,77)	241 (234.5,263.5)
September	29 (29,29)	127 (104.7,164.2)	11.8 (11.7,13)	76 (75.5,77)	234.5 (226.3,243.8)
October	27 (27,28)	90.4 (50.2,182.7)	11.7 (9.1,13.2)	72 (69,77)	222 (205.5,236.5)
November	25 (25,26)	46.2 (19.2,144.8)	12.1 (11.5,15.1)	70 (66,71)	207 (202,217.5)
December	24 (24,24)	4.2 (2.6,14.6)	13.8 (12,14.4)	67 (66,70)	225.5 (222,228)

For peer review only - http://bmjopen.bmj.com/site/about/guidelines.xhtml

Region/ Place (State) and months	Temperature (°C)	Rainfall (mm)	Wind speed (Kmph)	Humidity (%)	Sunshine (hour)
3.2. Hyderabad (Telangana)					
January	22 (21,23)	0.5 (0.1,1.5)	12.1 (10.1,12.6)	47 (46,51)	277.5 (274.5,277.5)
February	24 (24,25)	0 (0,1.6)	12.4 (10.6,12.9)	41 (38,45)	280 (277.5,288.5)
March	28 (28,29)	3.4 (0.1,10.6)	11.1 (10.3,11.9)	34 (32,39)	308.5 (298,310)
April	32 (31,33)	2.9 (1.5,5.3)	9.7 (9.2,11.9)	33 (27,35)	330 (320,335)
May	34 (33,35)	9.4 (7.3,17.6)	12.9 (12,14.4)	30 (28,34)	378 (371,381.5)
June	29 (28,29)	111.6 (33.3,131.2)	18.7 (18.1,20.7)	57 (49,63)	274.5 (268.5,306)
July	26 (26,27.5)	66.4 (47.1,81)	20.7 (18.4,23)	72.5 (65,75)	252.5 (226.8,289.8)
August	26 (25,26.5)	68.6 (36,77.7)	16.6 (15.3,17.5)	76 (70,78)	241 (234.5,263.5)
September	26 (25,26)	51.9 (40.3,74.1)	12.8 (11.3,13.9)	74.5 (70.5,78.5)	234.5 (226.3,243.8)
October	24 (24,25)	35.9 (14.9,45.2)	10.1 (9.2,10.6)	65 (59,70)	222 (205.5,236.5)
November	22 (21,23)	3.1 (0.5,18.8)	10.2 (9.5,10.8)	60 (51,62)	207 (202,217.5)
December	21 (20,22)	0.3 (0.2,1.1)	10.3 (10.1,11.3)	50 (49,53)	225.5 (222,228)
3.3. Coimbatore (Tamil Nadu)					
January	23 (23,23)	2.2 (1.1,26.6)	7.4 (6.9,8.6)	63 (63,66)	290 (285,305.5)
February	25 (24,25)	3 (0.9,8.3)	7.8 (7.4,8.9)	55 (51,56)	277 (272,286)
March	28 (27,28)	26.7 (10.1,60.5)	7.1 (7.1,8.6)	53 (50,55)	299.5 (298,302.5)
April	30.5 (30,31)	8.9 (6.7,28.2)	9.2 (8.3,11.6)	52 (52,54)	297.5 (284.5,297.5)
May	30 (29,30)	29.7 (29.7,30.5)	12.4 (11.9,13.2)	59 (59,61)	334 (317,335)
June	27 (27,27)	4.9 (4.9,6.7)	18.1 (17.7,18.3)	64 (64,66)	301.5 (285.5,302)
July	27 (27,28)	0.2 (0,1.7)	18.5 (17.9,19.3)	63 (61,64)	291 (268.5,308.5)
August	27 (26,27)	3.1 (0.9,7.9)	16.1 (14.8,17.6)	65 (63,67)	291.5 (274.5,296.5)
September	27 (27,27)	15.6 (8.3,42.8)	16.3 (14.5,17.6)	65.5 (65,67)	278 (256.5,282)
October	26 (26,26)	53.2 (53.2,63.8)	9.7 (8.3,10.6)	68 (68,71)	268.3 (254.5,270)
November	24 (24,24)	32.6 (32.6,34.9)	6.1 (6.1,7)	72 (69,78)	205.5 (194,206.5)
December	23 (23,23)	14.3 (6,15.5)	7 (6.7,7.9)	71 (67,74)	242.3 (225.5,267)
3.4. Chennai (Tamil Nadu)					
January	25 (25,25)	7.7 (4.4,10.5)	15.4 (12.4,16.6)	73 (70,75)	267 (259.5,273.5)
February	25 (25,26)	1.5 (0.3,26)	13.2 (11.7,14)	72 (70,74)	275.5 (272.5,287)
March	28 (27,28)	1.8 (1.1,10.1)	14.8 (13.6,15.5)	72 (71,73)	301 (294,304)
April	30 (30,30)	7.2 (0.3,35.3)	18.9 (16.9,20.1)	72 (71,74)	344.5 (330,352.5)
May	32 (32,32)	18.6 (8.3,37.5)	18.9 (18.2,19.1)	66 (66,67)	381.5 (355,383)
June	31 (31,32)	37.8 (19,63.2)	17.4 (16.9,18.4)	62 (61,66)	336.5 (327.5,348.5)
July	30.5 (30,31)	37.9 (22.1,61.8)	16.8 (15.7,17.5)	65 (63,65.5)	343.5 (339,354.3)
August	30 (29,30)	75.4 (61.7,88.7)	15.2 (14.1,16)	69 (66.5,70.5)	339.3 (335.8,359.8)
September	29.5 (29,30)	101.1 (91.5,105.6)	13.3 (13,13.8)	72 (70,72)	333.5 (317,347.8)
October	29 (28,29)	102.6 (81.5,154.2)	12.2 (10.9,13.8)	73 (71,74)	252 (247,263.5)
November	27 (27,27)	60 (48.4,344.4)	17.3 (15.3,17.7)	74 (72,79)	201 (190.5,213)
December	26 (25,26)	84.8 (58.6,111.3)	18 (16.5,20)	76 (72,77)	199 (188.5,208)
3.5. Trivandrum (Kerala)					
January	26 (26,26)	3.2 (1.5,7.7)	8.7 (8.2,9.5)	70 (68,71)	299.5 (296.5,301)
February	26 (26,26)	6.1 (1.4,23)	8.8 (8.7,10.7)	71 (65,72)	278.5 (269.5,284)
March	28 (27,28)	37.8 (14.8,53.6)	9.8 (9.1,10.7)	71 (70,75)	293.5 (288,299.5)
April	28 (28,29)	57.3 (32.2,115.9)	12.2 (10.7,14.3)	79 (72,79)	278 (264,285)
May	28 (28,29)	24.9 (17.4,42.5)	15 (14.6,18)	79 (72,79)	252 (245.5,267.5)
June	27 (27,28)	82.2 (46.9,110.2)	20.1 (17.4,21.5)	78 (74,79)	243 (213,256)
July	26.5 (26,27.5)	65.6 (15.1,101.1)	21.1 (18.7,22.1)	78 (75,80)	266.3 (248.3,278.8)
August	26 (26,27.5)	33.6 (7.8,72.9)	18.8 (17,20.5)	80 (75.5,81)	264.5 (240.8,285.5)
September	26.5 (26,27)	61.7 (40.3,77.2)	17 (13.8,17.4)	80 (77,81.5)	256 (244,263.3)
October	27 (26,27)	117.9 (32.3,220.7)	11.4 (10.1,14.9)	78 (75,80)	266 (245.5,286)

For peer review only - http://bmjopen.bmj.com/site/about/guidelines.xhtml

Region/ Place (State) and months	Temperature (°C)	Rainfall (mm)	Wind speed (Kmph)	Humidity (%)	Sunshine (hour)
November	27 (27,27)	107.7 (47,140)	8.6 (8,9.1)	74 (72,76)	189 (174,205)
December	26 (26,27)	25.1 (15.7,37)	8.9 (8.6,9.6)	72 (72,75)	272.5 (267,280.5)
4. West region					
4.1. Mumbai (Maharashtra)					
January	24 (24,25)	0 (0,0.4)	10 (9.7,10.9)	52 (51,53)	310 (310,310)
February	25 (25,26)	0.4 (0,2.9)	10 (9.2,10.2)	54 (51,55)	280 (275.5,281)
March	28 (27,28)	0.6 (0,1.3)	10.5 (9.6,10.7)	53 (51,56)	308.5 (305.5,310)
April	29 (29,29)	0.1 (0,0.2)	9.7 (9.4,10.3)	63 (61,66)	300 (292.5,300)
May	30 (29,30)	1.3 (0.6,8)	10.5 (10.1,10.7)	72 (68,73)	305.5 (292,307)
June	29 (29,29)	292.8 (156.7,370)	16 (14.6,16.7)	78 (75,78)	217 (190,252)
July	27 (27,28)	450.2 (391.4,540.5)	17.3 (16,18.7)	83.5 (82,85.5)	164.8 (147.5,193.3)
August	27 (27,27)	282.7 (213.5,376.4)	14.1 (12.7,15.5)	85.5 (84,86.5)	190.8 (186.5,212.8)
September	27 (27,27)	136.2 (103.3,196.1)	9.8 (9.5,11.7)	82 (80,83)	229.8 (225.5,245)
October	28 (27,29)	29.9 (25.5,48.4)	8.1 (7.7,8.8)	67 (67,72)	299.5 (286.5,304)
November	27 (27,28)	2.3 (0,9.9)	9 (8.6,9)	55 (53,58)	257.5 (251.5,258.5)
December	26 (25,26)	0.2 (0,3.4)	9.8 (9,10)	51 (49,55)	295 (293.5,297.5)
4.2. Jamnagar (Gujarat)					
January	22 (21,23)	0 (0,0.1)	15.8 (14.4,17)	44 (41,49)	310 (307,310)
February	24 (23,24)	0 (0,0.6)	16 (14.5,16.8)	45 (44,50)	278.5 (274.5,281)
March	27 (27,28)	0.1 (0,0.2)	16.5 (15.9,17.1)	47 (44,53)	308.5 (306,308.5)
April	29 (29,30)	0 (0,0.4)	20.8 (18.3,21.3)	56 (54,56)	300 (297,300)
May	31 (30,31)	0.1 (0.1,0.2)	24.8 (23.9,25.3)	65 (63,67)	355 (353.5,357.5)
June	31 (31,32)	35.8 (5,93.6)	23.9 (21.7,27.9)	68 (67,69)	311.5 (296.5,321)
July	29 (29,29)	119.4 (88.5,141.7)	25.4 (23.8,27.9)	77.5 (75,78)	167.8 (143.5,197.5)
August	28 (28,28.5)	61.6 (39.7,107.3)	23.2 (22.1,24.6)	79 (77,80)	156.5 (143.5,182)
September	28 (28,29)	70.6 (24.3,113.3)	18.2 (17.8,19.7)	74 (73.5,77)	244.8 (225.3,259)
October	30 (29,31)	0.8 (0.2,12.4)	14 (12.5,14.1)	51 (48,62)	310 (308,310)
November	28 (27,29)	0.1 (0,1.9)	13.9 (13,14.6)	43 (40,50)	230 (228.5,231)
December	24 (24,25)	0 (0,0)	16.2 (14.5,17.1)	43 (39,46)	252.5 (250,252.5)
4.3. Jaipur (Rajasthan)					
January	14 (14,15)	1.9 (0.3,7.9)	8.3 (8.2,8.5)	47 (42,50)	253.5 (245.5,255)
February	18 (18,20)	1 (0.3,3.5)	10.2 (8.9,10.9)	34 (31,44)	277.5 (275.5,288.5)
March	25 (24,25)	0.8 (0,4.1)	10.2 (10,11)	24 (24,29)	304 (301.5,308.5)
April	31 (31,32)	3 (0.8,4.6)	11.2 (10.5,11.7)	19 (16,20)	323 (315.5,327.5)
May	36 (36,36)	5.5 (0.4,6.9)	14.3 (13.1,16.5)	19 (15,21)	383.5 (381.5,385)
June	36 (36,37)	9.9 (3.5,20.5)	13.9 (12.4,17.4)	30 (28,34)	363 (359.5,367.5)
July	32 (32,33.5)	48.4 (33.6,69)	12.8 (10.6,13.9)	55.5 (51,56.5)	351.8 (338.8,369.5)
August	29.5 (29,31)	74.1 (59.6,138.3)	11.1 (10.5,12.7)	66.5 (58.5,73.5)	287.5 (278,302.5)
September	29.5 (28.5,30)	19.4 (14.2,31)	10.4 (9.4,11.5)	53.5 (46.5,62.5)	281.8 (274.8,290.5)
October	28 (27,28)	2.3 (0,4.7)	8.4 (7.4,9.4)	31 (27,41)	263.5 (262.5,265)
November	22 (21,23)	0 (0,0.8)	7.2 (6.7,8)	29 (26,34)	225 (223.5,225)
December	16 (16,17)	0.4 (0,0.7)	7.6 (6.3,8.4)	37 (33,39)	232.5 (229.5,232.5)

Note: IQR- Interquartile range

For peer review only - http://bmjopen.bmj.com/site/about/guidelines.xhtml

Supplementary Figure 2: The pattern of monthly meteorological parameters across the regions

SF2a: Monthly median temperature for different regions in India (^{0}C)

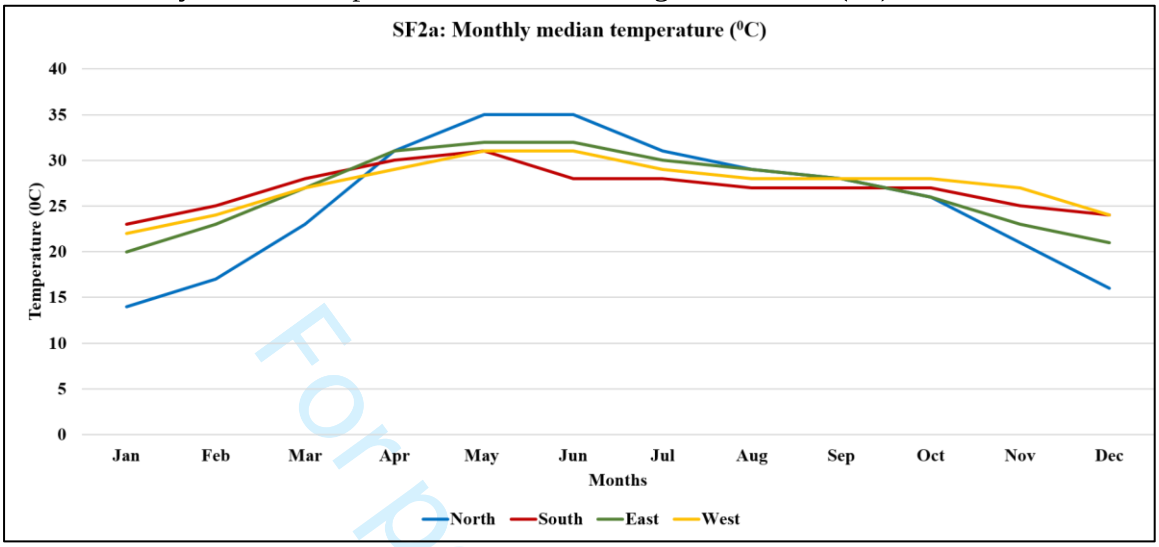

SF2b: Monthly median rainfall (mm) for different regions in India

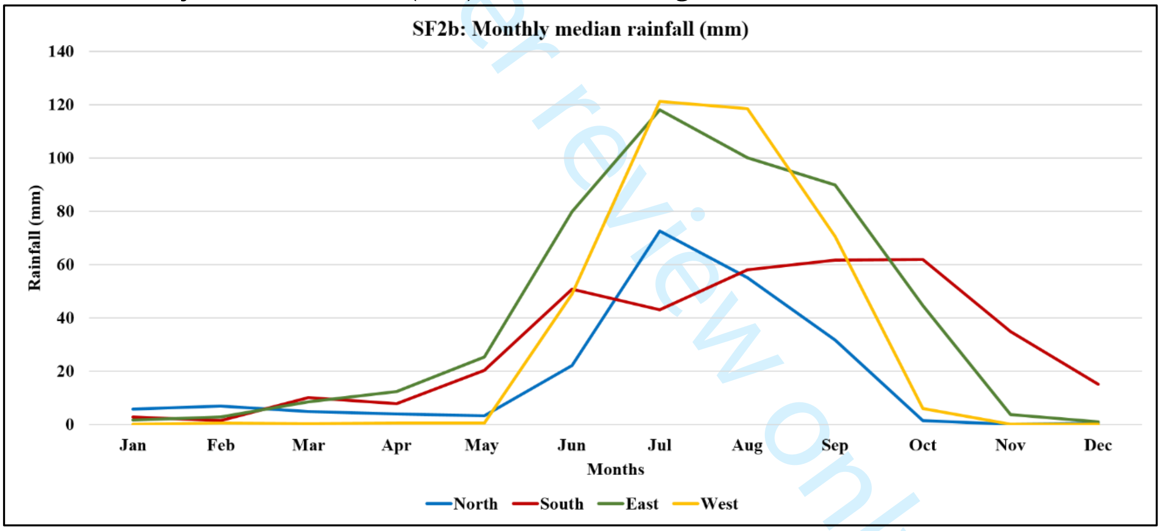

For peer review only

SF2c: Monthly median wind speed (Kmph) for different regions in India

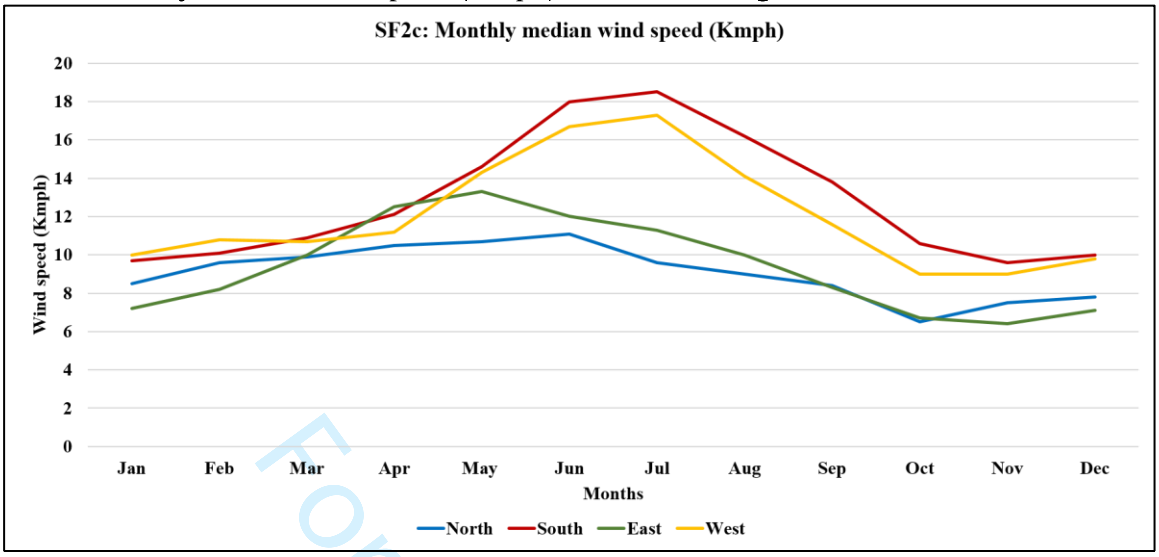

SF2d: Monthly median humidity (%) for different regions in India

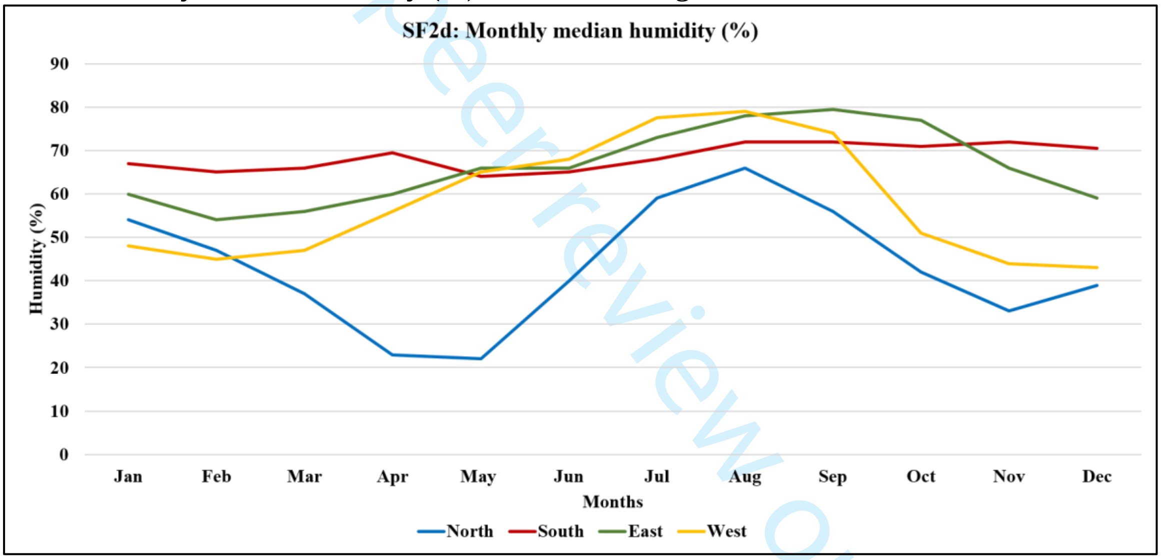

SF2e: Monthly median sunshine (hours) for different regions in India

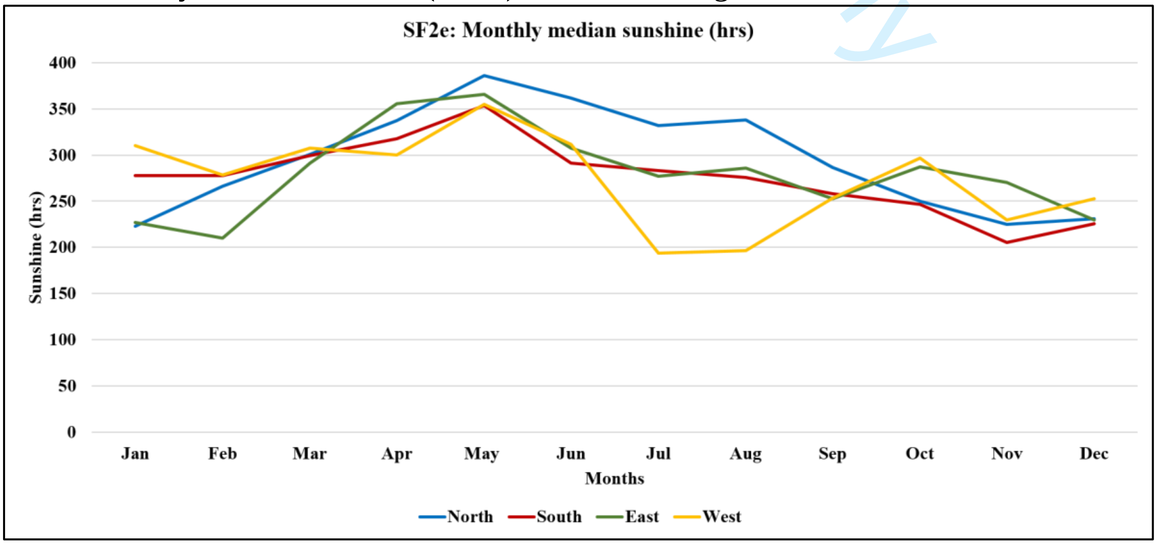

For peer review only - http://bmjopen.bmj.com/site/about/guidelines.xhtml

For peer review only

Supplementary Figure 3: The pooled monthly (SF3a) and seasonal (SF3b) distribution of intussusception in children during July 2010 to September 2017

SF3a: The monthly trend of intussusception cases

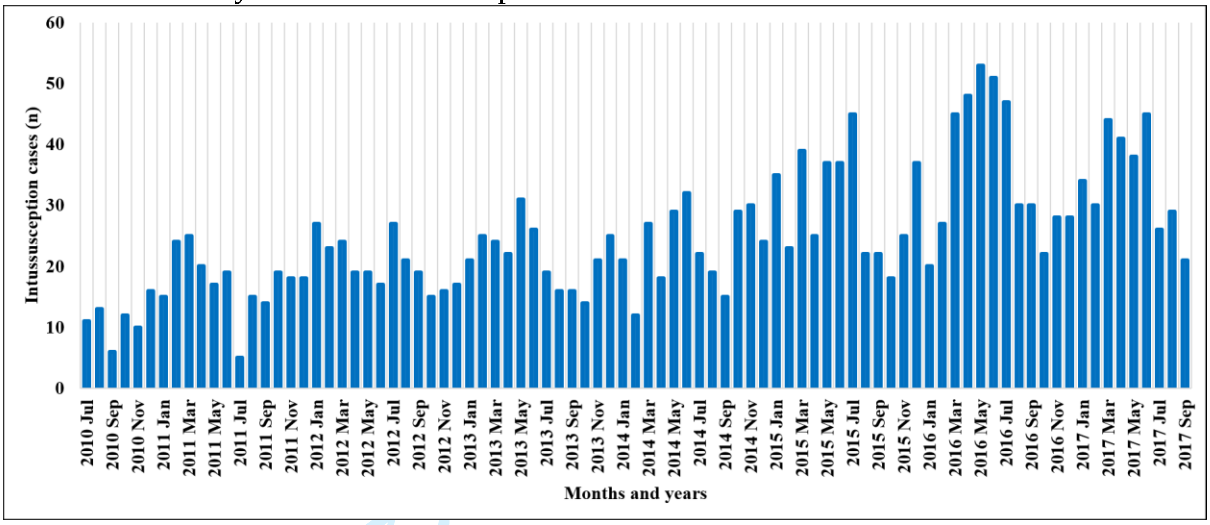

SF3b: The seasonal trend of intussusception cases

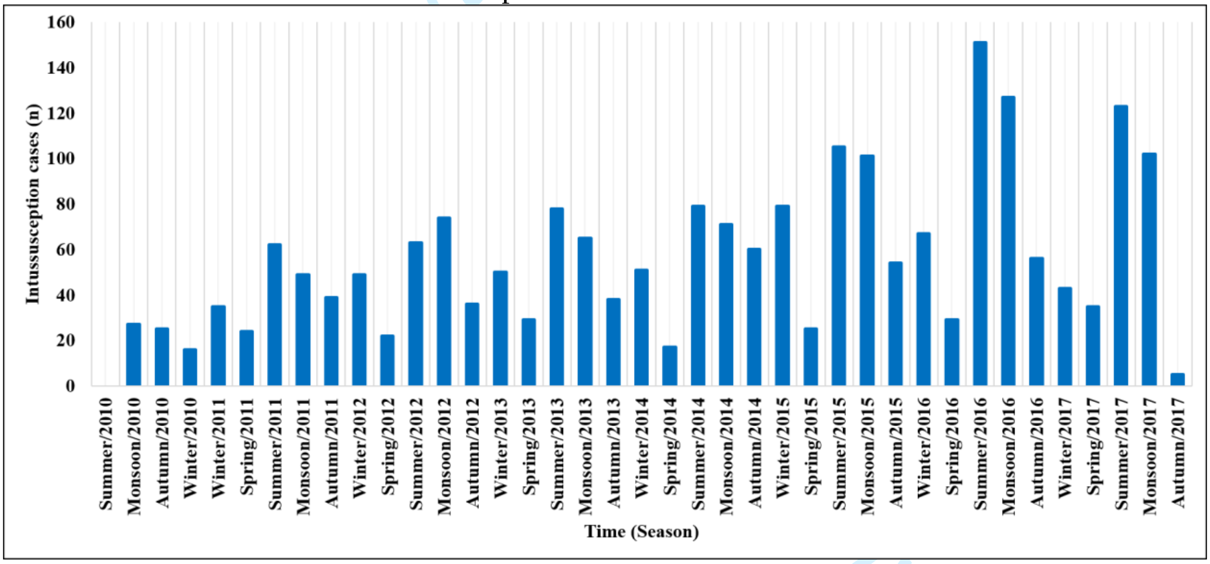

Supplementary Table 5. Pearson correlation analysis between monthly intussusception cases and monthly median meteorological parameters for the study sites

Regions/Study sites (n, number of cases)	Pearson correlation coefficient (r) for meteorological parameters				
	Temperature (°C)	Rainfall (mm)	Wind Speed (kmph)	Humidity (%)	Sunshine (hour)
North region					
Srinagar (n=145)	.253*	.164	.183	.012	.239*
Delhi and Gurgaon (n=96)	.107	-.045	.183*	-.173*	.134
Lucknow (110)	.091	.003	.285**	-.106	.192
Indore (n=19)	.101	-.211*	.290**	-.309**	.188
East region					
Bhubaneswar/Cuttack (n=335)	.047	-.056	.125	-.167*	.035
Berhampur (n=30)	.050	.047	.035	.044	.036
Kolkata (n=80)	.220*	-.052	.291**	-.144	.253*
Patna (n=19)	-.124	-.189	-.038	-.216*	-.040
Guwahati (n=112)	.220*	.001	.317**	-.119	.194
Agartala (n=17)	.082	.080	.139	-.017	.016
South region					
Vishakhapatnam (n=77)	.088	.053	-.002	.022	.029
Hyderabad (n=2)	-.030	-.061	-.131	.022	-.096
Coimbatore (n=136)	-.015	-.075	-.155	-.028	.017
Chennai (n=92)	.052	-.017	.007	.015	-.016
Trivandrum (n=774)	.085	-.286**	-.011	-.323**	.229*
West region					
Mumbai (n=34)	-.066	-.081	-.027	-.103	.035
Jamnagar (n=54)	.110	-.017	.011	.026	.105
Jaipur (n=29)	.187	-.078	.148	.001	.122

Note: Pearson correlation coefficient (r); Significance two tailed
** Significance level- p value <0.05; ** Significance level- p value <0.01*

For peer review only

Supplementary Table 6. Spearman's Rank correlation analysis between monthly intussusception cases and monthly median meteorological parameters for the study sites

Regions/Study sites (n, number of cases)	Pearson correlation coefficient (r) for meteorological parameters				
	Temperature (°C)	Rainfall (mm)	Wind Speed (kmph)	Humidity (%)	Sunshine (hour)
North region					
Srinagar (n=145)	.208	.122	.146	.032	.169
Delhi and Gurgaon (n=96)	.067	-.101	.136	-.184*	.065
Lucknow (110)	.166	.064	.331**	-.101	.225*
Indore (n=19)	.095	-.148	.286**	-.301**	.138
East region					
Bhubaneswar/Cuttack (n=335)	.036	-.068	.117	-.143	-.009
Berhampur (n=30)	.058	.037	.044	.035	.021
Kolkata (n=80)	.097	-.084	.170	-.236*	.125
Patna (n=19)	-.146	-.264*	-.085	-.231*	-.040
Guwahati (n=112)	.240*	-.014	.290**	-.118	.165
Agartala (n=17)	.107	.107	.154	.021	.044
South region					
Vishakhapatnam (n=77)	.106	.081	.048	.017	.060
Hyderabad (n=2)	-.012	-.052	-.138	.040	-.043
Coimbatore (n=136)	.002	-.123	-.147	-.091	.070
Chennai (n=92)	.055	.041	.025	.055	-.027
Trivandrum (n=774)	.134	-.287**	-.026	-.353**	.301**
West region					
Mumbai (n=34)	-.025	-.140	-.092	-.120	.046
Jamnagar (n=54)	.056	.071	.019	-.009	.106
Jaipur (n=29)	.131	-.082	.069	-.075	.079

Note: Pearson correlation coefficient (r); Significance two tailed
** Significance level- p value <0.05; ** Significance level- p value <0.01*

For peer review only - http://bmjopen.bmj.com/site/about/guidelines.xhtml

Supplementary Table 7. Regression analysis of the association between the intussusception cases and meteorological parameters for the study sites.

Study sites/ Variables	Regression coefficient, β (95% CI)	Standard error, S_β	χ2 - value	p value
North region				
Srinagar (n=145)#				
Temperature (^{0}C)	0.043(0.003,0.082)	0.020	4.479	**0.034**
Rainfall (mm)	0.007(-0.004,0.018)	0.005	1.616	0.204
Wind Speed (Kmph)	0.150(-0.048,0.347)	0.100	2.207	0.137
Humidity (%)	0.001(-0.018,0.020)	0.009	0.010	0.921
Sunshine (hour)	0.005(0.000,0.010)	0.002	3.930	**0.047**
Delhi and Gurgaon (n=96)*				
Temperature (^{0}C)	0.025(-0.005,0.055)	0.015	2.712	0.100
Rainfall (mm)	-0.003(-0.012,0.006)	0.004	0.476	0.490
Wind Speed (Kmph)	0.229(0.068,0.390)	0.082	7.805	**0.005**
Humidity (%)	-0.021(-0.036,-0.005)	0.007	7.041	**0.008**
Sunshine (hour)	0.004(0.000,0.008)	0.002	4.205	**0.040**
Lucknow (n=110)*				
Temperature (^{0}C)	0.013(-0.014,0.041)	0.014	0.873	0.350
Rainfall (mm)	0.000(-0.008,0.008)	0.004	0.001	0.972
Wind Speed (Kmph)	0.224(0.074,0.374)	0.077	8.580	**0.003**
Humidity (%)	-0.008(-0.022,0.006)	0.007	1.195	0.274
Sunshine (hour)	0.004(0.000,0.007)	0.002	3.915	**0.048**
Indore (n=19)*				
Temperature (^{0}C)	0.036(-0.033,0.105)	0.035	1.055	0.304
Rainfall (mm)	-0.039(-0.078,0.001)	0.020	3.723	0.054
Wind Speed (Kmph)	0.623(0.193,1.054)	0.219	8.062	**0.005**
Humidity (%)	-0.059(-0.097,-0.021)	0.019	9.110	**0.003**
Sunshine (hour)	0.009(0.000,0.017)	0.004	3.556	0.059
East region				
Bhubaneswar/Cuttack (n=335)#				
Temperature (^{0}C)	0.012(-0.032,0.057)	0.023	.286	0.593
Rainfall (mm)	-0.001(-0.006,0.003)	0.002	.429	0.513
Wind Speed (Kmph)	0.060(-0.021,0.141)	0.041	2.131	0.144
Humidity (%)	-0.022(-0.044,0.000)	0.011	3.953	**0.047**
Sunshine (hour)	0.001(-0.003,0.005)	0.002	.157	0.692
Berhampur (n=30)*				
Temperature (^{0}C)	0.023(-0.068,0.114)	0.046	0.239	0.625
Rainfall (mm)	0.002(-0.006,0.01)	0.004	0.211	0.646
Wind Speed (Kmph)	0.027(-0.128,0.182)	0.079	0.118	0.731
Humidity (%)	0.009(-0.032,0.051)	0.021	0.186	0.666
Sunshine (hour)	0.001(-0.006,0.009)	0.004	0.125	0.724
Kolkata (n=80)*				
Temperature (^{0}C)	0.113(0.050,0.176)	0.032	12.448	**0.000**
Rainfall (mm)	-0.002(-0.007,0.003)	0.002	0.710	0.400
Wind Speed (Kmph)	0.233(0.134,0.332)	0.050	21.269	**0.000**
Humidity (%)	-0.032(-0.059,-0.005)	0.013	5.433	**0.020**
Sunshine (hour)	0.010(0.005,0.014)	0.002	16.577	**0.000**

For peer review only - http://bmjopen.bmj.com/site/about/guidelines.xhtml

Patna (n=19)*				
Temperature (^{0}C)	-0.067(-0.177,0.042)	0.056	1.452	0.228
Rainfall (mm)	-0.012(-0.025,0.001)	0.006	3.157	0.076
Wind Speed (Kmph)	-0.037(-0.235,0.161)	0.101	0.137	0.711
Humidity (%)	-0.063(-0.124,-0.003)	0.031	4.208	**0.040**
Sunshine (hour)	-0.002(-0.012,0.008)	0.005	0.150	0.698
Guwahati (n=112)*				
Temperature (^{0}C)	0.050(0.002,0.098)	0.025	4.097	**0.043**
Rainfall (mm)	0.000(-0.004,0.004)	0.002	0.000	0.995
Wind Speed (Kmph)	0.118(0.038,0.199)	0.041	8.408	**0.004**
Humidity (%)	-0.012(-0.034,0.01)	0.011	1.212	0.271
Sunshine (hour)	0.004(0.000,0.008)	0.002	3.207	0.073
Agartala (n=17)*				
Temperature (^{0}C)	0.060(-0.066,0.186)	0.064	0.874	0.350
Rainfall (mm)	0.005(-0.006,0.016)	0.005	0.838	0.360
Wind Speed (Kmph)	0.166(-0.042,0.374)	0.106	2.435	0.119
Humidity (%)	-0.005(-0.061,0.050)	0.028	0.037	0.848
Sunshine (hour)	0.001(-0.009,0.011)	0.005	0.033	0.857
South region				
Vishakhapatnam (n=77)*				
Temperature (^{0}C)	0.043(-0.058,0.144)	0.051	0.702	0.402
Rainfall (mm)	0.003(-0.007,0.012)	0.005	0.255	0.613
Wind Speed (Kmph)	-0.001(-0.071,0.070)	0.036	0.001	0.981
Humidity (%)	0.008(-0.069,0.085)	0.039	0.042	0.837
Sunshine (hour)	0.001(-0.005,0.007)	0.003	0.078	0.780
Hyderabad (n=2)*				
Temperature (^{0}C)	-0.088(-0.717,0.541)	0.321	0.075	0.784
Rainfall (mm)	-0.018(-0.085,0.048)	0.034	0.298	0.585
Wind Speed (Kmph)	-0.650(-2.074,0.774)	0.726	0.801	0.371
Humidity (%)	0.051(-0.436,0.539)	0.249	0.043	0.837
Sunshine (hour)	-0.017(-0.056,0.022)	0.020	0.764	0.382
Coimbatore (n=136)*				
Temperature (^{0}C)	-0.007(-0.082,0.068)	0.038	0.033	0.857
Rainfall (mm)	-0.003(-0.011,0.004)	0.004	0.825	0.364
Wind Speed (Kmph)	-0.053(-0.108,0.002)	0.028	3.506	0.061
Humidity (%)	-0.010(-0.067,0.047)	0.029	0.119	0.730
Sunshine (hour)	0.000(-0.004,0.005)	0.002	0.043	0.836
Chennai (n=92)*				
Temperature (^{0}C)	0.027(-0.065,0.120)	0.047	0.337	0.562
Rainfall (mm)	-0.001(-0.010,0.008)	0.005	0.036	0.850
Wind Speed (Kmph)	0.003(-0.062,0.067)	0.033	0.006	0.938
Humidity (%)	0.006(-0.064,0.076)	0.036	0.028	0.868
Sunshine (hour)	0.000(-0.006,0.005)	0.003	0.030	0.863
Trivandrum (n=774)*				
Temperature (^{0}C)	0.015(-0.016,0.047)	0.016	0.895	0.344
Rainfall (mm)	-0.005(-0.008,-0.002)	0.002	10.159	**0.001**
Wind Speed (Kmph)	-0.001(-0.024,0.021)	0.011	0.016	.898
Humidity (%)	-0.044(-0.068,-0.020)	0.012	12.938	**0.000**

For peer review only - http://bmjopen.bmj.com/site/about/guidelines.xhtml

Sunshine (hour)	0.002(0.001,0.004)	0.001	6.538	**0.011**
West region				
Mumbai (n=34)*				
Temperature (^{0}C)	-0.040(-0.165,0.085)	0.064	0.387	0.534
Rainfall (mm)	-0.003(-0.011,0.005)	0.004	0.580	0.446
Wind Speed (Kmph)	-0.016(-0.140,0.108)	0.063	0.065	0.799
Humidity (%)	-0.013(-0.039,0.013)	0.013	0.929	0.335
Sunshine (hour)	0.001(-0.006,0.008)	0.004	0.108	0.742
Jamnagar (n=54)*				
Temperature (^{0}C)	0.066(-0.042,0.173)	0.055	1.440	0.230
Rainfall (mm)	-0.001(-0.006,0.005)	0.003	0.035	0.852
Wind Speed (Kmph)	0.006(-0.090,0.102)	0.049	0.015	0.903
Humidity (%)	0.003(-0.017,0.023)	0.010	0.080	0.777
Sunshine (hour)	0.003(-0.002,0.009)	0.003	1.305	0.253
Jaipur (n=29)*				
Temperature (^{0}C)	0.150(-0.009,0.309)	0.081	3.425	0.064
Rainfall (mm)	-0.003(-0.012,0.005)	0.004	0.600	0.439
Wind Speed (Kmph)	0.092(-0.031,0.216)	0.063	2.141	0.143
Humidity (%)	0.000(-0.027,0.027)	0.014	0.000	0.993
Sunshine (hour)	0.005(-0.003,0.013)	0.004	1.471	0.225

*Note: Tests used for regression analysis: * Poisson Regression; # Negative Binomial Regression*

For peer review only

Supplementary Table 8. Intussusception cases in children at different temperature ranges at pooled and regional level

Pooled/ Region	Temperature range (°C)	Years of data collection								Total
		2010	2011	2012	2013	2014	2015	2016	2017	
Pooled	<20°C	8	14	24	29	26	39	55	51	246
	21°C -25°C	2	31	44	32	43	54	42	31	279
	26°C -30°C	56	149	153	166	164	208	245	164	1305
	>30°C	2	15	23	33	45	63	88	62	331
	Total	68	209	244	260	278	364	430	308	2161
North Region	<20°C	5	8	18	28	20	27	45	46	197
	21°C -25°C	1	5	3	2	10	7	4	1	33
	26°C -30°C	1	2	4	3	4	6	13	3	36
	>30°C	2	4	12	11	14	21	29	11	104
	Total	9	19	37	44	48	61	91	61	370
South Region	<20°C	0	0	0	0	0	0	0	0	0
	21°C -25°C	1	14	15	10	7	11	11	8	77
	26°C -30°C	49	117	113	132	116	157	167	116	967
	>30°C	0	3	2	7	1	6	6	12	37
	Total	50	134	130	149	124	174	184	136	1081
East Region	<20°C	3	6	6	1	5	10	9	4	44
	21°C -25°C	0	10	21	17	22	36	23	21	150
	26°C -30°C	6	24	26	23	37	36	53	35	240
	>30°C	0	5	8	13	20	30	46	37	159
	Total	9	45	61	54	84	112	131	97	593
West Region	<20°C	0	0	0	0	1	2	1	1	5
	21°C -25°C	0	2	5	3	4	0	4	1	19
	26°C -30°C	0	6	10	8	7	9	12	10	62
	>30°C	0	3	1	2	10	6	7	2	31
	Total	0	11	16	13	22	17	24	14	117

For peer review only - http://bmjopen.bmj.com/site/about/guidelines.xhtml

Supplementary Table 9. Variations in the intussusception cases between the different temperature categories by Kruskal-Wallis H test

Level	df	χ^2-statistic	P- value
Pooled	3	15.933	< 0.001
North Region	3	21.054	< 0.001
South Region	3	50.625	< 0.001
East Region	3	3.502	0.3205
West Region	3	5.606	0.1324

Notes: Pooled- all sites/regions combined; df: degrees of freedom; χ^2-statistic: critical chi square value

For peer review only

For peer review only - http://bmjopen.bmj.com/site/about/guidelines.xhtml

Supplementary Figure 4: Association of monthly intussusception cases with mean monthly temperatures

Pooled for all regions

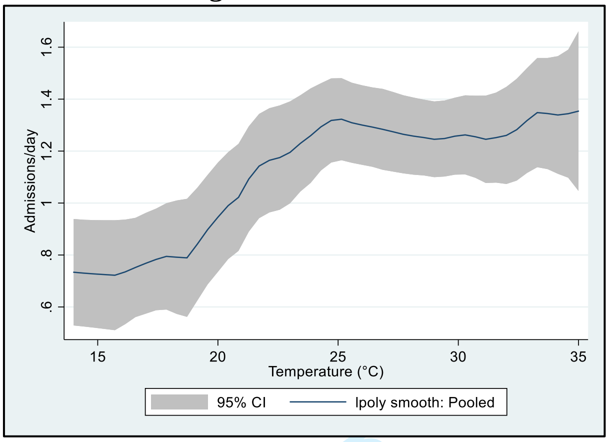

North region

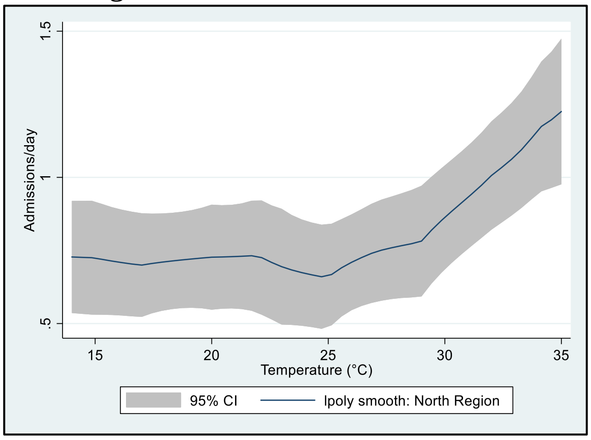

South region

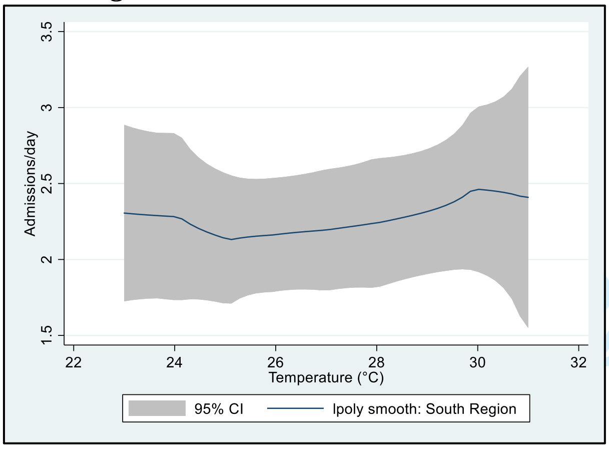

East region

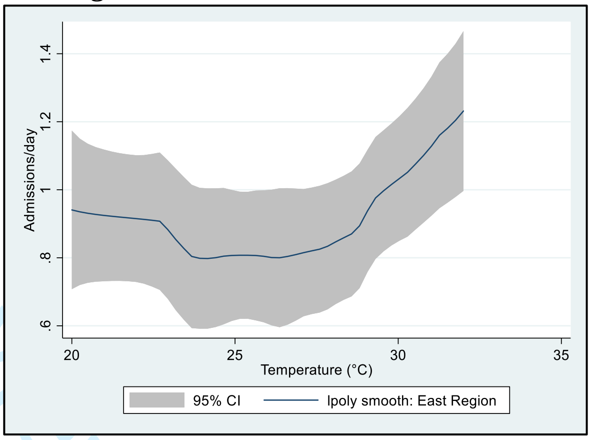

West region

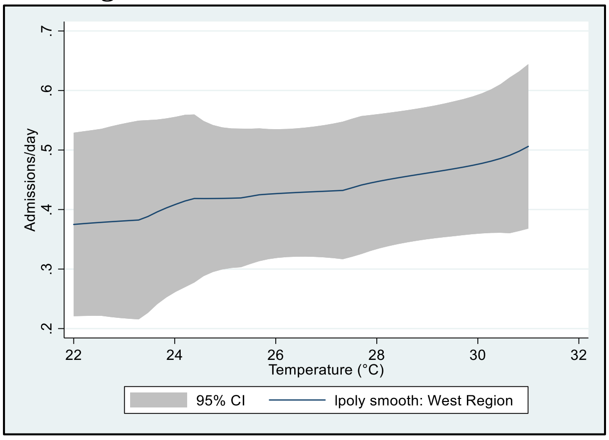

Supplementary Figure 5: Association of monthly intussusception cases with mean monthly humidity

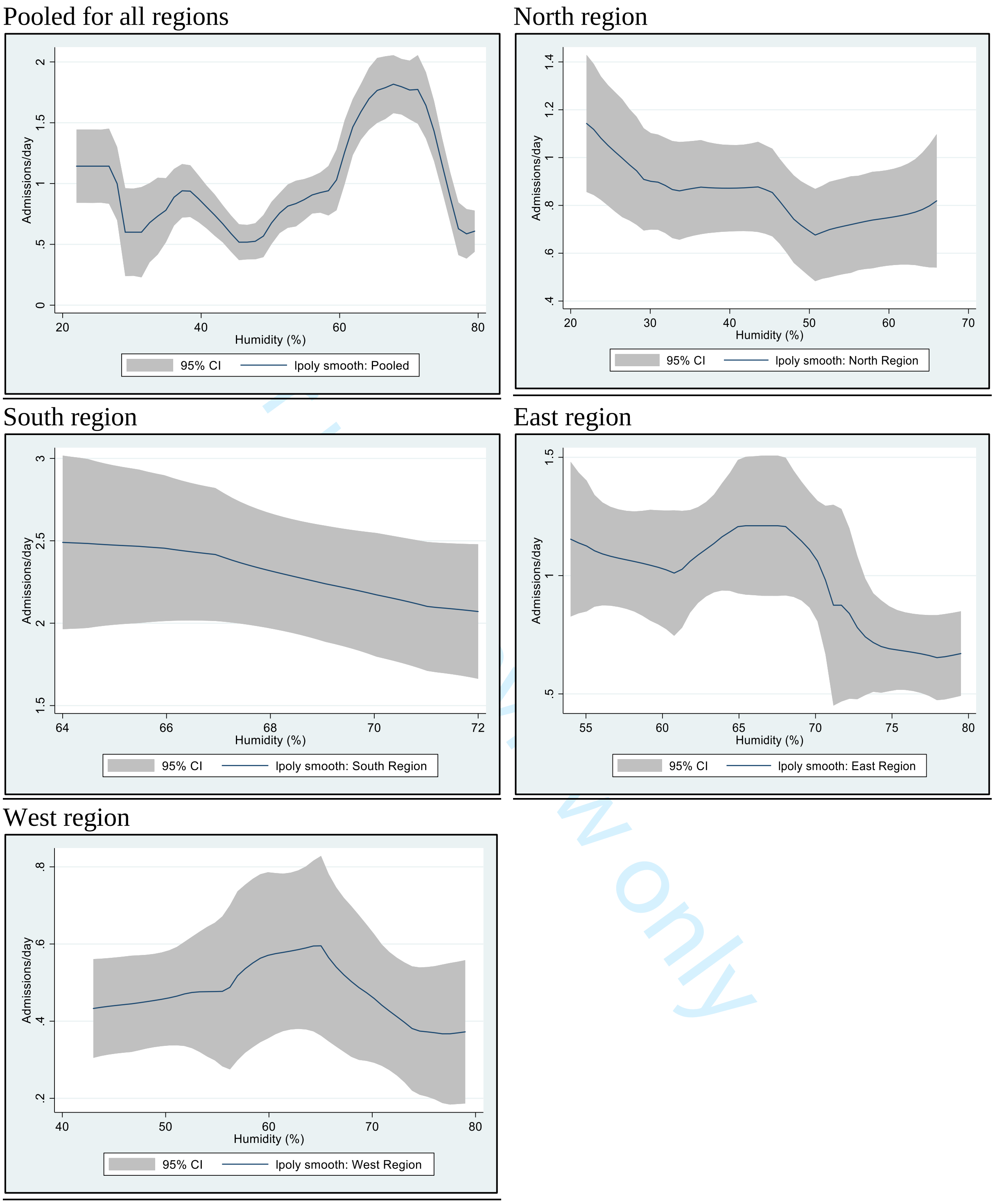

Supplementary Figure 6: Association of monthly intussusception cases with mean monthly wind speed

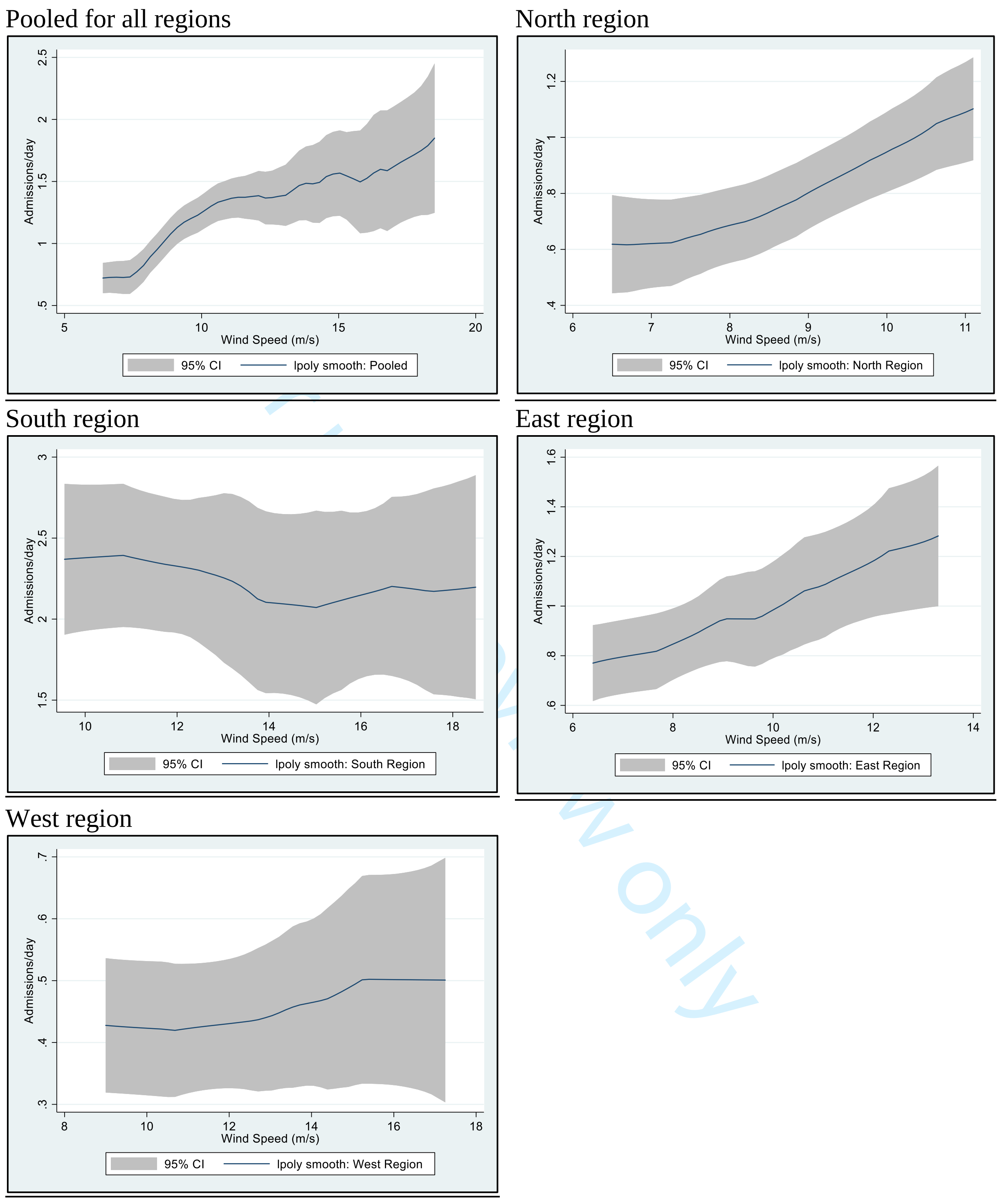

For peer review only - http://bmjopen.bmj.com/site/about/guidelines.xhtml

Supplementary Figure 7: Association of monthly intussusception cases with mean monthly sunshine

Pooled for all regions

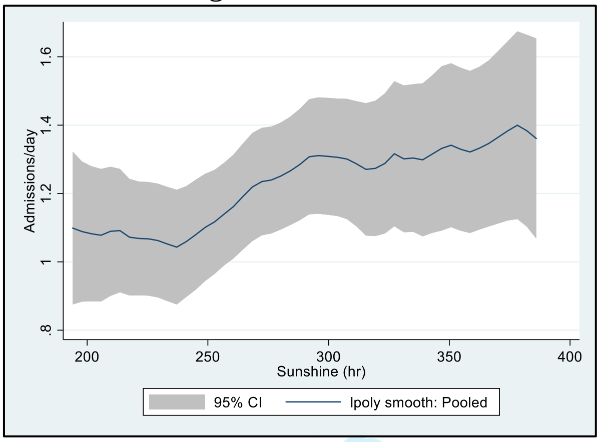

North region

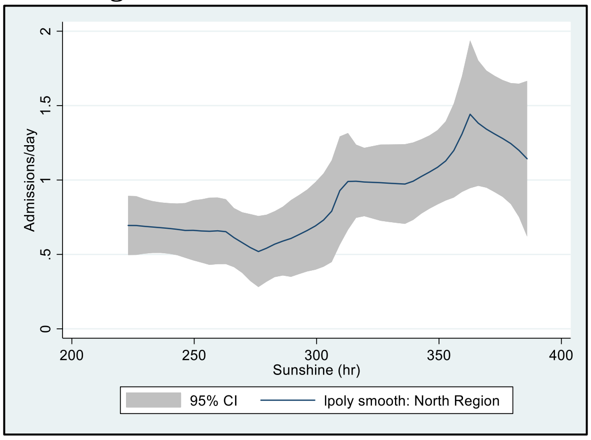

South region

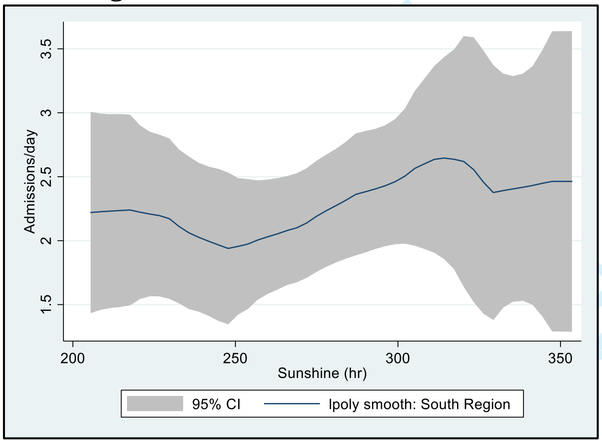

East region

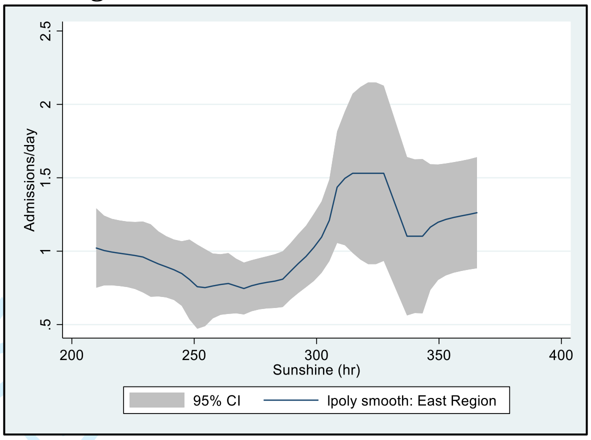

West region

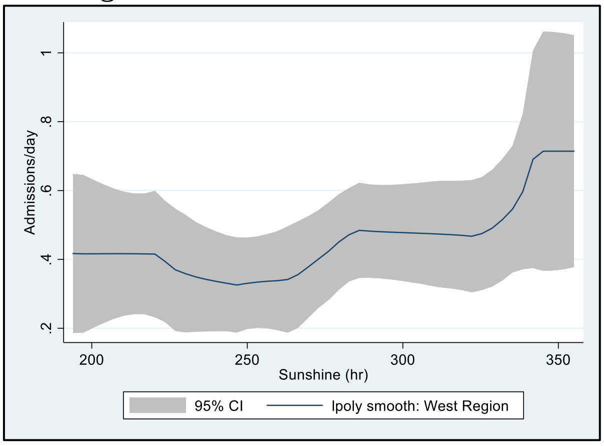

For peer review only - http://bmjopen.bmj.com/site/about/guidelines.xhtml

Supplementary Figure 8: Association of monthly intussusception cases with mean monthly rainfall

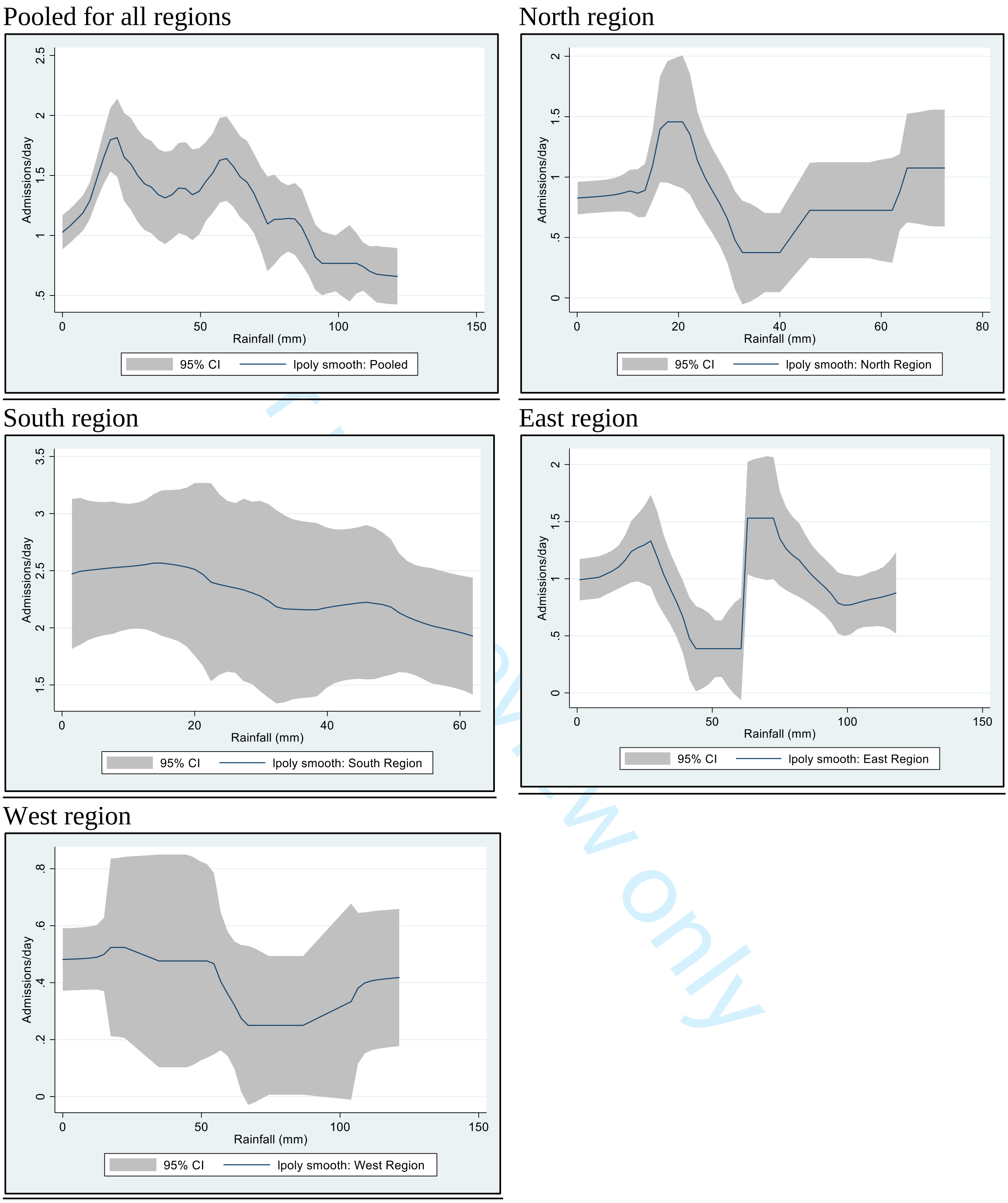

For peer review only

For peer review only - http://bmjopen.bmj.com/site/about/guidelines.xhtml

Supplementary Table 10. Factorial analysis-of-variance of intussusception in children with season and age groups

Source	Dependent variables	Type III Sum of squares	Degree of freedom	Mean square	F	p value
Corrected model	Seasonal IS cases	285.847[a]	38	7.522	1.442	**.040**
	Seasonal IS cases of 2-6 months	49.145[b]	38	1.293	1.579	**.014**
	Seasonal IS cases of 7-12 months	45.682[c]	38	1.202	1.012	.449
	Seasonal IS cases of 13-24 months	31.855[d]	38	.838	1.334	.085
Intercept	Seasonal IS cases	789.283	1	789.283	151.302	.000
	Seasonal IS cases of 2-6 months	101.336	1	101.336	123.760	.000
	Seasonal IS cases of 7-12 months	135.705	1	135.705	114.285	.000
	Seasonal IS cases of 13-24 months	40.684	1	40.684	64.740	.000
Season	Seasonal IS cases	8.674	4	2.169	.416	.797
	Seasonal IS cases of 2-6 months	1.930	4	.482	.589	.670
	Seasonal IS cases of 7-12 months	3.207	4	.802	.675	.609
	Seasonal IS cases of 13-24 months	.856	4	.214	.340	.851
Season year	Seasonal IS cases	82.483	7	11.783	2.259	**.027**
	Seasonal IS cases of 2-6 months	17.669	7	2.524	3.083	**.003**
	Seasonal IS cases of 7-12 months	3.504	7	.501	.422	.889
	Seasonal IS cases of 13-24 months	11.481	7	1.640	2.610	**.011**
Season* Season year	Seasonal IS cases	57.160	27	2.117	.406	.997
	Seasonal IS cases of 2-6 months	13.795	27	.511	.624	.934
	Seasonal IS cases of 7-12 months	19.521	27	.723	.609	.943
	Seasonal IS cases of 13-24 months	7.717	27	.286	.455	.993
Error	Seasonal IS cases	9092.547	1743	5.217		
	Seasonal IS cases of 2-6 months	1427.189	1743	.819		
	Seasonal IS cases of 7-12 months	2069.674	1743	1.187		
	Seasonal IS cases of 13-24 months	1095.327	1743	.628		
Total	Seasonal IS cases	11999.000	1782			
	Seasonal IS cases of 2-6 months	1816.000	1782			
	Seasonal IS cases of 7-12 months	2545.000	1782			
	Seasonal IS cases of 13-24 months	1272.000	1782			
Modified total	Seasonal IS cases	9378.393	1781			
	Seasonal IS cases of 2-6 months	1476.334	1781			
	Seasonal IS cases of 7-12 months	2115.356	1781			
	Seasonal IS cases of 13-24 months	1127.183	1781			

Note: a: R squared= .030 (adjusted R squared= .009); b: R squared= .033 (adjusted R squared= .012); c: R squared = .022 (adjusted R squared= .000); d: R squared= .028 (adjusted R squared= .007)

For peer review only - http://bmjopen.bmj.com/site/about/guidelines.xhtml

Supplementary Table 11. Factorial analysis-of-variance of intussusception in children with season and regions

Source	Dependent variables	Type III Sum of squares	Degree of freedom	Mean square	F	p value
Corrected model	Seasonal IS cases	285.847[a]	38	7.522	1.442	**.040**
	Seasonal IS cases of North region	36.237[b]	38	.954	1.809	**.002**
	Seasonal IS cases of South region	55.831[c]	38	1.469	.321	1.000
	Seasonal IS cases of East region	53.071[d]	38	1.397	1.333	.085
	Seasonal IS cases of West region	2.721[e]	38	.072	.662	.944
Intercept	Seasonal IS cases	789.283	1	789.283	151.302	**.000**
	Seasonal IS cases of North region	29.881	1	29.881	56.676	**.000**
	Seasonal IS cases of South region	180.247	1	180.247	39.373	**.000**
	Seasonal IS cases of East region	59.322	1	59.322	56.607	**.000**
	Seasonal IS cases of West region	2.250	1	2.250	20.798	**.000**
Season	Seasonal IS cases	8.674	4	2.169	.416	.797
	Seasonal IS cases of North region	10.274	4	2.569	4.872	**.001**
	Seasonal IS cases of South region	9.239	4	2.310	.505	.732
	Seasonal IS cases of East region	1.801	4	.450	.430	.787
	Seasonal IS cases of West region	.207	4	.052	.479	.752
Season year	Seasonal IS cases	82.483	7	11.783	2.259	**.027**
	Seasonal IS cases of North region	11.119	7	1.588	3.013	**.004**
	Seasonal IS cases of South region	5.618	7	.803	.175	.990
	Seasonal IS cases of East region	18.661	7	2.666	2.544	**.013**
	Seasonal IS cases of West region	.464	7	.066	.612	.746
Season* Season year	Seasonal IS cases	57.160	27	2.117	.406	.997
	Seasonal IS cases of North region	8.198	27	.304	.576	.960
	Seasonal IS cases of South region	28.377	27	1.051	.230	1.000
	Seasonal IS cases of East region	15.527	27	.575	.549	.971
	Seasonal IS cases of West region	1.325	27	.049	.454	.993
Error	Seasonal IS cases	9092.547	1743	5.217		
	Seasonal IS cases of North region	918.939	1743	.527		
	Seasonal IS cases of South region	7979.411	1743	4.578		
	Seasonal IS cases of East region	1826.595	1743	1.048		
	Seasonal IS cases of West region	188.597	1743	.108		
Total	Seasonal IS cases	11999.000	1782			
	Seasonal IS cases of North region	1032.000	1782			
	Seasonal IS cases of South region	8691.000	1782			
	Seasonal IS cases of East region	2077.000	1782			
	Seasonal IS cases of West region	199.000	1782			
Modified total	Seasonal IS cases	9378.393	1781			
	Seasonal IS cases of North region	955.176	1781			
	Seasonal IS cases of South region	8035.242	1781			
	Seasonal IS cases of East region	1879.666	1781			
	Seasonal IS cases of West region	191.318	1781			

Note: a: R squared = .030 (adjusted R squared= .009); b: R squared = .038 (adjusted R squared= .017); c: R squared = .007 (adjusted R squared= -.015); d: R squared= .028 (adjusted R squared= .007); e: R squared = .014 (adjusted R squared= -.007)

For peer review only - http://bmjopen.bmj.com/site/about/guidelines.xhtml

Supplementary Table 12. Multiple comparisons of seasonal intussusception in children in different seasons pooled for all sites

Dependent variable		Season (A)	Season (B)	Mean difference (A-B)	Standard error	p value	95% CI
Seasonal IS cases pooled	Tukey HSD	Monsoon	Spring	-.175	.2285	.940	-0.8 , 0.45
			Summer	-.409	.1643	.093	-0.86 , 0.04
			Monsoon	-.038	.1573	.999	-0.47 , 0.39
			Autumn	.160	.1758	.892	-0.32 , 0.64
		Spring	Winter	.175	.2285	.940	-0.45 , 0.8
			Summer	-.234	.2221	.831	-0.84 , 0.37
			Monsoon	.137	.2169	.970	-0.46 , 0.73
			Autumn	.336	.2307	.592	-0.29 , 0.97
		Summer	Winter	.409	.1643	.093	-0.04 , 0.86
			Spring	.234	.2221	.831	-0.37 , 0.84
			Monsoon	.371	.1478	.089	-0.03 , 0.77
			Autumn	.569*	.1673	**.006**	0.11 , 1.03
		Monsoon	Winter	.038	.1573	.999	-0.39 , 0.47
			Spring	-.137	.2169	.970	-0.73 , 0.46
			Summer	-.371	.1478	.089	-0.77 , 0.03
			Autumn	.199	.1604	.729	-0.24 , 0.64
		Autumn	Winter	-.160	.1758	.892	-0.64 , 0.32
			Spring	-.336	.2307	.592	-0.97 , 0.29
			Summer	-.569*	.1673	**.006**	-1.03 , -0.11
			Monsoon	-.199	.1604	.729	-0.64 , 0.24

*Note: * The difference is significant at $p<0.05$ level.*

For peer review only

For peer review only - http://bmjopen.bmj.com/site/about/guidelines.xhtml

Supplementary Table 13. Multiple comparisons of seasonal intussusception in children in different seasons at regional level

Dependent variable		Season (A)	Season (B)	Mean difference (A-B)	Standard error	p value	95% CI
North Region	Tukey HSD	Winter	Spring	-.151	.0726	.231	-0.35 , 0.05
			Summer	-.039	.0522	.943	-0.18 , 0.1
			Monsoon	.125	.0500	.089	-0.01 , 0.26
			Autumn	.139	.0559	.093	-0.01 , 0.29
		Spring	Winter	.151	.0726	.231	-0.05 , 0.35
			Summer	.111	.0706	.512	-0.08 , 0.3
			Monsoon	.276*	.0690	**.001**	0.09 , 0.46
			Autumn	.290*	.0733	**.001**	0.09 , 0.49
		Summer	Winter	.039	.0522	.943	-0.1 , 0.18
			Spring	-.111	.0706	.512	-0.3 , 0.08
			Monsoon	.165*	.0470	**.004**	0.04 , 0.29
			Autumn	.179*	.0532	**.007**	0.03 , 0.32
		Monsoon	Winter	-.125	.0500	.089	-0.26 , 0.01
			Spring	-.276*	.0690	**.001**	-0.46 , -0.09
			Summer	-.165*	.0470	**.004**	-0.29 , -0.04
			Autumn	.014	.0510	.999	-0.13 , 0.15
		Autumn	Winter	-.139	.0559	.093	-0.29 , 0.01
			Spring	-.290*	.0733	**.001**	-0.49 , -0.09
			Summer	-.179*	.0532	**.007**	-0.32 , -0.03
			Monsoon	-.014	.0510	.999	-0.15 , 0.13
South Region	Tukey HSD	Winter	Spring	-.014	.2140	1.000	-0.6 , 0.57
			Summer	-.197	.1539	.705	-0.62 , 0.22
			Monsoon	-.141	.1473	.875	-0.54 , 0.26
			Autumn	.032	.1647	1.000	-0.42 , 0.48
		Spring	Winter	.014	.2140	1.000	-0.57 , 0.6
			Summer	-.183	.2080	.905	-0.75 , 0.39
			Monsoon	-.127	.2032	.971	-0.68 , 0.43
			Autumn	.046	.2161	1.000	-0.54 , 0.64
		Summer	Winter	.197	.1539	.705	-0.22 , 0.62
			Spring	.183	.2080	.905	-0.39 , 0.75
			Monsoon	.056	.1384	.994	-0.32 , 0.43
			Autumn	.229	.1568	.588	-0.2 , 0.66
		Monsoon	Winter	.141	.1473	.875	-0.26 , 0.54
			Spring	.127	.2032	.971	-0.43 , 0.68
			Summer	-.056	.1384	.994	-0.43 , 0.32
			Autumn	.173	.1503	.779	-0.24 , 0.58
		Autumn	Winter	-.032	.1647	1.000	-0.48 , 0.42
			Spring	-.046	.2161	1.000	-0.64 , 0.54
			Summer	-.229	.1568	.588	-0.66 , 0.2
			Monsoon	-.173	.1503	.779	-0.58 , 0.24
East region	Tukey HSD	Winter	Spring	.035	.1024	.997	-0.24 , 0.31
			Summer	-.125	.0736	.433	-0.33 , 0.08
			Monsoon	-.001	.0705	1.000	-0.19 , 0.19

For peer review only - http://bmjopen.bmj.com/site/about/guidelines.xhtml

For peer review only

			Autumn	.010	.0788	1.000	-0.21 , 0.23
		Spring	Winter	-.035	.1024	.997	-0.31 , 0.24
			Summer	-.160	.0995	.490	-0.43 , 0.11
			Monsoon	-.036	.0972	.996	-0.3 , 0.23
			Autumn	-.025	.1034	.999	-0.31 , 0.26
		Summer	Winter	.125	.0736	.433	-0.08 , 0.33
			Spring	.160	.0995	.490	-0.11 , 0.43
			Monsoon	.124	.0662	.331	-0.06 , 0.31
			Autumn	.135	.0750	.372	-0.07 , 0.34
		Monsoon	Winter	.001	.0705	1.000	-0.19 , 0.19
			Spring	.036	.0972	.996	-0.23 , 0.3
			Summer	-.124	.0662	.331	-0.31 , 0.06
			Autumn	.011	.0719	1.000	-0.19 , 0.21
		Autumn	Winter	-.010	.0788	1.000	-0.23 , 0.21
			Spring	.025	.1034	.999	-0.26 , 0.31
			Summer	-.135	.0750	.372	-0.34 , 0.07
			Monsoon	-.011	.0719	1.000	-0.21 , 0.19
West region	Tukey HSD	Winter	Spring	-.046	.0329	.637	-0.14 , 0.04
			Summer	-.048	.0237	.260	-0.11 , 0.02
			Monsoon	-.022	.0227	.872	-0.08 , 0.04
			Autumn	-.021	.0253	.921	-0.09 , 0.05
		Spring	Winter	.046	.0329	.637	-0.04 , 0.14
			Summer	-.002	.0320	1.000	-0.09 , 0.09
			Monsoon	.024	.0312	.941	-0.06 , 0.11
			Autumn	.025	.0332	.947	-0.07 , 0.12
		Summer	Winter	.048	.0237	.260	-0.02 , 0.11
			Spring	.002	.0320	1.000	-0.09 , 0.09
			Monsoon	.026	.0213	.743	-0.03 , 0.08
			Autumn	.027	.0241	.805	-0.04 , 0.09
		Monsoon	Winter	.022	.0227	.872	-0.04 , 0.08
			Spring	-.024	.0312	.941	-0.11 , 0.06
			Summer	-.026	.0213	.743	-0.08 , 0.03
			Autumn	.001	.0231	1.000	-0.06 , 0.06
		Autumn	Winter	.021	.0253	.921	-0.05 , 0.09
			Spring	-.025	.0332	.947	-0.12 , 0.07
			Summer	-.027	.0241	.805	-0.09 , 0.04
			Monsoon	-.001	.0231	1.000	-0.06 , 0.06

*Note: * The difference is significant at p<0.05 level.*

For peer review only - http://bmjopen.bmj.com/site/about/guidelines.xhtml

Supplementary Table 14. Multiple comparisons of seasonal intussusception in children in different seasons for different age groups

Dependent variable		Season (A)	Season (B)	Mean difference (A-B)	Standard error	p value	95% CI
Seasonal IS cases in children aged 2-6 months	Tukey HSD	Winter	Spring	-.003	.0905	1.000	-0.25 , 0.24
			Summer	-.073	.0651	.798	-0.25 , 0.11
			Monsoon	.019	.0623	.998	-0.15 , 0.19
			Autumn	.123	.0696	.395	-0.07 , 0.31
		Spring	Winter	.003	.0905	1.000	-0.24 , 0.25
			Summer	-.070	.0880	.933	-0.31 , 0.17
			Monsoon	.022	.0859	.999	-0.21 , 0.26
			Autumn	.126	.0914	.643	-0.12 , 0.38
		Summer	Winter	.073	.0651	.798	-0.11 , 0.25
			Spring	.070	.0880	.933	-0.17 , 0.31
			Monsoon	.092	.0585	.517	-0.07 , 0.25
			Autumn	.195*	.0663	**.027**	0.01 , 0.38
		Monsoon	Winter	-.019	.0623	.998	-0.19 , 0.15
			Spring	-.022	.0859	.999	-0.26 , 0.21
			Summer	-.092	.0585	.517	-0.25 , 0.07
			Autumn	.104	.0636	.478	-0.07 , 0.28
		Autumn	Winter	-.123	.0696	.395	-0.31 , 0.07
			Spring	-.126	.0914	.643	-0.38 , 0.12
			Summer	-.195*	.0663	**.027**	-0.38 , -0.01
			Monsoon	-.104	.0636	.478	-0.28 , 0.07
Seasonal IS cases in children aged 7-12 months	Tukey HSD	Winter	Spring	-.185	.1090	.439	-0.48 , 0.11
			Summer	-.210	.0784	.057	-0.42 , 0
			Monsoon	-.046	.0750	.973	-0.25 , 0.16
			Autumn	.021	.0839	.999	-0.21 , 0.25
		Spring	Winter	.185	.1090	.439	-0.11 , 0.48
			Summer	-.026	.1059	.999	-0.32 , 0.26
			Monsoon	.138	.1035	.667	-0.14 , 0.42
			Autumn	.206	.1101	.336	-0.1 , 0.51
		Summer	Winter	.210	.0784	.057	0 , 0.42
			Spring	.026	.1059	.999	-0.26 , 0.32
			Monsoon	.164	.0705	.136	-0.03 , 0.36
			Autumn	.231*	.0798	**.031**	0.01 , 0.45
		Monsoon	Winter	.046	.0750	.973	-0.16 , 0.25
			Spring	-.138	.1035	.667	-0.42 , 0.14
			Summer	-.164	.0705	.136	-0.36 , 0.03
			Autumn	.067	.0765	.906	-0.14 , 0.28
		Autumn	Winter	-.021	.0839	.999	-0.25 , 0.21
			Spring	-.206	.1101	.336	-0.51 , 0.1
			Summer	-.231*	.0798	**.031**	-0.45 , -0.01
			Monsoon	-.067	.0765	.906	-0.28 , 0.14
Seasonal IS cases in	Tukey HSD	Winter	Spring	.012	.0793	1.000	-0.2 , 0.23
			Summer	-.126	.0570	.176	-0.28 , 0.03
			Monsoon	-.011	.0546	1.000	-0.16 , 0.14

For peer review only

For peer review only - http://bmjopen.bmj.com/site/about/guidelines.xhtml

children aged 7-12 months			Autumn	.016	.0610	.999	-0.15 , 0.18
		Spring	Winter	-.012	.0793	1.000	-0.23 , 0.2
			Summer	-.138	.0771	.378	-0.35 , 0.07
			Monsoon	-.024	.0753	.998	-0.23 , 0.18
			Autumn	.004	.0801	1.000	-0.21 , 0.22
		Summer	Winter	.126	.0570	.176	-0.03 , 0.28
			Spring	.138	.0771	.378	-0.07 , 0.35
			Monsoon	.115	.0513	.167	-0.03 , 0.25
			Autumn	.143	.0581	.102	-0.02 , 0.3
		Monsoon	Winter	.011	.0546	1.000	-0.14 , 0.16
			Spring	.024	.0753	.998	-0.18 , 0.23
			Summer	-.115	.0513	.167	-0.25 , 0.03
			Autumn	.028	.0557	.987	-0.12 , 0.18
		Autumn	Winter	-.016	.0610	.999	-0.18 , 0.15
			Spring	-.004	.0801	1.000	-0.22 , 0.21
			Summer	-.143	.0581	.102	-0.3 , 0.02
			Monsoon	-.028	.0557	.987	-0.18 , 0.12

*Note: * The difference is significant at p<0.05 level.*

For peer review only

For peer review only - http://bmjopen.bmj.com/site/about/guidelines.xhtml

Revised Title: Association of meteorological parameters with intussusception in children aged under-two years: Results from a multisite bidirectional surveillance over seven years in India

Manuscript ID: bmjopen-2020-043687.R2

Corresponding author: Manoja Kumar Das, MD

The Institute Ethics Committees (IECs) of all the participating institutes reviewed and approved the study protocol titled "Intussusception in Infants: Multisite Retrospective Surveillance in India". The list of IECs reviewed and approved the protocol with the reference numbers for the respective IECs are listed below.

1. Name of IRB: The INCLEN Independent Ethic Committee
   IRB Ref no: IIEC-023
   Name of the Institute: The INCLEN Trust International, New Delhi, India
   Principal Investigator's Institute/Coordinating Institute

2. Name of IRB: Institutional Ethics Committee
   IRB Ref no: IEC/SKIMS Protocol#42/2015
   Name of the Institute: Sher-I-Kashmir Institute of Medical Sciences, Srinagar, Jammu & Kashmir, India

3. Name of IRB: Institutional Ethics Committee
   IRB Ref no: IEC/MAMC/50/4/2015/308
   Name of the Institute: Maulana Azad Medical College, Delhi, India

4. Name of IRB: Medanta Institutional Ethics Committee
   IRB Ref no: MICR 559/2015
   Name of the Institute: Medanta- The Medicity, Gurgaon, Haryana, India

5. Name of IRB: The INCLEN Independent Ethic Committee
   IRB Ref no: 7951/Ethics/R.Cell-15
   Name of the Institute: King George's Medical University, Lucknow, Uttar Pradesh, India

6. Name of IRB: Institutional Ethics Committee
   IRB Ref no: EC/Oct/15/20
   Name of the Institute: Choithram Hospital and Research Centre, Indore, Madhya Pradesh, India

7. Name of IRB: Institutional Ethics Committee
   IRB Ref no: 26/12/2015
   Name of the Institute: King George Hospital, Andhra Medical College, Vishakhapatnam, Andhra Pradesh, India

8. Name of IRB: Ethics Committee
   IRB Ref no: 13/12/2015
   Name of the Institute: Apollo Hospital, Hyderabad, Telengana, India

9. Name of IRB: Institutional Human Ethics Committee

For peer review only - http://bmjopen.bmj.com/site/about/guidelines.xhtml

For peer review only

IRB Ref no: Project No 15/294
Name of the Institute: PSG Institute of Medical Sciences, Coimbatore, Tamil Nadu, India

10. Name of IRB: Institutional Ethics Committee- Clinical Studies
IRB Ref no: 14/12/210
Name of the Institute: Apollo Hospitals, Chennai, Tamil Nadu

11. Name of IRB: Institutional Ethics Committee
IRB Ref no: IEC-06/05/2015/MCT
Name of the Institute: Government Medical College & SAT Hospital, Thiruvananthapuram, Kerala, India

12. Name of IRB: Institutional Ethics Committee
IRB Ref no: 1256/Acad
Name of the Institute: Indira Gandhi Institute of Medical Sciences, Patna, Bihar, India

13. Name of IRB: Institutional Ethics Committee
IRB Ref no: 210/5/10/2015
Name of the Institute: IMS & SUM Medical College & Hospital, Bhubaneswar, Odisha, India

14. Name of IRB: Institutional Ethics Committee
IRB Ref no: 311/27-04-16
Name of the Institute: SCB Medical College, Cuttack, Odisha, India

15. Name of IRB: Institutional Ethics Committee
IRB Ref no: 210/5/10/2015
Name of the Institute: MKCG Medical College, Berhampur, Odisha, India

16. Name of IRB: Institutional Ethics Committee
IRB Ref no: IEC/2016/197
Name of the Institute: Institute of Post Graduate Medical Education and Research, Kolkata, West Bengal, India

17. Name of IRB: Institutional Ethics Committee
IRB Ref no: MC/02/2015/274
Name of the Institute: Gauhati Medical College, Guwahati, Assam, India

18. Name of IRB: Institutional Ethics Committee
IRB Ref no: 2015/3894
Name of the Institute: Agartala Government Medical College, Agartala, Tripura, India

19. Name of IRB: Institutional Ethics Committee
IRB Ref no: FEHJ/IEC/15/0023
Name of the Institute: Fortis Escorts Hospital, Jaipur, Rajasthan, India

20. Name of IRB: Institutional Ethics Committee
IRB Ref no: IEC/46/2016
Name of the Institute: MP Shah Government Medical College, Jamnagar, Gujarat, India

21. Name of IRB: Institutional Ethics Committee

For peer review only - http://bmjopen.bmj.com/site/about/guidelines.xhtml

IRB Ref no: IEC/Pharm/288/15
Name of the Institute: Grant Medical College & JJ Hospital, Mumbai, Maharashtra, India

For peer review only

For peer review only - http://bmjopen.bmj.com/site/about/guidelines.xhtml

Manuscript Title: Intussusception in children under-two years, seasonal variations and associations with meteorological parameters: Results from multisite bidirectional surveillance in India over eight years

STROBES Checklist

Section/Topic	Item #	Recommendation	Reported on page #
**Title and abstract**	1	(*a*) Indicate the study's design with a commonly used term in the title or the abstract	1
		(*b*) Provide in the abstract an informative and balanced summary of what was done and what was found	1-2
**Introduction**			
Background/rationale	2	Explain the scientific background and rationale for the investigation being reported	4
Objectives	3	State specific objectives, including any pre-specified hypotheses	5
**Methods**			
Study design	4	Present key elements of study design early in the paper	5
Setting	5	Describe the setting, locations, and relevant dates, including periods of recruitment, exposure, follow-up, and data collection	5
Participants	6	(*a*) *Cohort study*—Give the eligibility criteria, and the sources and methods of selection of participants. Describe methods of follow-up<br>*Case-control study*—Give the eligibility criteria, and the sources and methods of case ascertainment and control selection. Give the rationale for the choice of cases and controls<br>*Cross-sectional study*—Give the eligibility criteria, and the sources and methods of selection of participants	5
		(*b*) *Cohort study*—For matched studies, give matching criteria and number of exposed and unexposed<br>*Case-control study*—For matched studies, give matching criteria and the number of controls per case	
Variables	7	Clearly define all outcomes, exposures, predictors, potential confounders, and effect modifiers. Give diagnostic criteria, if applicable	5-6
Data sources/ measurement	8*	For each variable of interest, give sources of data and details of methods of assessment (measurement). Describe comparability of assessment methods if there is more than one group	5-6
Bias	9	Describe any efforts to address potential sources of bias	6

For peer review only

Study size	10	Explain how the study size was arrived at	5-6
Quantitative variables	11	Explain how quantitative variables were handled in the analyses. If applicable, describe which groupings were chosen and why	6
Statistical methods	12	(*a*) Describe all statistical methods, including those used to control for confounding	6-7
		(*b*) Describe any methods used to examine subgroups and interactions	6-7
		(*c*) Explain how missing data were addressed	6
		(*d*) *Cohort study*—If applicable, explain how loss to follow-up was addressed<br>*Case-control study*—If applicable, explain how matching of cases and controls was addressed<br>*Cross-sectional study*—If applicable, describe analytical methods taking account of sampling strategy	6-7
		(*e*) Describe any sensitivity analyses	6-7
**Results**			
Participants	13*	(a) Report numbers of individuals at each stage of study—eg numbers potentially eligible, examined for eligibility, confirmed eligible, included in the study, completing follow-up, and analysed	7
		(b) Give reasons for non-participation at each stage	NA
		(c) Consider use of a flow diagram	Narration given
Descriptive data	14*	(a) Give characteristics of study participants (eg demographic, clinical, social) and information on exposures and potential confounders	7-8
		(b) Indicate number of participants with missing data for each variable of interest	7-8 and tables
		(c) *Cohort study*—Summarise follow-up time (eg, average and total amount)	
Outcome data	15*	*Cohort study*—Report numbers of outcome events or summary measures over time	7-11<br>Given in tables and results
		Case-control study—Report numbers in each exposure category, or summary measures of exposure	
		Cross-sectional study—Report numbers of outcome events or summary measures	
Main results	16	(*a*) Give unadjusted estimates and, if applicable, confounder-adjusted estimates and their precision (eg, 95% confidence interval). Make clear which confounders were adjusted for and why they were included	7-11<br>Given in tables and results
		(*b*) Report category boundaries when continuous variables were categorized	7-11<br>Given in tables and

For peer review only

			results
		(*c*) If relevant, consider translating estimates of relative risk into absolute risk for a meaningful time period	NA
Other analyses	17	Report other analyses done—eg analyses of subgroups and interactions, and sensitivity analyses	7-11 Given in tables and results
**Discussion**			
Key results	18	Summarise key results with reference to study objectives	11-12
Limitations	19	Discuss limitations of the study, taking into account sources of potential bias or imprecision. Discuss both direction and magnitude of any potential bias	14
Interpretation	20	Give a cautious overall interpretation of results considering objectives, limitations, multiplicity of analyses, results from similar studies, and other relevant evidence	11-14
Generalisability	21	Discuss the generalisability (external validity) of the study results	14
**Other information**			
Funding	22	Give the source of funding and the role of the funders for the present study and, if applicable, for the original study on which the present article is based	17

*Give information separately for cases and controls in case-control studies and, if applicable, for exposed and unexposed groups in cohort and cross-sectional studies.

Note: An Explanation and Elaboration article discusses each checklist item and gives methodological background and published examples of transparent reporting. The STROBE checklist is best used in conjunction with this article (freely available on the Web sites of PLoS Medicine at http://www.plosmedicine.org/, Annals of Internal Medicine at http://www.annals.org/, and Epidemiology at http://www.epidem.com/). Information on the STROBE Initiative is available at www.strobe-statement.org.

For peer review only
